# NextTopDocker: A Large-Scale Docking-Power Benchmark Reveals Limitations of Current End-to-End Machine-Learning Docking and the Strength of Hybrid Rescoring

**DOI:** 10.1021/acs.jmedchem.6c01143

**Published:** 2026-08-06

**Authors:** Cao-Minh Truong, Pedro J. Ballester, Olivier Taboureau, Viet-Khoa Tran-Nguyen

**Affiliations:** † Université Paris Cité, CNRS UMR 8251, INSERM ERL 1133, F-75013 Paris, France; ‡ Department of Bioengineering, 4615 Imperial College London, London SW7 2AZ, U.K.

## Abstract

Predicting three-dimensional binding orientations of drug-like molecules remains challenging in structure-based drug design. Despite methodological advances, docking performance is often assessed on small and outdated benchmarks. We present “NextTopDocker,” a large, up-to-date, open-access data set for docking-power assessment comprising 14,038 training and 5201 test entries across 3173 unique protein targets, constructed from the Protein Data Bank. Developed with open-source tools, it includes crystallographic structures, Smina-generated docking poses, and ligand-similarity-aware training subsets. We benchmarked four state-of-the-art machine-learning (ML) docking frameworks (DeepDock, Interformer, SurfDock, and Uni-Mol Docking v.2) against classical (Smina) and hybrid baselines (GNINA 1.3 and logistic regression using Smina and GNINA 1.3 scores). Interformer alone matched the docking power of logistic regression on Smina poses, while the others showed dependence on downstream physics-based correction. Most raw ML-generated poses displayed steric clashes and/or implausible geometries, highlighting the need for physics-informed constraints in autonomous docking. “NextTopDocker” is available at https://github.com/caominhtr/NextTopDocker and https://zenodo.org/records/17492994.

## Graphical abstract

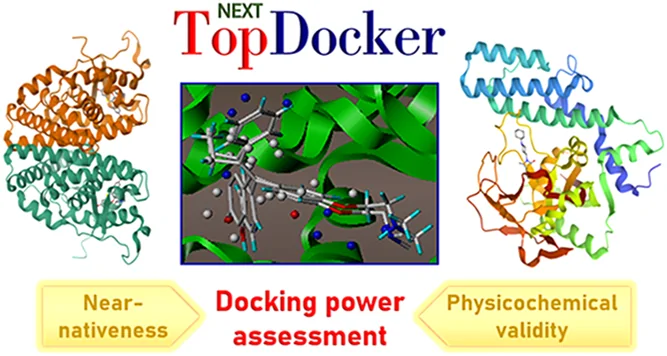

## Introduction

Determining the three-dimensional (3D) binding orientation of a drug-like molecule and the molecular interactions stabilizing its complex with a receptor remains a central challenge in structure-based (SB) drug discovery.^1–4^ It represents the first and most critical stage of virtual screening (VS) workflows aimed at identifying bioactive candidates. Over the past decades, numerous computational strategies, most notably molecular docking, have been developed to predict plausible ligand binding conformations.^5,6^ These approaches rely on pose sampling algorithms that explore ligand conformational and orientational space, with or without receptor flexibility, thus enabling rigid-body or flexible docking schemes. Sampling strategies range from shape matching and incremental construction to stochastic searches such as Monte Carlo or simulated annealing.^5^ Historically, such methods have been able to recover at least one near-native binding pose, defined as a conformation within 2-Å root-mean-square deviation (RMSD) of the experimentally determined structure.^2^ Following pose generation, pose scoring is performed to evaluate each ligand–receptor complex. Classical scoring functions (SFs), whether physics-based, knowledge-based, or empirical, serve this purpose but frequently do not prioritize native-like docking poses, as shown in retrospective evaluations.^7–10^ To address these limitations, machine-learning (ML)-based SFs have emerged, leveraging data-driven approaches to rescore docking output. Examples include RF-Score-VS,^11^ the GNINA convolutional neural network (CNN)-based SF and its successors,^12,13^ as well as ClassyPose,^10^ all of which have demonstrated superior docking power compared with traditional, fully non-ML docking protocols combining conventional pose sampling and pose scoring.

Since the introduction of AlphaFold,^14^ a new generation of end-to-end ML docking tools has emerged, integrating ML for both pose sampling and scoring. Among the early entrants, DeepDock (2021) applies geometric deep learning (DL) to predict ligand binding conformations: it learns a tailored statistical potential based on distance likelihoods for each ligand-target pair, thereby combining statistical modeling with SB reasoning.^15^ In the subsequent years, especially following AlphaFold 3 and its related innovations (e.g., Boltz-1, Boltz-2),^16–18^ several more ML docking methods have appeared. In November 2024, Interformer was released: it uses a graph-transformer backbone to model noncovalent interactions via an interaction-aware mixture density network and incorporates a negative sampling strategy to improve the predicted distribution of interactions across ligand-protein pairs.^19^ In 2025, SurfDock and Uni-Mol Docking v.2 further extended the field.^20,21^ SurfDock integrates protein sequence, 3D structure and surface features within an equivariant architecture, and uses a diffusion model on a non-Euclidean manifold to optimize translation, rotation and torsion for reliable binding pose generation.^20^ Uni-Mol Docking v.2 is an upgraded version of Uni-Mol Docking, a universal molecular representation learning framework.^21^ It uses a transformer architecture equivariant to 3D rotations and translations, shared by its molecular and pocket pretraining branches, enhancing practical representation capacity.^21^

However, the literature has raised concerns about the chemical and physical plausibility of poses generated by such ML docking methods.^22–24^ The seminal PoseBusters study, in particular, argued that artificial intelligence (AI)-based pose generators do not always produce physically valid conformations or generalize well to novel proteins.^22^ Moreover, the docking power of these tools has typically been benchmarked on small and somewhat outdated data sets such as the Astex Diverse set (85 entries),^25^ CASF-2016 (285 entries),^26^ PDBbind core sets (a few hundred entries),^27^ and PoseBusters (308 entries).^22^ Larger and more recent benchmarks like PDBbind are not fully open-access: as of August 2025, the latest freely available version is PDBbind v.2020R1, which was last updated in 2020 and lacks recent Protein Data Bank (PDB) entries. Furthermore, many PDBbind entries were processed using proprietary software (e.g., Sybyl),^27^ which limits reproducibility and free reprocessing by the community.

In light of these limitations, our study has four main objectives:

(i) To construct a large-scale and meticulously curated open-access benchmarking data set of protein–ligand complexes retrieved from the PDB (as of May 2025) for docking-power assessment, using exclusively open-source tools. The data set was partitioned into a training set (over 14,000 entries) and a test set (over 5000 entries) via a strict cold-ligand split (no duplicating ligands across the two partitions), and further divided into nine training subsets following a ligand-similarity-aware scheme, enabling both performance evaluation and future training of ML-based docking/scoring tools;
(ii) To benchmark the docking power of four state-of-the-art (SOTA) end-to-end ML docking tools, including DeepDock, Interformer, SurfDock, and Uni-Mol Docking v.2, on our test set. This represents the first joint assessment of their docking performance on such extensive test data;
(iii) To compare these ML methods against more traditional or hybrid approaches: pure non-ML docking (Smina),^28^ CNN-based rescoring of Smina poses (GNINA 1.3),^13^ and logistic regression models trained on Smina and GNINA 1.3 scores across our training partitions. Docking performance was assessed using the RMSD of the top-ranked pose per PDB entry, together with PoseBusters-based physicochemical plausibility evaluation.^22^ Beyond the main training-test framework, additional stringent evaluation scenarios were also considered;
(iv) To reveal the limitations of these ML docking programs, particularly in their intrinsic pose sampling capability, by explicitly excluding any postgeneration physics-guided or non-ML optimization (e.g., energy minimization), ensuring that performance reflects the true generative power of their ML sampling algorithms.

All data and code are available on GitHub (https://github.com/caominhtr/NextTopDocker) and Zenodo (https://zenodo.org/records/17492994). Additionally, details on the training data of each of the four evaluated ML docking tools are provided in the Supporting Information (ListsS1–S4) to facilitate potential unbiased retraining and independent evaluation of these frameworks in future work.

## Results and Discussion

### Computational Workflow

The computational workflow of our study is outlined in Figure 1. First, a list of experimentally resolved protein–ligand complex structures was retrieved from the PDB using the “Advanced Search” option, combined with in-house filtering rules (May 2025). These rules primarily assessed the structural quality of the complexes as well as key parameters related to the drug-likeness of the cocrystallized small molecules (cf. “Methods section”). Selected PDB entries were prepared using custom Python and Bash pipelines, incorporating ChimeraX (v.1.8) and PyMOL (v.2.5), to allow robust and fully automated large-scale curation. The resulting data set provides: (i) a comprehensive, machine-readable data table (csv) for downstream analysis, and (ii) thoroughly prepared protein (mol2) and ligand (mol2 and sdf) files, all chemically and structurally validated and directly ready for computational use. A new benchmark for docking-power evaluation was then established as follows. The curated PDB entries were first assigned to either a training set or a test set using a strict cold-ligand split, which excluded any overlap of crystallographic ligands across partitions. This split considered both ligand HET codes and 2D molecular similarity, thereby eliminating potential data leakage. To facilitate model training not only on the full training corpus but also under progressively harder generalization scenarios, the training data were further divided into nine progressively smaller subsets. These subsets were constructed using a ligand-similarity-aware design, whereby training PDB entries containing ligands with higher maximum similarity to test-set ligands were progressively excluded, yielding subsets of decreasing size and increasing dissimilarity relative to the test set. Redocking of each cocrystallized ligand into its corresponding binding pocket was then performed (cf. “Methods section”). We employed both Smina (v.2019–10–15), an enhanced fork of AutoDock Vina that relies on non-ML sampling algorithms,^28^ and several ML docking tools, namely DeepDock,^15^ Interformer,^19^ SurfDock,^20^ and Uni-Mol Docking v.2.^21^ Docking pose rescoring was conducted on Smina-generated redocked poses using GNINA 1.3, a CNN-based SF,^13^ across both training and test sets. Importantly, no postsampling energy minimization or clash-correction options were enabled during docking and scoring runs, including Smina/GNINA 1.3 “minimize” and Uni-Mol Docking v.2 “steric-clash-fix.” Pose sampling parameters (such as Smina “exhaustiveness”) were kept at default settings, and no structural relaxation using external tools or third-party pipelines was performed. Using the Smina docking scores and GNINA 1.3 CNN scores of redocked poses from the training set and each of its nine subsets as features, after standardization with scikit-learn’s “StandardScaler” (v.1.6.1), we trained a series of 10 logistic regression classifiers. Each model was designed to discriminate near-native from non-native poses (defined by an RMSD threshold of 2 Å, cf. “Methods section”). Throughout this study, “logistic regression” refers to GNINA/Smina-score-based rescoring of Smina-generated docking poses within this hybrid docking-scoring workflow.

**Figure 1:**
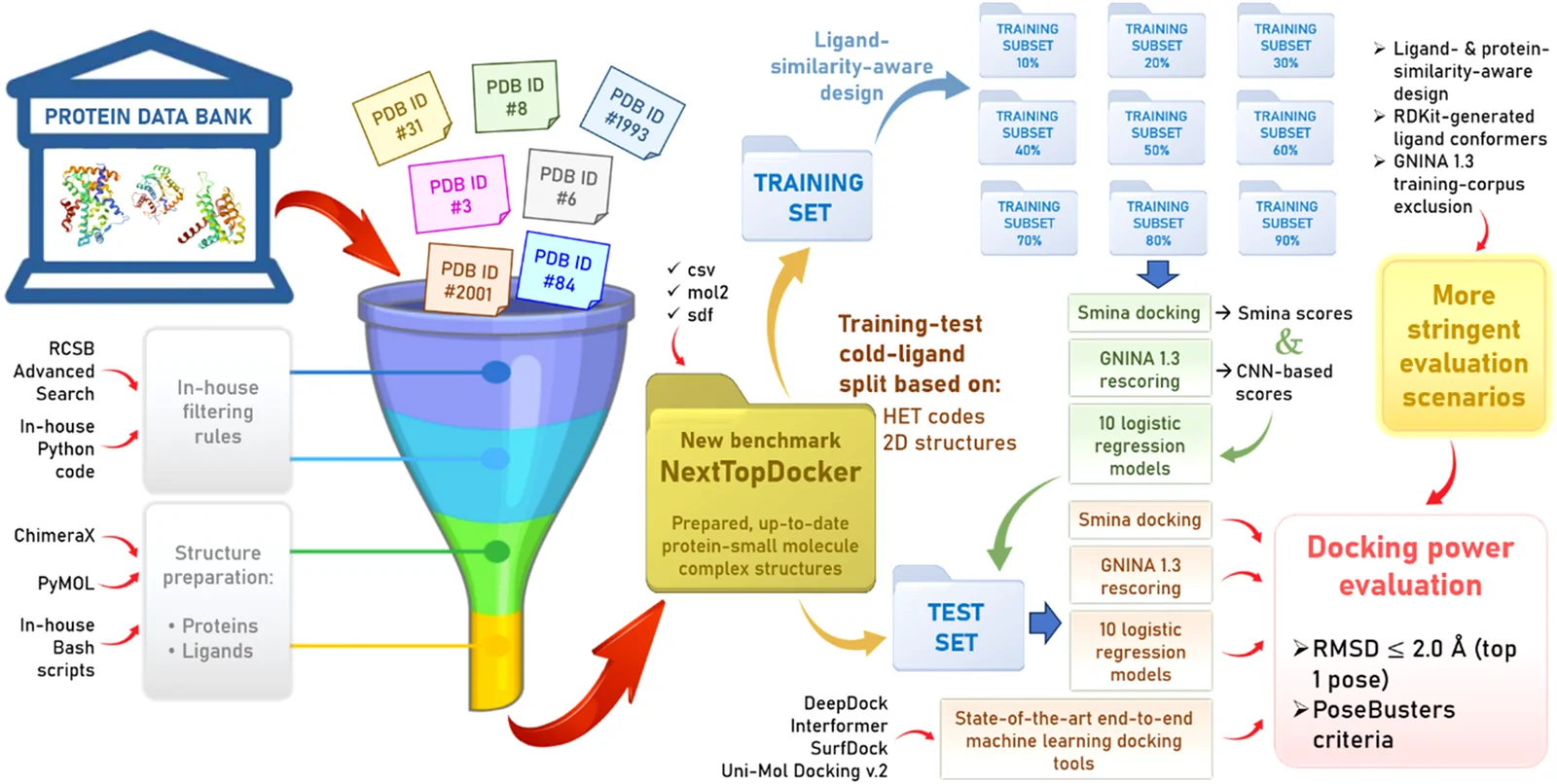
Computational workflow of the NextTopDocker benchmark. Protein–ligand complexes were retrieved from the PDB, curated using open-source tools, split into cold-ligand training and test sets, and used to evaluate Smina, GNINA 1.3 rescoring, logistic regression rescoring, and four end-to-end ML docking frameworks. Performance was assessed using top-ranked pose RMSD and PoseBusters validity.

Docking power was finally evaluated on the test set by comparing: (i) raw Smina docking scores, (ii) GNINA 1.3 CNN-based scores, (iii) the 10 logistic regression models trained on the training set and its subsets, and (iv) the four current ML docking tools. Evaluation criteria included RMSD of top-ranked poses relative to the crystallographic reference and PoseBusters compliance.^22^ To further assess robustness, more stringent settings were considered, including ligand- and protein-similarity-aware splits, RDKit-derived ligand starting conformations, and exclusion of test entries associated with the GNINA 1.3 training corpus. All source code and input/output data, including the full benchmark (prepared structures, docking output, and derived features), are freely available in our GitHub repository and on Zenodo (cf. “Data and Software Availability”).

### Data Retrieval, Data Curation and Stratified Data Split

Our final data set comprised 19,239 protein–ligand complexes retrieved, curated, and prepared from the PDB (cf. “Methods section”). It exhibited high diversity in terms of cocrystallized ligands and protein targets, as well as the functional families they represented.

In total, 13,750 unique ligands (distinct HET codes) were included. These ligands showed substantial structural diversity: among the 94,524,375 possible ligand pairs, only 1.85% had Tanimoto similarity coefficients (Morgan fingerprints, 2,048 bits, radius 2) greater than 0.2. The pairwise similarity distribution had a mean of 0.1105, further confirming the overall structural dissimilarity within the ligand set (Figure S1, Supporting Information). Moreover, these ligands spanned a broad chemical space, as reflected by the value ranges of 11 computed physicochemical properties (Figure S2, Supporting Information), while also satisfying an extended set of drug-likeness criteria (cf. “Methods section”).

Additionally, 3,173 unique protein targets, i.e., distinct UniProt identifiers (IDs) were included and grouped into 261 functional families based on UniProt classification, an approach widely accepted for protein family assignment (Figure S3, Supporting Information). The most represented families were transferases (24.98%), hydrolases (21.10%), oxidoreductases (11.47%), and transcription factors (5.22%), each accounting for over 1,000 complexes. The most frequent targets were 3C-like proteinase (P0DTD1, 596 entries), carbonic anhydrase II (P00918, 331 entries), and bromodomain-containing protein 4 (O60885, 301 entries).

A strict cold-ligand split, considering both ligand HET codes and 2D molecular similarity constraints, was applied to divide the data set into training (14,038 PDB entries) and test (5201 PDB entries) sets (cf. “Methods section”). The ligands across these two partitions were structurally dissimilar: among 36,683,616 ligand pairs that could be formed between the training (*n* = 10,128) and test (*n* = 3622) ligands, only 1.71% had Tanimoto similarity scores (Morgan fingerprints, 2048 bits, radius 2) above 0.2 (Figure 2A). The highest Tanimoto similarity of a training ligand to any test ligand was centered around 0.3753 and did not exceed 0.99 (Figure 2B), confirming minimal risk of data leakage or bias between the two sets.

**Figure 2:**
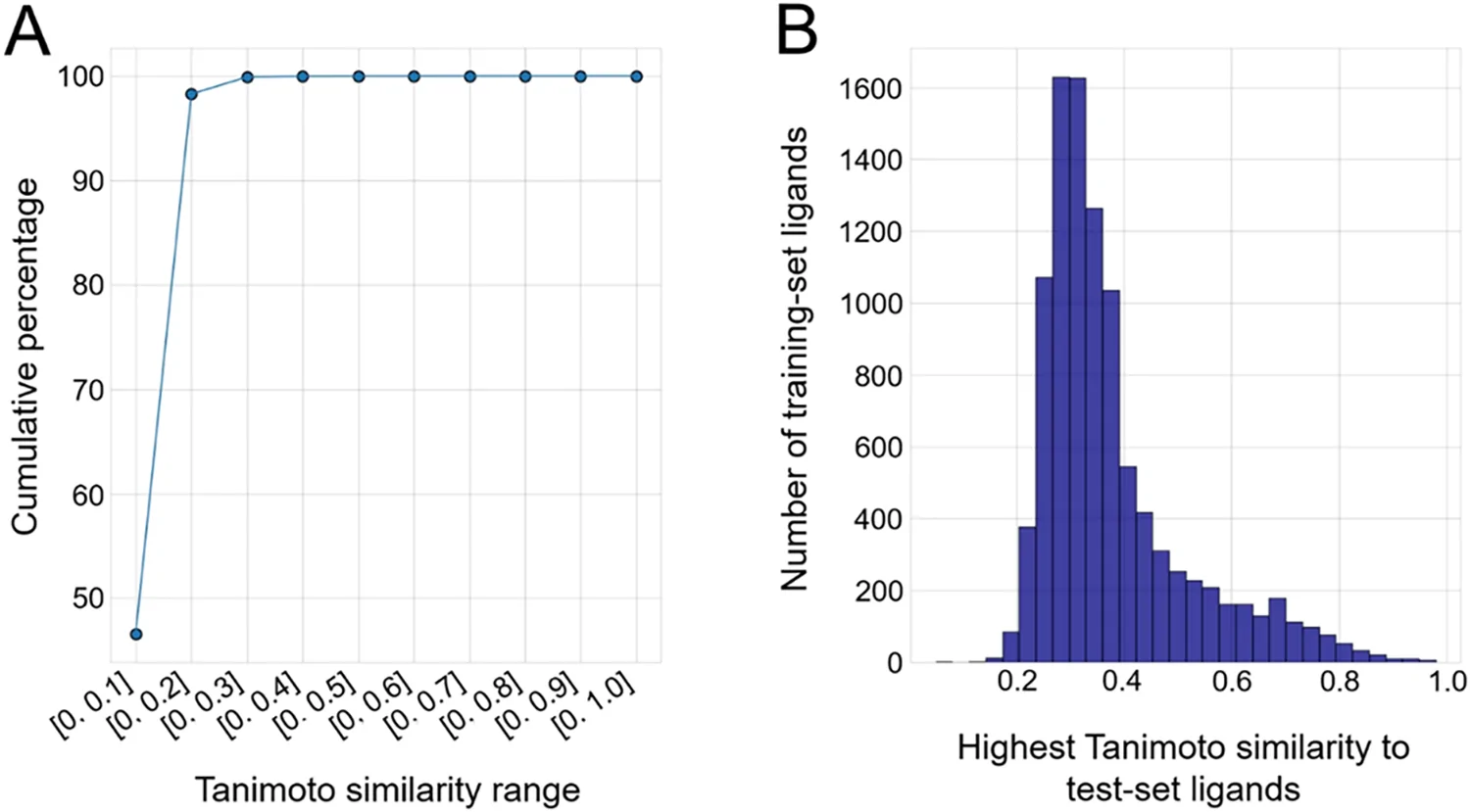
Structural dissimilarity between training-set and test-set ligands. (A) Cumulative percentage of training-set and test-set ligand pairs whose Tanimoto similarity coefficients (Morgan fingerprints, 2048 bits, radius 2) fell within defined ranges. (B) Distribution of the highest Tanimoto similarity coefficient of each training-set ligand to any ligand in the test set.

To further assess generalization under increasingly stringent conditions and to simulate realistic low-similarity scenarios, nine progressively dissimilar training subsets were derived from the full training set (cf. “Methods section”). As shown in Figure S4 (Supporting Information), nearly all ligands in the smallest training subset (10% of the training complexes) exhibited Tanimoto similarity coefficients below 0.2 to any ligand in the full test set. This design meant that a model trained on such a small, chemically distant subset and still performing well on the full test set would demonstrate strong generalization and robust docking power.

### Docking-Power Evaluation

#### Analysis of Smina-Generated Redocked Poses

Redocking of all cocrystallized ligands into their native binding sites was performed using Smina (v.2019–10–15) under a rigid-receptor/flexible-ligand protocol,^28^ based on preprocessed protein and ligand 3D structures (cf. “Methods section”). For each protein target, the search space was defined by a series of cubic grid boxes centered on the centroid of the corresponding cocrystallized ligand, with increasing edge lengths of 10, 20, 30, and 40 Å (volumes of 1000 to 64,000 Å^3^; up to 20 poses generated per size). This hierarchical design decoupled the docking search space from ligand size and experimental orientation, thereby avoiding the bias inherent to distance-based box definitions commonly used by ML docking frameworks such as DeepDock and Interformer.^15,19^ The structural deviation between each redocked and crystallographic conformation was then quantified as the heavy-atom RMSD (Å), with poses classified as near-native (RMSD ≤ 2.0 Å) or non-native (RMSD > 2.0 Å).

A total of 1,160,559 redocked poses were generated by Smina across 19,239 PDB entries. The number of poses generated per entry was significantly negatively associated with the percentage of near-native poses (Spearman’s ρ = −0.6205, *p*-value <0.05; Figure 3A), with the relationship exhibiting a nonlinear decreasing trend. This indicates that entries for which Smina produced a larger number of poses tended to yield a smaller fraction of near-native ones, suggesting that more sampling often corresponds to more challenging docking cases, where high-quality binding geometries are harder to reproduce. This observation may be partly attributed to the use of default Smina parameters, without increasing “exhaustiveness” for pose sampling or enabling postdocking minimization, in order to ensure a fair comparison with end-to-end ML docking methods. Among the 68,565 near-native poses successfully generated across all entries, nearly 80% (*n* = 53,879) satisfied all PoseBusters validity criteria, including chemical, intramolecular, and intermolecular plausibility. A strong positive correlation was observed between the percentage of near-native poses and the percentage of poses that were both near-native and PoseBusters-valid per entry (Pearson’s *r* = 0.8882, *p*-value <0.05; Figure 3B), indicating that entries generating more near-native poses also tended to yield a higher proportion of physicochemically valid poses among them. Figure 3C shows the density of near-native and PoseBusters-valid poses (red) and near-native but PoseBusters-invalid poses (blue), both exhibiting two peaks, indicative of bimodal distributions. Based on this observation, Figure 3D presents the RMSD densities of PoseBusters-valid and PoseBusters-invalid near-native poses within the [0.0,1.0] (Å) and [1.0,2.0] (Å) intervals. In each RMSD range, the two distributions differed significantly (two-sided Mann–Whitney U test with Bonferroni correction, *p*-value <0.05). Moreover, within each range, the median RMSD of PoseBusters-valid poses was lower than that of invalid ones, suggesting that physicochemically valid poses tended to be closer to the crystallographic binding mode. These findings reinforce that, although increasing the number of poses generated by Smina may also raise the number of non-native and PoseBusters-invalid poses, within the near-native population, poses that are more tightly redocked (lower RMSD) tend to exhibit higher physicochemical validity.

**Figure 3:**
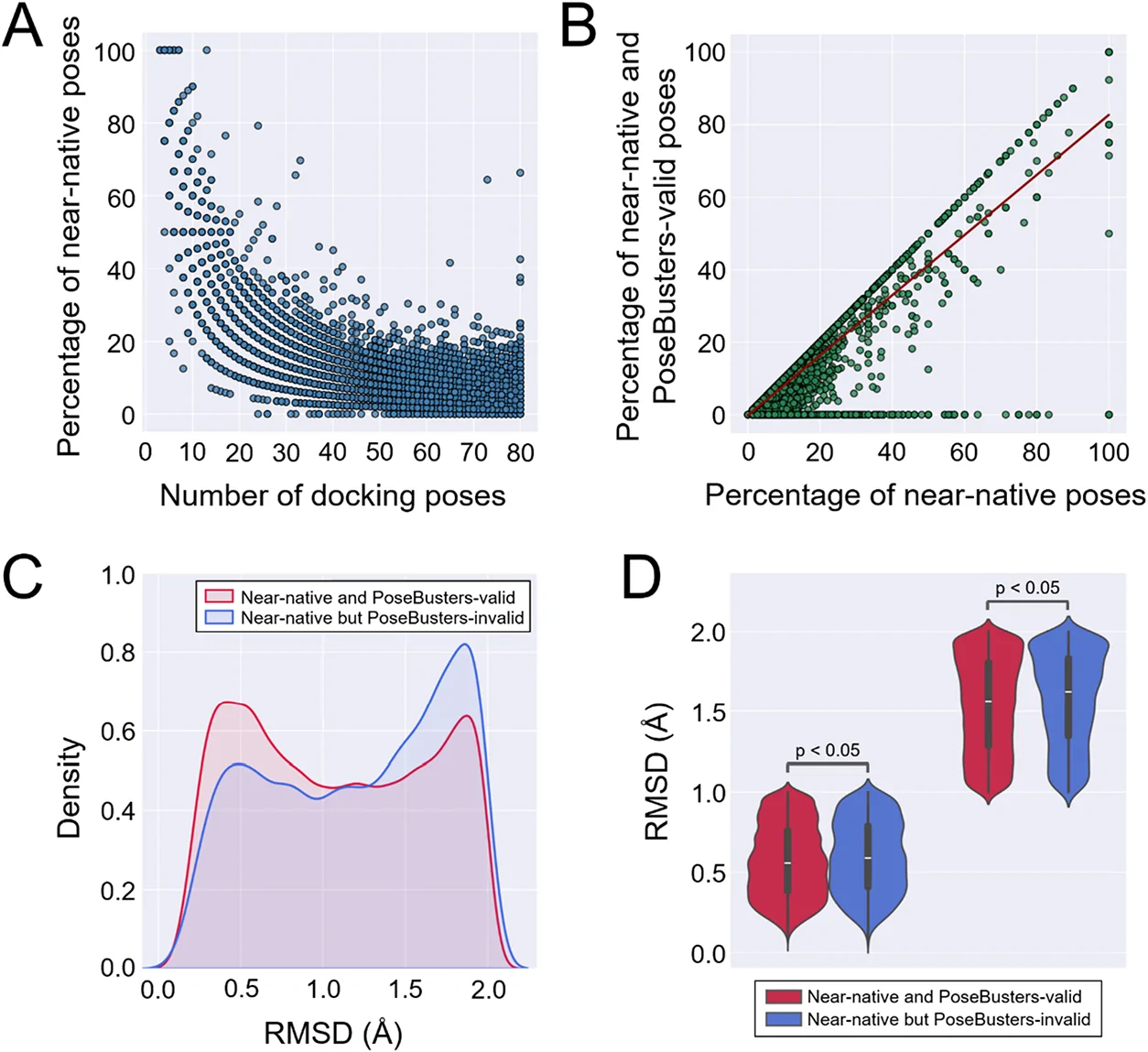
Analysis of Smina-generated redocked poses. (A) Relationship between the number of poses generated per PDB entry and the percentage of near-native poses (RMSD ≤ 2.0 Å), revealing a statistically significant negative association with a nonlinear trend. (B) Correlation between the percentage of near-native poses and the percentage of poses that were both near-native and PoseBusters-valid per PDB entry. (C) RMSD density distributions of near-native and PoseBusters-valid poses (red) and near-native but PoseBusters-invalid poses (blue). (D) RMSD density distributions of PoseBusters-valid and PoseBusters-invalid near-native poses within the [0.0, 1.0] (Å) and [1.0, 2.0] (Å) intervals.

#### Docking-Power Evaluation on the Final Benchmarking Data Set

The docking power of Smina, GNINA 1.3 (CNN-based rescoring), logistic regression rescoring on Smina-generated poses, and four ML docking tools was evaluated according to near-nativeness (RMSD ≤ 2.0 Å) and PoseBusters physicochemical validity criteria on our full test set of 5201 PDB entries (Table 1, and Figure 4). For Interformer, docking power was assessed using both Pose energy and Pose score metrics. Importantly, GNINA 1.3 rescoring and our 10 logistic regression models were applied exclusively to target-bound ligand conformations generated through physics-based docking, without any AI-driven pose generation. All poses were evaluated in the absence of nondefault and downstream correction. In line with the CASF-2016 definition of docking power,^26^ only one top-ranked pose per test-set entry (according to each method’s internal scoring) was retained for evaluation, preventing artificial inflation of performance due to inclusion of multiple top poses.

**Figure 4:**
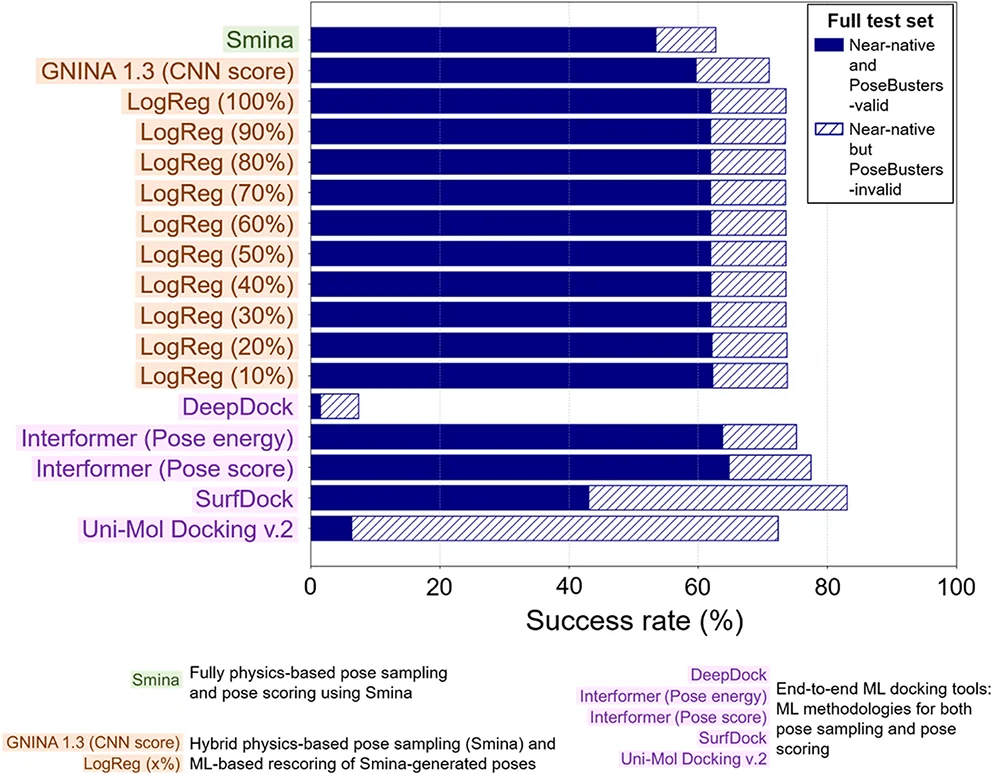
Docking power of Smina, GNINA 1.3 rescoring, and logistic regression rescoring on Smina-generated poses, alongside four ML docking frameworks, shown as stacked bar plots of near-native success rates (%) and PoseBusters physicochemical validity. The visualization facilitates comparison across methods on the full test set. The 10 logistic regression models are denoted as “LogReg (*x*%),” where *x*% indicates the proportion of the training data used.

**Table 1:** Docking Power of Smina, GNINA 1.3 Rescoring, and Logistic Regression Rescoring on Smina-Generated Poses, Alongside Four ML Docking Frameworks on the Full Test Set^a^

Docking framework/model^b^	Median RMSD of top-ranked poses across entries (Å)^c^	Percentage of entries with a near-native pose ranked first^c^	Percentage of entries with a near-native and PoseBusters-valid pose ranked first^c^
Smina	1.1922	62.74	53.47
GNINA 1.3	1.0441	71.01	59.68
LogReg (100%)	0.9935	73.60	61.93
LogReg (90%)	0.9942	73.52	61.91
LogReg (80%)	0.9942	73.52	61.91
LogReg (70%)	0.9942	73.54	61.91
LogReg (60%)	0.9935	73.60	61.93
LogReg (50%)	0.9913	73.60	61.93
LogReg (40%)	0.9913	73.60	61.95
LogReg (30%)	0.9913	73.60	61.95
LogReg (20%)	0.9852	73.76	62.14
LogReg (10%)	0.9831	73.81	62.24
DeepDock	5.5899	7.42	1.50
Interformer (Pose energy)	0.9951	75.24	63.78
Interformer (Pose score)	0.9635	77.49	64.78
SurfDock	0.8944	83.08	43.07
Uni-Mol Docking v.2	1.3171	72.39	6.33

a Numerical values are reported for precise reference.

b LogReg (*x*%): Logistic regression model trained on the x% training (sub)­set.

c Only one top-ranked pose per test-set entry (according to each method’s internal scoring) was retained for evaluation.

When physicochemical plausibility was taken into account, three of the four current ML docking tools did not produce valid poses for more than half of the test set. Among these, only Interformer achieved performance comparable to our logistic regression-based rescoring models, regardless of whether Pose energy or Pose score was used for pose selection. However, when considering only top-1 poses per test-set entry that were both near-native and PoseBusters-valid, all 10 of our logistic regression models recovered significantly more key noncovalent interactions (hydrogen bonds, ionic contacts, π-π stacking), analyzed using the ProLIF Python library (v.2.0.3),^29^ than Interformer, while no significant differences were observed among these models (Figure 5, two-sided Mann–Whitney U tests with Bonferroni correction). For each such pose generated by Smina and selected by our logistic regression models, an average of 80% of key interactions were reproduced relative to the corresponding crystal ligand, compared with 72–74% for Interformer-generated poses. Moreover, nearly 60% of top-1 poses prioritized by each logistic regression model achieved a 100% interaction recovery rate relative to the ground-truth pose, versus only 45–47% for Interformer. These results indicate that our simple rescoring models more reliably prioritize poses that are not only geometrically accurate and physicochemically plausible, but also faithfully reproduce the key protein–ligand interactions observed in experimental structures.

**Figure 5:**
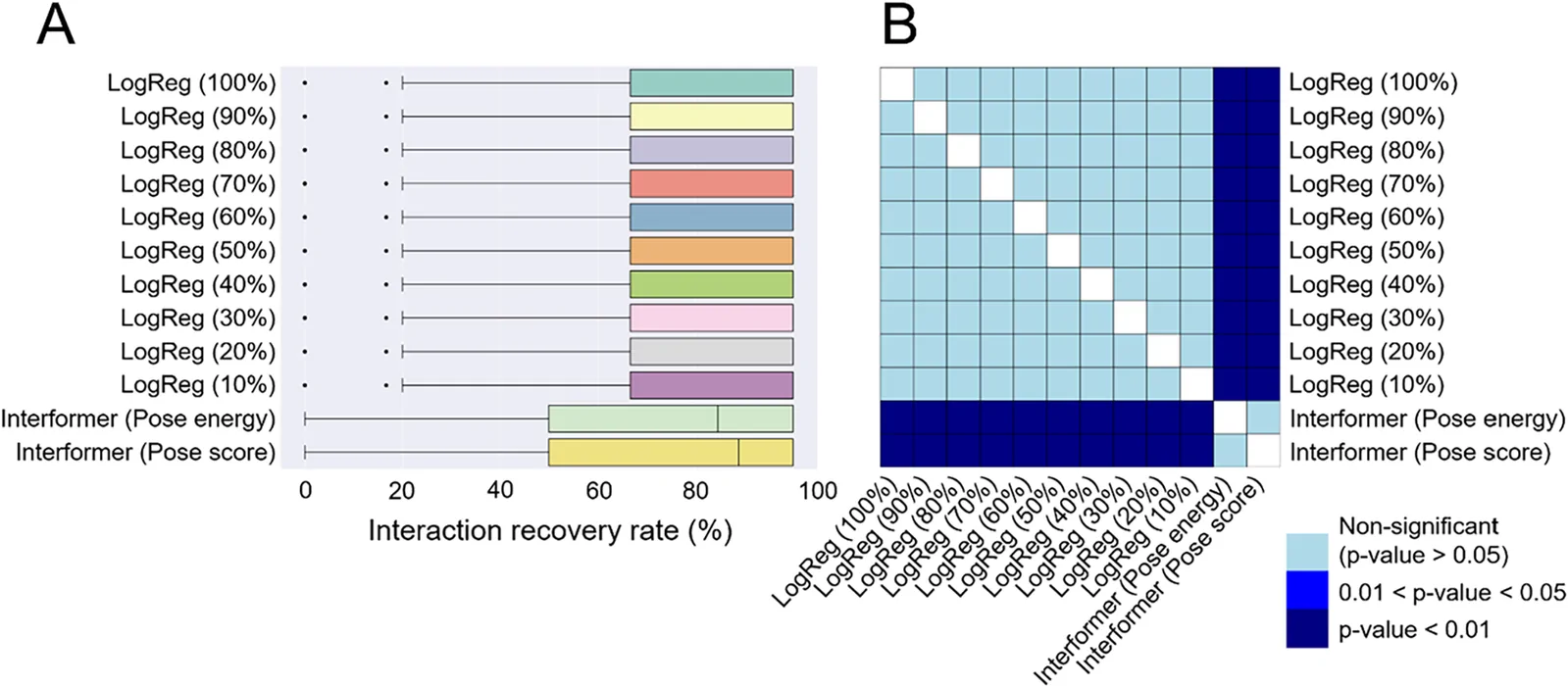
Ability of our 10 logistic regression models and Interformer to recover key noncovalent interactions among top-1 poses per test-set entry that were both near-native and PoseBusters-valid. (A) Boxplots showing the distribution of interaction recovery rates across top-1 near-native and PoseBusters-valid poses. (B) Heatmap showing pairwise p-values obtained from two-sided Mann–Whitney U tests with Bonferroni correction, comparing interaction recovery rates between models.

Additionally, DeepDock exhibited particularly poor results even under the RMSD-only criterion, while SurfDock and Uni-Mol Docking v.2 displayed a clear dependency on subsequent energy minimization: without this refinement step, SurfDock often generated poses suffering from severe steric clashes with protein atoms, and Uni-Mol Docking v.2 frequently produced distorted ligand geometries with incorrect bond lengths and angles (Figure S5, Supporting Information). Similar observations were made when the aforementioned docking approaches were evaluated on their respective cold test subsets, generated from the full test set by excluding PDB entries containing ligands with a Tanimoto similarity ≥ 0.99 (Morgan fingerprints, 2048 bits, radius 2) to any ligand included in that tool’s training data (Figures S6 and S7, Supporting Information). The reliance of SurfDock on downstream correction was, in fact, explicitly demonstrated in its original publication (cf. Figure 2 of the SurfDock paper),^20^ where the tool alone performed worse than several classical docking programs on the PoseBusters and Astex benchmarks. Similarly, Uni-Mol Docking v.2′s dependence on a “post-processing” refinement functionality has even been raised in its GitHub repository (e.g., https://github.com/deepmodeling/Uni-Mol/issues/358, accessed May 2026).

Our 10 logistic regression models were trained solely using near-native and non-native labels defined by RMSD, without any information from PoseBusters physicochemical criteria. Since each model outputs a probability score from a binary classifier, these models can be regarded as classification-based pose scorers that achieved balanced accuracies exceeding 0.85 in both 5-fold cross-validation (CV) and the full test set (Table S1, Supporting Information). This demonstrates that such simple models, trained only on Smina and GNINA 1.3 scores, are capable not only of distinguishing near-native from non-native poses but also of ranking PoseBusters-valid conformations at the top with performance comparable to, or much better than, DL-based pose generation methods. Even when trained on smaller, ligand-similarity-aware subsets, our logistic regression models retained full docking power on the complete test set, both in terms of near-nativeness combined with PoseBusters validity (Table 1 and Figure 4) and interaction recovery rate (Figure 5), underscoring the robustness of this straightforward rescoring approach and the strong complementarity between physics-based sampling and lightweight ML-based pose scoring.

Moreover, we examined how robust our conclusions were to changes in the RMSD threshold defining near-native poses. For this purpose, we evaluated two additional thresholds: 1.0 Å and 0.5 Å. When a 1.0-Å threshold was applied, the same trend as with 2.0 Å was observed: among the four ML docking tools, only Interformer matched the performance of our 10 logistic regression models, yielding comparable proportions of test-set entries for which a redocked pose with RMSD ≤ 1.0 Å and satisfying all PoseBusters criteria was ranked first (Figure S8A, Supporting Information). Interestingly, with a stricter 0.5-Å threshold, our hybrid Smina docking-ML scoring workflow ranked the largest fraction of such ultranear-native poses as top-1 (nearly 25% without PoseBusters checks and still over 20% when these were enforced). In contrast, Interformer and SurfDock ranked such highly accurate and PoseBusters-valid poses first for only about 15% of test-set entries, whereas DeepDock and Uni-Mol Docking v.2 fell below 5% (Figure S8B, Supporting Information). This once again highlights the superior robustness and generalization of hybrid physics-ML docking-scoring strategies over current generative architectures.

#### More Stringent Evaluation Scenarios

In addition to the strict cold-ligand/ligand-similarity-aware training-test splits presented above, we further evaluated the docking power of our hybrid docking-scoring strategy involving logistic regression models under several more stringent scenarios: (i) ligand- and protein-similarity-aware training-test splits, (ii) RDKit-generated ligand conformations as starting structures for docking, and (iii) removal of all test-set entries associated with the GNINA 1.3 training corpus.

To construct ligand- and protein-similarity-aware training-test splits, we removed entries from the 10% training subset (already distinct from the full test set under the ligand-similarity-aware splitting scheme described above) whose proteins exhibited ≥ 30% sequence identity with any protein in the test set after structural alignment using TM-align.^30^ Defined as the ratio of identical residues to total aligned residues, sequence identity has been cited as indicative of nonhomologous protein chains at the 30% threshold.^30^ After this filtering, the resulting training subset contained only 467 PDB entries, representing a more stringent training-test configuration enforcing both cold-ligand and cold-protein conditions. Despite the reduced training set size, a logistic regression model trained on Smina and GNINA 1.3 CNN scores derived from this subset still generalized well to the full test set, achieving success rates of 73.81% and 62.28% for near-native poses without and with PoseBusters physicochemical validity checks, respectively. These results were comparable to those obtained with larger training sets (Table 1), further highlighting the robustness of our hybrid approach under strict ligand- and protein-similarity-aware evaluation.

Another stringent evaluation scenario involved using initial 3D ligand conformations generated by RDKit, rather than the cocrystallized ligand conformations, as starting points for docking. For each test-set ligand, a 3D conformation was obtained following the same protocol used in Interformer and PoseBusters (cf. “Methods section”), representing a less biased docking scenario that more closely resembles prospective applications, in which the experimental receptor-bound pose of a ligand is unknown. With these RDKit-generated conformations, we evaluated the docking power of our hybrid docking-scoring approach as well as that of Interformer, the only ML framework giving comparable performance when crystal ligand conformations were employed. Under this more challenging scenario, the docking power of our 10 logistic regression models trained on Smina and GNINA 1.3 CNN scores was slightly higher than that of Interformer (both Pose energy and Pose score; Table S2, Supporting Information). Notably, the original Interformer study recommended using cocrystal ligand conformations as docking input:^19^ as expected, when RDKit-derived conformations were used, its success rates decreased by 26.44–26.90% without PoseBusters validity checks and by 28.13–29.50% when PoseBusters criteria were applied. On the other hand, the docking power of our hybrid strategy declined by only 16.38–16.55% and 20.48–20.88% without and with PoseBusters filters, respectively. These results suggest that the ML docking strategy implemented in Interformer might be more sensitive to the choice of the initial ligand conformation, with docking performance decreasing more markedly when the recommended cocrystal starting pose is replaced by a generic conformer.

A final stringent evaluation scenario involved explicitly removing all test-set data associated with the GNINA 1.3 training corpus.^13^ Although our training-test splits ensured that no identical PDB entries were shared between the training and test partitions of our logistic regression models (while also accounting for ligand similarity and, in one configuration, protein similarity), potential bias could still arise if some test-set protein–ligand pairs, albeit not identical conformations (thus not qualifying as data leakage in this docking-power context), originated from complexes used to train the GNINA 1.3 CNN-based SF. After their removal, the resulting test set still contained 4,860 PDB entries. Even under this stricter evaluation setting, our hybrid physics-ML approach for pose sampling and scoring continued to exhibit competitive docking power relative to Interformer (both Pose energy and Pose score; Table S3, Supporting Information), also comparable to its own performance on the full test set (Table 1). This indicates that excluding GNINA 1.3 training corpus-associated data from the test set did not affect the performance of our models.

## Discussion

This work presents a rigorous evaluation of molecular docking performance based on a large-scale benchmark comprising 5,201 carefully curated PDB entries in the test set and 14,038 entries in the training set, each with Smina-generated docking poses, hereafter referred to as “NextTopDocker.” To our knowledge, this is the first study to employ such a chemically diverse and structurally representative benchmark (updated as of May 2025) to assess the docking power of four ML frameworks: DeepDock,^15^ Interformer,^19^ SurfDock,^20^ and Uni-Mol Docking v.2.^21^ This data set provides a more representative evaluation of the current structural biology landscape than older or narrower benchmarks. Although some recently introduced resources appear larger, the effective number of complexes suitable for docking-power evaluation is substantially reduced after quality filtering. For example, the PLINDER benchmark contains over 400,000 protein–ligand pairs spanning more than 100,000 PDB entries,^31^ but only 1,035 complexes remain usable after structural quality criteria are applied.^32^ Moreover, docking-power evaluation is restricted to redocked complexes, whereas cross-docked structures are typically used for other tasks and are not appropriate for this setting, as established in CASF-2016 and PoseBusters.^22,26^ These considerations further highlight the practical scale, relevance, and recency of our “NextTopDocker” benchmark.

One key distinction of our study lies in its definition of the docking search space. Many ML docking tools define it as an expansion around the bound ligand by a fixed distance,^15,19,21^ which simplifies sampling and can favor recovery of the ground-truth pose. In contrast, our workflow uses fixed grid box sizes independent of ligand size or crystallographic orientation, two of which even exceeded the cubic search space used in the PoseBusters benchmark.^22^ In both setups, the grid box was centered at the geometric centroid of the ligand heavy atoms, maintaining spatial consistency. However, our use of larger box dimensions in several configurations further reduced spatial constraints relative to PoseBusters, which its authors already described as “comparable with blind docking.”^22^ This design helped prevent docking performance from being influenced by artificially restricted search spaces.

A notable weakness of many current ML docking pose generators is their dependence on downstream physics-based correction. Such correction was deliberately excluded from our benchmark. Note that the evaluation is not intended to assess full practical pipeline performance, but rather refinement-free intrinsic pose-generation capability. Although modern diffusion- and generative-based architectures can efficiently explore large conformational spaces, they often lack explicit physical constraints during pose generation.^22^ The generated poses may, therefore, contain steric clashes and unrealistic bond geometries, as highlighted by the PoseBusters study.^22^ More broadly, current data-driven approaches are primarily optimized to reproduce patterns present in training data rather than to explicitly enforce energetic or physicochemical realism.^23,33–38^ They may capture global structural trends but overlook the energetic landscape governing molecular stability and binding feasibility.^22^ While ML docking can be viewed as a global search process followed by local relaxation through energy minimization, our results indicate that such refinement is not inherently required for strong docking performance. Indeed, Interformer achieved competitive docking power and maintained PoseBusters-valid poses without subsequent processing.^19^ This demonstrates that DL-based pose-sampling strategies can generate physically plausible poses directly, whereas methods that only attain acceptable performance after additional correction likely rely on it to compensate for limitations in their own sampling procedures.

DeepDock, an ML framework trained and evaluated using a time-based split between training and test data, warrants particular discussion. Temporal splits commonly used in data partitioning can introduce serious bias, particularly when structurally similar complexes with homologous proteins and/or related ligands are present across splits.^22,39,40^ As a result, models may exploit such similarities rather than learning genuinely generalizable patterns, leading to overestimated performance on temporally split test sets.^40^ Consequently, DeepDock’s reported performance on its original benchmark may not reliably generalize to unseen data lacking this temporal bias. Numerous independent studies have shown that DeepDock’s performance declines substantially when evaluated on alternative test sets, often falling below classical docking methods such as GOLD, Vina, and Smina.^19–22,41,42^ Our findings further support this observation, suggesting that models trained under such biased settings may generalize poorly to chemically and structurally diverse systems.

Overall, this work offers both a comprehensive benchmark for advancing future ML and hybrid docking methods and a conceptual framework that aligns with recent efforts to critically reassess the direction of the field.^22,38,43,44^ Comparative performance trends across purely physics-based, ML-based, and hybrid methodologies indicate that the primary bottleneck in molecular docking does not lie in pose generation, but rather in pose scoring and selection.^2,10^ Achieving more accurate docking predictions requires the integration of physically grounded principles. One promising avenue is the development of hybrid physics-informed neural architectures. For example, a diffusion model could be pretrained on PoseBusters-valid docking poses generated by classical sampling methods, such as the Smina-generated poses in the training set of our “NextTopDocker” benchmark, and subsequently fine-tuned and evaluated on our test set. This approach would enable the model to capture both the energetic realism and the statistical diversity inherent to biomolecular recognition.

## Conclusions

Our new “NextTopDocker” benchmark provides a challenging framework for evaluating pose generation and docking performance. It addresses a key limitation of widely used but relatively small data sets such as Astex, CASF, and PoseBusters, which do not fully capture the chemical and structural diversity of modern docking problems. Our comprehensive evaluation of four end-to-end ML docking frameworks highlights their systematic limitations:

(i) Their training-test partitions are often bias-prone, sometimes relying on time-dependent splits that may lead to inflated performance estimates;
(ii) The definition of their search spaces often relies on the cocrystal ligand’s 3D size and orientation, which artificially increases the likelihood of near-native pose generation;
(iii) The resulting poses are frequently physically or chemically implausible, as echoed by PoseBusters analyses; and
(iv) Several frameworks (SurfDock, Uni-Mol Docking v.2) rely on downstream physics-based (non-ML) correction to achieve acceptable accuracy, thereby masking shortcomings in their ML-based sampling capability.

In contrast, our simple logistic regression models, trained on Smina and GNINA 1.3 scores from chemically dissimilar ligands and applied to Smina-generated poses, achieved docking power exceeding that of most SOTA ML docking tools. Even under ligand- and protein-similarity-aware constraints, reduced training set sizes and other stringent evaluation scenarios, this hybrid approach generalized robustly across test complexes, underscoring the resilience of classical sampling combined with ML-based scoring. Altogether, our findings highlight the limitations of current ML pose generation and emphasize the importance of embedding physics-informed constraints directly within ML docking architectures to ensure physically valid poses without reliance on subsequent refinement. Furthermore, our results suggest that future progress in molecular docking will depend more critically on improving pose scoring and selection than on pose generation alone.

## Methods

### Data Retrieval and Curation

A raw data table containing information on experimental structures was first downloaded from the PDB,^45^ using the “Advanced Search” option with the following criteria: (i) the structure must be compatible with the pdb format; (ii) the polymer entity type must be “Protein” and the entry polymer type must be “Protein (only)”; (iii) the refinement resolution must be ≤ 2.5 Å; (iv) the experimental method must be “X-ray diffraction”; (v) the refinement R factors, “R-value free” and “R-value work,” must be lower than 0.30 and 0.25, respectively;^46^ and (vi) the UniProt accession code must not be empty. As of May 2025, a total of 126,961 PDB entries satisfied these selection criteria.

The data table obtained from the above search was subsequently processed as follows:

(i) PDB entries containing unknown ligands (“UNL”) or lacking cocrystallized ligands (apo-structures) were removed;
(ii) Only entries with an R-factor discrepancy between 0 and 0.05 were retained;^46^
(iii) Inorganic ligands were removed, defined as those containing at least one atom other than C, H, O, N, P, S, F, Cl, Br, or I;
(iv) PDB entry ID-ligand ID pairs were retained only if the ligand satisfied an extended set of drug-likeness criteria:

- Molecular weight (MW) between 250 and 800 Da (values taken directly from the PDB),
- Lipophilicity (WlogP) between −2.0 and +6.0 (calculated using the ‘rdkit.Chem.Crippen’ module implemented in RDKit, v.2025.03.3),
- Number of rotatable bonds lower than 15 and topological polar surface area smaller than 180 Å^2^ (calculated using ‘rdkit.Chem.rdMolDescriptors’),
- Numbers of hydrogen bond acceptors and hydrogen bond donors were each lower than 10 (calculated using ‘rdkit.Chem.Lipinski’).

These physicochemical ranges were chosen to capture bioactive, nonfragment molecules while covering a broader ligand space than conventional filters (e.g., Lipinski’s Rule of Five or Ghose’s filter). The criteria included modern drugs such as kinase inhibitors, antivirals, macrocycles, natural products, and allosteric modulators, while maintaining tractability for downstream docking with Smina;^47–49^

(v) Solvents, buffers, additives, detergents, and coenzymes were removed;
(vi) Complexes containing invalid ligands due to missing atoms were excluded;
(vii) In complexes that contained more than one ligand after Steps (i)–(vi), the ligand exhibiting a therapeutically relevant phenotype on the target was chosen as the redocked ligand.

Each remaining PDB complex was then prepared according to the following procedure:

(i) Protein file preparation:

An in-house Bash script was developed to automate the following tasks:

- From each original PDB entry, the protein structure (with associated metal ions, if present in the binding site) was extracted and saved in pdb format;
- To better mimic a realistic binding environment, metal ions were retained if located within 5 Å of any heavy atom of the cocrystallized ligand, provided they were supported by Smina’s built-in atom type definitions and/or considered biologically relevant (e.g., involved in catalysis or ligand coordination, rather than crystallization artifacts). Retained ions included: Ca, Cd, Co, Cu, Fe, K, Mg, Mn, Mo, Na, Ni, and Zn;
- Target structures were then prepared using ChimeraX’s ‘DockPrep’ tool (v.1.8). Hydrogen atoms and charges were added according to the atomic type of each hydrogen-bearing heavy atom. Noncomplex ions (those not covalently or coordinatively bound in the binding site) were deleted. Incomplete side chains were rebuilt using the Dunbrack rotamer library. Final protein structures were saved in mol2 format.

(ii) Ligand file preparation:

A PyMOL (v.2.5) batch script was created to automate the following tasks:

- Each complex structure was fetched by its PDB ID, and the cocrystallized ligand was extracted using its HET code and chain ID;
- Hydrogens were added in PyMOL, and the ligand was saved in both mol2 and sdf formats;
- Ligand sanitization was checked using the ‘SanitizeMol’ function from RDKit’s ‘Chem’ module (v.2025.03.3). If sanitization failed, the ligand’s experimental 3D conformation (sdf format) was downloaded manually from the RCSB database, hydrogens were readded in PyMOL, and the molecule was saved again in mol2 and sdf.

Each PDB entry successfully prepared and retained in our data table corresponds to a protein target cocrystallized with a drug-like ligand of therapeutic relevance for that protein. For each entry, the data table includes: (i) the PDB ID; (ii) the cocrystallized ligand’s HET code, MW (Da), IUPAC name, SMILES string, WLogP, number of rotatable bonds, numbers of hydrogen bond donors and acceptors, and topological polar surface area (Å^2^); (iii) the R-value free, R-value work, and R-factor discrepancy; (iv) the entry resolution (Å); and (v) keywords describing the protein family of the target and the phenotype of the cocrystallized ligand. This data table is provided as a csv file, along with all prepared protein and ligand structures (proteins: mol2; ligands: mol2 and sdf) on GitHub and Zenodo (cf. “Data and Software Availability”).

### Stratified Data Split

Since our approach would be evaluated on its docking power, i.e., its ability to operate on the 3D conformations of ligands generated by docking, the training-test data split (at the level of PDB entries) followed a strict cold-ligand principle. Specifically, no cocrystallized ligand HET code was shared between the training and test sets. However, because distinct HET codes do not necessarily preclude high structural similarity (e.g., enantiomers with identical 2D fingerprints), we further enforced a 2D cold-ligand constraint. In the training set, only PDB entries whose ligands had a maximum Tanimoto similarity (Morgan fingerprints, 2,048 bits, radius 2) ≤ 0.99 to any ligand in the test set were retained.^6,50^ This additional filtering ensured that no training-test bias or ligand-level data leakage occurred. To further challenge the models, we adopted a similarity-aware design by partitioning the training set into nine subsets with sizes ranging from 90% to 10% of the full training set, in 10% decrements. Subsets of decreasing size were created by progressively excluding PDB entries whose cocrystallized ligands exhibited Tanimoto similarity (Morgan fingerprints, 2,048 bits, radius 2) above successively lower thresholds relative to the test-set ligands (Table 2). Thus, the smaller the subset, the more structurally dissimilar its ligands were to those in the test set. This design allowed us to evaluate model performance not only as a function of training set size but also under increasing distributional shift, mimicking realistic scenarios in which ligands for novel targets differ substantially from those in the training data. Note that, since multiple training-set ligands could share identical maximum similarity scores to test-set ligands, exact 10% splits could have required random selection among tied training-set PDB entries. To avoid introducing such randomness, we instead allowed subset sizes to deviate slightly from the target percentages (e.g., 9.63%, 20.03%, 30.08%), which were subsequently rounded to the nearest 1% for reporting purposes. Additionally, tool-specific cold test subsets were constructed by removing from the full test set all entries whose ligands exhibited a Tanimoto similarity (Morgan fingerprints, 2,048 bits, radius 2) ≥ 0.99 to any ligand included in the training data of each ML docking tool. This procedure yielded four test subsets: DeepDock-cold, Interformer-cold, SurfDock-cold, and Uni-Mol Docking v.2-cold, facilitating unbiased evaluation by preventing any data overlap between training and testing (Table 2).

**Table 2:** Composition of the Training Set, Its Nine Similarity-Aware Subsets, the Test Set and Its Four Tool-Specific Cold Subsets

data partition	number of unique PDB entries	number of cocrystallized ligands (unique HET codes)	highest similarity between each training-(sub)set ligand and all test-set ligands^a^
Training Set			
100%	14,038	10,128	0.9839
90% (89.96%)	12,628	9517	0.6620
80% (80.08%)	11,241	8794	0.5349
70% (70.00%)	9826	7828	0.4382
60% (60.03%)	8427	6757	0.3793
50% (50.00%)	7019	5592	0.3431
40% (40.10%)	5629	4443	0.3175
30% (30.08%)	4222	3266	0.2963
20% (20.03%)	2812	2051	0.2747
10% (9.63%)	1352	822	0.2481
Test Set			
100%	5201	3622	Not applicable
DeepDock-cold	4284	3451	
Interformer-cold	4175	3420	
SurfDock-cold	4248	3442	
Uni-Mol Docking v.2-cold	3899	3366	

a Tanimoto similarity according to Morgan fingerprints (2,048 bits, radius 2).

### Molecular Docking with Smina and Rescoring Using GNINA 1.3

We performed redocking of all cocrystallized ligands into their respective binding sites using a rigid-receptor/flexible-ligand protocol with Smina (v.2019–10–15).^28^ Docking was carried out on the 3D mol2 structures of proteins and ligands previously prepared (cf. “Data retrieval and curation section”). For each protein target, the binding pocket was defined by a grid box centered at the centroid of the corresponding cocrystal ligand (‘autobox_ligand’ option). The ‘exhaustiveness’ parameter was set at 8 (default value), and a fixed number of seeds was appointed. To increase the diversity of generated poses for subsequent analyses, we tested four grid box volumes: 1,000 Å^3^ (10 Å × 10 Å × 10 Å), 8,000 Å^3^ (20 Å × 20 Å × 20 Å), 27,000 Å^3^ (30 Å × 30 Å × 30 Å), and 64,000 Å^3^ (40 Å × 40 Å × 40 Å). For each grid box size, up to 20 poses were retained in both mol2 and sdf formats, resulting in a theoretical maximum of 80 poses per ligand. The ‘minimize’ option was not used during docking to avoid additional minimization of poses. The RMSD (Å) between each redocked conformation and the crystallographic pose (heavy atoms only) was computed using Open Babel’s ‘obrms’ tool (v.3.1.1).^51^ Redocked conformations with RMSD ≤ 2.0 Å from the experimental pose were classified as near-native, and as non-native otherwise.

Since the number of non-native redocked poses largely exceeded that of near-native ones, we alleviated this imbalance in the training set by reducing redundancy among non-native poses. Specifically, two non-native poses (from the same PDB entry) were considered redundant if their pairwise RMSD did not exceed 0.5 Å. In such cases, the pose with the higher RMSD relative to the experimental conformation was discarded. This filtering step was applied exclusively to the training set. The test set, on the other hand, was left unmodified, as all generated poses must be retained to enable a fair comparison between our docking workflow and the evaluated ML frameworks. All remaining redocked poses (mol2 format) in the training set, as well as all poses in the test set, were rescored using GNINA 1.3 with the ‘score_only’ option.^13^ The protein conformations were identical to those used in previous Smina docking runs, while the ligand poses were retained exactly as generated by Smina. Default parameters were used for CNN-based scoring, and a “CNNScore” was recorded for each redocked pose. All docking poses (mol2, sdf), along with the corresponding Smina docking scores and GNINA 1.3 CNN-based scores are publicly available (cf. “Data and Software Availability”).

### Evaluation of Docking Poses Using PoseBusters

We assessed the physical and chemical validity of redocked poses using the PoseBusters test suite, following the authors’ recommendations.^22^ Three input files were prepared for each complex: (i) an sdf containing the redocked ligand pose, (ii) an sdf of the corresponding crystal ligand (prechecked for sanitization as described above), and (iii) a pdb file of the protein with only the cocrystallized ligand removed. A redocked pose was considered PoseBusters-valid if its RMSD to the crystal ligand did not exceed 2.0 Å and it passed all 26 PoseBusters criteria, grouped into three categories: (i) chemical validity and consistency (file loading, sanitization, InChI conversion, atom connectivity, molecular formula, molecular bonds, double bond stereochemistry, and tetrahedral chirality); (ii) intramolecular validity (bond lengths and angles, internal steric clashes, aromatic ring planarity, nonaromatic ring nonplanarity, double bond planarity, and energy ratio); and (iii) intermolecular validity (overlapping volume and minimum distance between ligand atoms and protein atoms, organic or inorganic cofactors and water molecules, as well as the maximum protein–ligand atom distance). Note that we computed RMSD values using the ‘obrms’ function in Open Babel (v.3.1.1),^51^ ensuring consistency across all docking tools in this study.

### Construction of Logistic Regression Models

We used the ‘LogisticRegression’ function from scikit-learn (v.1.6.1) with default hyperparameters (including L2 regularization with *C* = 1.0) to train 10 logistic regression models, each corresponding to the entire training set or one of its nine similarity-aware subsets. These models were trained to discriminate between near-native and non-native redocked poses, as defined by their RMSD. Using default hyperparameters is standard practice for logistic regression when interpretability and reproducibility are prioritized, especially since this model type has few sensitive hyperparameters and is not prone to overfitting in its regularized default form.^52^

For each model, 5-fold CV was conducted on the corresponding training data. Each fold was defined using a cold-ligand split (based on the HET codes of the cocrystallized ligands) to prevent information leakage. For every redocked pose, its Smina docking score and GNINA 1.3 CNN-based score were normalized using the ‘StandardScaler’ function (default parameters) from scikit-learn (v.1.6.1): the scaler was fit on each training fold and applied to the corresponding validation fold. Since logistic regression is a binary classifier, the decision threshold was optimized to balance performance between classes. Out-of-fold predictions (i.e., the predicted probabilities of each pose being near-native) were collected across the five CV folds, and the optimal threshold was defined as the probability cutoff that maximized the mean balanced accuracy across folds. This optimization was performed using Optuna (v.4.4.0) with a search space ranging from 0.01 to 0.99, aiming to maximize the mean balanced accuracy. The Tree-structured Parzen Estimator (TPE) sampler was used with a fixed random seed to allow reproducibility. The number of optimization trials was set to 100, as recommended in the Optuna documentation.^53^

After CV, each logistic regression model was retrained on its entire training data and evaluated on the test set to assess generalizability. The ‘StandardScaler’ was fit on the corresponding complete training data (either the full training set or one of its subsets) and subsequently applied to the test set. The decision threshold determined during CV was then used to compute the confusion matrix and balanced accuracy. For each PDB entry, the Smina redocked pose with the highest predicted near-native probability was retained for docking-power evaluation.

### Molecular Docking with End-to-End ML Docking Frameworks

#### DeepDock

Input files for DeepDock consisted of the protein structures (in pdb format) and their respective cocrystallized ligands (in mol2), both prepared following the same protocol used for the PoseBusters benchmark.^22^ Using these two files, a polygon mesh representing the molecular surface of the binding site was generated with MSMS and PyMesh,^54^ employing the default parameters provided in the DeepDock GitHub repository (https://github.com/OptiMaL-PSE-Lab/DeepDock, accessed August 2025). As described by the authors of DeepDock, the binding site was defined as all protein atoms within 10 Å of any ligand atom. The resulting mesh, saved in ply format, together with the crystal ligand, was used for redocking with the pretrained DeepDock model and its corresponding checkpoint. For each PDB entry, DeepDock generated a single optimized conformation as an RDKit Mol object, which was subsequently saved in sdf using the ‘SDWriter’ function from the ‘rdkit.Chem’ module. In cases where the optimization process exceeded the maximum number of iterations but still produced a conformation that could be saved as an sdf, the pose was retained for evaluation of near-nativeness and physicochemical plausibility. For conformations that were successfully generated by DeepDock but could not be written by the ‘SDWriter’ function integrated into the DeepDock code (as provided by its authors), provided that the corresponding crystal ligand was successfully loaded and sanitized with RDKit, we considered them as dockable cases that subsequently did not pass any criteria. The RMSD between generated and crystal poses was computed using Open Babel (v.3.1.1), and all resulting redocked poses were further evaluated with the PoseBusters test suite to assess their physicochemical validity.

#### Interformer

Input for Interformer consisted of the original protein–ligand complexes downloaded from the Protein Data Bank (May 2025, in pdb format) and the corresponding cocrystallized ligands in sdf.^19^ All input structures were prepared following the Interformer authors’ guidelines to ensure its optimal performance. For the complexes, only heteroatoms annotated as “UNX” were removed. Ligand structures in sdf were the same as those obtained from our previously described preparation procedure (cf. “Data retrieval and curation”). In cases where ligands could not be processed by Interformer, the crystallographic conformations were retrieved directly from the RCSB database and hydrogens were added using PyMOL (v.2.5). All 5,201 cocrystallized test-set ligands were successfully loaded with RDKit and passed its sanitization.

As recommended in the Interformer literature, the cocrystallized conformation of each ligand was used as the starting structure, rather than a geometry relaxed by the universal force field (UFF). For each entry, 20 conformations were generated following the energy functions and checkpoints provided in the Interformer GitHub repository (https://github.com/tencent-ailab/Interformer, accessed August 2025). These conformations were used without modification as input to the pose-scoring model. To evaluate docking performance, for each entry, we selected the single conformation with either the lowest pose energy or the highest pose score. RMSD values between the selected conformations and their corresponding experimental crystal structures were computed with Open Babel (v.3.1.1). Finally, to assess the fraction of successful cases that also satisfied structural validity criteria, generated protein (in pdb, without waters or cocrystallized ligands) and ligand files (in sdf) were subjected to PoseBusters analysis.

#### SurfDock

For protein structures, SurfDock employed a trilevel representation comprising the sequence level, the residue graph level, and the protein surface level. The sequence and residue graph embeddings were obtained using the large language model ESM-2,^55^ while the surface representation was prepared following a protocol similar to that used in DeepDock and MaSIF.^56^ As described by the authors of SurfDock, the binding site was defined as all protein atoms within 8 Å of any ligand atom. Before surface triangulation with MSMS and processing with PyMesh, each protein structure was prepared with Open Babel (v.3.1.1), all receptor hydrogens were corrected using Reduce,^57^ and the resulting structure was saved in pdb format. For ligand structures, the cocrystallized conformations (in sdf) were prepared in the same manner as those used for the PoseBusters test suite. For each PDB entry in the test set, SurfDock generated 40 candidate conformations using the confidence score functions and checkpoints provided in the SurfDock GitHub repository (https://github.com/CAODH/SurfDock, accessed August 2025). No postdocking energy minimization was performed: the “force_optimize” argument was disabled, while all other parameters were kept at their default values. For docking-power evaluation, the top-ranked conformation based on the highest confidence score was selected per test-set entry (in sdf). The RMSD between the selected pose and the corresponding crystal ligand was computed using Open Babel (v.3.1.1), after which the pose was further analyzed with the PoseBusters test suite to assess its physicochemical validity.

#### Uni-Mol Docking v.2

Input files for Uni-Mol Docking v.2 consisted of the template protein structures (in pdb format) and their corresponding cocrystallized ligands (in sdf). The preparation of the initial protein and ligand files followed the protocol described in the PoseBusters paper, which was also used for generating the PoseBusters benchmark.^22^ Specifically, from each test-set complex, only the cocrystallized ligand of interest (the one selected for redocking) was removed. This resulted in a protein structure that was identical to the original crystal complex except for the absence of that single ligand, while all other structural components were retained. Ligands were extracted from their test-set complexes using PyMOL (v.2.5) and saved in sdf. All 5,201 were successfully loaded with RDKit and passed its sanitization prior to docking.

The docking grid box was defined based on the coordinates of the crystallographic ligand. The grid center corresponded to the centroid of all heavy atoms of the ligand, and the box dimensions were computed as the orthogonal extent of the ligand plus an additional 10-Å margin along each Cartesian axis. Consequently, the final grid box size depended on both the size and 3D conformation of the crystallographic ligand. Grid box information was stored in a json file. No postgeneration pose refinement (e.g., steric clash correction) was applied. Specifically, the ‘steric-clash-fix’ argument was disabled, and all other parameters were retained at their default settings, using the publicly available implementation of Uni-Mol Docking v.2 (https://github.com/deepmodeling/Uni-Mol/tree/main/unimol_docking_v2, accessed October 2025). For each test-set complex, a single docking pose was generated (in sdf). The RMSD between the predicted and crystallographic poses was computed using Open Babel (v.3.1.1). Finally, the docked ligand structures were evaluated with PoseBusters, alongside their corresponding protein templates, to assess the chemical and physical plausibility of the generated docking poses.

### More Stringent Evaluation Scenarios

#### Sequence Identity

Structural alignment was performed using TM-align (https://aideepmed.com/TM-align, v.20220412, accessed December 2025).^30^ Sequence identity after alignment was obtained from the ‘Seq_ID’ field output by TM-align and computed as the ratio of identical residues to the total number of aligned residues.

#### RDKit-Generated initial 3D Ligand Conformations

The ETKDG v.3 algorithm implemented in the ‘rdkit.Chem.rdDistGeom’ module (RDKit, v.2025.03.3) was used to generate 30 ligand conformations per test-set ligand. These conformations were subsequently energy-minimized using the UFF (‘maxIters’ parameter set at 10,000), and the lowest-energy conformation was selected as the initial structure for docking.

## Supplementary Material



## Data Availability

All source code and input/output data are available free of charge at our GitHub repository https://github.com/caominhtr/NextTopDocker and have also been archived on Zenodo (https://zenodo.org/records/17492994) for long-term accessibility.

## Notes

Additional data analyses, docking- and classification-power results, and PDB ID lists for training sets of four ML docking tools (PDF)

## Abbreviations Used

AI: artificial intelligence
CNN: convolutional neural network
CV: cross-validation
DL: deep learning
ID: identifier
LogReg: logistic regression
ML: machine learning
PDB: Protein Data Bank
RMSD: root-mean-square deviation
SB: structure-based
SF: scoring function
SOTA: state-of-the-art
TPE: Tree-structured Parzen Estimator
UFF: universal force field
VS: virtual screening
