# Supplementary material for: NextTopDocker: A Large-Scale Docking-Power Benchmark Reveals Limitations of Current End-to-End Machine-Learning Docking and the Strength of Hybrid Rescoring
Source: J Med Chem. 2026 Aug 6;69(16):19808–21. doi: 10.1021/acs.jmedchem.6c01143 (PMC13699998; doi:10.1021/acs.jmedchem.6c01143)
Supplement: Supplementary file 1 [file jm6c01143_si_001.pdf]

Supporting Information

NextTopDocker: a large-scale docking-power benchmark reveals limitations of current end-to-end machine-learning docking and the strength of hybrid rescoring

Cao-Minh Truong,[1] Pedro J. Ballester,[2] Olivier Taboureau,[1]* Viet-Khoa Tran-Nguyen[1]*

[1] Université Paris Cité, CNRS UMR 8251, INSERM ERL 1133, F-75013 Paris, France

[2] Department of Bioengineering, Imperial College London, London SW7 2AZ, UK

*Corresponding authors: Olivier Taboureau (olivier.taboureau@u-paris.fr) and Viet-Khoa Tran-Nguyen (viet-khoa.tran-nguyen@u-paris.fr).

Contents of Supporting Information:

Fig. S1. Structural diversity of the 13,750 unique co-crystal ligands included in the final data set........... **S3**

Fig. S2. Distribution of 11 ligand-based physicochemical properties across 13,750 unique ligands in our final data set... **S4**

Fig. S3. Most represented protein families and most frequent protein targets in our final data set. **S5**

Fig. S4. Cumulative percentage of Tanimoto similarity coefficients (Morgan fingerprints, 2,048 bits, radius 2) between ligands in each training subset and those in the full test set... **S6**

Fig. S5. Heatmap showing the proportion of top-1 poses that were near-native but PoseBusters-invalid across 5,201 PDB entries of the full test set. **S7**

Fig. S6. Docking power evaluated on each ML docking tool's cold test subset. **S8**

Fig. S7. Heatmaps showing the proportion of top-1 poses that were near-native but PoseBusters-invalid across each ML docking tool's cold test subset. **S9**

Fig. S8. Percentage of test-set entries for which a redocked pose with RMSD ≤ 1.0 Å or 0.5 Å relative to its corresponding crystallographic pose was ranked first. **S10**

Table S1. Classification power of 10 logistic regression models. **S11**

Table S2. Docking power evaluated on the full test set using RDKit-generated ligand conformations as docking input. **S12**

Table S3. Docking power evaluated on the test set after excluding complexes present in the GNINA 1.3 training data set. **S13**

List S1. List of PDB IDs included in the training data of DeepDock. **S14**

List S2. List of PDB IDs included in the training data of Interformer. **S35**

List S3. List of PDB IDs included in the training data of SurfDock. **S60**

List S4. List of PDB IDs included in the training data of Uni-Mol Docking v.2. .... **S82**

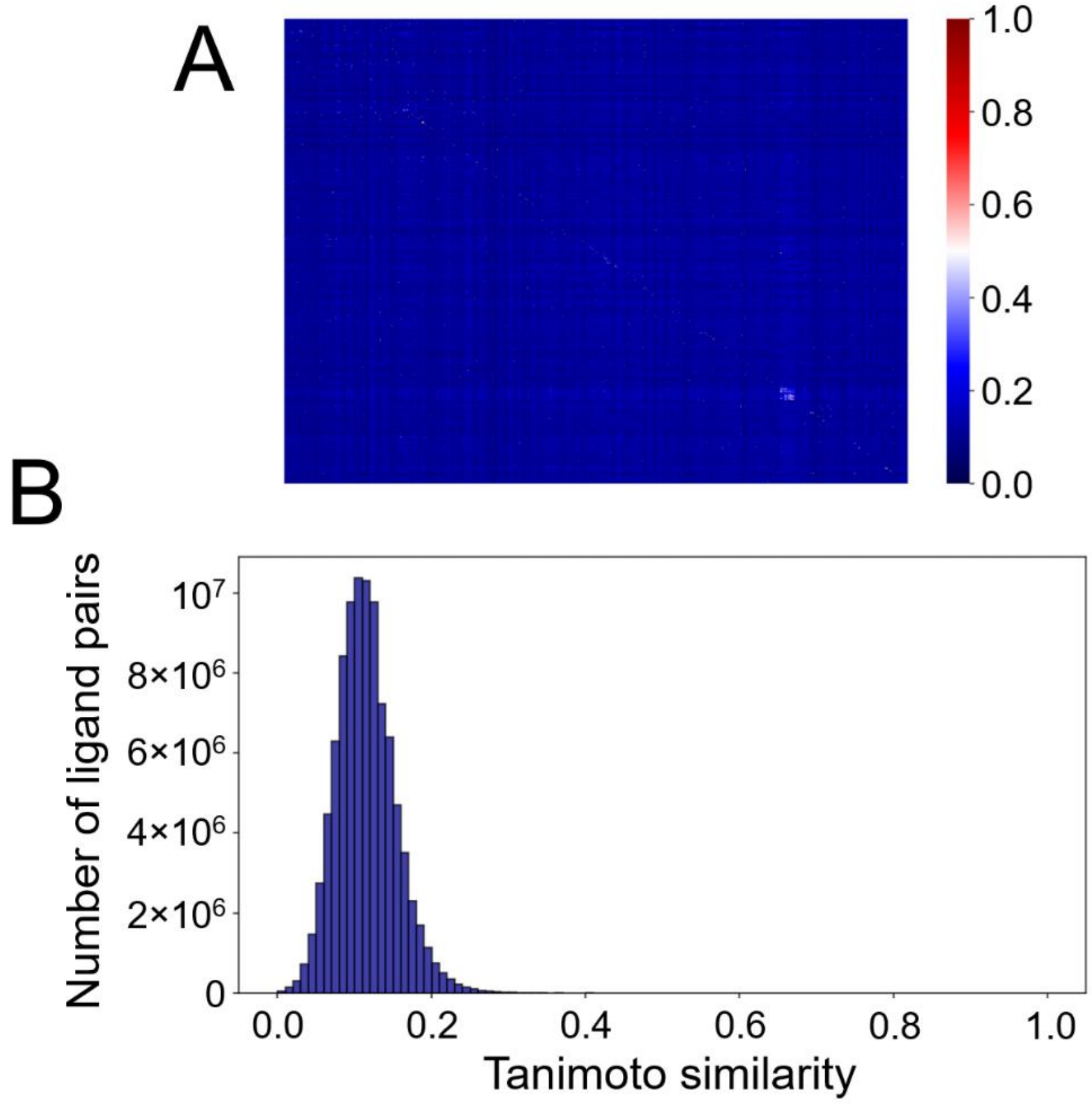

Fig. S1. Structural diversity of the 13,750 unique co-crystal ligands included in the final data set. (A) Heatmap illustrating pairwise Tanimoto similarity coefficients based on Morgan fingerprints (2,048 bits, radius 2). (B) Histogram showing the distribution of pairwise similarity coefficients, centered at 0.1105, highlighting the high structural dissimilarity among ligands retrieved from 19,239 PDB entries.

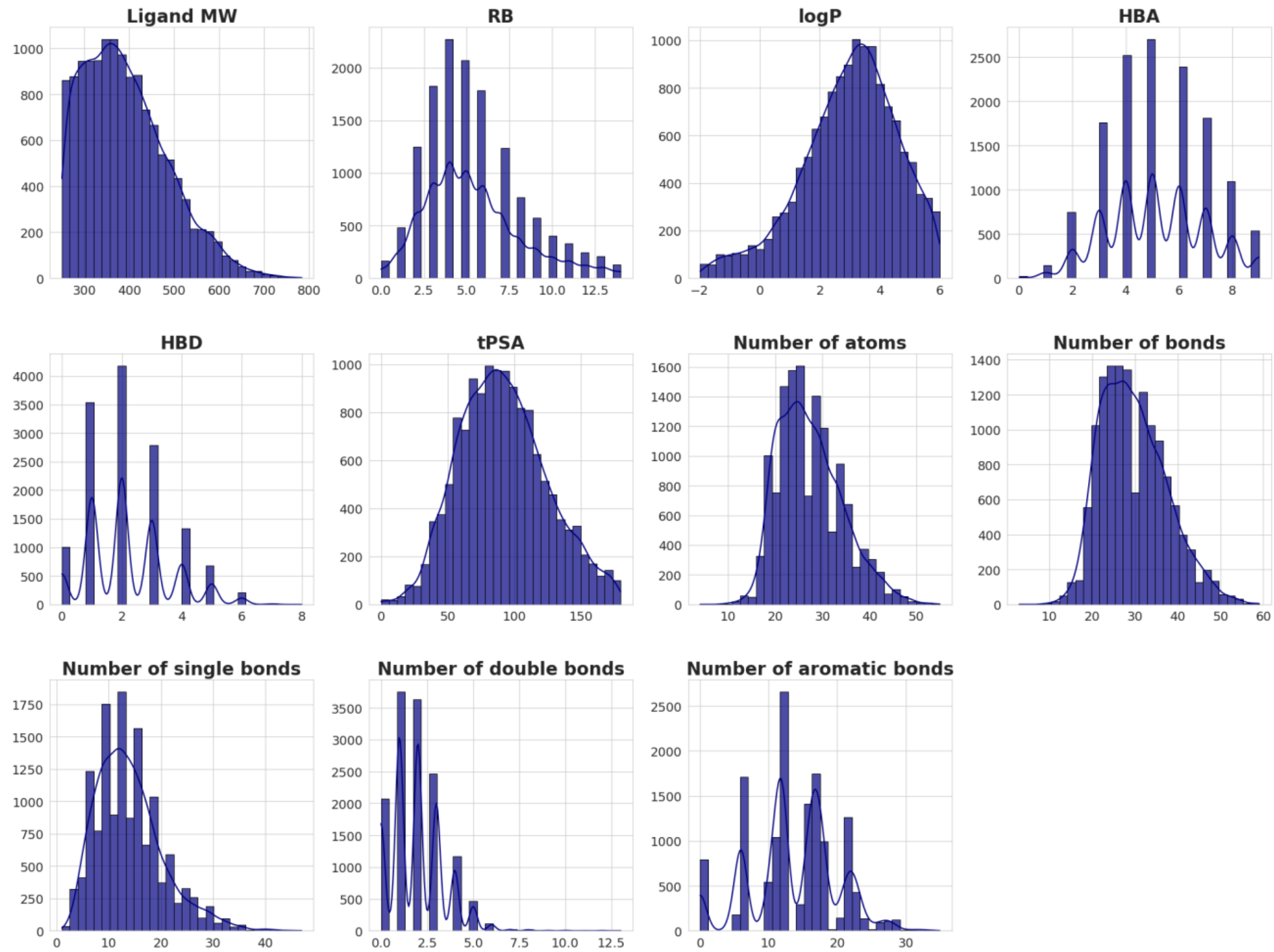

Fig. S2. Distribution of 11 ligand-based physicochemical properties across 13,750 unique ligands in our final data set: molecular weight (MW, in Da), lipophilicity (logP), hydrogen bond acceptor count (HBA), hydrogen bond donor count (HBD), topological polar surface area (tPSA, in Å^2), number of atoms, number of bonds, number of single bonds, number of double bonds, number of aromatic bonds, and number of rotatable bonds (RB). MW values were taken directly from the PDB, whereas the other properties were computed using RDKit (v.2025.03.3). All properties spanned a wide range of values, reflecting high chemical diversity among the ligands.

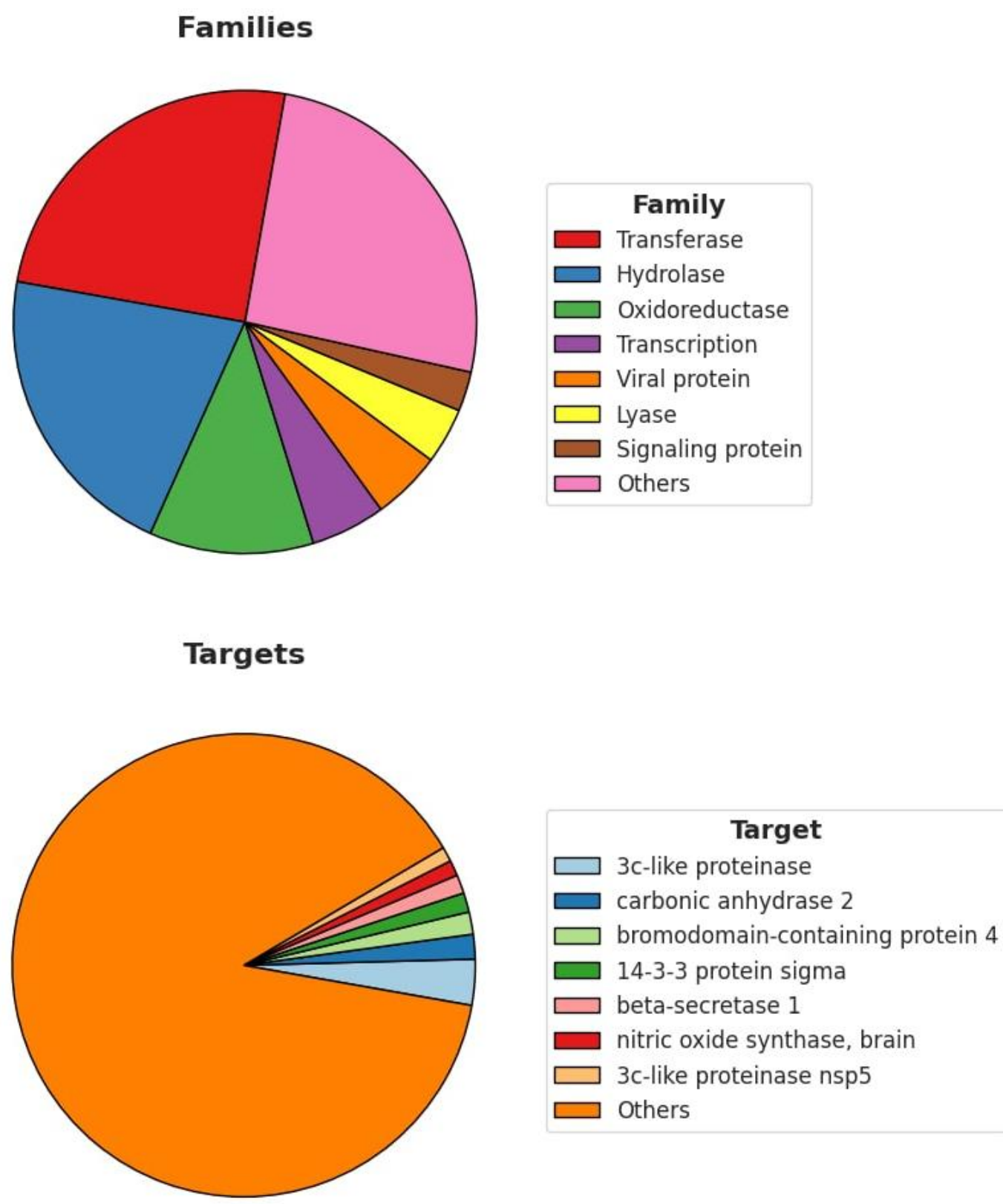

Fig. S3. Most represented protein families and most frequent protein targets in our final data set. Major families commonly studied in biochemistry constitute a substantial proportion of the retrieved PDB entries.

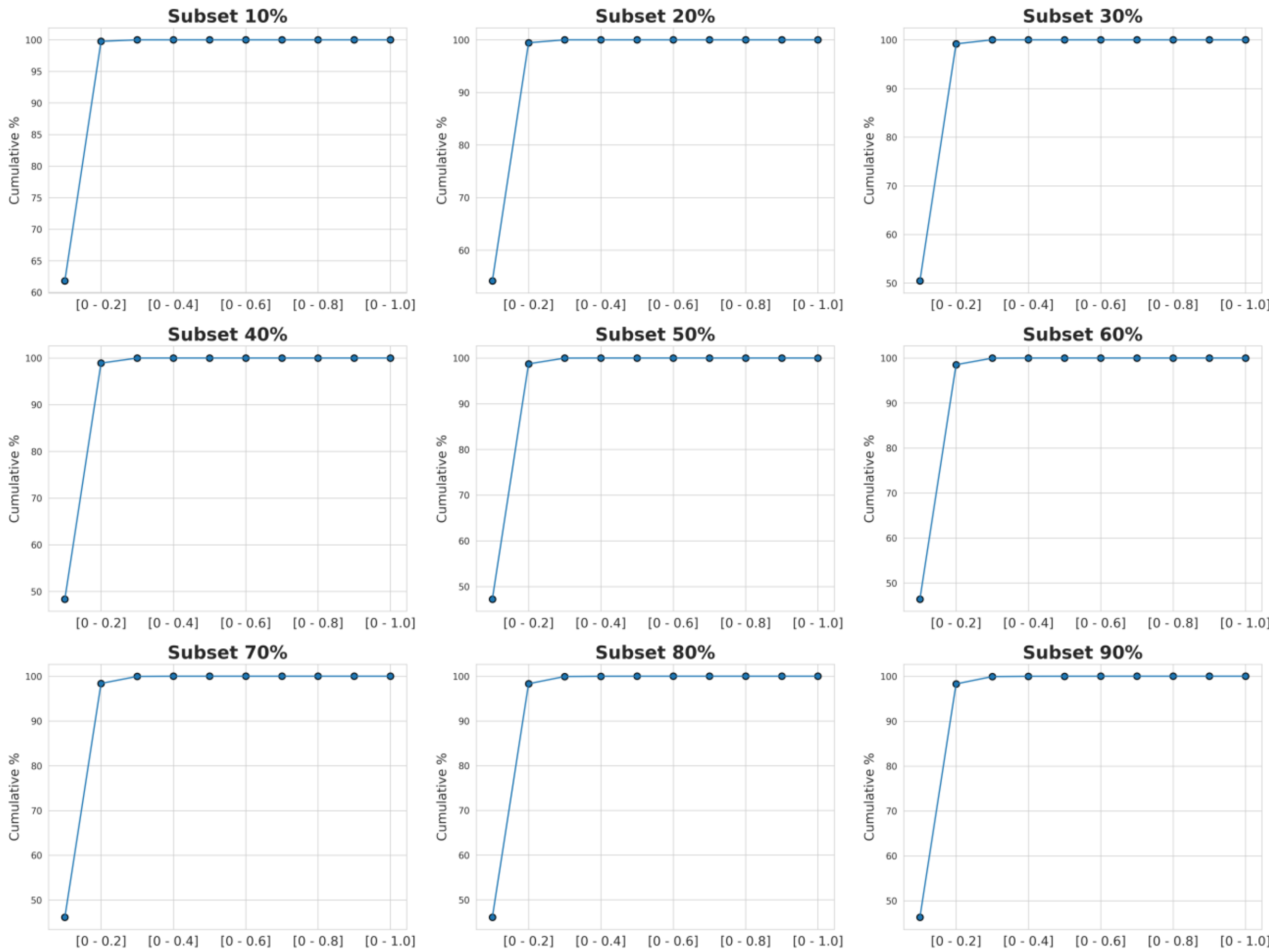

Fig. S4. Cumulative percentage of Tanimoto similarity coefficients (Morgan fingerprints, 2,048 bits, radius 2) between ligands in each training subset and those in the full test set. The nine training subsets were created by progressively removing PDB entries with ligands more similar to those in the test set, reducing each subset by 10% of the full training set. Smaller subsets therefore contained ligands increasingly dissimilar to test-set ligands, presenting a greater challenge for predictive model training.

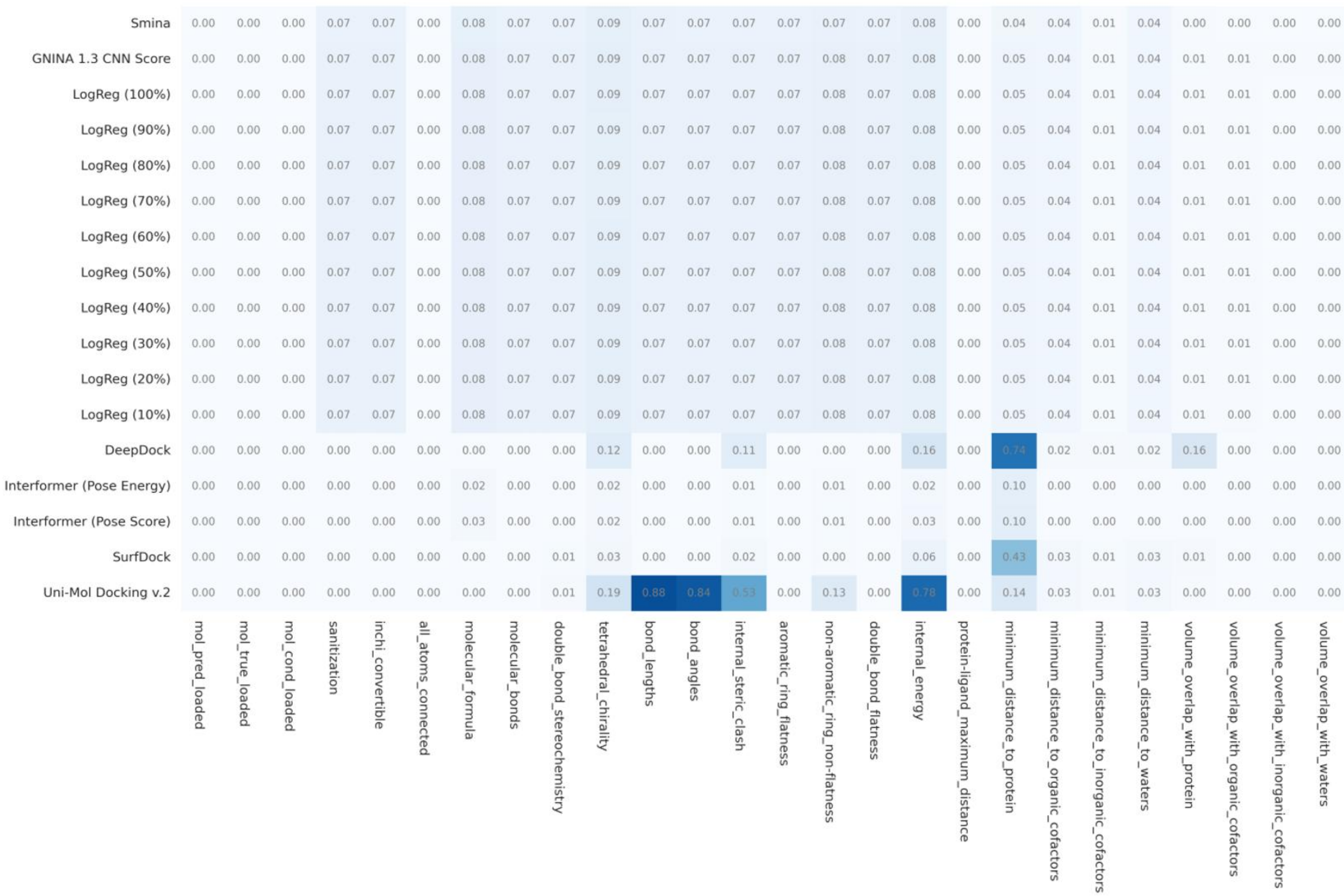

Fig. S5. Heatmap showing the proportion of top-1 poses (i.e., those ranked first per entry) that were near-native but PoseBusters-invalid across 5,201 PDB entries of the full test set, as predicted by Smina, GNINA 1.3 (CNN-based rescoring), logistic regression rescoring on Smina-generated poses, and four end-to-end ML docking frameworks (DeepDock, Interformer, SurfDock, Uni-Mol Docking v.2). These poses satisfied the RMSD ≤ 2.0 Å criterion but failed each of the 26 PoseBusters physicochemical validity checks.

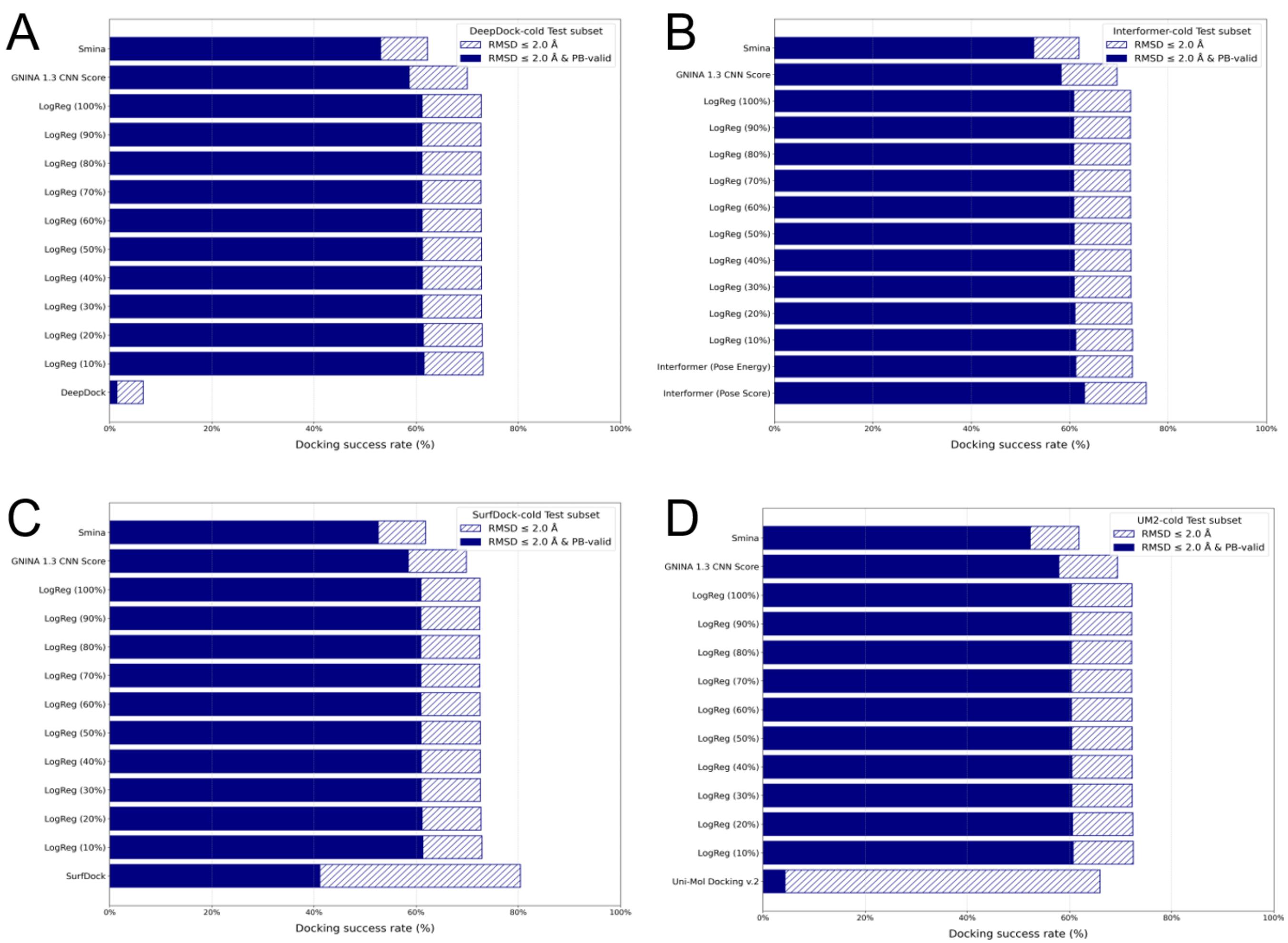

Fig. S6. Docking power of Smina, GNINA 1.3 (CNN-based rescoring) and logistic regression rescoring on Smina-generated poses, as well as four end-to-end ML docking frameworks, expressed as success rate (%) according to near-nativeness (RMSD ≤ 2.0 Å) and PoseBusters physicochemical validity checks, evaluated on on each tool's corresponding cold test subset: DeepDock-cold (A), Interformer-cold (B), SurfDock-cold (C), and Uni-Mol Docking v.2-cold (D).

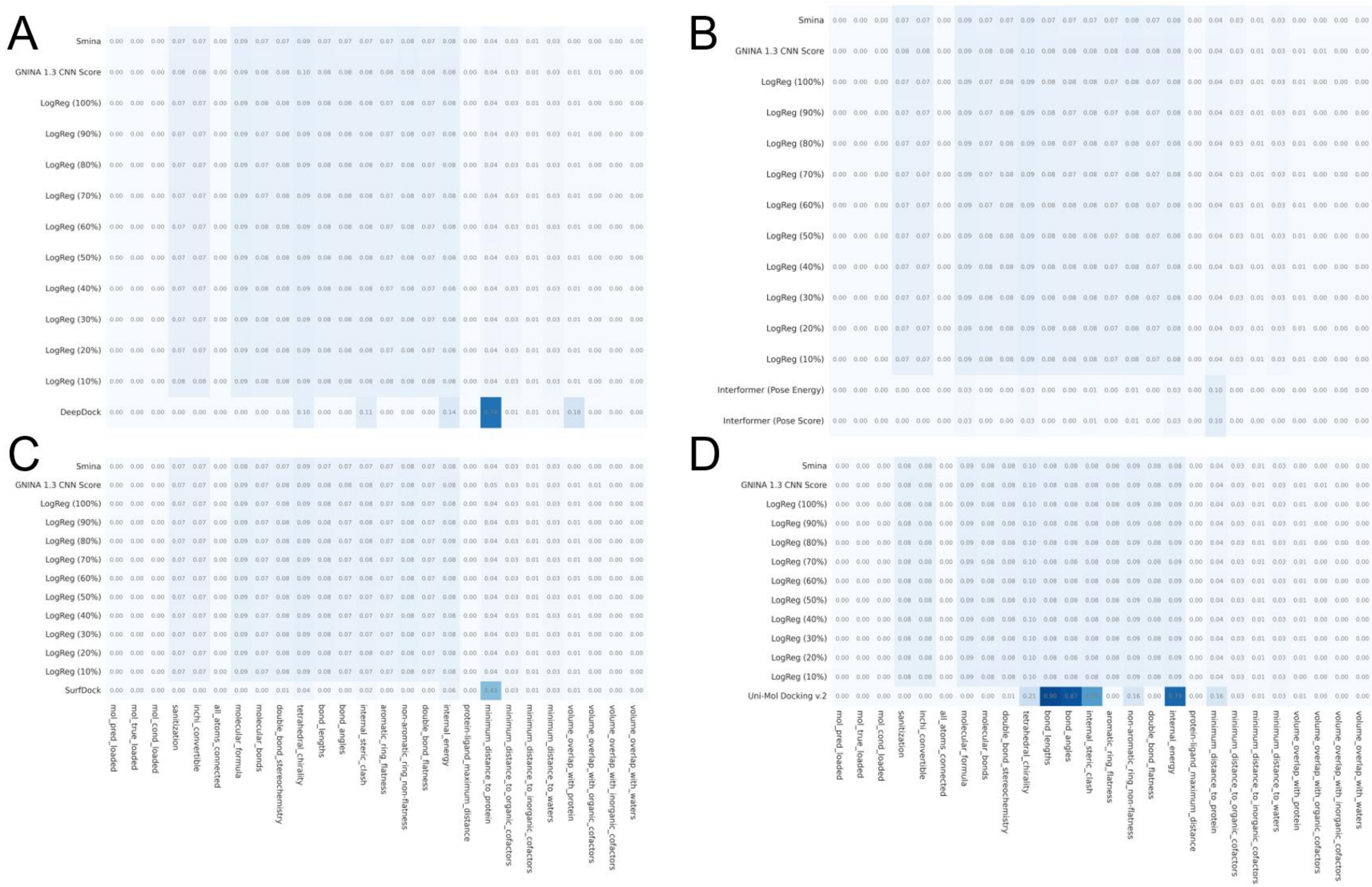

Fig. S7. Heatmaps showing the proportion of top-1 poses (i.e., those ranked first per entry) that were near-native but PoseBusters-invalid, as predicted by Smina, GNINA 1.3 (CNN-based rescoring), logistic regression rescoring on Smina-generated poses, and four end-to-end ML docking frameworks across their respective cold test subsets: DeepDock (A), Interformer (B), SurfDock (C), and Uni-Mol Docking v.2 (D). These poses satisfied the RMSD ≤ 2.0 Å criterion but failed each of the 26 PoseBusters physicochemical validity checks.

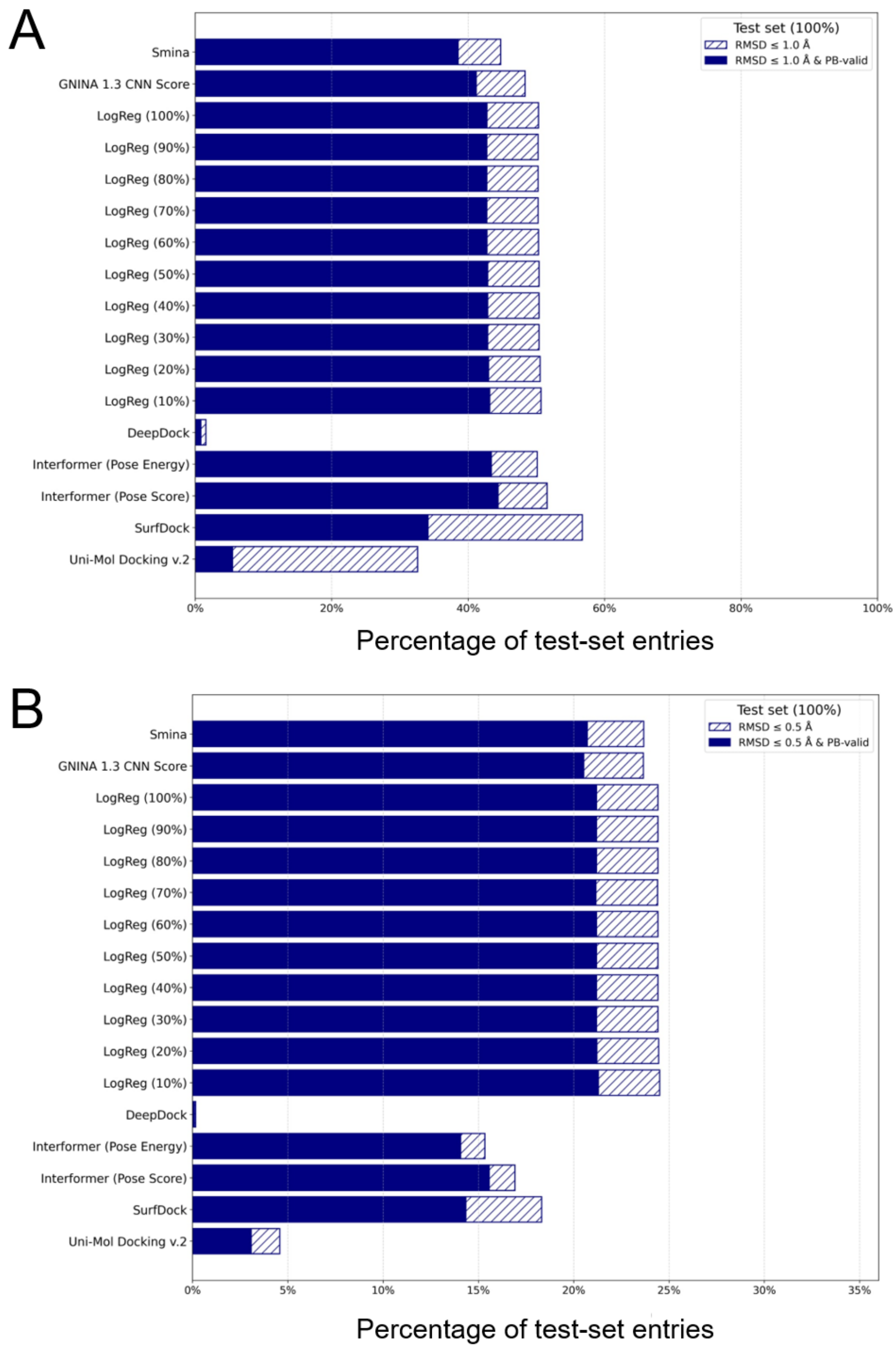

Fig. S8. Percentage of test-set entries for which each docking framework or tool: Smina, GNINA 1.3 (CNN-based rescoring), logistic regression rescoring on Smina-generated poses, and four end-to-end ML docking tools (DeepDock, Interformer, SurfDock, Uni-Mol Docking v.2) ranked first a redocked pose with RMSD ≤ 1.0 Å (A) or 0.5 Å (B) relative to its corresponding crystallographic pose, with and without PoseBusters validity checks.

Table S1. Classification power of 10 logistic regression models.

Model[†]	Optimal decision threshold	Balanced accuracy (5-fold cross-validation)[#]	Balanced accuracy (full test set)
LogReg (100%)	0.0648	0.8877 ± 0.0035	0.8722
LogReg (90%)	0.0671	0.8871 ± 0.0041	0.8719
LogReg (80%)	0.0654	0.8871 ± 0.0059	0.8723
LogReg (70%)	0.0735	0.8864 ± 0.0040	0.8711
LogReg (60%)	0.0730	0.8879 ± 0.0065	0.8710
LogReg (50%)	0.0697	0.8886 ± 0.0042	0.8716
LogReg (40%)	0.0712	0.8888 ± 0.0042	0.8711
LogReg (30%)	0.0712	0.8844 ± 0.0099	0.8714
LogReg (20%)	0.0692	0.8807 ± 0.0127	0.8717
LogReg (10%)	0.0901	0.8648 ± 0.0269	0.8701

[†]LogReg (x%): Logistic regression model trained on the x% training (sub)set.

[#]Mean ± standard deviation (SD) across five folds.

Table S2. Docking power of Smina, GNINA 1.3 (CNN-based rescoring), logistic regression rescoring of Smina-generated poses, and Interformer evaluated on the full test set using RDKit-generated ligand conformations as docking input.

Docking framework/model†	Percentage of entries with a near-native pose ranked first#	Percentage of entries with a near-native and PoseBusters-valid pose ranked first#
Smina	44.81	32.88
GNINA 1.3	55.51	40.37
LogReg (100%)	57.12	41.40
LogReg (90%)	57.14	41.43
LogReg (80%)	57.14	41.43
LogReg (70%)	57.12	41.42
LogReg (60%)	57.10	41.38
LogReg (50%)	57.18	41.38
LogReg (40%)	57.18	41.36
LogReg (30%)	57.18	41.36
LogReg (20%)	57.26	41.45
LogReg (10%)	57.26	41.36
Interformer (Pose energy)	48.34	34.28
Interformer (Pose score)	51.05	36.65

†LogReg (x%): Logistic regression model trained on the x% training (sub)set.

#Only one top-ranked pose per test-set entry (according to each method's internal scoring) was retained for evaluation.

Table S3. Docking power of Smina, GNINA 1.3 (CNN-based rescoring), logistic regression rescoring of Smina-generated poses, and Interformer evaluated on the test set after excluding complexes present in the GNINA 1.3 training data set.

Docking framework/model[†]	Percentage of entries with a near-native pose ranked first[#]	Percentage of entries with a near-native and PoseBusters-valid pose ranked first[#]
Smina	62.18	52.98
GNINA 1.3	70.37	59.12
LogReg (100%)	72.86	61.30
LogReg (90%)	72.86	61.30
LogReg (80%)	72.86	61.30
LogReg (70%)	72.86	61.30
LogReg (60%)	72.86	61.30
LogReg (50%)	72.92	61.36
LogReg (40%)	72.94	61.38
LogReg (30%)	72.88	61.32
LogReg (20%)	73.02	61.52
LogReg (10%)	73.09	61.58
Interformer (Pose energy)	74.32	62.86
Interformer (Pose score)	76.89	64.20

[†]LogReg (x%): Logistic regression model trained on the x% training (sub)set.

[#]Only one top-ranked pose per test-set entry (according to each method's internal scoring) was retained for evaluation.

List S1. List of PDB IDs included in the training data of DeepDock.

4ej8,1qx1,4p4f,1ajn,5flq,2x39,1ii5,4cqf,3fj7,3m8p,3ut5,2ydv,1uxa,4huo,5tjx
,4yrg,6g85,6hdo,1vgc,3zmg,5oq5,4j3e,5jf1,4uvc,1a08,5ubg,2cem,4erz,2wsx,2ow
6,1idb,2v2q,1y98,6f3g,1s26,3pjt,4dfu,4yyn,3ixg,2bes,2xaf,3s9t,3puk,5m0s,3g
cs,3rak,1f5k,6dvo,5ei2,1i7i,3new,4wks,4j78,5y5n,5lws,6fhu,5y86,4wkc,5nho,2
dxs,2ohv,2bz8,1o36,4cli,3hdm,5wb6,5hkb,3l17,5agj,4i60,2er9,6bmx,4e3g,1yly,
2q2n,3bc5,5ipc,4rll,5mi3,5ix0,3o6m,3hlo,3el7,6evq,4mf0,2xkf,2w10,3ijz,1fq5
,4omd,5xmr,1jil,1o5g,5lpj,5afv,3zst,4oel,4m3f,3ekx,3vw0,2yz3,5ak6,6cki,3rg
2,4h3c,5er2,3p78,2anm,3gcp,1q66,1b2m,4oba,4mfe,2vwv,2ofv,1uu3,5anw,2w54,3f
ed,5tcj,2a3a,2v87,2m41,2wev,1wbv,5o9o,2ovq,3rl8,1xlz,5i29,1ydb,2zxd,3wci,4
tz8,4ad3,5jjr,4rdn,1wbg,4z2j,2x7u,5cau,1npv,5o9q,6fut,1sve,3rsr,5tw3,1t4j,
4dgm,5yj8,1g7f,5vdp,5dxe,4b7q,5alx,1rzx,5fdz,6ccs,5vc5,2wkz,4aua,5mgm,2h42
,3g0e,4fic,3d0e,5d0r,3ej1,4odn,4mdt,5two,3odi,5dhr,2vmd,2ow1,5v5n,1i6v,2ai
g,5h0e,3m6p,5dde,4e4l,5csx,3e90,5akj,4do3,3vyd,5oss,3k7f,1bwb,4u2w,4oag,4m
q2,4bg1,5khk,2vt3,2fde,6fa5,4ql1,5ntk,6g86,5ejl,4mrd,1fig,2vqt,3t3u,1vyf,5
ive,1h1b,5mfs,5ekn,3bwf,4nuf,5wg7,1e34,6g9j,5l4e,5kya,3ggc,1ox9,2irz,4k60,
2f71,4osf,5fb0,5k32,4yxi,4dgg,3arz,4ze6,3kqb,4j2t,4cq0,3mvm,6f1x,1ugy,3k5x
,4nq6,3ia6,6hjj,1ctr,3kce,5ea4,4i9h,1q5l,4mgb,5ii1,3vhc,1a85,6g0z,1bzh,5e2
v,2qn2,5cil,1j19,4wzv,3tic,6epa,6gue,5n7x,5el9,3f88,1uef,5alk,6mv3,3tyv,3l
w0,2g6q,2w8j,2j3q,3bi0,3k99,4w5a,3owb,4daf,5ti4,4nnw,4zx3,3oe6,4gzf,4o3u,3
svv,4pb1,5f4n,3tu9,4gm8,4jt9,2w8w,5wag,5mhp,4cqe,5twj,2c9t,1x8b,4bie,3h2f,
3fe7,5ggn,4o9s,3o9p,3d91,1ynd,5mpk,3ws9,4zxy,3l4z,4hvg,5wei,1w8m,3k0k,4zz3
,1hvk,3cx9,4n9d,3nf9,3nex,3qup,4uy1,5f1z,2e9d,1d6w,3iiw,5nkg,4l2x,5n53,5mp
z,5ald,4joj,4bqs,4yw6,4xcb,6euz,1w70,5i13,1tou,3ti3,3amv,3udq,1yyy,2yne,5t
q5,1yvh,5kby,2vpg,4ajk,1kak,5enf,4zo5,5fwg,4mwq,3zxr,3kyr,3dda,3f68,3td4,4
tkj,6d5g,6f8b,3qkm,2pu2,2vwz,4d1s,5en3,5cuq,2qiq,5l6o,3p1d,6dzq,5he1,5v4q,
4hwt,3c6u,1w9v,6ar2,1iq1,1o0n,4deb,5w99,5dtk,4inr,3mw1,4dv9,4nms,4bi7,4k6y
,5u7m,3r6g,3l5r,2y2j,1lgs,6hm2,3snb,4zzx,1t4v,1v7a,4qms,2x6w,3rkb,2ha4,2i2
b,4rx5,3e62,1h0w,6hlx,5bqh,2gg3,3djk,4zyw,1r6z,4uyg,1f8c,1zgv,6gji,1rdn,6e
dr,3uvp,4jkw,3che,3umq,2fm0,6cpw,4n1t,2ay9,4e3o,2qnb,5nn6,5g6u,2qcn,6etj,2
bua,5gtr,5loh,4qmz,2oiq,3kc1,3zov,1f4g,2ddf,5fv7,1o34,4avi,2zdm,5xs8,3qcs,
1hvy,4j0y,1g1e,5kde,3rme,2aa6,2r5d,4bcb,4crl,3tjh,6bh3,3sue,5uc4,5jcj,3h0a
,5c5h,2hqu,4hvb,1d4h,1o47,4deg,6cvd,6bfe,3dnd,1f74,5vwk,2xxt,3s2o,5j8x,2av
o,4qjx,6ct7,4fxy,3pcb,2ri9,5jog,1k1n,4fns,5txy,5lvn,5nr7,6mrg,5bpa,4ks2,2f
zk,5ov9,4rh5,1n9m,2wzy,2gm1,2qzl,2xui,3vry,3qzv,4b7r,3l79,5t2p,1rt9,4fz3,4
qem,2i2c,1fpi,1gzg,6gin,3ws8,4a50,3cgf,4mlt,4zla,2j83,3zeb,2pbw,1jcx,1x39,
1wcc,4kju,5u4c,3shy,2y6d,1lv8,5xva,2pqz,5ngt,4eoh,6cjv,4y3b,4ymj,4li7,2vvo
,3uqf,4luo,3u3z,5hcx,4ggl,5fum,6c7x,4kbi,2flr,3m3c,4bd3,3iwy,4azp,2z3z,5ed
u,5za9,5tef,2g70,2wxl,5w12,1n3z,3psd,1egh,3hb8,5h21,4tsx,3uvq,3dkj,5y2f,5v
se,3o5x,6ck6,5ct7,4q99,5ofw,1leg,3f35,5mro,4rpo,4qtb,1g98,4pkw,2p8h,5acw,2
q70,4qt2,4ciy,1ets,6g34,5fah,6clv,2ya6,2gmv,1gz4,4mq1,2v8w,4cj4,4hyf,1oiu,
5lmb,4djx,1qi0,1mmq,1qj1,1t7j,5odu,3d7h,5jq9,4i0r,5i9z,2yis,4zyz,5eng,6gl8
,3ft2,1q4x,1ykp,1i7g,3hqz,6df4,5afk,1yej,1o41,6d1u,1toi,4q8x,2zdl,2rnw,4z0
e,5f2k,4jpe,2cgw,5ueu,4u5v,4tpt,2bz5,4i12,1sw1,4q9s,1c88,1fpp,1x07,1ov3,3d
jp,3vw1,4ayx,5khx,1n7m,3u2q,3sjo,6fgf,4lw1,5am6,1bm2,3f9y,6fyl,4prg,4urv,3
pa3,1p0y,2bza,6bh8,5ygf,3o8h,1h4w,3kr2,4u54,5k48,2y55,3rf5,1jd6,4acd,4old,
3b9s,6g9i,2v88,5cep,5cnm,1ik4,1h07,2gg0,4y2p,5y5u,4tjy,1fsw,6ed6,6fav,3f8s
,1fq7,3rt4,3cm7,4hvd,4tky,2ceq,2ohm,1k1p,3st5,5toe,5n99,2xyr,3w2o,4op1,4m4
8,1q6m,3ine,2ndo,6eln,4zt4,3vyf,5ya5,5vqi,2oh4,3mlb,3fr5,2vcw,4gao,4w9p,4r
02,4zz2,4ypf,6mwe,5za7,5fdd,5t6p,5a14,3rjw,3ehx,5g17,3bcn,4lno,2pj5,2iyf,3
nsh,3d1f,3jqf,5cyv,1q83,4url,5c0k,2ywp,4umr,4o7d,4mbj,5nka,3r92,4oyb,4cfm,
1bv9,2p3b,3uda,4mzj,6cms,1adl,2jdu,3snd,4o7e,5u7l,8cpa,3tl5,5o1a,3shc,4mao
,1qiw,1xnx,3hzy,5wjj,5ug9,3br9,1bn4,5ofu,6di0,5hvp,4gj8,3dv1,1nmk,5j86,2cm

b,2f9u,5yh8,2q5k,2r5q,4pvo,2pow,6eku,5tw5,3eqs,4hnf,2xgo,5lz5,4jx7,1il5,5u
l1,5i2f,5oyd,5ey9,4aci,5eb3,3ty0,2wxi,4del,3pch,6c0t,5xyx,5d29,4n9e,4l0t,4
ohm,2cer,5aqj,2mwy,1o4b,3sna,5mnc,1m9n,4gy5,5j3v,4djr,1q4l,5u12,4l7h,5cr7,
5wzu,4wh9,1xot,1x8s,4uat,3oap,5evk,4os1,1y6q,6exs,1a1b,4kij,5cw8,5wxf,1yqj
,2esm,1fpy,4b9k,3o0g,4uff,4bs4,3qqa,5mlw,3cfn,4jzi,4ci3,6chh,3pcc,5fqb,3hf
8,4o0v,2wnj,1qhc,4nw7,2jbj,3u7l,4x21,3sx9,3r8i,4tu4,3gpe,4nat,2rjp,2rgp,3e
hn,4ocz,4q7s,1jfh,3nil,4op3,3cst,2hha,3mxd,5hpm,2za5,2wd3,1mmp,4g3f,3deh,2
bo4,5vs6,6fdc,1ouy,3v5t,5ta4,3v43,5u4e,5c8k,4zx9,2qd6,3rpv,1loq,4hcz,6f05,
5ajp,6eqw,4ayy,3ipa,5ggp,5jjs,1hiv,3kiv,2wbb,1dhi,1fmb,4joo,4qvm,4bci,4acm
,4o77,6ebe,2o64,1nfw,4q2k,4o0t,4r75,1a4r,3q71,2e7l,4wph,1tx7,1ppm,3lxg,5nw
1,2b5j,1wv7,5f00,5dp9,3bet,3ddp,4c52,4je7,4hgl,5mo8,3dg8,3h0j,5kv9,4whq,1o
l1,2e98,4hw3,3fup,1ec2,1a9u,4mwy,2xzq,3c4f,5vqs,4nyf,6cck,3l7a,3s3o,3az8,5
qa7,3v4t,4mbp,3arx,5tku,6h2z,3mp6,5ek0,2ha3,3i02,4bqt,3ew2,1zc9,5eie,5yqw,
5f2p,3rbu,4u0m,1x6u,3a6t,5e13,4r0a,2bge,6dj7,4ks1,2f80,3uil,1h8s,5uff,5f5z
,3odl,3uix,4lte,1nqc,4kzl,4bbf,4oho,5wr7,4wtu,6dxg,4l4l,4p4j,2f34,5cf8,4fg
t,5xvg,3bym,5kqg,4dhr,5ehy,1nym,5ml6,4y2v,4n9a,4guj,4jmg,5eg4,6gjy,5mw6,4e
hg,5tg4,5lj0,6b4u,3oy1,3ekp,4p6e,2v3d,1r5w,4fci,1swp,5nsp,5k0m,5eyr,2vr4,6
cve,1oir,5ost,4i0f,4pee,2oh0,3o75,3zsq,1qxw,5ei3,6dq6,5ut6,2xk6,2chm,1ogd,
6azk,3gnv,2yhy,2vr3,4h3a,2wos,3jq8,3drs,4qt0,4pm0,3ove,1p01,3hg1,4ge1,2r5a
,5afn,1ukh,2oah,6awo,5tv3,4kiu,1v41,4hev,3lmk,1xlx,2pv2,4l02,5trj,3twj,1a0
q,1ogu,5o1c,1g05,4zt5,6f8t,4z7h,6g0w,3bgq,5o1i,4up5,3v7s,5un9,6c4g,2wnl,1i
bc,1k06,3jqg,2gkl,4kzb,2psu,3ctj,1gnj,5bs0,5dri,3ap4,2w2u,1a7t,5orw,1xpc,3
d50,4lil,4omj,4wsy,6cfc,4odm,1qbr,2xef,1srg,3m8q,4km2,4os4,4dst,3dyo,4p7e,
1pun,4wrq,5fxq,4np9,4ejl,2rox,2v83,5o4s,4rn2,4v04,3sur,4epy,4xh6,1s63,1rhm
,3ud5,5gvl,5hdu,3nb5,4wkp,1i72,4ly1,4ps1,5hmh,4h36,3wut,3ipx,1a4h,1p4u,1t4
e,3f0r,3fzr,6g3o,4qvq,2nwn,3ux0,1zrz,4bda,6b8j,2g79,5j5x,5emm,1o3i,4agc,3t
km,5c7e,3tdc,5b4l,5lzg,5llo,1xh8,1eld,2viz,6bhv,2wf2,5wzr,4k9g,5cqj,3t4v,4
wkt,4hbn,5vee,2gni,2vsl,5lgs,4urx,2joa,4e1n,4l4z,4i2w,3g1m,1nhg,5x8i,4h5d,
6i3s,1re1,3g2w,4a4l,5g45,5a0c,5myg,3zpr,3lk0,3vqh,4xmo,2lbv,4aro,5h0h,3v4j
,1kjr,1exv,3vc4,1slg,3l0v,1om2,4nyj,1kf0,3lc5,4uiz,5g3w,3pa4,1jvp,5o07,6c0
n,3wch,3v5j,5hx6,4oq6,5v6y,4app,3lbz,1oif,5f74,5ntw,4ps7,5wac,4jv6,1lhg,4u
8w,1k6t,5hex,1dfo,2lha,3e9i,3cjo,5l2z,2ynd,6fiv,4cc7,1no9,2buv,3c0z,5eqq,5
w5u,1oit,5g2n,6mvx,5zah,3m3x,3mrx,4mq6,4jbs,2i5j,5i3w,4ngt,5t4f,5d3c,3pj3,
4g1f,5nvx,2va6,6eaa,3at4,4mro,1dwd,5vyy,3ckr,4fl3,5os1,2q7q,2q1l,4jmh,2o4k
,1v2j,3p3h,3vrt,4led,2xl3,1lb6,4ogn,3fi3,5bry,5c2a,1wxz,5dkn,3mjl,5tzy,4bk
j,4gih,3p55,1ajq,5fs5,5y0z,5aux,4n7m,1c4v,1kpm,3iw7,4fev,4o74,2kdh,4qp2,4w
f2,4dwk,4uxq,4ufg,5ew3,4bzs,3v3q,3w54,2nn8,4u5l,2wxh,4jju,4l52,3krr,5e5g,3
fsm,1cet,4e5d,4azy,1t31,1bmm,3qxv,5zag,5xsu,1lhw,3vb4,1aj7,5nfh,3bu8,3p4f,
1fv9,5eel,3rxg,4is6,5yg4,5vdw,2ivz,3l81,4e4a,3rwq,1q7a,4i8z,4kot,3e8r,4n4v
,1o3p,2oz2,5lhh,1uze,4jai,2pqc,2f6j,4bh4,3ldq,4ode,5n16,5vgo,5j7b,3wqw,4kb
c,6chm,2ath,4ezq,4v05,1y6b,4gtm,3rk9,3eyg,3thb,5dg6,3k3a,2aog,4p4t,3a5y,3n
nw,6hm7,3doz,6hkj,4uvy,5mi5,5al4,4rcd,2in6,4k3o,3bva,3sus,3pwh,4csy,4i0s,6
ax4,2nqg,3bkk,5f6u,5ewy,1b9s,4zyc,1o4r,5mpn,5u49,3upf,4yur,4rrv,5n3w,4pks,
3nyx,2h9m,3uoj,2buc,4jyv,1q4w,4p74,5l8o,3chs,6eip,3azb,5q0x,4wvl,3hha,4a95
,2mov,4anq,3rk5,2y76,2c3j,1elb,1sb1,4btx,1vrt,5mkr,4fxp,5huw,3iub,1o2w,5iu
7,2d41,5k03,5l4h,6bhi,5als,3u10,4ps3,5z1c,3juq,6eog,2wa8,1xz8,4o4g,2byr,1h
xw,3ejs,3ern,4dn0,5caq,3ftw,1nh0,2rfh,1wum,2bve,3faa,4q4e,5afx,3ip6,6fby,5
ezz,5dva,4iho,4bi1,5vds,2srt,2vur,5mwj,2i5f,5m51,2x24,5f3g,1dx6,4ovh,3f7i,
4crd,4jlm,5oxl,4eoy,4e3h,1g4j,5l3g,1i8i,1h6h,2poq,5lwe,1nhw,2zx9,4d1b,2is0
,3rx8,2xyd,1yqy,5vt1,4yh4,6h7j,4j06,5cls,2an5,3lk8,3g6m,4n7u,6ezq,5zo8,4yy
l,4cc3,2e1w,2g19,4za0,1hps,4tuh,4knx,4pnm,4rt0,5y93,6eqv,4rd3,2g1r,4bb2,5o
ui,1fkh,3lbl,5ggl,1jak,4jg8,3b8q,4b70,3tif,2j7b,3dxm,2il2,1nwl,3ndm,4pmm,4
nra,2yiv,3csl,2g1y,4a6s,5cvd,2idw,2hwo,5nfb,1hte,1ym4,4mo8,2pvw,2ewa,3qci,
5etp,2xez,3eko,1bmk,3r8z,2uwd,2bts,1you,5w4v,2osm,4uro,4k6u,5y3n,4bqy,3jsw

,1pwy,4hzx,4dma,4kp6,5fto,2ylp,4zyr,3a1t,2kzu,1rej,1g2a,5lbq,3lbk,4lxk,3k4
8,2qmf,1xd0,5c8m,4eh9,3wix,5k4i,5zeq,3qu0,1a4g,3wq6,3atp,4x1q,1ez9,3o84,5v
qe,3r24,3rz7,5k09,4mwb,3eml,2r0y,4j5d,6ckc,2rly,5aic,4ewr,2clv,2o1c,1b8n,4
mr4,6gjn,5zzw,1n5r,4txs,5czm,4lwi,5gmj,2wyn,4twc,4clz,5tq3,4csd,5xmt,3iaw,
4o9w,5ijr,3uvm,4hnp,4acf,5f2f,4zz0,6f6s,6f22,5wg6,4zhm,3kmc,1u2r,4qzx,3ess
,4d85,5ixt,4meq,1o42,4myq,2nq6,1gny,4w9w,3sw2,3s71,6eqx,4rj7,1xbc,2f01,6f4
x,6coj,966c,5u6d,1h3b,3rux,2gtv,2xae,5t4b,4f65,3sk2,3fuc,5ece,2y6c,4a4w,3e
ta,4uof,1rgj,4dea,2e27,3ujb,4lh2,5dda,4ww8,5em9,3qkd,2k4i,4mue,3qlb,4fmu,5
ni5,5tg7,4dzw,5e90,5nhh,1g4o,3kjf,5j1x,3m56,5yc2,5vdk,3pkc,3hqy,3n5u,4hzt,
5qaz,4raq,2pll,3pce,5ccm,4xt9,5nwg,5t6z,4p4i,5lz8,4b1d,3n1v,1h24,3zy2,1c5c
,4uuq,4qf9,6mxe,2er6,5urj,1met,5ldp,2xk1,3kbz,3tfk,5id1,4dv8,4mex,6gxu,2du
a,3svj,1rlp,5m9d,5kq5,1pfy,1o3g,2r9b,5jer,6agg,1ggd,5hg5,2a2x,5g5v,2fw6,4x
g8,2gh7,1m0q,3vvz,5xw6,5mk3,2d1o,5i9y,3h5b,2doo,3avz,3d25,2w3k,1mtr,3el5,3
arn,4u5t,3lgp,1oi9,1xdd,2wtj,4l7u,3s43,1flm,2bpy,2ria,4h1m,5wlo,4m7x,1oe8,
2pym,4ded,3o3j,5ugc,5vqw,5sxm,3n46,5hu1,3e8u,4d2d,5tuz,3o23,3b1t,4qh8,3tne
,1n1v,2vta,4lxd,4mk2,5ne3,2y36,3l6x,4fyh,2q54,6g2n,3b5j,5e3d,2uzo,3qbn,6g1
w,3lpk,4ezy,4x3s,1okw,3rtm,5o1b,4zph,4b5s,5dx4,2yjw,1kc7,4pl5,3ds3,3t1n,3u
bd,5em5,1fta,3abu,6aol,3fv7,5op4,3s7b,5nih,4b2l,4gnf,3ska,4an3,4u0x,4m5j,4
ivk,4h2m,6eyt,5h1t,1uy8,4pli,3zha,2pcp,5x79,3i91,3t6j,1fax,2ltv,4dgb,1juq,
5c13,3ifo,2x97,4hbw,4bkz,5gso,4b12,5cso,3ivc,1csi,1xh5,4c9x,3agl,5jnl,6fnx
,4egi,5jhb,2jk9,4l4m,5ha9,3zxh,2auz,4x7o,5dyy,1sgu,3nii,1qpl,2ban,1bnt,1ut
c,4eos,2vaq,3zcz,4kpx,7std,6b5t,2pre,4qmn,2y8q,3bim,1nyy,3umx,3if7,2fie,5g
4n,5ect,1jqy,5epn,1y8p,5hgc,5ywy,4efu,6ey7,5ubt,4zuq,4nwc,6f7q,2zc9,2ych,4
o7b,1sqp,4p2t,3p3t,3b8r,2avv,2zdt,2g9r,5j74,3sl8,5wlv,5i83,6bjo,3ebi,5est,
4aoi,3gi5,4ef6,3o0u,5tg1,4qxt,1yrs,1q0b,2rip,1lt6,4u0a,3kck,5dms,4j0r,4bfy
,1r5h,3f6h,4a6l,3fkt,3p2h,4zwy,4jr3,4z5w,4xsx,2iog,3p9m,5hn8,3eb1,1mj7,4um
a,5vd2,5o5a,1hyv,4fkk,3s00,4azt,3gta,3o2m,5ny3,5fb1,5e8w,5wxh,4cfe,4k72,3g
po,4ejn,2z52,5ou3,1o4a,3u4u,5n8w,4pb2,1o2r,4ci2,4qvp,4u0e,4ono,3iu7,5nar,5
c4k,3p4q,4o7a,2ok1,4lar,4amy,6csq,1bky,1vcu,3zmm,4gsc,5wqa,1qcp,2zlf,1n8v,
3hy9,2gh6,4y3j,3rcj,1tt1,3s8l,3zmt,6b1k,1ct8,3rxj,4u01,5dqf,5isl,3in4,3jzq
,3c49,4e5j,5fom,4tyt,4o7f,4xdo,5aqt,4zzd,1kyn,4s2i,5nai,1pq3,1j17,4ycl,1c3
x,4x5p,4hlc,1r1h,4y46,5kzp,3g1d,4vgc,2pvm,4q9y,3cd8,1hkm,6asz,1qsc,1g3m,5k
8s,4tya,4mqu,4l0l,3e6v,2h15,2xm8,4uwh,4cr5,4g0z,1str,3p0g,3fcl,4jv7,3s4q,2
cle,4d1d,5edc,1mfi,6dh1,2wyj,1nxy,5yic,4rcf,4ogv,4yz9,5nf5,5a3x,5vo2,2bmc,
4rao,2xu3,6c91,3hj0,3g32,1v3x,6g9b,2ylc,1zsf,4btm,1b55,4yuy,5gj9,6fim,1w1g
,2glm,3hu3,4hla,4zpg,1jjt,2xbx,3r6c,5a3t,5eue,4utr,5td2,5fh7,5wgq,1yw8,3rx
e,3msk,6few,2c6l,2axi,3mtd,3mi3,5yto,3eys,2k62,3r0t,4rad,1ywi,3zy5,3dpo,4e
o6,2idk,5trk,5ttg,1s17,1qbv,6eiq,4ivt,4k66,1ajp,4riv,6hvh,4zz1,5n9r,3buo,3
ikd,2r3t,1g9t,1pl0,2c6i,4g5y,4nvp,5wgp,3bx5,3ooz,4jg7,5hcv,2vti,4p5z,5i40,
4qp6,4ucu,5mjb,5u62,4gv1,4jxw,4hxq,5kva,1ckb,3stj,4qsv,2e7f,1jmf,2x4u,2j4k
,4g0p,3gfe,1o3l,1hsg,5d1t,3zln,4bcp,4d2r,3g0b,4c4h,2xoi,1hvi,1uom,1ws1,4g1
9,5yc8,3dpe,3rxl,6ffs,4nus,4ny3,1ove,2viw,5of0,4r6t,1kcs,4exz,3l13,5akg,5y
e8,1fh7,5v35,1qon,3krd,3r5n,3ms9,3neo,5zt1,5nw7,2on3,1u9q,6dh6,5y97,3n8n,6
m9c,3ql9,4i0d,4xv3,4ek9,4n84,2jkp,5ti7,1tps,2w5i,5z7b,3ucj,4yay,2ql7,6hh3,
4c9w,4x8p,3skc,4lm4,4hyb,3w2r,5o5f,1o33,1ghy,5n1p,2r7g,5n18,4trc,3iog,5cqu
,5vij,2bjm,2cbs,6gu4,5mgg,3t2t,5kcv,2ydj,4w50,3rv7,2a14,1ndw,5div,1f0t,4hy
s,3u4o,4m7y,5id0,1s89,1bdl,5exx,5j5r,6eab,4k5z,2r3g,5sz1,4oyt,3skf,6ffn,3b
tl,6dvn,2wxq,4ynl,4xtv,4ycw,3ptg,5h0g,5e8r,6exi,3g4f,6fuh,5xpi,2z50,2nxl,6
h96,3ncg,4iwd,3sgx,1syh,4nyt,1hq5,3u8w,4ppb,4ayp,4lov,1yid,1dl7,4e20,1ql7,
4rvk,4zcw,5sz6,5mrm,1gaf,1b42,1nhv,4lwh,1a61,1jqe,4mpn,4qvn,2ll6,4w9e,5ely
,5nve,2rnx,4i06,5cxh,2zcs,4fl1,4anm,6ay5,3pi5,4ty9,3qaq,1we2,3avl,3eeb,4o5
b,5ddf,5b6c,2ank,5l9o,3r4n,4yvc,3jpx,4e93,1ony,2i3z,3d1z,1syo,4wnm,3r17,5m
ay,3iaf,3o5n,5n2d,6bl2,5th2,5naw,4wvt,4y5h,6aum,3cph,4zhl,3p7a,2clx,3s6t,4
qga,4ryc,1ny0,4non,5uwl,4p10,6miq,5ivf,5aib,5e1e,1sje,1jwm,1sbg,5d9p,1ork,

2vtl,4a6b,2aoh,3c5u,4ptc,3ie3,3okc,5ivz,4yc9,6ful,1mcz,5uz0,4ydf,4gwk,1sle
,5u0e,3nu5,3sc1,4qwu,5a6i,4wx4,5aaf,3h3c,3gcq,5w7x,3gsg,3p3s,6drt,1agw,4js
a,1vr1,4lxb,5j3l,1pr5,4yx9,3r2b,5k6s,5lgo,3jzp,1hs6,2lkk,5mts,5ye7,3r2a,4j
zd,5mav,6fa4,5qav,1ypg,5yij,3fpm,1bqm,5ldm,4z2l,1a8t,5t1m,5nvv,2xzc,4dce,3
vnt,4lk7,3ppj,5lz9,4xyn,4btb,4ght,6b0v,4ie2,2e95,4tyo,4ufy,5ek9,4q6r,2y59,
4q0l,3u8d,3bxz,5mlo,3mbz,5uiq,4qjr,4j4n,2yj8,4pv0,3qip,3d8z,4kjv,4w5j,1uyc
,1ttm,4os2,6fug,5ekj,3tpp,5wzs,2r3p,5fdi,4yff,5gjf,2m3m,6ej2,6biy,5n34,5vk
m,4ara,5t2i,4dus,1lpz,3h23,4foc,5ix1,2wmv,4ciz,3u15,3vtb,2aac,2aoe,3ipb,5l
30,1o2n,5edd,2xy9,4qsm,5d10,3l18,4obv,4r7m,2j2i,1o48,3bmy,6hly,2gde,4gtq,6
b27,1k1j,3hik,1lf3,1y3n,1xp6,5lwn,5b0x,5vfc,5duw,2e5y,5wae,2b07,3fdm,1rst,
5eyz,2vrx,5teg,3k27,5edi,2ie4,2c02,4x3u,4lkh,4jql,5fnc,4a0j,2ajd,4ui5,4i33
,5nb7,4psh,1nu1,1afl,4puz,3kid,2vew,3p4w,6bmr,4ud7,1gni,4hj2,4g2f,4y59,2vi
n,4oas,4f9v,3b2q,5vqz,2c3l,3tll,5kjm,6boy,2xbj,4lm3,3io7,6bsk,4h42,5goe,4n
h9,4li8,1ywr,4um3,5b5o,4fmn,1inq,4emv,3obu,2hdr,5nn4,2jql,5g4o,2wd1,3dz6,6
dif,3hac,3ppo,2i0j,4r5b,1c5q,3cen,2y4l,1l5s,5fck,3rzi,3cy2,4k3l,5wad,1gfz,
6dar,2zn7,6gih,3l5d,4uj2,4zh4,3jzh,3bxe,4b73,2f0z,2yel,4ktc,5qau,3kec,5m23
,5ddd,2vnp,5o4f,4ega,2xxn,4aac,2gyi,3e81,3uec,3bug,4lh3,3wcl,5kcx,4mh7,6c4
u,2v57,4f1s,4u93,5kpm,3zpt,4b13,1c3i,2gz2,4kn1,3gqo,1pig,2ha0,5zia,5jq5,4k
0u,5lom,2d3u,4ona,6bbx,2q6b,4muw,3wcg,3sut,3qi3,3g2s,3ti4,4mbi,3zlo,5u6j,5
qah,5q0f,2x8d,2xu5,2vts,5gwz,2oo8,3pbb,1h3h,1oeb,1qyg,3zt4,4zt6,1v2v,4mot,
4u7o,3n9r,4qsx,5wkl,4ea3,4g90,5oh9,2qnx,4kao,4nal,3djq,4y83,4jss,1k6v,5ctb
,4j0t,2vh6,3ti5,1bux,6f9v,2n7b,3rxh,5j7g,3l7c,2hug,4wf4,5itd,3mb7,2qtg,3kr
1,5ett,4da5,4cd5,2qtn,4x50,2wf4,1qxk,5lhg,1xh9,1q9m,4o45,3pd4,3buw,2xbp,4u
ye,5v24,5d48,2r7b,1o4l,5lx6,6mim,3hrb,1sqo,4p0v,4aa2,6eq8,4gqp,2qpj,1nlj,2
bzz,6g4m,3kxz,3m5a,2bgn,4uu7,5jek,2qcd,4re9,5ema,1h01,1n7j,2nq7,2fzc,1zp8,
4own,3s8o,5ayy,1rs2,4m84,5w94,5trg,1h61,5tln,4llp,4wf6,4g2l,5t3n,2wo8,1tc1
,3hjo,1mpa,3sgt,6g37,3nf6,4gny,2j4i,3qce,4zbi,4pnw,1v2q,2cng,4avs,4qtc,3fq
l,3ttp,4lts,4b8p,5bwc,2c0o,4r3c,5avi,6q3q,4knj,3u6a,6g3a,4dem,1w6r,3ztx,5b
vf,2qi5,5qaf,4m2v,4m5m,6fg6,5nzo,5hyr,4jib,5nhf,4pda,3fc8,5y6k,2exg,1h46,1
o4p,4yes,4em7,6evm,4ynd,2xjj,1ero,6ayq,1g9d,4qlt,4je8,3vbx,3rdh,5njz,3kqr,
5em3,1swk,3lpl,4c4g,4og3,2qc6,4knm,3hiw,4xcu,4zw6,3vb5,5qad,5up3,4afj,4qgi
,2x7s,4q1e,3s56,3rm8,2vpp,4uub,1pi5,6fap,1s39,4qf8,2jdk,3gjw,3i3b,3dwb,3bt
j,4bfp,2zdn,1a1e,3tnh,1ida,5aqz,3t5i,2ycm,1tl7,3ka2,6mjf,6iil,1y2d,5csz,3q
lc,5i88,5n1r,4d1j,3wdd,5v8v,2chx,5uir,1r5n,2pvv,4r5y,6guk,4az3,4gj6,4lpg,2
j50,3cf1,3zqt,1qk3,1njc,4f6x,6b7a,5hfu,2wez,4q1d,4dkq,5uit,3s53,2fda,1e1y,
5bvk,2wxk,1pxk,4caf,5k0f,3rpy,3fue,5o5h,5tks,2wxd,4kww,6fnt,3dgl,4hg7,4w4y
,4hxw,4ewn,2who,3d20,3jqb,2ilp,4azi,4eh4,4q1s,5fbn,2xnp,3sl0,3jzf,1w4q,4km
z,5uhi,2xpc,3own,3b28,4m5l,5enc,3q0z,3wab,1qj7,5ixf,5u4f,4qk4,6da4,4o05,3c
aj,4do4,6eux,1fh8,4g8r,3huc,4rt1,4xqb,2uzj,5fut,2wmu,4pkr,5aln,2p3d,5mtv,3
ncr,4o10,1gnn,3sdi,5yfz,5o22,3b78,5xsr,5drq,2f70,5mfr,4a4q,2zif,2hob,4zls,
4nbk,2vgo,6fdp,5qby,4y63,5l3j,4zy0,1tmb,2w9h,4pp7,4jnm,6gip,5tpg,4ht6,4n6y
,4nja,3pjc,3du8,3v04,5he0,4puj,3bh3,3vp1,5drt,4mbf,2wgi,4jbo,5jdc,2vgc,5aj
z,6c4d,5znr,6h29,6af9,2oz6,3w2p,2j34,5ey0,3s3v,4k3p,5l26,3o9v,3lnk,3wtn,5t
wg,6fu4,5fsm,1gz9,4z1n,4myd,4fvq,1ya4,3vqu,4ljh,4zbf,3wk8,5moc,5unp,3b3w,4
aia,1yys,5o9p,2w73,3os3,5nwh,1w12,3r4m,6eir,1if8,4ahs,4fyo,5adr,5mes,2hyy,
1ulg,2eh8,5mge,5eld,3a1c,5aae,3nm6,4afg,1q63,2ha7,3p9t,1y2f,4hyh,4yzc,4mwr
,4zh2,5i8p,4y7r,3rup,4eej,3ipu,4hcv,3uyr,4tyb,1xka,4ju7,3vbg,4zb8,3ips,3lp
0,3f8c,6gl3,3bgs,6afi,3g4k,5kr8,4eym,3gx0,2xup,3wkc,1zog,4k5p,3tu1,3mam,3p
z2,1no6,5lu2,1pgp,3dgn,2brn,4phu,4m13,4c1u,4ztl,3g45,1q84,1w8l,4jpc,3ffu,5
f1j,2yc3,3dz5,4j03,3uij,4ua8,3jzg,5tex,4cu8,3cbs,4pkt,1qb6,4od9,1h0a,3g19,
2w6c,3l4x,5myo,4cc6,6g92,6c7b,1igj,1f0r,1qwe,4wno,5aei,4cnh,2xkc,3lxo,4oyo
,3ddf,1lxf,5olk,4p45,5fjx,4bak,6ap7,1oxq,5dh5,2oc2,3ugc,1ztq,4qsh,1i9o,5jz
9,2y1d,2z7r,2p16,2l6j,5g1a,2cn0,4lgh,4yne,2qky,2yiu,3co9,5sz7,3bu1,3p5l,2y
it,5kgx,4bbe,5zwf,3v66,4wlb,5mgl,3ioc,4mx9,3rjc,5abe,1vkj,5evb,3qt7,5q0q,3

ij0,1g52,3bum,1wbo,4eh7,4jfd,4wj7,6cgt,2vtt,3v3l,3eou,4ww6,6emu,3nk8,3q7q,
6gnr,1cj1,3d5m,3ipe,6fgl,16pk,3d7b,5f0f,4hdf,3que,1n4h,3k15,3dgo,4pjt,6b0y
,1bqn,2rol,2y8i,5jgd,4g55,3qtv,6au9,1c85,5ef8,5upe,5oae,6c0s,1o2v,2w1c,5t1
u,2hb3,4odq,5ow1,2dwx,4u6w,3qio,3cgo,1me4,2wo9,5wle,3kah,2jds,1pzj,4uwk,1f
o2,3kmx,4ue1,3zvy,4f14,4gk3,4muv,1pxi,6ee3,4yk6,1rq2,4tk5,2oxx,4qiz,2r0u,5
q1f,4gvu,2mps,4fod,3m2w,5ts0,4f7j,4n8e,4ikt,4h3b,3wzk,2mow,1j16,2xne,4b3b,
4euc,6fam,1ros,5aad,1z9y,4rrs,2l12,2ow7,1hih,5drs,5i2r,1nja,1o46,5w0e,2w3i
,4z83,4mk7,5eqe,3odk,5nqr,5dxb,5mlj,1jq9,3gpj,3tfn,5f5i,3k54,1l7x,5aku,3sh
1,3zw3,2pj3,4q09,3afk,3tf6,1dar,4xm7,3ovn,1g4k,2rd6,5d1s,3gk1,4ngr,3fxw,5e
tm,2a4l,1ta2,3bgc,2rkg,5nwd,1pf7,1jm4,2fix,5y9l,1l6m,1d7i,4i0t,4dvf,2r5b,1
u9v,3v5g,4jq7,1veb,6guf,1p6e,5t23,3g2y,6dry,5i8b,1l2s,1nnu,5bqg,1v0p,5ih5,
5k4l,5m39,5alw,5hdv,5jsg,5u28,3k41,5bvn,6equ,4c1f,5wkm,3t2w,1nhz,6g35,2qwd
,2qki,5d45,2g6p,4ozn,3wkd,4ohk,5aqh,6dh3,5fh6,6c7j,3ewj,2qlq,1gvw,4zqt,4en
x,5o9h,3iod,1j07,5y1y,3cwk,3dxj,13gs,7gpb,4m5g,5tzx,5kh3,5h1v,2w8f,5v8h,3r
dv,2qpq,1tkc,3o9a,2ha5,5ak5,1cny,4otf,3tiz,4gpl,3mpt,4ds1,4whh,4o36,5vcx,5
za2,1njb,4u91,3l1n,3h8b,3rj7,4qoc,6c7d,4n7y,2gph,2uvm,1gwr,1o5p,2aqu,4q90,
5j7f,1aj6,2z6w,5nr8,1fzo,4llk,4xrq,3m5e,5ocj,1pyw,4mcv,6apt,2izl,3n2u,4i7b
,5mt4,2ydm,6fyk,5a2i,3kjn,4bcg,3ghe,1hp0,5gs4,1fl3,4p0a,2onz,1p19,2z7g,3h2
n,6h1i,3iu8,5j79,5dq8,1zea,2hwh,1kdw,5w5v,5kpl,1g36,2yac,3e4a,4k2y,2wtd,5w
g9,1aze,4i0z,3i6z,5nrf,4w4s,6gmx,3ce3,4bvb,6dlj,4mti,5yjm,3sd5,2jb5,4lv2,2
w6q,6aff,4o6w,1i9l,1azx,2yjb,6ckx,1rt2,2rjs,6cmj,3up7,4i5h,1bmq,3wzn,5dif,
4pxf,4btw,2o7n,5mw3,4abd,2c3k,5vio,4eg5,5uzk,4kfp,5kyj,3c8e,3o9e,1qb9,4ewo
,6htt,5mnr,5ake,2brm,4j2c,6ma4,2fzg,6ckw,5gx7,4pn1,1p5e,5yyf,2xhm,2ogz,1b9
v,1cim,4bdg,2r6n,5m4f,5oxk,2xdm,4im0,5q1d,2hwp,6h1h,3irx,5fwj,2vvs,5v8o,2w
ei,3drp,5c6p,4qp8,5t2y,1wva,4iq6,3t6r,2mc1,5vc6,2o4p,6cb5,5amg,5fue,3sfc,3
t83,2bvd,1jif,4ncm,4bcm,4i9s,4mzl,4z0k,4o4r,5f3t,5f3i,2bmg,5hbn,3vbv,4l0v,
2e94,6dgt,5m28,4b32,6cis,5ap2,5aa8,4fqo,5xjm,3ioe,4l7f,1h9z,4xas,4nni,5npf
,3f6e,4y29,2yg2,1jys,2d1n,5a0b,2r9m,6giu,4ebw,3pp1,5duc,4o1l,3rth,1g9r,3tw
d,4a4x,1xxh,3hfb,1ikt,4zb6,2qg0,1xmy,4dcd,6es0,3s75,4m3g,4ttv,4htp,4wwo,4l
bu,4jp9,4b4n,5hcy,4wkv,3khj,6b7e,3oim,1qaq,5xiw,1d3q,5dfp,3sfg,3vf7,5w1v,6
g8n,1mqg,5oh7,5aqk,1v0k,2vj1,5h8e,3nnx,3zyf,5a8y,2yk1,4ngn,1g7g,5d3s,4iu0,
5wou,1g2l,5ulp,5al3,1yq7,6bgu,2c90,2wzs,4ezl,3ztd,3adv,5ai8,4c94,3ozr,2aoi
,2vip,6fi5,4qsu,2oxy,4r4t,2oic,1bhf,1dhj,1m7q,3rl3,2e2b,4c1c,2wxf,2uw7,5sz
3,2k0x,2h96,6gzm,4rrf,5j4y,3iqi,1gx0,4lk6,1oyq,4kni,4a4f,2l1b,4py1,5aiv,5z
99,3e3c,3h8c,4i1c,2pri,6fyi,5fos,5oh1,1ing,3fwv,3ijh,5csw,3vp3,6m8y,1tg5,2
vc9,2x7t,3f5p,1ikv,3py1,2uzd,1o37,4qpl,4eh2,4en4,5dh3,1p57,6g22,3il5,1q8w,
1gu3,2oc9,3kek,2h5j,4qll,3s3m,4b2i,3nnu,1sc8,4ybt,3peq,5qil,4npv,4rfd,3zhx
,5lmk,1ai5,1zfp,1zoh,2pjt,6c7q,3ip9,4uyh,5mos,5dyw,6fv4,3oad,5j3s,3u4r,5ic
p,5o1e,4msn,5f94,3cic,3vye,6hkz,3s7a,3f5k,5a8x,4pvv,4mcb,6guh,3k26,3dd8,1f
d7,5nea,1kkq,4lyw,4pvt,3hkw,4rce,3w2t,1gym,5ak2,3sl5,4u4x,5tpc,2x6f,1m7i,1
u9w,4b77,3mg8,2qmd,1w4l,1w2x,3l54,1k1m,5em6,2yhd,5vdr,5bsk,1d5r,2y8l,2lcs,
6gqm,6fkz,4j84,5gid,2qi1,2x6i,1c7e,4jrg,6cjh,4dff,5fus,4al4,3ig6,2i3i,2z78
,5t5g,3f18,4edy,5eiw,1g48,2xn5,6czu,4bea,6el5,3da9,4cku,4yv8,3qk0,1o32,5us
y,5gx6,3zrl,5o1l,5ewz,5ahw,6gon,5nib,5zo9,5u13,2fgi,1r5v,5vo1,2wyf,4rxh,5c
83,2iiv,2xag,6ccq,5xih,4gjd,5akk,1ka7,5oqu,4u7v,1d6s,4njd,2j4g,2fci,3l4u,5
nhy,2w47,1cea,4b84,5c1x,2i4d,5oa2,3ug2,1z3v,2pqj,2ybp,3nfk,1sbr,4nvq,3ktr,
2xas,6f0y,2nxd,3lpu,2g01,2aq9,4er1,2qhc,5x73,3ctr,6gfx,3e85,4lys,5uxm,3p8h
,2bmk,3mbl,1lpk,3lvp,4ui7,1w1v,3tjm,2bqv,3n35,6fx1,1n0s,3qyy,2xhr,2ww2,5vs
c,1maw,5fnj,5iwg,3vf8,3kac,4ewh,3ijg,4kxm,3sqq,3cf9,4nxu,4q8y,6c5q,3koo,4j
6i,1f8e,5ioy,3eoc,5okt,2i3h,4z2o,4bic,4loj,6f8u,5vqq,3qc4,5wev,5brz,5mgj,4
cby,3lmp,5etq,2eg8,4z84,3qox,2woa,3sv2,5xhr,3ao5,5ea5,1tkx,5ff6,2ph6,1nli,
5q15,5mym,4qg7,3f2a,1ekb,4acc,5ci7,2pj4,3usn,4pzx,2vjx,1z3j,4d2s,6biv,6g9n
,4aa4,1rwq,6ma2,2x9f,4yk5,3n3j,2hah,5q17,3q77,3zm6,1mx1,6ghp,4qlk,3efs,1gn
o,3upv,6ce8,1xoq,5a3w,3jq9,5nmf,4qjp,5ai5,5lsy,5f5b,2won,5yhl,5v7i,1bnn,3q

s8,4k4e,3znr,3zkf,4f1q,4k6z,5dxh,3wzj,4xir,4ps5,1dbb,2w7y,4orx,4b3d,3ekn,1
n95,6at0,2ohr,4n70,4qwi,2zdz,1lhd,1pop,5orx,3chr,4x7h,6dk1,3dp1,5lh4,2gnl,
4ju4,6g7f,2gga,5j1w,4q1y,1l0a,4mvx,3hfj,2x5o,3skk,5th7,4msk,4ehm,1rnm,5kr1
,6fzu,3ns7,2o8h,6bu0,3h06,3o9h,6cus,4hz5,5nvw,1f2o,5izk,3fqk,1w25,4att,1cg
l,6apz,6eks,4hy9,5c3p,4k42,6bfx,4b5d,5vd3,3sni,5alj,3x00,2y80,1w6y,5iuh,6b
2c,2i0y,1o4q,3met,3tib,3nzs,1noi,2p4i,5ezh,4xtt,5i8c,4hlf,1oxg,4l58,3a2c,5
l15,1a7c,5ux4,2mnz,4u70,4a51,5flo,2qo1,3t3e,5jm4,6cks,4pgc,4zro,5ny6,2y1n,
5cj6,4yee,4auj,5y20,3wax,5k1v,5aok,2k2g,2l3r,6cqt,5ll5,3aox,4u0c,4ufi,5twx
,5hvy,3lxs,3fhe,4y8y,2jdm,2y7x,6esj,5fdc,3h89,3eg6,4b6o,1e4h,3vx3,4meo,4pn
l,3u8l,2bub,3nam,1ule,6d50,4tw8,3zpu,5ops,2rl5,3uik,4cxy,3r7q,4prh,5nk7,3v
6r,4kp5,4gmc,4avj,4p02,4cwq,4iku,2w2i,5lz7,3upy,2vcj,2zp0,1oz0,1x8d,4dtt,4
uwl,3vsw,2g1q,2h13,5lz2,1bgo,4wmx,5u6k,3uh2,3fui,5jin,2ity,4m5n,5k0b,4his,
5alf,5eko,3e6k,3fn0,5cu4,3g72,2aia,3mag,1rhq,3i7i,5l2s,6f6i,3kdt,5khg,3vje
,4abu,4gvc,1nj5,3hab,4c36,4cpu,5lkr,1akq,4jht,3lle,4mzh,1kvo,6bg5,4pox,5nx
p,5enb,3k5u,2l6e,2h5d,5fsn,5km3,4pvx,5il1,5we9,6g3y,2yol,3bux,3rin,1mhw,2f
dp,3n2p,4ms0,2hzi,3c2u,3feg,1dmb,4qj0,2wtc,2vw1,3pjg,1o0m,4k6w,2b1r,5olb,5
y94,6d6t,3gcu,4ebv,1xuc,1xm6,1ydd,4asy,4g0k,3fty,2obj,5kgn,3rq7,5mky,2xda,
5usz,4h2o,1ozv,1lcp,5u7o,3avg,5aly,6bfa,5da3,3sn8,2tpi,5eou,3cho,1phw,1a5h
,2uw0,5os2,5dlx,4mre,3aid,6c2x,2w17,3cd5,4jlh,3bhx,6cd4,2xz5,4zg7,1q3w,4n4
t,5mgx,6fac,3vs3,6c6o,4nj3,1uwh,4pci,5g11,2r2w,5jss,4knb,4ezr,5duf,4i71,1a
f6,1vru,5k76,2ckm,5t27,2j62,5lso,3qcx,3t70,3rtn,1v2m,5qar,3dng,2ito,4yab,2
c4g,4crf,2nyr,4eqj,5l2i,1awi,4ehv,6dj2,1w3k,2ff1,3bho,1bzj,5c4s,4kby,5od5,
3way,4knn,5q14,5hls,4uvv,3kqd,4ymq,5j71,5u7j,4zs3,2n06,3gdt,2w0j,4ci1,4at4
,1y2h,4hfp,1g3b,3pp7,3gy7,4qws,6f3f,2xah,3vbt,5l9i,3dc2,3d52,3soq,3d62,3ox
z,4eop,3exf,5ajo,3oof,5i3v,4zs2,2waj,4nyi,2brp,3g0i,1zhk,3hvi,3u9n,2y5l,3n
w7,4at5,3aho,4gqr,5vpm,5aoi,3zm5,1ld7,4tpp,5e7n,5n70,4zw7,4xj0,3mdz,4to8,5
foo,1owe,6g4n,5lss,4c0r,2h6t,2am2,4r95,3u7s,5abf,3gwt,5ejv,2yq6,2jiu,2a0c,
4b4q,2qtb,1c1v,2f4b,2byi,4n3r,2fxr,1odj,2qd9,2yex,3ur9,3oxc,3opm,2xye,1yhm
,4op2,1y6a,2csm,5q0t,5em7,2qbu,1zs0,5jah,5qac,2jkh,5oq6,1hmr,4x24,3kqo,4y6
2,1avn,1oiq,1txr,5hjc,1hiy,5icy,2ph8,4q4o,1a7x,5nhl,5c4l,6h5w,3zm4,2no3,2j
kt,5xwr,2aod,3pj8,1j7z,4cmo,3cbp,5lyy,3ppk,5bml,3qzq,1sdt,6eeh,6c99,1nms,5
een,5f63,4arb,2wc3,3g0c,5c0m,2qlb,3uxd,4j81,6bbs,4m14,3ft5,3f5l,3lc3,1i5r,
2ihj,2pwc,5o1f,3ccn,3f38,6bbv,1tnh,2isv,1pmn,1gj4,4lnp,3vhe,3rz3,4fjz,6ced
,6c7g,4u7t,2psv,1r0p,1gai,1ocn,4tw6,5n93,2igw,4c70,4h4b,3lxe,5t8o,4q1x,4j5
1,4jsc,2ew6,2mpa,4cjr,1w51,3h9o,1aq7,3v5q,2p7z,3k84,3pn1,5oot,2xp3,5ier,3p
76,4h71,4au8,4j4o,5h14,4lm5,2xa4,3mb6,1ax2,3zzf,1pzi,4uib,6gub,6b5r,5klz,2
c2l,4lm1,3m37,4bfz,4b5w,1o4f,3d9o,2x7d,4hy0,2r5p,6cdm,2hxm,1hpo,5ldo,4ay6,
3gi4,5d3x,4m8y,4m8t,2v22,2qtu,3t8s,5ne1,2xhx,4phw,2euf,3ewh,5doh,5i24,4rrr
,1v0l,3r0i,5gmv,4wet,1ofz,4pd7,2wvz,5y59,4hw7,2qt9,5ka3,4jvb,4fnz,4qer,1w7
g,3twu,2n0u,4umn,2pvk,2xib,1vcj,4uau,4zuo,3qcl,5g61,4rro,2i1m,4ase,3zvv,3q
ai,1a07,4ekg,3vbw,4nkt,5qaj,1bn3,3qj0,3fh8,3l8s,3tsd,2ot1,2r3l,3zvt,3uii,5
h9s,1bjv,4owv,4cps,3t3y,4nrl,5zms,4at3,5jq7,4h3i,6d56,4exs,3nzk,3i6c,6bee,
2xkd,4wz8,2ha2,4j46,2jnw,4g0a,1awf,5nmg,6dxl,3di6,5c7f,2q3z,4hs8,2q9n,3uvn
,1pum,5lvq,4mm4,1hnn,4tk4,1qr3,5dgu,1apw,3a73,4tz2,6ap6,4ijq,2hiz,2bq6,4sg
a,3ap7,3nee,5fnr,3st6,3vvy,4bbg,6fil,2i4u,5upj,4e67,4bqw,1mrs,2oc1,4y2t,2g
ej,1h1q,1thl,3eyl,5db3,4few,1uyg,4fbe,5o4t,4l4v,3s7f,4mc9,4fcr,3ryx,2iok,4
e5h,6upj,2qrl,2x4z,5ev8,4oth,3qsd,4i67,3nwe,4d08,5isz,4pr5,2vd1,4pv5,3n5j,
3fei,4eqc,3fuh,4ql8,1b57,4xjr,4kmd,4zxx,4jlj,6afg,5t1w,3wuu,3cii,3f7u,5ml5
,3tkh,5jat,4fs3,6elo,4ixu,4zec,4kii,5ap5,1qjb,2i40,6h7b,6h0b,1b11,3meu,5wa
9,4f3k,4y3y,1j1a,3g9e,1hvl,1fo3,4oaz,1zyj,4r18,4ge7,1h1p,6fa2,4jyt,4wh7,3m
rt,2vqj,4j0p,2xno,3r4p,4hbv,2xdk,5ko1,4hdp,4bup,4kfq,5tdr,1h2u,2n0w,3r16,5
t1k,5izm,3mkn,1ml1,3mp1,1s19,2x0y,6b3v,4ce3,3prz,3sio,5ti2,6css,1zxv,4xkx,
5hlw,1fzm,4e5f,4eki,4btk,4z0u,2qi7,4ban,4jgv,4hxj,6hd4,2bxt,5bnj,4mr5,3rsv
,2ynr,1ujj,2pv3,1r2b,5m17,1y2b,1qxz,3qg6,6h7z,5tca,2gnj,5jsq,3lq5,5am7,2gz

l,4y38,2q38,4ts1,3ro4,4ge4,4j79,4kw6,4hpy,4kio,5otz,4pv7,2wji,5q0l,3g70,1v
wl,2iws,5i1q,4awi,4n00,4men,6bj3,3t07,5mw4,830c,3o9c,3ind,2cgf,2zft,5oul,3
w55,5n55,2fkf,2g8r,4qnu,4fiv,2ltz,6h33,4w7t,2xgs,1vj9,1v48,6afa,4pgb,5tci,
2h44,4ujb,5ap7,2yc0,3bra,4qwf,4rn0,4ck3,3imc,6eda,1a69,5e1m,3bm8,2liq,3hs4
,1bra,1oai,1tq4,5yhg,8lpr,5mjn,5ktx,1bcj,5lif,4b83,1yms,2xk8,3kmy,4pnc,2yd
e,5urm,3c89,4ock,2j7f,4b74,5ggo,4j59,1yt9,1gj6,3isw,4l7l,4zsj,2xe4,3r2y,1b
q3,6axb,1w31,3axm,4jzb,5w6u,4fvr,3pxy,3m59,5nwe,2a3c,2yjq,6ds0,4g2y,3pkn,6
dh5,4gby,2eum,3v0p,4hxz,6aaj,1tmm,4k63,1gj5,2ov4,4kc2,5amn,5ceq,5a4l,4pnu,
3wf7,6dq8,5dpa,1al7,2j7d,3ex2,3oyp,3r7b,3k39,4nzb,5wbq,2y7p,4ddl,2xwd,4gfd
,5wuk,3ps1,5v42,5lzj,5nzn,3g5y,5diu,4n1b,4x13,3su2,1puq,4c6u,3wvm,3av9,5e0
h,5yg3,4eu0,2isw,4ruz,2whp,3v49,5wfm,1uxb,2imb,2xjg,1xjd,3eqb,3dzt,5yof,5n
vf,4cxx,4ayq,1c5f,6ftz,1n94,3uwk,3jyr,3iet,1nu8,6ckr,4og8,2pqb,3jsi,4apr,1
hpx,1z6q,3s76,4o6e,2fxv,5egs,5n9t,5wi0,4rxe,1v2k,5op8,4bzo,1ody,5q13,3egk,
4mrh,3f17,5e8f,3wz7,5a82,2gj4,1if7,2k0g,3psb,4rn6,1cps,3qel,1jgl,6b5i,2br8
,5tri,3jzo,4i8w,4mmf,2vo4,5t66,2y2k,1wm1,5ief,3d32,4d1y,3shv,4e0w,2nd0,4mb
l,4i8n,1tni,4gzx,1nl9,1w3j,4o1d,2xx5,3sl1,5hv0,5ttf,1ih0,5l2y,2ja3,4zam,5a
eh,1ukt,3kqy,4rww,4l1u,4hyi,1amw,3jwr,3cqw,4bdb,4j0a,1ms6,5osy,3vzd,4i54,5
nlk,3bmo,6eww,4utv,6f3i,4c66,4flp,4mnv,2jbl,2qcg,6g6w,4dds,4kiq,1qfs,2b53,
4jin,2y7z,2ea4,1gjd,4r06,1a5g,1qk4,4gs8,2ra0,3tv4,4lwv,5w6e,6fyz,4fcd,2g0h
,2b9a,2ce9,6ezi,3p9j,6g4y,5tqg,5ct2,5fky,4qgd,2v77,4heu,2jdp,2qtr,5zh2,4l6
t,3tay,1o43,1lzo,5y0x,2bvs,1a4w,5fa7,3we4,5nhv,4mc1,4f6u,3ce0,5knv,5ugh,5x
mv,5m5d,3tti,3u6i,2jbo,1ke7,1m0n,4crb,5auy,3l0n,3slz,3r6t,6gw4,1utj,3jvk,3
wb4,5nba,4qlu,1q1y,2zva,4yrr,3d2e,6faf,6ayi,1uvt,5ox6,4pnt,5uk8,3s7m,6gch,
1ym1,3emg,5swf,6apr,3tg5,3qx8,4o9v,3rde,4ngs,1owd,3fhb,3oli,3wq5,3pix,5c26
,6fex,4cd8,3vw7,6g14,3snc,4dhs,4ks5,3lp7,1m21,5cqt,4ay5,3sie,1c8l,3ow6,4z6
i,3re4,4l7j,6cq0,4jvj,3zsy,5ai1,4der,3hqh,4qtl,1vsn,5u00,4ipn,3lq4,4lg6,5x
13,3qo2,5cs6,5q0v,4xbb,6b7b,4zzy,3hv4,6cpa,1d4w,5ura,3zzh,4xkb,2nno,3ch9,5
ukj,2cne,2qbx,6bil,4i1r,5f02,2xni,1ppl,4ksp,5emb,1mem,2cgv,2xxy,1erb,1qan,
1tze,5hld,2qi4,3ck7,3lce,5eq1,3kdb,4uyd,2of4,6cwf,3ma3,1nvr,1nfy,3ztc,4eok
,6g15,2q7m,5eqy,3po1,4yrd,1d3d,3wf9,1abt,5mi6,4bam,5diq,2jxr,3jzr,6b4w,3o8
p,2we3,3zke,2clm,1lox,2uw4,5ah2,6ax1,5aqg,5jan,1z4u,2a2g,3zze,6f6n,2g63,1w
80,4zsq,2aez,5b25,6dnp,4gw5,4qge,2uxz,6fi1,1re8,5ekg,4fs4,4e3f,4rme,1p17,1
zsr,1atr,6dvl,5ie1,3mof,4mds,4kva,5vqy,4tpm,4wj5,5hki,3wtm,1xoz,4kvm,4xmb,
5k13,2qp8,6gfy,5abg,5xxf,3tfp,2qw1,5kjn,6hm4,5hlm,4ff8,6fqu,3ff3,6d59,4y5i
,4jjf,5nhz,4abv,5z9e,4cp5,5mxr,5waf,3rti,5exw,6b5m,5uvc,5hu9,1amk,4o76,1b2
h,2hfp,6g39,1hsh,3zlq,4ys7,1c5t,2p2h,2cbz,4k5l,3t3v,1jeu,4qp9,5li3,3fdn,5c
eo,6eo9,4mc6,4yef,3bwj,4b1c,5kns,2uzl,2qdt,2mas,5w6i,3d28,5wgd,4jfk,3w2s,1
klu,3poa,3dt1,3uw5,5hf1,4fys,4x8o,5th4,2fle,3px9,1qs4,5j6n,3nzc,3efr,5n9l,
3be9,4msa,6g84,6ccy,5aes,5ld8,4h2j,5ivj,2w71,2qhm,5ufc,2hmh,4xuz,3bg8,5ugd
,1xqc,3njq,4eke,1y57,4mjo,3d6o,4egh,3le8,5ihc,5t9u,2g83,4ddy,4gja,2q6h,3zq
i,3vfq,3l3a,3osi,5ant,4gto,1lbk,3f3t,4ui4,2r75,2fky,5kos,5qin,1ugw,4dxg,2o
9r,2lpr,4jzr,4gtv,6cec,5j59,2g9x,1hkj,3owl,2n9x,1alw,1ysi,3avj,2fov,5efj,5
obr,4pin,4q46,3upx,4ufl,3b26,6gjr,4tsz,4puk,2vpe,2vmf,6gop,4lxz,4bck,2kgi,
6f5w,2cfg,5a81,1o3c,6h3k,2kwn,4tx6,3unz,3s8x,4tkb,2pou,4e26,5gvn,2x6k,5ka9
,4rra,6ay3,6ey8,1ql9,5vfn,6bfw,1akw,4kql,3i7e,3omc,3eov,2wu6,2gmx,1df8,5ef
a,3v2x,5j47,2i0d,4gbz,4j1h,3shb,5cqx,5lqq,1uvu,6dkb,5wxo,3t0b,6bhd,4cpz,5i
kb,2r05,5e8z,5cbr,6biz,1y3y,5izf,4yjn,4kz0,2p9a,5yf1,2pcu,1oxr,5l0h,6bw8,4
xmr,2c1a,3pa5,6eea,6d8e,3ds4,6ekq,1qwf,5zk8,2wey,5n9n,4pre,1g37,4d8e,2duv,
3dk1,6c8x,4jfj,5u14,2cvd,2gvz,4q3t,3g90,2c1b,5iu6,4hva,6fc6,5t2d,5iv4,2vxj
,5lvr,5auv,2qzk,2w8g,4db7,4alu,4iu4,4cqg,2qlf,6bm6,6e2n,4kp4,4qn9,1c1u,3ct
h,5v19,4ryl,6lpr,4jk6,4wvu,5g1n,4ark,5wfw,2las,1tvr,4l2k,2wa3,5k05,3unn,4y
ll,2xuc,3iuc,2y4s,2xpk,5bv3,3wzu,4cpy,6hlz,4bty,2xzg,3eig,4cfv,4qta,1hk1,1
dmt,4kb9,2qci,4l8m,5fcz,3ui2,2ohu,4pl0,3ejt,4wn5,4y5s,1j01,4y4g,3rwd,4d8i,
5kkr,1ihy,5luu,1w76,1nkm,6cn5,4lh6,4oc0,4aji,4c6x,8hvp,4b1j,5gu4,1y3v,3zt3

,1rv1,4b7p,4qkx,1gj8,3l5c,1aht,3fl5,2cc7,4ie7,3p8e,5l4f,6b1x,4i32,3le6,4mr
a,3daz,2oj9,1a52,3v4x,3jzk,4v24,3evd,4ra1,3qch,3tv5,5sz4,5o83,4o4k,4oiv,4n
p3,1cqp,3zj8,3i7g,5ukm,3fzc,5ai6,3vws,1rdi,4mhy,5ais,2bdy,3kab,1wug,5e28,1
kwr,4mm8,4mlx,2gss,5gg4,2uzn,4u71,2ybt,4imq,2y67,4b85,3drr,1qvt,5t92,4oma,
3tvc,4g8y,6eol,4imz,3mct,1di9,4wov,6fu5,4lmn,5eok,3qdd,5vfj,1jp5,2wq5,3pcg
,1ggn,4jyb,3vha,3gep,5f04,5lh8,5u9d,2z9g,5mb1,2xv1,1bji,1h35,3kqc,5ukl,3db
d,3tt4,1n5z,5wbm,6eif,5fov,1iih,4or6,2vd4,5agi,3obq,2ad5,4z8a,1vja,1c50,1r
sd,5kyk,1dqx,3bzf,5km2,5izc,5k0x,3ral,3wka,5d7r,4afe,5yba,5g4m,4exg,6ez6,5
kh7,5l9l,4owo,1gbq,3h22,2xm1,3ed0,4m8h,3ryz,4gj9,5cf6,4mz4,1qft,4gdy,3gm0,
4btt,1c12,6exm,5d6y,6bed,2xto,1nl6,4iax,5vp0,2q7y,6ep4,4z46,2g9v,4efg,1xp9
,5mk9,4el5,1gag,2xiy,2o4n,4mmm,4bi2,3b95,1ele,3aqa,5lpm,4ou3,2og8,4f3i,5jy
3,5web,2x85,4he9,2xuz,6f7t,5iyv,2llq,5ta6,1eei,5ltn,1z3t,2viq,1mxo,1g45,5h
g7,4wq2,5ung,3ccb,1kv2,1hn4,1e37,1tr7,6chl,5m56,3kdd,5f41,3pvu,2w4i,4wko,3
vv6,4zum,5k1d,2bfr,4mx5,5jur,5ut4,3u3f,5t35,2ga2,4hld,3s2v,3e01,2tsr,6m9d,
4cwb,1e8h,5j8i,5qas,7hvp,6b4d,2euk,2bmz,4unn,5t6f,5vcz,5vsf,2v86,4tln,4drm
,2ez5,2zfs,1d4l,5cas,2fs8,6g5j,1rr6,2oc4,2nn1,3tjc,4k55,6bw2,3v0l,5yqn,3kp
w,4qz7,4mw6,5ye9,1d2s,5hct,4efk,3dri,1m0o,3q2m,4bb4,4kne,3og7,3nox,3lco,1d
4k,1dtt,1ryf,2ltx,3jzs,4hw2,5ew0,1nde,2afw,3dpf,1ro6,5e3g,1hlk,4usw,3l16,3
tjd,4g93,1nyx,5eol,3li2,5fb7,3tdh,4b11,3gus,3biz,1bmb,1v1m,5wp5,2vf6,1c8k,
4aj2,6axk,4rcp,2hm1,1q6k,4ezt,2hmb,3f80,2c5n,2vcg,3gl6,5jdi,2k31,5mgk,4f9u
,2x8e,5iok,1wc6,2jsd,1w11,1irs,1rm8,3ezr,3i7c,2ykc,4z2p,3iqu,2n14,5ifu,5dr
o,2q15,1hos,2evm,3e63,5f2s,1ong,4x7i,2xej,4kn2,1h08,3q6z,3aav,3dz4,4q4s,4a
w8,5xg4,3g2k,4pqn,2jdy,1o2q,3gwx,4y4v,3n7o,3rk7,4ezj,4ajl,3c72,4qvl,1ebz,4
xsy,5cp9,1iig,6do5,5iw0,5npd,2ai8,4cfw,1tka,4zud,4e3i,4lkg,2o65,4l3p,4b4g,
1cpi,3zsz,1c3e,5a5s,3pn3,4igt,5vdq,1dub,2jgs,5anv,2fr8,3kdc,3ob1,4uin,4x9r
,3zls,2vtj,2i72,3mv5,2d3z,4a4h,1f0s,3uvk,4kbk,1i33,2fwz,2ajl,3rmf,3fsj,4gj
3,3d2r,2cgr,3dcv,6h7l,4e3k,2y8c,5qb1,5etv,3odu,3h85,4wki,6b30,1jd0,4hkk,5d
hs,7upj,3wi2,1me7,5d7c,1f4e,5er4,5nxy,1owk,4yha,2vj8,2pgz,4xxs,1b39,3lfs,2
pr9,3t2c,3g4g,1jq8,1jii,4gvd,6f8w,5mw2,4yhm,4zl4,3th8,3mt8,4v1c,5km9,4und,
3ip8,2d0k,3ds6,3vp4,3d9m,5djv,3uu1,5c4o,1lke,1z71,4qp1,2bpm,1siv,1bt6,3tkz
,4eox,1xoe,2ohq,1kv1,1zd5,2qg2,2i4x,1rtf,1o39,4rqk,5q1h,1rev,5gp7,4elh,5di
t,6glb,4aof,3et7,6g9h,5im3,2cia,5lhi,4av4,3kr5,4xqa,2yga,2a5s,4dhp,2pzi,3r
1v,4lmu,4kwp,6ba7,5o58,4azc,5xkm,6g9k,2viv,6gzh,5xvk,1zh7,6bh2,3q4c,4x1r,2
xm9,3da6,1zz2,2a25,3wha,4bdd,5igm,6awn,3gy3,3qs4,6ewe,4m3p,6czb,2c94,4zei,
6fe1,5tt3,3khv,5cfb,2xmy,2za0,1u6q,5l2t,5ofv,2ei6,4hiq,2cbr,4wym,1i7z,5ep7
,5j4v,5ech,4j1f,5t1i,3dbs,2yxj,1pa9,6e99,5t90,2iu0,5xqx,3lzv,6dq4,5mql,5et
k,3ms4,6afd,4au7,3lp1,3rjm,5jga,4gfn,3w2q,1s4d,4ayr,3hv5,6bgx,4ayt,5bs4,3r
v4,2hai,2uyn,4zsh,2co0,4xtp,5a3n,1xa5,5m4k,4rsc,5m4u,3vf5,3moe,2y82,1ad8,5
hfb,4j58,5lg3,3sxu,2zga,2igx,2zbk,5wxg,5ndf,2wij,3fal,2p3o,1pwp,2ph9,3lil,
4uix,3q44,3sbi,4wsk,6eqp,2lnw,5lle,1z34,4h4m,5nee,3f8f,5tiu,2cnh,2wq4,1f57
,4xu1,4aqh,4g95,2c8y,3ay9,5ofi,2wig,1udu,3rw9,4myh,5amd,5dtt,1pg2,4nw5,5cs
h,2pj6,6dub,1kqb,5tuo,3l5f,2cni,1uvr,1jld,5ygd,5t97,2pmk,2b2v,4r59,3dux,5u
wn,4lkj,1fq8,4qdk,4h3q,1k4g,1bbz,5axq,4b2d,5myd,4b6e,4yhq,3lpp,2wl4,4ivs,3
h1z,4yik,2baj,3skg,6aqf,3np7,5fwr,2ivu,1jwu,2io6,6fau,4dh6,4txc,1w1p,3mle,
4de0,2r2b,4lnb,2rcu,3zhf,4yho,2b7d,4q9o,6aqo,3inh,4zyq,5nya,5t2m,4zij,4hym
,3pe2,1nm6,3el9,3f07,4msg,4og4,3ujc,2vwn,4eyj,3c4h,3eu7,2x2m,3v8s,6baw,2bb
b,4cd6,3sha,1ocq,4w7p,5za1,5xs2,4wmy,4j0s,3kc0,1q1m,5yp6,5auw,1tcx,3cib,5k
5n,4ovg,4l9i,5t68,1i91,5ll9,6bij,4otw,5tkb,2vio,1szd,5fi7,1stc,2vcx,5dgj,1
bv7,3ml5,3bl7,4bzr,2zmd,5yvx,3lqi,2fs9,5var,3bex,5cei,5t2g,5sym,5wbl,2f8g,
5km5,3ffg,3g3r,3sh0,3le9,2r43,5hn7,5q1e,2vpn,2byh,4q1w,2gpp,4dmn,4qjo,1my8
,1cnx,4k7o,2vey,3nfl,4zwx,4zyf,5er5,5zmq,4est,5fao,5c29,2q94,4whl,5tqu,3q2
g,3tpr,2n1g,1mkd,4qwr,5w1w,1jrs,5f39,1tft,1kna,4hf4,3ryw,3uok,5y7z,2v0z,3g
z9,3lq2,1gww,5t4e,2ama,6cco,2fdd,4mm7,3oik,3il6,3n3g,3bm9,3igp,3vhd,3mg7,4
ury,5l97,1o4h,4ppc,5ehg,2wgj,4m6q,1qj6,1n7i,2fwp,3fmq,2qhz,4oew,1pwu,4g5f,

4crj,5j8u,4cg9,3ueo,4y79,4gk7,3bzi,5n49,3pb3,2w6u,5hfj,5dgm,3wp1,5f1r,5bnm
,4ktu,3c45,3blr,5if6,5mqv,6bfn,5ezx,6h37,4l19,4x69,2hd6,5svx,4yym,6fo5,1j3
7,3drg,4wpn,5hbe,5q1c,3d04,2xm2,4xnv,4dpi,3udv,1n1g,4odp,5ly2,4obo,6hu1,5s
vk,2zdk,2pl0,4zki,1bnm,3wiz,1xp1,4msc,5d6q,4xiq,6anl,1f28,1mzs,4wn0,3q6w,3
cow,6dgy,5jzi,9hvp,5hmk,3zpq,1ayv,1uwb,1vjb,1y2j,5nzp,3snl,1o4m,6aak,4qt1,
5wii,3e9b,4hkn,3n75,3zn0,4ok3,4hgs,3dtc,4ks4,1g1d,2o4s,5k0s,3gjs,5izj,1vyw
,6hsk,1hdq,1xff,2lzg,4l7b,6b41,4hpi,2ow3,3eu5,5kmh,2aw1,1n1m,1yfz,4r5t,2g9
u,3olf,3fgc,2wj1,3db8,3tfv,4ehe,5ia2,7lpr,2i0e,3cdb,4ieh,5j4n,4f64,4kn4,4r
r6,1ysg,2ig0,1pus,3h21,2p8s,4g8v,4ok5,4qsw,2gv6,2qrp,3c2o,3g9l,3f1a,5u6v,2
h5e,5ut0,3okv,1uyf,6hmg,4hs6,1o2t,4ojq,4tq3,3ayd,3lcd,6das,5kre,3d0b,4gv8,
2q63,2vcb,3u6h,2i0a,5u5k,4jjg,5lgn,4i7d,3eks,5trf,4jq8,5ii2,4xv1,4no8,1iau
,4hwp,3qj9,4oya,3atw,1g7q,1m2q,3qaa,3g0g,4mk0,5flt,4g3g,3be2,1tpz,4luz,3r0
1,3nw6,1c5p,4zlz,2fum,2vl8,2f18,4rxc,3i8t,3tb6,3ii5,6mub,4hvs,5k1i,1oau,4u
nr,1bim,1lf9,5qaa,3iue,3ebl,4l7n,4kc1,2wyg,5fou,4m3b,5t8e,4loh,4yti,4nga,1
ll4,5uiu,4xjt,4okg,5ty1,1fvv,6gxw,5jzb,5jfu,5up0,1yc4,5d3p,3efw,2ndf,5qal,
4iue,3ibi,5gnk,3bys,1njs,4pnr,5u7k,4kqo,6mx8,1tl1,2itz,3fu5,3cct,3hhm,2pjl
,3kyq,4zts,5jvi,3sjt,4hai,4fgz,3ixj,4bid,1w13,6gr7,2qu6,3s77,5fox,3ati,2uw
l,6fel,1zzl,2cf8,5f2u,4cws,4xoc,3gf2,2yek,4xyf,4rfr,1c5x,2vhj,2bj4,4bfr,1p
y1,3oay,5dp8,4qwl,3e3u,4u5n,2xx2,3h5u,5xyy,5laq,3t7g,1lgt,4dhm,4oys,4j74,3
e3b,4rx8,2ooh,5hng,5w86,2bxu,4mvw,4emt,5jmp,3s22,3b9g,1hgi,3wzq,4iut,3avk,
4ngm,4dko,3g2v,3ncz,2pix,1c3r,4ly9,2qju,5q1g,5ene,5am1,4dkt,5ggj,6bsx,3c8a
,3uzc,3hnb,3qnj,5vwi,3ovz,4tpw,5dlv,3gkz,3b25,1j36,3n3l,2r3j,3krj,3ee2,4i5
c,5j19,6evp,5ukk,2fl5,5wl0,1wbs,4rd0,4kqr,3wpn,6cfd,1oky,1y2e,4b7j,4as9,5b
pp,2off,1z6f,4k69,5q0r,1n2v,3gur,5c7c,6aom,1lqe,3u7n,5mek,3uvx,4ayw,3ik3,3
nq3,5nge,4loo,4fr3,1fv0,1h1d,1n8u,3d9l,3h0q,4avw,4bpj,3iit,1nc6,2wxp,4yxd,
5hg1,5n1x,1mes,4g2j,2q9m,3d3x,5u4g,5yjp,6f8v,4qmv,1gwv,5y13,3c7n,2wcg,2vtn
,5hz6,5d21,5tzf,5l4m,5thn,3db6,5c4t,1njd,5egm,3pb8,1d4y,2xog,3kw9,5hx8,5tq
8,5pzm,5gmi,5qcn,5l8n,4dow,5ap0,6hvi,3rv3,1kmv,5h09,4k3k,6fni,3l8v,3fyj,4e
w2,3vdb,2tmn,5m5q,2f3r,6g3q,4olc,5dw2,2zu3,5a8z,3uvu,4ibf,5hoa,3dnt,2w06,4
yog,2hzn,3m6r,3ant,1f9g,1p02,2x2c,5wdc,5enh,4wyz,2xi7,5lck,3bv3,4k8s,4mxa,
1czo,4ia0,6fgg,4ymb,3q5h,1p05,4yxo,3jxw,1swn,4k7n,3muz,6cw4,5dls,6eo0,1i1e
,1hlf,3ckz,3mj2,2iwu,6b7f,1uj5,4zt2,5m77,1z1r,5aol,1hk2,1yc5,4lj3,4ipf,1e7
2,3p9l,2h02,1y2g,3otf,1nhx,2gz7,2yjc,5qay,4oks,6g9d,4bgh,4qbb,6eox,3mv0,3t
2p,4lch,4y87,3i25,4zt7,3frg,4xty,1wc1,4hbm,2cm7,2x8z,1g5s,3bki,4klv,4a4c,6
hth,5tq4,1m13,1tnj,5tx5,2lty,1xmu,4eol,5egu,4yy6,4glr,1i7m,4gxs,5c1y,2oqs,
1gvu,1o4j,3fee,4joe,6b1y,3c7p,4uja,4fai,2uyq,2q11,3k22,5alh,2lgf,4bcc,5nk9
,5mmp,1dgm,2ces,3h0z,2psj,4acg,4bah,4o04,4j1i,4qyh,4dlj,6fa3,1xh7,4luv,2ns
j,6faw,4fll,5fns,4gzt,1vjc,5f32,1ur9,2b17,4ijh,5eml,5mih,3d3p,6df2,5m0m,4n
rk,5t19,3eid,1di8,4pji,6bnh,4w9x,4mpe,5u2e,4x2l,2vba,2a4r,4qzs,5usq,3fx6,4
azf,4pd6,5a3s,5kqy,6min,6dxx,4b5b,5trs,1izh,1ke3,2qoe,1sjh,5a3h,2pvj,1dis,
4nxq,5hbh,5ekx,3oka,4d63,3uvw,4o44,5e0m,1czr,3cpb,3h0v,4ask,3c8b,2ieo,1ci7
,2oz5,4u6x,4dk7,4p0b,6drx,5y24,1izi,2nmx,6f9g,2f8i,3fea,5ur1,2vtm,4j5b,1du
d,2bro,6exj,5ksv,1u8t,5wzv,4p58,3mhc,3rwc,4ty6,2aoc,1lol,5w4r,4l32,2kfx,5n
31,5jf4,4xtx,4qhp,5n2x,5nk8,4p3h,5aa9,2jjb,3mj1,5u0y,2qyn,4bqg,5n8v,6d9x,5
ful,1i80,1e5a,4c1m,3ubx,5btv,4uru,5ku3,5w6o,1bgq,5u5l,5jao,4qvv,5inh,3iny,
5a6b,2kup,1ujk,5vdu,4u0i,5yjy,4lhm,1od8,1nw4,4c4e,3gv6,2a8h,4gfo,4gue,5j27
,3amb,2xpa,4f7l,2ql5,5c20,3t8w,1d7x,1vwf,5o7e,3cn0,4ibk,4x68,1p7m,3heg,4ba
3,5qa6,2mlm,4blb,4dmw,3p17,2pov,3s3k,4unp,4y16,4qnb,4ufj,5fnq,6afr,3qo9,6d
5e,4m8x,5kr2,3ot3,1w5y,6awp,5hka,3qiy,5i86,3nzw,4cae,6ft9,6b8y,2wuu,2c6n,3
wyl,6csr,5lch,4po7,4kxn,3fyk,3eyf,3va4,3fzn,3e7o,4btl,3h0b,5oa6,5ylj,2v58,
4yc0,4ylu,5hja,5maj,1xdg,4nrq,4ucd,1o8b,2aow,5h08,5m4q,5eei,4nwm,5dp7,3exe
,4qgh,9icd,1o9d,3dcc,2o0u,1yet,1c2t,4qkd,3of8,3d9k,5ih6,2osf,2brh,4mss,4r0
i,4kov,4qw7,1m2r,3ncq,2b8l,3lzs,5j6a,3ow4,2ewy,4j3d,5kej,4qo9,2y58,3btc,4x
v9,5hfc,4ch2,4b82,5a83,1o6i,4hxr,5tc0,5a00,1os5,3fl8,2etr,5u7d,6di1,4bds,2

qve,1xog,5izu,5uey,5v3x,2xct,3muf,3tao,4dgn,5htc,4lko,4mzs,4wkn,4zom,3fvh,
5wej,6ei4,3qi1,4afh,5lz4,6fmj,2xg3,5hmi,6hai,1bzf,3v6s,3nim,3dz2,1heg,4zga
,3doy,6mr5,4mvn,7kme,3d9n,6ea1,1uwt,5jv2,4abf,5f2r,3i81,5lc0,3gvu,4yl1,1k2
v,4cwf,4cjp,4avh,4tk3,5wqk,2c6m,4yps,1hbj,6gnm,3kqp,4u0n,1rnt,5t52,4o0x,3g
c7,5f1u,4e0x,4kom,1nny,6apu,4kwg,2wmw,3kf7,3uqp,4r6e,1w1d,2fv9,4y32,5mkz,3
c6t,2w1e,4otg,5ar7,2r0h,6beb,5u2j,5igl,1o9e,1oar,6br3,2xg5,5ism,5uch,3k5i,
1b6k,4r6w,5mja,5jy0,4uco,1zeo,3hl5,2r3h,4jnj,1r4w,2v96,3cr5,2hy0,5uwf,1nje
,3liw,5yzd,4pzw,2v2c,4eon,1t1r,2gc8,4o97,5kww,5svz,5jc1,1uwf,5xmx,3cpj,4jf
e,3s3n,5u6c,4j9a,4nrc,2rcn,4xhk,1f40,1vj6,4zed,5vp9,3mg4,3sb0,2ylq,4e96,1e
ix,1hrn,5d0c,5v40,8a3h,2c1n,6ft7,4w4x,3v7x,5v84,4mm5,2j87,2hrm,5edb,4x3r,4
q3r,6cd9,3d6p,2h2g,5jf7,3hb4,5mat,5ipj,5elw,4b6s,4q93,4y6r,3hxf,1e03,1t7f,
4wcu,2pax,5jap,5jgb,1y4z,3qck,1nki,1uod,4fcb,4xs2,1n7t,4idv,2ybk,6b3e,5hd0
,1xud,4pml,4rfc,2g0g,6q73,4o55,1lkk,3f7z,6cd8,1xuo,4ifh,2isc,1t46,4e90,1h9
l,1npz,5tyh,5nme,4qht,4jln,6h7k,2ylo,4qn7,3nuu,4jiz,2yfa,3i90,3k5k,2j4q,3v
1r,2csn,4aje,2lsv,5f29,4zx5,2j79,5oje,1uu8,5jq8,1jut,1thr,4q1n,3p7b,5wdj,2
vrj,4ai5,3mf5,2xk4,4na8,1gfy,5l9h,3gwv,4zip,4yb6,5al2,1t13,4cc2,5c3k,3nti,
4wnp,6esm,4d09,4o2f,2hs2,6bc9,5xij,4p5d,4jx9,4oeu,3h52,2pk6,2ydf,5uci,3lk1
,5hz8,1onh,6br2,6ma1,2g5u,5cuh,4j3j,3b7j,3rse,2xjx,6eee,1oh4,3msj,5a7y,2jj
k,1mui,4zyv,5qae,6e9a,2ca8,4gz3,5he3,2fts,4fcm,4r5v,5eps,1o5r,4pax,5bot,3u
6j,3el4,4jvq,2itk,2zz1,5lxb,1c7f,5wfz,5iql,5k0c,4wx7,6dvm,5j9y,5try,2f1b,2
ow2,2oym,5hj9,3v30,4amw,3nif,3sx4,5llm,4j77,2xx4,1fzj,3lxk,3lcv,4mzk,3g3d,
1h26,5w92,1xbo,3dcq,1mmr,7cpa,5m7s,3dcr,1ru1,4a9u,6dcy,5td7,5tkj,4g16,2qwg
,1dqn,5xii,5fpp,4qqc,2ien,3hdz,4hxm,4ye3,4kxl,4mf1,1ym2,6gh9,4wku,1ec0,4dp
y,4nru,6hx5,3ccw,1ols,5idp,5toz,4azg,1tsm,5fcw,2c8x,4yh3,3t0l,3kej,5e2o,3s
zm,6czc,6c2y,3oyn,4e3m,5azg,5dxg,4hbx,3piy,5otr,5ehe,5wxp,6b97,2vw2,4ofb,5
qa8,4nau,5d1n,5ajv,6arv,5lgr,4dj7,1i90,2erz,5wa4,5u51,5y8w,5euk,6gu7,2uup,
5jas,1y3a,3l9m,1oth,4itp,3olg,2fys,1pxl,2x6j,3guz,3byu,4i6b,5tuq,3n7h,2q7o
,3zll,5fd2,4ez5,3rz8,5qa4,4uwf,3dx3,2zx8,4yw2,4l7o,6ffi,6aqq,1g7v,5ewd,1k1
o,5jf6,3gp0,4cfu,3acx,5epr,6eji,1hi4,5lpr,6g8m,4dkr,5ewm,5lzh,3oui,3wv3,5m
k1,3fud,4l23,3ud8,5mng,5js3,3njy,2zm1,2y5k,4uiw,5t8p,4x8n,2xu4,5tcy,5cf5,3
hu2,4qtn,6gjl,4ezz,4j04,1uye,4ymg,1jdj,5eh5,4nue,2r3m,5gmp,1dbj,4p6c,3p7c,
1o44,5ehw,2bmv,3d9z,5g5z,2fqx,3v7c,1m83,1o45,3cft,4lyn,4dhn,5kpk,5svy,3acl
,5ivt,3s7l,2rk8,3arf,4b6p,5j9k,5nra,2vwx,5eu1,3qw8,5g1z,4qzw,4y64,4elf,3v2
o,1z2b,6mvu,5owf,2y1w,4zwz,1mn9,3oev,4jfl,2xp7,3srv,4m7j,5j7p,2ggb,4qz2,4r
e3,5ty8,2qmj,5upf,5q0u,3wto,2xwy,4i11,2q8g,5lp1,4prp,3ly2,2fr3,1xsc,1ke6,5
h17,5zwe,4jaj,1yk7,4ef4,1gi9,3dkf,3bla,1ajx,6g97,3gc4,4m5i,1g9b,3hvk,4aml,
6czv,5alr,5aqo,4n7h,4tmk,1k9r,1cbx,3r0w,3g86,4msl,2c5o,5cuu,3np9,5gvp,5q0g
,5e74,5kkt,5a7j,5umx,6b5a,4kx8,3tpu,4ido,4l2f,3k98,3rdo,5vfm,4hxl,3zqe,2xb
w,3axz,4iwz,5fl0,5orv,2wd7,2r3w,5vb7,1xn2,2x2k,1km3,6bin,3ewu,4pmp,3iut,5f
ls,3r7o,4pz8,1uy6,1gu1,5vjn,6fgq,4fcf,6std,6bh0,2vyt,1inf,2uw3,1nnb,1tl9,3
rsb,3qzt,4zt8,3t4p,4jj8,6ce6,3d94,4f7n,2ya8,3d1g,5ggz,3okh,4iuv,5di1,6hpw,
5v5y,4og6,4cgi,3zdv,3qvv,3t08,2ydt,1h6e,5k0h,3m58,2wjg,3iw5,4gg5,4nb6,5q19
,4bf1,5g57,3q43,2fv5,5a0a,3o96,5l4j,1nl4,4c73,6g1v,2r38,2wky,5ij7,5d3h,1rh
r,5e7r,4ufk,1los,4ty8,4mdr,3e0p,1g2m,2wih,2jdh,4nxr,4zpe,5etu,3ezv,4yzn,6f
3e,2pjc,2qrm,2lbm,2uzv,4uns,1c84,4qip,6bt6,5mmg,4civ,5k4j,5fe6,3g6h,2xn3,5
l8y,2avm,1mfg,4hrc,6g47,5ee8,2pwr,3oz1,4i9u,6ftn,4xe0,4gs9,6dcz,3k3i,5f9e,
3s54,2azb,4ce1,1soj,4oar,4kox,1zoe,4qpa,3d4y,6bw5,2z5o,2i4w,3v3b,3rtp,1vik
,4x0u,5k5e,5o3q,6gf9,4agm,5yc4,4djp,4eh3,4xuc,4mo4,5dht,4hlw,1j5i,1i41,4bs
5,2kp8,5h1e,5z89,4ccd,1svh,4lxm,5m1z,2c68,3rx7,3rxm,6tim,5bqi,5z4h,5etf,5n
1y,3nw5,5lxd,2wbd,4bc5,5vi6,2wi2,5e0l,4wmc,4j53,4i31,5jha,2ff2,6e0q,3h9f,5
x28,4k19,1mh5,4cbt,6h7y,4n1z,3kmm,5xyf,3ibl,4nzo,4ll3,5u4a,4zy1,2jo9,2pnc,
3p8n,4gg7,4mx1,3fmr,2aof,4xaq,1rri,3d7k,5icv,5oh3,4bjx,6bec,5h1u,2f81,3qkv
,4yax,3gxt,6chp,2qta,4l0b,4jfx,4r1e,3r91,5gsw,1gjb,1lvu,6evr,4lgu,4b8y,4xa
r,3a4p,3h6z,1mv0,2g5t,5khd,4zjc,2rkm,1g54,2y68,6gy1,4gd6,1yxd,6dqb,5ort,3p

s6,4g68,1eb2,1ow4,1jd5,1qwu,5ewh,3rhx,1me8,4zx0,5f6d,3ukr,1nw5,4rfy,5t4h,4
rlu,3t3d,4eh5,3qem,4cff,2aei,5za8,6eiz,4in9,4fc0,5d2r,4awo,3rxp,4qxs,3dm6,
3sax,2eg7,5cap,5nap,3t85,2owb,3ook,4qag,4r4i,1qji,1p28,4u6r,3g08,2wzz,1ceb
,4hu1,3r9h,4hv3,2q8y,3rm9,4dcs,6fi4,2nw4,3g2j,3h78,3igb,3qc9,5vfd,5zkb,1lq
2,1a2c,4e1z,6ei5,6gmd,2y8o,1bm6,3zs1,5bq0,6hh5,5os7,4m3m,4uit,6czi,3uqr,4k
75,3v9b,1szm,4r3s,4b81,1z9h,5aer,1q6p,3rf4,4d7b,3w8o,6eed,6gnw,5ufi,4esi,1
zom,5dx3,6nao,2gv7,5osd,5n8j,1r58,5cgc,4ca6,4igk,5ka7,5wys,3f82,4c38,3ddq,
2ypo,2xl2,3mtw,5i4v,1tsl,4z7q,5iu2,5meh,2fo4,4bis,4oi6,4o91,1i9n,4y6p,3k83
,2xj2,3i1y,5j6l,1a4q,6cnj,2p53,2va5,3rvg,5mi7,4o37,2y4a,1q72,1ogg,5prc,5fp
u,1v11,3hkn,1zgi,3ok9,3n5h,1gyx,5wg5,5e0a,1k6p,5dtr,2hoc,4n1u,2xba,5tyr,4j
px,4ga3,2aoj,5v2q,3voz,2pja,4ddm,4jve,2cmf,2gvv,3ies,6esy,3vzv,1t08,4el0,2
nww,3g30,3i5n,5aoj,4uxj,6b1e,3dp0,1aku,2qnn,1x0n,5kv8,3o9f,5dqc,1vzq,1pi4,
6eya,1mjj,3lpj,1eat,2go4,1bio,4keq,3rqw,2igy,2nmz,4tn2,4c1e,4qmq,4umt,5wkh
,4hyu,1tl3,5fdo,1nfx,5mra,2v7d,3ldp,6ev0,5j9l,4ykn,1jqd,4m3d,4l10,2fwy,5ar
0,5cao,4px6,4zpf,5nf6,2qm7,4gsy,2r6y,2yln,3u4w,2zx6,4pd8,5x27,4a4o,4x1n,4q
ij,1cbr,3mg6,2v95,4aph,4l0i,3qw5,2ewp,5cu2,3qlm,3kdm,4ris,1w5w,5ni8,3wz6,2
o48,4lge,3cda,3t64,4ago,4pp9,2r3f,3ot8,1pmu,2gcd,3fu0,4rqi,5ef7,2lh8,3rl7,
1wbt,3f16,1kwq,5n7g,6beh,1tqf,3uyt,3ao1,4erf,4z2i,4fxz,5lwm,2xaj,3ggj,6csp
,4p0w,6hti,1uwu,5qam,2y1g,3zyh,4z07,3r7n,2zmm,3sgv,5hvu,6bx6,3eq7,1o1s,5ka
m,1d1p,6bnl,6f9t,5ml2,1xon,3c6w,1a0t,5h15,6c5f,3pab,3pxz,3e51,2o7v,2upj,4n
ro,4loi,4a4v,1v0o,1lf8,2vzr,4lxa,4ibd,2gbi,4mg9,4u0s,1f3j,4gtp,5auu,3hxe,5
dy7,3zo2,2j94,3t03,5opc,4gq4,5op6,5ujv,6g2l,5l7k,4j7d,5fl5,4dhq,5ho8,5xst,
5jiq,1em6,3c1x,3ibc,3uib,4q15,5lax,4loy,1mau,2yof,6b5j,4cg8,4a9i,4xx9,5wbf
,4fse,5ahu,3s68,3tza,3oeu,5a85,3aza,3hdk,5bnr,3f3v,5cp5,2xqq,2w70,1aqi,5jf
5,4j3i,1s5z,4ahu,4nwk,1q6t,3gds,4gk2,2vex,3mxf,4yv9,4aft,4lop,3k16,5tuy,5j
fp,4wsj,1qin,3uol,5hlb,2w87,2wpb,4j24,1pxm,1uz1,5kbq,3qa2,2qtt,1zpa,4aba,5
mob,3chq,1iky,5aqv,4yyi,4clp,3kku,4x5q,5kap,2j7e,6fe0,4a7b,4e3n,5aqn,3dpc,
5ia3,3od0,1c6y,1a8i,1qpb,4ouj,1prm,4gii,5z5f,3aw0,5vb6,3krx,3lpb,2vxn,1dth
,5kqx,4x8t,1swr,2oxn,3ryv,3g2t,3ry8,5ks7,5mri,3nyn,2bu5,2p3g,5ti6,3e73,4fl
j,4drn,5j7w,2j47,5sz2,4ad2,5hvs,4wxi,1f8b,2vwu,1y1m,3su4,6c7f,5i2i,2sim,1o
0o,2a8g,5tha,3omg,6cjy,4m3q,3jzi,3skh,5eob,4qfs,5ty9,2ai7,5lyh,4kup,1zd3,4
b6f,6gvz,1xo2,3uz5,4nku,5a1f,5nt4,3tam,1mik,1thz,4q81,1dxp,2q8m,5uw5,3c3r,
5azf,1p10,2i0h,3p79,4c35,2q2z,3vb7,4pl4,4jps,2ykj,1auj,3mmr,1lvc,4d52,3hek
,4ywa,2ccb,5f3z,5j87,3f69,4res,4fz6,5l2m,2qx0,3vap,3pdc,2ym6,3f15,4erq,5o3
r,1yei,3w69,1qbn,5nq5,5anu,2cv3,3eio,1zdp,4wnk,3ebb,2vth,5h8g,4c6z,2fes,4a
g8,5ovp,5q18,4dkp,3k4q,2bdj,5x9p,5j1v,4o70,3v8w,6cbf,4iva,2qoa,4b3u,4nmp,1
e1v,6gjj,4qir,1b6m,5w3i,5n8e,1rin,6d5j,2qwb,5buj,3qtz,3t4n,6ccn,4dcv,1k03,
6ar9,5nz4,4x9w,2fx6,1m7y,3gt9,5uor,3nal,4drk,3fdt,2xyt,1hty,6rnt,4cga,5cxz
,4fzc,5wio,5hg9,4jpa,3zmh,2lsp,5hn9,5syn,2ojg,5kab,2z8e,1bhx,4u82,5n2t,3ow
3,5dp6,3dej,2uxx,6g38,5myx,2p0d,4yo8,1nzl,5ows,6cdo,3avf,5ual,1swg,3sm0,4y
k0,4wz4,2vx1,6er3,1gui,2bfq,4an2,4umc,5ct1,4x7j,1bma,4jwr,2ya7,3iqv,3oys,5
mxo,5jqb,3m67,3zim,5ktw,4c8r,2zx5,3tv8,4g69,2boi,4j8t,3ftz,4kai,3ggv,3rey,
3pb9,2wcx,4or0,4jsr,5ncz,4kmu,2gqn,3lzz,3wig,4z16,1umw,4dhf,5thj,3o6t,3s1y
,4x9v,1xgi,1fls,2a3b,3qcj,4k10,5e2s,1xgj,1kui,3w5e,6apv,3g4l,3uod,5os5,6g2
8,4jog,5yfs,6eis,4ehz,5d9l,4ryg,3luo,4i6q,2rr4,6hdq,5g10,2wa4,5zk3,5alc,5b
yi,3tge,4b95,2jt5,3rxb,2j7g,4w97,5axp,4l2l,5mxv,1ucn,2wk2,3kze,5exl,3ivq,1
msn,6aqs,2j77,4q4i,2wpa,3miy,3owj,3iph,1eol,5j75,4dfg,4phv,2xei,5t7s,5ggk,
3art,1m7d,5awt,6ayh,4ht2,3cke,6atv,1hvj,1pxh,4ozl,3rxk,1db1,3uwl,4ca4,4zcs
,3k00,3h03,3ao2,5k0i,2h4k,1ec1,4iaw,6fjf,1avd,4pyx,4mg6,2w6t,6fky,2ydk,5ey
c,2c6o,4ufd,2i1r,6ezh,1m4h,3fum,2r0z,1ecv,3qmk,3asl,4agl,5oh4,5fh8,3w5n,1q
q9,4gjc,4qfl,6afh,1f3e,2wzf,2epn,3uef,3kfa,4y5d,4abi,1qpe,4ixh,6aud,5zaj,1
nlt,2os9,4j44,4ra5,1g35,3ljz,4v11,4u1b,4mc2,2ate,5es1,5exn,5ngr,3cyw,3kme,
3k3e,2cma,2bpx,5i7y,4mvy,3s3q,3nzi,5enm,3s0j,6czd,5j5t,4dwb,4ob0,1ek1,3dpk
,1xos,3apc,4rn4,1u2y,1gfw,3hzm,5h9r,3d7m,3el8,4hy5,3eft,3udm,4yoz,3nrm,2zv

j,1ezq,3rlq,5vqx,5q0s,2vte,3rx5,1cyn,5t4v,1w96,5mwz,2f1g,3mtb,5lne,4yo6,5q
a5,4fk6,3uvl,5mks,5opu,3qnd,2o4l,5n0f,1yhs,3th0,5ka1,5vl2,3eky,4d8s,1wb0,4
ffs,4hv7,5jxn,5nu5,3nc4,5mz3,2xyu,4mjq,3ej5,3vid,1td7,6cwi,1grp,4ap7,1bxq,
4man,5fe9,1d6v,4n9b,2vd7,5fni,4b8o,3t1m,5jk3,5ti5,4i5p,4byi,4tzm,1lan,3gtc
,1q6j,3fts,1o30,3dct,3hmp,3q7p,4j7e,4uil,4z8d,5nki,1il3,4x49,5l4i,5wfd,6b7
h,4z1q,4m5r,3cs8,2o4z,2qcf,2v13,1kav,5h85,3m57,3c2r,4dpf,2qe5,3b2w,5oug,5l
6h,4jpy,3tvw,6esn,1v2t,1npa,5eis,1xur,5jyy,1t1s,5l13,3g76,1qkn,1g3e,3wqm,6
fnq,4umb,3qto,5jf2,5dw1,5vdo,4ruy,3igv,3zxv,5yid,4c4f,3d27,3qbc,4md6,2hnc,
3f36,5pzn,3a1d,1bl6,2vd0,6bh1,3d4l,6b67,6dh2,4css,5tco,4pz5,5i25,4g0q,3dla
,2w76,1pwq,5x5o,6mil,1fh9,4ybj,3lvw,5sz5,5wqc,4lm2,3v5p,4mz5,3hxi,5ia4,1nd
v,5qc4,4xtw,5zob,1pkx,6dl9,6dj5,5yjf,4b78,2vnn,2c9d,4b00,3oaw,5j0d,2yke,4m
su,5wcl,3ads,2fgh,4nuc,1jn4,2jew,2z7h,4uyn,3g9n,5zwh,3wqv,2pgj,6e9l,4ge6,2
hnx,3f3w,5y6e,4pyo,4zim,2zlg,1m74,6bqj,4mcc,3pqz,1k22,4o42,4rlk,5f1c,2gm9,
3sm1,5tho,5nwi,3cr4,4rhx,4fxq,3dv5,1fao,3mhm,2gtk,5vr8,4xum,2a3i,5l3e,4qw6
,3kdu,1e5j,1z5m,4dgo,3moh,5mys,4k5n,4ps8,4ym2,4eyr,4abe,6aam,1o3k,5c84,6ar
j,2w16,2w1i,3a1s,3abt,5nvz,4n99,4lnw,3w32,5uy8,2v3e,5yyb,4y8z,4pyv,4umu,2i
w8,5ael,3oe0,3ibu,2wfj,1ycm,2q55,4ow0,2p8o,4tqn,5tzd,1b6j,4bgy,4r8y,5l17,3
mt9,6eqm,4ht0,1n9a,1usn,4wy6,6g91,3b8h,4usj,4i72,5tzc,4wr8,4wk7,4qxr,3zbf,
5nyh,4eoi,6cyb,4heg,3qtr,4jik,3nsn,1i9m,2fme,2ybu,5jiy,3oy8,5ctc,2xcg,2qoh
,3lf0,1jr1,5alm,3ufl,2o5d,3zo1,1a86,3wv1,1snk,5kx7,2q1q,2vvv,4hlh,3gyn,3zx
z,5wf6,4u5o,2gd8,3ocp,5tq1,3fun,2ceo,2gsu,1dg9,5xig,2xpb,4fpf,1eas,4ylj,3q
6k,1yw7,4qz3,2wc4,4qz5,3wc7,5ni7,2oxd,2x4r,4gj2,1w0y,6ew7,6bg3,4ufu,5t9w,3
rum,5eth,4hhz,2qf6,5a7c,2h65,6e7j,3ekq,5dya,1q6n,3zt1,3rbq,3nw3,2q6c,4cd1,
4ym4,6cz3,5apr,3k3g,4urn,3rxd,1tkt,2gv2,5xg5,6bqh,5jg1,4gts,6ayd,3oj8,3wnt
,3ufa,5kxc,4zy6,4bcs,3rxf,3g8e,5d6f,5c42,3pp0,4u5j,3w9r,5dyo,5wyz,3n87,2oj
j,1c5o,2cmo,6guc,3djf,4b6c,3udp,3dei,4baq,1vj5,4jg1,3iop,4uua,3wnr,5hrv,4c
l9,5fyx,1o4g,3th9,4hgt,5mgf,4cy1,1f73,3g2i,5wyx,2xck,5u4d,5i7u,1zvx,2jdn,6
fdt,4pgd,3p3r,1o3j,1olu,3mfv,4re2,3hbo,4dew,3t1l,1fq4,4muf,4f9g,5nu3,1v2w,
2amv,4l3o,5owa,1n43,4xta,4m8e,6fnj,4eg6,5afl,3ij1,5dey,1w0z,2etm,2y5g,4z93
,4pmt,3wcb,4pms,1jtq,1t7r,1jj9,4ool,4cc5,1hv5,3t60,4deu,4qyy,4bg6,4u2y,6m9
f,3fz1,5hrw,5alg,2y3p,1it6,5fso,2l98,5ags,3g6g,5y62,2yig,1yi3,2ycf,3suf,3b
tr,5kql,3uo6,5or9,4u0b,4z0d,5yz2,1c3b,2arm,4o7c,4yjl,6cn6,1f0u,6ew3,4ap0,1
y2c,4yrt,6f09,3duy,5ayf,3wmb,6f9r,3oil,1mfd,1ymx,2avi,2hds,3ohf,1ado,5ur9,
5t54,5lhu,1wtg,2yb9,2h1h,3f8e,5e80,1p03,1okv,1fkw,2l0i,4zae,5iha,5c1m,2q8z
,3jy0,2qe2,5tzh,3ttm,1azm,2uym,5tzo,3pck,6b95,4joa,1gz3,4deh,6euv,5fsy,3wd
c,1s3k,4kod,3f9w,2bks,2flh,2vb8,5nhp,2qlm,5o4y,1pph,3g0d,5ajx,2p0x,3ihz,2y
2i,5aep,3vjm,2a58,4bdj,5w4w,2yog,4nrm,2i19,2jup,1tvo,3blu,3twv,3hl7,4no9,4
hlg,6mi6,3r6u,5tqs,3o4l,4p5e,3elj,5icz,6ezg,4u68,2pj0,5a3q,3u51,4i73,5g46,
3au6,4x47,5edq,4lbp,1jmi,6cvf,5f3c,3l2y,3wcf,2yns,1epq,5k0j,1eoc,4idz,3f7g
,2xnm,3kqs,5fsb,1onz,1xb7,4q87,3p58,1jsv,2peh,2wl5,1ohr,4wyy,4dmy,5abw,1hg
t,4b76,1fq6,5ar8,5vlp,3gjq,5g0q,4jkt,4duh,2wi5,2hog,3mg0,3zly,1ke8,4nxs,5m
zg,3bxs,2r1y,2c97,4xy9,5xv7,5umy,5irr,2ql9,4qfg,4c4i,4zme,1me3,2gst,5ue4,5
esq,3mkf,3djx,3cl2,5f4p,5muc,3ipy,3c43,3dsz,1joc,3hqr,3ftu,4mrg,4bhn,4wgi,
4q1c,3dhk,1jlx,2vl4,4io8,4rqz,5fbe,1icj,5lpl,4ret,5svl,1zkn,4xhe,3zmz,5mrb
,4bcj,1gwm,2jfz,5qb3,2z4y,5wap,1trd,5fnu,1inh,3puj,5n84,6cvy,6ceh,2xht,4m5
o,5ll4,5aki,3m1k,3tki,2aq7,2xk3,6dm8,2j6m,3neg,1acj,2r4b,4ynk,3qfd,3kjq,5n
x2,1d9i,2qk5,3cfs,5w44,6am8,5iua,3ert,2rcw,5he5,1wn6,4zup,4l31,3zi8,5aix,3
ftq,4wwp,1xh6,6ftp,4az0,2zu5,5j64,3k02,6ccl,3q96,3lok,3zps,1hef,4iks,3sov,
4nmt,5dbm,4km0,5u0d,1ybo,1eoj,2ew5,5fxr,6gl9,4jof,1o3b,5vrp,3cs7,3tdj,1qf2
,3l3l,2gfa,1jwt,6cnk,5u5t,2e9o,5wrs,4ijp,4jfz,3k9x,5cst,4bcn,3gb2,3ds1,2pv
l,5ime,2brc,1utn,5ult,1nfs,4pd5,3zuk,2az9,3c79,5eje,4dfn,5n87,4uia,4ozo,2h
k5,2qrh,3oc0,2adm,2z92,4pg9,1rs4,4ah9,5o91,3eor,1lf2,4djh,2y1o,9lpr,4od0,6
eq1,4kz5,6g4z,5ko5,4x5r,5tnt,1eed,1e55,6b59,1al8,1mrx,3b82,3oq5,3ns9,4ppa,
3uw4,4r5w,5jr6,1a9m,3ft8,4oeg,4lrr,1tys,4f4p,4knz,3wb5,4xue,6c5t,3opr,4des

,2mg5,6goo,4rak,4z1e,6g2a,4aqc,1zkl,3ijy,1uyd,4avu,2ou7,4isu,4az2,5eom,4q7
w,5yie,5ey4,4ezx,3x1k,4rrg,1osv,4w9o,4z2g,4zt3,3uxg,3ig7,5tg6,6fqo,4dum,2a
0t,3o9d,3m3e,3arw,3tkw,5a4q,4zzz,5dk4,5wfj,4rj4,3atv,2of2,1hi3,1u9l,3hiv,5
k6a,2k3w,4nbl,2x6x,4mcd,6c7e,5tgy,4jyu,5jop,5vd1,5gty,1pme,2oph,3zcl,1yon,
4g3e,1h5v,2y2n,6bny,5d11,5yve,4lng,4ei4,1c86,3n7s,3dst,4nah,5yz7,3mxy,5uoo
,1ajv,5mim,6bgz,2p83,3fhr,5n7v,5j8z,6fns,4c37,4g0l,2ds1,1pf8,2am9,1nz7,6hw
z,3o8g,1n4k,4m0e,5aqr,5mte,4aa1,4txe,5t8r,5vm0,5xyz,3k1j,3kpu,4alv,4u43,5z
o7,3dp2,5drr,3zjv,5q1b,5zz2,5o0a,6b98,5bwb,2o5k,4bf6,3rxo,2br6,3czv,5o0j,5
elq,2yiq,4gtr,1svg,6f3b,4awq,2e9a,3d7f,5am2,3ai8,5y1u,3clp,1gsz,6fzx,3f7h,
4gzp,3str,4x14,3ued,5npc,3gxy,5ads,4aj4,5one,5dxt,4whz,5lyn,4l2g,5l98,1au2
,5e2k,1u0g,6g2m,4p75,4nmr,6ex0,5n2f,3dvp,4mji,4m7b,2l7u,4q6f,2ko7,4yml,3vd
9,6g6z,3ppr,5t1t,5ksu,5t2t,3wke,2vww,1uu9,2flb,1r0x,5o4z,1w1y,2ans,5anq,4p
zv,5iu8,4li0,4xtm,3c10,3vuc,1hvs,5gic,4yv1,5b5p,2bcd,4w9j,1n51,4bbh,2azr,1
axr,5nk2,3znc,5v1b,1o5a,1hti,4uj9,4tmp,5al1,2yme,6bdy,5hvt,4ceb,1i3z,4rkx,
5g22,1ej4,1eb1,2w3l,1ghz,3ljt,6dak,2h9n,3ljj,1ela,3zot,3kry,1xh4,5tv1,4fck
,3d83,2vci,3tyq,2az5,3hwn,5em8,1f7b,1ijr,1ktt,1ha2,5mxk,3myq,4na4,5a5z,3su
d,5j1r,1bnq,5nxo,3qpn,3mfw,3veh,3s2p,3i28,3djv,3n4c,2fou,1wcq,4pov,2w67,5m
f6,6hsz,4f39,2uw6,3q3k,3blt,1elr,1b9t,3mnu,4xg6,6ge7,2ole,4p6g,5hff,3fck,4
lqg,3ery,1rgk,4j70,3ob2,1q3d,5a3u,1utm,4xg9,4wvs,4piq,5hkm,4ui3,5ei8,4oon,
5gof,2vpo,1pmv,6evn,3uqg,4zly,4hnn,4anw,1jws,3ejp,2hj4,4wy1,3wv2,1b5g,5u0z
,5m04,1pye,6fng,4xek,4q4q,4awj,2vx9,5ksx,4mic,6ea2,1j4r,5igk,2q6f,2pv1,2vi
5,4tw7,1ftl,3lir,4ir6,1oe0,3gxl,3zbx,5tw2,3kag,2v11,5yun,2w6p,2xke,5yl2,1m
48,4ery,4pkv,3k3b,3lau,4oc4,5nwk,5lud,4h39,1kfy,4x7q,6ex1,6c0u,3l9h,1jg0,4
rrn,1dmp,5jrs,5hyq,1tv6,5ovv,4ibb,6dne,4lgg,4z1s,3q5u,5ji8,3ddb,3hhk,5fiv,
6d4o,5v2l,3lnj,4odl,2qi3,1kr3,5t9z,5aut,4lbo,1tsy,2v0c,3r0h,6er4,4qwg,4aif
,3n5k,5d1r,4etz,4i47,1z3c,2wb5,5xup,2fpz,4m2u,5f8y,3n2e,5lcf,5bue,3mww,4n7
g,6epy,4jj7,4dgr,1d3v,5upz,3itz,5agv,3pwd,3ny3,2xvn,3wym,5cal,5kup,6h38,2q
lj,2qyk,3f3u,5ir1,4oc6,2pj1,3alt,3zyu,4ydq,1uti,2vnm,4r5n,4e1k,4yv2,5enk,4
n8q,3bgb,5e0j,1t48,4pnq,4ogi,4qls,4o4y,5ztn,3nnv,1x9d,3d9v,1bnu,2jh6,3r8v,
4n9c,1dkd,5f1h,5fjw,4x8s,5n3v,3f34,1ppk,4a22,4rj8,1d4j,4y73,3buh,3lhj,1gmy
,2w7x,4cts,3pj2,3qxt,5bvo,3ouj,4tjw,4de5,3ipq,5ybi,4azb,4z2b,4zyu,1o2s,4r5
a,4d88,4yat,4u6e,5ttv,3kyg,2yix,5vp1,3hqw,5oei,1jmg,1ze8,1csh,3lbj,2wik,3v
d7,2xcn,2ypi,1xhm,4c5w,2xyn,6dh4,4yv5,4qmx,3ocg,5nxw,5ejw,5lav,3l3m,4nxv,4
kzc,5g53,4wmv,5x4m,5ulg,6ayo,5l3f,1k9q,2vo7,4jhz,5vzy,5alu,3q8h,4b72,4os5,
5alp,4dfw,2qzx,1okl,5a6a,4rx7,3cf8,4aj1,5x4p,1vyg,2zjw,2uy0,1k3t,2xo8,5ehp
,1bjr,4btu,3a4o,4hzw,2odd,3hmm,4xpj,1a3e,4wz5,4j17,3ekr,5ttu,3k8q,1qhr,4aw
m,4rg0,2hpa,2w12,2ohk,6afc,4xip,1h1r,4b34,4tw9,5m4i,3exh,3i0s,5ji6,4tzq,5a
lo,3hf6,4b35,5oxg,2wej,5iu4,1zp5,5he4,1amn,4wey,5klt,2lyw,1w7h,3rni,5llc,5
ou2,2r3o,1pau,5akz,1tkb,2piz,4bw2,5nzf,4mww,5tpx,5jau,5faq,5ak4,3qps,2c80,
2ggd,6f1j,4ncg,2r9s,4x1p,1sqb,5to8,1ai6,1h27,1jvu,5h6v,2pk5,6gbx,2c6g,1f8d
,1bzs,3h26,3h59,1nlo,2o9v,1rhj,4z6h,4tun,6ar4,4ovz,5e2w,5eh8,1jjk,5uez,3fw
3,1f4y,1yds,5fol,5l72,3qsb,3g42,1lrh,4ibc,2d06,5vqr,3nf7,1qbs,1eef,5cf4,2e
9u,1vyz,4wi1,5wqj,4o62,3isj,4br3,7gch,4wmu,2xlc,3fuf,3mna,3tpx,4mnp,1yvm,4
j0z,5y61,5v0n,5qap,4xua,3q4j,4mw4,1nt1,4wt2,5w8h,4zlo,5lgt,1hbv,1xp0,4hlk,
3qtx,1r17,6fmc,1fyr,4fab,2oi9,5o48,5jgi,4ngq,2qic,3u92,2y2p,2auc,5lca,2w6m
,3hii,5odx,3uzj,1b0f,1pxj,5mod,2kaw,6b5o,1v2p,1yy6,5dsx,5fdg,5jf3,5ap3,3bu
f,1gx8,4n4s,4z7i,2ypp,1jlr,4xbo,5w0l,2c5y,4mp2,5i3m,3kqm,5jyo,5a3r,5hm0,2y
mt,4ai8,2opb,4rd6,2q1j,3hp5,3nkx,5f6v,3piz,3d1v,4lv1,6fer,3ud7,6e9w,1caq,3
ts4,4tnw,2jg8,1ga8,4f6s,6en5,4a6w,1lyb,5eds,4uct,2z1w,4fsl,5lsc,4zj8,2gek,
3cyx,5ti3,2qrk,4wri,1nox,5lma,4j1k,4k1b,6b7d,3q7j,3s74,1x11,4x6h,3wyy,1tyr
,4tk1,2yj9,3n1w,3otq,5yia,4li5,4zsl,5fyq,1h1h,3kx1,4zs9,5if4,4p90,4z7o,3p5
0,2r6w,4djy,4tjz,4py2,5e2r,5l2w,4m5u,3wbl,5ory,3tl0,1ro7,1ok7,1f92,4j1e,5t
p0,1aqj,4yp1,6eil,4l7r,4lwe,1zm6,4y2q,4cgj,4b60,3aje,2j27,6fgy,3ua9,5ujo,1
om1,2x7o,1h8l,3tdz,2y2h,5bmm,6f6d,4ylk,3jdw,5aml,6e4f,5v79,4yhz,1g3d,5j9z,

4ucs,2c5x,1aq1,2hdx,1a4k,5d6p,1tlp,4jv8,4gql,2xru,2jmj,3wiy,1ru2,2i47,6fs1
,6hvj,2chw,4joh,3te5,4z9l,4ocv,3pwm,6b1j,2vj7,3lkz,4wef,3bi1,6b2p,3ms7,6ap
8,5k4z,3bbt,3r93,5npe,4kb7,6got,1v2n,4e49,2r2l,2qhd,5i9i,5t36,2v10,3atu,5n
db,1tve,5epk,3emh,5hln,5nsx,6dlx,4ayv,4ca5,1e9h,3vqs,4ii9,4hn2,3jpv,4hby,4
eny,6mnf,1m51,3opp,3h2c,3lpf,5oxn,1nzq,4anb,1zfq,4o3b,2xuf,5mhq,5fpi,3tf7,
5zh3,4o07,4a7j,5yyz,4mw1,5f67,2fb8,1rdj,4b9w,6g9u,5e89,6ayn,3tfu,3g3n,4asd
,6hgz,3fcb,2gfs,5q1i,3b3c,2e91,3iob,5n4t,2hz0,3hl8,6dik,5vii,5jsm,6fba,4i4
e,4cpq,5he7,2r4f,5a3o,1kne,5qbv,4ft2,2boh,3n45,5mnb,5erg,3dga,2jbu,4jhq,6c
hn,5i0l,4odk,5w6t,2ycq,1rhk,2p2i,4lkq,4h1j,2wi4,2qhr,4yqu,1czk,1bsk,4e35,5
i5x,5lj1,4wbo,5w6r,3b0w,3ntp,4hy4,1mdl,1zxc,1d04,2g00,4acx,2i0v,5dd0,4ixv,
5l2o,4ya8,1ths,4r4q,3qw6,4wz6,2uuo,6ami,1njf,4d1c,4m3e,1xxe,1so2,5gow,5i23
,2oyl,6e59,5ih2,5zc5,5gja,4w4v,5fsx,2fqo,6msy,3o4k,1v1j,4o0y,4x3i,5mka,4gs
6,3mvh,5etl,5jt2,4k6t,2ada,2f6t,5m9w,3dog,1rry,1add,5n21,2a5c,1uvs,6bgy,5e
1o,6f8r,3ru1,3zvw,6ghj,4w9g,3ith,5o1h,5ne5,3qpo,6cze,5tdb,3qi4,2xdw,1wax,1
q6s,3fv8,3m3r,1qw7,4jdf,4xu0,5nvc,4pum,4yl0,5lb7,3zmi,2nns,1b2i,1epp,5c7b,
2fsv,4i9z,3tku,6cvx,3o1g,5vlk,3loo,1a37,5du6,3coh,4jmx,1nj1,4mrw,4jne,4i8x
,1rti,3c9e,4k3r,3zlr,3t1a,3uo5,3eqy,4x61,4hmh,4uxh,1axs,3gy2,4xx3,5can,2gg
7,1c5n,5klr,4y0a,4gvm,1pk0,2zxb,2v54,5he2,4cwt,2fxu,4mi6,3o9g,5tts,6f5u,2x
iz,1bcd,5x54,4dx9,3ump,2xj0,3suw,4hfz,6gmq,4hkp,2x4o,1utp,3ddu,3uzd,3bun,2
h5i,4zyy,6duf,5f2w,4g2w,4an0,2gz8,4pce,3bvb,3my1,3qvu,6gfz,4fak,2z60,1vyj,
5jzs,5afj,3rv6,2ye9,5zuj,3uza,3qxh,1m2x,1gux,4lh7,1sh9,5abh,3rwf,5nkh,5u2c
,2qk8,3tcy,1l8g,4dy6,5ouh,6dh8,3saz,2ntf,4obp,4yux,4g11,3tiy,5vb9,2op9,4nn
n,3kwf,5n69,3cl0,2or9,4k6v,3iqj,2nnv,3ecn,5c7a,4prd,3zp9,3fv3,3ha6,4ahv,4m
vh,3u18,4ks3,5m57,3ik1,3are,3wc5,4h3f,3rik,3t0d,4iqt,1udt,4xo8,4d9p,5uqx,4
c16,4k78,3e8n,4qz4,3v8t,5mnh,4qpd,2ow0,3p2e,4gki,1akt,2y7w,4av0,6frf,4umj,
4u6z,4kn7,4yuz,2kbs,1err,1qbo,6axp,3ms2,5t1a,2yi0,6b2q,1bwa,5km1,3wuv,5y80
,4waf,5nkd,4cwn,5j9x,1mu8,5vex,5i2e,2xs8,3rz9,4bdh,4h58,3ho9,4fi9,3qkk,3q9
2,3k3j,1afk,3ivh,6cf5,4r6x,5ugg,3rl4,3hmo,4obq,4ycv,4wpf,5g42,3si3,4j82,1h
ge,1om9,4b9h,5tkd,2c1p,3o95,1pz5,5ug8,4rj6,4zw3,5o1g,1z6d,1bug,5b1s,4prj,5
ljt,2vnt,3u4i,2yem,4zsm,4ibe,1obx,3e37,4bw3,3el0,3f81,3udy,3lj7,4ov5,2jbp,
4gw6,4y6m,3cso,4qc1,2ccs,3uw9,4arw,4cwo,4lkd,5v1d,4mwv,1gzc,4u0f,4h3g,4hlm
,4urw,1f90,1pdz,4j3m,5aag,4hzm,3ti1,4q3s,1qpf,1ouk,6enm,4glx,3q6s,3d9p,1cw
b,3bjm,4mma,3veu,3p8p,5gr9,3s0n,5aia,5acx,5lub,4kp0,4q3q,6gg5,4gm3,3qiz,4f
cq,6cdg,5u8f,1pdq,2vev,5j82,3daj,1sfi,3t0x,2x38,5f1l,4kwo,4buq,5ap1,2xey,4
ozj,4d89,1avp,2on6,3nlb,4xk9,1tnl,3dxk,3cj5,1m2p,6dqa,1ejn,1xor,3pxe,2pzy,
5whc,5wal,2zq0,3old,5cbs,4jck,3su1,1t29,4mwc,5j2x,3poz,2cbu,1csr,1rrw,6evo
,4v0i,4jv9,5u4x,5ho6,2w1d,3h30,1w10,2ovv,5l8a,2yir,1y2a,4x6k,3iqq,4b6q,1ok
x,5vgi,4uv9,5vqu,1fhd,3mn8,3p44,3l0k,3kga,4gr3,1f4f,5wdw,4p1u,4p4s,4fob,3h
hu,3rz5,5a5r,2ews,4iie,5i89,1g30,3d7g,4j26,5juz,2wzx,5drc,1ttv,5d1u,2jjr,5
jpt,2i03,2psx,3vg1,3o56,3su5,4ab9,4utn,1yz3,4bae,4mwe,5jgq,5dyt,6hm6,6cwn,
5yy4,4e70,1pyg,5vja,3zki,4d8n,2gg2,5vkc,4hup,2wxm,1uyk,6bh5,2izx,5u69,5jrq
,2xfj,2jke,4n5d,1uh1,3kv2,3p9h,3bl0,3dab,1g27,4d83,5u5h,5elv,3egt,5u9i,4rl
w,2op3,4qok,3s5y,4av5,4zx1,1fiv,2g72,3iad,5vil,1py2,3nmq,2wec,5y0f,1jlq,1n
4m,3w1f,4x6j,2kpl,3hp2,1ny2,5jaz,5mme,5ntp,5fi2,4o1b,3ogx,6g0q,4ysi,4amz,5
fho,4uiv,1msm,4ui6,5byy,3qtu,4ucv,4dwg,3pr0,4kqq,1q41,5ybe,6h7n,2nxm,2rg5,
5nb6,1ws4,3pgu,4no1,2o7e,1pzo,2afx,4u58,2wl0,3ttz,4uc5,5eyd,1wht,1bl4,4n5t
,6dki,2ajb,3i6m,5khh,2fvc,1w83,4qxo,5tpb,4hco,5yzc,3jyj,4qvx,1v1k,4cmu,5hw
v,3ahn,1efy,6h41,2xtk,3h0e,4c1t,5kau,6fmk,4ie3,5jxq,3pcn,5jid,4zow,2vc7,2f
10,3zmp,3ppq,3qfv,2jnp,5k7h,4q1b,5lcj,4ohp,3l3n,1kll,3unj,1ngw,2ym5,6cvv,5
t18,4l51,6duh,5i59,1lzq,5cte,1qxl,3qkl,3k5f,5j31,4c5d,3si4,3nu9,4g2r,2vj6,
4lkt,3g5v,5ow8,3ua8,4kp8,4mk1,5h2u,6eum,4q0k,2x6d,4rz1,5n0d,5d6j,5evd,3d51
,4uv8,2iw6,5op5,3ff6,2btr,1p2a,5cwa,4llj,5h5o,1wvc,4isi,5ngf,3vw2,6cdj,2ns
l,1esz,4bxn,3gww,4ach,4iid,3wdz,3b3x,2wuf,6e8m,3sou,3wyj,5fgk,4gng,4uzh,2j
do,4d8c,1wdr,5er1,2xyf,2wgs,1mwt,5k9w,3kn0,1skj,4tyl,3cqu,6cgp,1utr,2h6k,5

mxf,3gvb,3ttj,3ctt,4lps,5hg8,3exo,5ieg,2f6v,5xvq,5m3b,3mta,6bqk,1tfz,3t6b,
5ljq,3oy3,3pcf,2lct,5dh4,6dhc,5nev,4xg4,4lvt,4dr9,3d45,2vo5,5v1y,4lq9,5jt9
,6cen,3uvo,6flg,1gx4,2web,4lpf,4hki,2xh5,1qka,5ogk,4yad,5lrj,3hmv,3l3x,4mi
b,3rwp,4fl2,5ml8,5elf,1i2s,6f92,1sm3,1stp,5zae,1ew8,3s1h,2yer,1l5q,4cdr,5a
yt,2agv,3b1u,3m6q,4jnc,6b1o,4cfx,5wlt,5w5o,1ta6,5x26,3rwi,4qqi,2hz4,2y71,5
jv0,3u6w,3m11,1r1i,3phe,5dry,5yp5,1ett,6gla,4jjm,3fzt,1e6s,3l08,2pj2,1fw0,
5j6d,4lm0,5hdx,4xg7,5hl9,3oyw,2phb,4c1g,6axj,5ur5,4pct,5w0i,5hbj,2lwi,3idp
,4a7c,6cbh,3i9g,1o2z,4mw5,5xhs,5bui,2xel,5aip,1gah,2gnf,3bwk,1hmt,3tgs,5q0
e,3udk,3sv8,2jkq,2wf5,4prn,4l53,3nef,2hxl,3f5j,5lt6,4gjb,6eyz,5cy3,2fqt,2y
im,4ln7,1olx,5tvn,1doj,5y0g,4dve,2np9,5wf3,3sxf,5emk,2i80,1fzk,5j32,4u0g,4
feq,2p1c,5w5j,2xdx,3vtc,4i2z,5nn5,3diw,4e81,2q8s,5vcy,2f1a,5ets,4k64,3mhl,
5mev,6an1,3mz3,6htg,4u0d,1kug,4qxj,1ent,5tza,4iur,1igb,5cc2,4jfv,4py4,3mbp
,2q8i,5trr,3fqe,4o43,5i3x,5uv1,4d8a,3d7d,4djw,1a94,2e9n,1zsb,2oaz,4m4q,3dc
w,5yjk,5aqf,2yoh,3so9,4q1a,1gqs,3mmf,3zxe,2cm8,4rn1,1jmq,3itc,4r17,2pu1,4j
09,2x6e,1wok,1t37,5lvd,4zsa,4rss,3ggu,3r02,4bjb,5wzw,5dhf,1eub,6cv8,6d28,5
hjq,5jyp,5u0g,1mfa,3suu,4inh,6c28,2cbj,3nba,1him,2qq7,4aq3,4tkg,4urz,3ske,
1pro,6cj5,5a5o,5qaw,5cxa,3uo9,2zgm,6cex,3vjc,1utl,1bnw,3iu9,5t6j,1w9u,5t37
,1btn,4z1k,3mo0,3m2u,1uv5,5ajc,1sdv,5hgq,5ixq,3s45,4xc2,5cxi,4hww,2wi7,1v2
r,5mwg,1qxy,1hp5,5cav,5qag,6hqy,3l4w,2y0j,3pkd,2uyw,4j0v,2y54,5u3b,3p4v,3g
s7,4rsp,3g2u,4pp5,5i43,4xm8,3eka,1oim,1px4,2zaz,5mqt,4ggz,3fc2,1z6s,1awh,1
t79,1h25,3w37,3dp9,5fl6,5eym,2fqw,4yrs,2hmv,5aen,3ckp,4ydn,4zjw,1yp9,5uov,
3k8d,4zyt,4eft,4zmf,1ikx,5dhj,2jt6,6fr2,4oue,4jfw,5ywx,1tnk,4qp7,4f1l,5a2k
,3kvw,1py5,2etk,1lhv,2g9q,3v4v,2qu2,3gs6,5mar,6g1u,5e2l,5eeq,5ax3,5lsx,4eg
4,2hwi,5l87,4utx,2hf8,3kl6,4y8x,3m94,4u69,4anu,6fnf,3qti,5v13,3uli,4a9r,4c
jn,3i5r,3eax,1jje,4qgf,2i6b,4aq4,3e7a,3fh5,1y3x,4krs,3ldw,1lqf,3sug,4ezw,2
p3c,4c1h,4awp,5ap4,1q5k,3bxg,5mm9,5ehr,3nus,4a2a,3hzv,1g46,6d2o,5ehn,2f14,
2xb9,4jg6,4qna,1rhu,1hk5,5d3t,4j22,2jk7,4ty1,4idt,1hvh,5zh5,4erw,4knr,3hv6
,5mwd,2xu1,1gja,4yhf,5dt2,3fkv,4ie5,4aa0,1xk5,1h0r,1bwn,3ctq,3bm6,2g96,2l8
4,3hvh,4fzg,3a2o,1xfv,3hku,1nq7,1tkz,4qiy,3q72,5enj,2evo,1uml,1xpz,4umq,2l
1r,1ke5,5mae,1j81,6bsl,4c61,5uv5,1hii,6f23,1atl,3gn7,4hmq,4zsr,3u78,3t5u,3
h2a,6e4u,5u6b,5dia,4oyi,4r4o,3g15,3okp,5y12,1g9a,4f63,3wk5,6cqf,4hwr,5xhz,
4qr5,6b1h,1u9x,4bcw,4jr5,4uhg,5yls,2wxv,3mpm,2x2i,1n0t,4o2p,4qab,1agm,3owd
,5fdr,2uz6,1gi1,4qmm,4rvr,2qyl,1i32,456c,4qw3,2y07,3d14,1u71,2pe1,4rgd,3su
0,5n1s,4gtt,1fl6,4lqy,3qtq,3wyx,2vfz,2p59,2aie,3t0m,3ifl,1cze,2ksb,6ee4,2c
4v,3zm9,4e9d,3tia,4p4d,3cvk,5oq8,5oq7,5jv1,1h1s,5alb,5vih,6ayr,4oc5,1xh3,1
xq0,1y3p,5fog,3tn8,1lhc,6gu6,3u7m,5koq,3tv6,2uw8,5opv,3g5k,3sv7,6eqs,3spf,
4bdc,5jfr,3dp3,5f62,2am1,3chd,6do3,4hgc,3c56,4j86,3su3,4m5h,4j5e,4wk1,1p4r
,2zyn,1nzv,3ah8,4nrp,5hey,3l4v,4cft,2b8v,3o87,2qmg,1x38,3t8v,4jr0,5heb,6ap
s,3mvl,2f2c,1yci,1tng,1fkf,1sw2,3f9n,2cmc,3q2h,4w9n,2xab,2qd7,5nau,5tbe,5k
00,2ieh,3wqh,3nsq,4xoe,5xpm,5opr,5ngs,4ezo,3p2k,5f88,4rlp,3e1r,5umw,6fsd,5
wi1,4k3m,5mrh,1qja,2wyi,2h9p,4hcu,2hd1,5tur,1iyl,5ylt,5a69,5f01,4an1,4kv9,
4eqf,1ao0,3vi7,2wu7,2wxj,4emr,2zoq,4fpk,4u45,3ddg,2xml,3ls4,5os4,3hio,1kc5
,4pg3,5uf0,1wbw,4z88,1zzz,3zh8,5iv2,5ehi,1ndj,3rhk,5in9,6bl1,4oyp,5ijp,2ov
x,5tg2,3kgt,4zg6,4ztn,1koj,2e99,4no6,3r9o,3axk,6ela,4det,1p6d,1h7a,4e3d,1g
3c,4ysl,3gol,5a7i,3uph,5tol,2vfk,2b1g,2ovz,3rdq,6cho,4hxs,5trh,2y5f,4n6z,3
nf3,4pyq,5k4x,1wbn,5wic,4pl6,2ica,1rsi,4m12,5x72,3bjc,5j18,3kqw,4axa,5kbf,
4bhf,3byo,5o55,3hd3,1tsi,1zgb,4yfi,1hgj,4tvj,6bsm,3t3i,5knt,3a29,1aqc,3a1e
,1ybg,5dhp,4b80,5ntt,4qwj,3k0h,2xn6,2rio,5ai4,4xu3,2vwm,1akv,6e06,6faa,3pr
f,3iss,5b5g,2bba,1v2o,1x70,6dil,5ngb,1njt,4jt8,4fxj,2glp,2w0p,4f5y,5v88,2o
63,1ksn,3zc6,5hna,6f55,4r91,2yj2,5mkx,2a4z,1t4s,4q18,4izy,4dsu,1juj,1iy7,4
qhc,5lla,4x1f,2ycs,5lwd,5v3o,3zjt,1f0q,6fbv,3iw8,2n3k,5gub,5fkj,1c5s,5voj,
5fbi,5wtt,4i7c,5etx,6d55,4uj1,4uuh,6bm5,4dt2,2yi5,4zgk,5fnt,2qbs,1sln,5c7n
,1pw6,1h62,5tz3,1q54,1a5v,1zge,5zfi,4dpu,1at6,3hc8,2weq,5d7x,2zzu,6hjk,6dr
z,5cjf,5osk,1jyc,5om3,3whw,2pyi,3t9t,4leq,4gkc,3kaf,3o0e,3u0t,6htz,4jvi,2g

gu,2w08,6g9m,3d2t,3nij,4ycm,3vhk,3sff,4twd,5ans,2wf3,3fjg,3lm1,1pcg,6fhq,5
zkc,1kyv,4y2j,3asx,1n1t,4oti,5am0,1bil,3g0f,5yc3,2lk1,5hrx,3m3z,1i9q,2f3k,
5czb,5hva,3mks,5nxv,5f20,5l3a,1hk4,5hlp,4yvz,3q2j,4p7s,5v7a,5wre,2q2y,1biw
,2jld,3gbq,5vew,2fyv,6prc,6f9u,1ydk,4ehr,2nnk,4qmw,2asu,4htx,6ey9,2wj2,4a9
n,5kzq,3qtw,2puy,5du8,1qm5,3fxv,1lbf,3zi0,5btx,4jit,2vh0,1epo,5hjb,3i60,6c
dc,4jg0,4b33,3oe9,4q3u,1lhe,5ov8,3bu6,3bh9,1ddm,3tws,3n2v,5h9q,5d1j,4l5j,4
j5p,1hfs,4whs,4xjs,5ftq,3hit,5w14,5efc,1np0,4o12,1orw,1ft7,2gfd,5xag,1ui0,
2bkt,4wq3,4hso,4a4g,2nnp,3m8u,4k43,4cix,3fzs,3sww,2qzr,3zev,4er2,2fl6,5kzi
,3ewz,1c4u,3zyr,3adt,3vtr,3s0o,4ob1,1o5f,3qw7,1fe3,3l58,4wn1,3ebo,3n4l,5zu
0,3tc5,3bbf,1fd0,3vi2,2f3e,6fkp,5sz0,5w8v,4h5c,2bpv,6acb,2ihq,3bxh,1w5x,2h
xq,4n7e,3vd4,6bt0,4h75,1hqg,1pr1,3psl,5dd9,3oy0,5n20,3wf8,4k0y,3gi6,4fli,1
uho,4w4w,1qti,3sfi,4mha,3ob0,4i3z,3erk,2h6q,1uk0,6gn1,2r6f,4nl1,4v01,3pju,
5ewj,2hl4,3juk,3c1k,1fki,5vc3,2vhq,4xnw,5u2f,4jz1,4drp,2oi3,1ind,2znu,3px8
,1mu6,4hct,4b14,4rs0,3bz3,1qbq,2v2h,3s1g,5ih8,4mm9,4r1v,3nrz,5ivc,2nt7,4xu
h,5n24,3l5e,4bio,6azl,2dq7,1t2v,4c71,2xp5,6g0v,2e9c,5e0g,2w77,5ofx,2zgx,4q
lv,4fm8,4x3k,2c6c,4d2w,5n25,3hub,3vva,1bai,3v2w,4ej2,1oe7,4ezk,2jkk,2kvm,5
j7s,3vfa,2z7i,3lhs,4ufm,4a9c,2oag,4b7z,5k8v,4pis,4na7,3c7q,5l8c,3t0w,3fci,
2w92,5orb,3uf9,4ijl,5x62,1kl3,3bhb,5ta2,4ih6,4o2e,1jn2,3rz0,3d4q,4dhu,5fhn
,1wzy,1jzs,3qxd,5z7j,4dfl,5orz,5dp5,3twr,5cs2,4esg,4q6d,3jrs,5wip,2fah,5kr
0,4qz1,2rke,5nps,3rz1,5v86,4olh,1cr6,5ak3,2x7c,4jn2,1x7a,6c7r,4d1a,4lv3,3h
ll,4wkb,3rwg,5ytu,3bb1,5yvt,1ilq,3vbd,1dbk,2rqu,2xc0,5wbr,1ppi,2xs0,2oc7,1
owi,5jh6,1ke9,2fl2,3oct,6ghh,5btr,5alv,5axi,2x4s,2bvx,2zir,1b6p,4occ,4k3n,
5ivs,3kwj,2ncz,3elm,4w9q,5w8j,3o9l,5iyy,2oi0,5h7h,5gvm,3ivi,4bi6,5k5s,2k1q
,3gsm,4int,1ai4,3adu,4zvi,4tkn,3nww,3eyh,5dtj,5adq,4my6,4pqa,1ms0,4kyh,2vu
3,3ds0,1fdq,3u7k,1d4t,1kds,5xn3,5qab,1joj,5ovx,4bch,5dp4,5x9o,3i6o,5fdp,1v
jd,4bcd,6fmp,3fvl,3r22,1oss,2hmu,2r3i,4bgk,4oty,5sxk,2pgl,2ydo,4po0,3wi6,2
aqb,1gwq,2j4z,5ezg,4kwf,4zur,2fq6,4mep,3e0q,5dpx,3lp4,1juf,5jcb,4j47,6dgz,
5c5t,5brn,5neb,1hc9,3oki,3ni5,4anv,1rpa,3n7r,1w3l,4f9y,4x6n,4qh7,2wi1,1m5w
,4zdu,6bhh,2ha6,4ybk,6fse,4fn5,5nkc,4uac,4ih3,5d3j,1ype,1ew9,5lm4,5jmw,3rc
d,4o0a,4pf5,3q4l,2f9v,5c2e,2yhw,5dl1,1guw,3t2q,4re4,1k08,5m2v,1yvz,5nob,1u
x7,2o2u,1fo0,4clb,3kgu,1mpl,3ho2,1sld,2qi0,5dkr,2fjp,2f5t,4dju,3f19,5jeo,4
kba,4uvb,4pyy,4ifi,4a6v,3w9k,4nhx,5xmu,4rvl,1qng,3wk6,5oah,5iee,2hkf,1rd4,
2wot,4djq,2am4,4auy,1qtn,3qak,4l09,2g8n,5tr6,4hwo,4oq5,5llh,3q4b,2e9v,3qk5
,6chq,2g5p,2drc,5tg5,2xk7,6dj1,3qd0,4zy4,1cwc,1uy7,5yu9,4nmv,5mno,3i97,4f3
h,2fgv,5akh,6euw,3s2a,5fq9,1y2k,6bu3,3tvl,2b1q,5ntq,2wmr,5lrg,3nin,6gnp,3f
l9,1i00,1w84,4rsk,4xya,5ivn,1a4m,5fnf,3sw9,4q06,6aah,2c4f,5nk3,5c91,4f0c,3
udn,5moo,2eep,6boe,2wf1,4bh3,4bxk,3pty,4lrh,5cs3,5u8c,3n1c,4rj5,5vo6,5qa9,
4eu3,2w1h,4pte,3nth,2xfi,4eb9,5jzn,5dxu,5ekm,4rxd,6bqg,1ikw,4gw1,2bdf,3eyu
,1kl5,4tkf,4jc1,3oag,5kmf,1etz,4cpw,1ce5,2w0d,3ifp,3pax,1way,3orn,2zv9,3vv
8,4ikr,1uym,2zb0,1uz4,6fdu,3rlp,2fmb,4lsj,3uig,1ppw,3kyf,4mpc,2llo,2p93,3k
ig,3std,3mxe,5ddc,6fhk,1cs4,3g4i,2h5a,2vtp,3sdk,3p3g,4kin,5g3m,2o3p,5ml4,2
azc,5t28,3qqk,4fil,3d67,2loz,4jls,1bdq,5acy,4eh8,2evl,3iqg,5eyk,3atk,2wfg,
5vlr,2qv7,2zpk,4qw4,2kmx,5njx,2hh5,2vxa,2vwf,3i0r,4mw2,4zx8,4kxb,1tpw,2hrp
,3k4d,5kdf,1sps,1nvs,1t5a,2a29,2g97,2xfk,1bo5,4qjm,4r1y,4b05,4z7f,3mke,5it
f,1gj7,4hbp,4tt2,2h2h,6cq4,4tn4,4fbx,3pwk,4oc3,4jmu,5khm,4oym,5yc1,3n49,3b
cs,5w85,3rlb,2vcq,5ul5,5ws3,3zj6,2ftd,3pkb,4q7v,4gq6,1d2e,5hkh,5nze,4qr4,5
ohj,5etb,5gjg,3vdc,3q3t,1fkb,2bw7,6h9b,3h0s,2jdt,3zep,3ng4,6ekn,4r76,5gsa,
4qz6,1oay,6ht8,4d8z,3efk,4l1a,5fqt,4cwr,3h0y,5w73,4uyf,1w4p,3roc,4aom,4uzd
,2x91,1hxb,4mul,5uwm,2c4w,3d1e,4ztm,6h34,1i43,4e9u,6g3v,4bco,3nuy,1kmy,5bn
s,5qb0,4glw,2jdl,5akl,3b92,2wks,3qcy,2i4t,6b4n,4j8s,6dug,3hv8,2x2r,1bkm,2b
yp,4a23,1zhl,4kip,4n7j,3wns,5tq6,4zek,3q3b,5m4c,8gpb,2gfk,2ay3,4mi3,6e8k,6
cwh,5eek,3g7l,5ewa,5kat,4iqu,5ogl,6cvw,4o2a,1bp0,4b3c,2zu4,4wup,3waw,2ci9,
3otx,1h60,5fe7,1gyy,5nu1,4e8w,3at1,1erq,5m3a,4ajw,2c93,3ppm,3hcm,4o13,1mai
,3usx,3txo,3l5b,2ohs,2xsb,1hwr,5v8p,3k5d,4nud,3bqn,5fhm,2xwe,2igv,4ish,5e2

p,4az6,1upk,6g98,4uwg,2ke1,5flp,4x48,3k37,5zh4,5twh,3chf,3cgy,2ork,4n6g,5v
41,1rbp,5nbw,2pmn,5ehv,2qt5,5edl,4og5,4tk2,2oqi,1zz3,4c68,2mip,2w1g,5nvy,5
dex,6e4a,3cyy,4akn,4g31,1k1y,1ebw,3nik,5aaa,6iiv,1fcy,6cee,3hrf,5afm,5kw2,
3fcf,2ym4,1v2s,6d5h,4v1f,3v5l,5f08,5dtq,5orr,1xnz,1hvr,5hes,3ft3,5fas,4zs0
,2xow,4cu7,6iiu,5yji,3pm1,4lkl,2iku,1d4p,4oq3,3tmk,5vrl,3r21,1uys,2rcx,5jj
m,2er0,1dwc,6h5x,4ps0,5ula,3ejq,2q96,1vot,1fsy,3vw9,2wlz,6c7w,6dud,1a9q,4o
5g,3qwc,5fun,1zm7,4qaa,1ciz,2j2u,3m1s,2cnf,5tb6,4j73,2nsx,1mnc,3ivv,4ew3,5
ele,2qu3,2xtn,3d1x,3g34,1x7q,5wbp,6f08,2f94,4trz,4qew,3lpr,2pjb,3h5s,4ibj,
5n8b,6fnr,1evh,5nk4,3ixk,4i6h,2fzz,5y8y,6mu3,2y6s,6afj,3o1e,3esj,2qfo,2zyb
,5d3l,3bi6,4eh6,1mf4,6mx3,2vj9,2iw9,5ody,5c11,6h7m,4x1s,5wbz,3qxc,4j7i,2bk
z,4zjr,4l34,2vwo,4yrc,1vjy,3eyd,4u5u,3bmn,5jar,5l6i,4gne,1u59,4qme,1r6n,3t
ct,3zrc,4dro,2p33,4c1y,3zlv,1i8z,1i5d,1ai7,4ptg,2x3t,1t69,4cik,2uwp,4c1d,1
sqt,3at3,2cfd,3cj2,4tv3,4l33,5exm,4p72,4rfz,4zsg,4oz3,5ny1,4aze,5v9p,5n1v,
1d6n,5w7j,3h91,2r58,1d7j,4j3u,3hyf,5mxq,2qp6,3my5,4ee0,5e1s,3gen,1d5j,3lj3
,3k8c,5ioz,3w33,5m2q,4u0u,1qbu,1h28,5dlz,1d8m,5nkn,1eve,6gu3,4hvh,3lik,2vm
c,5f3e,3upz,4mw7,3iww,4ckr,2adu,4yxu,5f4l,4clj,1fpc,5eud,5fow,2jfh,3uwo,5d
tm,2pe0,5tbp,1pu7,2x9e,5le1,4l6s,3chc,2f89,5m25,4ib5,2w6n,3l4y,3ccz,6bu1,4
dei,5f25,5wkf,4ab8,4yth,3oxi,5aqp,4ln2,3f7b,4r5x,4yoj,3n0n,5izl,5mnx,4p4e,
2xk9,1efi,4fnn,3ohh,2y9g,4g17,3ckb,4k3h,4ymx,1ywh,1mrn,5opb,6db3,1g6g,5aan
,2yi7,5os0,2jbk,3g6z,5h9p,5ecv,2df6,4jal,2w6z,4nw2,4qrh,2p4j,4i7f,5iez,2c8
w,2ay8,3ika,4o0j,4a6c,4pnn,1lrt,5tkk,2b55,4f08,4ac3,5aom,2p09,5v2p,3cde,3f
i2,4hdb,4alw,4ojr,2c6k,5eef,5agu,4uce,4a7i,2h23,3llm,5hk9,4lke,3oot,2vvc,4
e8z,3as0,2jdv,1o38,1g49,1ndy,5a46,4rj3,3zmq,2o9i,5e6o,2xf0,4os6,4bdf,3wmc,
4oz2,5qak,2x6y,3l7d,2oqv,2q95,2o9k,4ynb,5my8,3pz1,4iic,1bkj,3igg,2f9k,1elc
,6db4,3eql,2aux,3kd7,3so6,1oq5,4nw6,3upi,2xnn,3gk2,3vf9,2gg5,5f9b,4eg7,4fm
7,4wrs,1o4i,4fgx,2vnf,4jzf,3r5j,4d62,5tgc,3vh9,1cka,2bal,2z5s,3djo,2xch,4m
5k,6bj2,1ba8,2w68,1ypj,5iy4,4kcx,5eqp,1akr,1rtl,1cp6,1hdt,3gcv,5dhq,1iiq,2
rg6,4i9i,5kad,3hvj,5ckr,4nbn,3mz6,4nka,2exc,4nh7,3r42,1o5m,2y4k,5ivy,2y81,
2l8r,4xct,1lnm,1r10,5tt8,4nrb,4r5g,6c0r,5dgz,5l2n,5eay,5n17,3ozp,1ao8,1i9p
,4jn4,3fyz,3eju,3er5,6fui,3ikc,5nzq,5agr,3hzk,3k2f,3c52,3rxa,5f1v,4o78,4ah
r,4x6i,4qr3,5wex,1tbz,6mj7,3l6f,1lqd,3h0w,1ax0,3aya,2itt,1c1r,2r3k,4j8b,4o
15,3wgw,1jaq,5vt4,2fm2,2alv,5n4s,3pz4,4urm,1ax1,3c2f,1g53,4o28,3owk,2a4m,1
h8y,1vwn,1k6c,4dru,4ycu,2rk7,6co4,2bow,5mqy,4bfd,4ocp,5oh2,5svi,2wi3,5ndd,
1bl7,4daw,2zhd,1c87,2zq2,1css,4hra,4lp9,6q74,4j08,4lwt,2obo,3s8n,6cyc,3ogp
,5fqs,6gu2,5npr,2j9h,4l6q,4qwk,2cex,4ogt,1oiy,1rpf,4zk5,3ljo,5jo0,1ld8,5el
2,4mt9,1cnw,4uiu,1xge,4j8r,1ua4,1ezf,2feq,5vsj,4g0c,1rek,5bw4,1cil,4hvi,4h
zz,2fu8,4y24,4tk0,4qxq,2r8q,5x74,6as6,4yx4,1gar,5wir,2ms4,5cks,2xp8,5e1d,3
mo8,2pyn,5v49,1xk9,4qy8,5c4u,1gzv,5ovr,2hvx,5knx,2c9b,5mnn,6f3d,2wtx,4cfl,
4i9r,1p06,3lqj,3dba,4rwj,4d0w,2c92,4f6w,3evf,4iu1,1h2t,4itj,5eak,5hm3,5uwi
,4c4j,6cef,4cd0,4os7,3hv7,3kcf,4yb7,2xde,2k46,4i9o,3ken,3oqk,4k5o,4zyx,4in
b,4g8o,3kvx,5hi7,3nhi,1j80,1iew,3lrh,4kab,4e4x,1gpy,2r1x,5bpe,4tpk,1det,4k
ln,3jq7,1yvx,3dcs,3ovx,4xe1,4dcy,3b4f,4rcg,5i58,5apj,1qbt,5v8j,3bl9,4pyn,4
ot6,5ti0,5vsb,5tob,5t1l,3oqf,5ega,4x6s,4giu,5j20,5ho7,5ylu,5am5,2aox,3pka,
3t6y,5v6u,3vhv,6ggn,5qim,4j93,3hfv,3mzc,4oru,6e2m,5y6d,3l8x,4hhy,3wti,1xug
,6bhe,4pnk,4bti,1fjs,3mxs,5csd,5l99,4gw8,2qln,6e05,3lzu,5m44,4rio,4ydg,4kc
g,4rwk,4ui8,3zq9,4ibm,2z5t,3hdn,1rmz,3e9h,3rah,6f34,2o1v,2bed,4ofl,1rxp,2r
3n,5nut,2q80,1fvt,3uoh,1y0l,5hz9,2g2r,4ie4,6dh0,5e2n,4mrz,3vjl,4zyi,1shc,4
lj8,4ke1,3o6l,2hb9,5gmh,6a6w,5arf,4agd,3inf,2r23,4yt6,3cm2,3oyq,4fk7,6cq5,
5kbr,4mp7,5idn,3brn,3r04,3zrm,4n07,2amt,4yht,5l8t,4r3b,4ch8,5lvl,3s73,1zpb
,5o1s,1v0n,5v8q,3m9f,2xhs,4anx,4gly,4k1e,6ee2,3ud9,4pio,4ytc,3ekv,1gjc,4ii
f,2hiw,3ta1,4zba,1lt5,1w22,2j95,5ax9,4acu,1ek2,5epy,5clm,5vom,2gh9,2bgr,4j
rv,5m7t,6cdp,2h2d,2gdo,5ly1,5u8a,4trw,5vdt,2b4m,4d2p,3ta0,4tte,6f6r,3hp9,3
eos,4qye,4prb,5jox,3as2,1xr8,2vie,2jkr,5q0w,5auz,1oy7,3rm4,4dfb,4ngp,3n4b,
3wgg,3lhg,3dgq,6bfd,1q4k,2wou,5lyx,5lgu,1o3d,5i38,4kon,3ful,3krw,4mhs,5lvx

,4x0f,3umo,2zdx,3bkl,3qts,2osc,2fjm,3hfz,4bqh,1c5y,4q08,4r6v,5dv2,1tet,5f9
5,2xj1,3pb7,3mt7,4ju3,6b4h,5v4b,2fhy,1ppx,5eij,2x96,4unq,4odf,4gqq,5tya,3z
ya,4oee,6h8s,5fl1,5h13,3e6y,4yqv,5kcb,2uyi,2xn7,4zx4,5o1d,4n6h,4mnw,5x4n,3
qgw,5i8g,5iug,4ucr,4lkk,3hig,1uyh,1zyr,2z94,6fs0,6ekd,6gg4,1o2x,3jzj,5d26,
4jlg,2kce,1c83,4du8,4rvm,4r74,1os0,4tkh,5oci,4jyc,3dxh,4kow,5yjb,1iht,1sm2
,1nhu,3qtf,4x11,4ufe,4xy8,5ke0,1tlo,6f1n,5d3n,1kv5,3ual,1jij,5al5,3v3v,3fr
z,3c39,4dsy,3eq9,4hdc,2ym7,3sv9,1ex8,5tzw,4zw8,1hi5,3ro0,1d8e,4q6e,3jy9,5h
3q,4idn,5h8b,4a9s,1z1h,2zxa,1rw8,4do5,4er4,6fh6,3fqh,5os8,3m35,1odi,2j9l,5
sz9,4frj,3avn,5uzj,3k05,3ieo,5jic,4kza,2wic,4zx6,2hdq,5j9f,1tuf,2ony,4zfi,
4pul,3v51,4nj9,6g33,5fqc,1hee,4ayu,4wbg,4mi9,5vad,3nf8,1ogx,2n9e,5ox5,6dai
,4kn0,3atm,3anr,4yt7,4m0r,3ivx,5gvk,4ybi,2wap,3fh7,3u1i,2uxi,4rxa,5w7i,4tl
r,3hky,4fgy,1mrw,4asj,4dht,4dho,3wt5,2nqi,4zji,4gxl,4x7n,3bc4,4f8h,2ayp,3p
ke,1fch,4cmt,5dhu,1fgi,2zis,5hmy,1w82,2aj8,3uh4,1e3v,4g0y,1azl,2b7a,4psb,1
nw7,1w7x,3haw,1g6r,3lq8,3kf4,3eqr,4e1m,3wk9,4f49,2y6o,1a42,5mon,4m6p,2p3a,
6bix,2vv9,5unj,4ndu,5nfa,4nzn,5ewk,5kez,5ei6,5e1b,4frs,3t82,3pgl,3ov1,5nhj
,2j75,4omc,4o71,3vb6,5alz,4pri,5eh0,4gk4,1r5g,6hu2,1ws5,5ap6,3aas,1iem,5o0
b,4w52,1il9,4u6y,3uxm,5klp,3u3u,4tww,3ole,2wxo,3mwu,3cp9,3t2v,3iiy,1pxp,2i
4z,5i80,5igq,1y3w,1tsv,4bi0,5i12,5kj0,3suv,2f2h,4dtk,5ihh,1o4k,4az5,1ayu,3
zc5,1ljt,1hpv,2opy,2b1i,4zun,4gu6,4pge,5lvf,5av0,1apv,4tmr,2xix,5lsg,5bvd,
3nu3,3u4h,3bpr,6fyv,4ad6,3v7t,3tww,4mk8,1f8a,5h7g,3u8m,4y2y,4xwk,3r4o,2m0u
,3anq,1rwx,2r64,3dx0,2oji,5i3y,5evz,5kqf,4qq5,5unh,4lkm,3ur0,5ufo,3r9n,6af
e,2nmb,5g5f,3ekt,3arg,1fkn,3nan,2y4m,4o09,1o2h,4ob2,5q0p,3l4t,4r3w,3fw4,4m
dn,3rwh,4p7m,2iko,6eij,2xp4,5yv5,6eyb,4riu,2khh,2h21,6b1w,5vjp,4csj,1k21,4
mr6,3nuo,3hw1,3sr4,3wd1,1w1t,6hu3,1bxo,4o2b,2uwo,2bqw,4djo,4bs0,5iaw,4poh,
5xvf,5lp6,5e8a,5thi,4a9m,5o9y,3t3h,3zjc,5wf5,5ja0,3b2t,3rv8,5m34,5jal,1g7p
,5wa7,1ivp,5efb,2gvd,2gg9,1s64,5q1a,5xo7,2lko,5lsh,6cer,4fny,2wly,4wr7,3rx
i,3kr0,4e34,4qmp,5i56,4bek,2rfn,6en4,4x8g,5tt7,4rab,6bkx,4uva,3hww,1pq6,4x
it,5joh,4lhv,2n8t,3iof,3h2o,2dbl,4p1r,4amx,6d1m,3ad7,4uxl,2gvj,4j4v,4avg,1
kuk,3m53,5djp,2wmx,5d0j,2i4p,5n2z,6df7,5nz2,2rka,2ww0,1br6,3po6,2uue,4dvi,
3u2k,3b24,4i9c,1nd5,5orh,2f35,4uvx,2wor,3zhz,3qd4,1inc,3oku,4qf7,4qvw,3rwj
,3gss,1lkl,3hau,5byz,1u65,2ycr,5ia1,4m2w,3szb,4brx,4nxo,1bdr,4y2x,2evc,5du
4,1h4n,5iis,5a6k,4fe6,5wef,5g2b,4w53,2bt9,6ary,1mq1,2iuz,2qnp,1hxk,5j6s,6c
e2,1ryh,1bmn,2zju,3zcw,1f2p,1o2g,5qb2,3kqe,5yg2,5uah,3l3q,3udd,4y4j,4poj,3
ekw,5hv1,5y3o,3hnz,5nf9,5ekh,3vw6,3mxr,3o64,1xxi,5ih9,5fbo,3rpr,2y56,3bea,
5t2l,5g43,3ilq,3hkt,5kdr,3dbu,4rhy,3udr,5jbi,6d1l,5g1p,3i3d,4j48,4qgg,2e93
,4lpb,5tq7,1prl,3ara,4ksq,4m1d,4kif,4xhl,5n8t,4bao,4bgg,3bl2,5om9,3h2m,4gb
9,1o4n,2h03,3qfz,3hxb,3wd9,2onc,4u7q,6g46,5ai0,5tqe,4zy5,4p4b,4kiw,3kgq,6f
20,2vk6,2iv9,5uex,5myr,3sad,3p8o,2nn7,4hwb,3dya,2p99,5h4j,1fj4,6ej4,1ch8,3
jrx,3i73,5nqe,3as1,4o3t,2i4j,4pp3,2itp,5wg4,3uzp,2cf9,3ion,2bys,4awg,5law,
1upf,4ec4,4ibg,4rx9,6bef,3unk,1k4h,5csp,5kly,6gfs,5d4a,1zpc,1zuc,6ek3,5eb2
,2w78,3o99,3n51,6g6y,3hu1,4nnr,4gpk,1jpl,4yzm,3wyk,5qax,2frd,6g36,3u81,3cp
c,3t0t,4wuy,3iw4,2ozr,1v0m,1l5r,3mo5,2vtr,3u0p,5niy,6ej3,6hdn,5iop,3t09,3z
k6,4bsq,3mqf,4u73,4kyk,5kxi,1zaj,4j1c,1ntk,1ol2,4q1f,3r8u,3ked,5j7q,5eq0,1
ppc,4x2i,4egk,5ghv,5ovc,4q9z,3lxl,4hze,5efh,4lwu,5nwb,3twx,3evc,1uk1,2wxg,
2zm3,2mpm,5vcv,4k8o,3vv7,2n27,2zg1,3gfw,3krl,3bf1,2cej,2nta,5tig,1o4o,4s1g
,1uj0,3f33,3hec,4pfu,1k2i,5ahj,2zit,2xng,5uoy,5y8z,1f5l,1ke0,2i6a,3sap,3as
k,5tl9,3ime,4zw5,5tbn,1dm2,2vr0,1g32,5k51,2bgd,1clu,4o0z,4z68,4mnq,3ue4,5c
fa,2vto,5owt,5wzt,2pvh,4gui,1o5c,5sy3,5ea3,4c72,3gjt,5n9k,4jxv,4uu8,3k3h,1
lek,2qwc,3pcu,2ohl,3ioi,3n6k,2oht,3gw5,5a09,3nga,1waw,4wcf,5g1c,4li6,3c4c,
4mk5,5qao,3ksq,2f4j,5hcl,4bde,6egs,1utt,4g8l,2ez7,3zlx,2wqb,1ntv,4nzm,2jh0
,5e2q,3zlw,2yc5,4gmy,4oo9,1enu,4dq2,6ecz,4b4m,4jfi,2f6y,2hs1,2a4g,5e3a,4qf
r,3fmz,4h1e,1rww,3ccc,5h63,6f28,4v27,3mxc,1gbt,1hkk,2a4q,5x33,2veu,2hmw,3m
ea,5mmn,4ep2,2v5a,5adt,5b5f,5lj2,1b74,5v82,2bdl,3b8z,4gye,4ux9,4xv2,1v2u,3
c4e,1sj0,3ohi,4u79,2oyk,3fnu,3qcf,1ie9,2yay,2ggx,2zfp,4nks,1o2o,6f4n,2gj5,

5b4w,4jok,4w9f,2qnz,5his,1pxo,1d4i,3npa,1h5u,4w9s,3cid,1jiz,5w8i,4y8d,3atl
,1nju,5f61,2i4v,4n8d,2qrg,5uyu,5qan,4cpt,2g24,2xrw,5a2j,4jbp,1pip,4whr,5i2
z,3d8y,3dow,2r59,4ux6,4emf,1b8y,3d7z,3vgc,3hvg,4uxb,4nym,6cz4,2x8i,4kb8,1b
n1,4x3h,5ork,3arr,5wg3,5v9t,4qz0,5cnj,4u03,3ti8,5htl,1xm4,5lyw,3s78,3l9l,4
u90,2boj,4oc1,1rgl,3bti,5eh7,5lqf,1iup,3zmv,3sfh,5jlz,5zun,6dh7,2bet,1ewj,
1tyn,1pbk,4lzr,3i06,1rp7,4axm,4b9z,3wth,3q32,2jiw,5n0e,4ele,4y85,5ncq,1k9s
,1ugx,5hn0,3i7b,4qev,1bty,4wht,4uvw,3i4a,6iin,5acb,1ag9,3h9k,2fm5,3npc,4ql
q,2r2m,2yak,3rbm,5nxq,3s3i,4d2v,3bqc,6ee6,3vzg,1urg,6f2n,2ael,2z4o,1wun,4k
o8,2zxg,1o2u,1ung,5mn1,3q4k,2ly0,5nt0,3mj5,4cxw,5mo2,4mho,2h9t,4k4j,5m0d,5
hzx,2q64,1axz,1nlp,5ak0,1h79,4xsz,2z3h,5ez0,1iwq,3wf5,4aig,4pft,4pni,3wf6,
2cji,4euv,4ce2,1bqo,1wss,2x09,6hrq,4u44,2nmy,2aeb,3b7i,2wti,4eo4,4z7n,4ovf
,1sqi,3is9,4k5m,3kqt,4elg,5osl,5cdh,2d2v,2h2j,2w0z,3t3c,3kb7,4ie0,2w26,6ma
5,5j6m,5e91,5ncy,4n5g,4mx0,1rth,3bmq,5mwo,4h3j,2bvr,5d47,2it4,3l59,5eec,3r
5t,5ia5,5orl,5q0h,3fej,5nne,1u33,3elc,2az8,4cs9,3u9c,3e64,4qyg,5ei4,1aaq,4
ymh,4mu7,5q0n,3o9b,2hvc,3qbh,5aph,2qd8,5tdw,4bqx,1dtq,1zd4,5sve,3bv2,5hed,
3arb,5xzr,4h38,1v2h,3pd2,3fuk,5ur6,6dkg,4iz0,2hr6,3oe8,1k1l,1o4d,5u0f,2bak
,5n9s,5vnb,1jyi,3cwj,2e2p,4rwl,2ccc,3fqs,5i3a,5uln,6b1f,5h0b,4len,4p73,4ab
j,2ccu,4loq,2rok,1lev,3ama,5vsd,5w13,3n5e,5o0s,4rse,5km0,3lkh,6en6,2xln,2y
fx,5e73,1s9v,5ugb,3d1y,5sy2,1t7d,2rf2,2rt5,2f9b,6fcl,1wuq,5i0b,4hp0,5tkt,3
kc3,5xaf,6ew6,5hhx,3nu6,2ym3,6asu,5t70,6b7c,5ea6,5a9u,2h4n,3lea,3zv7,3a3y,
2qrq,6f6u,6bgw,1r1j,5mny,4urk,5yco,5uv2,3pn4,3spk,4ycn,6axq,5q0o,1j14,5j8m
,6dq5,3qxp,3ogq,3k97,1shd,6fii,4x6y,2cgx,1db5,4wrb,5w0f,1bxl,4ura,5dtw,2bo
k,1xr9,1m0b,2rkd,2xeg,4bj9,1w6h,3qcq,5a0e,5owc,6fyp,4o3f,5dpw,4yve,4j8m,2c
ht,2rgu,5fat,2qn3,4i80,2hny,3wtk,5nzm,3kjd,2o9j,4oz1,2weo,5a4t,4fut,4pra,4
i4f,1sts,1uu7,4y67,4kz4,5bve,4gcj,6cqz,5c87,3uih,3k23,2j7w,4oc2,3h98,4mrf,
3ip5,4aa5,1klg,4dk5,3avb,4kpz,4ztr,4bzn,3b7u,4elb,5kit,5etr,5dv4,2vgp,5wcm
,4or4,4xbd,4lp0,1lee,4jjs,2zx7,4bks,5yea,1urc,1wdy,1mue,3eq8,2y57,3v31,3ri
l,4jk5,2yjx,5vqt,5nwc,6fuj,3ljg,4x7l,1d3p,3jqa,5qcl,5q0j,5w0q,3bel,5f4r,5n
vh,4qrc,4yih,4zsp,6gjm,3iok,5nin,1exw,3cj3,1kzk,5t78,2v59,3nzx,4pns,4m7c,6
gi6,2o3z,4b5t,5u98,4bjc,5i9x,1riw,4kz7,6f5m,5izq,5jmx,1rlq,3r2f,5u7i,4r73,
4x6o,5ee7,6f8g,3g2h,3dkg,5eni,5uxn,5iq6,2k00,5uwp,5cpr,3qfy,2r9x,6cbg,6bgv
,4nk9,2vuk,5nkb,4nie,2c69,3hvc,5wj6,5w7u,5owl,5bms,4zg9,2qi6,6b4l,3iw6,4k5
y,4u3f,4i10,5v7t,5htz,1jk7,2zsc,3gst,1pfu,5z95,4nhy,1z6p,4qmo,5orj,4wa9,3r
7r,2fjn,5lxp,3er3,4m2r,4onf,3vfb,2pj7,4m0f,3m54,4abb,2m3z,2pks,3kr4,5v7w,4
fzj,3wk7,2y77,4p0n,4jaz,6cyd,4xg3,4mka,4l0s,4awf,5ew9,3m40,3fy0,6bbu,4pdk,
188l,4ca8,4a4e,5ku9,5a6x,4lww,3qqu,5q0d,3sji,5vgy,5f4u,3msc,3mrv,3fu3,2anl
,5mty,2jaj,1jyq,5edr,5fpk,4wyp,2exm,2viy,2rku,4r92,4qyo,1bnv,4ge2,3kqa,3px
q,1mzc,4e9c,3iae,2uze,3agm,4dbn,4v25,5wfc,4lph,4ryd,4na9,4rux,2qpu,3ay0,6r
sa,2fx8,1xn0,3wsy,1o0f,4fe9,2aou,4mr3,4frk,3vbq,3v01,2v6n,4z89,1gnm,3rqe,5
awj,2m0o,2gg8,3zdh,1ga9,6cjw,1qca,4o2c,4e6c,2pem,2ykb,3r9d,4ajn,4p0x,4k7i,
2ghg,1ogz,1v2l,1ni1,2wib,4ncn,4gee,1wkm,3zmj,4ntj,3nok,2vwy,3i5z,1w2g,2onb
,4ufz,2ogy,4qps,2l75,4x60,2ayr,4nan,4alg,3t4h,4de7,3hxd,3fpd,2p94,1yt7,3vb
y,1xkk,4gr8,1bm7,4yyt,4ca7,1ph0,5vd0,1qf0,5hzn,1w5v,3hkq,2v5x,3zs0,4b0j,3c
oj,3d78,4cki,4l7g,3wd2,5y5t,6cmr,5xof,2q92,1uz8,3in3,4y2b,1qb1,3al3,2xzw,5
tzg,5vqv,5xxk,4ckj,3bpc,3lnz,2w05,4hnc,4eeh,5ar5,5awu,3qx9,3zyb,3dnj,4mnx,
6hu0,2gvf,4bdi,3sz1,1wqv,1o86,5uxf,2vvt,4mb9,5hwu,5h5f,1utz,4r93,5jf8,5f60
,4nld,2rkf,3v7d,3g35,1o7o,5mtx,5lxc,4mqp,2j9a,5vzu,3vsx,2qqs,2ybs,5yql,5ho
r,4ir5,1rdl,2ygf,6hf5,2g78,4ajo,1db4,4qwx,4abk,5xo2,5op2,3bls,5u11,2xaq,3b
l1,5w38,1j15,4o72,2oei,4gj7,2gnh,3m3o,5ls6,5c2o,2xp6,4igq,6df1,3gzn,5uac,4
x63,1yvf,5ceh,3h1x,4ksy,4nh8,2fsa,2jkm,5tx3,3rwe,4e28,1a46,5all,5ddb,5boj,
5kx8,3pdq,5ivv,5vb5,4qw0,5laz,1zd2,1sz0,5a5q,2j9n,5ml0,5wqd,2uzb,1pu8,5ar4
,3mho,3p3u,4k76,4y94,3qpp,5wa8,3dy6,2cgu,4xub,2qhy,3ard,2w6o,4oem,5aac,4kz
8,5ljj,6epz,3o88,1kz8,1ykr,5tfx,3g58,2va7,4nk3,2b54,1qnh,4rvt,4xtz,4z1j,4o
ex,2hu6,4b71,3ryy,6d6u,4d2t,4xu2,2zg3,1oba,1tu6,5myv,1sri,3dne,2gbg,5msb,6

cea,3pfp,4x8u,3u0d,4e6d,6b8u,2f7o,3cjf,6g07,6dgx,2q72,4ikn,1o2y,2yb0,4oow,
1lhf,4es0,3sn7,2iqg,4lwc,1a09,5boy,5zwi,2pj9,6hoy,5mg2,3tlh,1xbb,3chp,4hej
,5kma,2wf0,2yoj,5f91,5khi,1g9c,1mto,4xyc,6c7i,3tu7,4iuu,5q0y,4qmu,4bky,5du
s,4cpx,5urk,4wx6,1r78,3efj,3r00,5icx,1qvu,5mby,2a5b,6f90,3ppp,3pd3,5f0c,3b
t9,2jc0,5ose,6h36,1htf,3t3g,2c6e,4uiy,5xgl,5t31,1zfk,4dpt,4mzo,5eto,2q93,4
x6x,4o24,3ds9,1lfo,1dy4,3wav,2g94,4q83,3bxf,5ali,4lc7,1v79,3ocb,2ctc,4qbm,
3kfn,2jq9,4jff,5oha,3i4b,3shj,6m8w,3umw,1hyz,3aau,5ijj,5ni0,4ea1,3o1d,1hms
,3cwe,4jjq,3lpi,5llp,4hws,4ygf,2d1x,5uuu,4tmf,6alz,3iqh,4bib,4bhi,2weh,1to
w,5umz,3n23,5ylv,3e12,1tfq,1hk3,5yqo,5nxg,1w2h,4u6c,4a9t,6eo8,1o0d,5gjd,6c
f6,5t6g,2vot,3hy7,5e2m,3sl4,5ngz,5mrd,4xm6,3shz,6eeo,5unf,4exh,5c6v,1e1x,2
brg,5tcc,5ad1,3jwq,3b3s,5ale,3ig1,6au5,2jh5,4psq,5g3n,4dt6,5d9k,2x52,3v3m,
6fn9,5d6e,1gsf,4wp7,1kzn,1fm9,4mjr,3m93,2z4z,1w14,6et8,4af3,1oxn,4rac,3fu6
,1oyn,4yoi,2vx0,4oef,6bnk,4k4f,4w9k,5nxi,5h8x,5f0h,4w4z,4ran,4ke0,5kjk,3qt
6,3ibn,2ima,3rkz,3me9,5ccl,3uat,2rfy,3fuj,1sl3,3sz9,5ab9,1f1j,2p3i,5a4c,2w
0s,4gkh,5obj,5tqf,4yec,2nng,4fea,4z0f,5wo4,5h19,3hng,1jt1,5fqp,2pql,4obz,1
b6l,4ael,4f6v,1sdu,4pid,4twy,5bvw,5fxs,4ph4,2aov,2h8h,1uj6,4k8a,2uxu,1zhy,
1unl,2pj8,3lgl,3q8d,3dx4,4x3e,1o35,4ztq,5am3,4jwk,1hww,4cd4,4qw1,1lvk,4e3j
,1ff1,2azm,3gqz,1bq4,5ajy,2pe2,4mhz,5jhk,1urw,2rib,4o61,4ory,1qm4,4i5m,1q6
5,4x6m,4okp,4jje,5kks,4pl3,1xws,2v0n,5huy,2ohp,4e5i,6b5q,5hip,4q7p,5c6o,3o
0j,4x8v,4a1w,2gbf,6mu1,5tdi,4zeb,2fw3,5zz4,5wbk,4bw4,4mz6,6b96,2m0v,1fwe,4
mxc,5iub,3ffp,3qx5,4eev,4yz5,3oaf,4zy2,5qik,2uy4,6ma3,5om7,2p98,3zrk,2cn8,
2qch,4efs,6cct,2wwj,4dij,3nes,6e4t,4pde,5knr,3el1,4nwd,2vle,3tcp,2v12,4ge5
,4xhv,4kil,4apo,3nwb,3n0h,3g5d,5m7u,1au0,3fxz,4qo4,5vcw,4b0g,2avq,4o75,4g9
c,5j5d,4el9,5vnd,5m53,3pa8,3hav,4j1p,2uy3,5a6h,1u0h,4wyo,5knu,3sw8,3py0,3h
yg,5dj5,3nuj,2gl0,2ojf,2gfj,5l44,3qd3,2iit,6b16,3zsw,1sv3,1q91,3c14,5d7e,6
h7o,6cj1,5vsk,3avi,3tv7,3bc3,5ick,4why,5agt,4usi,4mbc,4z8m,4pgh,1b8o,4dcx,
5m7m,4fmo,5moe,6bvb,3b7r,6gwr,2be2,5cgj,1pzp,3lpt,5ai9,3p7i,5etn,5mli,4wag
,5znp,5ufr,5b56,2yk9,5i5z,5nk6,4qsk,3hv3,3qs1,4mm6,3ujd,5g2g,4us4,4c7t,5c1
w,5uis,3az9,4lj5,3rzb,6c98,3bgp,2w1f,5cyi,3tzm,3zns,3qrk,3uhm,6ehh,2lp8,5s
xn,3gk4,1y91,5nwz,2y34,5qck,4qmt,5j58,4q07,3sm2,2wp1,2jal,3kpv,2mji,4znx,4
yl3,4ju6,2ze1,5hjd,4n3w,2f3f,1anf,3bgm,6ffh,6f5h,2qbw,4n8r,3msl,3bsc,2yaz,
4nmo,3dsu,1w0x,6dcg,3wk4,3pup,1mq5,2foy,5uox,3qo3,5npb,4jze,4erk,4axd,1zsh
,3hxc,3ti6,7prc,4u5s,1kf6,1o2p,3vo3,4bhz,5bqs,4r4c,4i6f,5lli,4p00,6gzd,1jh
1,5wmt,1wu1,3kwz,5a5p,5ng9,6djc,3r0y,3f70,1fzq,4nct,4gu9,3cd7,4wop,3ens,6b
1c,5yib,3pz3,3hr1,6ft8,5t4u,5owh,4jft,4tki,3zo4,6gw1,1t32,5uwj,5fsc,5l6j,4
tzn,5kax,6ge0,5dcz,5mwh,4cst,3tzd,4qtd,1n3w,4x7k,1iep,4mk9,5myl,1yw2,4hrd,
4lnf,1b38,4byj,3qri,3u1y,6fmf,2x2l,4mzf,4z2h,4qvy,1ghw,2y1x,3wzp,4e8y,5ety
,1npw,4ha5,4ucc,4wt6,3ouh,4gzw,1br5,5ohi,4mny,5m6u,4k0o,4f20,6fjm,5v83,3qa
r,3tt0,4ax9,4cu1,3nxq,5ma7,4k9h,3ava,2x95,3k5c,4l7d,4xii,4ere,2ydi,3lfn,4y
2u,3e7b,3d4f,2avs,1x81,5usf,2h2e,3s72,4b7n,1meu,1ftk,2oc0,5ehq,5uc1,3obx,2
jko,1sre,3wkb,5wf7,5o9r,2qo8,1p04,4qy3,1zub,4kax,5d75,5lpk,1haa,5wvd,2fgu,
1bju,4bt9,3itu,6enx,4w9d,1gvk,4yqm,4rrq,4dhl,4ec0,3m89,3l7g,2ow9,3p8z,4bcq
,2wtw,5a54,1o4e,4hni,1njj,1rne,5e88,5arg,1lkx,1fkg,6bir,5xms,4wci,1e6q,5qa
i,2hdu,2wxn,3vjk,4ux4,4p44,5f1x,5eif,6afl,4b6u,1eou,3c94,2v85,3ans,2xc4,2n
p8,5cs5,2z4r,4a16,5hu0,5kz0,4cwp,1czl,5gmn,1o9k,2qu5,4e3b,5aqu,1ghv,2qwe,5
i7x,2x4t,4mw9,5am4,3f37,3fzy,5cbm,4h5e,5ucj,3ikg,2zym,4zh3,3p23,5fp0,3zos,
4z0q,4aa7,1aw1,1ssq,5v5e,5lny,1hy7,5w84,6fty,5v5d,2ioa,3lox,4e4n,2zwz,4ufh
,1gi4,2cen,1jao,4czs,6ccu,1xn3,2yiw,3ocz,5htb,4q19,4bdk,3pj1,4dbm,4k67,4gh
6,4j52,2o22,4ocq,3hwx,5t2b,3avm,3vp2,3su6,4ez3,6cdl,2vvu,6grp,2ea2,4d4d,1s
me,6g9a,2ves,4c3k,1okz,2f7i,3ri1,5lyr,3s3r,3osw,4zjj,4aoc,4gah,4y8c,3k5g,6
e2o,6f8x,1pyn,5akw,4wy3,5w9g,2w3o,5y8c,4yzu,4xkc,4qjw,6bmv,3d8w,2f7p,2hw2,
6gzl,4ocx,6e4w,1v16,2ym8,2zq1,1wdq,2w0x,6ht1,6bmi,5os3,4pvy,5dts,2zo3,4z3v
,5n3y,5na0,3p5k,1unh,2xg9,1kel,2qwf,5yhe,1i7c,4muk,5c85,1t5f,5ab0,5lm6,4j4
5,4wke,1owj,4oct,6bto,5knj,3dek,4hl5,4rqv,4yc8,2pg2,1wur,3cd0,4cp7,2oi2,3u

8h,5a2s,3nu4,4i74,4bgx,6fbw,5f37,2bv4,5mrp,3sdg,5oax,6hsh,3pvw,1dif,3u90,4
uvu,4klb,3vf3,4rpv,1gi7,5d12,6do4,6hd6,2xvd,1onp,5l6p,3sv6,4jda,3zmu,4rlt,
4kz3,2w97,3bh8,5v3y,3m55,5t8q,3max,5ipa,3s9i,6g93,1l2z,5nad,2usn,4tim,5l01
,1y3g,4d0x,4f7v,5cy9,5d24,2hhn,4e5g,1at5,5b4k,4n98,3l3z,4pku,3u5l,6apw,5mf
t,1u32,6hmx,3ft4,4lbl,4cpr,1m6p,5d7a,4pd9,2ofu,4od7,4ok6,3lzb,4pzh,6gxa,5n
ud,4inu,1gi6,4j5c,3t84,3p3j,4mwu,3ebh,4ytf,4l7c,4xh2,5ldk,1tcw,4pop,1vij,6
q6y,2xgm,4abh,1h00,5aqq,2r02,5fwa,1gvx,2fj0,4qw5,3zju,4aw5,5oxm,4wk2,4g34,
3ml2,1xs7,5ktu,3zlk,4aaw,2bq7,1ec3,1oko,2vqm,4mn3,4y2s,4o0b,3mwe,6cha,5iui
,5om2,2pvn,1xom,1c4y,3smq,5uqv,4bcf,5ftg,3kad,5i4o,5a4e,4pry,1c70,5nw0,3bz
e,4flk,1ibg,3fc1,4e3l,3lkj,3as3,5fqv,5vdv,4awk,4og7,6buu,2gu8,1ndz,4z22,5d
gw,5ou1,2qhn,2xb7,4ctj,3dc3,1pbq,3mhi,5qcm,2vwc,1vyq,5g60,6b31,3nht,2zv2,3
iej,3aaq,4yp8,5wuu,1f9e,3ml4,2v16,5zaf,4qb3,4flh,3ftv,5epl,5e4w,2b1p,2e92,
3t19,3kwb,5ngu,4xx4,4mgv,3tsz,4psx,5x9h,3f66,3nkk,3pe1,1o3h,2wed,3l9n,2f6z
,3p4r,5nw2,4xxh,2imd,5hyx,4cjq,1htg,5ttw,5d25,5uwk,4jbl,4d3h,2oq6,3vtd,2xp
2,3g8i,2vtd,5mxx,2uw5,2lo6,2r03,4jfm,3hk1,5elz,2vtq,1nfu,3aig,5n6s,4um9,5c
8n,1br8,5ly3,2w5g,2clf,5z68,2pze,1x8j,3drf,6elp,3tk2,3zn1,6gx3,1vea,6bod,4
c2v,1rt1,5ia0,1q1g,3bbb,5hda,5dy5,5q0m,3rvi,6iik,3o57,4f70,4k9y,3ksl,5c7d,
5eva,3qin,4fri,2bz6,4uw1,5k1f,1w2k,2m3o,5wbo,4np2,4mjp,6emh,5xpl,1f4x,1xhy
,6fr0,5ml3,4ie6,1d8f,5a5d,3kai,2wm0,6dge,6hpg,5uga,5tmp,5k0t,3sbh,1llb,4l7
0,4qq4,4c6v,1t49,4kc4,3wde,5gmm,5dah,2sfp,4hy1,6f4w,1a1c,1flr,3l1s,5m55,4d
i2,5emj,4ual,5djr,6ck3,3e16,2b52,1tom,3mo2,2xd6,4x3t,5m29,6fmi,1oqp,6cje,2
h4g,5qat,5kqd,6c1s,4ibi,4k3q,3ckt,4jh0,5nn0,3oyl,2zkj,3o86,1yda,4o0r,4r07,
4an9,4ear,3ad8,3lp2,5lay,5b2d,2piy,4bw1,4xqu,5vm6,4yuw,5fsl,5ajw,5tus,3lpg
,5llg,2vwl,3e2m,2toh,5vc4,2p8n,1zz1,5qaq,3n9s,4lh5,4tju,3thd,3km4,4yje,2rk
n,4bgm,3tvx,5fng,2v2v,1o49,5fi6,4ybs,1y8o,5cgv,5fqr,5mt0,4uoh,2x81,3m6f,5w
a1,3qn7,6ciy,5w4s,5kgw,4mw0,5fot,4fxf,4wwn,5a6n,4xy2,3nzu,3nc9,3m36,5wb3,1
gcz,2wi6,3mhw,3sow,1jbd,5lgq,1tmn,5j41,1cin,5t1s,2cct,5n58,1aid,2wzm,5aab,
4yqh,4e7r,1il4,5u48,5mkj,2uy5,5myk,3m96,5tzz,3c88,2ll7,1g9s,1is0

List S2. List of PDB IDs included in the training data of Interformer.

10gs,11gs,13gs,16pk,184l,185l,186l,187l,188l,1a07,1a08,1a09,1a0q,1a0t,1a1b
,1a1c,1a1e,1a28,1a2c,1a30,1a37,1a3e,1a42,1a46,1a4g,1a4h,1a4k,1a4m,1a4q,1a4
r,1a4w,1a50,1a52,1a5g,1a5h,1a5v,1a61,1a69,1a7c,1a7t,1a7x,1a85,1a86,1a8i,1a
8t,1a94,1a99,1a9m,1a9q,1a9u,1aaq,1abf,1abt,1acj,1ad8,1add,1adl,1ado,1af2,1
af6,1afk,1afl,1ag9,1agm,1agw,1aht,1ahx,1ahy,1ai4,1ai5,1ai6,1ai7,1aid,1aj6,
1aj7,1ajn,1ajp,1ajq,1ajv,1ajx,1akq,1akr,1akt,1aku,1akv,1akw,1al7,1al8,1alw
,1amk,1amn,1amw,1anf,1ao0,1ao8,1apb,1apv,1apw,1aq1,1aq7,1aqc,1aqi,1aqj,1at
5,1at6,1atl,1atr,1au0,1au2,1auj,1avd,1avn,1avp,1aw1,1awf,1awh,1awi,1ax0,1a
x1,1ax2,1axr,1axs,1axz,1ayu,1ayv,1aze,1azg,1azl,1azm,1azx,1b05,1b0f,1b0h,1
b11,1b1h,1b2h,1b2i,1b2m,1b32,1b38,1b39,1b3f,1b3g,1b3h,1b3l,1b40,1b42,1b46,
1b4d,1b4h,1b4z,1b51,1b52,1b55,1b56,1b57,1b58,1b5g,1b5h,1b5i,1b5j,1b6h,1b6j
,1b6k,1b6l,1b6m,1b6p,1b74,1b7h,1b8n,1b8o,1b8y,1b9j,1b9s,1b9t,1b9v,1ba8,1ba
i,1bap,1bb0,1bbz,1bcd,1bcj,1bcu,1bdl,1bdq,1bdr,1bgo,1bgq,1bhf,1bhx,1bil,1b
im,1bio,1biw,1bji,1bjr,1bju,1bjv,1bkj,1bkm,1bky,1bl4,1bl6,1bl7,1bm2,1bm6,1
bm7,1bma,1bmb,1bmk,1bmm,1bmn,1bmq,1bn1,1bn3,1bn4,1bnm,1bnn,1bnq,1bnt,1bnu,
1bnv,1bnw,1bo5,1bp0,1bq3,1bq4,1bqm,1bqn,1bqo,1br5,1br6,1br8,1bra,1bsk,1bt6
,1btn,1bty,1bug,1bux,1bv7,1bv9,1bwa,1bwb,1bwn,1bxl,1bxo,1bxq,1bxr,1bzc,1bz
f,1bzh,1bzj,1bzs,1bzy,1c12,1c1r,1c1u,1c1v,1c29,1c2t,1c3b,1c3e,1c3i,1c3r,1c
3x,1c4u,1c4v,1c4y,1c50,1c5c,1c5f,1c5n,1c5o,1c5p,1c5q,1c5s,1c5t,1c5x,1c5y,1
c5z,1c6y,1c70,1c7e,1c7f,1c83,1c84,1c85,1c86,1c87,1c88,1c8k,1c8l,1c8v,1c9d,
1ca8,1caq,1cbr,1cbx,1ce5,1cea,1ceb,1cet,1cgl,1ch8,1ci7,1cil,1cim,1cin,1ciz
,1cj1,1cka,1ckb,1clu,1cnw,1cnx,1cny,1cp6,1cpi,1cps,1cqp,1cr6,1cs4,1csh,1cs
i,1csr,1css,1ct8,1ctr,1ctt,1ctu,1cw2,1cwb,1cwc,1cx9,1cyn,1czc,1cze,1czk,1c
zl,1czo,1czq,1czr,1d04,1d09,1d1p,1d2e,1d2s,1d3d,1d3p,1d3q,1d3v,1d4h,1d4i,1
d4j,1d4k,1d4l,1d4p,1d4t,1d4w,1d4y,1d5j,1d5r,1d6n,1d6s,1d6v,1d6w,1d7i,1d7j,
1d7x,1d8e,1d8f,1d8m,1d9i,1dar,1db1,1db4,1db5,1dbb,1dbj,1dbk,1ddm,1det,1df8
,1dfo,1dg9,1dgm,1dhi,1dhj,1di8,1di9,1dif,1dis,1dkd,1dl7,1dm2,1dmb,1dmp,1dm
t,1doj,1dpu,1dqn,1dqx,1drj,1drk,1drv,1dth,1dtq,1dtt,1dub,1dud,1duv,1dva,1d
wc,1dwd,1dx6,1dxp,1dy4,1dzj,1dzk,1dzm,1dzp,1e00,1e02,1e03,1e06,1e1v,1e1x,1
e1y,1e2k,1e2l,1e34,1e37,1e3g,1e3v,1e4h,1e55,1e5a,1e5j,1e66,1e6q,1e6s,1e72,
1e8h,1e9h,1eas,1eat,1eb1,1eb2,1ebg,1ebw,1eby,1ebz,1ec0,1ec1,1ec2,1ec3,1ec9
,1ecq,1ecv,1eed,1eef,1eei,1efi,1efy,1egh,1eix,1ej4,1ejn,1ek1,1ek2,1ekb,1el
a,1elb,1elc,1eld,1ele,1elr,1els,1em6,1ent,1enu,1eoc,1eoj,1eol,1eou,1epo,1e
pp,1epq,1erb,1ero,1erq,1err,1esz,1ets,1ett,1etz,1eub,1eve,1evh,1ew8,1ew9,1
ewj,1ex8,1exv,1exw,1ez9,1ezf,1ezq,1f0q,1f0r,1f0s,1f0t,1f0u,1f1j,1f28,1f2o,
1f2p,1f3e,1f3j,1f40,1f47,1f4e,1f4f,1f4g,1f4x,1f4y,1f57,1f5k,1f5l,1f73,1f74
,1f7b,1f8a,1f8b,1f8c,1f8d,1f8e,1f90,1f92,1f9e,1f9g,1fao,1fax,1fbm,1fch,1fc
x,1fcy,1fcz,1fd0,1fd7,1fdq,1fe3,1ff1,1fgi,1fh7,1fh8,1fh9,1fhd,1fhr,1fig,1f
iv,1fj4,1fjs,1fkb,1fkf,1fkg,1fkh,1fki,1fkn,1fkw,1fl3,1fl6,1flm,1flr,1fls,1
fm9,1fmb,1fo0,1fo2,1fo3,1fpc,1fpi,1fpp,1fpy,1fq4,1fq5,1fq6,1fq7,1fq8,1fsg,
1fsw,1fsy,1ft4,1ft7,1fta,1ftj,1ftk,1ftl,1ftm,1fv0,1fv9,1fvt,1fvv,1fw0,1fwe
,1fwu,1fwv,1fyr,1fzj,1fzk,1fzm,1fzo,1fzq,1g05,1g1d,1g1e,1g27,1g2a,1g2k,1g2
l,1g2m,1g2o,1g30,1g32,1g35,1g36,1g37,1g3b,1g3c,1g3d,1g3e,1g3f,1g3m,1g42,1g
45,1g46,1g48,1g49,1g4j,1g4k,1g4o,1g50,1g52,1g53,1g54,1g5f,1g5s,1g6g,1g6r,1
g6s,1g74,1g7f,1g7g,1g7p,1g7q,1g7v,1g85,1g98,1g9a,1g9b,1g9c,1g9d,1g9r,1g9s,
1g9t,1ga8,1ga9,1gaf,1gag,1gah,1gai,1gar,1gbq,1gbt,1gca,1gcz,1gfw,1gfy,1gfz
,1ggd,1ggn,1ghv,1ghw,1ghy,1ghz,1gi1,1gi4,1gi6,1gi7,1gi8,1gi9,1gj4,1gj5,1gj
6,1gj7,1gj8,1gja,1gjb,1gjc,1gjd,1gmy,1gni,1gnj,1gnm,1gnn,1gno,1gny,1gpk,1g
pn,1gpy,1gqs,1grp,1gsf,1gsz,1gt1,1gt3,1gt4,1gt5,1gu1,1gu3,1gui,1guw,1gux,1
gvk,1gvu,1gvw,1gvx,1gwm,1gwq,1gwr,1gwv,1gww,1gx0,1gx4,1gx8,1gym,1gyx,1gyy,
1gz3,1gz4,1gz9,1gzc,1gzg,1gzv,1h00,1h01,1h07,1h08,1h0a,1h0r,1h0w,1h1b,1h1d
,1h1h,1h1p,1h1q,1h1r,1h1s,1h22,1h23,1h24,1h25,1h26,1h27,1h28,1h2k,1h2t,1h2

u,1h35,1h36,1h37,1h39,1h3a,1h3b,1h3c,1h3h,1h46,1h4n,1h4w,1h5u,1h5v,1h60,1h
61,1h62,1h6e,1h6h,1h79,1h7a,1h8l,1h8s,1h8y,1h9l,1h9z,1ha2,1haa,1hbj,1hbv,1
hc9,1hdq,1hdt,1hee,1hef,1heg,1hfs,1hge,1hgi,1hgj,1hgt,1hi3,1hi4,1hi5,1hih,
1hii,1him,1hiv,1hiy,1hk1,1hk2,1hk3,1hk4,1hk5,1hkj,1hkk,1hkm,1hlf,1hlk,1hmr
,1hms,1hmt,1hn2,1hn4,1hnn,1hos,1hp0,1hp5,1hpo,1hps,1hpv,1hpx,1hq5,1hqf,1hq
g,1hqh,1hrn,1hs6,1hsg,1hsh,1hsl,1hte,1htf,1htg,1hti,1hty,1hv5,1hvh,1hvi,1h
vj,1hvk,1hvl,1hvr,1hvs,1hvy,1hwr,1hww,1hxb,1hxk,1hxw,1hy7,1hyo,1hyv,1hyz,1
i00,1i1e,1i2s,1i32,1i33,1i37,1i3z,1i41,1i43,1i48,1i5d,1i5h,1i5r,1i6v,1i72,
1i7c,1i7g,1i7i,1i7m,1i7z,1i80,1i8h,1i8i,1i8j,1i8z,1i90,1i91,1i9l,1i9m,1i9n
,1i9o,1i9p,1i9q,1iau,1ibc,1ibg,1icj,1ida,1idb,1idg,1ie9,1iem,1iep,1iew,1if
7,1if8,1igb,1igj,1ih0,1iht,1ihy,1ii5,1iig,1iih,1iiq,1ijr,1ik4,1ikt,1ikv,1i
kw,1ikx,1iky,1il3,1il4,1il5,1il9,1ilh,1ilq,1imx,1inc,1ind,1inf,1ing,1inh,1
inq,1iq1,1irs,1is0,1it6,1iup,1ivp,1iwq,1iy7,1iyl,1izh,1izi,1j01,1j07,1j14,
1j15,1j16,1j17,1j19,1j1a,1j36,1j37,1j4k,1j4p,1j4q,1j4r,1j5i,1j7z,1j80,1j81
,1jak,1jao,1jaq,1jbd,1jcx,1jd0,1jd5,1jd6,1jdj,1jet,1jeu,1jev,1jfh,1jg0,1jg
l,1jh1,1jif,1jii,1jij,1jik,1jil,1jiz,1jj9,1jje,1jjk,1jjt,1jk7,1jld,1jlq,1j
lr,1jlx,1jm4,1jmf,1jmg,1jmi,1jmq,1jn2,1jn4,1joc,1joj,1jp5,1jpl,1jq3,1jq8,1
jq9,1jqd,1jqe,1jqy,1jr1,1jrs,1jsv,1jt1,1jtq,1juf,1juj,1juq,1jut,1juy,1jvp,
1jvu,1jwm,1jws,1jwt,1jwu,1jyc,1jyi,1jyq,1jys,1jzs,1k03,1k06,1k08,1k1i,1k1j
,1k1l,1k1m,1k1n,1k1o,1k1p,1k1y,1k21,1k22,1k27,1k2i,1k2v,1k3n,1k3q,1k3t,1k4
g,1k4h,1k6c,1k6p,1k6t,1k6v,1k9q,1k9r,1k9s,1ka7,1kak,1kat,1kav,1kc5,1kc7,1k
cs,1kdk,1kds,1kdw,1ke0,1ke3,1ke5,1ke6,1ke7,1ke8,1ke9,1kel,1kf0,1kf6,1kfy,1
kjr,1kkq,1kl3,1kl5,1klg,1kll,1klu,1km3,1kmv,1kmy,1kna,1kne,1koj,1kpm,1kqb,
1kr3,1ksn,1kti,1ktt,1kug,1kui,1kuk,1kv1,1kv2,1kv5,1kvo,1kwq,1kwr,1kyn,1kyv
,1kz8,1kzk,1kzn,1l0a,1l2s,1l2z,1l5q,1l5r,1l5s,1l6m,1l6s,1l6y,1l7x,1l83,1l8
g,1laf,1lag,1lah,1lan,1lb6,1lbf,1lbk,1lcj,1lcp,1ld7,1ld8,1lee,1leg,1lek,1l
ev,1lf2,1lf3,1lf8,1lf9,1lfo,1lgt,1lgw,1lhc,1lhd,1lhe,1lhf,1lhg,1lhu,1lhv,1
lhw,1li2,1li3,1li6,1ljt,1lke,1lkk,1lkl,1lkx,1ll4,1llb,1lnm,1lol,1loq,1lor,
1los,1lox,1lpg,1lpk,1lpz,1lq2,1lqd,1lqe,1lqf,1lrh,1lrt,1lst,1lt5,1lt6,1lv8
,1lvc,1lvk,1lvu,1lxf,1lxh,1lyb,1lyx,1lzo,1lzq,1m0b,1m0n,1m0o,1m0q,1m13,1m1
b,1m21,1m2p,1m2q,1m2r,1m2x,1m48,1m4h,1m51,1m5b,1m5c,1m5d,1m5e,1m5f,1m5w,1m
6p,1m74,1m7d,1m7i,1m7q,1m7y,1m83,1m9n,1mai,1mau,1maw,1mcz,1mdl,1me3,1me4,1
me7,1me8,1mem,1mes,1met,1meu,1mf4,1mfa,1mfd,1mfg,1mfi,1mh5,1mhw,1mik,1mj7,
1mjj,1mkd,1ml1,1mm6,1mm7,1mmp,1mmq,1mmr,1mn9,1mnc,1mns,1moq,1mpa,1mpl,1mq1
,1mq5,1mq6,1mqd,1mqg,1mqh,1mqi,1mqj,1mrn,1mrs,1mrw,1mrx,1ms0,1ms6,1ms7,1ms
m,1msn,1mto,1mtr,1mu6,1mu8,1mue,1mui,1mv0,1mwn,1mwt,1mx1,1mxl,1mxo,1mxu,1m
y2,1my3,1my4,1my8,1mzc,1mzs,1n0s,1n0t,1n1g,1n1m,1n1t,1n1v,1n2v,1n3i,1n3w,1
n3z,1n43,1n46,1n4h,1n4k,1n4m,1n51,1n5r,1n5z,1n7i,1n7j,1n7m,1n7t,1n8u,1n8v,
1n94,1n95,1n9a,1n9m,1nax,1nc1,1nc3,1nc6,1nd5,1nde,1ndj,1ndv,1ndw,1ndy,1ndz
,1nf8,1nfs,1nfu,1nfw,1nfx,1nfy,1ngw,1nh0,1nhg,1nhu,1nhv,1nhw,1nhx,1nhz,1ni
1,1nj1,1nj5,1nja,1njb,1njc,1njd,1nje,1njf,1njj,1njs,1njt,1nju,1nki,1nkm,1n
l4,1nl6,1nl9,1nli,1nlj,1nlo,1nlp,1nlt,1nm6,1nmk,1nms,1nnb,1nnk,1nnu,1nny,1
no6,1no9,1noi,1noj,1nok,1nox,1np0,1npa,1npv,1npw,1npz,1nq0,1nq7,1nqc,1nt1,
1ntk,1ntv,1nu1,1nu3,1nu8,1nvq,1nvr,1nvs,1nw4,1nw5,1nw7,1nwl,1nxy,1ny0,1ny2
,1nym,1nyx,1nyy,1nz7,1nzl,1nzq,1nzv,1o0d,1o0f,1o0h,1o0m,1o0n,1o0o,1o1s,1o2
g,1o2h,1o2j,1o2k,1o2n,1o2o,1o2p,1o2q,1o2r,1o2s,1o2t,1o2u,1o2v,1o2w,1o2x,1o
2y,1o2z,1o30,1o32,1o33,1o34,1o35,1o36,1o37,1o38,1o39,1o3b,1o3c,1o3d,1o3e,1
o3f,1o3g,1o3h,1o3i,1o3j,1o3k,1o3l,1o3p,1o41,1o42,1o43,1o44,1o45,1o46,1o47,
1o48,1o49,1o4a,1o4b,1o4d,1o4e,1o4f,1o4g,1o4h,1o4i,1o4j,1o4k,1o4l,1o4m,1o4n
,1o4o,1o4p,1o4q,1o4r,1o5a,1o5b,1o5c,1o5e,1o5f,1o5g,1o5m,1o5p,1o5r,1o6h,1o6
i,1o6q,1o6r,1o79,1o7o,1o86,1o8b,1o9d,1o9e,1o9k,1oai,1oar,1oau,1oay,1oba,1o
bx,1ocn,1ocq,1od8,1odi,1odj,1ody,1oe0,1oe7,1oe8,1oeb,1ofz,1ogd,1ogg,1ogu,1
ogx,1ogz,1oh4,1ohr,1oi9,1oif,1oim,1oiq,1oir,1oit,1oiu,1oiy,1oj5,1ok7,1okl,
1oko,1okv,1okw,1okx,1oky,1okz,1ol1,1ol2,1ols,1olu,1olx,1om1,1om2,1om9,1ong

,1onh,1onp,1ony,1onz,1opi,1oq5,1oqp,1ork,1orw,1os0,1os5,1osg,1oss,1osv,1ot
h,1ouk,1ouy,1ov3,1ove,1ow4,1ow6,1ow7,1ow8,1owd,1owe,1owh,1owi,1owj,1owk,1o
x9,1oxg,1oxn,1oxq,1oxr,1oy7,1oyn,1oyq,1oyt,1oz0,1ozv,1p01,1p02,1p03,1p04,1
p05,1p06,1p0y,1p10,1p17,1p19,1p1n,1p1o,1p1q,1p28,1p2a,1p2g,1p4r,1p4u,1p57,
1p5e,1p6d,1p6e,1p7m,1p93,1pa9,1pau,1pb8,1pb9,1pbk,1pbq,1pcg,1pdq,1pdz,1pf7
,1pf8,1pfu,1pfy,1pg2,1pgp,1ph0,1phw,1pi4,1pi5,1pig,1pip,1pk0,1pkx,1pl0,1pm
e,1pmn,1pmu,1pmv,1pmx,1pop,1pot,1ppc,1pph,1ppi,1ppk,1ppl,1ppm,1ppw,1ppx,1p
q3,1pq6,1pqc,1pr1,1pr5,1prl,1prm,1pro,1ps3,1pu7,1pu8,1pum,1pun,1puq,1pus,1
pvn,1pw6,1pwp,1pwq,1pwu,1pwy,1px4,1pxh,1pxi,1pxj,1pxk,1pxl,1pxm,1pxn,1pxo,
1pxp,1py1,1py2,1py5,1pye,1pyg,1pyn,1pyw,1pz5,1pzi,1pzj,1pzo,1pzp,1q0b,1q1g
,1q1m,1q1y,1q3d,1q3w,1q41,1q4k,1q4l,1q4w,1q4x,1q54,1q5k,1q5l,1q63,1q65,1q6
6,1q6j,1q6k,1q6m,1q6n,1q6p,1q6s,1q6t,1q72,1q7a,1q83,1q84,1q8t,1q8u,1q8w,1q
91,1q95,1q9d,1q9m,1qan,1qaq,1qaw,1qb1,1qb6,1qb9,1qbn,1qbo,1qbq,1qbr,1qbs,1
qbt,1qbu,1qbv,1qca,1qcp,1qf0,1qf1,1qf2,1qf4,1qf5,1qfs,1qft,1qhc,1qhr,1qi0,
1qin,1qiw,1qj1,1qj6,1qj7,1qja,1qjb,1qji,1qk3,1qk4,1qka,1qkb,1qkn,1qkt,1qku
,1ql7,1ql9,1qm4,1qm5,1qng,1qnh,1qon,1qpb,1qpe,1qpf,1qpl,1qq9,1qr3,1qs4,1qs
c,1qti,1qtn,1qvt,1qvu,1qw7,1qwe,1qwf,1qwu,1qx1,1qxk,1qxl,1qxw,1qxy,1qxz,1q
y1,1qy2,1qyg,1r0p,1r0x,1r10,1r17,1r1h,1r1i,1r1j,1r2b,1r4w,1r58,1r5g,1r5h,1
r5n,1r5v,1r5w,1r5y,1r6g,1r6n,1r6z,1r78,1r9l,1rbo,1rbp,1rd4,1rdi,1rdj,1rdl,
1rdn,1rdt,1re1,1re8,1rej,1rek,1rev,1rgj,1rgk,1rgl,1rhj,1rhk,1rhm,1rhq,1rhr
,1rhu,1rin,1riw,1rjk,1rlp,1rlq,1rm8,1rmz,1rne,1rnm,1rnt,1ro6,1ro7,1ros,1rp
7,1rpa,1rpf,1rpj,1rq2,1rql,1rr6,1rri,1rrw,1rry,1rs2,1rs4,1rsd,1rsi,1rst,1r
t1,1rt2,1rt9,1rtf,1rth,1rti,1rtl,1ru1,1ru2,1rv1,1rw8,1rwq,1rww,1rwx,1rxp,1
ryf,1ryh,1rzx,1s17,1s19,1s26,1s38,1s39,1s3k,1s4d,1s50,1s5q,1s5z,1s63,1s64,
1s89,1s9t,1s9v,1sb1,1sbg,1sbr,1sc8,1sdt,1sdu,1sdv,1sfi,1sgu,1sh9,1shc,1shd
,1siv,1sj0,1sje,1sjh,1skj,1sl3,1sld,1sle,1slg,1sln,1sm2,1sm3,1sme,1snk,1so
2,1soj,1sps,1sqa,1sqb,1sqc,1sqi,1sqn,1sqo,1sqp,1sqq,1sqt,1sr7,1sre,1srg,1s
ri,1ssq,1stc,1std,1stp,1str,1sts,1sv3,1sve,1svg,1svh,1sw1,1sw2,1swg,1swi,1
swk,1swn,1swp,1swr,1syh,1syi,1syo,1sz0,1szd,1szm,1t08,1t13,1t1r,1t1s,1t29,
1t2v,1t31,1t32,1t37,1t3t,1t46,1t48,1t49,1t4e,1t4j,1t4s,1t4v,1t5a,1t5f,1t69
,1t79,1t7d,1t7f,1t7j,1t7r,1ta2,1ta6,1tbz,1tc1,1tcw,1tcx,1td7,1tet,1tfq,1tf
t,1tfz,1tg5,1thl,1thr,1ths,1thz,1tjp,1tka,1tkb,1tkc,1tkt,1tkx,1tkz,1tl1,1t
l3,1tl7,1tl9,1tlo,1tlp,1tmb,1tmm,1tmn,1tng,1tnh,1tni,1tnj,1tnk,1tnl,1tog,1
toi,1toj,1tok,1tom,1tou,1tow,1tps,1tpw,1tpz,1tq4,1tqf,1tr7,1trd,1tsi,1tsl,
1tsm,1tsv,1tsy,1tt1,1ttm,1ttv,1tu6,1tuf,1tv6,1tve,1tvo,1tvr,1tx7,1txr,1tyn
,1tyr,1tys,1tze,1u0g,1u0h,1u1b,1u1w,1u2r,1u2y,1u32,1u33,1u3q,1u3r,1u3s,1u5
9,1u65,1u6q,1u71,1u8t,1u9e,1u9l,1u9q,1u9v,1u9w,1u9x,1ua4,1ucn,1udt,1udu,1u
ef,1ugp,1ugw,1ugx,1ugy,1uh1,1uho,1ui0,1uj0,1uj5,1uj6,1ujj,1ujk,1uk0,1uk1,1
ukh,1ukt,1ule,1ulg,1uml,1umw,1ung,1unh,1unl,1uod,1uom,1uou,1upf,1upk,1ur9,
1urc,1urg,1urw,1usi,1usk,1usn,1utc,1uti,1utj,1utl,1utm,1utn,1uto,1utp,1utr
,1utt,1utz,1uu3,1uu7,1uu8,1uu9,1uv5,1uv6,1uvr,1uvs,1uvt,1uvu,1uw6,1uwb,1uw
f,1uwh,1uwt,1uwu,1ux7,1uxa,1uxb,1uy6,1uy7,1uy8,1uy9,1uyc,1uyd,1uye,1uyf,1u
yg,1uyh,1uyi,1uyk,1uym,1uys,1uz1,1uz4,1uz8,1uze,1v0k,1v0l,1v0m,1v0n,1v0o,1
v0p,1v11,1v16,1v1j,1v1k,1v1m,1v2h,1v2j,1v2k,1v2l,1v2m,1v2n,1v2o,1v2p,1v2q,
1v2r,1v2s,1v2t,1v2u,1v2v,1v2w,1v3x,1v41,1v48,1v79,1v7a,1vcj,1vcu,1vea,1veb
,1vfn,1vgc,1vij,1vik,1vj5,1vj6,1vj9,1vja,1vjb,1vjc,1vjd,1vjy,1vkj,1vot,1vr
1,1vrt,1vru,1vsn,1vso,1vwf,1vwl,1vwn,1vyf,1vyg,1vyj,1vyq,1vyw,1vyz,1vzq,1w
0x,1w0y,1w0z,1w10,1w11,1w12,1w13,1w14,1w1d,1w1g,1w1p,1w1t,1w1v,1w1y,1w22,1
w25,1w2g,1w2h,1w2k,1w2x,1w31,1w3j,1w3k,1w3l,1w4l,1w4o,1w4p,1w4q,1w51,1w5v,
1w5w,1w5x,1w5y,1w6h,1w6j,1w6r,1w6y,1w70,1w76,1w7g,1w7h,1w7x,1w80,1w82,1w83
,1w84,1w8l,1w8m,1w96,1w9u,1w9v,1waw,1wax,1way,1wb0,1wbg,1wbn,1wbo,1wbs,1wb
t,1wbv,1wbw,1wc1,1wc6,1wcc,1wcq,1wdn,1wdq,1wdr,1wdy,1we2,1wht,1wkm,1wm1,1w
n6,1wok,1wqv,1ws1,1ws4,1ws5,1wss,1wtg,1wu1,1wug,1wum,1wun,1wuq,1wur,1wv7,1
wva,1wvc,1wvj,1wxz,1wzy,1x07,1x0n,1x11,1x1z,1x38,1x39,1x6u,1x70,1x76,1x78,

1x7a,1x7b,1x7e,1x7q,1x7r,1x81,1x8b,1x8d,1x8j,1x8r,1x8s,1x8t,1x9d,1xa5,1xap
,1xb7,1xbb,1xbc,1xbo,1xd0,1xdd,1xdg,1xff,1xfv,1xge,1xgi,1xgj,1xh3,1xh4,1xh
5,1xh6,1xh7,1xh8,1xh9,1xhm,1xhy,1xjd,1xk5,1xk9,1xka,1xkk,1xlx,1xlz,1xm4,1x
m6,1xmu,1xmy,1xn0,1xn2,1xn3,1xnx,1xnz,1xo2,1xoe,1xog,1xom,1xon,1xoq,1xor,1
xos,1xot,1xow,1xoz,1xp0,1xp1,1xp6,1xp9,1xpc,1xpz,1xq0,1xqc,1xr8,1xr9,1xs7,
1xsc,1xt3,1xt8,1xuc,1xud,1xug,1xuo,1xur,1xws,1xxe,1xxh,1xxi,1xz8,1xzx,1y0l
,1y0x,1y19,1y1m,1y1z,1y20,1y2a,1y2b,1y2c,1y2d,1y2e,1y2f,1y2g,1y2h,1y2j,1y2
k,1y3a,1y3g,1y3n,1y3p,1y3v,1y3w,1y3x,1y3y,1y4z,1y57,1y6a,1y6b,1y6q,1y6r,1y
8o,1y8p,1y91,1y98,1ya4,1ybg,1ybo,1yc1,1yc4,1yc5,1yci,1ycm,1yda,1ydb,1ydd,1
ydk,1ydr,1yds,1ydt,1yei,1yej,1yet,1yfz,1yhm,1yhs,1yi3,1yid,1yk7,1ykp,1ykr,
1ylv,1yly,1ym1,1ym2,1ym4,1yms,1ymx,1ynd,1yon,1you,1yp9,1ype,1ypg,1ypj,1yq7
,1yqj,1yqy,1yrs,1ysg,1ysi,1yt7,1yt9,1yvf,1yvh,1yvm,1yvx,1yvz,1yw2,1yw7,1yw
8,1ywh,1ywi,1ywr,1yxd,1yy4,1yy6,1yye,1yyr,1yys,1yyy,1yz3,1z1h,1z1r,1z2b,1z
34,1z3c,1z3j,1z3t,1z3v,1z4n,1z4o,1z4u,1z5m,1z6d,1z6e,1z6f,1z6j,1z6p,1z6q,1
z6s,1z71,1z95,1z9g,1z9h,1z9y,1zaf,1zaj,1zc9,1zd2,1zd3,1zd4,1zd5,1zdp,1ze8,
1zea,1zeo,1zfk,1zfp,1zfq,1zgb,1zge,1zgi,1zgv,1zh7,1zhk,1zhl,1zhy,1zkk,1zkl
,1zkn,1zky,1zm6,1zm7,1zoe,1zog,1zoh,1zom,1zp5,1zp8,1zpa,1zpb,1zpc,1zrz,1zs
0,1zsb,1zsf,1zsh,1zsr,1ztq,1zub,1zuc,1zvx,1zxc,1zxv,1zyj,1zyr,1zz1,1zz2,1z
z3,1zzl,1zzz,220l,223l,2a0c,2a0t,2a14,2a25,2a29,2a2g,2a2x,2a3a,2a3b,2a3c,2
a3i,2a3w,2a3x,2a4g,2a4l,2a4m,2a4q,2a4r,2a4w,2a4z,2a58,2a5b,2a5c,2a5s,2a5u,
2a8g,2a8h,2aa6,2aa9,2aac,2aay,2ad5,2ada,2adm,2adu,2aeb,2aei,2ael,2aez,2afw
,2afx,2agv,2ai7,2ai8,2aia,2aie,2aig,2aj8,2ajb,2ajd,2ajl,2al5,2alv,2am1,2am
2,2am4,2am9,2ama,2amt,2amv,2an5,2ank,2anl,2anm,2ans,2ao6,2aoc,2aod,2aoe,2a
of,2aog,2aoh,2aoi,2aoj,2aou,2aov,2aow,2aox,2aq7,2aq9,2aqb,2aqu,2arm,2asu,2
ate,2ath,2auc,2aux,2auz,2avi,2avm,2avo,2avq,2avs,2avv,2aw1,2ax6,2ax9,2axi,
2ay1,2ay2,2ay3,2ay4,2ay5,2ay6,2ay7,2ay8,2ay9,2ayp,2ayr,2az5,2az8,2az9,2azb
,2azc,2azm,2azr,2b07,2b17,2b1g,2b1i,2b1p,2b1q,2b1r,2b1v,2b1z,2b2v,2b4l,2b4
m,2b52,2b53,2b54,2b55,2b5j,2b7a,2b7d,2b7f,2b8l,2b8v,2b9a,2baj,2bak,2bal,2b
an,2bb7,2bba,2bbb,2bcd,2bdf,2bdj,2bdl,2bdy,2be2,2bed,2bes,2bet,2bfq,2bfr,2
bgd,2bge,2bgn,2bgr,2bj4,2bjm,2bks,2bkt,2bkz,2bmc,2bmg,2bmk,2bmv,2bmz,2bo4,
2boh,2boi,2boj,2bok,2bow,2bpm,2bpv,2bpx,2bpy,2bq6,2bq7,2bqv,2bqw,2br1,2br6
,2br8,2brb,2brc,2brg,2brh,2brm,2brn,2bro,2brp,2bt9,2btr,2bts,2bu5,2bua,2bu
b,2buc,2buv,2bv4,2bvd,2bve,2bvr,2bvs,2bvx,2bw7,2bxt,2bxu,2byh,2byi,2byp,2b
yr,2bys,2bz5,2bz6,2bz8,2bza,2bzz,2c02,2c0o,2c1a,2c1b,2c1n,2c1p,2c1q,2c2l,2
c3i,2c3j,2c3k,2c3l,2c4f,2c4g,2c4v,2c4w,2c57,2c5n,2c5o,2c5x,2c5y,2c68,2c69,
2c6c,2c6e,2c6g,2c6i,2c6k,2c6l,2c6m,2c6n,2c6o,2c80,2c8w,2c8x,2c8y,2c90,2c92
,2c93,2c94,2c97,2c9b,2c9d,2c9t,2ca8,2cbj,2cbr,2cbs,2cbu,2cbv,2cbz,2cc7,2cc
b,2ccc,2ccs,2cct,2ccu,2ce9,2cej,2cem,2cen,2ceo,2ceq,2cer,2ces,2cet,2cex,2c
f8,2cf9,2cfd,2cfg,2cgf,2cgr,2cgu,2cgv,2cgw,2cgx,2chm,2cht,2chw,2chx,2chz,2
ci9,2cia,2cji,2ckm,2cle,2clf,2clh,2cli,2clk,2cll,2clm,2clo,2clv,2clx,2cm7,
2cm8,2cma,2cmb,2cmc,2cmf,2cmo,2cn0,2cn8,2cne,2cnf,2cng,2cnh,2cni,2co0,2csm
,2csn,2ctc,2cv3,2cvd,2d06,2d0k,2d1n,2d1o,2d1x,2d2v,2d3u,2d3z,2d41,2dbl,2dd
f,2df6,2doo,2dq7,2drc,2dri,2ds1,2dua,2duv,2dw7,2dwx,2dxs,2e1w,2e27,2e2b,2e
2p,2e2r,2e5y,2e7f,2e7l,2e91,2e92,2e93,2e94,2e95,2e98,2e99,2e9a,2e9c,2e9d,2
e9n,2e9o,2e9u,2e9v,2ea2,2ea4,2eep,2eg7,2eg8,2eh8,2ei6,2emt,2epn,2er0,2er6,
2er9,2erz,2esm,2etk,2etm,2etr,2euf,2euk,2eum,2evc,2evl,2evm,2evo,2ew5,2ew6
,2ewa,2ewb,2ewp,2ews,2ewy,2exc,2exg,2exm,2ez5,2ez7,2f01,2f0z,2f10,2f14,2f1
8,2f1a,2f1b,2f1g,2f2c,2f2h,2f34,2f35,2f3e,2f3f,2f3k,2f3r,2f4b,2f4j,2f5t,2f
6j,2f6t,2f6v,2f6y,2f6z,2f70,2f71,2f7i,2f7o,2f7p,2f80,2f81,2f89,2f8g,2f8i,2
f94,2f9b,2f9k,2f9u,2f9v,2fah,2fai,2fb8,2fci,2fda,2fdd,2fde,2fdp,2feq,2fes,
2ff1,2ff2,2fgh,2fgi,2fgu,2fgv,2fhy,2fie,2fix,2fj0,2fjm,2fjn,2fjp,2fkf,2fky
,2fl2,2fl5,2fl6,2flb,2fle,2flh,2flr,2flu,2fm0,2fm2,2fm5,2fmb,2fme,2fo4,2fo
u,2fov,2foy,2fpz,2fq6,2fqo,2fqt,2fqw,2fqx,2fqy,2fr3,2fr8,2frd,2fs8,2fs9,2f
sa,2fsv,2ftd,2fts,2fu8,2fum,2fuu,2fv5,2fv9,2fvc,2fvd,2fw3,2fw6,2fwp,2fwy,2

fwz,2fx6,2fx7,2fx8,2fx9,2fxr,2fxs,2fxu,2fxv,2fys,2fyv,2fzc,2fzg,2fzk,2fzz,
2g00,2g01,2g0g,2g0h,2g19,2g1q,2g1r,2g1y,2g24,2g2r,2g5p,2g5t,2g5u,2g63,2g6p
,2g6q,2g70,2g71,2g72,2g78,2g79,2g83,2g8n,2g8r,2g94,2g96,2g97,2g9q,2g9r,2g9
u,2g9v,2g9x,2ga2,2gbf,2gbg,2gbi,2gc8,2gcd,2gd8,2gde,2gdo,2gej,2gek,2gfa,2g
fd,2gfj,2gfk,2gfs,2gg0,2gg2,2gg3,2gg5,2gg7,2gg8,2gg9,2gga,2ggb,2ggd,2ggu,2
ggx,2gh6,2gh7,2gh9,2ghg,2giu,2gj4,2gj5,2gkl,2gl0,2glm,2glp,2gm1,2gm9,2gmk,
2gmv,2gmx,2gnf,2gnh,2gni,2gnj,2gnl,2go4,2gph,2gpp,2gqn,2gss,2gst,2gsu,2gtk
,2gtv,2gu8,2gv2,2gv6,2gv7,2gvd,2gvf,2gvj,2gvv,2gvz,2gyi,2gz2,2gz7,2gz8,2gz
l,2h02,2h03,2h13,2h15,2h1h,2h21,2h23,2h2d,2h2e,2h2g,2h2h,2h2j,2h3e,2h42,2h
44,2h4g,2h4k,2h4n,2h5a,2h5d,2h5e,2h5i,2h5j,2h65,2h6b,2h6k,2h6q,2h6t,2h8h,2
h96,2h9m,2h9n,2h9p,2h9t,2ha0,2ha2,2ha3,2ha4,2ha5,2ha6,2ha7,2hah,2hai,2haw,
2hb1,2hb3,2hb9,2hd1,2hd6,2hdq,2hdr,2hds,2hdu,2hdx,2hf8,2hfp,2hh5,2hha,2hhn
,2hiw,2hiz,2hj4,2hjb,2hk5,2hkf,2hl4,2hm1,2hmb,2hmh,2hmu,2hmv,2hmw,2hnc,2hn
x,2hny,2hob,2hoc,2hog,2hpa,2hqu,2hr6,2hrm,2hrp,2hs1,2hs2,2hu6,2hug,2hvc,2h
vx,2hw2,2hwg,2hwh,2hwi,2hwo,2hwp,2hxl,2hxm,2hxq,2hy0,2hyy,2hz0,2hz4,2hzi,2
hzl,2hzn,2hzy,2i03,2i0a,2i0d,2i0e,2i0g,2i0h,2i0j,2i0v,2i0y,2i19,2i1m,2i1r,
2i2b,2i2c,2i3h,2i3i,2i3v,2i3z,2i40,2i47,2i4d,2i4j,2i4p,2i4t,2i4u,2i4v,2i4w
,2i4x,2i4z,2i5f,2i5j,2i6a,2i6b,2i72,2i7c,2i80,2ica,2idk,2idw,2ie4,2ieh,2ie
n,2ieo,2ig0,2igv,2igw,2igx,2igy,2ihj,2ihq,2iit,2iiv,2iko,2iku,2il2,2ilp,2i
ma,2imb,2imd,2in6,2io6,2ioa,2iog,2iok,2ipo,2iqg,2irz,2is0,2isc,2isv,2isw,2
it4,2itk,2ito,2itp,2itt,2ity,2itz,2iu0,2iuz,2iv9,2ivu,2ivz,2iw6,2iw8,2iw9,
2iws,2iwu,2iwx,2iyf,2izl,2izx,2j27,2j2i,2j2u,2j34,2j3q,2j47,2j4a,2j4g,2j4i
,2j4k,2j4q,2j4z,2j50,2j62,2j6m,2j75,2j77,2j78,2j79,2j7b,2j7d,2j7e,2j7f,2j7
g,2j7h,2j7w,2j7x,2j83,2j87,2j94,2j95,2j9a,2j9h,2j9l,2j9n,2ja3,2jaj,2jal,2j
b5,2jb6,2jbj,2jbk,2jbl,2jbo,2jbp,2jbu,2jbv,2jc0,2jdh,2jdk,2jdl,2jdm,2jdn,2
jdo,2jdp,2jds,2jdt,2jdu,2jdv,2jdy,2jew,2jf4,2jfh,2jfz,2jg0,2jg8,2jgs,2jh0,
2jh5,2jh6,2jiu,2jiw,2jj3,2jjb,2jjk,2jjr,2jk7,2jk9,2jke,2jkh,2jkk,2jkm,2jko
,2jkp,2jkq,2jkr,2jkt,2jld,2jle,2jmj,2jnp,2jnw,2jo9,2joa,2jq9,2jqi,2jqk,2jq
l,2jsd,2jst,2jt5,2jt6,2jup,2jxr,2k00,2k0g,2k0x,2k1q,2k2g,2k2r,2k31,2k3w,2k
46,2k4i,2k62,2k7l,2kaw,2kbr,2kbs,2kce,2kdh,2ke1,2kff,2kfg,2kfh,2kfx,2kgi,2
khh,2kmx,2knh,2ko7,2koh,2kp8,2kpl,2krd,2ks9,2ksa,2ksb,2ksp,2kup,2kvm,2kwn,
2kzu,2l0i,2l11,2l12,2l1b,2l1r,2l3r,2l65,2l6e,2l6j,2l75,2l7u,2l84,2l8j,2l8r
,2l98,2las,2lbm,2lbv,2lcs,2lct,2lgf,2lh8,2lha,2liq,2lk1,2lkk,2lko,2ll6,2ll
7,2llo,2llq,2lnw,2lo6,2loz,2lp8,2lpr,2lsk,2lsp,2lsr,2lsv,2lto,2ltv,2ltw,2l
tx,2lty,2ltz,2lwi,2ly0,2lya,2lyb,2lyw,2lzg,2m0o,2m0u,2m0v,2m3m,2m3o,2m3z,2
m41,2mas,2mc1,2mg5,2mip,2mji,2mkr,2mlm,2mnz,2mov,2mow,2mpa,2mpm,2mps,2ms4,
2mwo,2mwp,2mwy,2n06,2n0u,2n0w,2n14,2n1g,2n27,2n3k,2n7b,2n8t,2n9e,2n9x,2ncz
,2nd0,2ndf,2ndg,2ndo,2nm1,2nmb,2nmx,2nmy,2nmz,2nn1,2nn7,2nn8,2nnd,2nng,2nn
k,2nno,2nnp,2nnq,2nns,2nnv,2no3,2np8,2np9,2nq6,2nq7,2nqg,2nqi,2nsj,2nsl,2n
sx,2nt7,2nta,2ntf,2nv7,2nw4,2nwl,2nwn,2nww,2nxd,2nxl,2nxm,2nyr,2o0u,2o1c,2
o1v,2o22,2o2u,2o3p,2o3z,2o48,2o4h,2o4j,2o4k,2o4l,2o4n,2o4p,2o4r,2o4s,2o4z,
2o5d,2o5k,2o63,2o64,2o65,2o7e,2o7n,2o7v,2o8h,2o9a,2o9i,2o9j,2o9k,2o9r,2o9v
,2oa0,2oag,2oah,2oax,2oaz,2obf,2obj,2obo,2oc0,2oc1,2oc2,2oc4,2oc7,2oc9,2od
d,2oei,2of2,2of4,2off,2ofu,2ofv,2og8,2ogy,2ogz,2oh0,2oh4,2ohk,2ohl,2ohm,2o
hp,2ohq,2ohr,2ohs,2oht,2ohu,2ohv,2oi0,2oi2,2oi3,2oi9,2oic,2oiq,2oj9,2ojf,2
ojg,2oji,2ojj,2ok1,2olb,2ole,2on3,2on6,2onb,2onc,2ony,2onz,2oo8,2ooh,2ooz,
2op3,2op9,2opb,2oph,2opy,2oq6,2oqi,2oqs,2oqv,2or4,2or9,2ork,2os9,2osc,2osf
,2osm,2ot1,2ou7,2ov4,2ovq,2ovv,2ovx,2ovy,2ovz,2ow0,2ow1,2ow2,2ow3,2ow6,2ow
7,2ow9,2owb,2oxd,2oxn,2oxx,2oxy,2oyk,2oyl,2oym,2oz2,2oz5,2oz6,2oz7,2ozr,2p
09,2p0d,2p0x,2p15,2p16,2p1c,2p2a,2p2h,2p2i,2p33,2p3a,2p3b,2p3c,2p3d,2p3g,2
p3i,2p3o,2p4i,2p4j,2p4s,2p4y,2p53,2p59,2p7a,2p7g,2p7z,2p83,2p8h,2p8n,2p8o,
2p8s,2p93,2p94,2p95,2p98,2p99,2p9a,2pax,2pbw,2pcp,2pcu,2pe0,2pe1,2pe2,2peh
,2pem,2pfy,2pg2,2pgj,2pgl,2pgz,2ph6,2ph8,2ph9,2phb,2pix,2piy,2piz,2pj0,2pj
1,2pj2,2pj3,2pj4,2pj5,2pj6,2pj7,2pj8,2pj9,2pja,2pjb,2pjc,2pjl,2pjt,2pk5,2p

k6,2pks,2pl0,2pl9,2pll,2pmc,2pmk,2pmn,2pnc,2pnx,2pog,2poq,2pou,2pov,2pow,2
pq9,2pqb,2pqc,2pqj,2pql,2pqz,2pr9,2pre,2pri,2prj,2psj,2psu,2psv,2psx,2pt9,
2ptz,2pu0,2pu1,2pu2,2puy,2pv1,2pv2,2pv3,2pvh,2pvj,2pvk,2pvl,2pvm,2pvn,2pvu
,2pvv,2pvw,2pwc,2pwd,2pwg,2pwr,2py4,2pyi,2pym,2pyn,2pyy,2pze,2pzi,2pzy,2q1
1,2q15,2q1j,2q1l,2q1q,2q2a,2q2c,2q2n,2q2y,2q2z,2q38,2q3z,2q54,2q55,2q5k,2q
63,2q64,2q6b,2q6c,2q6f,2q6h,2q70,2q72,2q7m,2q7o,2q7q,2q7y,2q80,2q88,2q89,2
q8g,2q8h,2q8i,2q8m,2q8s,2q8y,2q8z,2q92,2q93,2q94,2q95,2q96,2q9m,2q9n,2q9y,
2qa8,2qaf,2qbp,2qbq,2qbr,2qbs,2qbu,2qbw,2qbx,2qc6,2qcd,2qcf,2qcg,2qch,2qci
,2qcm,2qcn,2qd6,2qd7,2qd8,2qd9,2qdt,2qe2,2qe4,2qe5,2qf6,2qfo,2qft,2qfu,2qg
0,2qg2,2qh6,2qhc,2qhd,2qhm,2qhn,2qhr,2qhy,2qhz,2qi0,2qi1,2qi3,2qi4,2qi5,2q
i6,2qi7,2qic,2qiq,2qju,2qk5,2qk8,2qki,2qky,2ql5,2ql7,2ql9,2qlb,2qlf,2qlj,2
qlm,2qln,2qlq,2qm7,2qm9,2qmd,2qmf,2qmg,2qmj,2qn1,2qn2,2qn3,2qnb,2qnn,2qnp,
2qnq,2qnx,2qnz,2qo1,2qo8,2qoa,2qoe,2qoh,2qp6,2qp8,2qpj,2qpq,2qpu,2qq7,2qqs
,2qrg,2qrh,2qrk,2qrl,2qrm,2qrp,2qrq,2qry,2qt5,2qt9,2qta,2qtb,2qtg,2qtn,2qt
r,2qtt,2qtu,2qu2,2qu3,2qu5,2qu6,2qv7,2qve,2qw1,2qwb,2qwc,2qwd,2qwe,2qwf,2q
wg,2qx0,2qyk,2qyl,2qyn,2qzk,2qzl,2qzr,2qzx,2r02,2r03,2r05,2r0h,2r0u,2r0y,2
r0z,2r1w,2r1x,2r1y,2r23,2r2b,2r2l,2r2m,2r2w,2r38,2r3c,2r3f,2r3g,2r3h,2r3i,
2r3j,2r3k,2r3l,2r3m,2r3n,2r3o,2r3p,2r3t,2r3w,2r3y,2r43,2r4b,2r4f,2r58,2r59
,2r5a,2r5b,2r5d,2r5p,2r5q,2r64,2r6f,2r6n,2r6w,2r6y,2r75,2r7b,2r7g,2r8q,2r9
b,2r9m,2r9s,2r9w,2r9x,2ra0,2ra6,2rc8,2rc9,2rcb,2rcn,2rcu,2rcw,2rcx,2rd6,2r
eg,2rf2,2rfh,2rfn,2rfy,2rg5,2rg6,2rgp,2rgu,2ri9,2ria,2rib,2rin,2rio,2rip,2
rjp,2rjr,2rjs,2rk7,2rk8,2rka,2rkd,2rke,2rkf,2rkg,2rkm,2rkn,2rku,2rl5,2rly,
2rm0,2rnw,2rnx,2rny,2rok,2rol,2rox,2rqu,2rr4,2rt5,2rvn,2sfp,2sim,2srt,2std
,2tmn,2toh,2tpi,2tsr,2upj,2usn,2uue,2uuo,2uup,2uvm,2uw0,2uw3,2uw4,2uw5,2uw
6,2uw7,2uw8,2uwd,2uwl,2uwo,2uwp,2uxi,2uxu,2uxx,2uxz,2uy0,2uy3,2uy4,2uy5,2u
yi,2uym,2uyn,2uyq,2uyw,2uz6,2uz9,2uzb,2uzd,2uze,2uzj,2uzl,2uzn,2uzo,2uzv,2
v00,2v0c,2v0n,2v0z,2v10,2v11,2v12,2v13,2v16,2v22,2v25,2v2c,2v2h,2v2q,2v2v,
2v3d,2v3e,2v3u,2v54,2v57,2v58,2v59,2v5a,2v5x,2v6n,2v77,2v7a,2v7d,2v83,2v85
,2v86,2v87,2v88,2v8w,2v95,2v96,2va5,2va6,2va7,2vaq,2vb8,2vba,2vc7,2vc9,2vc
b,2vcg,2vci,2vcj,2vcq,2vcw,2vcx,2vd0,2vd1,2vd4,2vd7,2ves,2veu,2vev,2vew,2v
ex,2vey,2vf6,2vfk,2vfz,2vgc,2vgo,2vgp,2vh0,2vh6,2vhj,2vhq,2vi5,2vie,2vin,2
vio,2vip,2viq,2viv,2viw,2viy,2viz,2vj1,2vj6,2vj7,2vj8,2vj9,2vjx,2vk2,2vk6,
2vkm,2vl1,2vl4,2vl8,2vle,2vmc,2vmd,2vmf,2vnf,2vnm,2vnn,2vnp,2vnt,2vo4,2vo5
,2vo7,2vot,2vpe,2vpg,2vpn,2vpo,2vpp,2vqj,2vqm,2vqt,2vr0,2vr3,2vr4,2vrj,2vr
x,2vsl,2vt3,2vta,2vtd,2vte,2vth,2vti,2vtj,2vtl,2vtm,2vtn,2vto,2vtp,2vtq,2v
tr,2vts,2vtt,2vu3,2vuk,2vur,2vv9,2vvc,2vvn,2vvo,2vvs,2vvt,2vvu,2vvv,2vw1,2
vw2,2vw5,2vwc,2vwf,2vwl,2vwm,2vwn,2vwo,2vwu,2vwv,2vww,2vwx,2vwy,2vwz,2vx0,
2vx1,2vx9,2vxa,2vxj,2vxn,2vyt,2vzr,2w05,2w06,2w08,2w0d,2w0j,2w0p,2w0s,2w0x
,2w0z,2w10,2w12,2w16,2w17,2w1c,2w1d,2w1e,2w1f,2w1g,2w1h,2w1i,2w26,2w2i,2w2
u,2w3i,2w3k,2w3l,2w3o,2w47,2w4i,2w4x,2w54,2w5g,2w5i,2w66,2w67,2w68,2w6c,2w
6m,2w6n,2w6o,2w6p,2w6q,2w6t,2w6u,2w6z,2w70,2w71,2w73,2w76,2w77,2w78,2w7x,2
w7y,2w85,2w87,2w8f,2w8g,2w8j,2w8w,2w8y,2w92,2w97,2w9h,2w9r,2wa3,2wa4,2wa8,
2waj,2wap,2wb5,2wbb,2wbd,2wbg,2wc3,2wc4,2wca,2wcg,2wcx,2wd1,2wd3,2wd7,2we3
,2web,2wec,2wed,2weg,2weh,2wei,2wej,2weo,2weq,2wer,2wev,2wey,2wez,2wf0,2wf
1,2wf2,2wf3,2wf4,2wf5,2wfg,2wfj,2wgi,2wgj,2wgs,2who,2whp,2wi1,2wi2,2wi3,2w
i4,2wi5,2wi6,2wi7,2wib,2wic,2wig,2wih,2wij,2wik,2wj1,2wj2,2wjg,2wji,2wk2,2
wk6,2wks,2wkt,2wky,2wkz,2wl0,2wl4,2wl5,2wly,2wlz,2wm0,2wmr,2wmu,2wmv,2wmw,
2wmx,2wn9,2wnc,2wnj,2wnl,2wo8,2wo9,2woa,2won,2woq,2wor,2wos,2wot,2wou,2wp1
,2wpa,2wpb,2wq4,2wq5,2wqb,2wqp,2wr8,2wsx,2wtc,2wtd,2wti,2wtj,2wtv,2wtw,2wt
x,2wu6,2wu7,2wuf,2wuu,2wva,2wvt,2wvz,2ww0,2ww2,2wwj,2wxd,2wxf,2wxg,2wxh,2w
xi,2wxj,2wxk,2wxl,2wxm,2wxn,2wxo,2wxp,2wxq,2wxv,2wyf,2wyg,2wyi,2wyj,2wyn,2
wzf,2wzm,2wzs,2wzx,2wzy,2wzz,2x00,2x09,2x0y,2x24,2x2c,2x2i,2x2k,2x2l,2x2m,
2x2r,2x38,2x39,2x3t,2x4o,2x4r,2x4s,2x4t,2x4u,2x4z,2x52,2x5o,2x6d,2x6e,2x6f
,2x6i,2x6j,2x6k,2x6w,2x6x,2x6y,2x7c,2x7d,2x7o,2x7s,2x7t,2x7u,2x7x,2x81,2x8

5,2x8d,2x8e,2x8i,2x8z,2x91,2x95,2x96,2x97,2x9e,2x9f,2xa4,2xab,2xae,2xaf,2x
ag,2xah,2xaj,2xaq,2xas,2xb7,2xb8,2xb9,2xba,2xbj,2xbp,2xbv,2xbw,2xbx,2xc0,2
xc4,2xcg,2xch,2xck,2xcn,2xcs,2xct,2xd6,2xd9,2xda,2xde,2xdk,2xdl,2xdm,2xdw,
2xdx,2xe4,2xef,2xeg,2xei,2xej,2xel,2xey,2xez,2xf0,2xfi,2xfj,2xfk,2xg3,2xg5
,2xg9,2xgm,2xgo,2xgs,2xh5,2xhm,2xhr,2xhs,2xht,2xhx,2xi7,2xib,2xii,2xix,2xi
y,2xiz,2xj0,2xj1,2xj2,2xj7,2xjg,2xjj,2xjx,2xk1,2xk3,2xk4,2xk6,2xk7,2xk8,2x
k9,2xkc,2xkd,2xke,2xkf,2xl2,2xl3,2xlc,2xln,2xm1,2xm2,2xm8,2xm9,2xml,2xmy,2
xn3,2xn5,2xn6,2xn7,2xnb,2xne,2xng,2xni,2xnm,2xnn,2xno,2xnp,2xo8,2xog,2xoi,
2xow,2xp2,2xp3,2xp4,2xp5,2xp6,2xp7,2xp8,2xpa,2xpb,2xpc,2xpk,2xqq,2xru,2xrw
,2xs0,2xs8,2xsb,2xtk,2xtn,2xto,2xu1,2xu3,2xu4,2xu5,2xuc,2xuf,2xui,2xup,2xu
z,2xv1,2xvd,2xvn,2xwd,2xwe,2xwy,2xx2,2xx4,2xx5,2xxn,2xxr,2xxt,2xxw,2xxx,2x
xy,2xy9,2xyd,2xye,2xyf,2xyn,2xyr,2xys,2xyt,2xyu,2xz5,2xzc,2xzg,2xzq,2xzw,2
y06,2y07,2y0j,2y1d,2y1g,2y1n,2y1o,2y1w,2y1x,2y2h,2y2i,2y2j,2y2k,2y2n,2y2p,
2y34,2y36,2y3p,2y4a,2y4k,2y4l,2y4m,2y4s,2y54,2y55,2y56,2y57,2y58,2y59,2y5f
,2y5g,2y5h,2y5k,2y5l,2y67,2y68,2y6c,2y6d,2y6o,2y6s,2y71,2y76,2y77,2y7i,2y7
k,2y7p,2y7w,2y7x,2y7z,2y80,2y81,2y82,2y8c,2y8i,2y8l,2y8o,2y8q,2y9g,2y9q,2y
a6,2ya7,2ya8,2yac,2yak,2yay,2yaz,2yb0,2yb9,2ybk,2ybp,2ybs,2ybt,2ybu,2yc0,2
yc3,2yc5,2ycf,2ych,2ycm,2ycq,2ycr,2ycs,2yde,2ydf,2ydi,2ydj,2ydk,2ydm,2ydo,
2ydt,2ydv,2ydw,2ye9,2yek,2yel,2yem,2yer,2yex,2yfa,2yfe,2yfx,2yg2,2yga,2yge
,2ygf,2ygu,2yhd,2yhw,2yhy,2yi0,2yi5,2yi7,2yig,2yim,2yiq,2yir,2yis,2yit,2yi
u,2yiv,2yiw,2yix,2yj2,2yj8,2yj9,2yjb,2yjc,2yjq,2yjw,2yjx,2yk1,2yk9,2ykb,2y
kc,2yke,2yki,2ykj,2ylc,2yln,2ylo,2ylp,2ylq,2ym3,2ym4,2ym5,2ym6,2ym7,2ym8,2
ymd,2yme,2ymt,2ynd,2yne,2ynn,2ynr,2yns,2yof,2yog,2yoh,2yoj,2yol,2ypi,2ypo,
2ypp,2yq6,2ywp,2yxj,2yz3,2z1w,2z3h,2z3z,2z4b,2z4o,2z4r,2z4w,2z4y,2z4z,2z50
,2z52,2z5o,2z5s,2z5t,2z60,2z6w,2z78,2z7g,2z7h,2z7i,2z7r,2z8e,2z92,2z94,2z9
7,2z9g,2za0,2za3,2za5,2zas,2zaz,2zb0,2zb1,2zbk,2zc9,2zcq,2zcr,2zcs,2zda,2z
dk,2zdl,2zdm,2zdn,2zdt,2zdx,2zdz,2ze1,2zfp,2zfs,2zft,2zg1,2zg3,2zga,2zgm,2
zgx,2zhd,2zif,2zir,2zis,2zit,2zjf,2zju,2zjv,2zjw,2zkj,2zlf,2zlg,2zm1,2zm3,
2zmd,2zmj,2zmm,2zn7,2zns,2znt,2znu,2zo3,2zof,2zoq,2zp0,2zpk,2zq0,2zq1,2zq2
,2zsc,2zu3,2zu4,2zu5,2zv2,2zv9,2zva,2zvj,2zwz,2zx5,2zx6,2zx7,2zx8,2zx9,2zx
a,2zxb,2zxd,2zxg,2zy1,2zyb,2zym,2zyn,2zz1,2zz2,2zz6,2zzu,3a1c,3a1d,3a1e,3a
1s,3a1t,3a29,3a2c,3a2o,3a3y,3a4o,3a4p,3a5y,3a6t,3a73,3a9i,3aaq,3aas,3aau,3
aav,3abt,3abu,3acl,3acw,3acx,3ad7,3ad8,3ads,3adt,3adu,3adv,3afk,3ag9,3agl,
3agm,3ah8,3ahn,3aho,3ai8,3aid,3aig,3aje,3al3,3alt,3ama,3amb,3amv,3anq,3anr
,3ans,3ant,3ao1,3ao2,3ao4,3ao5,3aox,3ap4,3ap7,3apc,3aqa,3aqt,3ara,3arb,3ar
d,3are,3arf,3arg,3arn,3arp,3arq,3arr,3art,3aru,3arv,3arw,3arx,3ary,3arz,3a
s0,3as1,3as2,3as3,3ask,3asl,3asx,3at1,3at3,3at4,3ati,3atk,3atl,3atm,3atp,3
atu,3atv,3atw,3au6,3av9,3ava,3avb,3avf,3avg,3avh,3avi,3avj,3avk,3avl,3avm,
3avn,3avz,3aw0,3ax5,3axk,3axm,3axz,3ay0,3ay9,3aya,3ayc,3ayd,3az8,3az9,3aza
,3azb,3b0w,3b1m,3b1t,3b1u,3b24,3b25,3b26,3b27,3b28,3b2q,3b2t,3b2w,3b3c,3b3
s,3b3w,3b3x,3b4f,3b4p,3b50,3b5j,3b5r,3b65,3b66,3b67,3b68,3b78,3b7i,3b7j,3b
7r,3b7u,3b82,3b8h,3b8q,3b8r,3b8z,3b92,3b95,3b9g,3b9s,3bar,3bb1,3bbb,3bbf,3
bbt,3bc3,3bc4,3bc5,3bcn,3bcs,3be2,3be9,3bea,3bel,3bet,3bex,3bf1,3bft,3bfu,
3bg8,3bgb,3bgc,3bgl,3bgm,3bgp,3bgq,3bgs,3bgz,3bh3,3bh8,3bh9,3bhb,3bho,3bhx
,3bi0,3bi1,3bi6,3bim,3biz,3bjc,3bjm,3bki,3bkk,3bkl,3bl0,3bl1,3bl2,3bl7,3bl
9,3bla,3blr,3bls,3blt,3blu,3bm6,3bm8,3bm9,3bmn,3bmo,3bmq,3bmy,3bpc,3bpr,3b
qc,3bqn,3br9,3bra,3brn,3bsc,3bt9,3btc,3bti,3btj,3btl,3btr,3bu1,3bu6,3bu8,3
buf,3bug,3buh,3bum,3bun,3buo,3buw,3bux,3bv2,3bv3,3bv9,3bva,3bvb,3bwf,3bwj,
3bwk,3bx5,3bxe,3bxf,3bxg,3bxh,3bxs,3bxz,3bym,3byo,3bys,3byu,3bz3,3bze,3bzf
,3bzi,3c0z,3c10,3c14,3c1k,3c1n,3c1x,3c2f,3c2o,3c2r,3c2u,3c39,3c3o,3c3q,3c3
r,3c43,3c45,3c49,3c4c,3c4e,3c4f,3c4h,3c52,3c56,3c5u,3c6t,3c6u,3c6w,3c72,3c
79,3c7n,3c7p,3c7q,3c84,3c88,3c89,3c8a,3c8b,3c8e,3c94,3c9e,3caj,3cbp,3cbs,3
ccb,3ccc,3ccn,3cct,3ccw,3ccz,3cd0,3cd5,3cd7,3cd8,3cda,3cdb,3cde,3ce0,3ce3,
3cen,3cf1,3cf8,3cf9,3cfn,3cfs,3cft,3cfv,3cgf,3cgo,3cgy,3ch9,3chc,3chd,3che

,3chf,3chg,3cho,3chp,3chq,3chr,3chs,3cib,3cic,3cid,3cii,3cj2,3cj3,3cj4,3cj
5,3cjf,3cjo,3ck7,3ck8,3ckb,3cke,3ckp,3ckr,3ckt,3ckz,3cl0,3cl2,3clp,3cm2,3c
m7,3cn0,3co9,3coh,3coj,3cow,3coy,3coz,3cp9,3cpb,3cpc,3cph,3cpj,3cqu,3cqw,3
cr4,3cr5,3cs7,3cs8,3csl,3cso,3cst,3cth,3ctj,3ctq,3ctr,3ctt,3cvk,3cwe,3cwj,
3cwk,3cx9,3cy2,3cyu,3cyw,3cyx,3cyy,3cyz,3cz1,3czv,3d04,3d0b,3d0e,3d14,3d1e
,3d1f,3d1g,3d1v,3d1x,3d1y,3d1z,3d20,3d25,3d27,3d28,3d2e,3d2r,3d2t,3d32,3d3
p,3d3x,3d45,3d4f,3d4l,3d4q,3d4y,3d4z,3d50,3d51,3d52,3d5m,3d62,3d67,3d6o,3d
6p,3d6q,3d78,3d7b,3d7d,3d7f,3d7g,3d7h,3d7k,3d7m,3d7z,3d83,3d8w,3d8y,3d8z,3
d91,3d94,3d9k,3d9l,3d9m,3d9n,3d9o,3d9p,3d9v,3d9z,3da6,3da9,3dab,3daj,3daz,
3db6,3db8,3dba,3dbd,3dbs,3dbu,3dc2,3dc3,3dcc,3dcq,3dcr,3dcs,3dct,3dcv,3dcw
,3dd0,3dd8,3dda,3ddb,3ddf,3ddg,3ddp,3ddq,3ddu,3deh,3dei,3dej,3dek,3dg8,3dg
a,3dgl,3dgn,3dgo,3dgq,3dhk,3di6,3diw,3djf,3djk,3djo,3djp,3djq,3djv,3djx,3d
k1,3dkf,3dkg,3dkj,3dla,3dln,3dm6,3dnd,3dne,3dng,3dnj,3dnt,3dog,3dow,3doy,3
doz,3dp0,3dp1,3dp2,3dp3,3dp4,3dp9,3dpc,3dpd,3dpe,3dpf,3dpk,3dpo,3drf,3drg,
3dri,3drp,3drr,3drs,3ds0,3ds1,3ds3,3ds4,3ds6,3ds9,3dst,3dsu,3dsz,3dt1,3dtc
,3du8,3dux,3duy,3dv1,3dv5,3dvp,3dwb,3dx0,3dx1,3dx2,3dx3,3dx4,3dxg,3dxh,3dx
j,3dxk,3dxm,3dy6,3dya,3dyo,3dz2,3dz4,3dz5,3dz6,3dzt,3e01,3e0p,3e0q,3e12,3e
16,3e1r,3e2m,3e37,3e3b,3e3c,3e3u,3e4a,3e51,3e5a,3e5u,3e62,3e63,3e64,3e6k,3
e6v,3e6y,3e73,3e7a,3e7b,3e7o,3e81,3e85,3e8n,3e8r,3e8u,3e90,3e92,3e93,3e9b,
3e9h,3e9i,3eax,3eb1,3ebb,3ebh,3ebi,3ebl,3ebo,3ebp,3ecn,3ed0,3ee2,3eeb,3efj
,3efk,3efr,3efs,3eft,3efw,3eg6,3egk,3egt,3ehn,3eht,3ehw,3ehx,3ehy,3eid,3ei
g,3eio,3ej1,3ej5,3ejp,3ejq,3ejr,3ejs,3ejt,3eju,3eka,3ekn,3eko,3ekp,3ekq,3e
kr,3eks,3ekt,3ekv,3ekw,3ekx,3eky,3el0,3el1,3el4,3el5,3el7,3el8,3el9,3elc,3
elj,3elm,3emg,3emh,3eml,3ens,3eoc,3eor,3eos,3eou,3eov,3eq7,3eq8,3eq9,3eqb,
3eql,3eqr,3eqs,3eqy,3er3,3er5,3erd,3erk,3ern,3ert,3ery,3esj,3ess,3et7,3eta
,3eu5,3eu7,3evc,3evd,3evf,3ew2,3ewc,3ewh,3ewj,3ewu,3ewz,3ex2,3ex3,3ex6,3ex
e,3exf,3exh,3exo,3eyd,3eyf,3eyg,3eyh,3eyl,3eys,3eyu,3ezr,3ezv,3f07,3f0r,3f
15,3f16,3f17,3f18,3f19,3f1a,3f2a,3f33,3f34,3f35,3f36,3f37,3f38,3f39,3f3a,3
f3c,3f3d,3f3e,3f3t,3f3u,3f3v,3f3w,3f48,3f5j,3f5k,3f5l,3f5p,3f66,3f68,3f69,
3f6e,3f6g,3f6h,3f70,3f78,3f7b,3f7g,3f7h,3f7i,3f7u,3f7z,3f80,3f81,3f82,3f88
,3f8c,3f8e,3f8f,3f8s,3f8w,3f9n,3f9w,3f9y,3fa3,3faa,3fal,3fas,3fat,3fbr,3fc
1,3fc2,3fc8,3fcb,3fcf,3fci,3fck,3fcl,3fcq,3fdm,3fdn,3fdt,3fe7,3fea,3fed,3f
ee,3feg,3fei,3fej,3ff3,3ff6,3ffg,3ffp,3ffu,3fgc,3fh5,3fh7,3fh8,3fhb,3fhe,3
fhr,3fi2,3fi3,3fj7,3fjg,3fjz,3fk1,3fkt,3fkv,3fl5,3fl8,3fl9,3fmq,3fmr,3fmz,
3fn0,3fnm,3fnu,3fpd,3fpm,3fq7,3fqa,3fqe,3fqh,3fqk,3fql,3fqs,3fr2,3fr4,3fr5
,3frg,3frz,3fsj,3fsm,3ft2,3ft3,3ft4,3ft5,3ft8,3ftq,3fts,3ftu,3ftv,3ftw,3ft
y,3ftz,3fu0,3fu3,3fu5,3fu6,3fuc,3fud,3fue,3fuf,3fuh,3fui,3fuj,3fuk,3ful,3f
um,3fun,3fup,3fur,3fuz,3fv1,3fv2,3fv3,3fv7,3fv8,3fvg,3fvh,3fvk,3fvl,3fvn,3
fw3,3fw4,3fwv,3fx6,3fxb,3fxv,3fxw,3fxz,3fy0,3fyj,3fyk,3fyz,3fz1,3fzc,3fzn,
3fzr,3fzs,3fzt,3fzy,3g08,3g0b,3g0c,3g0d,3g0e,3g0f,3g0g,3g0i,3g0w,3g15,3g19
,3g1d,3g1m,3g1v,3g2h,3g2i,3g2j,3g2k,3g2l,3g2n,3g2s,3g2t,3g2u,3g2v,3g2w,3g2
y,3g2z,3g30,3g31,3g32,3g34,3g35,3g3d,3g3m,3g3n,3g3r,3g42,3g45,3g4f,3g4g,3g
4i,3g4k,3g4l,3g58,3g5d,3g5k,3g5v,3g5y,3g6g,3g6h,3g6m,3g6z,3g70,3g72,3g76,3
g7l,3g86,3g8e,3g8i,3g8o,3g90,3g9e,3g9l,3g9n,3ga5,3gb2,3gba,3gbb,3gbe,3gbq,
3gc4,3gc5,3gc7,3gcp,3gcq,3gcs,3gcu,3gcv,3gds,3gdt,3ge7,3gen,3gep,3gf2,3gfe
,3gfw,3ggc,3ggj,3ggu,3ggv,3ggw,3ghe,3gi4,3gi5,3gi6,3gjd,3gjq,3gjs,3gjt,3gj
w,3gk1,3gk2,3gk4,3gkz,3gl6,3gm0,3gn7,3gnv,3gnw,3gol,3gp0,3gpe,3gpj,3gpo,3g
qo,3gqz,3gr2,3grj,3gs6,3gs7,3gsg,3gsm,3gss,3gst,3gt9,3gta,3gtc,3gur,3gus,3
guz,3gv6,3gv9,3gvb,3gvu,3gw5,3gws,3gwt,3gwu,3gwv,3gww,3gwx,3gx0,3gxl,3gxt,
3gxy,3gxz,3gy2,3gy3,3gy4,3gy7,3gyn,3gz9,3gzn,3h03,3h06,3h0a,3h0b,3h0e,3h0j
,3h0q,3h0s,3h0v,3h0w,3h0y,3h0z,3h1x,3h1z,3h21,3h22,3h23,3h26,3h2a,3h2c,3h2
f,3h2m,3h2n,3h2o,3h30,3h3c,3h52,3h59,3h5b,3h5s,3h5u,3h6z,3h78,3h85,3h89,3h
8b,3h8c,3h91,3h98,3h9f,3h9k,3h9o,3ha6,3hab,3hac,3hau,3hav,3haw,3hb4,3hb8,3
hbo,3hc8,3hcm,3hd3,3hdk,3hdm,3hdn,3hdz,3hec,3heg,3hek,3hf6,3hf8,3hfb,3hfj,

3hfv,3hfz,3hg1,3hha,3hhk,3hhm,3hhu,3hig,3hii,3hik,3hio,3hit,3hiv,3hiw,3hj0
,3hjo,3hk1,3hkn,3hkq,3hkt,3hku,3hkw,3hky,3hl5,3hl7,3hl8,3hll,3hlo,3hmm,3hm
o,3hmp,3hmv,3hnb,3hng,3hnz,3ho2,3ho9,3hp2,3hp5,3hp9,3hpt,3hq5,3hqh,3hqr,3h
qw,3hqy,3hqz,3hr1,3hrb,3hrf,3hs4,3hs8,3hs9,3hu1,3hu2,3hu3,3hub,3huc,3hv3,3
hv4,3hv5,3hv6,3hv7,3hv8,3hvc,3hvg,3hvh,3hvi,3hvj,3hvk,3hw1,3hwn,3hww,3hwx,
3hx3,3hxb,3hxc,3hxd,3hxe,3hxf,3hxi,3hy5,3hy7,3hy9,3hyf,3hyg,3hzk,3hzm,3hzv
,3hzy,3i02,3i06,3i0r,3i0s,3i1y,3i25,3i28,3i3b,3i3d,3i4a,3i4b,3i4y,3i51,3i5
n,3i5r,3i5z,3i60,3i6c,3i6m,3i6o,3i6z,3i73,3i7b,3i7c,3i7e,3i7g,3i7i,3i81,3i
8t,3i90,3i91,3i97,3i9g,3ia6,3iad,3iae,3iaf,3iaw,3ibc,3ibi,3ibl,3ibn,3ibu,3
idp,3ie3,3iej,3ieo,3ies,3iet,3if7,3ifl,3ifo,3ifp,3ig1,3ig6,3ig7,3igb,3igg,
3igp,3igv,3ihz,3ii5,3iit,3iiw,3iiy,3ij0,3ij1,3ijg,3ijh,3ijy,3ijz,3ik1,3ik3
,3ika,3ikc,3ikd,3ikg,3il5,3il6,3ilq,3imc,3ime,3img,3imy,3in3,3in4,3ind,3in
e,3inf,3inh,3iny,3io7,3iob,3ioc,3iod,3ioe,3iof,3iog,3ioi,3iok,3ion,3iop,3i
p5,3ip6,3ip8,3ip9,3ipa,3ipb,3ipe,3iph,3ipq,3ips,3ipu,3ipx,3ipy,3iqg,3iqh,3
iqi,3iqj,3iqq,3iqu,3iqv,3irx,3is9,3isj,3iss,3isw,3itc,3ith,3itu,3itz,3iu7,
3iu8,3iu9,3iub,3iuc,3iue,3iut,3iux,3ivc,3ivg,3ivh,3ivi,3ivq,3ivv,3ivx,3iw4
,3iw5,3iw6,3iw7,3iw8,3iww,3iwy,3ixg,3ixj,3ixk,3jdw,3jpv,3jpx,3jq7,3jq8,3jq
9,3jqa,3jqb,3jqf,3jqg,3jrs,3jrx,3jsi,3jsw,3juk,3juo,3jup,3juq,3jvk,3jvr,3j
vs,3jwq,3jwr,3jxw,3jy0,3jy9,3jya,3jyj,3jyr,3jzb,3jzc,3jzf,3jzg,3jzh,3jzi,3
jzj,3jzk,3jzo,3jzp,3jzq,3jzr,3jzs,3k00,3k02,3k05,3k0h,3k0k,3k15,3k16,3k1j,
3k22,3k23,3k26,3k27,3k2f,3k37,3k39,3k3a,3k3b,3k3e,3k3g,3k3h,3k3i,3k3j,3k41
,3k48,3k4d,3k4q,3k54,3k5c,3k5d,3k5f,3k5g,3k5i,3k5k,3k5u,3k5v,3k5x,3k7f,3k8
3,3k84,3k8c,3k8d,3k8o,3k8q,3k97,3k98,3k99,3k9x,3ka2,3kab,3kac,3kad,3kaf,3k
ag,3kah,3kai,3kb3,3kb7,3kba,3kbz,3kc0,3kc1,3kc3,3kce,3kcf,3kck,3kd7,3kdb,3
kdc,3kdd,3kdm,3kdt,3kdu,3kec,3ked,3kej,3kek,3ken,3kf4,3kf7,3kfa,3kfc,3kfn,
3kga,3kgp,3kgq,3kgt,3kgu,3khj,3khv,3kid,3kig,3kiv,3kjd,3kjf,3kjn,3kjq,3kku
,3kl6,3kl8,3km4,3kmc,3kme,3kmm,3kmx,3kmy,3kn0,3koo,3kpu,3kpv,3kpw,3kqa,3kq
b,3kqc,3kqd,3kqe,3kqm,3kqo,3kqp,3kqr,3kqs,3kqt,3kqw,3kqy,3kr0,3kr1,3kr2,3k
r4,3kr5,3kr8,3krd,3krj,3krl,3krr,3krw,3krx,3kry,3ksl,3ksq,3ktr,3kv2,3kvw,3
kvx,3kw9,3kwa,3kwb,3kwf,3kwj,3kwz,3kx1,3kxz,3kyf,3kyg,3kyq,3kyr,3kze,3l08,
3l0e,3l0k,3l0n,3l0v,3l13,3l16,3l17,3l1n,3l1s,3l2y,3l38,3l3a,3l3l,3l3m,3l3n
,3l3q,3l3x,3l3z,3l4t,3l4u,3l4v,3l4w,3l4x,3l4y,3l4z,3l54,3l58,3l59,3l5b,3l5
c,3l5d,3l5e,3l5f,3l5r,3l6f,3l6h,3l6x,3l79,3l7a,3l7b,3l7c,3l7d,3l7g,3l81,3l
8s,3l8v,3l8x,3l9h,3l9l,3l9m,3l9n,3lau,3lbj,3lbk,3lbl,3lbz,3lc3,3lc5,3lcd,3
lce,3lco,3lcu,3lcv,3ldp,3ldq,3ldw,3le6,3le8,3le9,3lea,3lf0,3lfn,3lfs,3lgl,
3lgp,3lgs,3lhg,3lhj,3lhs,3li2,3lik,3lil,3lir,3liw,3lj3,3lj7,3ljg,3ljj,3ljo
,3ljt,3ljz,3lk0,3lk1,3lk8,3lka,3lkh,3lkj,3lkz,3ll8,3lle,3llm,3lm1,3lmk,3lm
p,3lnj,3lnk,3lnz,3lok,3loo,3lox,3lp0,3lp1,3lp2,3lp4,3lp7,3lpb,3lpf,3lpg,3l
pi,3lpj,3lpk,3lpl,3lpp,3lpr,3lpt,3lpu,3lq2,3lq4,3lq5,3lq8,3lqi,3lqj,3lrh,3
ls4,3luo,3lvp,3lvw,3lw0,3lxe,3lxg,3lxk,3lxl,3lxo,3lxs,3ly2,3lzb,3lzs,3lzu,
3lzv,3lzz,3m11,3m17,3m1k,3m1s,3m2u,3m2w,3m35,3m36,3m37,3m3c,3m3e,3m3o,3m3r
,3m3x,3m3z,3m40,3m53,3m54,3m55,3m56,3m57,3m58,3m59,3m5a,3m5e,3m67,3m6f,3m6
p,3m6q,3m6r,3m89,3m8p,3m8q,3m8u,3m93,3m94,3m96,3m9f,3ma3,3mag,3mam,3max,3m
b6,3mb7,3mbl,3mbp,3mbz,3mct,3mdz,3me9,3mea,3met,3meu,3mf5,3mfv,3mfw,3mg0,3
mg4,3mg6,3mg7,3mg8,3mhc,3mhi,3mhl,3mhm,3mho,3mhw,3mi2,3mi3,3miy,3mj1,3mj2,
3mj5,3mjl,3mke,3mkf,3mkn,3mks,3ml2,3ml4,3ml5,3mlb,3mle,3mmf,3mmr,3mn8,3mna
,3mnu,3mo0,3mo2,3mo5,3mo8,3moe,3mof,3moh,3mp1,3mp6,3mpm,3mpt,3mqf,3mrt,3mr
v,3mrx,3ms2,3ms4,3ms7,3ms9,3msc,3msj,3msk,3msl,3mss,3mt7,3mt8,3mt9,3mta,3m
tb,3mtd,3mtw,3muf,3muk,3muz,3mv0,3mv5,3mvh,3mvj,3mvl,3mvm,3mw1,3mwe,3mwu,3
mww,3mxc,3mxd,3mxe,3mxf,3mxr,3mxs,3mxy,3my1,3my5,3myg,3myq,3mz3,3mz6,3mzc,
3n0h,3n0n,3n1c,3n1v,3n1w,3n23,3n2c,3n2e,3n2p,3n2u,3n2v,3n35,3n3g,3n3j,3n3l
,3n45,3n46,3n49,3n4b,3n4c,3n4l,3n51,3n5e,3n5h,3n5j,3n5k,3n5u,3n6k,3n75,3n7
6,3n7a,3n7h,3n7o,3n7r,3n7s,3n86,3n87,3n8k,3n8n,3n9r,3n9s,3nal,3nam,3nan,3n
b5,3nba,3nc4,3nc9,3ncg,3ncq,3ncr,3ncz,3ndm,3nee,3nef,3neg,3neo,3nes,3new,3

nex,3nf3,3nf6,3nf7,3nf8,3nf9,3nfk,3nfl,3ng4,3nga,3nhi,3nht,3ni5,3nif,3nii,
3nij,3nik,3nil,3nim,3nin,3njq,3njy,3nk8,3nkk,3nkx,3nlb,3nm6,3nmq,3nnu,3nnv
,3nnw,3nnx,3nok,3nox,3np7,3np9,3npa,3npc,3nq3,3nq9,3nrm,3nrz,3ns7,3ns9,3ns
h,3nsn,3nsq,3nth,3nti,3ntp,3nu3,3nu4,3nu5,3nu6,3nu9,3nuj,3nuo,3nus,3nuu,3n
uy,3nw3,3nw5,3nw6,3nw7,3nw9,3nwb,3nwe,3nww,3nx7,3nxq,3ny3,3nyd,3nyn,3nyx,3
nzc,3nzi,3nzk,3nzs,3nzu,3nzw,3nzx,3o0e,3o0g,3o0j,3o0u,3o1d,3o1e,3o1g,3o23,
3o2m,3o3j,3o4k,3o4l,3o56,3o57,3o5n,3o5x,3o64,3o6l,3o6m,3o6t,3o75,3o7u,3o84
,3o86,3o87,3o88,3o8g,3o8h,3o8p,3o95,3o96,3o99,3o9a,3o9b,3o9c,3o9d,3o9e,3o9
f,3o9g,3o9h,3o9i,3o9l,3o9p,3o9v,3oad,3oaf,3oag,3oap,3oaw,3oay,3ob0,3ob1,3o
b2,3obq,3obu,3obx,3oc0,3ocb,3ocg,3ocp,3oct,3ocz,3od0,3odi,3odk,3odl,3odu,3
oe0,3oe4,3oe5,3oe6,3oe8,3oe9,3oeu,3oev,3of8,3og7,3ogm,3ogp,3ogq,3ogx,3ohf,
3ohh,3ohi,3oik,3oil,3oim,3oj8,3ok9,3oka,3okc,3okh,3oki,3okp,3oku,3okv,3old
,3ole,3olf,3olg,3oli,3omc,3omg,3omm,3oob,3oof,3ook,3oot,3ooz,3opm,3opp,3op
r,3oq5,3oqf,3oqk,3orn,3os3,3osi,3osw,3ot3,3ot8,3otf,3otq,3otx,3ouh,3oui,3o
uj,3ov1,3ove,3ovn,3ovx,3ovz,3ow3,3ow4,3ow6,3owb,3owd,3owj,3owk,3owl,3own,3
oxc,3oxi,3oxz,3oy0,3oy1,3oy3,3oy8,3oyl,3oyn,3oyp,3oyq,3oys,3oyw,3oz1,3ozg,
3ozj,3ozp,3ozr,3ozs,3ozt,3p0g,3p17,3p1d,3p23,3p2e,3p2h,3p2k,3p3g,3p3h,3p3j
,3p3r,3p3s,3p3t,3p3u,3p44,3p4f,3p4q,3p4r,3p4v,3p4w,3p50,3p55,3p58,3p5k,3p5
l,3p5o,3p76,3p78,3p79,3p7a,3p7b,3p7c,3p7i,3p8e,3p8h,3p8n,3p8o,3p8p,3p8z,3p
9h,3p9j,3p9l,3p9m,3p9t,3pa3,3pa4,3pa5,3pa8,3pab,3pax,3pb3,3pb7,3pb8,3pb9,3
pbb,3pcb,3pcc,3pce,3pcf,3pcg,3pch,3pcj,3pck,3pcn,3pcu,3pd2,3pd3,3pd4,3pd8,
3pd9,3pdc,3pdh,3pdq,3pe1,3pe2,3peq,3pfp,3pgl,3pgu,3phe,3pi5,3pix,3piy,3piz
,3pj1,3pj2,3pj3,3pj8,3pjc,3pjg,3pjt,3pju,3pka,3pkb,3pkc,3pkd,3pke,3pkn,3pl
u,3pm1,3pma,3pn1,3pn3,3pn4,3po1,3po6,3poa,3poz,3pp0,3pp1,3pp7,3ppj,3ppk,3p
pm,3ppo,3ppp,3ppq,3ppr,3pqz,3pr0,3prf,3prs,3prz,3ps1,3ps6,3psb,3psd,3psl,3
ptg,3pty,3puj,3puk,3pup,3pvu,3pvw,3pwd,3pwh,3pwk,3pwm,3pww,3px8,3px9,3pxe,
3pxf,3pxq,3pxy,3pxz,3py0,3py1,3pyy,3pz1,3pz2,3pz3,3pz4,3q0z,3q1x,3q2a,3q2g
,3q2h,3q2j,3q2m,3q32,3q3b,3q3k,3q3t,3q43,3q44,3q4b,3q4c,3q4j,3q4k,3q4l,3q5
h,3q5u,3q6k,3q6s,3q6w,3q6z,3q71,3q72,3q77,3q7j,3q7p,3q7q,3q8d,3q8h,3q92,3q
96,3qa2,3qaa,3qai,3qak,3qaq,3qar,3qbc,3qbh,3qbn,3qc4,3qc9,3qce,3qcf,3qch,3
qci,3qcj,3qck,3qcl,3qcq,3qcs,3qcx,3qcy,3qd0,3qd3,3qd4,3qdd,3qel,3qem,3qfd,
3qfv,3qfy,3qfz,3qg6,3qgw,3qgy,3qi1,3qi3,3qi4,3qin,3qio,3qip,3qiy,3qiz,3qj0
,3qj9,3qk0,3qk5,3qkd,3qkk,3qkl,3qkm,3qkv,3ql9,3qlb,3qlc,3qlm,3qmk,3qn7,3qn
d,3qnj,3qo2,3qo3,3qo9,3qox,3qpn,3qpo,3qpp,3qps,3qqa,3qqk,3qqs,3qqu,3qri,3q
rj,3qrk,3qs1,3qs4,3qs5,3qs6,3qs8,3qsb,3qsd,3qt6,3qt7,3qtf,3qti,3qto,3qtq,3
qtr,3qts,3qtu,3qtv,3qtw,3qtx,3qtz,3qu0,3que,3qup,3qvu,3qvv,3qw5,3qw6,3qw7,
3qw8,3qwc,3qx5,3qx8,3qx9,3qxc,3qxd,3qxh,3qxm,3qxp,3qxt,3qxv,3qyy,3qzq,3qzt
,3qzv,3r00,3r01,3r02,3r04,3r0h,3r0i,3r0t,3r0w,3r0y,3r16,3r17,3r1v,3r21,3r2
2,3r24,3r2a,3r2b,3r2f,3r2y,3r42,3r4m,3r4n,3r4o,3r4p,3r5j,3r5m,3r5n,3r5t,3r
6c,3r6g,3r6t,3r6u,3r7b,3r7n,3r7o,3r7q,3r7r,3r88,3r8i,3r8u,3r8v,3r8z,3r91,3
r92,3r93,3r9d,3r9h,3r9n,3r9o,3rah,3rak,3ral,3rbm,3rbq,3rbu,3rcd,3rcj,3rde,
3rdh,3rdo,3rdq,3rdv,3re4,3rey,3rf4,3rf5,3rg2,3rhk,3rhx,3ri1,3rik,3ril,3rin
,3rj7,3rjc,3rjm,3rjw,3rk5,3rk7,3rk9,3rkb,3rkz,3rl3,3rl4,3rl7,3rl8,3rlb,3rl
p,3rlq,3rlr,3rm4,3rm8,3rm9,3rme,3rmf,3rni,3ro0,3ro4,3roc,3rpr,3rpv,3rpy,3r
q7,3rqe,3rqf,3rqg,3rqw,3rr4,3rsb,3rse,3rsr,3rsv,3rsx,3rt4,3rt6,3rt8,3rtf,3
rth,3rti,3rtm,3rtn,3rtp,3rtx,3ru1,3rul,3rum,3run,3rup,3rux,3rv3,3rv4,3rv6,
3rv7,3rv8,3rv9,3rvg,3rvi,3rw9,3rwc,3rwd,3rwe,3rwf,3rwg,3rwh,3rwi,3rwj,3rwp
,3rwq,3rx5,3rx7,3rx8,3rxa,3rxb,3rxd,3rxe,3rxf,3rxg,3rxh,3rxi,3rxj,3rxk,3rx
l,3rxm,3rxo,3rxp,3ry8,3ryj,3ryv,3ryw,3ryx,3ryy,3ryz,3rz0,3rz1,3rz3,3rz5,3r
z7,3rz8,3rz9,3rzb,3rzi,3s00,3s0b,3s0d,3s0e,3s0j,3s0n,3s0o,3s1g,3s1h,3s1y,3
s22,3s2a,3s2o,3s2p,3s2v,3s3i,3s3k,3s3m,3s3n,3s3o,3s3q,3s3r,3s3v,3s43,3s45,
3s4q,3s53,3s54,3s56,3s5y,3s68,3s6t,3s71,3s72,3s73,3s74,3s75,3s76,3s77,3s78
,3s7a,3s7b,3s7f,3s7l,3s7m,3s8l,3s8n,3s8o,3s8x,3s9e,3s9i,3s9t,3s9y,3s9z,3sa
d,3sap,3sax,3saz,3sb0,3sbh,3sbi,3sc1,3sd5,3sdg,3sdi,3sdk,3sfc,3sff,3sfg,3s

fh,3sfi,3sgt,3sgv,3sgx,3sh0,3sh1,3sha,3shb,3shc,3shj,3shv,3shy,3shz,3si3,3
si4,3sie,3sio,3sjf,3sji,3sjo,3sjt,3sk2,3ska,3skc,3ske,3skf,3skg,3skh,3skk,
3sl0,3sl1,3sl4,3sl5,3sl8,3slz,3sm0,3sm1,3sm2,3smq,3sn7,3sn8,3sna,3snb,3snc
,3snd,3sni,3snl,3so6,3so9,3soq,3sou,3sov,3sow,3spf,3spk,3sqq,3sr4,3srb,3sr
c,3srg,3srv,3st5,3st6,3std,3stj,3str,3su0,3su1,3su2,3su3,3su4,3su5,3su6,3s
ud,3sue,3suf,3sug,3sur,3sus,3sut,3suu,3suv,3suw,3sv2,3sv6,3sv7,3sv8,3sv9,3
svj,3svv,3sw2,3sw8,3sw9,3sww,3sx4,3sx9,3sxf,3sxu,3sym,3syr,3sz1,3sz9,3szb,
3szm,3t01,3t03,3t07,3t08,3t09,3t0b,3t0d,3t0l,3t0m,3t0t,3t0w,3t0x,3t19,3t1a
,3t1l,3t1m,3t1n,3t2c,3t2p,3t2q,3t2t,3t2v,3t2w,3t3c,3t3d,3t3e,3t3g,3t3h,3t3
i,3t3u,3t3v,3t3y,3t4h,3t4n,3t4p,3t4v,3t5i,3t5u,3t60,3t64,3t6b,3t6j,3t6r,3t
6y,3t70,3t7g,3t82,3t83,3t84,3t85,3t8s,3t8v,3t8w,3t9t,3ta0,3ta1,3tam,3tao,3
tay,3tb6,3tc5,3tcg,3tcp,3tct,3tcy,3td4,3tdc,3tdh,3tdj,3tdu,3tdz,3te5,3tf6,
3tf7,3tfk,3tfn,3tfp,3tfu,3tfv,3tg5,3tge,3tgs,3th0,3th8,3th9,3thb,3thd,3ti1
,3ti3,3ti4,3ti5,3ti6,3ti8,3tia,3tib,3tic,3tif,3tiw,3tiy,3tiz,3tjc,3tjd,3tj
h,3tjm,3tk2,3tkh,3tki,3tkm,3tku,3tkw,3tkz,3tl0,3tl5,3tlh,3tll,3tmk,3tn8,3t
ne,3tnh,3tpp,3tpr,3tpu,3tpx,3ts4,3tsd,3tsk,3tsz,3tt0,3tt4,3tti,3ttj,3ttm,3
ttn,3ttp,3ttz,3tu1,3tu7,3tu9,3tv4,3tv5,3tv6,3tv7,3tv8,3tvc,3tvl,3tvw,3tvx,
3twd,3twj,3twp,3twr,3tws,3twu,3twv,3tww,3twx,3txo,3ty0,3tyq,3tyv,3tz0,3tz2
,3tz4,3tza,3tzd,3tzm,3u0d,3u0p,3u0t,3u10,3u15,3u18,3u1i,3u1y,3u2k,3u2q,3u3
f,3u3u,3u3z,3u4h,3u4i,3u4o,3u4r,3u4u,3u4w,3u51,3u5j,3u5l,3u6a,3u6h,3u6i,3u
6j,3u6w,3u78,3u7k,3u7l,3u7m,3u7n,3u7s,3u81,3u8d,3u8h,3u8j,3u8k,3u8l,3u8m,3
u8n,3u8w,3u90,3u92,3u93,3u9c,3u9n,3u9q,3ua8,3ua9,3ual,3uat,3ubd,3ubx,3ucj,
3ud5,3ud7,3ud8,3ud9,3uda,3udd,3udh,3udj,3udk,3udm,3udn,3udp,3udq,3udr,3udv
,3udy,3ue4,3uec,3ued,3uef,3ueo,3ueu,3uev,3uew,3uex,3uf9,3ufa,3ufl,3ug2,3ug
c,3uh2,3uh4,3uhm,3ui2,3ui7,3uib,3uig,3uih,3uii,3uij,3uik,3uil,3uix,3uj9,3u
jb,3ujc,3ujd,3ukr,3uli,3umo,3ump,3umq,3umw,3umx,3unj,3unk,3unn,3unz,3uo4,3
uo5,3uo6,3uo9,3uod,3uoh,3uoj,3uok,3uol,3up2,3up7,3upf,3uph,3upi,3upk,3upv,
3upx,3upy,3upz,3uqf,3uqg,3uqp,3uqr,3ur0,3ur9,3uri,3usn,3usx,3ut5,3utu,3uu1
,3uug,3uuo,3uvk,3uvl,3uvm,3uvn,3uvo,3uvp,3uvq,3uvu,3uvw,3uvx,3uw4,3uw5,3uw
9,3uwk,3uwl,3uwo,3ux0,3uxd,3uxg,3uxk,3uxl,3uxm,3uyr,3uyt,3uz5,3uza,3uzc,3u
zd,3uzj,3uzp,3v01,3v04,3v0l,3v0p,3v1r,3v2n,3v2o,3v2p,3v2q,3v2w,3v2x,3v30,3
v31,3v3b,3v3l,3v3m,3v3q,3v3v,3v43,3v49,3v4j,3v4t,3v4v,3v4x,3v51,3v5g,3v5j,
3v5l,3v5p,3v5q,3v5t,3v66,3v6r,3v6s,3v78,3v7c,3v7d,3v7s,3v7t,3v7x,3v8s,3v8t
,3v8w,3v9b,3va4,3vap,3vb4,3vb5,3vb6,3vb7,3vbd,3vbg,3vbq,3vbt,3vbv,3vbw,3vb
x,3vby,3vc4,3vd4,3vd7,3vd9,3vdb,3vdc,3veh,3veu,3vf3,3vf5,3vf7,3vf8,3vf9,3v
fa,3vfb,3vfj,3vfq,3vg1,3vgc,3vh9,3vha,3vhc,3vhd,3vhe,3vhk,3vhu,3vhv,3vi2,3
vi5,3vi7,3vid,3vjc,3vje,3vjk,3vjl,3vjm,3vjs,3vjt,3vnt,3vo3,3voz,3vp1,3vp2,
3vp3,3vp4,3vqh,3vqs,3vqu,3vrt,3vru,3vrv,3vrw,3vry,3vs3,3vsw,3vsx,3vtb,3vtc
,3vtd,3vtr,3vuc,3vv6,3vv7,3vv8,3vva,3vvy,3vvz,3vw0,3vw1,3vw2,3vw6,3vw7,3vw
9,3vws,3vx3,3vyd,3vye,3vyf,3vzd,3vzg,3vzv,3w07,3w0l,3w1f,3w2o,3w2p,3w2q,3w
2r,3w2s,3w2t,3w32,3w33,3w37,3w54,3w55,3w5e,3w5n,3w5t,3w69,3w8o,3w9k,3w9r,3
wab,3wav,3waw,3wax,3way,3wb4,3wb5,3wbl,3wc5,3wc7,3wcb,3wcf,3wcg,3wch,3wci,
3wcl,3wd1,3wd2,3wd9,3wdc,3wdd,3wde,3wdz,3we4,3wf5,3wf6,3wf7,3wf8,3wf9,3wff
,3wfg,3wgg,3wgw,3wha,3whw,3wi2,3wi6,3wig,3wix,3wiy,3wiz,3wjw,3wk4,3wk5,3wk
6,3wk7,3wk8,3wk9,3wka,3wkb,3wkc,3wkd,3wke,3wmb,3wmc,3wnr,3wns,3wnt,3wp0,3w
p1,3wpn,3wq5,3wq6,3wqh,3wqm,3wqv,3wqw,3ws8,3ws9,3wsy,3wt5,3wt7,3wth,3wti,3
wtj,3wtk,3wtl,3wtm,3wtn,3wto,3wut,3wuu,3wuv,3wv1,3wv2,3wv3,3wvm,3wyj,3wyk,
3wyl,3wym,3wyx,3wyy,3wz6,3wz7,3wz8,3wzj,3wzk,3wzn,3wzp,3wzq,3wzu,3x00,3x1k
,3zbf,3zbx,3zc5,3zc6,3zcl,3zcw,3zcz,3zdg,3zdh,3zdv,3zeb,3zep,3zev,3zh8,3zh
a,3zhf,3zhx,3zhz,3zi0,3zi8,3zim,3zj6,3zj8,3zjc,3zjt,3zju,3zjv,3zk6,3zke,3z
kf,3zki,3zlk,3zll,3zln,3zlo,3zlq,3zlr,3zls,3zlv,3zlw,3zlx,3zly,3zm4,3zm5,3
zm6,3zm9,3zmg,3zmh,3zmi,3zmj,3zmm,3zmp,3zmq,3zmt,3zmu,3zmv,3zmz,3zn0,3zn1,
3znc,3znr,3zns,3zo1,3zo2,3zo4,3zos,3zot,3zov,3zp9,3zpq,3zpr,3zps,3zpt,3zpu
,3zq9,3zqe,3zqi,3zqt,3zrc,3zrk,3zrl,3zrm,3zs0,3zs1,3zso,3zsq,3zst,3zsw,3zs

x,3zsy,3zsz,3zt1,3zt2,3zt3,3zt4,3ztc,3ztd,3ztx,3zuk,3zv7,3zvt,3zvv,3zvw,3z
vy,3zw3,3zxe,3zxh,3zxr,3zxv,3zxz,3zy2,3zy5,3zya,3zyb,3zyf,3zyh,3zyr,3zyu,3
zze,3zzf,3zzh,456c,4a0j,4a16,4a1w,4a22,4a23,4a2a,4a4c,4a4e,4a4f,4a4g,4a4h,
4a4l,4a4o,4a4q,4a4v,4a4w,4a4x,4a50,4a51,4a6b,4a6c,4a6l,4a6s,4a6v,4a6w,4a7b
,4a7c,4a7i,4a7j,4a95,4a9c,4a9i,4a9m,4a9n,4a9r,4a9s,4a9t,4a9u,4aa0,4aa1,4aa
2,4aa4,4aa5,4aa7,4aac,4aaw,4ab8,4ab9,4aba,4abb,4abd,4abe,4abf,4abg,4abh,4a
bi,4abj,4abk,4abu,4abv,4ac3,4acc,4acd,4acf,4acg,4ach,4aci,4acm,4acu,4acx,4
ad2,4ad3,4ad6,4ael,4af3,4afe,4afg,4afh,4afj,4aft,4ag8,4agc,4agd,4agl,4agm,
4agn,4ago,4agp,4agq,4ah9,4ahr,4ahs,4ahu,4ahv,4ai5,4ai8,4aia,4aif,4aig,4aj1
,4aj2,4aj4,4aje,4aji,4ajk,4ajl,4ajn,4ajo,4ajw,4akn,4al4,4alg,4alu,4alv,4al
w,4alx,4aml,4amw,4amx,4amy,4amz,4an0,4an1,4an2,4an3,4an9,4anb,4anm,4anq,4a
nu,4anv,4anw,4anx,4aoc,4aof,4aoi,4aom,4ap0,4ap7,4aph,4apo,4app,4apr,4aq3,4
aq4,4aq6,4aqc,4aqh,4ara,4arb,4ark,4aro,4arw,4as9,4asd,4ase,4asj,4ask,4asy,
4at3,4at4,4at5,4att,4au7,4au8,4aua,4auj,4auy,4av0,4av4,4av5,4avg,4avh,4avi
,4avj,4avs,4avt,4avu,4avw,4aw5,4aw8,4awf,4awg,4awi,4awj,4awk,4awm,4awo,4aw
p,4awq,4ax9,4axa,4axd,4axm,4ay5,4ay6,4ayp,4ayq,4ayr,4ayt,4ayu,4ayv,4ayw,4a
yx,4ayy,4az0,4az2,4az3,4az5,4az6,4aza,4azb,4azc,4aze,4azf,4azg,4azi,4azp,4
azt,4azy,4b00,4b05,4b0b,4b0c,4b0g,4b0j,4b11,4b12,4b13,4b14,4b1c,4b1d,4b1j,
4b2d,4b2i,4b2l,4b32,4b33,4b34,4b35,4b3b,4b3c,4b3d,4b3u,4b4g,4b4m,4b4n,4b4q
,4b5b,4b5d,4b5s,4b5t,4b5w,4b60,4b6c,4b6e,4b6f,4b6o,4b6p,4b6q,4b6r,4b6s,4b6
u,4b70,4b71,4b72,4b73,4b74,4b76,4b77,4b78,4b7j,4b7n,4b7p,4b7q,4b7r,4b7z,4b
80,4b81,4b82,4b83,4b84,4b85,4b8o,4b8p,4b8y,4b95,4b9h,4b9k,4b9w,4b9z,4ba3,4
bae,4bah,4bak,4bam,4ban,4bao,4baq,4bb2,4bb4,4bb9,4bbe,4bbf,4bbg,4bbh,4bc5,
4bcb,4bcc,4bcd,4bcf,4bcg,4bch,4bci,4bcj,4bck,4bcm,4bcn,4bco,4bcp,4bcq,4bcs
,4bcw,4bd3,4bda,4bdb,4bdc,4bdd,4bde,4bdf,4bdg,4bdh,4bdi,4bdj,4bdk,4bds,4bd
t,4bea,4bek,4bf1,4bf6,4bfd,4bfp,4bfr,4bfy,4bfz,4bg1,4bg6,4bgg,4bgh,4bgk,4b
gm,4bgx,4bgy,4bh3,4bh4,4bhf,4bhi,4bhn,4bhz,4bi0,4bi1,4bi2,4bi6,4bi7,4bib,4
bic,4bid,4bie,4bio,4bis,4bj8,4bj9,4bjb,4bjc,4bjx,4bkj,4bks,4bkt,4bky,4bkz,
4blb,4bnt,4bnu,4bnv,4bnx,4bny,4bnz,4bo0,4bo1,4bo2,4bo3,4bo4,4bo5,4bo6,4bo7
,4bo8,4bo9,4bpi,4bpj,4bps,4bqg,4bqh,4bqs,4bqt,4bqw,4bqx,4bqy,4br3,4brx,4bs
0,4bs4,4bs5,4bsq,4bt3,4bt4,4bt5,4bt9,4btb,4bti,4btk,4btl,4btm,4btt,4btu,4b
tw,4btx,4bty,4bup,4buq,4bv2,4bvb,4bw1,4bw2,4bw3,4bw4,4bxk,4bxn,4bxu,4byi,4
byj,4bzn,4bzo,4bzr,4bzs,4c0r,4c16,4c1c,4c1d,4c1e,4c1f,4c1g,4c1h,4c1m,4c1t,
4c1u,4c1w,4c1y,4c2v,4c35,4c36,4c37,4c38,4c3k,4c4e,4c4f,4c4g,4c4h,4c4i,4c4j
,4c4n,4c52,4c5d,4c5w,4c61,4c66,4c68,4c6u,4c6v,4c6x,4c6z,4c70,4c71,4c72,4c7
3,4c7t,4c8r,4c94,4c9w,4c9x,4ca4,4ca5,4ca6,4ca7,4ca8,4cae,4caf,4cbt,4cby,4c
c2,4cc3,4cc5,4cc6,4cc7,4ccd,4cd0,4cd1,4cd4,4cd5,4cd6,4cd8,4cdr,4ce1,4ce2,4
ce3,4ceb,4cfe,4cff,4cfl,4cfm,4cft,4cfu,4cfv,4cfw,4cfx,4cg8,4cg9,4cga,4cgi,
4cgj,4ch2,4ch8,4ci1,4ci2,4ci3,4cig,4cik,4civ,4ciw,4cix,4ciy,4ciz,4cj4,4cjn
,4cjp,4cjq,4cjr,4ck3,4cki,4ckj,4ckr,4cku,4cl6,4cl9,4clb,4cli,4clj,4clp,4cl
z,4cmo,4cmt,4cmu,4cnh,4cp5,4cp7,4cpq,4cpr,4cps,4cpt,4cpu,4cpw,4cpx,4cpy,4c
pz,4cq0,4cqe,4cqf,4cqg,4cr5,4cr9,4cra,4crb,4crc,4crd,4crf,4crj,4crl,4cs9,4
csd,4csj,4css,4cst,4csy,4ctj,4ctk,4cts,4cu1,4cu7,4cu8,4cwb,4cwf,4cwn,4cwo,
4cwp,4cwq,4cwr,4cws,4cwt,4cxw,4cxx,4cxy,4cy1,4czs,4d08,4d09,4d0w,4d0x,4d1a
,4d1b,4d1c,4d1d,4d1j,4d1s,4d1y,4d2d,4d2p,4d2r,4d2s,4d2t,4d2v,4d2w,4d3h,4d4
d,4d52,4d62,4d63,4d7b,4d83,4d85,4d88,4d89,4d8a,4d8c,4d8e,4d8i,4d8n,4d8s,4d
8z,4d9p,4da5,4daf,4dai,4daw,4db7,4dbm,4dbn,4dcd,4dce,4dcs,4dcv,4dcx,4dcy,4
ddh,4ddk,4ddl,4ddm,4dds,4ddy,4de0,4de1,4de2,4de3,4de5,4de7,4dea,4deb,4ded,
4deg,4deh,4dei,4del,4dem,4der,4des,4det,4deu,4dew,4dfb,4dff,4dfg,4dfl,4dfn
,4dfu,4dfw,4dgb,4dgg,4dgm,4dgn,4dgr,4dh6,4dhf,4dhl,4dhm,4dhn,4dho,4dhp,4dh
q,4dhr,4dhs,4dht,4dhu,4di2,4dij,4dj7,4djh,4djo,4djp,4djq,4djr,4djs,4dju,4d
jv,4djw,4djx,4djy,4dk5,4dk7,4dk8,4dko,4dkp,4dkq,4dkr,4dkt,4dld,4dli,4dlj,4
dma,4dmn,4dmw,4dmy,4dn0,4do3,4do4,4do5,4dow,4dpf,4dpi,4dpt,4dpu,4dpy,4dq2,
4dr9,4drk,4drm,4drn,4dro,4drp,4dru,4ds1,4dst,4dsu,4dsy,4dt2,4dt6,4dtk,4dtt

,4du8,4duh,4dum,4dus,4dv8,4dv9,4dve,4dvf,4dvi,4dwb,4dwg,4dwk,4dx9,4dxg,4dx
j,4dy6,4dzw,4dzy,4e0w,4e0x,4e1e,4e1k,4e1m,4e1n,4e1z,4e20,4e26,4e28,4e34,4e
35,4e3b,4e3d,4e3f,4e3g,4e3h,4e3i,4e3j,4e3k,4e3l,4e3m,4e3n,4e3o,4e49,4e4a,4
e4l,4e4n,4e4x,4e5d,4e5f,4e5g,4e5h,4e5i,4e5j,4e5w,4e67,4e6c,4e6d,4e6q,4e70,
4e7r,4e81,4e8w,4e8y,4e8z,4e90,4e93,4e96,4e9c,4e9d,4e9u,4ea1,4ea2,4ea3,4ear
,4eb8,4eb9,4ebv,4ebw,4ec0,4ec4,4ede,4edu,4edy,4edz,4ee0,4eeh,4eej,4eev,4ef
4,4ef6,4efg,4efk,4efs,4eft,4efu,4eg4,4eg5,4eg6,4eg7,4ega,4egh,4egi,4egk,4e
h2,4eh3,4eh4,4eh5,4eh6,4eh7,4eh8,4eh9,4ehe,4ehg,4ehm,4ehr,4ehv,4ehz,4ei4,4
ej2,4ej8,4ejf,4ejl,4ejn,4ek9,4eke,4ekg,4eki,4eky,4el0,4el5,4el9,4elb,4ele,
4elf,4elg,4elh,4em7,4emf,4emr,4emt,4emv,4en4,4enx,4eny,4eo4,4eo6,4eo8,4eoh
,4eoi,4eok,4eol,4eon,4eop,4eor,4eos,4eox,4eoy,4ep2,4epy,4eqc,4eqf,4eqj,4er
1,4er2,4er4,4ere,4erf,4erk,4erq,4erw,4ery,4erz,4es0,4esg,4esi,4est,4etz,4e
u0,4eu3,4euc,4euo,4euv,4ew2,4ew3,4ewh,4ewn,4ewo,4ewr,4exg,4exh,4exs,4exz,4
eyj,4eym,4eyr,4ez3,4ez5,4ezj,4ezk,4ezl,4ezo,4ezq,4ezr,4ezt,4ezw,4ezx,4ezy,
4ezz,4f08,4f09,4f0c,4f14,4f1l,4f1q,4f1s,4f20,4f2w,4f39,4f3c,4f3h,4f3i,4f3k
,4f49,4f4p,4f5y,4f63,4f64,4f65,4f6s,4f6u,4f6v,4f6w,4f6x,4f70,4f7j,4f7l,4f7
n,4f7v,4f8h,4f8j,4f9g,4f9u,4f9v,4f9w,4f9y,4fab,4fai,4fak,4fbe,4fbx,4fc0,4f
cb,4fcd,4fcf,4fci,4fck,4fcm,4fcq,4fcr,4fe6,4fe9,4fea,4fem,4feq,4fev,4few,4
ff8,4ffs,4fgt,4fgx,4fgy,4fgz,4fhh,4fhi,4fht,4fi9,4fic,4fil,4fiv,4fjz,4fk6,
4fk7,4fkk,4fl1,4fl2,4fl3,4flh,4fli,4flj,4flk,4fll,4flp,4fm7,4fm8,4fmn,4fmo
,4fmq,4fmu,4fn5,4fnn,4fns,4fny,4fnz,4fob,4foc,4fod,4fp1,4fpf,4fpk,4fqo,4fr
3,4fri,4frj,4frk,4frs,4fs3,4fs4,4fse,4fsl,4ft2,4fut,4fvq,4fvr,4fxf,4fxj,4f
xp,4fxq,4fxy,4fxz,4fyh,4fyo,4fys,4fz3,4fz6,4fzc,4fzg,4fzj,4g0a,4g0c,4g0k,4
g0l,4g0p,4g0q,4g0y,4g0z,4g11,4g16,4g17,4g19,4g1f,4g2f,4g2j,4g2l,4g2r,4g2w,
4g2y,4g31,4g34,4g3e,4g3f,4g3g,4g4p,4g55,4g5f,4g5y,4g68,4g69,4g8l,4g8m,4g8n
,4g8o,4g8r,4g8v,4g8y,4g90,4g93,4g95,4g9c,4ga3,4gah,4gao,4gb9,4gbd,4gby,4gb
z,4gcj,4gd6,4gdy,4ge1,4ge2,4ge4,4ge5,4ge6,4ge7,4ge9,4gee,4gfd,4gfm,4gfn,4g
fo,4gg5,4gg7,4ggl,4ggz,4gh6,4ghi,4ght,4gid,4gih,4gii,4giu,4gj2,4gj3,4gj6,4
gj7,4gj8,4gj9,4gja,4gjb,4gjc,4gjd,4gk2,4gk3,4gk4,4gk7,4gkc,4gkh,4gki,4gkm,
4glr,4glw,4glx,4gly,4gm3,4gm8,4gmc,4gmy,4gne,4gnf,4gng,4gny,4gpk,4gpl,4gq4
,4gq6,4gql,4gqp,4gqq,4gqr,4gr0,4gr3,4gr8,4gs6,4gs8,4gs9,4gsc,4gsy,4gtm,4gt
o,4gtp,4gtq,4gtr,4gts,4gtt,4gtv,4gu6,4gu9,4gue,4gui,4guj,4gv1,4gv8,4gvc,4g
vd,4gvm,4gvu,4gw1,4gw5,4gw6,4gw8,4gwi,4gwk,4gxl,4gxs,4gy5,4gye,4gz3,4gzf,4
gzp,4gzt,4gzw,4gzx,4h1e,4h1j,4h1m,4h2j,4h2m,4h2o,4h36,4h38,4h39,4h3a,4h3b,
4h3c,4h3f,4h3g,4h3i,4h3j,4h3q,4h42,4h4b,4h4d,4h4e,4h4m,4h58,4h5c,4h5d,4h5e
,4h71,4h75,4h7q,4h81,4h85,4ha5,4hai,4hbm,4hbn,4hbp,4hbv,4hbw,4hbx,4hby,4hc
o,4hct,4hcu,4hcv,4hcz,4hdb,4hdc,4hdf,4hdp,4he9,4heg,4hej,4heu,4hev,4hf4,4h
fp,4hfz,4hg7,4hgc,4hge,4hgl,4hgs,4hgt,4hhy,4hhz,4hiq,4his,4hj2,4hki,4hkk,4
hkn,4hkp,4hl5,4hla,4hlc,4hld,4hlf,4hlg,4hlh,4hlk,4hlm,4hlw,4hmh,4hmk,4hmq,
4hn2,4hnc,4hnf,4hni,4hnn,4hnp,4hod,4hp0,4hpi,4hpy,4hra,4hrc,4hrd,4hs6,4hs8
,4hso,4ht0,4ht2,4ht6,4htp,4htx,4hu1,4huo,4hup,4hv3,4hv7,4hva,4hvb,4hvd,4hv
g,4hvh,4hvi,4hvs,4hw2,4hw3,4hw7,4hwb,4hwo,4hwp,4hwr,4hws,4hwt,4hww,4hxj,4h
xl,4hxm,4hxq,4hxr,4hxs,4hxw,4hxz,4hy0,4hy1,4hy4,4hy5,4hy9,4hyb,4hyf,4hyh,4
hyi,4hym,4hys,4hyu,4hz5,4hze,4hzm,4hzt,4hzw,4hzx,4hzz,4i06,4i0d,4i0f,4i0r,
4i0s,4i0t,4i0z,4i10,4i11,4i12,4i1c,4i1r,4i2w,4i2z,4i31,4i32,4i33,4i3z,4i47
,4i4e,4i4f,4i54,4i5c,4i5h,4i5m,4i5p,4i60,4i67,4i6b,4i6f,4i6h,4i6q,4i71,4i7
2,4i73,4i74,4i7b,4i7c,4i7d,4i7f,4i7j,4i7k,4i7l,4i7m,4i7p,4i80,4i8n,4i8w,4i
8x,4i8z,4i9c,4i9h,4i9i,4i9o,4i9r,4i9s,4i9u,4i9z,4ia0,4iaw,4iax,4ib5,4ibb,4
ibc,4ibd,4ibe,4ibf,4ibg,4ibi,4ibj,4ibk,4ibm,4idn,4ido,4idt,4idv,4idz,4ie0,
4ie2,4ie3,4ie4,4ie5,4ie6,4ie7,4ieh,4ifh,4ifi,4igk,4igq,4igr,4igt,4ih3,4ih5
,4ih6,4ih7,4iho,4ii9,4iic,4iid,4iie,4iif,4ij1,4ijh,4ijl,4ijp,4ijq,4ikn,4ik
r,4iks,4ikt,4iku,4im0,4imq,4imz,4in9,4inb,4inh,4inr,4int,4inu,4io2,4io3,4i
o4,4io5,4io6,4io7,4io8,4ipf,4ipi,4ipj,4ipn,4iq6,4iqt,4iqu,4ir5,4ir6,4irx,4
is6,4ish,4isi,4isu,4ith,4iti,4itj,4itp,4iu0,4iu1,4iu4,4iue,4iuo,4iur,4iut,

4iuu,4iuv,4iva,4ivb,4ivc,4ivd,4ivk,4ivs,4ivt,4iwd,4iwz,4ixh,4ixu,4ixv,4iz0
,4izm,4izy,4j03,4j04,4j06,4j08,4j09,4j0a,4j0p,4j0r,4j0s,4j0t,4j0v,4j0y,4j0
z,4j17,4j1c,4j1e,4j1f,4j1h,4j1i,4j1k,4j1p,4j21,4j22,4j24,4j26,4j28,4j2c,4j
2t,4j3d,4j3e,4j3i,4j3j,4j3l,4j3m,4j3u,4j44,4j45,4j46,4j47,4j48,4j4n,4j4o,4
j4v,4j51,4j52,4j53,4j58,4j59,4j5b,4j5c,4j5d,4j5e,4j5p,4j6i,4j70,4j73,4j74,
4j77,4j78,4j79,4j7d,4j7e,4j7i,4j81,4j82,4j84,4j86,4j8b,4j8g,4j8m,4j8r,4j8s
,4j8t,4j93,4j9a,4jai,4jaj,4jal,4jaz,4jbl,4jbo,4jbp,4jbs,4jc1,4jck,4jda,4jd
f,4je7,4je8,4jfd,4jfe,4jff,4jfi,4jfj,4jfk,4jfl,4jfm,4jfs,4jft,4jfv,4jfw,4j
fx,4jfz,4jg0,4jg1,4jg6,4jg7,4jg8,4jgv,4jh0,4jhq,4jht,4jhz,4jia,4jib,4jik,4
jin,4jit,4jiz,4jj7,4jj8,4jje,4jjf,4jjg,4jjm,4jjq,4jjs,4jju,4jk5,4jk6,4jkt,
4jkw,4jlg,4jlh,4jlj,4jlm,4jln,4jls,4jmg,4jmh,4jmu,4jmx,4jn2,4jn4,4jnc,4jne
,4jnj,4jnm,4joa,4joe,4jof,4jog,4joh,4joj,4jok,4joo,4jp9,4jpa,4jpc,4jpe,4jp
s,4jpx,4jpy,4jq7,4jq8,4jql,4jr0,4jr3,4jr5,4jrg,4jrv,4jsa,4jsc,4jsr,4jss,4j
sz,4jt8,4jt9,4ju3,4ju4,4ju6,4ju7,4jv6,4jv7,4jv8,4jv9,4jvb,4jve,4jvi,4jvj,4
jvq,4jwk,4jwr,4jx7,4jx9,4jxs,4jxv,4jxw,4jyb,4jyc,4jym,4jyt,4jyu,4jyv,4jz1,
4jzb,4jzd,4jze,4jzf,4jzi,4jzr,4k0o,4k0u,4k0y,4k10,4k18,4k19,4k1b,4k1e,4k2f
,4k2g,4k2y,4k3h,4k3k,4k3l,4k3m,4k3n,4k3o,4k3p,4k3q,4k3r,4k42,4k43,4k4e,4k4
f,4k4j,4k55,4k5l,4k5m,4k5n,4k5o,4k5p,4k5y,4k5z,4k60,4k63,4k64,4k66,4k67,4k
69,4k6i,4k6t,4k6u,4k6v,4k6w,4k6y,4k6z,4k72,4k75,4k76,4k77,4k78,4k7i,4k7n,4
k7o,4k8a,4k8o,4k8s,4k9g,4k9h,4k9y,4kab,4kai,4kao,4kax,4kb7,4kb8,4kb9,4kba,
4kbc,4kbi,4kbk,4kby,4kc1,4kc2,4kc4,4kcg,4kcx,4ke0,4ke1,4keq,4kfp,4kfq,4kif
,4kii,4kij,4kil,4kin,4kio,4kip,4kiq,4kiu,4kiw,4kju,4kjv,4klb,4kln,4klv,4km
0,4km2,4kmd,4kmu,4kmz,4kn0,4kn1,4kn2,4kn4,4kn7,4knb,4kne,4kni,4knj,4knm,4k
nn,4knr,4knx,4ko8,4kod,4kom,4kon,4kot,4kov,4kow,4kox,4kp0,4kp4,4kp5,4kp6,4
kp8,4kpx,4kpz,4kql,4kqo,4kqp,4kqq,4kqr,4krs,4ks1,4ks2,4ks3,4ks4,4ks5,4ksp,
4ksq,4ksy,4ktc,4ktu,4kup,4kv9,4kva,4kvm,4kw6,4kwf,4kwg,4kwo,4kwp,4kww,4kx8
,4kxb,4kxl,4kxm,4kxn,4kyh,4kyk,4kz0,4kz3,4kz4,4kz5,4kz6,4kz7,4kz8,4kza,4kz
b,4kzc,4kzl,4kzq,4kzu,4l02,4l09,4l0b,4l0i,4l0l,4l0s,4l0t,4l0v,4l10,4l19,4l
1a,4l1u,4l23,4l2f,4l2g,4l2k,4l2l,4l2x,4l31,4l32,4l33,4l34,4l3o,4l3p,4l4l,4
l4m,4l4v,4l4z,4l50,4l51,4l52,4l53,4l58,4l5j,4l6q,4l6s,4l6t,4l70,4l7b,4l7c,
4l7d,4l7f,4l7g,4l7h,4l7j,4l7l,4l7n,4l7o,4l7r,4l7u,4l8m,4l9i,4la7,4lar,4lbl
,4lbo,4lbp,4lbu,4lc7,4lch,4led,4len,4leq,4lg6,4lge,4lgg,4lgh,4lgu,4lh2,4lh
3,4lh5,4lh6,4lh7,4lhm,4lhv,4li0,4li5,4li6,4li7,4li8,4lil,4lj3,4lj5,4lj8,4l
jh,4lk6,4lk7,4lkd,4lke,4lkf,4lkg,4lkh,4lkj,4lkk,4lkl,4lkm,4lko,4lkq,4lkt,4
ll3,4llj,4llk,4llp,4llx,4lm0,4lm1,4lm2,4lm3,4lm4,4lm5,4lmn,4lmu,4ln2,4ln7,
4lnb,4lnf,4lng,4lno,4lnp,4lnw,4loh,4loi,4loj,4loo,4lop,4loq,4lov,4loy,4lp0
,4lp6,4lp9,4lpb,4lpf,4lpg,4lph,4lps,4lq3,4lq9,4lqg,4lqy,4lrh,4lrr,4lsj,4lt
e,4lts,4luo,4luv,4luz,4lv1,4lv2,4lv3,4lv4,4lvt,4lw1,4lwc,4lwe,4lwh,4lwi,4l
wt,4lwu,4lwv,4lww,4lxa,4lxb,4lxd,4lxk,4lxm,4lxz,4ly1,4ly9,4lyn,4lys,4lyw,4
lzr,4lzs,4m0e,4m0f,4m0r,4m0y,4m0z,4m12,4m13,4m14,4m1d,4m1j,4m2r,4m2u,4m2v,
4m2w,4m3b,4m3d,4m3e,4m3f,4m3g,4m3m,4m3p,4m3q,4m48,4m4q,4m5g,4m5h,4m5i,4m5j
,4m5k,4m5l,4m5m,4m5n,4m5o,4m5r,4m5u,4m6p,4m6q,4m6u,4m7b,4m7c,4m7j,4m7x,4m7
y,4m84,4m8e,4m8h,4m8t,4m8x,4m8y,4man,4mao,4mb9,4mbc,4mbf,4mbi,4mbj,4mbl,4m
bp,4mc1,4mc2,4mc6,4mc9,4mcb,4mcc,4mcd,4mcv,4md6,4mdn,4mdq,4mdr,4mds,4mdt,4
men,4meo,4mep,4meq,4mex,4mf0,4mf1,4mfe,4mg5,4mg6,4mg7,4mg8,4mg9,4mga,4mgb,
4mgc,4mgd,4mgv,4mh7,4mha,4mho,4mhs,4mhy,4mhz,4mi3,4mi6,4mi9,4mib,4mic,4mji
,4mjo,4mjp,4mjq,4mjr,4mk0,4mk1,4mk2,4mk5,4mk7,4mk8,4mk9,4mka,4mlt,4mlx,4mm
4,4mm5,4mm6,4mm7,4mm8,4mm9,4mma,4mme,4mmf,4mmm,4mmp,4mn3,4mnp,4mnq,4mnv,4m
nw,4mnx,4mny,4mo4,4mo8,4mot,4mp2,4mp7,4mpc,4mpe,4mpn,4mq1,4mq2,4mq6,4mqp,4
mqu,4mr3,4mr4,4mr5,4mr6,4mra,4mrd,4mre,4mrf,4mrg,4mrh,4mro,4mrw,4mrz,4ms0,
4msa,4msc,4mse,4msg,4msk,4msl,4msn,4mss,4msu,4mt9,4mti,4mu7,4mue,4muf,4muk
,4mul,4muv,4muw,4mvh,4mvn,4mvw,4mvx,4mvy,4mw0,4mw1,4mw2,4mw4,4mw5,4mw6,4mw
7,4mw9,4mwb,4mwc,4mwe,4mwq,4mwr,4mwu,4mwv,4mww,4mwx,4mwy,4mx0,4mx1,4mx5,4m
x9,4mxa,4mxc,4my6,4myd,4myh,4myq,4mz4,4mz5,4mz6,4mzf,4mzh,4mzj,4mzk,4mzl,4

mzo,4mzs,4n00,4n07,4n1b,4n1t,4n1u,4n1z,4n3r,4n3w,4n4s,4n4t,4n4v,4n5d,4n5g,
4n5t,4n6g,4n6h,4n6y,4n6z,4n70,4n7e,4n7g,4n7h,4n7j,4n7m,4n7u,4n7y,4n84,4n8d
,4n8e,4n8q,4n8r,4n98,4n99,4n9a,4n9b,4n9c,4n9d,4n9e,4na4,4na7,4na8,4na9,4na
h,4nak,4nal,4nan,4nat,4nau,4nb3,4nb6,4nbk,4nbl,4nbn,4ncg,4ncm,4ncn,4nct,4n
du,4ng9,4nga,4ngh,4ngm,4ngn,4ngp,4ngq,4ngr,4ngs,4ngt,4nh7,4nh8,4nh9,4nhc,4
nhx,4nhy,4nie,4nj3,4nj9,4nja,4njd,4nk3,4nk9,4nka,4nks,4nkt,4nku,4nl1,4nld,
4nmo,4nmp,4nmq,4nmr,4nms,4nmt,4nmv,4nmx,4nni,4nnn,4nnr,4nnw,4no1,4no6,4no8
,4no9,4non,4np2,4np3,4np9,4npv,4nq6,4nra,4nrb,4nrc,4nrk,4nrl,4nrm,4nro,4nr
p,4nrq,4nrt,4nru,4ntj,4nuc,4nud,4nue,4nuf,4nus,4nvp,4nvq,4nw2,4nw5,4nw6,4n
w7,4nwc,4nwd,4nwk,4nwm,4nxo,4nxq,4nxr,4nxs,4nxu,4nxv,4ny3,4nyf,4nyi,4nyj,4
nym,4nyt,4nzb,4nzm,4nzn,4nzo,4o04,4o05,4o07,4o09,4o0a,4o0b,4o0j,4o0r,4o0t,
4o0v,4o0x,4o0y,4o0z,4o10,4o12,4o13,4o15,4o1b,4o1d,4o1l,4o24,4o28,4o2a,4o2b
,4o2c,4o2e,4o2f,4o2p,4o36,4o37,4o3a,4o3b,4o3c,4o3f,4o3t,4o3u,4o42,4o43,4o4
4,4o45,4o4g,4o4k,4o4r,4o4y,4o55,4o5b,4o5g,4o61,4o62,4o6e,4o6w,4o70,4o71,4o
72,4o74,4o75,4o76,4o77,4o78,4o7a,4o7b,4o7c,4o7d,4o7e,4o7f,4o91,4o97,4o9s,4
o9v,4o9w,4oag,4oak,4oar,4oas,4oaz,4ob0,4ob1,4ob2,4oba,4obo,4obp,4obq,4obv,
4obz,4oc0,4oc1,4oc2,4oc3,4oc4,4oc5,4oc6,4occ,4ock,4ocp,4ocq,4oct,4ocv,4ocx
,4ocz,4od0,4od7,4od9,4ode,4odf,4odk,4odl,4odm,4odn,4odp,4odq,4oee,4oef,4oe
g,4oel,4oem,4oeu,4oew,4oex,4ofb,4ofl,4og3,4og4,4og5,4og6,4og7,4og8,4ogi,4o
gj,4ogn,4ogt,4ogv,4ohk,4ohm,4oho,4ohp,4oi6,4oiv,4ojq,4ojr,4ok3,4ok5,4ok6,4
okg,4okp,4oks,4olc,4old,4olh,4oma,4omc,4omd,4omj,4omk,4ona,4onf,4ono,4oo9,
4ool,4oon,4oow,4op1,4op2,4op3,4oq3,4oq5,4oq6,4or0,4or4,4or6,4oru,4orx,4ory
,4os1,4os2,4os4,4os5,4os6,4os7,4osf,4ot6,4otf,4otg,4oth,4oti,4oty,4ou3,4ou
e,4ouj,4ov5,4ovf,4ovg,4ovh,4ovz,4ow0,4owm,4own,4owo,4owv,4oya,4oyb,4oyi,4o
yk,4oym,4oyo,4oyp,4oys,4oyt,4oz1,4oz2,4oz3,4ozj,4ozl,4ozn,4ozo,4p00,4p02,4
p0a,4p0b,4p0n,4p0v,4p0w,4p0x,4p10,4p1r,4p1u,4p2t,4p3h,4p44,4p45,4p4b,4p4d,
4p4e,4p4f,4p4i,4p4j,4p4s,4p4t,4p58,4p5d,4p5e,4p5z,4p6c,4p6e,4p6g,4p6w,4p6x
,4p72,4p73,4p74,4p75,4p7e,4p7m,4p7s,4p90,4pax,4pb1,4pb2,4pce,4pci,4pcs,4pc
t,4pd5,4pd6,4pd7,4pd8,4pd9,4pda,4pde,4pdk,4pee,4pf3,4pf5,4pft,4pfu,4pg3,4p
g9,4pgb,4pgc,4pgd,4pge,4pgh,4ph4,4phu,4phv,4phw,4pid,4pin,4pio,4piq,4pis,4
pji,4pjt,4pkb,4pkr,4pks,4pkt,4pku,4pkv,4pkw,4pl0,4pl3,4pl4,4pl5,4pl6,4pli,
4pm0,4pml,4pmm,4pmp,4pms,4pmt,4pn1,4pnc,4pni,4pnk,4pnl,4pnm,4pnn,4pnq,4pnr
,4pns,4pnt,4pnu,4pnw,4po0,4po7,4poh,4poj,4pop,4pov,4pow,4pox,4pp0,4pp3,4pp
5,4pp7,4pp9,4ppa,4ppb,4ppc,4pqa,4pqn,4pr5,4pra,4prb,4prd,4pre,4prg,4prh,4p
ri,4prj,4prn,4prp,4pry,4ps0,4ps1,4ps3,4ps5,4ps7,4ps8,4psb,4psh,4psq,4psx,4
ptc,4pte,4ptg,4puj,4puk,4pul,4pum,4puz,4pv0,4pv5,4pv7,4pvo,4pvt,4pvv,4pvx,
4pvy,4px6,4pxf,4pxm,4py1,4py2,4py4,4pyn,4pyo,4pyq,4pyv,4pyx,4pyy,4pz5,4pz8
,4pzh,4pzv,4pzw,4pzx,4q06,4q07,4q08,4q09,4q0a,4q0k,4q0l,4q15,4q18,4q19,4q1
a,4q1b,4q1c,4q1d,4q1e,4q1f,4q1n,4q1s,4q1w,4q1x,4q1y,4q2k,4q3q,4q3r,4q3s,4q
3t,4q3u,4q46,4q4e,4q4i,4q4o,4q4p,4q4q,4q4r,4q4s,4q6d,4q6e,4q6f,4q6r,4q7p,4
q7s,4q7v,4q7w,4q81,4q83,4q87,4q8x,4q8y,4q90,4q93,4q99,4q9m,4q9o,4q9s,4q9y,
4q9z,4qaa,4qab,4qac,4qag,4qb3,4qbb,4qbm,4qc1,4qd6,4qdk,4qem,4qer,4qev,4qew
,4qf7,4qf8,4qf9,4qfg,4qfl,4qfn,4qfo,4qfp,4qfr,4qfs,4qg7,4qga,4qgd,4qge,4qg
f,4qgg,4qgh,4qgi,4qh7,4qh8,4qhc,4qhp,4qht,4qij,4qip,4qir,4qiy,4qiz,4qj0,4q
jm,4qjo,4qjp,4qjr,4qjw,4qjx,4qk4,4qkd,4qkx,4ql1,4ql8,4qlk,4qll,4qlq,4qls,4
qlt,4qlu,4qlv,4qme,4qmm,4qmn,4qmo,4qmp,4qmq,4qms,4qmt,4qmu,4qmv,4qmw,4qmx,
4qmy,4qmz,4qn7,4qn9,4qna,4qnb,4qnu,4qo4,4qo9,4qoc,4qok,4qp1,4qp2,4qp6,4qp7
,4qp8,4qp9,4qpa,4qpd,4qpl,4qps,4qq4,4qq5,4qqc,4qqi,4qr3,4qr4,4qr5,4qrc,4qr
h,4qsh,4qsk,4qsm,4qsu,4qsv,4qsw,4qsx,4qt0,4qt1,4qt2,4qta,4qtb,4qtc,4qtd,4q
tl,4qtn,4quo,4qvl,4qvm,4qvn,4qvp,4qvq,4qvv,4qvw,4qvx,4qvy,4qw0,4qw1,4qw3,4
qw4,4qw5,4qw6,4qw7,4qwf,4qwg,4qwi,4qwj,4qwk,4qwl,4qwr,4qws,4qwu,4qwx,4qxj,
4qxo,4qxq,4qxr,4qxs,4qxt,4qy3,4qy8,4qye,4qyg,4qyh,4qyo,4qyy,4qz0,4qz1,4qz2
,4qz3,4qz4,4qz5,4qz6,4qz7,4qzs,4qzw,4qzx,4r02,4r06,4r07,4r0a,4r0i,4r17,4r1
8,4r1e,4r1v,4r1y,4r3b,4r3c,4r3s,4r3w,4r4c,4r4i,4r4o,4r4q,4r4t,4r59,4r5a,4r

5b,4r5g,4r5n,4r5t,4r5v,4r5w,4r5x,4r5y,4r6e,4r6t,4r6v,4r6w,4r6x,4r73,4r74,4
r75,4r76,4r7m,4r8y,4r91,4r92,4r93,4r95,4ra1,4ra5,4rab,4rac,4rad,4rak,4ran,
4rao,4raq,4rcd,4rce,4rcf,4rcg,4rcp,4rd0,4rd3,4rd6,4rdn,4re2,4re3,4re4,4re9
,4res,4ret,4rfc,4rfd,4rfm,4rfr,4rfy,4rfz,4rg0,4rgd,4rh5,4rhu,4rhx,4rhy,4ri
o,4ris,4riu,4riv,4rj3,4rj4,4rj5,4rj6,4rj7,4rj8,4rkx,4rlk,4rll,4rlp,4rlt,4r
lu,4rlw,4rme,4rn0,4rn1,4rn2,4rn4,4rn6,4rpn,4rpo,4rpv,4rqi,4rqk,4rqv,4rqz,4
rr6,4rra,4rrf,4rrg,4rrn,4rro,4rrq,4rrr,4rrs,4rrv,4rs0,4rsc,4rse,4rsk,4rsp,
4rss,4rt0,4rt1,4ruu,4rux,4ruy,4ruz,4rvk,4rvl,4rvm,4rvr,4rvt,4rwj,4rwk,4rwl
,4rww,4rx5,4rx7,4rx8,4rx9,4rxa,4rxc,4rxd,4rxe,4rxh,4rxz,4ryc,4ryd,4ryg,4ry
l,4rz1,4s1g,4s2i,4s3e,4s3f,4sga,4std,4tim,4tju,4tjw,4tjy,4tjz,4tk0,4tk1,4t
k2,4tk3,4tk4,4tk5,4tkb,4tkf,4tkg,4tkh,4tki,4tkj,4tkn,4tky,4tln,4tlr,4tmf,4
tmk,4tmn,4tmp,4tmr,4tn2,4tn4,4tnw,4to8,4tpk,4tpm,4tpp,4tpt,4tpw,4tq3,4tqn,
4trc,4trw,4trz,4ts1,4tsx,4tsz,4tt2,4tte,4ttv,4tu4,4tuh,4tun,4tv3,4tvj,4tw6
,4tw7,4tw8,4tw9,4twc,4twd,4twp,4twt,4tww,4twy,4tx6,4txc,4txe,4txs,4ty1,4ty
6,4ty7,4ty8,4ty9,4tya,4tyb,4tyl,4tyo,4tyt,4tz2,4tz8,4tzm,4tzn,4tzq,4u01,4u
03,4u0a,4u0b,4u0c,4u0d,4u0e,4u0f,4u0g,4u0i,4u0m,4u0n,4u0s,4u0u,4u0w,4u0x,4
u1b,4u2w,4u2y,4u3f,4u43,4u44,4u45,4u4s,4u4x,4u54,4u58,4u5j,4u5l,4u5n,4u5o,
4u5s,4u5t,4u5u,4u5v,4u68,4u69,4u6c,4u6e,4u6r,4u6w,4u6x,4u6y,4u6z,4u70,4u71
,4u73,4u79,4u7o,4u7q,4u7t,4u7v,4u82,4u8w,4u90,4u91,4u93,4ua8,4uac,4ual,4ua
t,4uau,4uc5,4ucc,4ucd,4uce,4uco,4ucr,4ucs,4uct,4ucu,4ucv,4ud7,4uda,4udb,4u
e1,4ufd,4ufe,4uff,4ufg,4ufh,4ufi,4ufj,4ufk,4ufl,4ufm,4ufu,4ufy,4ufz,4uhg,4
ui3,4ui4,4ui5,4ui6,4ui7,4ui8,4uia,4uib,4uil,4uin,4uit,4uiu,4uiv,4uiw,4uix,
4uiy,4uiz,4uj1,4uj2,4uj9,4uja,4ujb,4um1,4um3,4um9,4uma,4umb,4umc,4umj,4umn
,4umq,4umr,4umt,4umu,4und,4unn,4unp,4unq,4unr,4uns,4uof,4uoh,4up5,4ura,4ur
k,4url,4urm,4urn,4uro,4uru,4urv,4urw,4urx,4ury,4urz,4us3,4us4,4usi,4usj,4u
sw,4utn,4utr,4utv,4utx,4uu5,4uu7,4uu8,4uua,4uub,4uuh,4uuq,4uv8,4uv9,4uva,4
uvb,4uvc,4uvu,4uvv,4uvw,4uvx,4uvy,4uw1,4uwf,4uwg,4uwh,4uwk,4uwl,4ux4,4ux6,
4ux9,4uxb,4uxh,4uxj,4uxl,4uxq,4uy1,4uyd,4uye,4uyf,4uyg,4uyh,4uyn,4uzd,4uzh
,4v01,4v04,4v05,4v0i,4v11,4v1f,4v24,4v25,4v27,4vgc,4w4s,4w4v,4w4w,4w4x,4w4
y,4w4z,4w50,4w52,4w53,4w54,4w55,4w57,4w5a,4w5j,4w7p,4w7t,4w97,4w9c,4w9d,4w
9e,4w9f,4w9g,4w9h,4w9i,4w9j,4w9k,4w9l,4w9n,4w9o,4w9p,4w9q,4w9s,4w9w,4w9x,4
wa9,4waf,4wag,4wbg,4wbo,4wcf,4wci,4wcu,4wef,4wet,4wey,4wf2,4wf4,4wf6,4wgi,
4wh7,4wh9,4whh,4whl,4whq,4whr,4whs,4wht,4why,4whz,4wi1,4wiv,4wj5,4wj7,4wk1
,4wk2,4wk7,4wkb,4wkc,4wke,4wki,4wkn,4wko,4wkp,4wks,4wkt,4wku,4wkv,4wlb,4wm
9,4wmc,4wmu,4wmv,4wmx,4wmy,4wn0,4wn1,4wn5,4wnk,4wnm,4wno,4wnp,4wop,4wov,4w
p7,4wpf,4wph,4wpn,4wq2,4wq3,4wr7,4wr8,4wrb,4wri,4wrq,4wrs,4wsj,4wsk,4wsy,4
wt2,4wt6,4wtu,4wup,4wuy,4wv6,4wvl,4wvs,4wvt,4wvu,4ww6,4ww8,4wwn,4wwo,4wwp,
4wx4,4wx6,4wx7,4wxi,4wy1,4wy3,4wy6,4wy7,4wym,4wyo,4wyp,4wyy,4wyz,4wz4,4wz5
,4wz6,4wz8,4wzv,4x0f,4x0u,4x0z,4x11,4x13,4x14,4x1f,4x1n,4x1p,4x1q,4x1r,4x1
s,4x21,4x24,4x2i,4x2l,4x2s,4x34,4x3e,4x3h,4x3i,4x3k,4x3r,4x3s,4x3t,4x3u,4x
47,4x48,4x49,4x50,4x5p,4x5q,4x5r,4x5y,4x5z,4x60,4x61,4x63,4x68,4x69,4x6h,4
x6i,4x6j,4x6k,4x6m,4x6n,4x6o,4x6p,4x6s,4x6x,4x6y,4x7h,4x7i,4x7j,4x7k,4x7l,
4x7n,4x7o,4x7q,4x8g,4x8n,4x8o,4x8p,4x8s,4x8t,4x8u,4x8v,4x9r,4x9v,4x9w,4xaq
,4xar,4xas,4xbb,4xbd,4xbo,4xc2,4xcb,4xct,4xcu,4xdo,4xe0,4xe1,4xek,4xg3,4xg
4,4xg6,4xg7,4xg8,4xg9,4xgz,4xh2,4xh6,4xhe,4xhk,4xhl,4xhv,4xii,4xip,4xiq,4x
ir,4xit,4xj0,4xjr,4xjs,4xjt,4xk9,4xkb,4xkc,4xkx,4xm6,4xm7,4xm8,4xmb,4xmo,4
xmr,4xnv,4xnw,4xo8,4xoc,4xoe,4xpj,4xqa,4xqb,4xqu,4xrq,4xs2,4xsx,4xsy,4xsz,
4xt2,4xt9,4xta,4xtm,4xtp,4xtt,4xtv,4xtw,4xtx,4xty,4xtz,4xu0,4xu1,4xu2,4xu3
,4xua,4xub,4xuc,4xue,4xuh,4xum,4xuz,4xv1,4xv2,4xv3,4xv9,4xwk,4xx3,4xx4,4xx
9,4xxh,4xxs,4xy2,4xy8,4xy9,4xya,4xyc,4xyf,4xyn,4y0a,4y16,4y24,4y29,4y2b,4y
2j,4y2p,4y2q,4y2s,4y2t,4y2u,4y2v,4y2x,4y2y,4y32,4y38,4y3b,4y3j,4y3y,4y46,4
y4g,4y4j,4y4v,4y59,4y5d,4y5h,4y5i,4y5s,4y62,4y63,4y64,4y67,4y6m,4y6p,4y6r,
4y6s,4y73,4y79,4y7r,4y83,4y85,4y87,4y8c,4y8d,4y8x,4y8y,4y8z,4y94,4ya8,4yab
,4yad,4yas,4yat,4yax,4yay,4yb5,4yb6,4yb7,4ybi,4ybj,4ybk,4ybm,4ybs,4ybt,4yc

0,4yc8,4yc9,4ycl,4ycm,4ycn,4ycu,4ycv,4ycw,4ydf,4ydg,4ydn,4ydq,4ye3,4yec,4y
ee,4yef,4yes,4yff,4yfi,4ygf,4yh3,4yh4,4yha,4yhf,4yhm,4yho,4yhp,4yhq,4yht,4
yhz,4yih,4yik,4yje,4yjl,4yjn,4yk0,4yk5,4yk6,4ykj,4ykk,4ykn,4yl0,4yl1,4yl3,
4ylj,4ylk,4yll,4ylu,4ym2,4ym4,4ymb,4ymg,4ymh,4ymj,4yml,4ymq,4ymx,4ynb,4ynd
,4yne,4ynk,4ynl,4yo6,4yo8,4yog,4yoi,4yoj,4yoz,4yp1,4yp8,4ypf,4yps,4yqh,4yq
m,4yqu,4yqv,4yrc,4yrd,4yrg,4yrr,4yrs,4yrt,4ys7,4ysi,4ysl,4yt6,4yt7,4ytc,4y
tf,4yth,4yti,4yur,4yuw,4yux,4yuy,4yuz,4yv0,4yv1,4yv2,4yv5,4yv8,4yv9,4yvc,4
yve,4yvz,4yw2,4yw6,4yw7,4ywa,4yx4,4yx9,4yxd,4yxi,4yxo,4yxu,4yy6,4yyi,4yyl,
4yym,4yyn,4yyt,4yz5,4yz9,4yzc,4yzm,4yzn,4yzu,4z07,4z0d,4z0e,4z0f,4z0k,4z0q
,4z0u,4z16,4z1e,4z1j,4z1k,4z1n,4z1q,4z1s,4z22,4z2b,4z2g,4z2h,4z2i,4z2j,4z2
k,4z2l,4z2o,4z2p,4z3v,4z46,4z5w,4z68,4z6h,4z6i,4z7f,4z7h,4z7i,4z7n,4z7o,4z
7q,4z83,4z84,4z88,4z89,4z8a,4z8d,4z8m,4z90,4z93,4z9l,4za0,4zae,4zam,4zb6,4
zb8,4zba,4zbf,4zbi,4zcs,4zcw,4zdu,4ze6,4zeb,4zec,4zed,4zei,4zek,4zfi,4zg6,
4zg7,4zg9,4zga,4zgk,4zh2,4zh3,4zh4,4zhl,4zhm,4zij,4zim,4zip,4zj8,4zjc,4zji
,4zjj,4zjr,4zjw,4zk5,4zki,4zl4,4zla,4zlo,4zls,4zly,4zlz,4zme,4zmf,4znx,4zo
5,4zom,4zow,4zpe,4zpf,4zpg,4zph,4zqt,4zro,4zs0,4zs2,4zs3,4zs9,4zsa,4zsg,4z
sh,4zsj,4zsl,4zsm,4zsp,4zsq,4zsr,4zt2,4zt3,4zt4,4zt5,4zt6,4zt7,4zt8,4ztl,4
ztm,4ztn,4ztq,4ztr,4zts,4zud,4zum,4zun,4zuo,4zup,4zuq,4zur,4zv1,4zv2,4zvi,
4zw3,4zw5,4zw6,4zw7,4zw8,4zwx,4zwy,4zwz,4zx0,4zx1,4zx3,4zx4,4zx5,4zx6,4zx8
,4zx9,4zxx,4zxy,4zy0,4zy1,4zy2,4zy4,4zy5,4zy6,4zyc,4zyf,4zyi,4zyq,4zyr,4zy
t,4zyu,4zyv,4zyw,4zyx,4zyy,4zyz,4zz0,4zz1,4zz2,4zz3,4zzd,4zzx,4zzy,4zzz,5a
00,5a09,5a0a,5a0b,5a0c,5a0e,5a14,5a1f,5a2i,5a2j,5a2k,5a2s,5a3h,5a3n,5a3o,5
a3q,5a3r,5a3s,5a3t,5a3u,5a3w,5a3x,5a46,5a4c,5a4e,5a4l,5a4q,5a4t,5a54,5a5d,
5a5o,5a5p,5a5q,5a5r,5a5s,5a5v,5a5z,5a69,5a6a,5a6b,5a6h,5a6i,5a6k,5a6n,5a6x
,5a7b,5a7c,5a7i,5a7j,5a7y,5a81,5a82,5a83,5a85,5a8x,5a8y,5a8z,5a9u,5aa8,5aa
9,5aaa,5aab,5aac,5aad,5aae,5aaf,5aag,5aan,5ab0,5ab1,5ab9,5aba,5abe,5abf,5a
bg,5abh,5abp,5abw,5acb,5acw,5acx,5acy,5ad1,5adq,5adr,5ads,5adt,5aeh,5aei,5
ael,5aen,5aep,5aer,5aes,5afj,5afk,5afl,5afm,5afn,5afv,5afx,5agi,5agj,5agr,
5ags,5agt,5agu,5agv,5ah2,5ahj,5ahu,5ahw,5ai0,5ai1,5ai4,5ai5,5ai6,5ai8,5ai9
,5aia,5aib,5aic,5aip,5ais,5aiv,5aix,5ajc,5ajo,5ajp,5ajv,5ajw,5ajx,5ajy,5aj
z,5ak0,5ak2,5ak3,5ak4,5ak5,5ak6,5ake,5akg,5akh,5aki,5akj,5akk,5akl,5aku,5a
kw,5akz,5al1,5al2,5al3,5al4,5al5,5alb,5alc,5ald,5ale,5alf,5alg,5alh,5ali,5
alj,5alk,5all,5alm,5aln,5alo,5alp,5alr,5als,5alt,5alu,5alv,5alw,5alx,5aly,
5alz,5am0,5am1,5am2,5am3,5am4,5am5,5am6,5am7,5amd,5amg,5aml,5amn,5anq,5ans
,5ant,5anu,5anv,5anw,5aoi,5aoj,5aok,5aol,5aom,5ap0,5ap1,5ap2,5ap3,5ap4,5ap
5,5ap6,5ap7,5aph,5apj,5apr,5aqf,5aqg,5aqh,5aqj,5aqk,5aqn,5aqo,5aqp,5aqq,5a
qr,5aqt,5aqu,5aqv,5aqz,5ar0,5ar4,5ar5,5ar7,5ar8,5arf,5arg,5aut,5auu,5auv,5
auw,5aux,5auy,5auz,5av0,5ave,5avf,5avi,5awj,5awt,5awu,5ax3,5ax9,5axi,5axp,
5axq,5ayf,5ayt,5ayy,5azf,5azg,5b0x,5b1s,5b25,5b2d,5b4k,5b4l,5b4w,5b56,5b5b
,5b5f,5b5g,5b5o,5b5p,5b6c,5b6g,5bjt,5bml,5bmm,5bms,5bnj,5bnm,5bnr,5bns,5bo
j,5bot,5boy,5bpa,5bpe,5bpp,5bq0,5bqg,5bqh,5bqi,5bqs,5brn,5bry,5brz,5bs0,5b
s4,5bsk,5btr,5btv,5btx,5bue,5bui,5buj,5bv3,5bvd,5bve,5bvf,5bvk,5bvn,5bvo,5
bvw,5bw4,5bwb,5bwc,5byi,5byy,5byz,5c0k,5c0m,5c11,5c13,5c1m,5c1w,5c1x,5c1y,
5c20,5c26,5c28,5c29,5c2a,5c2e,5c2h,5c2o,5c3k,5c3p,5c42,5c4k,5c4l,5c4o,5c4s
,5c4t,5c4u,5c5h,5c5t,5c6o,5c6p,5c6v,5c7a,5c7b,5c7c,5c7d,5c7e,5c7f,5c7n,5c8
3,5c84,5c85,5c87,5c8k,5c8m,5c8n,5c91,5cal,5can,5cao,5cap,5caq,5cas,5cau,5c
av,5cbm,5cbr,5cbs,5cc2,5ccl,5ccm,5cdh,5ceh,5cei,5ceo,5cep,5ceq,5cf4,5cf5,5
cf6,5cf8,5cfa,5cfb,5cgc,5cgd,5cgj,5cgv,5chk,5ci7,5cil,5cin,5cj6,5cjf,5ckr,
5cks,5clm,5cls,5cnj,5cnm,5cp5,5cp9,5cpr,5cqj,5cqt,5cqu,5cqx,5cr7,5cs2,5cs3
,5cs5,5cs6,5csd,5csh,5cso,5csp,5cst,5csw,5csx,5csz,5ct1,5ct2,5ct7,5ctb,5ct
c,5cte,5cu2,5cu4,5cuh,5cuq,5cuu,5cvd,5cw8,5cwa,5cxa,5cxh,5cxi,5cxz,5cy3,5c
y9,5cyi,5cyv,5czb,5czm,5d0c,5d0j,5d0r,5d10,5d11,5d12,5d1j,5d1n,5d1r,5d1s,5
d1t,5d1u,5d1w,5d21,5d24,5d25,5d26,5d29,5d2a,5d2r,5d3c,5d3h,5d3j,5d3l,5d3n,
5d3p,5d3s,5d3t,5d3x,5d45,5d47,5d48,5d4a,5d6e,5d6f,5d6j,5d6p,5d6q,5d6y,5d75

,5d7a,5d7c,5d7e,5d7r,5d7x,5d9k,5d9l,5d9p,5da3,5dah,5db0,5db1,5db2,5db3,5db
m,5dcz,5dd0,5dd9,5dda,5ddb,5ddc,5ddd,5dde,5ddf,5dex,5dey,5dfp,5dg6,5dgj,5d
gm,5dgu,5dgw,5dgz,5dh3,5dh4,5dh5,5dhf,5dhg,5dhh,5dhj,5dhp,5dhq,5dhr,5dhs,5
dht,5dhu,5di1,5dia,5dif,5diq,5dit,5diu,5div,5dj5,5djp,5djr,5djv,5dk4,5dkn,
5dkr,5dl1,5dls,5dlv,5dlx,5dlz,5dms,5dnu,5doh,5dp4,5dp5,5dp6,5dp7,5dp8,5dp9
,5dpa,5dpw,5dpx,5dq8,5dqc,5dqe,5dqf,5drc,5dri,5dro,5drq,5drr,5drs,5drt,5dr
y,5dsx,5dt2,5dtj,5dtk,5dtm,5dtq,5dtr,5dts,5dtt,5dtw,5du4,5du6,5du8,5duc,5d
uf,5dus,5duw,5dv2,5dv4,5dva,5dw1,5dw2,5dwr,5dx3,5dx4,5dxb,5dxe,5dxg,5dxh,5
dxt,5dxu,5dy5,5dy7,5dya,5dyo,5dyt,5dyw,5dyy,5e0a,5e0g,5e0h,5e0j,5e0l,5e0m,
5e13,5e1b,5e1d,5e1e,5e1m,5e1o,5e1s,5e28,5e2k,5e2l,5e2m,5e2n,5e2o,5e2p,5e2q
,5e2r,5e2s,5e2v,5e2w,5e3a,5e3d,5e3g,5e4w,5e5g,5e6o,5e73,5e74,5e7n,5e7r,5e8
0,5e88,5e89,5e8a,5e8f,5e8r,5e8w,5e8z,5e90,5e91,5ea3,5ea4,5ea5,5ea6,5ea7,5e
ak,5eay,5eb2,5eb3,5ece,5ech,5eci,5ect,5ecv,5edb,5edc,5edd,5edi,5edl,5edq,5
edr,5eds,5edu,5ee7,5ee8,5eec,5eef,5eei,5eek,5eel,5een,5eeq,5ef7,5ef8,5efa,
5efb,5efc,5efh,5efj,5eg4,5ega,5egm,5egs,5egu,5eh0,5eh5,5eh7,5eh8,5ehe,5ehg
,5ehi,5ehn,5ehp,5ehq,5ehr,5ehv,5ehw,5ehy,5ei2,5ei3,5ei4,5ei6,5ei8,5eie,5ei
f,5eij,5eis,5eiw,5eje,5ejl,5ejv,5ejw,5ek0,5ek9,5ekg,5ekh,5ekj,5ekm,5ekn,5e
ko,5ekx,5el2,5el9,5eld,5ele,5elf,5elq,5elv,5elw,5ely,5elz,5em3,5em5,5em6,5
em7,5em8,5em9,5ema,5emb,5emj,5emk,5eml,5emm,5en3,5enb,5enc,5ene,5enf,5eng,
5enh,5eni,5enj,5enk,5enm,5eob,5eoc,5eok,5eol,5eom,5eou,5ep7,5epk,5epl,5epn
,5epp,5epr,5eps,5epy,5eq0,5eq1,5eqe,5eqp,5eqq,5eqy,5er1,5er2,5er4,5er5,5er
g,5es1,5esq,5est,5eta,5etb,5etf,5eth,5etj,5etk,5etl,5etm,5etn,5eto,5etp,5e
tq,5etr,5ets,5ett,5etu,5etv,5etx,5ety,5eu1,5eud,5eue,5euk,5ev8,5eva,5evb,5
evd,5evk,5evz,5ew0,5ew3,5ew9,5ewa,5ewd,5ewh,5ewj,5ewk,5ewm,5ewy,5ewz,5exl,
5exm,5exn,5exw,5exx,5ey0,5ey4,5ey8,5ey9,5eyc,5eyd,5eyk,5eym,5eyr,5eyz,5ez0
,5ezg,5ezh,5ezx,5ezz,5f00,5f01,5f02,5f04,5f08,5f0c,5f0f,5f0h,5f1c,5f1h,5f1
j,5f1l,5f1r,5f1u,5f1v,5f1x,5f1z,5f20,5f25,5f27,5f29,5f2f,5f2k,5f2p,5f2r,5f
2s,5f2u,5f2w,5f32,5f37,5f39,5f3c,5f3e,5f3g,5f3i,5f3t,5f3z,5f41,5f4l,5f4n,5
f4p,5f4r,5f4u,5f5b,5f5i,5f5z,5f60,5f61,5f62,5f63,5f67,5f6d,5f6u,5f6v,5f74,
5f88,5f8y,5f90,5f91,5f94,5f95,5f9b,5f9e,5fa7,5fah,5fao,5fap,5faq,5fas,5fat
,5fb0,5fb1,5fb7,5fbe,5fbi,5fbn,5fbo,5fck,5fcw,5fcz,5fd2,5fdc,5fdd,5fdg,5fd
i,5fdo,5fdp,5fdr,5fdz,5fe6,5fe7,5fe9,5ff6,5fgk,5fh6,5fh7,5fh8,5fhm,5fhn,5f
ho,5fi2,5fi6,5fi7,5fiv,5fjw,5fjx,5fkj,5fky,5fl0,5fl1,5fl4,5fl5,5fl6,5flo,5
flp,5flq,5fls,5flt,5fnc,5fnd,5fnf,5fng,5fni,5fnj,5fnq,5fnr,5fns,5fnt,5fnu,
5fog,5fol,5fom,5foo,5fos,5fot,5fou,5fov,5fow,5fox,5fp0,5fpi,5fpk,5fpp,5fpu
,5fq9,5fqb,5fqc,5fqp,5fqr,5fqs,5fqt,5fqv,5fs5,5fsb,5fsc,5fsl,5fsm,5fsn,5fs
o,5fsx,5fsy,5ftg,5fto,5ftq,5fue,5ful,5fum,5fun,5fus,5fut,5fv7,5fwa,5fwg,5f
wj,5fwr,5fxq,5fxr,5fxs,5fyq,5fyx,5g0q,5g10,5g11,5g17,5g1a,5g1c,5g1n,5g1p,5
g1z,5g22,5g2b,5g2g,5g2n,5g3m,5g3n,5g3w,5g42,5g43,5g45,5g46,5g4m,5g4n,5g4o,
5g53,5g57,5g5f,5g5v,5g5z,5g60,5g61,5g6u,5gg4,5ggj,5ggk,5ggl,5ggn,5ggo,5ggp
,5ggz,5ghv,5gic,5gid,5gj9,5gja,5gjd,5gjf,5gjg,5glu,5gmh,5gmi,5gmj,5gmm,5gm
n,5gmp,5gmu,5gmv,5gn5,5gn6,5gn7,5gn9,5gnk,5go2,5goe,5gof,5gow,5gp7,5gr9,5g
s4,5gs9,5gsa,5gso,5gsw,5gtr,5gty,5gu4,5gub,5gut,5gv2,5gvk,5gvl,5gvm,5gvn,5
gvp,5gwa,5gwy,5gwz,5gx6,5gx7,5h08,5h09,5h0b,5h0e,5h0g,5h0h,5h13,5h14,5h15,
5h17,5h19,5h1e,5h1t,5h1u,5h1v,5h21,5h22,5h2u,5h3q,5h4j,5h5f,5h5o,5h5q,5h5r
,5h5s,5h63,5h6v,5h7g,5h7h,5h85,5h8b,5h8e,5h8g,5h8x,5h9p,5h9q,5h9r,5h9s,5ha
1,5ha9,5hbe,5hbh,5hbj,5hbn,5hbs,5hcl,5hct,5hcv,5hcx,5hcy,5hd0,5hda,5hdu,5h
dv,5hdx,5hdz,5he0,5he1,5he2,5he3,5he4,5he5,5he7,5heb,5hed,5hes,5hex,5hey,5
hf1,5hfb,5hfc,5hff,5hfj,5hfu,5hg1,5hg5,5hg7,5hg8,5hg9,5hgc,5hgq,5hh4,5hh5,
5hh6,5hhx,5hi7,5hip,5his,5hj9,5hja,5hjb,5hjc,5hjd,5hjq,5hk1,5hk2,5hk9,5hka
,5hkb,5hkh,5hki,5hkm,5hl9,5hlb,5hld,5hlm,5hln,5hlp,5hls,5hlw,5hm0,5hm3,5hm
h,5hmi,5hmk,5hmy,5hn0,5hn7,5hn8,5hn9,5hna,5hng,5ho6,5ho7,5ho8,5hoa,5hog,5h
or,5hpm,5hrv,5hrw,5hrx,5htb,5htc,5htl,5htz,5hu0,5hu1,5hu9,5huw,5huy,5hv0,5
hv1,5hva,5hvp,5hvs,5hvt,5hvu,5hvy,5hwu,5hwv,5hx6,5hx8,5hyq,5hyr,5hyx,5hz5,

5hz6,5hz8,5hz9,5hzn,5hzx,5i0b,5i0l,5i12,5i13,5i1q,5i22,5i23,5i24,5i25,5i29
,5i2e,5i2f,5i2i,5i2r,5i2z,5i38,5i3a,5i3m,5i3v,5i3w,5i3x,5i3y,5i40,5i43,5i4
o,5i4v,5i56,5i58,5i59,5i5x,5i5z,5i7u,5i7x,5i7y,5i80,5i83,5i86,5i88,5i89,5i
8b,5i8c,5i8g,5i8p,5i94,5i9i,5i9x,5i9y,5i9z,5ia0,5ia1,5ia2,5ia3,5ia4,5ia5,5
iaw,5iay,5ick,5icp,5icv,5icx,5icy,5icz,5id0,5id1,5idn,5idp,5ie1,5iee,5ief,
5ieg,5ien,5ieo,5iep,5ier,5iez,5if4,5if6,5ifu,5igk,5igl,5igm,5igq,5ih2,5ih5
,5ih6,5ih8,5ih9,5iha,5ihc,5ihh,5ii1,5ii2,5iis,5ij7,5ijj,5ijp,5ijr,5ikb,5il
1,5im3,5ime,5in9,5inh,5iok,5iop,5ioy,5ioz,5ipa,5ipc,5ipj,5iq6,5iql,5ir1,5i
rr,5isl,5ism,5isz,5itd,5itf,5ito,5itp,5iu2,5iu4,5iu6,5iu7,5iu8,5iua,5iub,5
iug,5iuh,5iui,5iv2,5iv4,5ivc,5ive,5ivf,5ivj,5ivn,5ivs,5ivt,5ivv,5ivy,5ivz,
5iw0,5iwg,5ix0,5ix1,5ixf,5ixq,5ixt,5iy4,5iyv,5iyy,5iz6,5iz8,5iz9,5iza,5izc
,5izf,5izj,5izk,5izl,5izm,5izq,5izu,5j0d,5j18,5j19,5j1r,5j1v,5j1w,5j1x,5j2
0,5j27,5j2x,5j31,5j32,5j3l,5j3s,5j3v,5j41,5j47,5j4n,5j4v,5j4y,5j58,5j59,5j
5d,5j5r,5j5t,5j5x,5j64,5j6a,5j6d,5j6l,5j6m,5j6n,5j6s,5j71,5j74,5j75,5j79,5
j7b,5j7f,5j7g,5j7j,5j7p,5j7q,5j7s,5j7w,5j82,5j86,5j87,5j8i,5j8m,5j8u,5j8x,
5j8z,5j9f,5j9k,5j9l,5j9x,5j9y,5j9z,5ja0,5jah,5jal,5jan,5jao,5jap,5jar,5jas
,5jat,5jau,5jaz,5jbi,5jc1,5jcb,5jcj,5jdc,5jdi,5jek,5jeo,5jer,5jf1,5jf2,5jf
3,5jf4,5jf5,5jf6,5jf7,5jf8,5jfd,5jfp,5jfr,5jfu,5jg1,5jga,5jgb,5jgd,5jgi,5j
gq,5jh6,5jha,5jhb,5jhd,5jhk,5ji6,5ji8,5jic,5jid,5jim,5jin,5jiq,5jiy,5jjm,5
jjr,5jjs,5jk3,5jlz,5jm4,5jmp,5jmw,5jmx,5jn8,5jn9,5jna,5jnc,5jnl,5jnn,5jo0,
5jog,5joh,5jop,5jox,5jpt,5jq5,5jq7,5jq8,5jq9,5jqb,5jr2,5jr6,5jrq,5jrs,5js3
,5jsg,5jsj,5jsm,5jsq,5jss,5jt2,5jt9,5jur,5juz,5jv0,5jv1,5jv2,5jvi,5jxn,5jx
q,5jy0,5jy3,5jyo,5jyp,5jyy,5jz9,5jzb,5jzi,5jzn,5jzs,5jzy,5k00,5k03,5k05,5k
09,5k0b,5k0c,5k0f,5k0h,5k0i,5k0j,5k0m,5k0s,5k0t,5k0x,5k13,5k1d,5k1f,5k1i,5
k1v,5k32,5k48,5k4i,5k4j,5k4l,5k4x,5k4z,5k51,5k5c,5k5e,5k5n,5k5s,5k6a,5k6s,
5k76,5k7h,5k8n,5k8o,5k8s,5k8v,5k9w,5ka1,5ka3,5ka7,5ka9,5kab,5kad,5kam,5kap
,5kat,5kau,5kax,5kbe,5kbf,5kbg,5kbh,5kbi,5kbq,5kbr,5kby,5kcb,5kcv,5kcx,5kd
e,5kdf,5kdr,5ke0,5kej,5kew,5kez,5kgn,5kgw,5kgx,5kh3,5kh7,5khd,5khg,5khh,5k
hi,5khj,5khk,5khm,5khx,5kit,5kj0,5kj2,5kjk,5kjm,5kjn,5kk8,5kkr,5kks,5kkt,5
klp,5klr,5kls,5klt,5kly,5klz,5km0,5km1,5km2,5km3,5km5,5km9,5kma,5kmf,5kmh,
5knj,5knr,5kns,5knt,5knu,5knv,5knx,5ko1,5ko5,5koq,5kos,5kpk,5kpl,5kpm,5kq5
,5kqd,5kqf,5kqg,5kql,5kqx,5kqy,5kr0,5kr1,5kr2,5kr8,5kre,5ks7,5ksu,5ksv,5ks
x,5ktu,5ktw,5ktx,5ku3,5ku6,5ku9,5kup,5kv8,5kv9,5kva,5kw2,5kww,5kx7,5kx8,5k
xc,5kxi,5kya,5kyj,5kyk,5kz0,5kzi,5kzp,5kzq,5l01,5l0c,5l0h,5l13,5l15,5l17,5
l26,5l2i,5l2m,5l2n,5l2o,5l2s,5l2t,5l2w,5l2y,5l2z,5l30,5l3a,5l3e,5l3f,5l3g,
5l3j,5l44,5l4e,5l4f,5l4h,5l4i,5l4j,5l4m,5l6h,5l6i,5l6j,5l6o,5l6p,5l72,5l7e
,5l7f,5l7g,5l7h,5l7k,5l87,5l8a,5l8c,5l8n,5l8o,5l8t,5l8y,5l96,5l97,5l98,5l9
9,5l9g,5l9h,5l9i,5l9l,5l9o,5laq,5lav,5law,5lax,5lay,5laz,5lb7,5lbq,5lc0,5l
ca,5lce,5lcf,5lch,5lcj,5lck,5ld8,5ldk,5ldm,5ldo,5ldp,5le1,5lg3,5lgn,5lgo,5
lgp,5lgq,5lgr,5lgs,5lgt,5lgu,5lh4,5lh8,5lhg,5lhh,5lhi,5lhu,5li3,5lif,5lj0,
5lj1,5lj2,5ljj,5ljq,5ljt,5lkr,5ll4,5ll5,5ll7,5ll9,5lla,5llc,5lle,5llg,5llh
,5lli,5llm,5llo,5llp,5lm4,5lm6,5lma,5lmb,5lmk,5lne,5lny,5lo5,5lo6,5loh,5lo
m,5lp1,5lp6,5lpd,5lpj,5lpk,5lpl,5lpm,5lpr,5lqf,5lqq,5lrg,5lrj,5lrk,5lrq,5l
s6,5lsc,5lsg,5lsh,5lso,5lss,5lsx,5lsy,5lt6,5lt9,5ltn,5lto,5lu2,5lub,5lud,5
luu,5lvd,5lvf,5lvl,5lvn,5lvq,5lvr,5lvx,5lwd,5lwe,5lwm,5lwn,5lws,5lx6,5lxb,
5lxc,5lxd,5lxp,5ly1,5ly2,5ly3,5lyh,5lyn,5lyr,5lyw,5lyx,5lyy,5lz2,5lz4,5lz5
,5lz7,5lz8,5lz9,5lzg,5lzh,5lzj,5m04,5m0d,5m0m,5m0s,5m17,5m1z,5m23,5m25,5m2
8,5m29,5m2q,5m2v,5m34,5m39,5m3a,5m3b,5m44,5m4c,5m4f,5m4i,5m4k,5m4q,5m4u,5m
51,5m53,5m55,5m56,5m57,5m5d,5m5q,5m63,5m6f,5m6h,5m6m,5m6u,5m77,5m7m,5m7s,5
m7t,5m7u,5m9d,5m9w,5ma7,5mae,5maj,5mar,5mat,5mav,5may,5mb1,5mby,5meh,5mek,
5mes,5mev,5mf6,5mfr,5mfs,5mft,5mg2,5mge,5mgf,5mgg,5mgi,5mgj,5mgk,5mgl,5mgm
,5mgx,5mhp,5mhq,5mi3,5mi5,5mi6,5mi7,5mi9,5mih,5mim,5mja,5mjb,5mjn,5mk1,5mk
3,5mk9,5mka,5mkj,5mkr,5mks,5mkx,5mky,5mkz,5ml0,5ml2,5ml3,5ml4,5ml5,5ml6,5m
l8,5mli,5mlj,5mlo,5mlw,5mm9,5mme,5mmg,5mmn,5mmp,5mn1,5mnb,5mnc,5mng,5mnh,5

mnn,5mno,5mnr,5mnx,5mny,5mo0,5mo2,5mo8,5mob,5moc,5mod,5moe,5mon,5moo,5moq,
5mos,5mpk,5mpn,5mpz,5mqe,5mql,5mqt,5mqv,5mqx,5mqy,5mra,5mrb,5mrd,5mrh,5mri
,5mrm,5mro,5mrp,5msb,5mt0,5mt4,5mte,5mts,5mtv,5mtw,5mtx,5mty,5muc,5mw2,5mw
3,5mw4,5mw6,5mwa,5mwd,5mwg,5mwh,5mwj,5mwo,5mwp,5mwy,5mwz,5mxf,5mxk,5mxo,5m
xq,5mxr,5mxv,5mxx,5my8,5myd,5myg,5myk,5myl,5mym,5myn,5myo,5myr,5mys,5myv,5
myx,5mz3,5mz8,5mzg,5n0d,5n0e,5n0f,5n16,5n17,5n18,5n1p,5n1r,5n1s,5n1v,5n1x,
5n1y,5n1z,5n20,5n21,5n24,5n25,5n2d,5n2f,5n2t,5n2x,5n2z,5n31,5n34,5n3v,5n3w
,5n3y,5n49,5n4s,5n4t,5n53,5n55,5n58,5n69,5n6s,5n70,5n7b,5n7g,5n7v,5n7x,5n8
4,5n87,5n8b,5n8e,5n8j,5n8t,5n8v,5n8w,5n93,5n99,5n9k,5n9l,5n9n,5n9r,5n9s,5n
9t,5na0,5nad,5nai,5nap,5nar,5nat,5nau,5naw,5nb6,5nb7,5nba,5nbw,5ncq,5ncy,5
ncz,5ndb,5ndd,5ndf,5ne1,5ne3,5ne5,5nea,5neb,5nee,5nev,5nf5,5nf6,5nf9,5nfa,
5nfb,5nfh,5ng9,5ngb,5nge,5ngf,5ngr,5ngs,5ngt,5ngu,5ngz,5nhf,5nhh,5nhj,5nhl
,5nho,5nhp,5nhv,5nhy,5nhz,5ni0,5ni5,5ni7,5ni8,5nib,5nih,5nin,5niy,5njx,5nj
z,5nk2,5nk3,5nk4,5nk6,5nk7,5nk8,5nk9,5nka,5nkb,5nkc,5nkd,5nkg,5nkh,5nki,5n
kk,5nkn,5nlk,5nme,5nmf,5nmg,5nn0,5nn4,5nn5,5nn6,5nne,5nob,5np8,5npb,5npc,5
npd,5npe,5npf,5npr,5nps,5nq5,5nqe,5nqr,5nr7,5nr8,5nra,5nrf,5nsp,5nsx,5nt0,
5nt4,5ntk,5ntp,5ntq,5ntt,5ntw,5nu1,5nu3,5nu5,5nud,5nut,5nuu,5nvc,5nve,5nvf
,5nvh,5nvv,5nvw,5nvx,5nvy,5nvz,5nw0,5nw1,5nw2,5nw7,5nw8,5nwb,5nwc,5nwd,5nw
e,5nwg,5nwh,5nwi,5nwk,5nwz,5nx2,5nx9,5nxg,5nxi,5nxo,5nxp,5nxq,5nxv,5nxw,5n
xx,5nxy,5ny1,5ny3,5ny6,5nya,5nyh,5nyz,5nz2,5nz4,5nze,5nzf,5nzm,5nzn,5nzo,5
nzp,5nzq,5o07,5o0a,5o0b,5o0e,5o0j,5o0s,5o11,5o1a,5o1b,5o1c,5o1d,5o1e,5o1f,
5o1g,5o1h,5o1i,5o1s,5o22,5o2d,5o3q,5o3r,5o45,5o48,5o4f,5o4s,5o4t,5o4y,5o4z
,5o55,5o58,5o5a,5o5f,5o5h,5o5m,5o7e,5o7i,5o7n,5o83,5o87,5o8t,5o91,5o9h,5o9
o,5o9p,5o9q,5o9r,5o9y,5oa2,5oa6,5oae,5oah,5oai,5oaj,5oax,5obg,5obj,5obr,5o
ci,5ocj,5od1,5od5,5ods,5odu,5odx,5ody,5oei,5of0,5ofi,5ofu,5ofv,5ofw,5ofx,5
ogb,5ogk,5ogl,5oh1,5oh2,5oh3,5oh4,5oh7,5oh9,5oha,5ohi,5ohj,5oht,5ohy,5oje,
5ok3,5ok6,5okt,5oku,5ol3,5olb,5olk,5om2,5om3,5om7,5om9,5one,5oot,5op2,5op4
,5op5,5op6,5op8,5opb,5opc,5opr,5ops,5opu,5opv,5oq4,5oq5,5oq6,5oq7,5oq8,5oq
u,5oqw,5or9,5orb,5org,5orh,5orj,5ork,5orl,5orr,5ort,5orv,5orw,5orx,5ory,5o
rz,5os0,5os1,5os2,5os3,5os4,5os5,5os7,5os8,5osd,5ose,5osk,5osl,5oss,5ost,5
osy,5ot3,5ot8,5ot9,5ota,5otc,5otr,5otz,5ou1,5ou2,5ou3,5oua,5oug,5ouh,5oui,
5oul,5ous,5ov8,5ov9,5ovc,5ove,5ovf,5ovg,5ovh,5ovi,5ovp,5ovr,5ovv,5ovx,5ow1
,5ow8,5owa,5owc,5owf,5owh,5owl,5ows,5owt,5ox5,5ox6,5oxg,5oxk,5oxl,5oxm,5ox
n,5oy3,5oyd,5prc,5pzm,5pzn,5q0d,5q0e,5q0f,5q0g,5q0h,5q0i,5q0j,5q0l,5q0m,5q
0n,5q0o,5q0p,5q0q,5q0r,5q0s,5q0t,5q0u,5q0v,5q0w,5q0x,5q0y,5q0z,5q10,5q11,5
q12,5q13,5q14,5q15,5q16,5q17,5q18,5q19,5q1a,5q1b,5q1c,5q1d,5q1e,5q1f,5q1g,
5q1h,5q1i,5qa4,5qa5,5qa6,5qa7,5qa8,5qa9,5qaa,5qab,5qac,5qad,5qae,5qaf,5qag
,5qah,5qai,5qaj,5qak,5qal,5qam,5qan,5qao,5qap,5qaq,5qar,5qas,5qat,5qau,5qa
v,5qaw,5qax,5qay,5qaz,5qb0,5qb1,5qb2,5qb3,5qbv,5qby,5qc4,5qck,5qcl,5qcm,5q
cn,5qik,5qil,5qim,5qin,5qj2,5qj3,5qqo,5qqp,5qtt,5qtu,5std,5sve,5svi,5svk,5
svl,5svx,5svy,5svz,5swf,5swg,5swh,5sxk,5sxm,5sxn,5sy2,5sy3,5sym,5syn,5sys,
5sz0,5sz1,5sz2,5sz3,5sz4,5sz5,5sz6,5sz7,5sz9,5szb,5szc,5t18,5t19,5t1a,5t1i
,5t1k,5t1l,5t1m,5t1s,5t1t,5t1u,5t1w,5t23,5t27,5t28,5t2b,5t2d,5t2g,5t2i,5t2
l,5t2m,5t2p,5t2t,5t2y,5t31,5t35,5t36,5t37,5t3n,5t4b,5t4e,5t4f,5t4h,5t4u,5t
4v,5t52,5t54,5t5g,5t66,5t68,5t6f,5t6g,5t6j,5t6p,5t6z,5t70,5t78,5t7s,5t8e,5
t8j,5t8o,5t8p,5t8q,5t8r,5t90,5t92,5t97,5t9u,5t9w,5t9z,5ta2,5ta4,5ta6,5tb6,
5tbe,5tbj,5tbm,5tbn,5tbp,5tc0,5tca,5tcc,5tci,5tcj,5tco,5tcy,5td2,5td7,5tdb
,5tdi,5tdr,5tdw,5tef,5teg,5teh,5tex,5tfx,5tg1,5tg2,5tg4,5tg5,5tg6,5tg7,5tg
c,5tgy,5th2,5th4,5th7,5tha,5thi,5thj,5thn,5tho,5ti0,5ti2,5ti3,5ti4,5ti5,5t
i6,5ti7,5tig,5tiu,5tjx,5tkb,5tkd,5tkj,5tkk,5tks,5tkt,5tku,5tl9,5tln,5tmn,5
tmp,5tnt,5to8,5tob,5toe,5tol,5toz,5tp0,5tpb,5tpc,5tpg,5tpx,5tq1,5tq3,5tq4,
5tq5,5tq6,5tq7,5tq8,5tqe,5tqf,5tqg,5tqs,5tqu,5tr6,5trf,5trg,5trh,5tri,5trj
,5trk,5trr,5trs,5try,5ts0,5tt3,5tt7,5tt8,5ttf,5ttg,5tts,5ttu,5ttv,5ttw,5tu
o,5tuq,5tur,5tus,5tuy,5tuz,5tv1,5tv3,5tvn,5tw2,5tw3,5tw5,5twg,5twh,5twj,5t

wl,5two,5twx,5twz,5tx3,5tx5,5txy,5ty1,5ty8,5ty9,5tya,5tyh,5tyi,5tyj,5tyk,5
tyl,5tyn,5tyo,5typ,5tyr,5tys,5tz3,5tza,5tzc,5tzd,5tzf,5tzg,5tzh,5tzo,5tzw,
5tzx,5tzy,5tzz,5u00,5u06,5u0d,5u0e,5u0f,5u0g,5u0w,5u0y,5u0z,5u11,5u12,5u13
,5u14,5u1q,5u28,5u2c,5u2e,5u2f,5u2j,5u3b,5u48,5u49,5u4a,5u4b,5u4c,5u4d,5u4
e,5u4f,5u4g,5u4x,5u51,5u5h,5u5k,5u5l,5u5t,5u62,5u66,5u69,5u6b,5u6c,5u6d,5u
6j,5u6k,5u6v,5u7d,5u7i,5u7j,5u7k,5u7l,5u7m,5u7o,5u8a,5u8c,5u8f,5u94,5u98,5
u9d,5u9i,5uac,5uah,5ual,5ubg,5ubt,5uc1,5uc4,5uch,5uci,5ucj,5ue4,5ueu,5uex,
5uey,5uez,5uf0,5ufc,5uff,5ufi,5ufo,5ufp,5ufr,5ufs,5ug8,5ug9,5uga,5ugb,5ugc
,5ugd,5ugg,5ugh,5ugm,5uhi,5uig,5uiq,5uir,5uis,5uit,5uiu,5ujo,5ujv,5uk8,5uk
j,5ukk,5ukl,5ukm,5ul1,5ul5,5ul6,5ula,5ulg,5uln,5ulp,5ult,5umw,5umx,5umy,5u
mz,5un1,5un9,5unf,5ung,5unh,5unj,5unp,5uoo,5uor,5uov,5uox,5uoy,5up0,5up3,5
upe,5upf,5upj,5upz,5uq9,5uqv,5uqx,5ur1,5ur5,5ur6,5ur9,5ura,5urj,5urk,5urm,
5usf,5usq,5usy,5usz,5ut0,5ut4,5ut6,5uuu,5uv1,5uv2,5uv5,5uvc,5uw5,5uwf,5uwi
,5uwj,5uwk,5uwl,5uwm,5uwn,5uwp,5ux4,5uxf,5uxm,5uxn,5uy8,5uyu,5uz0,5uzj,5uz
k,5v0n,5v13,5v19,5v1b,5v1d,5v1y,5v24,5v2l,5v2p,5v2q,5v35,5v37,5v3h,5v3o,5v
3r,5v3x,5v3y,5v40,5v41,5v42,5v49,5v4b,5v4q,5v5d,5v5e,5v5n,5v5o,5v5y,5v6u,5
v6y,5v79,5v7a,5v7i,5v7t,5v7w,5v82,5v83,5v84,5v86,5v88,5v8o,5v8p,5v8q,5v8v,
5v9p,5v9t,5va9,5vad,5var,5vb5,5vb6,5vb7,5vb9,5vc3,5vc4,5vc5,5vc6,5vcv,5vcw
,5vcx,5vcy,5vcz,5vd0,5vd1,5vd2,5vd3,5vdk,5vdo,5vdp,5vdq,5vdr,5vds,5vdt,5vd
u,5vdv,5vdw,5vee,5vew,5vex,5vfc,5vfd,5vfj,5vfm,5vfn,5vgi,5vgo,5vgy,5vh0,5v
i6,5vih,5vii,5vij,5vil,5vio,5vja,5vjn,5vjp,5vk0,5vkc,5vkf,5vkm,5vl2,5vlh,5
vlk,5vll,5vlp,5vlr,5vm0,5vm6,5vnb,5vnd,5vo1,5vo2,5vo6,5voj,5vom,5vp0,5vp1,
5vp9,5vpm,5vqe,5vqi,5vqq,5vqr,5vqs,5vqt,5vqu,5vqv,5vqw,5vqx,5vqy,5vqz,5vr8
,5vrl,5vrp,5vs6,5vsb,5vsc,5vsd,5vse,5vsf,5vsj,5vsk,5vt1,5vt4,5vtb,5vwi,5vw
k,5vyy,5vzu,5vzy,5w0e,5w0f,5w0i,5w0l,5w0q,5w10,5w12,5w13,5w14,5w19,5w1e,5w
1v,5w1w,5w2p,5w2q,5w2s,5w38,5w3i,5w44,5w4e,5w4r,5w4s,5w4v,5w4w,5w5j,5w5k,5
w5o,5w5s,5w5u,5w5v,5w6e,5w6i,5w6o,5w6r,5w6t,5w6u,5w73,5w7i,5w7j,5w7u,5w7x,
5w84,5w85,5w86,5w88,5w8h,5w8i,5w8j,5w8v,5w92,5w94,5w99,5w9g,5wa1,5wa4,5wa5
,5wa6,5wa7,5wa8,5wa9,5wac,5wad,5wae,5waf,5wag,5wal,5wap,5wb3,5wb6,5wbf,5wb
k,5wbl,5wbm,5wbo,5wbp,5wbq,5wbr,5wbz,5wcl,5wcm,5wdc,5wdj,5wdl,5wdw,5we9,5w
eb,5wef,5wei,5wej,5wep,5wev,5wew,5wex,5wf3,5wf5,5wf6,5wf7,5wfc,5wfd,5wfj,5
wfm,5wfw,5wfz,5wg3,5wg4,5wg5,5wg6,5wg7,5wg8,5wg9,5wgd,5wgp,5wgq,5wh5,5wh6,
5whc,5wi0,5wi1,5wic,5wii,5wij,5wik,5win,5wio,5wip,5wir,5wj6,5wjj,5wkf,5wkh
,5wkl,5wkm,5wl0,5wle,5wlg,5wlo,5wlt,5wlv,5wmg,5wmt,5wo4,5wou,5wp5,5wpb,5wq
a,5wqc,5wqd,5wqj,5wqk,5wr7,5wre,5wrs,5ws3,5wtt,5wuk,5wuu,5wvd,5wxf,5wxg,5w
xh,5wxo,5wxp,5wyq,5wyr,5wys,5wyx,5wyz,5wzr,5wzs,5wzt,5wzu,5wzv,5wzw,5x02,5
x13,5x26,5x27,5x28,5x33,5x4m,5x4n,5x4o,5x4p,5x4q,5x54,5x5g,5x5o,5x62,5x72,
5x73,5x74,5x79,5x8i,5x9h,5x9o,5x9p,5xaf,5xag,5xbt,5xff,5xfj,5xg4,5xg5,5xgh
,5xgl,5xhr,5xhs,5xhz,5xig,5xih,5xii,5xij,5xiw,5xjm,5xkm,5xmp,5xmr,5xms,5xm
t,5xmu,5xmv,5xmx,5xn3,5xo2,5xo7,5xof,5xp7,5xpi,5xpl,5xpm,5xpn,5xpo,5xpp,5x
qx,5xs2,5xs8,5xsr,5xst,5xsu,5xup,5xv7,5xva,5xvf,5xvg,5xvk,5xvq,5xvu,5xvw,5
xw6,5xwr,5xxf,5xxk,5xyf,5xyx,5xyy,5xyz,5xzr,5y0f,5y0g,5y0x,5y0z,5y12,5y13,
5y1u,5y1y,5y20,5y21,5y24,5y2f,5y3n,5y3o,5y48,5y53,5y59,5y5n,5y5t,5y5u,5y5w
,5y61,5y62,5y6d,5y6e,5y6f,5y6g,5y6k,5y7h,5y7j,5y7k,5y7w,5y7z,5y80,5y86,5y8
c,5y8w,5y8y,5y8z,5y93,5y94,5y97,5y9l,5ya5,5yas,5yba,5ybe,5ybi,5yc1,5yc2,5y
c3,5yc4,5yc8,5yco,5ye7,5ye8,5ye9,5yea,5yf1,5yfs,5yft,5yfz,5yg2,5yg3,5yg4,5
ygd,5ygf,5ygi,5yh8,5yhe,5yhg,5yhl,5yia,5yib,5yic,5yid,5yie,5yij,5yj8,5yjb,
5yjf,5yji,5yjk,5yjm,5yjo,5yjp,5yjy,5ykp,5yl2,5ylj,5yls,5ylt,5ylu,5ylv,5ymx
,5yof,5yov,5yp5,5yp6,5ypo,5ypp,5ypw,5ypy,5yql,5yqn,5yqo,5yqw,5yqx,5yr4,5yr
5,5yr6,5yso,5yto,5ytu,5yu9,5yum,5yun,5yv5,5yve,5yvt,5yvx,5ywg,5ywx,5ywy,5y
y4,5yy6,5yy9,5yyb,5yyf,5yyz,5yz2,5yz7,5yzc,5yzd,5z1c,5z1d,5z1e,5z1r,5z1s,5
z1t,5z4h,5z4o,5z5f,5z5v,5z66,5z68,5z7b,5z7j,5z89,5z95,5z99,5z9e,5za1,5za2,
5za7,5za8,5za9,5zae,5zaf,5zag,5zah,5zaj,5zbz,5zc5,5zcu,5zdb,5zdc,5zdd,5zde
,5zdf,5zdg,5zdp,5ze6,5zee,5zef,5zeq,5zfi,5zg0,5zg1,5zg3,5zgg,5zh2,5zh3,5zh

4,5zh5,5zhj,5zhl,5zhm,5zhn,5zia,5zjf,5zjy,5zjz,5zk3,5zk5,5zk7,5zk8,5zk9,5zkb,5zkc,5zku,5zla,5zlf,5zma,5zml,5zmq,5zms,5znc,5zni,5znl,5znp,5znr,5zo7,5zo8,5zo9,5zob,5zoo,5zop,5zqo,5zqp,5zqq,5zqr,5zqu,5zr3,5zt1,5ztn,5zty,5zu0,5zuj,5zun,5zvw,5zw6,5zwe,5zwf,5zwh,5zwi,5zxi,5zxk,5zyg,5zyh,5zyi,5zyj,5zyk,5zyl,5zym,5zys,5zz2,5zz4,5zzw,6a03,6a04,6a05,6a06,6a1c,6a30,6a3n,6a5e,6a6k,6a6w,6a73,6a80,6a84,6a87,6a8g,6a8n,6a93,6a94,6a9c,6a9o,6aa4,6aa5,6aad,6aah,6aaj,6aak,6aam,6aan,6aao,6aaq,6abk,6abp,6abx,6acb,6aec,6aee,6aeh,6aej,6af9,6afa,6afc,6afd,6afe,6aff,6afg,6afh,6afi,6afj,6afk,6afl,6afr,6agg,6agk,6agp,6agt,6ahi,6ahs,6ai9,6ajg,6ajh,6aji,6ajj,6ajr,6ajv,6ajx,6ajy,6ajz,6ak4,6ak5,6ak6,6akw,6alc,6alz,6am8,6ami,6an1,6anl,6aol,6aom,6aox,6aoy,6ap6,6ap7,6ap8,6apr,6aps,6apt,6apu,6apv,6apw,6apz,6aqf,6aqo,6aqq,6aqs,6ar2,6ar4,6ar9,6arj,6arm,6arn,6aro,6arv,6ary,6as6,6as8,6asu,6asz,6at0,6atv,6au2,6au5,6au9,6aud,6aum,6avi,6awn,6awo,6awp,6ax1,6ax4,6axb,6axj,6axk,6axl,6axp,6axq,6ay3,6ay5,6ayd,6ayh,6ayi,6ayn,6ayo,6ayq,6ayr,6ays,6ayt,6azk,6azl,6b0v,6b0y,6b16,6b1c,6b1e,6b1f,6b1h,6b1j,6b1k,6b1o,6b1w,6b1x,6b1y,6b22,6b27,6b2c,6b2p,6b2q,6b30,6b31,6b33,6b3e,6b3v,6b41,6b4d,6b4h,6b4l,6b4n,6b4u,6b4w,6b59,6b5a,6b5i,6b5j,6b5m,6b5o,6b5q,6b5r,6b5t,6b67,6b7a,6b7b,6b7c,6b7d,6b7e,6b7f,6b7h,6b8j,6b8u,6b8y,6b95,6b96,6b97,6b98,6ba7,6bau,6baw,6bbs,6bbu,6bbv,6bbx,6bc9,6bcr,6bcy,6bd1,6bdy,6beb,6bec,6bed,6bee,6bef,6beh,6bfa,6bfd,6bfe,6bfn,6bfp,6bfw,6bfx,6bg3,6bg5,6bgg,6bgu,6bgv,6bgw,6bgx,6bgy,6bgz,6bh0,6bh1,6bh2,6bh3,6bh5,6bh8,6bhd,6bhe,6bhh,6bhi,6bhv,6bib,6bic,6bid,6bij,6bik,6bil,6bin,6bir,6biv,6bix,6biy,6biz,6bj2,6bj3,6bjo,6bke,6bkh,6bkw,6bkx,6bky,6bl1,6bl2,6bln,6bm5,6bm6,6bmi,6bmr,6bmv,6bmx,6bnh,6bnk,6bnl,6bnt,6bny,6bo6,6bod,6boe,6boy,6bq0,6bqa,6bqd,6bqg,6bqh,6bqj,6bqk,6br2,6br3,6bs3,6bs4,6bs5,6bsk,6bsl,6bsm,6bsx,6bt0,6bt6,6bto,6bu0,6bu1,6bu3,6buu,6buv,6bvb,6bvh,6bw2,6bw3,6bw4,6bw5,6bw8,6bx6,6bxy,6by8,6byk,6byz,6c0n,6c0r,6c0s,6c0t,6c0u,6c1s,6c28,6c2r,6c2t,6c2x,6c2y,6c3e,6c3l,6c3n,6c3u,6c42,6c4d,6c4g,6c4u,6c5f,6c5h,6c5j,6c5k,6c5q,6c5t,6c67,6c6o,6c7b,6c7d,6c7e,6c7f,6c7g,6c7i,6c7j,6c7q,6c7r,6c7w,6c7x,6c85,6c8c,6c8p,6c8x,6c91,6c98,6c99,6c9n,6c9p,6c9q,6c9r,6c9s,6c9v,6cb5,6cbf,6cbg,6cbh,6cc9,6cck,6ccl,6ccm,6ccn,6cco,6ccq,6ccs,6cct,6ccu,6ccx,6ccy,6cd4,6cd5,6cd8,6cd9,6cdc,6cdg,6cdj,6cdl,6cdm,6cdo,6cdp,6ce2,6ce6,6ce8,6cea,6cec,6ced,6cee,6cef,6ceh,6cen,6cer,6cex,6cf5,6cf6,6cf7,6cfc,6cfd,6cgp,6cgt,6cha,6chh,6chl,6chm,6chn,6cho,6chp,6chq,6cis,6ciy,6cj1,6cj5,6cje,6cjh,6cjj,6cjp,6cjr,6cjs,6cjv,6cjw,6cjy,6ck3,6ck6,6ckc,6cki,6ckl,6ckr,6cks,6ckw,6ckx,6clv,6cmj,6cmr,6cms,6cn5,6cn6,6cn8,6cnj,6cnk,6co4,6coj,6cpa,6cpw,6cq0,6cq1,6cq4,6cq5,6cqf,6cqt,6cqz,6cse,6csf,6csp,6csq,6csr,6css,6ct7,6cus,6cv8,6cvd,6cve,6cvf,6cvv,6cvw,6cvx,6cvy,6cw4,6cw8,6cwf,6cwh,6cwi,6cwn,6cyb,6cyc,6cyd,6cyg,6cyh,6cyi,6cz3,6cz4,6czb,6czc,6czd,6cze,6czf,6czi,6czu,6czv,6d07,6d08,6d15,6d16,6d17,6d18,6d19,6d1a,6d1b,6d1d,6d1g,6d1h,6d1i,6d1j,6d1k,6d1l,6d1m,6d1u,6d1x,6d28,6d2o,6d3g,6d3q,6d3x,6d3y,6d3z,6d40,6d49,6d4a,6d4o,6d4q,6d4r,6d4s,6d4t,6d4u,6d4v,6d4w,6d50,6d55,6d56,6d59,6d5e,6d5g,6d5h,6d5j,6d5w,6d6t,6d6u,6d78,6d8e,6d8v,6d9s,6d9x,6da1,6da4,6dai,6dak,6dar,6das,6dat,6db3,6db4,6dcg,6dcy,6dcz,6dd0,6dd1,6det,6df1,6df2,6df4,6df7,6dge,6dgl,6dgo,6dgq,6dgr,6dgt,6dgx,6dgy,6dgz,6dh0,6dh1,6dh2,6dh3,6dh4,6dh5,6dh6,6dh7,6dh8,6dha,6dhc,6dhu,6di0,6di1,6die,6dif,6dih,6dik,6dil,6dim,6dj1,6dj2,6dj5,6dj7,6djc,6djd,6dji,6djj,6dk1,6dkb,6dkg,6dki,6dko,6dkp,6dl2,6dl9,6dlj,6dlx,6dm8,6dn5,6dn6,6dne,6dnp,6do1,6do3,6do4,6do5,6dpt,6dpx,6dpy,6dpz,6dq4,6dq5,6dq6,6dq8,6dqa,6dqb,6dql,6drg,6drt,6drx,6dry,6drz,6ds0,6dsp,6dtn,6dtw,6dtx,6dub,6dud,6duf,6dug,6duh,6duk,6dvl,6dvm,6dvn,6dvo,6dxg,6dxl,6dxx,6dy7,6dya,6dyn,6dyo,6dyr,6dys,6dyu,6dyv,6dyw,6dyy,6dyz,6dz0,6dz2,6dz3,6dzq,6dzv,6dzx,6dzy,6e05,6e06,6e0q,6e0r,6e13,6e1a,6e1y,6e1z,6e22,6e23,6e2m,6e2n,6e2o,6e3g,6e3m,6e3n,6e3o,6e3p,6e3s,6e3z,6e49,6e4a,6e4c,6e4f,6e4t,6e4u,6e4v,6e4w,6e59,6e5b,6e5g,6e5l,6e5s,6e5t,6e5x,6e6j,6e6m,6e6v,6e6w,6e7j,6e7m,6e7r,6e7s,6e7t,6e7u,6e7v,6e7w,6e7x,6e83,6e86,6e8k,6e8m,6e8x,6e91,6e92,6e99

,6e9a,6e9l,6e9w,6ea1,6ea2,6eaa,6eab,6eb1,6eb2,6ebe,6ebw,6ecz,6ed6,6eda,6ed
l,6edr,6eds,6ee2,6ee3,6ee4,6ee6,6eea,6eeb,6eed,6eee,6eeh,6eeo,6efj,6efk,6e
g9,6ega,6egs,6egw,6eh2,6ehh,6ei4,6ei5,6eif,6eij,6eil,6eip,6eiq,6eir,6eis,6
eiz,6ej2,6ej3,6ej4,6eji,6ek3,6ekd,6ekn,6ekq,6eks,6eku,6el5,6ela,6eln,6elo,
6elp,6em6,6em7,6ema,6emh,6emu,6en4,6en5,6en6,6enm,6enx,6eo0,6eo8,6eo9,6eog
,6eol,6eox,6ep4,6ep9,6epa,6epy,6epz,6eq1,6eq2,6eq3,6eq4,6eq5,6eq6,6eq7,6eq
8,6eqa,6eqm,6eqp,6eqs,6equ,6eqv,6eqw,6eqx,6er3,6er4,6ert,6eru,6erv,6erw,6e
s0,6esa,6esj,6esm,6esn,6esy,6et8,6etg,6etj,6ets,6ety,6eu6,6euc,6eum,6euv,6
euw,6eux,6euz,6ev0,6evm,6evn,6evo,6evp,6evq,6evr,6ew3,6ew6,6ew7,6ewe,6ewk,
6eww,6ex0,6ex1,6exi,6exj,6exm,6exs,6ey7,6ey8,6ey9,6eya,6eyb,6eyt,6eyz,6ez6
,6ez9,6ezg,6ezh,6ezi,6ezq,6f05,6f08,6f09,6f0y,6f1j,6f1n,6f1w,6f1x,6f20,6f2
2,6f23,6f26,6f28,6f29,6f2n,6f34,6f3b,6f3d,6f3e,6f3f,6f3g,6f3i,6f4n,6f4w,6f
4x,6f55,6f5h,6f5l,6f5m,6f5u,6f5w,6f6d,6f6i,6f6n,6f6r,6f6s,6f6u,6f7b,6f7c,6
f7i,6f7q,6f7t,6f86,6f8b,6f8g,6f8j,6f8r,6f8t,6f8u,6f8v,6f8w,6f8x,6f90,6f92,
6f94,6f96,6f9g,6f9r,6f9t,6f9u,6f9v,6fa2,6fa3,6fa4,6fa5,6faa,6fac,6faf,6fag
,6fam,6fap,6fau,6fav,6faw,6fba,6fbv,6fbw,6fby,6fc6,6fcj,6fcl,6fdc,6fdp,6fd
q,6fdt,6fdu,6fe0,6fe1,6fe5,6fel,6fer,6few,6fex,6ffe,6fff,6ffg,6ffh,6ffi,6f
fn,6ffs,6fg6,6fgc,6fgf,6fgg,6fgl,6fgq,6fgy,6fh3,6fh6,6fh7,6fhk,6fhq,6fhu,6
fi1,6fi4,6fi5,6fii,6fil,6fiv,6fjf,6fjm,6fkp,6fkq,6fky,6fkz,6flg,6fmc,6fmf,
6fmi,6fmj,6fmk,6fmn,6fmp,6fn9,6fne,6fnf,6fng,6fni,6fnj,6fnq,6fnr,6fns,6fnt
,6fnx,6fo5,6fo7,6fo8,6fo9,6fob,6fod,6fpu,6fqo,6fqu,6fr0,6fr2,6frf,6frj,6fs
0,6fs1,6fsd,6fse,6fsy,6ft3,6ft4,6ft7,6ft8,6ft9,6ftf,6ftn,6ftp,6ftw,6fty,6f
tz,6fu1,6fu4,6fu5,6fug,6fuh,6fui,6fuj,6ful,6fut,6fv1,6fv2,6fv4,6fvl,6fvm,6
fvn,6fvo,6fx1,6fyi,6fyk,6fyl,6fym,6fyp,6fyv,6fyz,6fz4,6fzf,6fzg,6fzj,6fzm,
6fzp,6fzu,6fzx,6g01,6g02,6g07,6g0q,6g0v,6g0w,6g0z,6g14,6g15,6g1u,6g1v,6g1w
,6g22,6g24,6g25,6g27,6g28,6g29,6g2a,6g2b,6g2c,6g2e,6g2f,6g2l,6g2m,6g2n,6g2
o,6g2r,6g2s,6g33,6g34,6g35,6g36,6g37,6g38,6g39,6g3a,6g3c,6g3o,6g3q,6g3v,6g
3y,6g46,6g47,6g4m,6g4n,6g4y,6g4z,6g5j,6g5l,6g5u,6g6t,6g6w,6g6x,6g6y,6g6z,6
g7a,6g7f,6g84,6g85,6g86,6g8i,6g8j,6g8k,6g8l,6g8m,6g8n,6g8p,6g8q,6g91,6g92,
6g93,6g97,6g98,6g9a,6g9b,6g9d,6g9f,6g9h,6g9i,6g9j,6g9k,6g9m,6g9n,6g9s,6g9u
,6g9x,6gbe,6gbw,6gbx,6gch,6gcl,6gcr,6gcw,6gcx,6gdg,6gdy,6ge0,6ge7,6gev,6gf
3,6gf9,6gfm,6gfs,6gfx,6gfy,6gfz,6gg3,6gg4,6gg5,6gg8,6gga,6ggb,6ggd,6gge,6g
gf,6ggg,6ggn,6ggv,6gh9,6ghh,6ghj,6ghp,6ghv,6gi6,6gih,6gin,6gip,6giu,6gj5,6
gj6,6gj7,6gj8,6gjb,6gjd,6gji,6gjj,6gjl,6gjm,6gjn,6gjr,6gjw,6gjy,6gl3,6gl8,
6gl9,6gla,6glb,6gmd,6gmq,6gmx,6gn1,6gnm,6gnp,6gnr,6gnw,6gon,6goo,6gop,6got
,6gpb,6gqm,6gr7,6grp,6gu2,6gu3,6gu4,6gu6,6gu7,6gub,6guc,6gue,6guf,6guh,6gu
k,6gva,6gvf,6gvg,6gvh,6gvi,6gvx,6gvz,6gw1,6gw4,6gwe,6gwr,6gx3,6gxa,6gxb,6g
xe,6gxg,6gxq,6gxu,6gxw,6gxy,6gy0,6gy1,6gy5,6gz9,6gzd,6gzh,6gzl,6gzm,6gzy,6
h0b,6h0u,6h12,6h13,6h14,6h1d,6h1h,6h1i,6h1u,6h29,6h2t,6h2z,6h33,6h34,6h36,
6h37,6h38,6h3k,6h3q,6h41,6h4a,6h4d,6h4o,6h4p,6h4q,6h4r,6h4s,6h4t,6h4u,6h4v
,6h4w,6h4x,6h4y,6h4z,6h50,6h51,6h52,6h5w,6h5x,6h75,6h76,6h77,6h78,6h7b,6h7
d,6h7f,6h7j,6h7l,6h7m,6h7n,6h7o,6h7u,6h7y,6h7z,6h8c,6h8s,6h96,6h9b,6h9v,6h
9x,6ha4,6hah,6hai,6haj,6hax,6hay,6haz,6hb5,6hb6,6hb7,6hbm,6hbn,6hck,6hcu,6
hcv,6hcw,6hd4,6hd6,6hdn,6hdo,6hdq,6hf5,6hgf,6hgg,6hgi,6hgj,6hgr,6hgs,6hgv,
6hgx,6hgy,6hgz,6hh3,6hh5,6hhg,6hhh,6hhi,6hhj,6hhp,6hhr,6hjj,6hjk,6hk3,6hk4
,6hk6,6hk7,6hke,6hkj,6hkm,6hkn,6hks,6hkx,6hky,6hkz,6hlb,6hld,6hle,6hlx,6hl
y,6hlz,6hm1,6hm2,6hm4,6hm6,6hm7,6hmb,6hmg,6hmp,6hmr,6hmt,6hmx,6hmy,6hni,6h
oi,6hol,6hop,6hoq,6hor,6hot,6hou,6hov,6hoy,6hp0,6hp5,6hp9,6hpg,6hpw,6hq3,6
hq4,6hq7,6hqy,6hr2,6hro,6hrp,6hrq,6hrt,6hrw,6hrx,6hry,6hrz,6hs0,6hs4,6hsh,
6hsk,6hsz,6ht1,6ht8,6htc,6htd,6htg,6hth,6hti,6htn,6htp,6htr,6htt,6htz,6hu0
,6hu1,6hu2,6hu3,6hub,6huc,6huq,6huu,6huv,6hv0,6hv2,6hv4,6hv5,6hv7,6hva,6hv
h,6hvi,6hvj,6hvr,6hvs,6hvt,6hvu,6hvv,6hvw,6hwu,6hwv,6hwz,6hx1,6hx5,6hx8,6h
y2,6hy7,6hys,6hza,6hzb,6hzc,6hzd,6hzp,6hzu,6hzv,6hzx,6hzy,6i0b,6i0c,6i0k,6
i0p,6i0r,6i0x,6i0z,6i10,6i11,6i12,6i13,6i14,6i15,6i16,6i17,6i18,6i1r,6i3s,

6i3u,6i41,6i4x,6i5g,6i5i,6i5j,6i5n,6i5p,6i5y,6i61,6i62,6i63,6i64,6i65,6i66
,6i67,6i68,6i74,6i75,6i76,6i77,6i78,6i7a,6i8b,6i8l,6i8m,6i8t,6i8y,6i8z,6i9
6,6i97,6i9a,6ia7,6iae,6iam,6iar,6ib0,6ib2,6ibk,6ibl,6ibs,6ibv,6ibx,6iby,6i
bz,6ic0,6ic2,6ic5,6ic6,6ic7,6idb,6idg,6idz,6iez,6if0,6ift,6iht,6iik,6iil,6
iin,6iiu,6iiv,6iiw,6ijh,6iji,6ijl,6ikm,6ilq,6ilz,6im4,6im6,6imb,6imd,6imi,
6imo,6imr,6imt,6ind,6ink,6inm,6inz,6ior,6ios,6iot,6iou,6iph,6ipi,6ipj,6ipk
,6ipl,6ipm,6iqg,6iql,6irt,6isd,6iso,6itj,6itp,6itq,6iuo,6iup,6ixd,6iyv,6iy
w,6izq,6j06,6j0g,6j0k,6j0o,6j10,6j1l,6j3o,6j3p,6j5l,6j63,6j6m,6j72,6j7e,6j
7l,6j81,6j8q,6j8r,6j9w,6j9y,6jad,6jag,6jam,6jan,6jao,6jap,6jaq,6jav,6jaw,6
jax,6jay,6jb0,6jb4,6jbb,6jbe,6jdi,6jdl,6jfk,6jib,6jid,6jij,6jio,6jjm,6jjn,
6jjz,6jki,6jmf,6jn3,6jn4,6jn5,6jn6,6jno,6joe,6jof,6jon,6jpj,6jse,6jsf,6jsg
,6jsn,6jsz,6jt3,6jtc,6jut,6jwa,6jz0,6k04,6k05,6k0j,6k1q,6k1s,6k2n,6k3l,6k4
r,6k4t,6k4x,6k5r,6k5t,6k9u,6kdi,6kdx,6kdz,6ke0,6keb,6kjd,6kjf,6kla,6klk,6k
mj,6knh,6kqi,6kzc,6kzd,6lpr,6m7h,6m7i,6m87,6m88,6m89,6m8a,6m8b,6m8c,6m8d,6
m8e,6m8q,6m8w,6m8y,6m95,6m9c,6m9d,6m9f,6m9l,6m9t,6ma1,6ma2,6ma3,6ma4,6ma5,
6maj,6max,6mbo,6mbp,6mck,6md0,6md4,6md6,6md7,6md8,6mdb,6mdc,6mdd,6mdq,6mdu
,6me1,6me2,6me3,6me4,6me5,6mep,6mey,6mg5,6mh1,6mh7,6mhb,6mhc,6mhd,6mhm,6mi
6,6mia,6mil,6mim,6min,6miq,6miv,6miy,6mj4,6mj7,6mja,6mjf,6mji,6mjj,6mjq,6m
ju,6mjw,6mkq,6mku,6mkw,6ml9,6mla,6mle,6mlf,6mlg,6mlh,6mli,6mlj,6mll,6mln,6
mlo,6mlp,6mlw,6mm2,6mm5,6mmc,6mmo,6mmq,6mnc,6mnf,6mnp,6mnv,6mny,6mo0,6mo1,
6mo2,6mo4,6mo5,6mo7,6mo8,6mo9,6moa,6mob,6mod,6mom,6moo,6mq3,6mqc,6mqe,6mqm
,6mr5,6mrg,6ms1,6msa,6msc,6msn,6mso,6msy,6mt0,6mt4,6mt5,6mt6,6mtu,6mtv,6mu
1,6mu3,6mub,6mul,6mum,6mv3,6mvu,6mvx,6mwe,6mx3,6mx8,6mxb,6mxc,6mxd,6mxe,6m
yn,6mzq,6mzw,6n0j,6n0k,6n0m,6n0p,6n0q,6n17,6n19,6n3e,6n3f,6n3k,6n3l,6n3n,6
n3o,6n3v,6n3w,6n3x,6n3y,6n3z,6n48,6n4b,6n4e,6n4t,6n53,6n54,6n55,6n5c,6n5f,
6n5g,6n5h,6n5x,6n69,6n6o,6n77,6n78,6n79,6n7a,6n7b,6n7c,6n7d,6n7q,6n7y,6n7z
,6n82,6n83,6n87,6n8x,6n8y,6n92,6n93,6n94,6n95,6n96,6n97,6n9h,6n9l,6n9p,6n9
t,6nad,6naf,6nao,6ncj,6ncn,6nco,6ncp,6nd3,6ndl,6ne5,6nel,6nem,6nfg,6nfh,6n
fi,6nfn,6nfo,6nfy,6nfz,6ng0,6nj0,6njh,6nji,6njj,6njz,6nk0,6nk1,6nkp,6nlj,6
nm4,6nmb,6nng,6nnr,6no9,6np2,6np3,6np4,6np5,6npe,6npf,6npi,6npm,6npp,6npt,
6npu,6npv,6nrf,6nrg,6nrh,6nri,6nrj,6nsp,6nsq,6nss,6nsv,6nsx,6nt2,6ntb,6nti
,6ntj,6nu1,6nu5,6nv7,6nv9,6nvg,6nvh,6nvi,6nvj,6nvl,6nw3,6nwc,6nwk,6nwl,6nx
z,6ny0,6ny4,6nyh,6nyv,6nyw,6nze,6nzf,6nzg,6nzh,6nzk,6nzm,6nzp,6nzq,6nzr,6n
zt,6nzv,6o0h,6o0k,6o0m,6o0o,6o0p,6o1g,6o21,6o2p,6o3w,6o3x,6o3y,6o48,6o4w,6
o4x,6o50,6o57,6o5a,6o5g,6o5t,6o5u,6o5x,6o68,6o6f,6o6g,6o7g,6o8i,6o8u,6o94,
6o95,6o9b,6o9c,6o9d,6o9x,6oa1,6oa3,6oac,6oag,6oah,6ob0,6oco,6ocq,6ocu,6ocw
,6ocz,6od6,6ode,6odz,6oe0,6oe1,6oe3,6of5,6oh2,6oh3,6ohd,6oht,6ohu,6oi8,6oi
9,6oie,6oim,6oin,6oio,6oip,6oiq,6oir,6oja,6oko,6olx,6om2,6om4,6om8,6omu,6o
os,6oot,6oou,6ooy,6ooz,6op0,6op9,6oqb,6oqc,6oqd,6oqn,6orr,6os5,6os6,6otg,6
ott,6ouv,6ovc,6ovz,6ow2,6ow7,6ox0,6oxl,6oxp,6oxq,6oxr,6oxs,6oxt,6oxu,6oxv,
6oxw,6oxx,6oxy,6oxz,6oy0,6oy1,6oy2,6oyh,6oyt,6oyw,6oyz,6oz6,6p10,6p11,6p12
,6p14,6p1d,6p1l,6p3p,6p3t,6p3u,6p3v,6p3w,6p5o,6p5v,6p5w,6p7p,6p7q,6p83,6p8
4,6p85,6p86,6p87,6p88,6p89,6p8a,6p8x,6p8y,6p8z,6p9e,6pdj,6peb,6peg,6pek,6p
eu,6pf3,6pf4,6pf5,6pf6,6pfj,6pg3,6pg4,6pg5,6pg6,6pg7,6pg8,6pg9,6pga,6pgb,6
pgc,6pge,6pgo,6pgp,6pgx,6phr,6pht,6phx,6phz,6pi1,6pi5,6pi6,6pi7,6pia,6pic,
6pid,6pii,6pit,6pk7,6pka,6pl1,6pl2,6pl3,6plf,6plg,6pm9,6pml,6pnm,6pnn,6pno
,6poh,6poi,6poq,6ppy,6prc,6prf,6prg,6prt,6ps0,6ps1,6ps3,6ps4,6ps5,6ps6,6ps
9,6psb,6pt3,6pu3,6pve,6pvs,6pvu,6pvv,6pvw,6pvx,6pvy,6pvz,6pxc,6py0,6pya,6p
yb,6pyc,6pyd,6pyr,6pys,6pyu,6pz4,6q2y,6q30,6q35,6q36,6q37,6q38,6q39,6q3q,6
q3y,6q3z,6q4e,6q4g,6q4q,6q54,6q5b,6q60,6q6f,6q6m,6q6o,6q6y,6q73,6q74,6q7a,
6q7h,6q7v,6q7w,6q89,6q8a,6q8b,6q8c,6q8k,6q8p,6q92,6q96,6q9h,6q9l,6q9o,6q9q
,6q9s,6q9t,6q9u,6q9w,6q9y,6qaa,6qab,6qac,6qad,6qae,6qaf,6qas,6qat,6qau,6qa
v,6qc0,6qcg,6qcj,6qdx,6qe0,6qe5,6qed,6qef,6qeg,6qfe,6qff,6qfg,6qfh,6qfq,6q
ft,6qfu,6qfv,6qfw,6qfx,6qg4,6qg7,6qge,6qgf,6qgg,6qgh,6qgj,6qgk,6qh9,6qha,6

qhb,6qhc,6qhg,6qho,6qhr,6qi4,6qi7,6qk8,6ql1,6ql2,6ql3,6qln,6qlo,6qlp,6qlq,
6qlr,6qls,6qlt,6qlu,6qm7,6qmd,6qme,6qmj,6qmk,6qmr,6qmt,6qmu,6qpl,6qqq,6qqt
,6qqu,6qqv,6qqw,6qqz,6qr0,6qr1,6qr2,6qr3,6qr4,6qr7,6qr9,6qra,6qrc,6qrd,6qr
e,6qrf,6qrg,6qri,6qs1,6qs5,6qsz,6qtm,6qto,6qtq,6qtr,6qts,6qtw,6qtx,6quv,6q
uw,6qw7,6qw8,6qw9,6qwa,6qwb,6qwi,6qxa,6qxd,6qxj,6qxs,6qxu,6qyk,6qyl,6qyn,6
qyo,6qyp,6qz5,6qz6,6qz7,6qz8,6qzb,6qzh,6qzr,6r0k,6r0s,6r0u,6r0v,6r0x,6r11,
6r12,6r13,6r18,6r1a,6r1b,6r1c,6r1d,6r1k,6r1w,6r1x,6r49,6r4k,6r4s,6r4v,6r5f
,6r7d,6r7j,6r7k,6r7r,6r8i,6r8l,6r8o,6r8p,6r8q,6r8r,6r8w,6r9s,6r9u,6r9x,6ra
1,6rfi,6rfj,6rfn,6rfw,6rhe,6rih,6rj2,6rj3,6rj5,6rj6,6rjp,6rk4,6rln,6rmf,6r
ml,6rmm,6rn6,6rn7,6rn9,6rna,6rne,6rni,6rnt,6rnu,6rot,6rpg,6rqk,6rr0,6rrm,6
rsa,6rtn,6rtw,6rvf,6rvk,6rvl,6rw1,6rz6,6s07,6s0e,6s1s,6s43,6s4n,6s4t,6s4u,
6s55,6s56,6s57,6s5k,6s7k,6s7s,6s7z,6s88,6s89,6s8a,6s90,6s9b,6s9c,6s9d,6s9w
,6s9x,6saf,6sal,6sbt,6sd9,6sdc,6sdd,6sde,6sen,6seo,6sfc,6sfi,6sfj,6sfk,6sg
e,6skb,6skc,6skd,6slg,6sq0,6ssy,6st0,6st3,6std,6stl,6suo,6sy7,6sze,6szj,6s
zp,6t1i,6t1j,6t1l,6t1m,6t1n,6t1o,6t6a,6te6,6tel,6ten,6tim,6tl2,6tld,6tyz,6
u26,6u5m,6u5y,6u6w,6u7o,6u7p,6u80,6u8b,6u8l,6u8o,6u9v,6ud2,6udi,6udt,6udu,
6udv,6udx,6udy,6ueg,6uf0,6ufn,6ufo,6ugn,6ugo,6ugp,6ugq,6ugr,6ugz,6uh0,6uhu
,6uhv,6uii,6uil,6uim,6ul8,6un1,6un3,6upj,6uvp,6uvv,6uvy,6uwp,6uwv,6uyx,6uy
y,6uyz,6v1c,6v5l,7abp,7cpa,7gch,7gpb,7hvp,7kme,7lpr,7prc,7std,7upj,830c,8a
3h,8abp,8cpa,8gpb,8hvp,8lpr,966c,9abp,9hvp,9icd,9lpr

List S3. List of PDB IDs included in the training data of SurfDock.

3dpf,2zy1,6h77,5c0m,4o2c,3a2c,3b2t,1v2q,4q87,4j28,3efw,4mi6,3k8c,3s54,2fda
,2q55,5ylv,4ozo,3fzn,3eoc,4tn2,3hfv,5gg4,5svi,2r58,5d1n,4yqv,1hs6,3zxh,1ft
7,4hww,6b1j,3di6,5jaz,5ema,6gip,5am5,4bnt,1fq6,3pqz,6eil,2q11,4nw7,4ahr,1p
1o,3le8,3nzk,1eub,1eoc,3std,3qw8,4za0,5l8a,5teg,5u6j,3g2n,2wp1,1zyr,1xs7,4
m6q,2jq9,4m5h,4d63,3shb,3i6m,4hw3,6eq5,3hqy,3zos,6h96,2gv6,5yjb,4lm4,4lwv,
3g30,5f32,4dkr,6b4l,4j9a,5w86,4mpn,5m51,1ro6,5q0m,3bea,1mu6,2wtd,3sr4,6dqb
,5hh5,5q0e,2r03,4xv9,4y0a,5ota,3u5l,3m3o,4idz,2w47,2g5u,6cec,5wzs,3iu7,5mk
y,3ibc,2qwf,3p76,4np3,4kb8,1uyi,4tkf,2fai,2xvd,5ar8,2lzg,3e62,1p6e,5wqa,1p
02,6gjr,1nhu,4yyl,3jzq,4pce,4gvu,4n7g,3s9e,3k3b,1pmn,1ogd,2j4i,1oss,1v0k,6
cn6,3k3e,2btr,1ppk,6egw,4zx1,4zfi,5yso,2rfn,5cep,2xyu,3eou,4mme,4fnn,4og7,
4gkh,6d1l,6dlj,5i94,1eb2,4qzw,3fu0,4aif,5eec,2qlj,5cfb,5sy3,1mhw,2flu,3e6y
,4wiv,5oot,4bae,6bfd,3po6,2wo9,1rp7,1l2s,5ab1,3pce,2w6z,1iew,3e85,3e12,5ar
f,4jgv,1ikw,6goo,4zdu,3wcb,2w1h,4x2i,6ggv,3h0b,5nwe,1o46,5f2u,2feq,2jnw,3g
pj,5uex,1ndw,1zhk,4mcd,3r4m,4cps,3gcp,2g9v,5try,4c2v,4og3,3otq,2yns,4m0e,5
tkj,2m3z,3pa5,3iww,5o0b,3m58,5lz2,5u6k,4yw6,3ewh,3wc7,1rhk,3gi4,2wd7,3jq8,
5yr5,6fnf,3hx3,4mz6,1ol2,4aa5,1rry,1ozv,5jf6,4mnq,5wa5,3nlb,3f6e,2xu5,3wk7
,4p6w,2pmc,3byo,5ehe,6chq,6c8x,3pp1,3uev,3ad7,5aqv,3qzq,2z97,5d1r,5ajy,4k6
7,2w1e,5j3v,3upk,4gj8,1td7,5lz9,6erw,4rhx,1hxw,6cbf,5qal,1q8w,4mf0,5t1s,1f
zk,4tju,5t2y,4el5,2v87,1epo,2r5p,4pis,3ehy,5erg,2r1x,2f8i,2wi7,5wre,3dek,3
hp2,4bcw,4uau,5ap3,3udy,2ksa,5ekg,5lh8,2uw7,3uvq,2glp,3m3r,6bsx,2ddf,6eqm,
3ufl,2mwp,1h4n,5f4r,4ybj,4kz8,2z60,4ogi,5ufp,4uiw,3igv,5b56,1bn3,2xg9,1u59
,4m5j,5oua,5qaa,6h7j,2o7v,5v7t,2r02,2j94,5o87,1o3k,4mr4,5am7,6g35,1ljt,3sr
v,4bi6,2yoh,4zed,5tln,3rkb,5jiq,2ll6,1tkz,2vj6,3qrk,3c45,4pks,3nf3,2oaz,3p
wk,3ryy,2y7k,6dm8,5ism,5i7u,2qtn,5cuu,5n1y,4kot,5wa1,2wyi,188l,3h2o,1sw1,1
zuc,2nnq,4l8m,5j75,4afe,5nwi,2n06,3l4w,3ibl,4l31,4ruy,4ufm,5sxk,4hnn,5q0x,
4zij,3fdn,5fe6,3nuj,5h8x,5ets,4x5z,1w3k,3hs9,3ro0,3zi8,1nl4,5cao,4gjd,2p3d
,2z8e,3w2p,1f28,2iw6,1h9l,4o78,1soj,3krw,5g4n,3t4v,5drr,5o0a,4c4i,2vwx,6mi
q,6guk,5c3k,5fhn,3o75,5kap,4zo5,2jko,3iss,4eg6,4fcd,3v7s,4bcf,2qyn,1ikx,6f
6i,5os5,1om2,6axb,3s3n,1fl6,4luo,1qiw,5f2w,3udj,5tbj,5k0c,5ndf,1ek2,4zb8,3
r9o,5qah,3avb,4c52,4ybi,5mi6,6fu5,1yc4,4ymq,1xn3,3dpo,4z7q,5ehn,5mri,2vcw,
2byp,5du8,1i7z,1ec3,5jmx,4yxi,3iw7,3l4v,3wcf,5igk,1sz0,4x6i,1wum,1d3d,3rv6
,1x7r,2c57,4u82,6cwh,2vrj,2xag,5nw7,5qau,4ocz,3chg,5oh1,1hkm,3ayd,3mn8,5ng
f,4bo6,1b57,5wzt,4uzd,1cyn,3eys,4umc,6guh,2e99,2xb8,5tus,6ap7,4ajn,4od7,6g
2l,5v40,5wjj,2bow,3kc0,3zs1,4igq,3b24,5e8r,5a4l,4ci1,3qd0,2ohv,3uw5,3lp4,3
ftw,6c5q,4e0x,4hbw,2yoj,4b2i,3mkn,1i5r,5tig,5bqs,5j2x,1oz0,5a2s,3fkv,3qnj,
5w5o,2cht,5dj5,5oje,5o9y,4hxs,5t36,5gmh,4eh9,3kr2,3rk5,2b54,4ufh,2zxa,4mji
,4e70,4huo,3zrk,3wb5,2iw8,4ge4,6ccs,5nwc,5lmb,4i6h,4dhu,5y6e,5bvn,4lxd,3b3
s,5j9f,3jzp,2no3,1ouk,5epk,1fo2,1g7g,4dve,6au2,4wt6,3w2q,5tdr,4xx9,1jg0,2n
27,5lyn,1p1q,1hi3,2wih,4hym,4ew3,5ixq,1h60,3ppk,4x8s,5nzn,3ddf,1yvm,1kv2,4
djq,3sym,4fz3,1ze8,5ia1,2qcm,5m9d,2erz,4g0l,5tqg,2i5j,3ow6,1met,3r42,5tdi,
3i97,1alw,1doj,3f5j,2r5q,6eir,2ie4,5cav,1n4m,5wj6,2o7n,2r4f,5nkn,2g6p,1bnu
,4uc5,3kze,5dg6,1nym,6cki,4kmz,6mjf,4ch8,6hsh,1txr,4zj8,6ggn,4qzx,4qsu,5et
y,1o5a,4otf,1pgp,5wex,4p4b,4h4m,3k54,4j53,5dva,1e3v,4wop,1c3i,4jls,5mxv,4q
rc,3fvn,5ivn,4xu1,2o4n,1m5b,3t1a,1mqi,5n84,5ifu,5eie,5lgr,5unf,5w5u,5tq1,3
dkj,5ye8,1br8,3tsd,6dvn,1oxg,4kz5,5v3h,5in9,4avi,220l,4b7z,2xbv,3twx,5zo8,
2w4i,5opc,4q8y,5acy,5ncz,3in4,3arp,4rt1,4mq6,6ey7,3uzd,2b9a,5o4z,2ow0,6f5w
,1x7q,5agv,5pzm,6afh,4z1e,5flp,5xsr,1b4z,5f1r,1o2g,1uwh,3bmo,1p19,2ans,6fb
v,4lrr,1gi9,4xuh,2weo,5xpi,4btm,5h0e,4gye,6bgu,5wlt,5oul,1t49,5k1v,5lz4,1f
sw,6cvx,6c3e,6cj5,5fqc,1cze,3uwl,5l3g,5jnc,3ps6,6csr,6bw4,3hau,3ud9,6csp,5
uyu,5lqf,4bfz,2xp6,3sjo,5os3,4z8d,5un1,6esy,3kjq,3sl4,5mgl,5dp5,3n0h,5fao,
4dmy,6dxl,4qw0,3q92,3rwf,2xke,4gfo,3t03,3pd8,3f18,4utr,4cu7,5n1s,3wkb,2zdt
,2yit,4cmt,5dpa,4rt0,2y5h,3cde,5lud,3vva,2wev,5mrm,4ypf,5ea4,3fr5,4tkh,5ty

i,1db1,4hxj,3hu1,5ftg,2eh8,1jiz,1gww,5nih,1rr6,4fp1,4y24,1fki,3lea,4lch,2x
k1,4qjr,4os1,4pft,5eb3,5qad,3cpc,3c94,5jhb,6b22,3vfj,6hmx,1kuk,2gh9,1xh6,3
eyl,5hng,3nes,3i1y,2anl,4xit,3n2e,2n0u,5hdx,4zt6,4f6v,5n7g,4mc1,4yc9,3q6w,
2cmb,5g4m,3pww,5d7x,4cpr,3muf,5f95,5o9o,5e2w,2chx,2pk6,3bet,6cje,2rd6,5khk
,4eh5,1i9l,1swg,4q4o,1kf0,1llb,5aep,2zp0,3zze,5csp,5eyk,3u7n,4z0e,5fh6,5kj
2,2o4k,5llg,4b5t,3li2,3of8,3mvh,1jut,1auj,5awj,5x73,1eol,1f90,4crd,1d2e,2o
ht,1ua4,2h9m,5byz,3rse,4mp2,3hhk,5tur,2xxr,2r3w,4u6e,4qlt,3db8,2bub,1ths,3
s8o,3x1k,3eg6,1jaq,3anr,3ua8,2kfg,4m3g,3d3x,5a5v,3nuu,1jh1,1iy7,5dlz,4wpf,
4pvy,1sve,4qmy,1pyg,5drq,4ivt,2gcd,1azg,3wjw,2qiq,4leq,2qfu,1thz,1y3a,5yjp
,5cgv,1oi9,3cd5,3w1f,2v12,5f37,1e03,3b67,4q8x,5uk8,4v25,3u8k,5l8o,5hlb,4qf
n,4li7,1dth,4gkm,6f3e,4rn4,3vtb,5l6i,4lrh,4jvq,5est,3gtc,2jbu,2yfx,4yx9,5b
s0,5ea3,1vyf,5a4e,3kku,3cyw,3tvx,5mkj,6b4d,4bny,5j8x,5tza,1uk1,5yg2,1d6v,5
t4e,2j4g,5tci,1z9y,2ca8,4hyf,5mg2,4bcq,1lqf,6b3v,5htc,2qh6,4pul,1gj7,4mmf,
3sfg,5ap2,2oi9,4h81,3s77,1awh,3s74,3oyp,4u5u,6euz,6eq3,4obq,4u4x,2r23,4tjw
,4anq,2a4z,2mwy,4pzh,5m7m,4mzj,1o3l,3d62,2xow,5hog,3rwq,2kmx,4fpf,5ajx,1f5
k,4de7,5c7c,3q77,4pnr,5tkb,5hz8,2vle,5ho7,5xmx,2xpk,6mdq,3tge,5fls,3oku,5j
r2,3e0q,2l98,2uzj,5csz,5yr6,3f7i,1ex8,6ft7,2bdj,5l7g,1cnx,1mmp,5j8m,2ay2,6
fnq,5eng,1pg2,4a6v,3ivv,2zq2,4wag,3s7a,4is6,1lhw,5tq8,3v51,4w9x,5lto,1o86,
5a69,4zg7,4unq,2q88,5c1m,6bgy,4fil,2qi1,2x7o,6exj,3nkk,1q65,2xxw,3l7d,1om9
,4jq7,1lf2,4xg4,3sgx,1sdt,3ow4,4kp8,2cem,5nzo,3oaw,2wwj,2v6n,4g11,1lf3,3lj
z,1amn,6hm4,3jq9,1qx1,2wi5,4old,1g5s,3tic,3cs8,4er4,5kr0,2pe0,5abf,3v04,4e
6q,2yc3,3bys,5jeo,4b00,4bek,4fci,4qna,5eh5,4r02,5ef7,4hu1,5v5n,5ehp,5yr4,3
dt1,4cd0,6gl8,3uvl,3v0l,5ne5,4nvp,4qok,4ipj,4zyi,1o3j,1lrt,3iog,5zwe,4hbn,
2gz2,5tco,3gc5,4l6t,5mnx,5m23,2etr,5gwy,3b5j,1oai,2ath,2v11,5wuk,2ym6,4mwu
,5u69,4i8w,6g86,4f7l,2yi5,4nan,1j37,2xup,2byi,4f63,3m3x,2bpm,2wb5,6fel,3td
c,4z1s,4fgy,3cyz,6bh1,3m8p,2df6,3jrs,2wmv,6fau,5oah,3d50,3aza,1uwt,4nct,1j
qe,4nb3,4y3b,1okw,1db4,4n98,3inh,6hzp,1hdt,6aoy,4o3u,5es1,5i9i,6ccm,5nev,5
wp5,4bo7,5byy,3su2,4hlf,4c1g,3sww,2os9,4men,2wxd,4qq4,5eei,4hnf,3b26,3eht,
3oqf,3dd0,3si4,4v1f,4e6d,2c8w,1jyi,4ob1,5x4n,5alc,2pbw,5eym,4e28,2xui,4qtl
,5jz9,3chr,2p2a,1v1j,4o3b,5v83,5v5e,3byu,7abp,5jga,3sw2,5jgb,4dtk,1q91,3cq
u,1nje,3vhd,4knm,4yrt,1bgo,5ih5,6afe,4res,1akt,1kyv,1sqn,4oas,5u5l,1v79,3h
p5,5ivc,1q84,3avg,3tws,1rmz,4rse,5zae,5w2q,5glu,5b4w,5f62,4y6r,4mha,5etk,5
nzm,4o2b,4cwq,2pj8,3lvw,2fw3,1dwc,3zln,3ejq,2f6y,6bic,4p0x,2cct,3dxh,2xhx,
1d3q,3qbh,5tkt,4acd,1yt7,5aki,3zy2,4tw7,3b3w,1nkm,5uoo,2l75,4l7u,4iut,1pbq
,4dow,3hab,1owe,6ft3,4y2q,2hs1,6gu6,4dpi,5aac,4j2c,1s50,6nao,5fox,3jvs,4oz
l,1zub,5wxp,5iuh,5oh7,2c90,5owl,3oof,4ona,4ydg,1h08,1uk0,2fxs,3f37,4xtz,4m
ww,6gg4,4zz3,5cf4,3blr,4twc,3zsz,4er2,4uuh,1i8j,6faw,5opv,3voz,3d1g,5q0n,3
kfc,3qqs,1oay,1uvs,5os4,4abe,1r0x,4jwr,1l6m,3nf6,3qip,1nlt,3zdg,3o84,3ehn,
3k16,5szb,2j2u,5aiv,2weg,3dz4,4jdf,1ttm,3wyy,3ifo,5j41,3fl5,1q1m,2y80,3fr4
,3o56,1yrs,2gvv,3uxk,2zq0,5uac,3sfi,2wk6,184l,6gvz,6dik,3u8w,3rxb,2qpq,3nw
e,13gs,4gpl,4rxz,4qbm,5y9l,4nh8,4rra,5ey8,4j03,2ou7,5tt3,1uwf,4dgr,5xzr,4m
w4,3n5k,4umq,2l6e,6drg,6ghp,2ksp,1sps,5hna,6gnp,3dxk,5eom,1e34,2xfi,2itp,2
zlf,4iva,2y4m,4o1b,2o9v,4i7f,4ezw,1k4h,2xgm,4nud,3m55,6ezg,4x34,1czo,5fqr,
5he4,2h6k,3fmr,4kzb,2g9q,3tpx,4hpy,2bo4,4nhx,4mvh,2nq7,3n5u,3rjw,3emg,2xm9
,1kzn,5faq,5f1u,1c6y,4j8g,5lvr,1xuo,2vgo,1grp,4uxb,2rol,5cbm,6hke,5tr6,1uy
c,3d6o,5awu,4wz8,5egm,1duv,6b4n,1t7j,5aqj,4jh0,4q7s,5cpr,1bqm,1g9c,4fem,4w
nk,4uv8,5hgq,1ayu,6co4,6ck3,3uzj,2qwe,3u2q,1nl9,6bd1,5yzd,4r92,2i0j,4hvh,4
ool,3s0n,1ba8,3b4p,2ea4,5k76,2xzq,4yur,1pq6,2y81,3f8s,2hwh,1q4w,4h39,3zmh,
1ftl,4ok5,2qcn,3qtf,1g52,3vw0,3i4a,5x79,2am2,2ym7,5odu,3adu,6cea,3t6y,4pgc
,1k1j,3d14,3el4,4nyf,2y0j,2w1c,5l7k,4n6g,5cp5,3ekr,2isc,5cbs,5hvu,4n00,5mp
n,5zoo,3v43,1t69,1oj5,5lle,5wef,2ya8,4ezk,2c6o,5vrl,1b4d,5za8,1i80,2ccb,6c
e2,4cfx,3m5e,1k22,6eea,3s3m,5t4f,2xyn,6br3,3krl,3upy,2rm0,5q1f,4app,7gch,5
hx8,4ok6,4zla,5g3w,1h27,1fkb,1idg,5c8n,1h46,3w33,3rzb,4xg6,2rib,2uuo,1r1h,
5agu,6dcz,1r58,1nlo,3g70,2ofu,4k6z,4x13,5ihc,2yiw,6bgw,6gji,1tve,1gpn,5tq5

,4ujb,4l33,6cqz,4avh,5uga,5tzy,5op6,1m7d,4aia,2bxt,4qgh,3uuo,5vkm,4jr3,1wd
y,4o4r,2c3j,2r9m,5ual,3vfq,4c16,6h7l,3tv6,2pyn,2bj4,4qvy,4abk,3m53,3cwk,3d
52,1a4q,4rvl,1d04,3thd,1ogz,3luo,2ftd,4ran,3uph,3c8b,2xp3,2vtl,1fj4,2zm3,3
rxl,1xmy,3uo5,1q1y,2ql9,1bhf,2jg8,6fuh,2q2n,4aof,3ff3,1heg,4cae,6fkq,4i9i,
3ask,1dbj,4zbf,2io6,3d1y,5t2l,3kwf,1juj,3i0r,4gui,2hy0,3i7c,6g9m,3nnx,5u48
,2v2v,6f5h,5mwh,5m0d,5fue,3k5k,5edi,4d2p,2cm7,8cpa,3doy,3k99,1vyg,6ckw,6bm
v,5m4q,5j4n,4tpt,2jbv,3uqr,4di2,5ue4,5enj,3csl,3m35,4a9t,5khx,5f0h,4wq3,4k
tu,2qbr,1sbr,2f6z,6cgt,1t4v,5nra,3fed,5l2o,3mb7,4bzn,1efi,3nrm,5kjm,2cle,1
j07,3h9k,4d83,5n4s,5yie,5yz7,5cr7,4e35,6di0,3omm,4w9n,4z1j,6b98,4x61,4x6m,
2pu1,4q1s,3n7a,4qbb,6fng,5ya5,3qiz,5awt,3sv8,3doz,6dlx,4rkx,3h91,4uma,1lvc
,1uy9,5gso,5gsa,5drc,4j5e,2o22,4loo,5t66,3rz0,5unj,4g17,6bhv,3cii,5qcl,4nw
6,4qqi,3r0i,5uff,4lbp,4bqy,4l51,5akh,2yjb,3dx0,3v49,3g42,5xpp,2c4g,2bz6,4f
ny,3x00,4nat,6f8g,4ppc,3lpb,3tlh,4bcs,3nok,3lfs,3o9h,2vf6,3we4,2yig,2kgi,5
llc,2jgs,1fkh,5ogb,5w8i,1a42,5web,2llq,5j7b,2c6l,4u0e,2g9r,1lt5,5ai5,3khj,
6hh3,4uac,5nob,1ynd,2on6,6hd4,3msj,5ut4,2wkz,5jvi,3gww,4uja,1pq3,4u5v,4z83
,6dh8,1h36,3vjk,1unh,3hv7,2vxj,3l38,1a08,5lgo,1fgi,5fqs,5a5p,5oss,4o0z,1gv
x,1xur,5vqe,2j7w,2adm,3b1m,5zah,1iwq,4zx8,3wkd,5mtv,5myd,3t6r,2c6n,2c6e,2g
pp,3opm,5n9r,3r0t,6gue,4b70,3gnv,5nyz,1fh7,5wae,2bvs,1tow,1toj,5een,5fog,6
e9a,5jek,1jet,5qck,6coj,1if8,5t6g,4b85,2fm0,5eje,1ezf,3twd,3mo8,4rvt,6bbu,
4ejl,1waw,4heu,4hgs,6b67,2cmc,3arv,4r4t,1j80,5ads,6abp,3el7,6fex,4m5o,3qgw
,5t8o,3pcn,3nw3,5w84,3bqn,4pin,2w3o,5yic,4lwh,3vo3,4unr,4cp5,4b73,6c6o,1xz
8,5zo9,2qn3,2g1r,4xc2,1oe8,2z94,5aic,4m12,1sld,2h96,6cn5,3bvb,5x27,3usx,1d
dm,3fal,2iit,3fzt,5vgi,5jv1,1py1,5ktx,4b35,1m0q,5kjk,2vey,6dkg,5ei3,6gbx,3
e01,1p03,4zuq,4wbo,4rfd,3zlo,5alb,5b5o,3liw,2mwo,4yjl,4ab8,5nau,4at4,1ke0,
1zz2,2ccs,5hz6,2cll,4m2v,1q3d,3ozt,4ge7,3pkd,5t70,3ts4,5q0y,5q1a,3ogm,5t18
,5c7d,3g2k,1ikv,2j7b,4obp,4k6y,2fb8,3vbd,5ung,4b32,2qlq,3jzs,3tiz,1v2k,6ee
2,3zzf,1hpv,5xo2,5e2l,1jn4,5aly,2jjr,4agm,3rwp,3r7r,3zbf,4url,2wm0,6dcy,2b
rg,5dlx,4g2y,5jzs,5fd2,5nqr,4jpe,3tkm,5ti0,5lxp,3avi,5zuj,3p3h,3d9v,4gs8,4
cwn,5q1i,6b7a,1gjc,4dbn,5yp5,5ukj,6arj,4iuo,4ibk,5ali,6fgq,1b5h,2bvx,2y36,
3f81,4e3f,3t84,4na7,2ow7,4ob0,1qb1,2iku,5kh7,1eve,1k2v,3npc,2w68,6gqm,1b2h
,4dfl,4l19,3dri,5ab9,4qzs,3sqq,1d4l,4dn0,9hvp,2lko,1lev,3aau,4mib,3g2l,1le
e,1o5e,4os4,4b34,3m56,4qvw,4i4f,5wf6,3u7k,4j5d,1r4w,5iaw,3buw,1eou,3vbq,6g
5j,5t4u,3fuh,2pj4,2zn7,4n4v,2obo,1tjp,6b1w,1ru2,1fh9,4wcf,6hh5,5orz,4xmb,4
jfv,6ekq,5q0i,3h1z,4ycv,4ura,4prn,6ev0,1v3x,2pqz,2xjx,3k5i,5dtq,4pn1,5bue,
3ik1,2h5d,4jbl,2x97,2q72,2qpj,1acj,4k6t,6b1e,3f3e,2yog,4kio,3qem,3ued,5mge
,2bfq,3mxs,4k1b,4p6g,3td4,5mnh,5ien,3lk8,2zx6,1rql,3rm4,5u4c,4kb7,5ap1,4rj
8,5tkk,4j04,6i8b,2nxl,3zm4,4dy6,3h0z,4zyf,3e7o,5li3,4qjw,4aq4,3kpu,5oq7,3h
2c,2qnq,5jn9,5lvx,1r10,2l8r,5ega,2wi3,3rxk,1upf,6chm,4w9k,1vja,4zw7,5ov8,5
wag,1x8j,5a6b,6br2,4owv,5ev8,3drs,6fdc,3tvl,5mlo,2xaf,3h0j,4yas,1o35,5dex,
2x52,3lgs,3fkt,3dcr,2ax6,6cnj,4y5d,4umb,3l4y,6fkp,5aoj,1ony,6b95,5ueu,5ahj
,2xel,2g1y,6iiv,3b1t,5dht,4zup,4rlk,1axs,5fnj,1izi,4gvm,4xmr,5yhg,3g45,3ta
o,2q7o,1pme,4rrs,6gl3,4pct,2tsr,3qtw,4uua,5abe,3ozp,4lc7,2bu5,5kya,2q6h,1n
95,3k84,3bl1,3ui2,2cgv,4ez5,3t3c,3puk,6css,1ghw,3ezr,5il1,1a2c,5j6l,2gss,1
n1m,5f1x,5vt4,4y62,1bcd,1pwu,4fht,3w9k,3qu0,1zxc,2vtj,3dnt,2pu2,4hej,4x3e,
5ive,2wo8,4ozn,5i2f,6dj7,4a6w,4q1x,2g63,1jak,5knr,5y7z,5npb,2izx,3jpx,2x38
,4knr,3rwe,4aze,4nrm,6er3,6d59,5a2k,4cr9,5i29,4mbi,6ht1,2d1n,1uj5,3ljt,1ky
n,5osd,5whc,4qws,3uwo,3th9,2r2l,5n8v,2y54,4m5n,1yy6,6fui,3lvp,2gdo,3hyf,2z
va,3zmu,2pt9,5nxg,4zsm,5eif,4qsk,4djr,2ceo,4apo,5gj9,1q4x,4wz5,2vx9,2qzl,4
keq,2b17,4kzu,1bdr,4zjr,3ftz,3cic,2c2l,4yo8,1vj5,1w4p,1aaq,2q9n,2yay,4bea,
1szd,5acb,5db1,5ne1,1vj6,3n5h,3kpv,2hvc,3arr,3v0p,2fqw,5jf7,3udq,2n14,5mk3
,2pj6,4pp5,4c68,5q15,3u8h,4erw,1sb1,5iz8,6iiu,3txo,4nah,4ir6,4ufy,6fer,4zb
a,1jij,2piy,5td2,4yzc,5qap,5yhl,1h1p,5ni7,4pf3,4a6b,4uy1,2xwy,4el0,2w71,2p
8h,4d7b,4yoj,2ms4,4iwz,5ctb,3t85,4lpg,5bvw,3uri,3hfb,4mr3,3lq8,3mlb,4qwf,4
ixv,4njd,5tzo,5qa9,5ai8,3o23,4rn6,5i22,5u2j,4o72,4ej8,1rv1,4ixh,1w82,3arx,

3k02,3uf9,5wij,4oq5,1z4o,2qf6,3lq5,4yw2,3u4h,4f20,4mg5,5ufi,4n8d,6mu1,5zag
,4az2,4fbx,4emv,4bqt,3t2q,5ajw,4q81,2fqx,4kza,5k51,3u4i,5ho8,3lkz,4ciz,1yy
4,3bmy,3vf5,2w6p,6fa2,4flh,4w4s,3i28,3oyl,2nn1,5lrk,4e3i,4xg9,4d8n,3lik,4a
j2,4jt8,5j7w,5std,3h2a,2pwd,6bjo,3nik,5lxc,3qce,1vrt,4qxo,2y1w,1wok,5lne,5
am6,5er1,3ob1,1w4l,6e2m,5wh6,3kf4,4lnp,3el5,1w84,4gm8,3o6t,1q83,6fut,2ycm,
3cqw,5ot3,5dp8,1g53,5va9,4wx7,5kww,6bu1,1q6t,1uto,2bks,4fmq,5opr,3ies,6hti
,5u66,4qz6,6b1o,4gy5,5mrb,2xml,3skk,1c2t,4in9,4mss,4kju,1hti,5l6o,4wkb,4du
s,2am1,3t5i,1dkd,2onc,6hvh,1ypg,6cvf,6bkw,2jt5,3fdt,4wh7,5i3m,1azx,3fck,5u
6c,4ded,4k0u,5xmu,3wk9,4kz0,5a7c,6g9j,3iw6,4ddk,1icj,6eyt,4z2o,4e3h,4c70,2
y57,3dzt,5e0g,4nrb,4qr3,5ltn,3omg,6bod,4jpa,3fzs,6f5l,5edd,2oyl,2bmc,2xqq,
3kqy,1oiy,5y13,4d1s,3ked,5djp,3bxe,4bo0,3oy3,4o2a,5em6,5tfx,4d9p,3tku,4p1r
,5nwz,4bs4,3oys,5lgs,4nyi,3l58,2vxn,1y98,3ck7,1nh0,4an0,1mcz,2h9n,3h0s,2yc
q,2x4s,4acc,5ows,3rxa,4dhm,5lhi,5l8c,3rxi,3r00,5oq5,1jev,4ztn,4yb7,4a7j,4o
s7,3e3c,4acu,5hvt,3lf0,4jfj,4p44,5dxh,1d4k,5ewk,1gx8,1x6u,3g2z,1i7c,5opb,4
y85,6fii,6c7q,4jkt,4lbl,1w76,5dgz,3run,4qoc,456c,5twz,4a4h,6gl9,6h7z,4fxz,
2hu6,2ohp,4ayt,4fgx,1x8b,2y7x,4yb6,5i8c,2aoi,3kgt,3g2h,3gwx,5lyr,3nk8,1mrx
,1pf8,6dj1,4jss,3tu9,5cqt,1yds,3hy9,5y5u,6dko,3s0j,5hm0,3s3o,5om7,2j9n,4bf
d,5wzw,1d3v,4pvv,4i0s,4pmm,6ftp,4hct,1c5t,5i3v,5urk,5q0f,5xv7,4bj9,1css,4a
zc,1hqf,3kej,2p8n,5tg1,3cvk,6awp,1e3g,1t4e,2nns,5al5,3cr5,6equ,5fl5,2yex,5
lsh,4fgz,3mv0,3l5d,1q6m,5khh,5c85,2f3e,1npw,5w1e,4j0a,4mvn,5ioz,4yve,5tbm,
2fx7,4oex,4pjt,1nnu,3c4h,3vp1,1bky,5ajc,1jcx,8hvp,4wvs,2vcx,5hkh,2llo,2jkm
,1q6p,5mf6,5afj,4l50,1o3e,6evm,1pyw,3l3a,4mnx,4fll,6hx5,3ivc,6mx8,5wf5,5a1
f,4c4g,1w5w,2w26,4an9,5n8j,3aqt,4dho,4l7r,4aro,1stp,6g07,4hze,2cbj,5wbr,5a
fx,3bh3,4ca8,6aam,1r78,6aol,1gcz,3bjc,1o2x,1b40,4u45,5nat,4qiy,3v30,3kyq,3
unn,5elf,1xoe,2oqi,5mpz,4hxz,4rfr,3vp3,6upj,5xyy,4wci,2fwz,6buu,3ocz,3v3v,
2yq6,4m8e,4r6e,5ihh,5ngs,2v77,3nzs,5tyk,4l7g,3dne,1m5f,2xk8,4urx,3ft5,4br3
,1bgq,3fa3,2y07,4lpb,5k0m,4tlr,1ocq,4j5c,4awm,1ado,2n3k,4mf1,4oho,2xv1,5hh
6,5kmh,4lkt,3n2v,3o7u,2ieo,1wbo,5i25,4mhz,5mmp,3qs8,2ykj,5igm,4zji,3zxz,4b
71,1fpp,2y76,6eya,5lch,5gp7,3mmr,4qc1,3sbi,4bsq,5yhe,5hj9,4qw1,1px4,1q5l,4
i9z,1pbk,1o5b,1ryf,3oeu,3nx7,4izy,5lvf,3hll,4jbp,5icv,2yln,5om3,1e2l,2oyk,
2c0o,5w5v,1b2i,3w8o,4x60,6h2z,2a3i,5jnl,6m9d,4twp,5gmu,5wg4,2mkr,5e89,2j78
,3elc,3eb1,5wlv,3udk,4p0v,5ahu,5two,2jf4,1mm6,3c4c,5a2j,3ti4,3pd9,4rx8,4yh
z,2vr0,6mim,2k00,3fr2,5q0o,5dgw,3tiy,4jxs,5xst,5tpx,4j1h,5xva,1o49,2mlm,4a
lu,1tv6,4g2f,2xp4,1lhg,5aoi,2bz8,4kbc,1h22,5tho,1wun,1d8m,3m17,1slg,3pcc,1
oh4,2vio,3v6r,4n7h,3fv8,4f08,2p33,5a5d,3n1v,4j4o,5o1d,5ewm,3ds4,4b3b,1sqi,
2nmx,1gai,1fax,4deg,3suu,4rcd,6f6s,1juf,4zx3,1m48,5ttf,4man,5ljt,1y0l,6hsk
,1gyy,3bi6,3fue,1c87,4byj,4r18,6bnh,3qpn,4r4i,4e5h,4ojq,5aqt,3fu3,3oap,3d1
x,4j48,5uig,5vcz,5tzd,4hva,3mvl,6b1c,4e96,4d88,4ufi,4uvu,3met,5aeh,6dh5,4m
rh,5fqv,5t8q,6f7c,5l17,3zim,3k83,2p3a,4wsj,4cd1,2xk7,5w6u,4zs3,4u58,2qn1,6
f5m,2c8y,3vw6,1o42,5uor,2ycs,2i4z,4q1d,5m0s,3qup,1tr7,5xpn,2aa6,5uv2,3pgl,
4mk7,5uir,1p57,4ezx,3shj,5csw,3ibn,4ag8,6ceh,2p98,4yht,3k0k,5neb,4ysi,1bo5
,1a28,3wha,4nq6,1czc,1ckb,5j6d,3hr1,4acm,6hrq,1py5,3km4,4c66,1sr7,1rnm,3kr
x,3d25,5d6e,5w4v,1xbc,2x8e,4cmu,4f09,5afv,6bkx,3lqj,2hug,5dnu,2woa,5xvw,3f
i3,4yho,1h1s,1w3j,5y59,1sdv,5tmn,5myk,5f29,1efy,4ayy,4ykk,3m6p,1utl,3zls,4
ff8,5f6d,4k8s,2mpa,2gfs,1c86,5e5g,3zvy,2f3r,4e5j,3l5r,5l01,2vj8,4b9w,4i7m,
4qwu,3rpr,4i7k,2c1n,4ret,1owk,4mxc,2ohs,1akw,5tyh,5huw,5w14,5lb7,1f8e,4ciy
,5etm,2pwr,5o1f,4r1e,4ncg,4abf,1my4,4jib,4nxr,2jbo,1njd,1pdq,1qj7,5fi6,2vt
p,5dro,1o3b,4jln,2yis,1rt2,4feq,3gjs,5eef,2zx8,3ujd,1fkf,5eto,1c5p,5tys,6a
yo,4eh4,3djv,3mhm,2pow,1w0y,4dbm,2ywp,5uhi,1mj7,5am3,1pxp,4uff,2bq6,1n1g,5
nxq,966c,3vws,2of2,2ydf,4nbk,1l0a,4u43,5trh,1rpa,5etv,1h61,2vcb,3d1v,1xq0,
1b3h,3svj,5fun,4bw1,1kti,4jof,4izm,4phu,3jy9,4urw,5ael,4irx,3avn,5mm9,5twl
,4w9l,2ad5,4b4g,4a22,5vfc,3l3q,6fn9,4m14,2gl0,4c35,3iob,6apr,6g3v,4cik,2xp
b,3loo,3drr,3e4a,5thi,5mgm,3mrv,2c4v,4i8n,5obr,2qyl,6ee4,2wzx,5j8z,2itz,5t
2i,4mdn,5nw0,5l9o,1c5n,3bel,1nt1,4g69,5ovv,1i9n,1hlk,5hh4,2hhn,5v13,2ww0,2

rcx,5zms,2ncz,1a86,4uuq,5g2n,1o5g,2gfj,5d3j,3dpe,5ldp,3veu,2vaq,4ui5,1hp5,5g1a,3f39,4owm,1dl7,3ce3,2qmg,4poh,5azg,6by8,5l7f,4awo,6fh7,4ifi,6b7e,5i3x,5lws,5sve,3kn0,3q2h,5eoc,6b7h,5ekn,3rqw,4erk,5fso,5e80,2bpx,1yw8,3sc1,5o4t,5c2o,4u0f,2w17,5nxy,1oy7,6dvm,3k00,1py2,4zx0,4nkt,3s1h,2ym5,6cis,3hdm,2yj2,4ef4,3kvw,5cu2,6c1s,4j6i,6bij,5xgh,5u2c,3m3e,1yid,1b6k,4gd6,5lp1,4ww6,1zhy,3eky,2q38,4jym,2ymt,3gur,3suv,1njs,4na8,4msa,5nk8,1o4k,3l6h,1i5h,3t6b,5e1s,6gzd,2eum,1z71,2yc5,6hai,2a14,4aa4,4ask,4ea1,3pxz,4n4s,5wkm,4jg0,3sie,3pkc,1w1g,5y8c,2c6m,5ou3,6d50,5v3y,5lzj,5tya,4c9x,3eyu,4xtm,3agl,2brh,4mbf,5d4a,2zz6,2gbi,1t5f,5jiy,1nw7,3hl7,6h7f,1zd4,3suw,5fdc,3cct,4pm0,2y1x,5n9n,4gj2,5d6f,4joj,5klz,4jju,2gg7,5hn9,4tjy,4r91,6ffn,5usf,2clk,5n70,2ohu,3eka,5eq1,4qlq,6eis,4acf,5t35,4orx,2hmw,3fz1,3qx5,4zhm,3rxo,1atl,5t1k,3np7,3zt4,1bdl,4u6y,2jbk,5l13,5mob,2igy,5jlz,4flj,2zu4,2w70,5d6y,4kip,2uw0,4msg,6f8x,1r2b,4qvm,2ydo,1w4q,5okt,5op4,3al3,2rvn,3dp1,4luz,3o87,1w2h,1fiv,1uh1,6c8p,4g8m,4hai,2evc,2q2z,4yyt,4od0,5k9w,3fyj,5vh0,5ale,3th8,4yhp,4pvo,4whs,1c4v,5hed,5qbv,2oei,4y2s,1z6q,4px6,2knh,5kns,1m4h,2g96,3cm7,4gcj,1qft,3ztd,4yzu,1vwl,4kiq,6ax1,3zyh,2c1q,6mx3,5o9r,1q8t,4ue1,2y7z,3mdz,4gtr,6fh6,4qvq,3tti,5ap6,4pkt,3tdj,3qnd,4lvt,1xo2,1pcg,5fiv,1g3e,6gx3,5dhj,5ixt,1xhm,4po7,1a5g,1haa,6egs,3iw8,5qag,5o1b,5brz,3mpt,3ral,1j14,5wdj,4io3,2irz,3f35,5jan,6mrg,2oi3,1onp,4bjx,2i0v,4hmk,5mat,4h2m,4agl,4ayx,5iwg,2xez,5em7,5kdf,3vv6,4y59,3ti6,5gn6,4u7v,3rz7,5wxo,1o2w,4r0i,1a30,5nz2,3rf4,6ee6,4oiv,1a9u,3iop,5x4p,3gl6,1hqg,2oji,4ps3,2w05,1tnh,3djq,2c9b,2flb,5itd,5k00,1t37,3g0b,6hdo,2rfh,1b5g,4bdi,4ikn,3inf,1rww,4g68,4djw,5yum,4hxl,3dbd,4hw7,5mys,3p7i,2j7h,2vvt,3r2y,5ng9,1y2g,2vwm,3if7,2hl4,3ffp,4qwg,3g6z,5dhf,4y79,3m57,4oyi,3s0b,1yvx,3v7c,4md6,4g9c,2w6m,5tt7,3pkb,3upi,2va6,2weh,4bao,5gid,2w1g,3mxc,2fw6,3diw,1ind,3i3d,5k0i,4npv,2wf5,2q7q,4wyz,5xaf,1gj4,5hf1,2aw1,3dbu,3o9i,4l34,5j0d,5afl,2w3i,5ew3,5e0m,5jfu,4fm7,1o4h,4w9p,6eyb,4onf,4zhl,3mj2,3k22,2xxy,5m5q,4aci,5kab,4dpt,4rsk,1rsi,5c42,3tb6,6bmi,4myd,2i40,5nhj,5moc,1tom,3ds6,1lt6,3eft,2w8g,4d1y,4yrc,1tu6,5hmh,5nw2,1k9r,3mxf,2q1j,1u0h,4ye3,4k60,5zop,6htg,2fx9,4q83,4y8y,4gr0,3uqf,3vha,2yaz,1k1m,4i2w,5ovx,3db6,1v2p,5njx,1wtg,3uvn,3h89,2q7y,4o2f,3fv1,4eu3,3pwm,4tmp,5ulp,6di1,4iks,4dhs,5hg8,6b1x,5nw8,3tjd,5yve,4uwg,1jd5,5km5,2w4x,3umx,2ony,1bm2,4pku,2pj9,2m41,4z8a,2yjq,6mwe,1prl,5y1y,1c5y,5ak6,1ggn,2usn,2pv2,1w25,1c4u,4ba3,6c2x,4ijl,3gxy,3f3u,4io2,4drm,3cjo,4dst,5c87,5ni8,2ll7,2pem,2x7d,3zc6,1m2q,4gk2,3lqi,3c1x,2az9,1fv0,1gz3,1gfy,5mte,2nxm,4yml,4yog,2g6q,1t79,3hdk,3uij,5vl2,4ufk,4aph,6d1u,4rn2,1zxv,2p4i,1w80,3bl7,2vh0,3kqa,5vdu,4b2d,5jgi,4u0i,3g9n,5var,4lnf,3e9h,2fzz,3cfv,1ws4,5h21,2p7z,4j8s,5fdr,5z7b,6cj1,5tts,6bmx,1g49,1mqd,5ost,2qhd,6e4a,3fur,3v1r,4wwn,4hni,6e83,1gqs,3eko,1dmp,2wnj,4i6q,4d8s,2jb5,4p58,4usj,6eq6,3o0j,5apj,4ty1,5avf,2xef,1way,1a7c,5f2r,5k1f,2c9d,3a9i,3deh,3q3k,4xtw,6f0y,2r3l,4mra,5qa4,4zpg,4ijp,3wyx,1s5z,1y2h,3qi1,1c83,5dts,5e1d,5j9k,5o4f,2wxi,5f04,2qmj,3kvx,4l7j,1d5r,2vyt,4cfv,1qcp,4hlm,5d48,3snl,5uwn,4yll,2brm,4dzw,4q7v,5teh,1c4y,5mro,4ymg,3b1u,2z9g,2rt5,3gxz,6e0q,6f7b,4qz5,5dh5,3v3q,4do5,3k0h,5dt2,3l54,3ebh,2hd6,5hbe,4wri,2qi7,1jao,5ajp,6ej3,3gjq,6f8w,5y20,1rwx,4ibj,5op2,3c6u,2w0s,1c7e,5yyb,3ni5,3t8w,5ntp,4dfu,1o4e,5tqu,2xyd,4pl0,5tl9,2vpo,4ov5,4qps,5wdc,4awp,4a9s,3atk,4nja,4ms0,3fzr,1i9m,4b5s,2y71,3f33,5q0h,5x9p,4p4j,5ajo,3c56,4pnl,4fmu,3uli,5nlk,4cs9,6hkz,2vo5,2fts,3sfc,3pke,4dk5,1lor,2jdn,3jya,6bqg,3p9t,3qkk,6fnj,6fmc,5c0k,1mmq,4fck,5npe,4j59,4eol,5m57,4efk,3bt9,1oiq,5dwr,3wyk,4qf9,4c5w,5tuq,3oqk,4xj0,5mwg,2vwu,6dpy,4ara,3wi6,5owf,3nw5,4fhh,3sna,1uye,2vcg,5j7j,4gtt,5jc1,2ph6,4zpe,2hz0,5l4i,1y1m,5vo1,4y2v,1ecq,6eeo,5jrs,4l23,6f34,4r4q,4quo,6dq6,4e3k,3zov,1cet,5e2k,5jyy,2jld,5jna,4qls,4ksq,4py4,6biy,2hqu,4yk5,4i12,4km0,5ttu,2za3,4o5g,2y1g,4cff,4ztr,4jps,4uin,2q93,2x7t,2q6c,6ayr,1pwy,5lwe,5cxi,4n9b,1fzo,5ukm,1ork,4mk0,1jbd,1k2i,4ifh,4yh4,3fee,1flm,5tqe,1jj9,4li5,4jc1,5i8p,1lah,1apw,3ppm,4kn7,1m5w,3vbx,4ie2,3sur,1nz7,4trw,4qmu,4qnu,4os6,2bge,5xvg,4o6w,1nfu,2zjf,1fpi,5id1,3ccz,2d3u,5tyj,1fls,1ggd,5ofx,4o

12,4wf4,3pck,4ayv,5jt9,5nb7,4b84,5ggk,6h5x,1r5w,4zw6,5d6j,5ep7,4duh,4ps0,4
drk,1los,3s6t,3be9,4ipi,3wf7,4pow,4y46,5izj,1d4j,4qg7,2avm,1g85,1r1i,3g1v,
3l7c,5wyr,4oti,4a4o,1fvv,3uok,5n3v,4tim,3tv8,4a4g,6c0s,4fak,1w12,4jn2,4oyp
,4zt7,5emj,2rc8,2ypo,3kmc,4hwp,3q2j,4k5l,3hwn,5i83,1j4r,4fyh,1kfy,5g43,4bn
v,5fpu,1o48,6ei4,6ekd,2k3w,3ksq,5wkl,4hzz,2f8g,2uw3,6mi6,4u91,3f7u,3zk6,5n
2f,2qx0,3mrt,2f0z,3qvv,1l7x,2oc1,3gbe,5he3,1pun,5m4i,3pp7,5o91,4mjo,4fns,4
xnv,3tay,3ejs,4c1w,3njq,6guc,4dv8,4el9,1iep,1ta2,3h0e,1bsk,4pri,3pcb,4jg8,
3lbj,1i8h,4a4x,1lhd,2wa3,5tcy,3wth,4baq,1ylv,5bve,4g34,5u28,1qbv,5z5f,4ot6
,2g97,5n16,1m5e,3efk,4h7q,2v5a,5vs6,2cne,1pxn,1jqy,5j6s,4abv,6d6t,1t4s,3zs
x,1p01,2f4j,2gh7,3f78,5i38,3h0w,5d3x,3zst,2lbm,6fil,2vk2,6ew7,2iu0,3t0l,5a
i9,5wbm,4ark,3hlo,1np0,2w6c,6dk1,5xih,5hk2,1qhc,3v5l,1vr1,4no8,5hmy,2jql,1
a46,3kf7,4lv4,5hbh,3wf9,4ieh,2vr3,5dyw,4xuz,1mmr,5ar0,4h4b,5knj,5elv,2g79,
4w52,2rnx,4rgd,2az8,3c1k,5ick,4lv3,4mwb,5a83,1prm,4gg5,1rlq,1rq2,5fbn,4e49
,1f47,4yfi,2v10,3pa3,4j1f,4jvi,4gmy,2v3d,4cft,1epp,2hoc,4r5g,4k0y,5zz2,1d3
p,4ng9,6fav,5hor,3qci,4mbp,4msu,5hg1,1pyn,1hmt,2lpr,4yha,5hoa,1sln,4h3c,4k
64,2w0d,4lmu,3tf6,4z2p,2hny,5ctc,6cck,5vd1,3qcf,5ia0,3l7g,1eld,1xh9,1h3c,4
urm,4j7i,4ly9,1nu1,5sz5,6cdl,1uvu,2wl4,4eo4,5lj2,2qrl,3kc1,5nqe,6gon,2xs8,
5gmj,5eyd,6c42,4wks,3ka2,4p6e,4nnn,3zvw,4q6r,5v8v,2jdv,4lxm,5qcm,5e8f,5y80
,3ud5,4n70,1jwt,4cgi,2uw8,4ajw,4ivb,4m6u,2xu1,6bxy,4ud7,3k5f,2arm,5jin,3fm
q,2nt7,1p4r,3hxf,4zwx,4xx3,4yee,6aak,2xae,5t31,3bhb,5llh,5nge,4b9h,5oxl,5i
pa,1q3w,6d56,6df1,3ole,2v16,3g2s,4led,2uz6,4pnu,3nrz,1f7b,1j4k,5ku9,1a4g,4
uia,5kv8,2rke,4e8z,5ykp,4z1n,5sz1,4oak,3twu,2r3p,5cf6,1ym4,5l4j,4exs,4qpa,
2k1q,4eny,4i0r,2xz5,5fxq,3re4,6cf5,2cbr,5ece,5t2t,1x8r,4n3w,1klu,5nxx,5jcb
,5klt,4usw,5ek9,2mov,6bid,1mjj,1tlo,2hb9,2f94,4luv,5gu4,3wf8,5ufo,4n5t,5hc
x,5mtw,1o38,5z1c,4w4z,4v27,1k21,3jrx,5l96,1esz,2m3m,5ivf,1o3p,6std,4auj,2l
yb,2w87,5j3l,2fgu,3snd,2w5g,2wsx,4qfl,2vuk,4a23,5iu6,1siv,4ht2,2ltz,1o39,4
p0w,3p8p,5ypy,3zvt,2c5y,1ayv,3erk,6g4y,4ucd,3e16,4i7p,5alv,1osg,6gw1,5wxg,
4bvb,1nc1,5ugg,5t1w,3tvw,5hkb,2r3n,2iog,1h1r,5eyc,1ogx,3er3,5q0p,3rlq,5itp
,4nuc,4w9j,2b8l,5khj,4mz4,3zlv,3okh,2epn,1pwq,3gsm,3hzv,6dpx,4hvg,1s17,6ar
m,5jq7,2v88,5lay,5nn4,4hj2,3pp0,1rst,3qcq,3rx8,5ewz,5mgx,4c1t,3cso,4wq2,4g
xl,5isz,2xj2,4y5h,3zcw,1l8g,5vex,4tky,4elh,4ejn,4pgh,2wuf,2xbp,6gw4,3d7g,5
ksu,2qrp,4jg6,4eqf,3hbo,5yjy,2gv7,4hdf,1yi3,5edr,5nmf,4w7p,4fz6,1g4j,4a2a,
5kx8,5a0b,4qta,4mho,4fn5,1gj8,3d94,1kdw,6euw,6gpb,6glb,2xaq,3vh9,1aku,5j4v
,2f1g,2vfk,2r5a,1g1e,3buf,1bzj,4abg,3cho,5t6j,4jda,5ywy,6cdo,6fo9,1g74,3ox
i,5t8p,3pcu,1fyr,1fm9,5ddc,5hwu,1xud,5ai1,4e9u,4uyn,5k05,6fgl,5iha,4aa2,1w
zy,2w85,3s2v,4yuy,6dud,1xdg,3bgs,4hiq,3rqf,1jmg,2xeg,4g93,6c7b,3zso,4xjt,4
rsc,5q0u,5iyv,1qxy,5n20,1you,5c7e,4x9v,5tjx,3rtm,4b05,3tg5,1b52,1w1v,5lg3,
2bmg,5wii,5wew,5iua,2g00,1nm6,4wef,1i7m,4hrc,6at0,1biw,4uhg,5hvp,1ugy,3ery
,5ll4,5v6y,1ru1,2o4l,2evo,5lvn,2rc9,2fci,6evq,3rah,3g2v,5ny1,1p10,3o96,5vs
d,4cxx,5kcb,4g2l,5vqi,5svl,5qax,5h6v,4aa0,6g1w,3ddu,1pzi,3sax,4nmr,4c1f,5d
26,1elr,3gc7,3c84,2y1d,3vd4,2uzo,3v8s,5mqv,4zly,4p0a,5dlv,3qqa,4g3g,3h9f,4
wr8,5win,4hzm,3rqe,4j1c,4kii,5mw6,5mwj,1xap,4zv2,1ol1,1a69,4btt,5t4h,2oi2,
6bbs,6aaj,2y4a,5i80,1hte,6cfd,1o4l,5kbe,3saz,3kqr,2b07,3iue,3bxf,1is0,5a4t
,5ktu,4agd,3nf8,2nd0,5yyz,2ay4,6fhq,3f3t,1zhl,3vbv,1g9s,5aut,4u71,1q66,5vb
9,3qtr,4nw2,3qin,5mzg,1d9i,5h08,3rv7,3rv8,6ckx,2itk,4ij1,1pxo,5nt0,5x62,4o
c4,5qao,3le9,5dtr,4a6s,3bls,1em6,1pus,3asx,4lng,3mvj,4q3q,3s9t,4gfd,2r8q,5
wqc,3ri1,5ih8,6g6w,3cjf,6duh,3shv,4pda,3cpb,1bp0,4lmn,1zm6,2kup,3ij0,6cz3,
3v3b,3vru,3arg,2o64,6ayi,4qab,4xu2,5wzu,4lh2,1w6y,5eis,1czr,5jzy,4ejf,4ufe
,5wqd,5f0f,4aw5,3iqg,4p90,2ank,4n1u,6eku,2h23,1j5i,2y7w,5alj,5jfr,5eak,2gf
k,3be2,5hln,5uc1,3ax5,4f3k,5qb0,4llj,5ldk,4eyr,5v3x,4fys,4crl,2zxd,2etk,3d
dp,4q0a,3el0,2ica,4yv8,4jin,5enc,1pb9,5jur,5n1z,5ou2,4y8d,4gby,5acw,4r76,5
hrv,2jt6,4hhz,3ocb,4qxj,5er5,3hu2,1v2l,3dz5,4j8b,5vqs,5khd,5fho,2f71,3kdt,
5gmm,3sd5,5fck,3q8h,4h36,6bm6,4qmz,4ks5,4dew,1nf8,5ap0,3s73,6chp,4r3b,2oag
,2w06,5j74,2a2g,4ju7,3i81,5aol,1af2,5hda,3nu3,6das,1swp,5njz,1b74,1sts,2co

0,5od1,3pab,5u06,2gnh,3uig,2uw5,2vrx,1ele,3hp9,3fqa,4mh7,2xkc,2w3l,5w73,5h
ip,4xv2,1fch,2j4k,1o33,2v57,1kqb,1bhx,4a4w,5bmm,3dcw,4i9o,4ydn,5xii,5duf,5
j1r,4no1,1gvu,1ycm,2l3r,4wnp,4yzn,4p75,1b1h,5hwv,1s4d,3rlb,1c5f,4fqo,1iau,
5mim,5y93,5qa6,3wk5,4mcb,4qms,6b5m,6fba,2q8z,3r92,5mo0,3wvm,4qh8,6dgz,2w66
,2joa,6gih,5fqt,2viw,4o76,2j83,3cm2,3ion,4y2u,3fhb,2rip,2q94,6eiz,3ieo,1uy
g,1vsn,5fnf,2vgp,2pk5,2f6t,1zfq,5ypo,2nnv,4jvj,2cej,3hqw,2boj,2h21,3bzi,5u
t6,2toh,5urm,5ml8,2qpu,6f5u,3t3y,2zx5,5alu,2vxa,1z4n,1c8k,4w53,5cf8,4hcz,1
jik,4b81,5y86,5ul6,5l2n,3dhk,5vio,2piz,3jyj,5ly2,6do4,1v2s,4ezo,1iht,3hl8,
4q1c,3sk2,2xjj,6cdj,4w4w,1bm7,5lca,3rxf,4q7w,5ceq,5aen,3kjf,2psj,4avt,5nxo
,3qn7,1d6w,5ory,3ckr,6eyz,4lxa,2chz,6eqw,3nzc,5umw,1ilh,1o4j,2pks,6evp,2wk
s,2yc0,4xy8,2jg0,1u33,5iez,2zxb,2qnz,5wfz,3ob0,1g36,4at3,3pd3,5jjr,6eo8,4o
mc,1k3t,2vl8,4y8x,3l4t,3s2a,6f4x,2qrm,4u68,4o3f,4rpn,2zns,4zvi,5ul1,3fwv,1
exw,2vvv,2aof,5vfd,2qcf,3bl0,3gk1,5i2i,5w8v,4ptc,3piy,1nlj,4ddy,2vwo,3kdu,
6bt0,5l99,3rtn,2yk1,6f28,1zh7,5uiu,4eh7,5epp,1o6h,1b05,5t5g,6b1k,4k75,5hva
,3ips,5obg,5a3o,2q89,5owc,5ggo,4xk9,3cf9,1czl,2ewy,3a1e,1xlz,5k0b,2nta,4gv
c,1axr,5fou,1kkq,4u7o,5i4o,4cwr,5fa7,1i5d,5hdu,2zg3,2pmk,3mrx,3s1g,6mvu,1w
km,6dvl,5fe9,5odx,3sbh,2jiw,2r43,4ibb,4o05,2v13,4io8,4z2h,2vmc,5ggn,2afx,1
g3c,2cn0,3oki,4xty,1xgi,3mmf,5v1d,2uxz,4wwo,4kpz,4xbb,5up0,2gzl,4r5x,3iet,
1oq5,6afd,1b55,6chl,1xp0,2q5k,5ti7,5uqx,6bm5,5w94,1k1o,4lm3,1ek1,4ayr,2xxn
,5lsc,1o0m,4ui7,1ppl,6beh,3ud7,4zxx,6gzh,1uu9,5jgq,2q95,5l2y,3v3l,2gmv,5bv
o,3snb,5q13,5upf,5tq3,3gw5,4wwp,5wbo,2j7g,3rmf,1fhd,2w2u,4flk,2r0y,2wbb,3d
a6,3l4z,1v2w,2azb,2bz5,2oxn,3f8w,1w7g,3moh,5m3a,5aqq,3tz0,3zyu,2pzy,5usy,4
mx0,4cpq,4aq6,5eld,5gmv,3qbn,6rsa,3u5j,5h1t,1ms0,1ctr,5lub,4h5d,4n99,4cc2,
4hzw,5lbq,3ati,3pd2,1f74,1qyg,5dhg,4mwx,5ohy,4ob2,2b2v,3c0z,2gtv,4r75,5w2s
,3wsy,1rs2,2r6w,4agn,4gk4,4qq5,6bfa,1n7t,1ocn,1gnj,4u79,3hmm,2vl1,1ec1,5f4
u,2zsc,4u5j,4jfk,5kew,4btw,2cbu,1vkj,4jnc,1nli,6bw3,3fyk,3cl2,6f3i,1w3l,3g
v9,4h4e,2ylc,2fgi,2ha5,5sz9,5gjg,2q1q,4aza,5yia,2rny,1o30,3m1k,6dj5,1pfu,4
awf,5oqu,4k42,2wcg,3oj8,4ngs,4lq9,1jzs,6g9h,6g9u,3axm,4qp7,4mma,1g98,5wi0,
5iep,2opy,6eq1,2pri,4y2j,4x1s,1bux,4cjn,2vnn,5d2a,6cms,3g72,4wz4,2las,4his
,5ohi,1apv,2v86,5nap,3wdc,3d9m,4kzl,5ixf,1fd7,5f25,5js3,3d7h,4ceb,3dst,4yy
m,4x7n,4cr5,1gar,4z2b,3iph,5t37,3zqe,6m9t,3m54,3szm,2oxx,4mlx,3uxg,3qri,4l
0v,1a07,5qay,4cwo,3r0y,1wbs,8gpb,6bgz,1ecv,1ha2,2kfx,2be2,6cjw,2p94,5tq7,5
fsc,4btb,5yov,6eox,5am2,2pvm,3gn7,2ql7,5j58,1msm,2bjm,3rt8,3ds3,1m13,4nxo,
2vj9,3fqe,2m0v,3so9,4r5b,1v2j,4bks,4tkj,1jld,2ojf,1z5m,3m1s,4lpf,4zls,4af3
,5eue,2jh6,2kvm,5etl,5hn0,3f6g,3fdm,2jj3,2zjw,3sfh,1ong,1noi,3cen,2p4j,4rv
r,4az5,2wqp,6fnt,3ejt,5c2h,3sow,3ml2,1ld7,1yz3,3m2u,2wcx,1ec2,3iqj,3ccn,3r
1v,5foo,3cn0,2z4w,4ks1,3rtf,4bfr,2pvk,3ppr,3r93,4x9w,2z7i,4hdb,4bti,1eef,6
bln,4wyy,4bw3,3mks,3iit,2axi,4urz,3q2a,2w12,3aho,6e06,5dy7,1gi1,3az8,4ew2,
3tgs,3zc5,2ggx,4ih3,5u00,4qkx,4oef,6gjn,1ewj,2xii,5al2,1o4p,2xck,3dcs,2g0h
,5adr,1kv1,6h33,2wl5,6h3k,1qq9,5vdr,5x4q,2xk3,3p4f,5prc,4gxs,1qm5,4aw8,2ee
p,5l15,3tk2,5heb,5aia,4nh9,5nki,3eos,4m8y,5u49,5yh8,5ylu,6f8r,3nxq,2x3t,5e
xl,4x0z,4rj6,5ly1,3zo4,2nw4,3upx,5btv,4n3r,5ek0,3otx,4r4c,1y3n,5cal,4dld,5
z89,5sys,1ybo,2cmo,1k3q,2ima,3wv2,3caj,3vv7,1xug,4l2k,3aya,3gs6,5alg,4b4n,
3zyr,4hxm,2ov4,3n9s,4n07,2jnp,3zxr,3v7x,1aid,2zis,4u8w,6e2o,5yqn,3oc0,4b1d
,3g9e,4qw7,1c1r,1zsf,2nm1,3cs7,2x24,5d7c,1bji,4yl3,4k78,2f9u,4kab,3rl7,4n8
r,5etu,5drs,6es0,2g8n,5ol3,2i6a,5gvk,2qrk,3sx4,4q4r,5nq5,3ctt,5kxc,1tyr,4j
0z,5z4h,2uzn,4oon,2ohr,4re4,3prs,1f8c,4jvb,3eig,1dbk,4p74,4zg9,3wtn,3l7a,4
cdr,4fli,2am9,2jkp,2bts,1u9q,5ea6,6db3,4b6o,3nnw,3p5k,2c4f,1rbp,2xuc,4hvd,
3ijz,5n4t,4jr5,6e9w,5f63,4cjq,1b46,4j4n,4kcx,2a2x,4h3i,4k4f,6bky,2bak,5csd
,4mgc,4mwv,1mzs,4ym2,4ztm,5epn,5v7w,5hes,3n49,2zlg,2kbr,5fng,2fgv,1f4e,2gd
8,3g6h,5u0f,4j44,5v35,1c9d,6g6y,4azi,5kpk,4ui8,1no9,4p3h,3gcs,5ngu,6bl1,1q
6j,2zda,3b27,1o2j,3u2k,6mil,1n7i,2wec,1h6h,3owl,3g34,1ec9,4crf,3hha,3qiy,4
dh6,4pid,2y55,5hhx,5etf,4xar,2pvu,5ito,1li3,3dya,1xsc,3ti3,1tze,6fmf,1di9,
2yke,5f4p,5sz7,1m2p,1uu8,4y64,3rt6,3vs3,3nfl,4bdg,5jv0,3wd1,4pge,5cxz,5ak4

,4mdt,2j27,4lte,2ayr,5m6f,5dv4,2qbu,6ciy,3ekq,3c7q,2osc,4r6w,2ph9,3qkm,3hh
u,4xqb,5jr6,2uwl,5ezz,1y3y,1i8i,3sxf,2i7c,4m1j,6rnt,3g5y,3hwx,2p83,1mik,1m
rn,1sm3,3py1,5fah,4pnn,3tza,3zmp,2wxl,5qaz,4odf,1eix,5b2d,5kdr,3hcm,2x91,3
frg,6gch,4qz2,1hvh,1g3m,1tnl,3s7f,6cpa,6g0w,6ffh,3kec,1nj5,6afr,5kup,6ftn,
4o97,2i5f,5nzf,2f89,5fsn,3nex,6b2c,4zt5,3gt9,3sud,5btx,4fab,3fxb,2x4z,3l4x
,5svx,5u98,1oim,3lpr,4pqa,4pnk,4ogj,5k0s,4oba,6h7m,3aas,4zae,5u5t,4n6h,4ds
y,1ywh,4w9o,2qbp,5d45,5lhu,2bro,1uz4,1ugx,6avi,2aox,5nb6,4he9,2oz5,5n24,1h
8l,3pwd,1it6,4rvk,6b27,2qu5,4x7j,5tyo,2ym3,5mjn,3h26,5ufs,2wj1,1rt1,1kz8,2
zbk,4krs,5j87,3u3u,3g1d,5bms,1u9w,2z7g,4xv1,4ngm,4j8t,5d11,5c11,3f17,4alw,
3q3b,3rwd,1c70,3iiw,1hww,5kv9,4wey,4ok3,2ctc,4bcb,6e8m,3shy,4mnw,5ut0,3lcd
,5yc3,2vwz,2xbx,1o0o,2vj1,6b33,4hnp,1w70,4bh3,4gng,2q8m,4rrr,4rqv,1hih,3u1
i,5ovr,4ach,6c0u,4qiz,4k18,1e72,4ocq,1b11,3gds,3mwu,5ivs,3bux,3qc4,5ovc,1p
u7,1y3w,4v01,6e7j,1uu3,5dh3,3tu1,4q3s,4r5a,4z16,3uyr,5wir,4exg,5kby,4iur,4
zv1,5xsu,4gtp,2o8h,3wqw,5th7,5tv3,1tfz,2v3e,5ntw,3cyy,1ny2,4q6f,2pjt,5w7x,
5jap,3b68,5tyl,2a4w,2v00,4nks,1ets,2fq6,5hbs,4r59,2ama,2web,6e4t,4xm6,3bkl
,6fty,5v0n,6ge7,2boh,1ikt,2r5d,5alh,5szc,1cr6,4zzy,2p1c,1phw,5w92,3l9n,3k8
q,3eml,3nij,5amg,3bv2,2w73,1h35,3tne,3jyr,5l9g,2xsb,4f70,2iuz,1ml1,3ewj,4m
k9,2lo6,2jiu,4es0,6dqa,3kab,5mxk,2i0y,4zy2,6cqt,2i47,2i0d,2i80,1nfw,5uzk,6
hd6,1y6a,1vcu,1e2k,4wz6,1nc6,4zyv,1wcc,4og4,3il6,1kzk,4frk,1nms,5kax,1fwu,
5djr,3a1c,1hdq,5ml6,5l4h,4rj3,2x4t,5nk9,2xj1,3mfw,3gy3,5nvv,4rh5,4jxv,4zs2
,5ivv,4axa,5lli,3q5h,4wmu,2z50,4lkf,1rjk,6g93,4nni,6exs,1nki,3i7g,6ei5,6er
v,1z3c,3r5n,1lol,3qk5,1rhm,4jfe,1xm4,4gny,3ti1,4hhy,5b5b,4fjz,3w2o,1oxq,3w
k8,6bl2,3oe8,3f7z,4j3i,5ogl,4my6,6fob,4o1l,2uy0,5dqc,1g46,5orl,1uwu,185l,4
z07,2w8w,3l3l,3vvy,3jwq,1o2z,5lsy,3iok,3g6m,1sdu,4xtp,4qt2,5fdd,5ia2,1ux7,
5jhk,4ca4,3qfz,1qvt,3t9t,4j0y,4x49,3lpf,2bpv,3jzr,1fcz,3qps,4p7m,4hvb,3cp9
,5jm4,2q9y,3eid,4o4y,3cid,3nc4,6b1f,3rth,3rxe,1sl3,4ap0,4mu7,1pvn,4jfm,1re
j,3ens,3e9b,4hby,6guf,3iiy,2z4y,4to8,5t1i,5upj,3c89,4dju,3pcg,6gu2,2euk,3v
bw,3px8,3g4k,6dh0,1ukh,4rxh,2ien,2upj,5m44,3gzn,3eio,2hk5,3emh,1o3f,5ipj,6
dai,3su5,3sh1,4iic,5e0h,4fs4,4m3q,4wf2,2osf,2pj3,2r0z,1jrs,1i9q,2zgx,2jfh,
2ow9,2aa9,5vt1,5l0c,4ybk,3zmt,5kr1,2r7b,2lh8,2wyn,3ckz,6lpr,1usn,3v2n,5oq8
,6bhd,2pym,4xaq,3puj,4ek9,1eb1,5o1a,5k5c,2rnw,5qa8,2zg1,4gtm,3uec,4ncm,4al
g,5hvs,1lxh,1y6r,5aux,2qwb,2h2d,4qir,1s89,1qwf,2avv,5knt,4odn,3o1e,3lj7,3e
ov,6cpw,1qca,1jii,3lkh,3rul,4g4p,1qk4,5nkh,1w0z,1jlq,4ngt,4meo,4pmt,2v2h,4
lm2,3bu6,1bzs,2vvc,1v0n,5a7y,3d67,4u0c,4uxj,4b78,4bo2,2jsd,4aa1,3ga5,5hka,
3vtd,2gm1,4x6x,6ma2,2ces,4ayq,1tpw,2uxi,3gc4,5ye7,2cbv,1v0p,2uzb,5xvq,3r7b
,3umo,3b8q,2y6c,3bc5,2bes,5wa7,4f6x,3uvk,1hgt,6m8y,5twg,4avu,5vwi,1ms6,5xo
7,3dcq,5km9,2ygf,1zsr,1f40,5m2q,1bl7,4k7n,4hvs,4z2i,5voj,3ck8,5d6q,3g0e,5y
0f,3dpk,1bjv,6bw5,3dow,2zif,1mzc,2nng,2xnm,3f8e,2ndg,4lh5,3e8r,2e9o,3uih,4
hlc,5moo,4epy,2i3h,6fby,6ew6,3ama,4l0t,1vj9,3pup,2xct,5fh8,5ivz,3pxq,1str,
2xxx,2pqj,5mra,4o28,1d6n,1pr5,1rpf,4tt2,3bhx,2cma,3dct,4qt0,6f1j,3gvu,5yy9
,2x81,2cv3,6fa4,5jo0,3l0e,3p3j,1eed,5aib,3qbc,1n7j,5fpi,6bnl,5v8q,6gxw,1rw
8,5zw6,4e3d,2mji,6ayh,3lcv,1w2k,3zns,5mty,6c3u,4ks4,3ds9,4hld,3elj,4j45,5v
41,2yne,5nad,5lvq,3h0a,3d9p,4tu4,3ip5,1w22,4kbk,5lqq,5wyx,3ps1,5ou1,5j9x,2
xru,3t4p,3uzp,2o2u,3dei,3u93,4z8m,3lhs,2f81,3fn0,4iqt,5laz,1xot,2z52,1zs0,
1oau,5d47,3o4k,6dcg,1o4q,1csi,3tpp,5nwd,2wca,4ie5,1u1w,2w92,3r4n,5aae,4gja
,2pj2,5f2p,5hjq,2yme,3cbs,5u0y,5w3i,5qaw,5d3h,3sb0,5alx,5g57,3uhm,6afj,5jf
5,4ccd,5mhq,5etx,1clu,4k6u,6g28,4zyz,1sj0,4eh3,2r5b,4pyn,5eq0,1cil,5le1,5n
yh,6bdy,2auz,5vm0,2ff1,3f8f,2rka,3dda,4iu4,5bs4,5a0a,2qn2,4fyo,1p2a,2uze,1
x38,4ps8,2g19,3nzu,3gi6,5dtt,1gaf,3nii,2xm8,4att,2vex,4jx7,6h34,4tvj,4lh3,
3pvw,1ai7,1kc7,2rl5,6c0r,4rcp,1nhv,5mw2,4mg7,1drk,4mzh,1r6g,5xpl,4o74,3bu8
,3cgf,4nie,2a3b,1xos,2wlz,1a37,5lpj,1yvh,4xhe,2lct,3lau,3zki,1g2l,4uiv,6b4
u,3jqf,3ixk,4pvt,5lpl,3sdk,5km3,1t7f,4z2l,4tk1,2j9a,3bbt,3tjc,2qi4,4acg,5t
uz,4lbo,5uuu,5ckr,2h2e,5qai,1qxk,2wyf,3f0r,1rd4,2ipo,1ukt,2xx2,3hvh,2bmz,1
jqd,5d24,4cpy,4bdk,2xu4,2evm,6alc,4r5y,4oc5,3kdm,4gk3,5wvd,4pz5,1x1z,2duv,

6fnx,5u4b,4dfn,4agp,1n9a,5mny,3hqh,3o8h,4qem,3l81,4c0r,1utp,5z68,5tuy,5fb7
,3wv3,3n7r,3bze,2bvd,2fx6,5vcw,4ibf,1zom,4f6w,2fdp,1i91,1d2s,3ikd,4w9e,3fe
j,4ntj,4wk7,2a29,3f15,4u6c,1ing,3wq6,4ock,2yb0,3wtl,5yl2,2hz4,2gbf,3r8z,5c
so,2j95,5jxq,4ofb,2fr3,5b4l,3ovx,6cha,4qwl,1tl9,3mo2,3c8e,3k5x,1uw6,3fhr,1
b3f,5a8z,5bvk,4o62,2hb1,3qxd,3i4y,6g9d,6gop,5ajv,1oar,5flt,2zhd,2xey,4ikr,
4ckr,4ufg,5xvk,2vmd,5oci,1syo,4lq3,1dtq,5bpp,6fg6,4cki,5wei,3fl9,5cnj,4os5
,6awn,3atm,1ypj,1odj,5msb,4f2w,2h5a,2w3k,5w1v,1pi4,4kx8,5m25,1dhj,2qki,4b7
2,1n0t,3oag,5dyo,1ql9,5u7k,5mod,5mtx,4u6w,1yk7,3d8z,2pnc,5em8,5cil,3iny,5l
2t,3u6a,5fow,2owb,1k1y,5nf9,3ijg,1qan,4ad2,2p09,4xir,4tqn,5q11,3zzh,3qel,5
jha,3rxh,2ha0,5xqx,3q2g,5wl0,4bic,6ccn,1mh5,2ai8,1xp9,4ef6,5jzb,2h8h,5sxm,
3dm6,2ym8,6fmi,5f1h,4qyg,2qtu,3vfb,1vwf,1ivp,3vf7,5typ,5igq,4h1j,3sug,3h85
,2viy,6hf5,2poq,5q17,6afa,4mpc,6c0t,4zsh,5dit,6aom,3h8c,2e9v,2hfp,4u5n,5ca
q,4m2w,4tv3,3oy8,1pot,3owk,3arn,2dri,4ymj,3qto,5ix0,4jr0,5i5x,1nzl,5u4a,4e
g4,1wbw,3nee,5fdp,4cqe,1hvs,2w6q,1n8v,5ct7,5dx4,2fdd,4ydf,5c2e,3qsb,5zk3,5
vgy,5ot8,6boe,1c29,2pjl,2euf,6cnk,3as1,3abu,3dpd,5aku,4k6w,4j3l,4nau,3fpm,
5w7u,3c3q,3mg7,1c12,3buo,3rhx,5lyh,1b0h,4ynd,5y0x,5enb,2a5b,5mqe,3l5e,1tl1
,3way,3gs7,3ex3,4np9,5mxq,2cnh,1swr,3t1m,4eyj,6mj7,5f01,3rkz,4ln2,4jia,4l4
z,4qr4,2er9,5vnb,5xff,4prp,2jqk,1gi8,2ynr,1gfz,5nk2,1rsd,5umz,3jzf,4k69,6b
5o,4o4k,4jal,3nht,4j1i,5xof,4ie7,4f9w,5tvn,2qoh,2jdy,3wym,1w31,5c6o,3nim,1
vso,4z2g,1o37,3lbz,4bqs,3tfv,5i8g,3utu,3msl,1bra,2k46,4mg9,2w76,4wyp,5jcj,
3b92,3zha,2i4x,3wbl,5trk,2n8t,3ge7,4unn,2ria,4wgi,4bds,2y06,6bhh,4raq,5i56
,3vv8,2xk4,4az0,3ov1,4ycw,5ei4,4u1b,3bv3,5etp,3kcf,6fod,3eq9,1awf,1eat,4gi
u,3vyd,2pvh,5aaf,2hdr,1lgw,2lsp,4l02,6b7b,3ioe,4c1m,5kxi,5yof,4obv,3r6t,1u
j6,1a09,4yk6,1rzx,3cy2,6f8v,1xor,4la7,1qwe,5mxo,2opb,3psl,1aqi,5y5w,2j4q,5
vfn,3e37,4fzg,5qim,6da4,1oxr,5hgc,3at3,5ieg,6cq0,2ioa,3lpg,4mzo,1w7x,2aia,
5v2l,5abg,5x5o,2xcn,5toz,5tp0,4z22,4wlb,4pd5,3zbx,3pb9,3f5l,6bke,4p7s,6dhc
,5enh,4m3p,4jmg,1k03,4pni,4asy,1a4r,4deu,4hy9,4g0p,5b5p,3wqv,2r3m,6q73,5ar
5,1drv,4ua8,4loy,5eh8,4wy3,3jzh,2aov,6dj2,5t27,1kdk,2og8,10gs,4uw1,4hge,5q
cn,6c4d,1bai,3i5n,6epa,3dgq,5tzx,4h3b,5a4q,1e5a,5n93,2exg,5k4l,1qpb,1f3e,4
a0j,5ula,4cc3,2oym,1dis,3ns7,3uz5,3ewz,5q0r,1qbo,1nd5,2qzr,4oq3,6e9l,2n0w,
2hr6,5vse,5jsm,1ssq,3l4u,1y2k,2r3k,3e3u,4ibi,3kgu,1tni,5hfj,1dva,5mno,1qbu
,3px9,5xpm,5q1b,4zjw,4gqp,3r21,3lpp,4bdt,4ks2,2v58,3bft,1il5,5yco,4p5e,3rh
k,5fq9,5ko1,5bwc,4q7p,1ro7,3lil,4mwq,3wf6,1tft,3hpt,5t3n,4oo9,2b5j,2bet,3n
uy,5nea,3kdd,5ls6,6f2n,4rac,4j47,3iut,4uvv,2i4v,1a8i,5k48,2hpa,1mrs,6fv4,1
q8u,5aqz,4ytc,1ibc,4eft,2v95,3me9,4o0b,4kn0,3g2j,1ai5,1n51,1gnm,4zur,4zsj,
3v8w,4tzm,1swn,5ih9,3gen,4r6t,2ykb,5u7i,4e81,3v01,3n4b,1jmi,4dcx,3at4,2c6c
,4mzl,5ybi,5tcj,2w54,4o13,5ufc,2xs0,4flp,4yqm,3o8p,3tpu,5yft,5mql,5w6e,2yj
x,4pqn,4m48,2iiv,4ohm,4a9c,6en4,3wk4,3p50,3bpr,5gwz,1mai,5wys,4oel,1uy8,4u
01,4ibc,1qti,4jpy,1q41,2gvd,5aip,5i0b,4zyt,4yxd,4qt1,4w4y,1c8l,3e5u,3kqd,4
uj2,3k4q,4o42,5o1h,3b0w,3t64,1hty,5dgm,3bjm,1b8o,4l3p,2b1z,4yyi,5ceh,3l3m,
3ebl,4bo8,4fvq,3fup,5ot9,3eax,3vhk,3cda,5c1y,3r2a,1obx,4tzn,1d7i,3qd3,3wka
,3ovz,3ihz,5iis,5vo6,5tc0,3fqk,1dm2,4y63,6mvx,4ucu,5oht,4jff,1n8u,4xua,2yb
p,2cgf,3d4q,4gsy,4i32,2psv,2ych,5lck,1xxe,5wrs,5trg,5dp6,2lnw,5kr2,4utx,1a
8t,4qw5,6emu,2itt,4m7y,3kqo,5ku3,4n7u,1oit,1w1t,4j26,4nrp,4yh3,3nzw,5zh2,3
vw9,2hyy,1qj1,1csr,2we3,6mnf,4ijh,2jdk,4kcg,1kmv,3buh,4ovh,1mxu,4mg8,3lka,
4twy,5vsb,4ofl,6cdm,2f3k,5aqp,4tkg,5w4e,3zot,2yak,3qkd,2pv1,5mz8,5gut,3avz
,2q8g,3ilq,3zw3,4ckj,1qbr,4q08,3zpu,5u8f,2ydv,3rtp,4b95,2f9b,2uz9,4ehg,4xs
z,1bkj,6f22,1tet,3f3w,4abd,3sjf,2byh,5jsg,4op3,6fsy,3twv,5d25,2vto,2oa0,6c
sq,6dh3,5jq9,4xkx,4tmf,4brx,2ghg,2gvj,4wnm,5ayf,4osf,1u65,3lzs,1i90,3gdt,6
eru,5ops,2mpm,4cc7,3ckp,1o4d,2vvs,6ep4,4bkz,4aom,1wc6,1mpa,2pl9,2p53,4zyr,
3gqz,4og8,4mlt,1mf4,1xxh,3p3g,3g0c,5dbm,4xhk,5lny,3g8i,5g60,2zas,2z7h,3kfa
,4a9u,4gj7,4nwd,4ei4,5yjf,2won,2f2c,3mg8,3i06,2wa4,3hzm,3wkc,4j7e,3dxm,3m9
f,1gzv,4clp,5a09,5tg5,4wkn,1njf,3ce0,1tpz,6bh0,3vfa,3su1,2vtn,5yp6,5twj,4i
74,5zk8,2al5,3bh9,4djo,4oi6,5m4u,1wva,3uvx,2aod,1pqc,4n4t,5fdi,1lcp,4ere,1

ci7,1ykp,2qju,1ftj,5hk9,5y3o,5q0v,6ayn,5os1,2j9l,2qm9,2x6j,5ei2,5izc,3u18,
4zek,4s1g,2wq4,1nfy,6bnt,4jai,2jb6,5ahw,4uit,3bmn,4gzx,6gxu,4gki,3ccb,4ezq
,5v7a,6dzq,4az3,4ucr,4pnt,2wtj,5amn,4ux6,3t8v,1i33,3r7n,5f00,5nvy,3k41,4aq
3,3tdh,3wdz,5nib,6b8y,1v2h,5jpt,1xge,4umn,2xrw,2vvn,3t07,2vpp,1ghv,4ydq,2d
bl,3ho9,5w88,2g71,2lk1,2pwg,4ai5,1o41,1tsv,5al3,4fk7,2o4z,4a51,4gwk,3ay0,3
own,4e5g,1lcj,1hk3,4rhy,4yoi,3prf,5z99,3slz,4tmk,1au0,2qwg,1bdq,5h8b,4emt,
6bec,1g9d,1qf2,2r0u,1f2o,1b9t,2c1p,1hn4,3skg,4ee0,6c2y,4pg3,3uw4,1wdn,3mho
,4yl1,6cex,3b7r,4pop,5f02,3d7b,1xbb,2zft,4i8z,4oya,3otf,2xhm,5c20,1zrz,4rq
k,5k8o,1tvo,6fnr,1dwd,4zlo,1nyx,5aad,3kqw,3nw7,5ti6,6e59,1ywr,2xn5,4cy1,1y
fz,5e3a,4a7c,5ljq,5h9r,1gi6,2b1p,1kc5,3kpw,1q5k,4aml,4ibg,6e49,2w8y,3kmm,2
w6o,3hdn,6cef,1u8t,4e8y,4gj6,4hbm,4g3f,4eok,2rox,1t48,2xd6,2fjn,5ndb,4j77,
1n43,3ui7,5e73,1h1h,4mhs,3ohf,3kdc,4edy,2wzs,5h9s,3cth,2j34,1h62,4jnm,6bil
,3mhc,4zw8,3g90,4f7v,4eh8,5wkh,4ycm,4mk8,2xj0,2tpi,4clz,4ko8,5qaj,5dus,4xm
7,2uzl,1k1l,4g95,1c3e,5g10,4nnr,3e64,1fq8,4c71,4b0c,3ft3,1ybg,4y2t,5dk4,4d
ds,3ggw,5axi,3ueo,2r9b,3ppj,1meu,4z7o,3n8k,2jup,3p9j,3hxe,1e06,3ika,5yyf,4
kc4,3su3,4z7h,4x2l,5hcv,5ir1,1f9g,5law,3o99,1yci,3g3d,3aje,2o5d,3b7i,6af9,
2gvz,4qmt,3qj0,2y9q,1nw5,5wg8,4bzr,1k6t,5hd0,3fui,1lek,5kql,5d21,1iig,5ewh
,2vhq,2gh6,4x6y,4mbl,2v54,5ug9,3hvk,3qts,3q43,4nvq,3co9,4uxh,2gj4,4dwb,5q1
6,1uyf,5jbi,2il2,1yt9,3t6j,2zu5,5buj,2xiy,2fs9,1al8,4cig,3cgy,4mdq,3st6,4d
zy,6fcl,1m0o,3spf,1lkk,4uu7,1zky,5d9l,1zd3,5t6f,1g2o,2i1m,3khv,3o88,5fi2,3
idp,2pov,4x11,2qe4,3qtz,5alm,4pli,3fts,2x4u,4o45,1tkx,2q8h,1mue,4eop,2j7e,
5akk,5wtt,3h9o,3v4x,5b6c,5boy,5hdz,5nkg,6ajg,4dum,3whw,6b59,5aan,6fo5,4pmp
,5i0l,2xk9,5mon,3d4y,2xib,3c2r,5mfr,2ra6,2xl2,4w5a,3zm6,3zm5,3zhz,3ogq,4rr
q,4wxi,4asd,5txy,5u0w,4xta,3t2v,2q9m,5lyw,4kon,3sv2,2j7d,3hmv,6g92,1z6f,5a
46,3gnw,5k03,2bgn,3ohi,3acw,3hng,4j06,1owj,4fbe,1gbt,4fmo,4g8v,2fzc,2vx0,2
o1c,5unp,4o7c,4rxe,5t90,5jyo,2wzy,1tfq,1tqf,3s78,6g0v,2ei6,4r95,3peq,3o9f,
1mq6,1k6v,6gfs,6q6y,5m39,3wz7,1nwl,3ard,5e3d,2woq,4gq6,3iw5,2h15,3avh,2azr
,5vsk,6ckr,4n84,4eh2,4cpw,5f91,4k3r,4dgn,2yer,5ayt,4cpz,4y29,5tq6,3bcn,3ro
c,5lwm,3gjd,3k5v,4zip,4und,6exm,5qak,4u0n,5wo4,1rrw,6b2p,5hls,3pjc,1cin,3b
2w,1kmy,4ys7,4f0c,1zgi,3vqu,3uoh,1qr3,5i8b,4l7h,2aay,4jlm,3wnr,4en4,5w0f,4
lxz,4cjr,4jj7,2h5i,2avi,3ezv,4j09,3hkw,3uxm,4b5d,5vcv,2vtt,3bl9,2y1n,5nin,
3uvo,5anw,5nvw,4b9k,1j4p,4io5,4dkp,2jdt,1iem,4lm0,5wyq,5nr8,6f7t,5a81,3lnk
,2xuf,5ort,1h1q,4twd,1e1y,5lo5,4anm,1e1v,4tki,4wv6,4htx,1t13,4euo,6gy1,4ci
v,3dux,5knv,5hn7,6gi6,5vb5,3f5p,2kfh,4b3u,5nf5,5mes,4w55,1sv3,3p7a,6frf,6e
ks,5afm,5z4o,3cow,5hu1,6f9g,3ds1,6a6w,2g8r,2ze1,5q18,5cau,1s38,1zge,1me4,3
cj4,4pr5,4gzf,1q54,5l9i,1z6j,2ez5,3unk,3qwc,3o86,4j51,6fyi,3cj2,2viz,5ekm,
3f9y,2uy5,2brb,5g45,5jgd,4j1e,4ikt,5opu,4mwc,1t7d,2viq,3ocp,3wuv,4yay,3d7f
,2vh6,4foc,2ra0,5fwg,1o3c,4bi0,1kll,3qtq,3cl0,3ho2,3vw7,6h37,4rlw,6dak,3p3
t,3m11,6fdp,4c73,5ksx,6bcy,1okl,4gqq,3sji,2x2i,1hkj,5hu9,5vom,5fdo,1uu7,5i
ay,5nu5,6ghh,4q06,3apc,1re8,5uey,2ha6,4aaw,1tcx,3lpj,3w0l,3s45,3n5j,1rhj,5
d0r,2qp6,2vqj,2zaz,2zir,4mq1,5alt,5he5,3atp,5w5j,3ekw,4xas,5mwy,4mwy,2wzf,
2z1w,1d4t,4yk0,3hnb,4h3j,6g36,1rhr,2r3f,6bcr,5owh,5idp,1odi,3vw1,4n6z,3qcx
,4z89,4pkb,2ewp,3d9z,4c5d,1xoz,4meq,3so6,3tiw,6h7u,3u0d,1ur9,4lar,4css,2a3
c,1h3a,4aqc,5l2s,5vi6,6cmr,2wf4,3chs,5uc4,5tca,2x6e,4gne,5ey9,4ih5,1mto,4q
wi,5nai,4ym4,3prz,2nnp,1vij,5bvd,5swf,3pn3,2pql,3ekv,3nq9,5c7n,1els,4k5m,6
bq0,1o2k,2aux,4x69,5mts,1e9h,4ykj,1mkd,6i3s,1zpb,5mo8,2q92,4u70,4wph,4xy9,
4ith,5aph,5cy3,3fat,4ly1,2vti,1f0q,4wsy,3ej1,3ine,3rux,2vvu,5a3t,4qtd,5a5s
,3t7g,1xon,4gah,5flq,2hw2,3aig,4hl5,5wzr,2imd,3oxz,1ndv,5yji,2hxm,3l59,4jp
x,1ws5,4uix,2iv9,4dwk,4qvp,2c1b,5cw8,2r6n,4cws,2d3z,2oc9,4ehz,1ett,1fdq,2a
vo,4r0a,1zzz,3moe,4mmp,5muc,5zh5,3zqi,6f08,6elo,6dif,3mf5,5qas,3fu5,3rv9,4
jne,5urj,5oxk,4y3y,5hki,4tyb,2glm,2xnb,3tt0,6gmq,4i5p,1f92,3psd,5hcy,1q6n,
4tmn,1tmn,2gg0,3zep,1c88,4fea,5nwb,1ppc,5myx,4ci3,1z6d,4kww,4ixu,1leg,3a5y
,4bo3,5cyi,5uzj,4mw0,5j18,5ml3,2bmk,1e66,2brc,2fwy,5oxm,1bn1,4qlv,4qip,3ms
2,5wxf,5yib,3ig7,4pgb,4p73,2y4s,5zh4,4ch2,5tex,3b4f,3poa,4zyx,4m0y,2ot1,5e

91,1yc5,3iqi,2xpc,1o2s,1pmu,4l58,4mx1,5am4,2hs2,4yne,1ctt,3l5c,2zm1,1lxf,2
f1b,4zcs,4zw5,2oh0,5n2z,3f19,2obf,6f9t,2f01,6euc,5vee,4oyo,5moq,3ft4,6cbg,
5wuu,5f5b,4xs2,1ype,4rwl,4hpi,4e34,1yc1,5jy0,2fxr,5e1m,5oq6,1nhz,3pjt,5jzi
,4elf,4fxy,6dpz,5a6a,2waj,2fqt,3mxy,1y91,6biv,4j1k,3zmi,5xjm,2fv5,5db3,3ry
j,3b95,3i5r,2ajl,6c99,3bx5,5ave,3muz,2ye9,2rf2,2j2i,5g0q,4yuw,2r3g,4cwf,3t
3h,5ajz,2bgr,4l9i,4igk,6cz4,4qfp,5ty8,4odm,3hs8,4ele,5zia,2pvj,5w5k,4m5l,3
wz8,4egh,5w7j,4jfw,6eda,5tpb,5m5d,4o71,3kag,4u93,6eed,4zam,2jbp,4z3v,2pu0,
4zzd,4f14,4h4d,1pxh,2hwp,1u9e,4wko,5kqx,1tog,4yqu,2yi0,2xf0,4uof,4io7,4ds1
,5diq,3ti5,4x8u,2lp8,3avm,3kqm,4zph,2wou,4jyu,3ip8,5ti3,4cku,3h78,3vdb,3ch
d,4i5h,5i2r,3tyv,4uwk,5j6a,3lk0,5k13,2hj4,3bfu,4ajk,3n45,3te5,3ujb,5v9p,2b
zz,3a6t,2y6o,3b28,1hvl,5a2i,4mx9,4g8y,4dko,3kdb,1lf9,4qmp,5q10,3hqr,4d2d,3
w32,4ksp,6f8b,3h6z,5k0h,3h2n,5ke0,5w4w,5mxr,5igl,2h9p,4iuu,4d8a,3dvp,1lox,
3ao4,4h38,4bdb,4j1p,1o4b,4mro,5eel,2ay3,3e8n,6g8m,2pyy,4m0z,2mas,4ks3,4p72
,6ccx,3ttn,4jsr,6aqq,6em6,5o3r,4rqz,4hkk,4qll,2q6f,5he1,4fe9,2wu6,2pj7,1bt
y,1ule,6dry,5ezg,5h17,3s7b,4x48,3atw,1kug,2hdu,3odu,3l9m,1d1p,2ihj,5mgk,2n
1g,6hk6,1mq5,4kp5,1yyr,5al4,1pzj,6axl,2z4b,5mym,4gv8,2cni,4xub,6cjh,3ig1,4
m2r,1tt1,5ime,5oei,6d6u,1utj,1dfo,3ixj,5anu,2ogz,5bwb,5vad,2x9e,6epz,4yv2,
2jdh,1w13,4eos,4x1f,4gao,5c8k,4k5n,3pj1,5hz5,1x7a,1h9z,6gjm,3ipe,3fuj,4m3d
,5g11,4asj,5xig,4ybs,5gmi,3d04,3dv1,2fie,4twt,3ary,4anb,6hmg,4n9c,2lsk,2ks
b,5mwd,1gfw,5gja,3u3z,2f7o,3mzc,5w9g,5yz2,4nmq,2y2k,5lz5,4qz7,1bug,4zmf,1o
8b,5jf3,2qft,5jop,4jaj,3s9i,6cvy,3oaf,4hlw,5ta6,5mae,2yg2,5m2v,4zxy,1g9r,3
iqu,5i9y,3pch,5kkt,2fqo,5mn1,3e3b,4jlj,4tq3,4zga,4jlg,1pf7,5ewd,4dhf,4ywa,
5fxr,4e1k,2zmd,4jyv,1fkg,2lwi,6iik,3zxe,6dgx,2vx1,3hav,3pkn,1trd,3u10,2a0c
,3ly2,2xn6,1irs,2ntf,3rum,6iin,5hzx,1urw,2xb7,4umr,5apr,5ngt,3q71,3og7,4oe
g,4tte,2ow6,3sff,3eu7,2zju,5tx5,3v8t,1nqc,3t2c,4afh,3vhu,2d1x,2h2h,5nvz,4q
y8,3w2s,4w9h,4r6v,5esq,5v5d,3rbu,3d51,4q07,4jrv,2in6,3bki,5ov9,6ayd,4zyc,4
ybm,4xip,3ibu,5kde,1br5,1lan,1gwq,3ggv,3cke,4av0,4qpd,4urk,4o04,3d4z,4uct,
5uwj,4b8y,5nvc,6f23,5w0e,4m84,2hdq,5c6p,2amt,2rgu,4ax9,6g9n,6hu2,6cho,3v7d
,5iz9,2vev,3dej,2hxq,4mvx,2c3l,5c29,1we2,6f9u,1m6p,2f80,2ooz,4py2,4zcw,5kx
7,4m13,4dhp,4kz6,5ypw,2zdn,1qf5,2i4w,2xu3,1wbn,5lt9,3zmv,4efs,7cpa,2i72,4r
po,2h13,4obo,3f88,6g47,2y6d,3o57,5a0c,2fl6,5mja,5jal,3aqa,3g9l,5g22,5znp,1
hi4,1y2a,6ay5,3cf8,3f5k,1a0q,3et7,4eky,6bvb,5u94,1m1b,3ryv,2peh,3vw2,5e1e,
3i51,5fb0,1rtl,2i4p,1a9m,6au5,4jyt,4k3k,2c5o,2qu6,4hwr,6bqk,4bqh,5cs6,4dea
,4x3r,4iid,6gzm,2y2n,3fuk,2ypi,1b3l,4zsq,4o44,2e1w,3rdo,4zme,4zz2,4ucs,3sl
0,6ey9,5arg,3p3r,6an1,3eor,2uue,1n4h,3gk2,3oot,5i5z,3hv3,2hzi,5yc2,1g48,3b
xg,5wbq,4a1w,5q0q,4axm,6g91,4q0l,1d8e,1bb0,1lpg,1gmy,6gin,5yy4,2pvv,6ht8,5
j82,2x5o,3orn,2nmb,1pk0,3rik,4lkd,1v2n,4ufl,6ahi,5bsk,3uza,2w8f,4nka,6bef,
5abw,6bkh,5l30,2clx,4an1,4ufz,5nk6,1syi,3zsy,5ul5,3irx,5ux4,4r5n,4bdc,5fut
,3e2m,3lgp,2ivz,1akv,2ydi,4i11,3zxv,4m3e,4kwg,4g2w,3o95,6gu4,2doo,5yun,3n5
e,3mqf,2y5k,2vnm,3g0g,1qfs,4o7b,3ty0,4uil,3st5,3f36,6fqo,4pkw,5j6m,4xbo,1h
xk,6ghj,1h8y,2pq9,5em3,1rlp,1yvf,4cqg,3srb,6fmk,4o6e,5xmv,4agq,2j3q,1ke9,1
mfd,1a5h,4b1j,4i0f,3up2,2ay5,5mqx,3lpu,3bmq,5wfw,4j5b,5alp,5ee8,4qj0,1ka7,
3k3h,4np2,3bwj,5osl,5all,4jze,3b3x,3sm0,2xa4,3t3v,3t1n,4yp8,4bc5,5f08,4qw6
,5ofv,2bdl,4y83,4y4j,2prj,6cfc,5eth,1oky,2ban,4blb,3f38,2uy3,6fdq,4qmx,4bo
5,5wh5,6cvv,2oph,3owb,1xlx,2cf9,4av4,2wxv,5v8p,4eqj,4aoc,5jk3,4xtx,2aoj,1v
7a,3lzv,3bum,3fxw,4qhc,1b6j,3f80,4or0,6dq5,5lsx,4bdd,4x8v,4od9,3vye,2yix,1
i7i,4eoy,2k4i,2bv4,4z0f,5dp7,4xiq,3d20,5llo,2ymd,4hn2,5n31,4ffs,4b5b,5jf1,
4i73,2pqb,2i19,1cbx,1gsz,5ws3,4h3q,5a5q,4uvy,4guj,1i7g,3c8a,4ll3,4pp0,5hk1
,4rux,3coj,3d6p,1ndj,1o79,3nsh,5hl9,2e27,5iui,4og5,3wdd,1lnm,3igb,4x24,4ts
x,2gmk,5ox5,1bqn,4e8w,5xyf,4uco,1eas,2b7f,4m5u,5evk,3ipu,4p5d,2fsa,4ay5,4b
cp,5thj,3tam,4jsc,3ug2,5e7n,1ofz,3uo4,5clm,3gr2,2m0u,6f7q,3sdg,1sme,3g2i,1
oyt,3h3c,1xff,4oc6,3b3c,1xa5,1fe3,1g32,5olb,5a85,2oz2,4d8i,5swh,1y2d,1inc,
4obz,1c7f,2clh,1qkn,2etm,4i5c,3vd7,1p28,5j7s,1y2j,1aht,4iq6,2pe2,4f3c,4ih7
,1hkk,4puj,3o1g,4w9i,3qrj,1qj6,4azg,4joo,6dxx,4sga,1gja,5k4j,4yef,1sbg,3s0

0,5fcw,4txe,4mr5,1bxo,5xxf,5jic,4abh,3nzx,5edl,3pcf,3k98,3qqu,1hgj,2qch,2y
im,4dtt,4l09,3f07,4hki,4cl6,2z5o,4e7r,5amd,1c1v,1ym1,1s64,2w6t,3tl5,1w1y,4
eoh,5i58,4z5w,1u2y,6hu1,3wav,1nxy,3n4c,5c91,2kzu,5kre,2jkk,4hkp,4y2y,4w54,
4bnu,3lp7,3bla,1gni,2idw,2xy9,2q6b,3uxd,5o9p,3ao1,3v2o,1i00,5ewj,2rgp,2xln
,6ma5,4d2w,6eww,4lkq,4o7a,5m4k,5g3n,1wm1,4fzj,2nyr,2mc1,2qky,6eh2,5v1y,4gl
w,3arb,4gq4,2vin,4rss,3q7j,2h4g,2x2c,1gt4,1fo0,1ftk,4kby,1ch8,5fnu,3sv7,2v
vo,5uah,3k97,2wpa,1a4h,5lhg,6eeh,4mse,3nnv,6eq4,5f1l,3q0z,3elm,2uzd,3p2e,1
r0p,1j16,2qhz,5h0b,4nyt,5ij7,4l2l,1afl,5vd0,4i10,1opi,2ves,5tyn,3gf2,3nuo,
5w2p,4uv9,3sut,2f3f,1dmb,3wd2,4xm8,4kp4,1hy7,4xbd,5m6h,1g05,4zh3,5d0c,2e7l
,3kba,5nhh,2pax,1p0y,2l7u,3wns,5u4d,3axk,4qpl,5jt2,2rk7,5lss,3sni,5aaa,5wf
m,4pb1,3oe4,5m63,5xg5,3lle,2yac,4odk,2x95,2ig0,2zdl,4ay6,2c1a,1nny,1nw4,2f
j0,3kah,4db7,1bxq,3ifl,5f9e,4p2t,4exh,6d2o,5t2b,6eee,2oi0,3lpl,4tk3,1o6i,6
eux,2ojj,3pyy,4edz,3jy0,3r5t,4xdo,3qo9,4u2w,5nme,6hdn,4mre,4ryg,5ez0,3c14,
5kzi,6bix,3uug,5ldm,1o2v,4llx,3lw0,2op9,4nhc,5wqj,5wi1,4e20,6fjf,2x2m,5l98
,3qsd,5lcj,5t23,1adl,2wa8,4q9y,2rkg,4gid,3f70,2xk6,5t8e,4lxb,2xp2,3juk,5uf
0,3l1s,6evr,1lag,1awi,6ej4,1pmx,4kln,3wgg,5g61,5n69,4o9v,3kqc,1him,3cd0,2k
bs,1moq,2f10,2zb0,2d41,4bdh,1ebz,5lcf,5u13,4qwk,4cd8,4d85,1tvr,4l6s,3pd4,1
hq5,5tiu,1o44,6g22,2ckm,4btk,2cli,6cb5,3o0u,5u4x,4lp6,5afk,2o3p,1zpc,3qo3,
3d1z,2z3h,1hms,5ox6,5fpp,3f3d,5t78,5hg5,3mxe,4i7l,1tuf,5lmk,3nu9,5om9,2qi3
,3l5b,4v0i,2nv7,2wkt,4ndu,1aq1,3ddq,1vyj,5ods,1b8n,4xhl,4jft,3n7o,4og6,5j9
z,5ufr,4qjm,3g4g,5jox,2std,4uvx,2yb9,3qaa,5mwo,2onz,2hiw,1xnz,2agv,4qgg,5x
28,2dw7,2ohk,4oue,1o4a,6ds0,3t5u,3ij1,4klv,3lgl,2wey,1qk3,4i0d,1rti,3clp,4
f8h,4qwj,1bmb,5jar,5a5z,5ofu,2yiu,2z5t,5emm,4ibm,5lzh,4xh2,3t09,2p95,1fmb,
1gj5,3s72,5ia5,4kp6,1s9t,2fhy,4del,3ao5,4xii,5cp9,3vb4,3sha,5el2,2uup,6bfe
,4jv6,3e8u,3ubd,1tys,4p10,5ekj,1d5j,4bpi,3n23,3amb,4gue,4n8q,1o0d,4d1j,1a9
q,2vtq,1h1d,5uw5,6bfp,3tfu,5xyz,2q1l,4m7j,3m8u,5kbf,3btl,4ctj,1koj,2ajb,3u
nz,2xgs,4pd9,5g1c,5w19,5vqw,5yc8,4ufd,1w83,4qdk,1tyn,6buv,4amw,3dnd,1d7j,1
nzv,4ryl,2f6j,6bbx,5lgp,6ay3,1fwv,2qg0,3mg4,3qmk,4dma,2r3c,1d6s,5dsx,1tg5,
6do3,5n7v,1g5f,1c5q,1lb6,4pv7,3ii5,4rab,4eke,2w10,5i86,1uho,5vsf,3g08,2zxg
,4btl,3hb4,5j4y,5lgt,6boy,5kva,5mka,5wa8,2i0e,3ptg,5vp9,4mw2,3djf,6b7d,5t1
a,5hrw,5e2o,4xuc,3vg1,6c4g,2csn,3q32,4q9m,3vry,3fv7,5lhh,3fh5,2qry,4f9g,4c
8r,4m6p,4riu,3dp3,2r3i,3bwk,6ew3,4bs5,4g3e,2wvz,3wc5,4nb6,5xmr,4ps5,4dvi,3
uvm,5os7,2xix,5alw,3iaw,2xnp,6dvo,1cgl,4xv3,5dya,3c79,2e9a,6dil,4caf,4tpw,
5fov,5n3w,5ttv,6f3b,5cas,5aqg,3dz2,1xhy,3t01,5th4,4i80,4q3t,2ceq,4yff,3bti
,5vll,4hy4,6g3y,4ykn,5lu2,5wg7,3fnu,6g84,6ap6,6hpg,1c3x,5if4,5wgp,3d6q,2we
r,2qrq,4cfl,3uib,6fgf,5tx3,3plu,1zzl,6db4,5tpg,3k8o,2fxv,3c2u,1w8l,1di8,3u
dh,4cu1,5gvl,5nvx,1bt6,6d9x,1vjb,6b31,2qo1,3daz,1v0l,5nuu,2qtb,5ewy,4i9u,4
afj,5hzn,2ohq,4eqc,3p8h,5y53,1kat,4kwo,2hiz,2cmf,4bgx,4hys,4lww,3k39,3rdq,
4ega,5fsl,3ehw,5gnk,5n8b,6h29,1dy4,3nu6,5dxu,5jf8,2qd9,5zaj,1oif,2v8w,1ywi
,5uiq,1xr8,4yvz,2y82,1pkx,2qbw,1ebg,3a4p,3ful,4d62,5qb2,6bgx,1ela,5j59,2q6
3,6cmj,2p9a,5nwk,3w54,4rrg,2l1r,2gsu,2wva,2vpn,1qji,4gts,6gnw,4afg,5ty9,4p
s7,3r2f,3qak,4dgg,5f67,1kak,4mc9,4dpu,4u5o,3h06,2b4m,4q99,4dgb,4hw2,5uvc,1
yxd,4cix,4ehm,5his,2ihq,4kmd,5cin,5v7i,3itz,3il5,3brn,1h4w,2ilp,6bnk,1me3,
5fh7,1rgk,3iub,3p4q,1njt,1ya4,2x6i,3e81,4yl0,1at6,3pr0,5wf3,4uzh,5f2k,4j93
,4dwg,5ti2,4pcs,3qcj,4qjo,4dpf,4qer,2kp8,4o2p,4pd6,2op3,6got,2weq,4lh7,5l9
7,6bed,5o1e,4ezt,5y8w,1err,4rj7,2yol,5t68,2ltx,5g17,1mxl,4c7t,3uvu,5a3r,4y
uz,3nf9,2fix,3pgu,4kn1,3mi3,5dpw,3e5a,1y1z,5j8i,4e5d,3cd8,6ccl,4x9r,3rtx,2
zmj,1xp6,6cks,3fw3,4znx,4nyj,1q1g,2ews,5o5m,4w4v,2w1d,5d1j,5fbe,3ipq,2qk5,
2ydj,3gv6,1mxo,5l9h,3odk,6cze,6c98,2wmx,3qa2,5oax,6gnm,3q6z,2oqv,5exn,3s53
,5lzg,3mo5,6drz,5nn5,4x1q,2mow,3ow3,4ebw,4a4c,6ema,2h6q,2fjm,5uez,6et8,6ep
9,1avd,2q80,4nj3,4mdr,5ely,5dri,1v2r,1hk4,5xfj,4z0q,4heg,5h3q,2buv,4l7d,3n
b5,1g9b,3oay,4aj4,1bxr,2w9h,2iws,3oy1,3qpo,2hog,3rl8,1zgb,1a4w,3vvz,5ia4,6
f1n,1x70,3q5u,4do4,2pcp,5m77,4a50,1elb,1rri,2ydm,3ldp,4k9g,2ptz,3uo6,2qwd,
5fdz,6fiv,1hqh,5q12,5anv,5k5e,5aer,1jys,2zu3,2oiq,5lwn,1i9o,1npv,4piq,2w0j

,6c5f,1w8m,5gjd,5yql,1m83,4djv,3ns9,3zn1,4eg7,3u4r,6hu0,4awq,4l6q,1ad8,3d9
k,5ucj,3piz,3ekt,1g9a,3m2w,5q1c,1mv0,6emh,1ttv,5u12,1lhf,1w1p,2i4t,4z68,4g
qr,3uqp,1rhq,3oxc,4mc6,3adt,3ivi,3r8u,1udt,1o36,4ou3,6apz,5gmp,2l84,2gg9,3
o64,3uo9,4qht,6f6r,3hy7,1nox,5u6v,3ans,1mdl,3d2e,2fsv,5t2m,3p79,4je7,3gta,
4q9z,6f55,1gu1,5y6k,3ert,3ant,5a3h,3zdv,3o1d,6ar4,2ym4,3s3v,3fk1,3pbb,3r6c
,1xh8,5lh4,5q0w,2psu,5gty,2qi0,2nn7,5vb6,4b8o,1zp8,5uqv,4pci,5f5z,4rj4,6dh
4,3lce,4ea2,4y6m,5d3p,1gsf,4l7l,1tkt,3a29,5ljj,4gpk,3ayc,3qkv,3ern,3zhf,1f
4y,6bgg,5uz0,2znt,5du4,1utn,5ayy,1lzo,1gym,5k8s,3kb3,3ijy,5oa2,5wio,1yei,4
hlh,2eg8,5tkd,1kpm,3tpr,3mt9,3tcy,4qxr,3uyt,3o9l,5ejv,6ekn,2fky,5vp0,4rxa,
2xpa,1kav,3ecn,5vlh,4lko,2g83,4m5r,5iql,5g53,3fhe,2xj7,3u78,3zt3,4hkn,4h58
,4f1l,5q0t,2jkh,5w13,3r88,1yvz,4qmq,5aag,1b6m,4i47,6d5e,6c91,1z95,4ca6,4ys
l,4gbz,1aj7,1wcq,3m96,5t92,6gjl,4yxo,5l2m,4wov,3l2y,5v5y,3a3y,2gmx,2fo4,2h
5j,2r05,2qtt,4jn4,5npf,1gt5,4jfd,2qhr,2sfp,3rx5,4e4l,4mao,1zkl,3syr,3jzj,5
h9q,5one,4io6,3lp2,2i4j,3rlp,5eta,4yth,3r24,3gsg,2of4,5inh,3ed0,3zlr,5llp,
6esj,5c8m,3dkf,4z6h,4x6o,2j47,1usk,5w8j,4x1p,1xb7,1lqe,4apr,5khm,2ga2,2ly0
,4zro,4e26,4g16,4uvw,1f5l,4ipn,4hwt,4g90,1kds,4bck,5k6s,5eqp,1vwn,4uye,4gt
q,6fe1,6gjj,3hb8,2zdm,5alz,3eyg,4lkk,4os2,1j4q,4o36,5dtm,2wgi,6faf,1mx1,3l
fn,2i1r,3hzy,1a4k,4zl4,1dub,3qi3,3i90,3qgy,6gfy,1o2u,4xyn,3rk7,6atv,2wu7,4
tnw,2uwo,1z6p,3p8o,3my1,3s3q,5w0l,4tw8,6b8u,5jg1,2azm,4qw3,5ntk,5jq8,3gcq,
2zcs,2pvw,1o0n,1ph0,3nu5,5loh,1t08,4m7b,1qpl,4ge5,4w9s,5fat,5l2w,5nx2,2gst
,2pix,5myn,3ump,2xoi,4r7m,5ful,1kf6,2on3,5wa9,5tw3,1q4k,6fu4,5umx,2bdy,4ez
y,4crj,4pyx,5t97,2cet,1fao,1n46,2ooh,3dgo,4io4,1xjd,1q72,3mag,6euv,4g5f,4d
gm,5o1c,3ken,4d2r,5fnq,4z7f,2o3z,4xgz,4i2z,6g15,5ypp,2x09,5lt6,3k5g,2xej,1
uyh,4tln,4mrw,3c10,3sdi,4tw6,3pa4,3q4k,5l3a,3b9g,4ohp,3nq3,5vfj,4rqi,4x7k,
2q8s,1ca8,5a3x,4wf6,5oh4,2qu3,5dq8,6ffs,4loq,4prb,5vwk,2yhw,5eob,5dgj,2i4d
,5wlo,5lo6,5aln,3p5l,4q1y,6c4u,1gi7,2jkt,4yv1,3ctj,4qfr,4wmy,5c84,3ie3,5mn
n,3l8v,6eij,1o2n,4nmv,5ok6,4yab,2vnf,3r0w,2uyq,5j19,1y2c,3nth,5ni0,4tpk,1v
yq,4txs,2wgs,5gv2,1b3g,3kad,6hgz,3dng,4u7q,5fnd,2c5x,5ho6,2aqu,1ke6,5g5f,3
wff,3m36,4bb4,3ai8,3ee2,5u14,4xe1,4ggz,4av5,3bgc,5i4v,3g0d,4bkj,2aei,4hup,
4yv9,4uwf,2ovy,2oo8,4dlj,2v83,1ct8,5qan,4nwc,4msc,4iti,3guz,6i8l,2yof,1oyn
,3qti,5nf6,5n7x,3t0t,3oyw,4ab9,3aw0,2x2l,2lkk,3oyq,6c28,2ofv,1b58,4cpt,4em
7,1t5a,4xyf,3duy,4xh6,1j15,3b7j,2isw,3eqs,3tkz,3dgl,3qw5,6df4,2jjb,5auy,2r
cu,5mqy,2aoe,1d8f,1kl5,4zsg,3e0p,3su6,5tz3,4hco,3i6c,2ow1,2yjw,4nue,1uyd,4
hod,3oik,2viv,4wtu,4gih,1b9s,5i40,4zb6,5fns,4x6h,5hg9,5naw,1h37,1uj0,3e9i,
3i5z,1v2v,5wb3,3lpt,3k4d,3nhi,1cbr,5uis,3f68,5nk3,3okv,3krr,4gnf,3zyf,3ivg
,5d3n,4rs0,5oxn,1owd,1w7h,5o4s,3hrf,2r3h,1yp9,5oyd,4iif,4uvb,1b9j,2bgd,6gi
u,5eek,3tnh,3gp0,3bxs,2ay1,5cap,3ta0,3mkf,4uyf,4zx6,5a3n,3hek,6b5r,1fbm,5w
ou,3g76,2rkf,5j86,5lav,2rkn,3jzo,3kr5,2vo4,4lzr,2srt,4wbg,1vyz,3ebi,2qbx,3
gbb,3vnt,3erd,1o34,1lbk,6czi,4d1c,1k1p,2bmv,1jlr,5hx6,2igw,4o0r,2isv,3kr8,
4k3m,5xiw,2z5s,2w97,4u03,4p4d,1pwp,5j27,3w5e,5a3s,1zz3,4y5i,4rx9,4ty7,6g4z
,4j3e,1s3k,6ajh,2wei,5v5o,4p45,4nbl,4rxd,1cp6,1r5h,4nnw,4cga,3kb7,4phw,3zp
q,4b3c,1wv7,5hja,1mfi,3jzk,1hsh,6aro,3vyf,2v7d,4k66,5eeq,5mos,3ska,4bg1,4q
l8,4lgg,4anw,6gr7,2x7s,4ie4,1p05,4zbi,5c6v,3qzv,5etq,1nhw,2xx4,1uys,3zsq,4
h75,4d0w,4pml,3gyn,2fm5,4r93,5usq,4e3b,2hwi,3kl8,2vr4,3o0e,3dab,5h8g,4ynb,
1kr3,4w9d,4msl,5k8v,5ix1,5zaf,6cve,4h85,3d5m,3v78,5uox,5d1u,4fcm,2zz1,4i1r
,5hkm,4y8c,1fkn,5he2,3n3g,3g5v,2evl,3k3j,1utr,3ixg,4u44,4ahs,1o2y,3fcf,5dd
e,3bgm,2nxd,4yhq,4k19,5y7w,3v3m,4ph4,1nja,5i23,3rsv,5n17,4bt5,2ojg,5x72,6e
q7,3ehx,1a5v,6clv,3qcs,5cy9,3bgp,3nf7,4dmn,2dxs,4msk,2wmu,4hws,5c2a,3lco,4
x3u,5c7a,1pa9,5he7,3m67,1j7z,3s5y,3oe0,4kql,5l8y,5cks,4cc5,2rcb,3bi1,3jzc,
2qzx,4u73,2an5,2uvm,6g2n,1dzj,6b97,5y6d,1qvu,4ymx,1yej,6f3d,5tg7,2w1i,3dv5
,1vyw,3sw9,4qz3,8a3h,3o3j,5gn9,4b7j,2i4u,3m8q,2fu8,4hfp,1swi,2vd0,3pz1,2jo
9,3q4c,4bzo,4tyl,6fuj,2x7c,3hg1,3pty,5f3i,4q9o,3e1r,4j78,4ucv,6h38,3wtk,4l
no,2h65,3s4q,3lp0,4ajo,5maj,3d7d,4xe0,6hpw,4p0b,5fsb,4f6u,5h0g,2zdk,6hoy,6
bib,3rpv,1kel,4v24,5k1i,4j4v,3kai,3c9e,2v59,5cs5,5jqb,6as8,5dw2,1mq1,4gw1,

4gj9,6e2n,4qlu,5jog,4iu1,3tyq,3r2b,5alr,5c4s,3pa8,2qq7,4qn9,2uwd,1yye,1oir
,1gpy,6ert,3d7z,6dq8,4lsj,2wxm,3pj2,3nwb,3qxc,4hwo,3afk,2rk8,6acb,6g85,1kn
a,4pvx,5hex,3jqa,5xms,4an2,3lcu,3rdh,2r3y,4w7t,6few,4cd4,2gg2,4ga3,6anl,1y
yy,3r16,4al4,4uru,2ovv,4ya8,4re3,3bgq,5lpr,5fap,5cei,3v5q,1maw,4p4f,4jx9,4
xqa,3ghe,3fci,1zfp,2l12,4lil,6eq2,5vdv,6b0v,4l0i,4zsl,5yu9,1p04,1rnt,2chm,
1nhx,3ckb,2vot,5n1p,2jke,5ztn,4j82,3hxb,3qvu,1hk5,4pyo,3atl,3jzi,4ad3,4b6r
,3g8o,4kc2,7upj,5eyz,6do5,5wfj,2j4a,3kmx,5oy3,4o55,2qi5,4jfs,4h3g,2y68,3fv
2,3ioc,5jyp,2gde,4mm8,4jjm,5ccl,5umy,4n7e,4f64,3p9h,4hz5,4kin,6cqf,3feg,3a
aq,3sl1,1uxb,4oee,4i7d,5ll5,4qlk,3s9y,3t3g,2p3b,2jbj,2x8i,4ui3,3po1,5ji6,3
u90,4lts,4xrq,4jzr,5nkb,1km3,2qnx,3myg,2va7,4ymh,4nms,5ar7,2h3e,4lp0,2ay9,
5trs,4fse,6cdg,6ful,4x8n,4w4x,5m29,4ocx,3sov,4fxp,3ex2,5uwl,4wpn,3bi0,5nwh
,1vik,4uj9,5tg4,5hrx,3wz6,1mm7,5v37,5eol,4egi,5n21,3vhe,3cj3,4w9q,2qtg,4z9
3,3jqb,5f3c,5ll9,4zts,5aei,1o9d,2iw9,5b5g,1g54,3v5p,2yk9,1ez9,4c6x,2g24,1m
9n,5f60,1vzq,1rt9,5jq5,3ha6,2yj9,4nra,2h9t,6afi,3el8,4kni,5v79,4fxf,3ncg,4
x3s,1dif,4aa7,4d1b,5av0,2clm,4l10,1exv,2ltv,6g1v,3rwh,5yfs,5xkm,5mjb,2i03,
2v0z,3udm,3i3b,1h1b,6esa,4ai8,5ak3,5j20,5jjm,3wto,1std,1jjt,4kxb,1jdj,2chw
,3ll8,5j79,5adq,4r74,5n53,3ljg,2lsr,3rk9,1add,5ja0,5x02,5cc2,3u9c,5d12,1o6
q,1xn0,5f20,4lgh,5etr,3mj5,4km2,4f9u,6el5,1hmr,5n9k,3are,1lv8,5n99,4gly,1s
h9,4uns,4cpu,2or4,2rkd,4psq,5wcm,5vsc,4nl1,4um9,4wa9,3fxv,2obj,2ohm,4zw3,5
jao,6htz,3n2p,5dda,2vwy,4ge2,5d7e,4wx4,3sgv,5w12,4gmc,4dij,1r5g,4r3w,5hn8,
4fk6,5ggl,2ks9,1tq4,2qu2,4oyk,1my8,6mv3,3kqs,3gz9,3eta,1uv5,2bok,1b42,5mlw
,5d29,4qr5,1r6n,6f6u,4pio,6cee,3mp1,4pz8,6fgy,5moe,5yfz,5zwh,4kb9,2wmw,1na
x,1o4n,2znu,5gvp,3ma3,3k15,5qae,1v48,2psx,4d8e,1m5d,2v2c,2ql5,3h0y,3wpn,2q
a8,1rs4,5tgy,2b7d,4pl6,2w8j,3tdz,2wxj,5g2b,4nrk,3gwv,1kne,3kqe,5khg,4uiu,4
kyh,4e93,2qg2,1a4m,4byi,5ngz,4c1c,6fam,5lgq,2hb3,4c1h,3ms9,2pnx,4qmw,1lzq,
3aid,3psb,5w6r,4c4e,4jxw,5ngr,1utz,3r7o,3umq,3hw1,1tkc,1abt,3mbp,3i9g,1rwq
,4iz0,6dh2,4rx7,4kwf,2cn8,3bar,1cny,1npz,2bys,4y6p,4y6s,5q0s,2uxx,6eaa,5cs
2,4c1e,4mgv,2wxf,5q1e,5o83,3r4o,6fyk,1x8s,5b25,4f39,4tk5,5nhp,4lov,1kui,3a
rt,4d2v,1bnv,4kov,6cyc,4fkk,6hvj,2j4z,2p3c,1f0s,3dy6,5lz7,3mww,1hvk,1x76,2
aez,1bio,1lhe,3k48,2krd,4bbg,5otc,3kqp,4esi,2rku,2kpl,1erb,2xx5,2fl2,4a4v,
1lqd,1lfo,3hf6,3bm8,1a99,3zly,6cwn,3d8y,5icz,1hgi,4qgf,6fug,3tn8,6f3g,5kq5
,3tz2,1xws,5uov,2o9j,3coh,3h98,1mes,3ot3,6f1x,5gs9,2hha,1pfy,4j3j,4rn1,4ze
6,3zpt,4f9y,4lbu,2nno,5v42,2fyv,4j8m,6hjk,4ha5,3tu7,1izh,3o9d,6g4m,2r2b,2x
c0,1g30,3anq,5uv5,4ty8,2vew,3br9,1df8,3fgc,1utt,6cd8,6f4w,3l8x,4fe6,5adt,5
nzq,6htt,5k0t,3pn1,4ryc,4nga,4crc,4xcu,3f3c,3obx,6agg,6cq5,3w2r,3dsu,3zs0,
4wkv,3w07,1ntk,4dk8,3kgq,4anv,4ery,4jzd,3tjh,4jwk,4hyi,4kne,3u9q,5ih2,2h02
,4c6u,1i8z,4bid,3lmp,5ohj,4j81,5dey,5k8n,1pz5,1shd,3hhm,5n0f,5kh3,3nba,4u5
s,2ndo,3shc,3vid,1ik4,1al7,1fq7,2x4r,4kz3,6cen,3s8l,2aoc,2xh5,2p93,4qfg,5h
uy,3ebo,1agm,1fv9,4jnj,4c72,1vfn,4xg3,3hl5,2xah,5n58,3mof,3r22,5o45,1utm,3
p8e,1kl3,5o5h,5lpk,2zdx,6q74,3miy,5bpe,2vta,1bnq,6f90,4myh,5llm,4poj,1q6s,
1jws,5xmp,4ahv,4e4n,4qgi,4o75,3ogp,3dk1,4bdf,2ybs,4ght,1e5j,1veb,4wrq,5cqu
,1ow7,5nsp,2wtx,2rqu,5kpl,3g19,3gy7,4bhi,3rjm,3f3v,4h42,4y16,1o3g,1utc,3wz
u,6b5q,1u2r,3sx9,1nju,3drp,6azk,3r9d,4awi,1ce5,5vd2,5kzq,2bal,1y2f,2xxt,5h
lp,2y8c,5auu,1bwa,6gbe,2zv2,3f7b,1e4h,6arn,3ryw,6fr0,5wbz,1c8v,5kau,1v41,4
oew,5fbo,3l9h,5hi7,3igg,4hy0,5enf,1cwc,5yjo,2e92,186l,6epy,3qk0,6cq4,2jkq,
3gb2,5xmt,2ybu,3arz,2o5k,5c13,2amv,4std,4gfm,2fm2,4pv0,3qx9,1hnn,3bl2,5dhp
,6c7i,5pzn,5ur9,1pye,3upf,3ooz,3zcl,2vkm,2jdl,2uyi,1usi,1ezq,4qz0,3g5d,3ry
8,2vnt,5ehy,1mqj,5gs4,5xg4,3h59,1bv7,1br6,1mns,2ybk,1bcj,3ktr,1e02,1ykr,5v
ja,3r5j,5edu,3kga,6b5t,5f74,5qaq,1jpl,5hmi,3mjl,1eei,4n8e,1hrn,2o4s,1agw,4
yz5,4wn1,3tc5,2pjb,6ea1,5epy,3vc4,2i0g,3ah8,1ogg,1onh,1tmb,6bau,4ad6,1a3e,
4drn,1aw1,5fl1,2h2j,4prg,4ibe,2yem,3fql,5zwf,1x0n,3pi5,6ehh,1cps,5akl,3g2t
,3vsx,4dhl,1hsg,3vhv,3ro4,5myl,4lyw,3sl5,3e92,4o91,3ipa,1imx,1u71,5dx3,4hv
i,1h5v,3p7b,5v8o,2rg5,5nx9,4i8x,4rcf,4yrd,4bcn,4d09,5f8y,5mwp,3n76,3gba,4l
yn,1unl,3e6v,4dce,3rsr,1nlp,5dxg,3rcd,2cji,5zwi,3o9a,2x2k,4n5d,5l4f,5gx7,5

yqo,4gzt,3tki,5dd0,5ivt,5mnr,3asl,2oc0,3l3n,5izf,1i6v,2y56,4x0f,2h4k,3ivh,
4yy6,1drj,3hku,3kfn,6gmd,2c3k,1w5v,5oha,4myq,3new,1o5c,4k5y,5kbi,5of0,5dhs
,1y19,3hky,5nka,1wax,6h9b,3i7i,4b6c,1h3h,5xw6,4jql,4cra,3d4l,2nwn,2zyb,3rm
9,3q6s,3g4i,3zqt,4bbe,3olf,6gxa,5h4j,4ljh,4a4f,4nw5,2vi5,3eqy,4tpp,1pi5,4b
t3,3kiv,1pb8,4xyc,1zd2,2vur,2k2r,5j1x,3pfp,6g37,3nsq,6eq8,2wi6,1bxl,5eci,5
jrq,5ehr,4qz1,1nmk,5zkb,4f65,5a7b,5sym,3wti,1j1a,4nwk,5l8t,5k0f,1mqh,1h3b,
1wxz,3tt4,5k0x,2ez7,5wbp,4ddl,2ygu,3e7a,1aqj,5m7s,2odd,4eon,3fq7,3vf9,1pxm
,4umt,4rlt,4f8j,5dp9,1ung,4r5w,5ais,4btu,4hyb,3nyx,3nif,5hmk,2qyk,3tz4,3qx
m,6isd,6g97,5ccm,5u0z,2wbd,3aav,5vzu,2hai,6evn,2yga,1p17,5fus,5elw,4azt,6e
a2,1xbo,2yhy,4u0m,6b3e,5hff,3iu8,1dqx,4o0v,5t1u,4gs6,2xei,6gn1,2pze,2j87,4
hy1,3udn,5nmg,2pgz,1wdq,4er1,2l6j,4cmo,4gbd,3itu,5a7j,3su4,3r6g,5ea7,6eiq,
5qb1,4o61,3grj,4xjs,2x85,3b66,3le6,5t19,3itc,5ett,2esm,3dc2,3p2k,3u7s,3c6w
,4fcr,2clo,3d0e,2jqi,1ekb,4ovz,3soq,1hef,5os0,4htp,4tk2,4j22,4etz,3gus,3s7
6,4pyv,3c49,1ktt,2yz3,1xnx,1elc,5i24,6eif,5t9z,2qt5,5h1e,5ekh,5vdw,3fbr,2z
a0,5wej,2yel,4anx,1rtf,1r5y,1pzo,2ydw,5kam,5i13,5exw,2xyr,3qs1,2x9f,2np8,5
ci7,1m7y,1fcx,5ok3,1hp0,3l79,4jq8,4bhn,1i9p,4hyh,1jil,5f2s,1bwn,1xh7,5kbg,
4bg6,3qfy,4ycu,3lxk,4w57,4kxl,5mk9,6er4,2rcw,4jpc,6h1u,3iae,3fnm,3mxr,3c2o
,5eva,4kbi,3ogx,5owa,6bmr,4mgb,5ka9,2bza,3sad,4wj5,4o1d,4pnc,4mzf,1f9e,4wh
l,2imb,4llk,4ih6,1vjd,3stj,3usn,5tnt,4i4e,4hg7,5dxe,1hc9,3dcc,3d27,5azf,5c
5h,4m7x,1ysg,5nve,4dcy,2qrh,1r9l,3l0k,4iuv,5j31,5ulg,2wc3,2jxr,4qwx,4joe,4
djh,3sml,4prd,5etn,1ihy,3ut5,4gjc,5c1x,2oxy,5can,5hz9,4uxq,3nzi,4hfz,4mn3,
1jif,1svh,4zsa,5y24,5f9b,4mka,5wf7,4o0y,5w8h,1t1s,5w7i,4o2e,3g58,4jmu,3dg8
,5u7o,5owt,2oic,3mt7,5ka3,2wgj,1orw,1vea,1j01,4yec,6b7c,5wg9,2fv9,1e55,3cf
s,5nhy,4x6s,5ak0,4q1w,4o5b,1b32,2fzk,2p3g,1h24,3qfd,4xu0,3pe2,5ow1,4uyg,3d
u8,4cby,1mtr,3bug,4uj1,4omj,2y58,1onz,3esj,1y2b,1zyj,1fig,3cd7,2hf8,2v3u,1
o4f,5w0q,1ql7,3kv2,2wez,2loz,4zg6,5f4l,4mo4,3mam,2h44,3que,4y32,5cwa,5hyr,
4mnp,3wix,5tw5,4dem,5di1,3zlq,6en5,4ogv,2r6y,4k8o,6c7w,6eqv,3dx4,5qa7,2qlm
,4tn4,5vm6,4x5r,3k23,2q54,2pe1,4k2y,4rfc,6hqy,4ie3,2qbq,5o07,7std,4mm7,3ax
z,1a85,5etb,4ylj,2vpe,2qln,5zfi,5wbk,4bty,1nvs,5ftq,3dyo,2c9t,2h6b,5nz4,5a
r4,1xfv,6g6z,4e1n,2q2y,2zwz,2ozr,3fei,2xe4,3nin,4py1,4j52,4mrf,1ngw,1m7q,4
m8t,4qhp,1my3,5fgk,2ff2,5qat,3chf,4kox,2nsx,5v1b,1det,4cq0,2iwx,4isi,4bcg,
1pro,4ebv,2jh5,4mm6,4b82,4m5m,4pp3,3v31,4yt7,3vp4,3imy,1rbo,4b1c,2w7y,1k9q
,4erq,5tzh,2a3x,5f61,5orj,3efr,5g4o,3el1,5lkr,1mn9,5wdw,4oym,2iko,1h39,5dd
d,5nu1,4qz4,1o5p,4i9c,3wmc,4ajl,4zud,3kig,3wde,6fdu,4psb,4bw2,1hk1,1pph,3w
ut,2ate,3nw9,5ceo,1g2m,1o3i,5ald,2hjb,2cvd,5za1,4cnh,4pnw,4uwl,5c1w,1m0n,2
nsj,4imz,4x3k,4zgk,3pvu,2fah,6ex0,6gz9,1ke8,3tkw,2xfj,4bo9,4cqf,5sy2,3fh7,
5u4f,1k27,1msn,5ow8,5ekx,4re2,1dar,6iil,1bmm,3tsk,3tzm,3ttz,4ibd,4zx5,2c4w
,1qf4,2xgo,3bm6,2zfp,2uyw,3h8b,4acx,3ouh,4zy1,1c1u,3btc,1azl,4ivd,1o4i,5ev
b,5dpx,4k9y,3wtm,6mxe,5ygf,4mpe,1o2q,3ru1,4qtb,3qw6,4yb5,2hzl,1b5i,1dbb,4g
j3,6bfw,5y5n,5jdi,1kv5,4der,2xc4,3s3r,5lso,5cu4,1ysi,1n2v,4jje,5akw,5f3z,6
dl9,5wgd,3muk,3uw9,1o32,1jq3,2zpk,3hk1,2f1a,2haw,4psx,4i54,5d3s,5m56,2bkz,
5dxt,1qw7,4abu,2ggb,5fjw,4k5p,5als,2xjg,5eqy,4g2j,2a4g,3hf8,5em9,4ztl,2g1q
,4phv,2ylq,5tdb,6dh7,5kz0,3rz3,2vww,5t4b,5xs8,5j7g,1f4f,5mev,5cgd,3vrw,5t8
j,2o48,4cgj,4cts,4cbt,5ofw,3q2m,4ce2,2qqs,2j75,4yw7,5wip,6afl,6b16,2cgu,6e
xi,3mt8,4o24,1z34,3fvg,1w6j,3py0,5nkc,1os5,3t70,2nwl,6dq4,1n3z,4rme,5z95,1
tx7,4yc8,3t0m,4e5w,4f1s,1hvr,1xzx,3fty,6au9,4eu0,4d1a,2p7a,4z2k,5ap7,1sqb,
3wt5,3eqb,4o3c,1rgl,3r7q,2ay8,5enm,2ha7,5ak5,6dug,3wgw,6bfn,2fx8,4rxc,6bny
,6h36,5ugm,5trj,5xpo,1jmf,3ugc,4jhz,3p44,1zp5,1v2o,5tbe,1o2r,4wsk,5vdk,3rn
i,3kc3,4mi3,4pb2,5euk,5ukk,2k2g,4a4q,3m6q,3btj,4gtv,5u0g,4zh4,3v2p,3kqt,4i
m0,1ni1,3wp0,3vi7,4tkn,6bg3,1o1s,5d3l,2qc6,5lp6,2br6,5ku6,5d3c,5ny3,5hjb,4
txc,3waw,5b1s,4trz,2pwc,3nu4,4x3i,3djk,3qw7,3ivx,6b8j,1y0x,1ros,4rwj,6bto,
3ztc,1jlx,5h13,3l0v,4ycn,5qam,4ke1,3wv1,4mep,5tzc,1bil,4fl2,4u6r,4k6i,4g2r
,4pg9,2hzn,3r8v,5d9k,4gg7,3d9n,6cct,2c93,1uxa,3nfk,5f27,2w6n,3k8d,4pl4,4b9
z,4uiy,5fl4,2bbb,3b50,1qon,6ce8,1ydk,4jv8,2bw7,1li2,4qag,3hyg,2qtr,2cen,4k

3n,2fes,5kat,1dzp,3gcu,4nbn,4ctk,1okx,1nzq,5fb1,4r5v,4b7n,4det,2xm2,3exe,1
jje,4wup,3sjt,6h8s,6bx6,5tqf,3lpk,3h1x,4ps1,3c72,2dua,4w97,5diu,2cbs,4zy4,
4alx,1y6b,1tnj,2vo7,4qye,4no9,5ct1,4elb,5sz2,4u0x,4ztq,2nnd,1xom,4u0a,4abj
,3uh4,1pop,3hv5,3zll,4rak,5u7m,5tuo,5ur1,4iqu,3kac,1t7r,4lqg,4um1,5mwa,3dj
p,4zeb,3r91,4o77,4r5t,4ijq,4mxa,3d0b,5ld8,3hdz,4bxn,3vb5,5q0j,3rx7,1k9s,6c
jy,4zjj,5i3y,2wos,1bbz,4rx5,4nzm,1m5c,4djs,5ect,5ejw,4fvr,5vtb,6f20,1u9x,1
zea,2zzu,3rxp,2z3z,1oeb,1gux,4g8r,1hk2,1g4k,5dfp,5vc4,4g55,4o7d,1nc3,6cdp,
4gu9,5gvn,3l8s,4bis,4lwi,2baj,5wik,5a7i,4pnq,2xde,6e4u,1b38,3wzn,5nn0,4fl1
,3q6k,6eab,3s68,2dq7,1yw7,4x8p,5vgo,2o9k,4yo6,4lv1,5ose,4ogt,5e8a,4k76,2pr
9,1z3j,2g72,2l1b,4gdy,1lq2,6g0q,6cv8,5tri,1g7q,1o4g,4cg8,2ohl,1v2u,4b83,3w
yj,4hrd,4kfq,1no6,5lax,3egk,3f7g,4deh,5tmp,4rad,3d78,5l72,3avj,4rg0,4qev,5
nt4,5svk,2w08,3lxo,3ljj,4kif,2jdo,1v0o,1k3n,1rgj,4zt8,6dub,1zdp,187l,6dkb,
3n6k,1tnk,2h2g,4mjq,4a9i,4pre,2gni,3bgb,4ddm,2p99,1mwt,5nhl,6b4w,3sou,3sm2
,5qik,1loq,4yqh,5mi7,4ynl,2f9v,1qbn,2r2m,3uod,2w0z,4ewo,1fsy,1tmm,3va4,6b0
y,2adu,5dtj,3k3a,1sm2,3ozs,3o8g,5g42,4h3a,2fwp,6fbw,5tzw,1xkk,3tdu,3efj,3a
s2,2ity,4zwy,2nmy,4amy,5kad,3qtv,3ncq,3p58,6h1i,5x13,4d1d,3zev,6aum,1ftm,4
dsu,4hla,2qoe,5em5,3u0p,3dx2,4kn2,4ce1,3np9,3t2w,4b7r,5o9q,1rth,3eq7,3i0s,
2pgl,2yj8,2z6w,3v9b,6fs0,1dhi,4hdp,5svz,4mz5,1a1c,4p0n,3n7s,2aow,1j19,4k3o
,1m7i,3s9z,1igb,4o9s,5xyx,4mex,5jy3,4jht,3hxc,2c3i,4itp,2fl5,4b12,3o6m,2vd
4,3tib,5yj8,1aze,4yad,3w37,5e8z,3pz4,2p4y,2xda,3n4l,1i1e,1tbz,2bxu,1g6r,4h
5e,3fuz,1f2p,1b51,5lm6,5gx6,4wn0,3h21,3vd9,1x39,4bjc,6d5g,3nnu,3d32,3l9l,5
i3w,4kqo,5w38,2p2i,4ohk,5l2i,4jlh,4whh,3u6i,2khh,4gu6,1snk,1ogu,2p0d,1zeo,
1iq1,4knb,3lrh,5a5r,5iok,5t6p,4dx9,2a0t,1c5x,1qxw,3d83,3pwh,5g46,4wy6,1yw2
,1bm6,4yhm,1tka,3qpp,4g19,1pdz,4fnz,5mby,3zt1,1y3v,3tv4,2qmd,2phb,1bzf,1m2
r,6g46,3hvj,3mhl,4w9c,4imq,5k4i,3abt,3vuc,4oyt,5qb3,5yv5,4n1b,1pip,5iu8,3z
ke,5f1z,4qvx,4zlz,4qfs,5ai0,4op2,3tfn,5cnm,6dge,1htg,3rey,1o6r,3w5n,4l52,4
b76,5qar,4nj9,2kaw,4ezz,3hkt,5e1b,3ufa,4btx,5mgj,2ha2,5cst,5ea5,5kgn,3jq7,
1qy2,4fpk,3hq5,1at5,1hge,5ty1,4b3d,1lyb,5mrd,4zt4,4ovg,4pp7,2w5i,6ced,4elg
,5j71,3i6o,4mx5,4pnm,2g0g,1x81,4wkp,4kxm,1wht,6q3q,4gee,2f70,2wd1,6aah,4rf
m,2wvt,3dx3,5t4v,1owh,4k7o,4z7n,5lla,5ehw,3jsi,1ohr,4k3h,5y8y,1pu8,2mg5,3l
5f,3tfk,1t31,3t08,4iho,3i91,5fdg,5ntq,5wa4,2iqg,5tol,4tz2,2d1o,5ene,4mc2,6
ax4,4aoi,1u3r,4ngq,3src,5zzw,1sw2,6g2m,5fwa,2q7m,1xn2,5g6u,1puq,5ha9,3s56,
3ckt,2jaj,2qlb,1l6y,4tzq,5u9d,4nzb,3ccc,3isj,5kbr,3u92,5duw,3ijh,1uti,5tw2
,2hd1,4wuy,1riw,3u8n,6flg,2qzk,5w6o,2v2q,6e86,4e0w,5jf4,5a8y,2nq6,1tsy,1re
k,2xht,2gtk,4kom,6en6,3zmz,3efs,4whr,5j1v,1ld8,4o37,5qa5,4c9w,2zdz,1l5q,3k
ek,2olb,4dru,2h42,5jnn,1w0x,6chh,3av9,3uzc,1l83,2osm,5i89,5wb6,3vdc,1b4h,5
e0a,1nyy,4bqg,5otr,5axp,2w1f,4z1q,4q4q,4o07,3g0f,4alv,4jfx,5kjn,2jal,4u0w,
2k0g,3uwk,3k26,5iq6,6hjj,3o0g,3iu9,4xg8,4xya,1zfk,3jvk,5wxh,3t0w,5yg3,1xpz
,4b60,4j2t,3ds0,2l0i,5y1u,1pxk,4da5,1z6e,6fi5,4pry,8lpr,2ya7,3zv7,4bnz,6bh
5,4x2s,2vth,3tvc,5fky,1i43,5jau,4qsw,1m21,4xsy,2pvn,5htl,5o7i,3skh,5wic,1f
vt,4rio,3si3,6dnp,4c4n,4ufu,2ork,5dkr,3m59,3tmk,3btr,5nps,2oah,5ywx,4nxs,3
r8i,1z1h,4jik,3oui,4pzx,5o1g,5auw,6cjv,1t46,1uwb,4lgu,5xvu,4x3t,5dtk,3qkl,
3meu,3ove,5mw3,2zcq,4n7y,4e4a,2avs,4e3l,1hbv,3tl0,3g15,6c5t,4ayu,4aba,5ddb
,5mmg,5k09,3fj7,4y3j,3dxg,5a6n,5lvd,3c2f,1w51,4m3m,4x6k,6h7b,5gow,5n6s,2f1
8,4kvm,6d5j,2qhn,1ppm,5y2f,3fi2,3vzv,4r73,5h5q,5gic,1g6g,5j47,4tmr,4inb,1w
6h,1inf,5alk,3u8l,2bvr,5bnj,5nk4,2c6g,5tbn,5ug8,2wbg,6bgv,3fzc,5mgf,4ngp,4
akn,3k27,4gfn,1vjc,6ckc,3bqc,2k0x,4o0a,4m1d,3cfn,2ds1,3wnt,2ogy,6d28,6ffi,
6eu6,2it4,6h78,3s0e,3u1y,3r04,2nsl,4knj,5q14,1e1x,5cs3,5hfc,5zob,4erz,5ant
,6ft4,3th0,1ijr,4lnw,4pyq,2r3t,3ik3,5mkr,2gz7,1joj,1j17,1c5o,3avf,3u0t,1c8
5,5w99,6d4o,2qic,2tmn,4cc6,5q1h,5k5n,5n55,3rxj,3njy,3srg,4bgm,4m0r,5nxv,5j
1w,4bbf,4mot,1tl7,6gf9,4ivs,1bmn,1bju,1w4o,2pmn,1k6p,5akz,4ca7,4joa,1mfa,4
qqc,5e2q,1dgm,2fxu,3ejp,1a1b,5k0j,3h5u,5ha1,2rg6,4b77,4hzt,5fe7,4pgd,5uwm,
4rro,4rr6,4o70,5cfa,4ke0,4ju3,2wtw,2qhm,3m3z,2vqt,5q0d,3gcv,4fmn,3s7l,5kgx
,2n9e,2vmf,4x63,3fc1,1ok7,4pzv,5dd9,4nru,1udu,6fmp,5fxs,4dk7,4e5f,5ml0,3v6

s,4eeh,5cjf,5e1o,6bw8,3h2f,1yly,4avs,1sri,1avn,3i25,4fm8,4jg7,3fu6,1g7f,4l
1u,4dr9,3ocg,5wal,4dq2,1uz8,4qjp,2onb,4hgc,2lyw,5xgl,1ydr,2r0h,4q4s,5j64,4
riv,4cp7,5d9p,1il4,2qaf,1idb,3ft8,1hos,2xnn,5myv,5ti4,3gi5,2wxh,4eo6,3spk,
1g3f,5iy4,5h1v,1mem,1rdt,2c92,1n7m,5dif,2py4,4k9h,4ehr,1nfx,4cst,3h0v,5z7j
,5ta2,5nu3,3k3i,3gpe,3zq9,5cqj,6axk,4len,4nmp,3b25,3ch9,5djv,4hxw,4euc,1hv
y,2jk9,3ncz,3bu1,4bb2,3wqh,6alz,3qch,4jv7,2jmj,3nan,5vds,1oth,2aie,3dln,3m
v5,5egu,6dki,5am0,3vrt,6dpt,1ew9,2w6u,2ggu,5jsj,2xwd,3zya,5uwp,2exc,5e28,5
k32,3g4f,2xng,4x5y,6esm,1gi4,3d2t,5ttw,3ekx,2uwp,1klg,4y2x,2dwx,5lrq,1od8,
1ceb,4ci2,4m5i,1o9e,1nq7,3ejr,4wrs,4eor,1g1d,5ofi,2kce,5eko,4mhy,1ndy,2cc7
,2nnk,5ybe,1svg,5aes,4pkr,5j5x,1cka,3n7h,3pxe,4kz7,1tcw,3v5j,2xp8,4bv2,1gy
x,4i6b,4oyb,1ox9,2wf2,3zvv,5f90,1rdj,4azb,3o5n,4uub,3d1e,1n5z,6b1y,2o7e,3o
9v,4hf4,6ety,2qi6,5jas,3gss,1c5c,5l9l,3ifp,1czk,4fi9,1cs4,5e88,1mui,5vp1,3
vbt,3sh0,4avw,2asu,1qf1,3hjo,1iiq,2r2w,1ztq,2c8x,3wtj,4lys,4lv2,1u9v,1dx6,
3mtd,5ii1,6d5h,4nku,1w1d,1e37,4pte,3c7p,4glx,1hvi,5f4n,5f6u,1y8p,3kwb,5ggj
,3gx0,3ztx,4uiz,3ueu,2vl4,2wnl,4cl9,5aqn,3ipx,1i72,3dsz,3mhi,4ap7,2xcg,7pr
c,4e3o,4int,1zz1,5bjt,2xas,4ny3,5fi7,1owi,3qd4,1ew8,4hdc,4ac3,3h52,5nzp,1l
bf,1b6p,2ylp,3qxt,3kyr,1ksn,3twp,3ti8,6ewe,3t19,3eu5,4dvf,2flr,1oe7,2or9,1
v2m,5fjx,1m2x,2c80,3nm6,4nak,5y12,2vwn,5chk,4dv9,5akg,5x9h,5m7u,6axp,1upk,
1j36,1uv6,3f2a,1yet,1ov3,4kwp,1eoj,1yms,4o4g,3p0g,3tsz,2hrm,4tz8,5k4x,3hzk
,5w5s,4ekg,6gh9,5l26,2lsv,6eqa,5fp0,2zc9,2jk7,3h03,3wb4,1xow,4hra,4mk1,1y2
0,5yc1,5lx6,2er0,5x4m,4yih,3mbl,4a7i,5koq,3cx9,4zom,5sz6,2vqm,4yyn,1syh,1k
wr,6cyb,3pm1,2zym,5fv7,3shz,4dmw,6b5j,1wn6,5vd3,1njc,3ew2,5ia3,4a4e,4a9n,4
iue,2ewa,1akq,5ew9,5n8e,5a54,4kow,5w6i,5mgg,5oh3,4hmh,4f3i,3kd7,1w9u,5da3,
4n6y,5jid,1juy,1f0r,5iw0,4ui4,3tkh,2gc8,5qc4,3wzj,5l7h,2kdh,1uyk,3sv9,3uex
,5os8,4b7q,5a6i,5om2,5lgu,5uwk,5u1q,5unh,5nw1,4ck3,1g42,2e9d,5k4z,4l7b,1so
2,6esn,4pzw,1ida,3jzb,1o2h,5qby,1nde,6dne,1f8d,1nvr,5ult,1db5,4fr3,1g27,5a
lo,4o15,4ryd,4oar,4zy0,4pd7,2aq7,6etj,2zga,5luu,2l8j,2wky,4yrg,2v22,2ndf,1
jsv,4u6z,5wdl,5nxi,6i8y,4u6x,4mw5,4ufj,3ith,1wb0,3arq,3dp2,1lst,3fqh,2ivu,
1s5q,6ct7,3pma,2ay6,5e90,1re1,4l32,3g5k,4bw4,1pig,2xne,4qme,5ugh,1cpi,3wd9
,1o5m,5wfc,4tun,2y77,5laq,1ax2,1b39,3hrb,5dah,5iza,3thb,4bi1,3arf,4gwi,6h1
h,5yzc,5tg6,3vap,4lkh,2q2a,2fle,2vwf,1g2k,2zz2,5xhz,4zim,5fwr,6g9i,6bqa,1g
45,2ao6,3is9,2qmf,3wt7,5vlr,5fnr,6cf6,5z9e,5t1m,3uda,3ur9,3dp4,6c7g,5kmf,4
hev,6d8e,5acx,5ouh,2fde,6c0n,4wt2,6cgp,5zun,5dyt,4cwb,2xcs,4z1k,4b11,2i2c,
5ko5,2ccc,1xgj,6ayt,1ao8,1c5z,1b9v,5toe,4bup,4pox,1gnn,3a4o,5kqg,5am1,1i37
,5uln,3gpo,2g01,3fx6,5nba,1ke7,3my5,4inr,1jp5,5eqe,4muf,5xhr,2wmr,3ebb,4mw
e,5ap4,5nwg,4bde,4x8t,5tbp,1nl6,4oz1,3hvc,1flr,3pz3,4pkv,4drp,4awk,2ww2,4t
w9,5b4k,4o3t,4fev,1o45,4bcj,3upz,1qkb,5nho,2x8d,1aq7,5y21,5i12,3bsc,4wki,1
ouy,4jv9,5o9h,4ez3,3dd8,5h7g,1sqt,3pn4,5aab,2ha4,4enx,1bap,6enm,5za7,3dbs,
4mvw,3vrv,2azc,3mss,3i60,5hld,1zkn,2wxq,4y87,4m8x,5f6v,4fhi,5auv,4l7c,3wcl
,3gqo,5dhh,5u7d,6grp,1xm6,1lpz,2wf3,5cvd,3zmj,1g6s,5eud,3wyl,4wh9,2oq6,2wh
o,2nmz,2xiz,4hbx,6bee,3ryx,5ans,5zvw,4zwz,3q96,4v05,2pyi,5jf2,1wbv,6c7x,4q
sx,5m6u,1m0b,3qg6,3nyn,1rm8,3lhj,3g86,1ny0,3zj8,3o6l,2vb8,3p9l,4bnx,5uch,3
sv6,4ynk,223l,5orr,4o0j,3rxm,3rw9,4po0,4z84,4j21,5yjm,5n25,4bky,5svy,5gmn,
5u7l,1f1j,4aji,2oj9,3oli,4r4o,6ayq,4ql1,3f7h,1skj,2wfj,1wc1,2r3o,3m5a,4lwc
,4j5p,3n46,3ttp,1jjk,5vb7,2pj0,3dog,2bve,5vfm,4l2x,3uvp,4wno,4y38,2c69,6eo
l,2zx9,4q9s,2alv,3ip6,3sio,4xoe,3sn8,5y48,5u7j,2g9u,5f3g,1xh4,4j79,5ghv,2i
2b,1gah,3kr1,5npd,5mks,1jwu,4inh,1tsl,2wq5,6hkj,2yek,1hpo,2e9u,3mwe,6aff,6
e99,5vlp,2uxu,4u5t,2hwo,4ury,5byi,4v04,5e2s,3lj3,4bt9,4jt9,4rce,6b5a,4mk2,
4idv,4bah,5lj1,3nal,6d1m,5kpm,5sxn,5doh,3m6f,3u81,6bsk,5nfa,1gui,5i1q,4m4q
,4ow0,5ur6,5iu7,6d55,1bma,5uxn,1oiu,4ln7,2a5s,5u5k,5e4w,6afg,1oyq,4xtv,6ej
i,2xdl,3at1,1njb,4u0g,1tou,2pj5,4q1a,3avk,6fmj,2lto,6czc,3u8m,2y5l,3fjg,5u
wi,6m9c,5m3b,3jxw,4zqt,5vc6,4bj8,5knx,3pix,3e6k,4f7j,3koo,5n3y,3jdw,4pl3,4
nmo,1ody,5tpc,6bsm,2cm8,5gn7,5hu0,1yq7,2gga,4wy7,2brn,2fmb,5h14,4zsp,3nus,
5n8t,5x5g,5yls,5u3b,4x14,4puk,5wa6,2y2h,4nmt,3uol,4jzf,4um3,2wi2,5k7h,2ccu

,5x74,3ddb,1xd0,4mm9,1ulg,3coz,5epl,4ear,3vzg,4qac,4l5j,1ai6,3d8w,4mi9,2wt
v,5iee,6ge0,4q3r,4qb3,5cdh,2vip,3pz2,3f34,3rin,5n2t,5oqw,3p4w,4n5g,2pvl,1b
0f,5a3u,4p6x,3rsx,3rcj,5i9z,5ai6,3c4f,1o4o,1sje,3alt,5f41,5yid,3bgl,4rdn,4
hso,4ttv,3ok9,5y0g,5twx,1wug,4a6c,3gxt,4lzs,2zx7,3jvr,4as9,3f1a,4cd6,4r06,
5h5s,7gpb,5w0i,4wp7,5e3g,2ydt,3hec,4abb,5ji8,4olc,5ieo,5i2z,4zy5,4qga,3t82
,4dff,2wr8,3i7b,3v4v,4gw8,2hxl,4bkt,2wj2,4xwk,1xk9,2fgh,3tv7,1ajp,1tps,3ag
9,3ls4,5wg3,1cwb,3mea,1bjr,5auz,2y67,1q9m,2i3i,5ivj,2vd1,1p2g,4l7n,5efj,4j
zi,5mt0,5vlk,3rt4,3zmm,2wn9,2a25,4hmq,5jim,4zyy,4bi7,1f3j,2b52,2xkd,1yys,3
vtr,3zrl,4kao,5fnc,1ke5,6hwz,4d0x,4mzs,5mi5,4a6l,4eg5,1sgu,1w11,3dkg,1s19,
4wyo,3ppp,4rlu,2jjk,5ovp,6cd9,3qio,4wrb,5h9p,5na0,1wbt,4ael,5eu1,3nw6,1c5s
,1bl4,4ual,2vsl,5vil,4o7f,4dhr,1pw6,1b5j,3iad,3rwg,5wev,2pjc,3vi2,4xum,2zm
m,3fv3,3ukr,5myo,3lbl,4ppb,1q0b,4abi,6bfx,3kmy,1q6k,5ygd,4z90,4omk,5bqh,5l
rj,1ta6,5jzn,3ad8,5izu,5ief,4frj,2pre,4ty6,5nut,3zps,5e2v,4k43,5e7r,6ftz,3
rjc,4uvc,3uix,5up3,4i31,2p15,1ag9,1gu3,6cw8,5ei8,4psh,4i71,6f9v,4yoz,6hu3,
5e2m,5ap5,4fob,1uod,3e63,3mke,3io7,4whz,4mt9,4x21,1ppi,3t2p,5q0g,5jjs,5icp
,4g1f,1csh,4mwr,5al1,4qxq,3o9c,2wxo,4xmo,4ii9,5mlj,5hm3,4wr7,3iqv,4xjr,4w9
w,5fhm,5o2d,5osk,3bv9,3i7e,4kz4,2oz7,2vzr,4d4d,6eog,1sfi,5ehq,5uy8,4u0d,2l
11,5v2p,2vtm,5ef8,1zgv,4b2l,3g32,4q4e,4x50,2oax,4r8y,4c61,1w2g,5er2,6b2q,3
up7,3r01,5izq,3l6f,3p9m,6ey8,4b74,6fyz,5g1z,3t4h,4wkc,2o4j,4bdj,5mek,4gvd,
5qil,5go2,4m3f,2e2b,4bi2,5l0h,5ugb,2ynd,2wzz,5wyz,5may,4eev,4kiw,2y7i,3tcg
,3s71,2qcd,1vru,1h00,4uat,3nyd,3bkk,4n9d,1bim,5zt1,5ml2,3fpd,6h7o,3p8z,4ew
h,4uva,5a3q,4dai,5o55,3f69,4puz,6f05,3g35,4qij,4dxg,3n1c,4dt2,5u2e,6c7j,4c
j4,2w7x,6ccq,4qsv,5upz,5iu4,4x0u,3ffg,1y6q,5yba,2f35,4gsc,1jt1,4qvl,1mwn,1
jeu,1c3b,3ual,1rdn,6dh1,5iub,5mav,3t83,3iof,6cwf,2fpz,5ml4,5f2f,1fpc,7kme,
1erq,5t9u,1lhv,2qlf,5jmp,4s3f,6hm6,2qt9,4p4i,5vih,3mnu,6djc,5db0,4ca5,5mar
,2w77,2avq,5eqq,1tsi,2vw2,2ggd,6f6d,1f4g,3ndm,3l17,5fni,1c3r,5trf,1pau,3vf
3,6ewk,4re9,4li8,1n4k,4l7o,5kbh,2gj5,2oh4,4wht,1joc,1qxl,6elp,4qge,2yfe,3g
8e,1cea,6czu,2fs8,4mk5,4i60,5h5f,5tcc,4dpy,3fh8,4h1m,2anm,1uom,3oim,6ezi,3
da9,3r17,1l6s,2uzv,4e1e,3d28,4bcd,5kez,1bn4,6biz,4lwt,3jpv,5fsm,4mds,4e6c,
3o9p,2ylo,5fyq,4hxq,3mj1,1zm7,1s63,4nk9,4qmm,3g7l,4zyw,2q8i,3gfe,5ih6,4owo
,5o5f,4x7q,4wy1,1qbq,5du6,1oe0,1g2a,4iie,4x5p,1fhr,1ntv,1o5f,3os3,2cbz,4ar
b,3lxs,3lxg,2g94,4gv1,5n87,1gz9,1b6l,5kcx,2xni,3ig6,1p4u,3r9h,5n2x,6aud,5n
ee,1bnw,6bo6,3kaf,2fvd,2xaj,4a95,5iv2,3n2u,4q6e,1i2s,2f34,6evo,5ksv,2e9n,5
cgc,1m51,1d4h,5ncy,5kls,3s2p,4o09,1zaj,1f0u,4k3l,4tww,5f88,3fcb,4arw,4o9w,
2ay7,5dcz,5dqf,3ttj,3lc3,2xg3,1uml,3qt6,4oty,4wzv,1hxb,5l87,4ah9,5ma7,3npa
,4hs8,4qgd,5zkc,4d2t,5elz,1jk7,5cuh,3jqg,5w4s,4n7m,3tct,5ie1,4n1t,5aqh,5ok
u,5nhf,2yge,4gs9,6bu3,5vqr,2am4,5wr7,4j58,4zsr,1yon,2bkt,4buq,3u7m,3u51,3w
55,2gfa,3fum,4hyu,4pra,4kyk,4gzp,3ekn,3v2w,2pog,2bb7,3qj9,5d3t,2w67,5fs5,5
qaf,4crb,6fe0,3zmq,5t1t,2xm1,4oaz,6e8k,2fys,5egs,4aac,1akr,2wi1,2qbs,5h2u,
1lke,3smq,5eds,3wmb,4azy,3cz1,1s39,1n94,5lc0,5wg5,5ttg,1ndz,6gmx,4cfe,3fsj
,1cim,3kry,2f2h,2g9x,1o47,6aqf,4knn,1t29,4k5z,2ycf,5ijr,5kks,5tg2,4yxu,4i7
c,3ftv,1ke3,4bib,3coy,5orw,5bns,4bs0,3igp,2uw4,4avj,3qcy,1yqj,3a73,4uu8,4h
3f,4rwk,5j5r,2rkm,1w14,4edu,3e73,5o1s,4ngn,1mnc,2ypp,3wq5,4j74,3rz5,5upe,4
eh6,1c84,4fcf,1sc8,3agm,5w10,1gzg,4qvv,4qp6,4why,3ip9,5he0,4yv5,3ril,4uyd,
1nu3,3ozr,5qin,4utv,5x9o,1okv,5m4c,4xhv,5ll7,3cyx,4gm3,2w78,5q1d,3bym,5aqo
,1ga8,1p5e,4c4f,2mps,4cxw,4w9f,3lmk,3sz1,4tya,4qjx,2v85,5bw4,2ael,4pv5,5os
y,4m8h,3i02,6fs1,3twr,2vt3,4rrn,1h5u,2qoa,4lk6,3qtu,3k5d,2qhy,5jmw,3bun,1g
hy,4l70,6cd4,4ymb,6bh2,5h0h,1ppx,3r4p,6gnr,2x6k,3f9n,4oys,3rf5,3hub,4fcq,5
o1i,3ohh,4mqu,4gw5,5m4f,2r9w,5yea,1xp1,5lm4,2csm,5t2g,3n1w,3t8s,5nhz,4zk5,
1e6q,4cg9,4bf1,2ycr,1c50,3hd3,4ehe,5tku,5wle,2r64,5l3j,6ft8,5ti5,4tyt,5lma
,3oct,3zt2,5ai4,3zh8,3fl8,3old,3fqs,4xct,4mjp,3g2y,6g8n,5kkr,1fjs,6dxg,4cx
y,3che,3qfv,1r5v,1pr1,3nsn,4p1u,3szb,5u11,4o0t,3ux0,4q4i,2nww,2qp8,4rsp,2p
0x,1fsg,1u0g,4djx,6cco,3opp,4lg6,2clv,1hps,6bhe,6bir,5h09,3kwj,2bed,3e51,3
msk,6axj,3udd,1zvx,5u6d,4qnb,4eox,2rly,3ob2,3osi,5m7t,5vqy,5ei6,3mbz,4f7n,

3ipb,1w6r,4zx9,6gy5,2hh5,3nmq,1vjy,4hy5,4ts1,2qcg,5lwd,1kjr,3oyn,3rbq,3ujc
,3ekp,3p7c,4urv,3gws,3fw4,1ow8,4pov,4k4j,4m0f,1ow4,3qaq,4ovf,1ps3,4gw6,5a4
c,2ovx,5q19,1gwm,2kwn,4uce,4y2p,2q2c,5lgn,5oxg,3i4b,4qmo,2wyg,5eay,2b4l,2x
d9,3sus,5zu0,3gjt,2b1v,3ctq,5tzz,1h28,4b13,2q15,2aac,3kw9,3qox,4wku,5wqk,2
fjp,5eij,4mga,5ak2,5dp4,6f8u,4inu,4nrc,5drt,1tng,3vhc,1xh3,1toi,2lya,3mpm,
4k55,1qb9,5hlw,6ej2,4i33,4cwp,2fvc,3r02,6ezh,4muw,3d91,3img,4op1,4s3e,3qxp
,4f5y,4qmv,5u62,4qtc,6tim,4h2j,1x7e,1b6h,3opr,4gjb,5u5h,5v3o,5l44,2c6i,4ez
r,5u4e,1fta,4z2j,3n3j,5eg4,2yiq,4jmx,4q2k,2x2r,2fzg,1ajx,2yki,3ccw,5u0e,5j
32,1y2e,6gwr,6gu7,5dxb,1hyo,3lc5,3arw,3rz1,5kr8,5jv2,4xpj,4l2f,3vi5,5etj,2
a3w,3bra,1h79,6ed6,5w85,6mub,5od5,5ejl,3ubx,2c6k,4rn0,3pdq,4unp,5mng,4w5j,
5afn,6h0b,5yc4,1ahx,2jst,1o3h,3rz8,4na9,1f0t,4eym,5vew,4k3p,4zs9,3b9s,5wcl
,4mmm,2l65,4lph,3frz,3rz9,4kba,5a8x,5eps,2wor,5f1j,2oxd,3ldq,4g0k,1u32,5gr
9,4fl3,3uxl,3fud,4x68,3k2f,5lsg,1h23,3zrm,5v6u,4lke,1o4r,5mw4,4fcb,4qp8,1h
tf,4q0k,4yc0,3ebp,3q4b,5dh4,1bqo,3sn7,4waf,3gst,5ecv,1hvj,3m3c,4ocv,2ewb,5
h19,3ryz,3a2o,4ea3,4wx6,1i32,4lge,3cwe,2hmu,1h0r,5m9w,3jwr,2q70,4iu0,1tl3,
2gqn,3bc3,4gk7,2wd3,2x8z,4ybt,5dms,1nhg,4mw1,3cgo,5v4q,4x6n,3bh8,3f6h,4au7
,5i3a,3l6x,5d0j,4c1u,6fzx,3ook,5mt4,4hzx,3udp,4g8o,4mrz,4tyo,4umj,4rfz,1rx
p,2zyn,5t54,6bh3,4cd5,4hlk,3t0x,2az5,5dy5,1ft4,5org,5to8,6eqx,3b2q,6ez9,6a
ox,2y5f,4x7o,4y4v,5oae,1h25,5d2r,2xch,5ndd,2cf8,2yi7,4nzo,3mct,5dia,1iih,3
ime,3tll,2jle,1h26,3l7b,5v88,1o2o,3e90,4j46,3dp9,5ngb,5jh6,3rpy,2vfz,5xn3,
1e6s,4xx4,3ppo,4qfo,1o3d,3s43,6ar2,3cyu,1qf0,1ow6,5eml,4amz,5ye9,4mti,6f29
,5mk1,1qsc,1uvr,2off,4whq,4d08,6gu3,4b6q,4agc,4uu5,3blt,2zb1,1g37,3krd,3lm
1,1noj,1t32,3i6z,5kbq,1gwr,4k6v,2vba,3zrc,4nmx,4mbc,1ie9,3bgz,2k62,1mqg,1q
y1,1mau,2x7u,3iqq,5h8e,2aig,5n9l,2g5p,4fri,4q3u,5flo,4bo1,5tyr,5x26,2drc,4
bbh,3gkz,5e6o,4qy3,1nq0,5e8w,2gu8,1w9v,5uci,2veu,5hvy,3ewu,1jm4,5yas,4ojr,
1h0w,5eou,3as0,4e3g,4euv,2wly,3lir,3znr,3q3t,6ap8,3g3n,3c52,3g2w,4iku,5bry
,4wvu,3neo,1abf,5db2,1sjh,3hc8,2liq,5dtw,5dhu,1qhr,2pl0,4wcu,3exo,1jvp,4mr
d,5jxn,2yjc,2o9r,5en3,5hpm,4qf8,3k05,3h0q,2a4l,4xue,3ewc,1dtt,4yzm,4q4p,3d
9o,3s8x,4mq2,6ek3,5tgc,2hds,5fwj,3kme,1zog,6ce6,4c6v,1xdd,5bvf,1dud,3lzu,4
c1d,2er6,3f48,2xuz,5fto,4zei,3c39,6fqu,4fc0,1bzc,3pj8,2auc,4kp0,2y7p,4ono,
3nc9,4bjb,4qo4,3mz3,5edc,2xzg,6bqj,1ga9,4oq6,4b80,5ee7,1bcu,4y8z,5ka1,5lpd
,3qi4,5vii,5ele,2ew6,1s9v,4bqw,2x6w,5mnc,5eni,2xi7,1wqv,2b8v,6f92,4jve,3oy
0,6h41,4zx4,4q15,6czv,2wti,5j9l,1o5r,5n1v,2bq7,4fic,2ole,3umw,1a1e,1ax0,4u
x9,6bhi,3pj3,5m55,2uw6,2bqv,2ea2,5dl1,6g7f,5o8t,5nze,5fkj,5t1l,4wj7,3ibi,5
h22,4bgh,2j9h,2iyf,5m28,2v0c,6ez6,5b6g,2q3z,4des,3pcj,5y0z,3u6h,3dtc,3cr4,
2igv,4qaa,1yhm,3n35,5ylj,5dls,5h1u,4qw4,4hv3,4jfi,2go4,2m0o,5vzy,2h6t,2qnn
,2aqb,1zd5,3pxf,1p1n,1bnt,2hm1,4z46,5e2n,2p8s,1h2k,3uh2,1b2m,5eiw,4bfy,5cs
x,2z92,2rjp,5o7n,3d3p,4ban,4l0l,3zo2,3zhx,2hah,3dx1,4dkt,3sz9,3n0n,4l4m,6a
sz,3hv4,3imc,3eju,5t6z,4jaz,1gjb,5fl0,3r9n,3sw8,5ggp,1btn,5j8u,1x8t,3bva,2
wtc,5lom,2vwc,6bw2,6m9f,4n1z,3lzb,1fwe,2w16,2i6b,5ytu,4j8r,4je8,3t60,2n9x,
6f8t,4lwu,6ebe,6ajj,5wap,5a6k,2xp7,3ql9,1q7a,5dkn,2gnl,3rwc,5npr,2e9c,1ajv
,6e4f,1o7o,5alf,2xys,6h5w,2jfz,2xdx,4bda,3rxd,1f57,9icd,3zpr,4ciw,2zcr,2w9
r,3hnz,4r1y,3olg,5lce,1kwq,5lrg,5f0c,3u8j,1bl6,3amv,1ej4,4ht0,3u6w,4q09,5j
ss,3qlc,2b1i,5cls,3eyf,6eln,5mrh,3nox,2qe2,3u6j,1qka,3k5u,1ws1,5ew0,6ft9,3
kr4,5iug,1ajn,3zn0,2i3z,1y3x,2r9s,5vpm,4occ,4nym,3osw,3hy5,4j86,4dfg,5f1v,
5l4e,2qve,4erf,1wss,1ajq,5h5r,1ym2,1hee,3rq7,1dxp,1vgc,3huc,5fqb,4mue,1qbs
,1ujk,6fa3,4xo8,4ww8,4qxt,3in3,2wxp,5j3s,2h1h,5d7a,4lhm,2rjs,5nvf,3l0n,5b5
f,4oow,3ej5,5fum,1li6,5swg,6eum,3fas,5klr,2vv9,3v5t,2byr,5nr7,3suf,2xb9,1f
4x,5v86,3kl6,1qng,2a8g,2j62,3zi0,1h6e,3ap7,3zj6,4hp0,1bmk,1nj1,5xp7,4iwd,1
if7,4ia0,3ms7,5yjk,5j7f,5aba,5ake,1h2t,4mzk,4ezj,4an3,5jfp,5v24,2xmy,1yqy,
3aox,4yjn,2i0h,4lwe,4frs,2hwg,3o4l,3ddg,4cu8,2zfs,5wfd,4lnb,4j17,4qyy,2r3j
,4eoi,3pdh,1y3p,4g5y,3v4t,4okp,5meh,1xpc,3avl,6gzl,2pqc,4g0y,3wab,5iop,2ax
9,3bex,3cdb,6afc,1v0m,3ksl,3fvl,6c7d,1t2v,5bot,5yto,2wed,1jn2,4k4e,1a94,3o
zj,1j81,3e93,1q9d,4idt,4k2f,2whp,3snc,4otg,2lty,5bnr,6c7r,3as3,4ge9,4xg7,5

dw1,3ivq,2qrg,2xdk,1gno,2gg5,1d4y,5dqe,3rwi,4x8o,3el9,4kup,3eks,2kff,1w5y,
2flh,2vwv,3rvg,4oeu,3u4o,2pv3,4i0t,3aru,4yv0,5ura,5uv1,3vjc,4bam,5vc5,2ito
,1l5s,3adv,3oe6,5mnb,5gvm,1r1j,2qe5,2rjr,3wiz,4cfm,1gz4,3c1n,6ma3,1z9h,6g9
8,1qm4,4j24,3pju,5cqx,1wvj,2yde,4mqp,2np9,4zyu,3ua9,3fc2,2xp5,1umw,5jer,3d
cv,4aft,4rhu,3ahn,4ytf,5os2,5el9,3gjw,4oth,1lkl,6ela,5vkf,2oz6,4q1f,5usz,2
xfk,3n51,4fxq,4oc3,3ggu,1hiv,4d2s,2fkf,4oz3,6fkz,3lhg,1jyq,3uvw,4f6s,2qk8,
5jat,1lvu,1lgs,4cwt,4gz3,5vkc,1wu1,5m17,1rne,1l2z,3s0d,4nxq,5iu2,1x78,6bc9
,6drx,5juz,4qvn,5i7x,5o7e,4ktc,1fl3,1enu,2clf,4zs0,4llp,4djy,5nrf,4z0k,4k8
a,6cdc,6bin,4e1z,1a61,2q64,3s0o,4z0u,1ii5,4lk7,5ubt,5m1z,1t4j,1g7v,4q1n,3l
08,5d7r,2emt,4y2b,4j0t,2zof,6ecz,4gb9,4jfl,2ow3,1ibg,3fyz,5g5z,7lpr,4g8n,4
bie,2yir,4yps,5jdc,3p4v,2fum,5c4o,1kcs,4r3s,1cj1,6f09,4ce3,4ft2,2pcu,4mo8,
2g2r,4lxk,4utn,1stc,5nxp,5t52,3iod,1h01,4deb,4jjs,5ts0,3su0,4yhf,1y4z,3heg
,2g70,3p2h,1sqq,1swk,2f4b,1igj,3fcq,2pja,4ase,2o63,2xyf,4j0v,5ka7,5kzp,3oo
b,5w6t,1h2u,5a00,4zt3,2cer,1w96,2pzi,4qf7,3c88,4y67,4nrl,4du8,4qtn,3sue,6f
hu,1qs4,5u6b,5edb,2p2h,5gtr,4hbp,3tia,3cbp,2cgw,4bio,3gwt,5t8r,6fo7,1g9t,3
kr0,1gt3,5c83,2r9x,3f8c,5nk7,4cfu,5e2r,2wf1,3uil,5fl6,1ms7,4csj,4yat,4f1q,
4lm1,4oc0,3hj0,2xwe,3ms4,2jh0,3oad,2y2j,4bd3,6h7n,5exm,1ctu,5l3f,2ajd,2c94
,3g3r,2zo3,4dro,1uou,6bsl,1uy7,3zyb,3t0b,4o43,5q0l,3wch,5orb,3kgp,3zeb,4py
y,4b0j,1jq8,3u4w,2xyt,5jhd,3hmp,4dxj,5hg7,3zlx,1zkk,1gny,4ju6,1il3,4daf,4o
0x,5ygi,6gla,3mtw,3mg0,3d9l,2vcj,5edq,4bci,4e4x,2qdt,3wuu,3udv,4dkq,4bfp,5
i2e,3q1x,1qaq,3rm8,4ahu,1gx4,3wi2,4de2,5g2g,2wuu,5nud,4hcu,3k37,6prc,2e7f,
4bb9,1amk,4l1a,4lqy,4a16,5wkf,4qsm,3sxu,3uqg,5lif,4azf,5ntt,3vsw,5fos,1qin
,3e7b,1ll4,3vjm,3ny3,6aqs,1k6c,4ngr,6fc6,2gnj,4zy6,3u8d,2aou,4b0b,5t2p,5ef
b,5u0d,5vjp,5bui,5e13,5f5i,4ivk,7hvp,3d7k,4vgc,5g1p,6b1h,2br8,1cqp,4w50,4e
hv,5kqf,2yfa,3ws9,4xkb,5mrp,3str,5nkd,5mpk,5kqy,4lw1,1avp,3qdd,5xxk,4oks,4
hxr,3ur0,4pns,4i5m,4hgl,2vtd,2gph,3rme,5fqp,5hfu,2iok,4yux,6g1u,2hvx,1n0s,
4l4v,3s1y,1g7p,2rr4,2x00,2b55,4wmv,4kc1,2x39,5joh,2w0p,4az6,1o0f,3haw,5w44
,2xhr,1h8s,4ge1,3phe,1evh,6b30,1g35,2qfo,5gjf,6bt6,2gg3,5trr,5ly3,5eh0,5io
y,1xt8,3c3o,5e74,3acl,5abh,5fol,5ddf,3qxv,3iwy,3ttm,5n18,6beb,5nxw,5lxb,4r
a1,4muk,4mm4,4hs6,5ncq,3zmg,4hcv,4i72,4pee,1qtn,4awg,4rfy,5dyy,3ud8,5itf,5
cuq,5f1c,5hey,2ko7,3oil,5tks,3t3u,5o3q,4k72,4oma,3hv6,6df7,5er4,4no6,5ugd,
1ciz,1rev,6hdq,3c43,1a7t,6fky,5n1r,3rr4,5bqg,3b8r,2pfy,5c7f,5l6j,1etz,3drf
,3k7f,4b6e,6asu,1fd0,4zjc,1y3g,4joh,4c38,4h5c,5kej,6aji,4mvy,3lpi,4pms,6fx
1,3ioi,2xbj,1bnm,4tuh,4fgt,5mhp,4hwb,6eo9,4aje,1v2t,5h85,1x8d,3uoj,2aog,3o
5x,5vr8,2o4p,1fh8,4jz1,5oq4,3zdh,5otz,3lnz,1n9m,3rak,6ba7,6czb,2wyj,4qyo,3
mxd,2ri9,2zq1,3ljo,1kvo,6g2a,1gj6,5oug,1g4o,3mvm,1iyl,2cgx,3v2x,5iv4,4m2u,
4q1b,3ggc,2c68,3ind,1laf,4kxn,2ada,1om1,5eh7,4jk5,4q93,1b8y,1x11,4mr6,4c4j
,4yik,1osv,4y7r,4zow,2hdx,4ui6,4r6x,1pxi,5ni5,5v4b,1h0a,4pf5,5ggz,1o4m,1ur
c,5mqt,3ads,5gn5,4mrg,5ylt,5k6a,4nld,2xl3,4auy,1zoe,4pum,5lxd,5csh,3gbq,5x
s2,4ggl,5a14,4au8,5n49,5x8i,6c7f,3fvk,4kqp,5y97,6gub,5mxx,3lox,2oc7,3g1m,6
hsz,1ilq,3twj,5vcx,3svv,4na4,3v5g,4p7e,2vwl,4umu,3v4j,6gfz,6duf,5aqf,5ezh,
5nar,4yti,4zyq,4lop,4nus,2a3a,2o9i,3ia6,5nfh,1e00,5u8a,3n8n,4axd,3kxz,3owd
,4fxj,1gx0,4u90,1d7x,3eq8,3q44,4ago,5e2p,1pxj,4p6c,1zoh,4i7j,4hlg,5j6n,3s2
o,4ec0,3wiy,4k77,5yf1,1hsl,4tjz,6cbh,6g38,4efu,5wep,1jwm,3wfg,5lvl,5kqd,1f
q5,5nn6,3b5r,5xag,3w69,3udr,4c4h,5mgi,5ad1,5zh3,4i7b,6cwi,1tkb,3unj,4u54,2
x0y,3isw,2xbw,6fzu,5wgq,4kzc,1yhs,5btr,1juq,2idk,2v25,4prh,5o5a,3r0h,1sle,
2ieh,5ous,2fqy,1p7m,3fuf,1w10,1vot,1guw,1gbq,5thn,6b41,4xoc,5ugc,4h71,5i43
,4oc1,3t0d,4kw6,4jrg,4ra5,4azp,5sz3,5hbj,1npa,4wi1,4b7p,3biz,4bpj,3lxe,5qa
v,4ge6,4k1e,3vje,4mic,4qp9,2xg5,1w5x,2rin,3omc,5ii2,3pax,5elq,3bpc,1ec0,4x
8g,3cft,4qd6,5n1x,2v96,4ucc,5ocj,4mul,3lok,2e5y,3eql,5kyj,1n5r,3hfz,2pg2,3
g3m,4fs3,1nnk,4n9a,4jp9,2j77,6cvd,4tkb,3tfp,5a5o,5nsx,6bbv,5myg,3ws8,6ex1,
2jkr,5kcv,2vw5,4ezl,3dp0,5hyq,5iyy,2oc4,5div,1gag,2r38,3chp,3ot8,2wqb,6bqh
,3gxl,2pgj,6gfx,2i3v,5yqw,5n9t,6cc9,3p3s,3oe9,2y1o,2o4r,1dzm,3vp2,3djo,4oz
2,1p6d,5mih,4xt2,3tf7,5v19,6g9k,4ipf,3bim,4nwm,2aeb,3blu,3vtc,3nww,3mp6,1n

u8,3wzk,4zec,3gvb,4bt4,2f6v,5xwr,5m53,4yvc,1ai4,1z1r,1ryh,1rhu,5d1s,3g0i,1
gpk,4clb,5cte,4kmu,6f9r,3pjg,1uz1,3tww,2lbv,5w1w,1s26,3w9r,4yes,3sl8,5km0,
2j79,2h03,1qnh,4cpx,5enk,4mnv,3r5m,4tk4,5wg6,5uwf,4cjp,3uu1,4bxu,3h30,4qwr
,2gyi,1cnw,4fzc,6gdg,4e5i,3oq5,3wig,5my8,4aj1,3b65,2e95,4xcb,3wf5,5h15,5ht
b,3t3e,3h5b,1ugp,5anq,6g4n,1apb,2ai7,4xxs,1lrh,1ove,3v7t,1xk5,4fiv,4mcv,1m
rw,4b14,5isl,5cj6,5un9,3nil,1x07,6fni,4u2y,4hbv,6h2t,4q6d,3b8z,2cnf,4isu,5
u2f,2cng,4q90,4l0b,1z6s,6g3q,4ylu,3w5t,4dei,2zjv,2x6y,3ikg,5j7p,4mcc,3vqs,
3u9n,2g5t,3owj,5bnm,3hkn,3qtx,3qzt,5bpa,3p17,6fag,5nfb,1o43,1nok,3vby,6as6
,5vjn,3skc,3bg8,1tc1,5orh,5bq0,5qac,3l16,5kw2,3uj9,6byk,5kgw,4wvt,1bv9,4ru
z,1fzq,1mu8,1srg,4y73,3upv,4jj8,5xvf,1xka,4ewn,4ewr,4a7b,2xab,6g9b,4u69,1q
b6,3c6t,3qar,4k5o,2gnf,1u3q,3g31,3oev,4oc2,1eby,3uef,4k0o,5ehi,5h63,3wcg,6
hvi,1hii,4jhq,3sgt,4aua,4hnc,3bxh,5g5v,4j08,4anu,3hac,2vcq,2f7p,4ycl,3s22,
3obq,1o9k,4xy2,3k9x,1xog,5b0x,4m5g,4czs,5eyr,4lm5,5xup,4rao,3qcl,3lkj,2xno
,1hfs,1gjd,4bzs,4j7d,1fw0,1jfh,2cgr,4pd8,3mz6,5obj,3gm0,4j3u,1uvt,5ml5,4fu
t,4r3c,4kfp,4jck,5xhs,5mwz,3n86,5qab,5zeq,1fo3,4eki,5n8w,2exm,4klb,5ikb,2q
hc,5aqr,4qn7,5nbw,4k2g,3qck,3zo1,4knx,3m6r,6dh6,3p8n,5f3e,2vie,4uyh,5uoy,4
gr3,6cyd,2f14,5v2q,3h2m,3ao2,1k1n,3gfw,4q19,2b1g,4pdk,1oxn,2pj1,4o3a,2y6s,
1inh,4u5l,4esg,4mg6,4de5,3wke,6dgy,4mp7,4de1,4z6i,2r4b,5q0z,4b33,2koh,4qyh
,5y94,6fo8,3p4r,4mny,2vjx,5lj0,5yqx,5c26,4eb8,3lk1,3mg6,4oru,2wpb,4k7i,5fs
y,3ava,2z7r,3ex6,4b8p,3l13,5fbi,3s3k,3chc,2wf0,4hgt,6b5i,4ayw,3gk4,2lcs,5f
as,1lyx,6bvh,6edr,1ghz,1dg9,1egh,2x6f,5o48,4avg,3znc,1czq,5f39,2bpy,2vc9,5
op5,4ory,3hu3,1xuc,3hmo,2ybt,1v1k,5o22,3qt7,4eo8,4bco,2p3i,3hii,2xdm,6axq,
2xhs,5cyv,1sre,2y8o,9lpr,4l3o,1dzk,3lnj,3fsm,4ghi,2nqi,1i48,5mo2,6fr2,1bnn
,2jbl,1r17,4rrf,5jah,6apw,4trc,1lgt,2wl0,4i0z,3oe5,3fjz,2uyn,3hxd,6g3o,2nq
g,3fc8,2zvj,3mw1,2c5n,3poz,5ax9,3mhw,5tdw,5n7b,4kij,5axq,3pxy,2q96,3kjd,5c
f5,5id0,5e0j,4kjv,4e1m,3gy4,5m34,1xqc,2hzy,5brn,2d0k,3s8n,2j7f,5zc5,3p5o,1
xh5,2vj7,4zt2,2b7a,1thl,1jmq,5h7h,5mz3,3dxj,5q1g,6ays,3w2t,1bkm,3pb3,3vjl,
2a8h,4pax,5tt8,2y2p,5lyx,1zaf,3mb6,2fuu,4mw6,2j6m,4rll,3qai,4kzq,2uym,5v84
,3bm9,4egk,2bqw,2q8y,4pji,6ary,4daw,4i06,2wxk,1r5n,4lp9,1me7,2zv9,2qo8,1cw
2,3k5c,2o65,4kqq,3rdv,1d4w,1q4l,4b5w,4bgg,4mm5,3iej,3ftu,830c,2xye,1olu,2w
k2,4pxf,5o0j,1my2,5czm,4jit,5mb1,1sqp,3zlw,4xqu,3hkq,6fns,5e0l,2p8o,4gzw,3
n87,1lhc,4itj,4m7c,4olh,4q1e,5l7e,3faa,5vqx,3pka,5x54,5a9u,4n9e,4est,1il9,
4igr,3t2t,6dar,3gol,3vbg,2ydk,4zpf,5zo7,4xnw,1fpy,2r1y,6m8w,2jds,5icx,1hwr
,6bj2,4b4m,1zsb,4do3,3t3i,1f8a,2ke1,5ezx,3p78,4rvm,3ovn,5wzv,4udb,1okz,1mp
l,5npc,5ff6,1hlf,1nvq,4bhf,4y4g,5mkz,2o0u,3bcs,1wvc,4fsl,3oz1,6dgt,1me8,2p
uy,4odp,1hpx,4nrq,1z2b,3uik,3mfv,3vqh,4w9g,4xek,4jok,2wap,1g50,4j0p,2o9a,3
m94,4i1c,5a82,4i9h,1k1i,4uro,2f7i,5fpk,2lgf,4l7f,1g3d,4ir5,3mta,3jzg,5f94,
4nrt,4yax,5nhv,2xtk,4qh7,1tok,4b6p,3rg2,3q8d,3obu,4awj,3daj,2j50,5l2z,5bml
,2bba,5n34,2xvn,1dpu,5fnt,1jyc,4zz1,6hm7,4rrv,4rww,5orv,3qo2,3uii,6d1x,3ju
q,4qk4,6mr5,5hjc,2p4s,2hnc,1k4g,4g0c,2y5g,4u3f,3tv5,1i3z,4mw7,3n2c,6cvw,3v
66,3wzp,3s7m,5ujv,1p06,3ipy,4wkt,4ie0,5fot,5i59,5za9,4gii,4h2o,4yrs,5a6h,2
xo8,4e3n,4m5k,3dga,6fse,6ck6,1sqc,4x1r,3dnj,3rvi,2a58,4bf6,3zlk,4mbj,4tpm,
4d8c,1ejn,4yt6,2x7x,4qp1,4de3,5yg4,1x7b,5n9s,2fme,1ydt,2bdf,6baw,6fsd,2xn3
,4tk0,3q4j,1u9l,1oqp,5htz,4glr,5kj0,5ukl,3fun,4wk2,4ht6,5hv1,1uze,4bcc,3ff
6,5if6,1tsm,2r59,3iqh,2v7a,5d10,5nvh,3eqr,1jq9,1u1b,6cer,5uq9,1u3s,5icy,3e
xh,2oqs,1pzp,1d4i,4x6p,4mb9,5emk,1iky,6b7f,3chq,3h5s,5zmq,4ib5,2wej,6fjm,5
ewa,2igx,2z78,5lpm,4wet,3lxl,2xba,5wbl,5zla,2x6x,4mw9,5t2d,4j3m,4aqh,3lbk,
4djp,4odl,4x6j,1ero,5f3t,4k3q,5ta4,1caq,2eg7,1f73,3rxg,6ezq,1qkt,5l3e,5c28
,4pp9,4bgk,3iaf,5vrp,5zz4,5ur5,3ft2,5ech,4jjq,5iz6,5dhr,4l2g,4r17,3wk6,4h1
e,2aq9,5g1n,3zm9,5c4l,5mfs,1fzj,2ltw,4x7i,4c94,2cfg,2va5,3vb6,2hob,5ah2,5s
yn,3g6g,3rwj,5sz4,4f9v,5n2d,3n9r,5ldo,3vb7,1sqo,3drg,5j9y,6b96,4yz9,1vcj,5
epr,4tx6,3dz6,3czv,5v49,1ahy,3wzq,1bq4,5u8c,6bj3,2qnb,4a9m,3d4f,5oui,5wmg,
6ma4,4x5q,5cbr,6msy,5avi,1g3b,2wi4,3kjn,4dhn,4o7e,5kit,5y5t,3hfj,2qd8,5vsj
,2y2i,5m0m,3tcp,4bhz,1jd6,5idn,4zzx,4kn4,2a5c,6hly,1au2,4jbo,5cgj,3ske,3lq

2,4pxm,2wxg,5tb6,2vc7,3iw4,5hct,3skf,5lyy,3fmz,4p5z,5ktw,6e4w,1cx9,6em7,4m
jr,4u7t,3rde,4ux4,4i6f,3l3x,4ie6,4j70,1jd0,4iaw,1szm,2afw,3ess,3sap,1olx,1
bzh,5hfb,4x3h,5we9,3zsw,5ny6,1hn2,3l3z,4qp2,1d4p,4xkc,2is0,6c7e,5zku,4fai,
6g9a,4xu3,5dry,4d8z,3zcz,3kbz,2y59,4nal,4rpv,4yje,3vf8,4bqx,4z9l,4ep2,4ylk
,5mme,4dht,2uy4,6mu3,3kx1,5o0s,4bch,5c4k,2br1,4ddh,2f9k,2w2i,4ogn,4up5,5o4
y,5hjd,2qw1,5y8z,4kqr,1o2t,6e05,3u7l,2mip,3hvg,2p59,4d3h,4pl5,3tzd,2vnp,4e
3m,3vgc,5bqi,1b7h,1lhu,3rlr,3h22,2wnc,2wot,5d1t,3mo0,4wn5,3p3u,1nfs,4e90,5
aqu,1bmq,3kwz,6f6n,4rj5,4omd,6min,1ujj,4ppa,4uxl,5y3n,6df2,4wvl,1xt3,5oaj,
4a9r,5mli,4p4e,3juo,1z9g,2ykc,5a0e,3g0w,5t9w,1sqa,3wci,1fkw,5u4g,4mfe,4kpx
,3nti,3azb,2xog,3c3r,2buc,1hyz,4dcd,6azl,3t3d,3q4l,4few,1q95,4u0b,3b7u,4bo
4,4o10,5wmt,5v9t,5aok,1jtq,5uit,2vgc,2gfd,3mna,1aqc,4xtt,4z0d,4ty9,2yiv,2h
rp,4zh2,2z4o,1qku,2xdw,4n7j,4yp1,3exf,4c6z,6ccu,2wxn,1bwb,2gvf,1hiy,5c4t,2
za5,2xkf,4q18,1o2p,5th2,4dj7,3eyd,4j0r,2m3o,2b53,4m3b,2izl,2vtr,2x6d,2i0a,
5ehg,6cw4,4c37,3cwj,1azm,2qci,5sz0,2gkl,2z4z,6awo,1v11,4l53,3p55,2ynn,2vu3
,4dli,2bcd,4l0s,4uda,3m37,5j5t,2p16,4gh6,1mfg,3s3i,4j73,2v5x,2h4n,4jsz,4wk
1,4igt,4k63,3qqk,16pk,5aom,1hyv,5a3w,3veh,3g4l,2ph8,5mkx,5c4u,4gto,3cj5,4p
rj,2vd7,5duc,3odi,6bg5,1qwu,5jn8,1v1m,1qpe,5v3r,2wc4,2vte,1a52,4dhq,2qta,6
ccy,4jog,4bgy,5u9i,3az9,1gt1,2jew,3pdc,1n3i,5fyx,4f49,4nzn,6hm2,4a4l,5xij,
5vk0,4xsx,2aj8,4odq,2n7b,4ygf,2a4q,2jc0,4jsa,1inq,3dc3,5tob,4urn,6bik,4ju4
,5nya,5oh2,5znr,5ct2,3u4u,4x7h,3max,3rbm,3krj,1aj6,1pmv,5n0e,4nhy,4oem,6fi
4,4e3j,1fq4,5myr,2hkf,1os0,3rqg,4ivc,5c7b,3lq4,1u6q,1qxz,1l5r,4xxh,3m40,5o
r9,4okg,4d89,2gm9,5x33,4de0,4gr8,5lz8,1p93,2brp,2gg8,6fdt,5cxh,1jvu,3wp1,1
fzm,5cxa,2gbg,2g78,5aml,2y34,2qnp,1v16,1njj,2a5u,4z88,4wmx,5vo2,4fod,2pou,
3jsw,2ow2,5g3m,3odl,3o9e,3eyh,4ej2,3c4e,4b6f,1pl0,3pb8,6fap,4iax,2bua,6fgg
,2o4h,4uwh,5wbf,2yxj,1ff1,2giu,1qbt,2ovq,4bak,2y3p,2iwu,3hvi,2w0x,3fcl,1zp
a,5czb,3t1l,2cfd,3k3g,4cfw,2e91,5op8,3hig,6h7y,3mtb,4eb9,4lkg,5ehv,5ier,4o
de,1xoq,5d6p,3kwa,5np8,5v82,6ma1,3bz3,3myq,4j0s,4f4p,4lh6,1uef,4j3d,4yx4,4
amx,4ptg,2c97,4ec4,4r1v,1zc9,4nuf,3g2u,6hlx,5vij,2x4o,6hlz,4lkj,3s75,2gz8,
1gvk,2yhd,3hqz,3pb7,1thr,4ris,5twh,4gql,3n3l,3acx,5yvx,3gy2,1xmu,5l6p,5l8n
,4msn,4rz1,3f66,3ucj,5hcl,1t1r,3kce,3u15,1wbg,5khi,3er5,4qew,5mft,6eqp,5gs
w,2qd7,4cli,3f9w,3msc,1jgl,3kid,1ymx,1ui0,3d1f,1pxl,5kos,3vzd,5fcz,3ara,4l
i6,5ks7,4wym,5j7q,4qsh,2ce9,5vqz,3o2m,4bcm,5orx,1i41,3c5u,4kai,6gjy,4tsz,5
o0e,6drt,1y57,3kqb,3jup,5ork,3ikc,3gwu,4wke,4x7l,3lp1,5ivy,3f16,4c36,1w2x,
2d06,1hbj,1ols,1iup,5aix,1ydd,5w4r,3h23,3rj7,4ish,1ebw,1fcy,1d09,5hdv,4x1n
,5boj,2xn7,4b6s,3f82,4clj,4zzz,5j5d,2vts,1k08,3u3f,4jk6,4csy,6hth,2mnz,2vp
g,2qd6,4jkw,3ml5,1ih0,4at5,5dgu,4g31,5n0d,5aa9,4u4s,5oa6,2wzm,4b4q,6fi1,6c
hn,1z4u,5aa8,1lpk,3cib,5d75,5x4o,1ydb,5dhq,5t28,4zz0,3evf,5vyy,6eip,1q63,3
ldw,5tq4,5uxf,2j7x,4kil,1yda,3bc4,2ew5,6ee3,4yrr,3wax,3bzf,5ody,1k06,4j84,
5l6h,5eok,5nne,5m6m,2a4r,3p1d,2ayp,3iux,4b0g,1jr1,4qo9,4bh4,4xt9,2ok1,2r7g
,4uib,5mmn,5akj,3hs4,5wpb,6e5x,5vnd,5evd,5wlg,5l4m,4kiu,4own,5oh9,6arv,1xr
9,4hv7

List S4. List of PDB IDs included in the training data of Uni-Mol Docking v.2.

2r2e,2y6o,2wca,2dua,2uw9,2f81,2trs,2avq,2pwv,2am9,2fvx,2uza,2v04,2vqj,2d1o
,2bhp,2weg,2ehq,2os9,2r68,2wge,2ype,2xnj,2c31,2cdb,2jhl,2zyt,2ouq,2dxp,2eu
8,2pd5,2br1,2wzb,2piy,2xcv,2uus,2cpp,2fsi,2uzu,2hn7,2dvb,2cfz,2ewa,2am3,2j
j2,2zdl,2dhc,2pj7,2p2h,2fdy,2pk4,2vcj,2ckq,2bz1,2wev,2qs1,2ea1,2w6o,2e91,2
zxn,2o05,2bxa,2e0n,2vt3,2pzm,2ay3,2bkl,2jdz,2wd7,2ztj,2bt0,2g5c,2skd,2a5t,
2al7,2im1,2eal,2z24,2wew,2cdp,2h2q,2hgt,2bgi,2qwq,2yek,2fdx,2h4l,2pgt,2bfq
,2pyu,2osm,2ohr,2xvi,2yvk,2b56,2p2v,2wkk,2tdt,2brh,2vg8,2f2t,2ykc,2zy1,2zv
2,2q0g,2zgj,2aqu,2rl2,2wi4,2vd9,2x95,2xcw,2pgj,2h8x,2brk,2j7f,2qd2,2xbu,2z
x3,2ow1,2orw,2jdr,2ydi,2aaz,2wbk,2evm,2j73,2r46,2afu,2ao2,2pdk,2po3,2fqx,2
hx4,2x7e,2imp,2glr,2ycs,2a4l,2prj,2fjd,2vci,2vd1,2ar8,2zfx,2pzv,2hfu,2p1m,
2qft,2qbt,2cmb,2f3f,2zhl,2zm1,2g9x,2ipg,2c5o,2fdv,2ho2,2bdy,2vnm,2ipj,2daa
,2zuk,2du8,2gc0,2c6z,2c7g,2bo4,2hb1,2c43,2drc,2wsi,2ybp,2f8p,2izh,2d5a,2vt
m,2rcu,2o5l,2vtr,2jai,2jfo,2q0f,2xp7,2ycn,2cjf,2eqa,2ixc,2px6,2dc1,2fge,2g
28,2yck,2x2g,2hc4,2i3g,2bxk,2ivs,2c8m,2uvh,2r85,2wr8,2y0j,2xdo,2f2u,2w71,2
nmy,2gmv,2aa9,2cm7,2rto,2bq7,2wxf,2inx,2vpo,2gg3,2v61,2is7,2ze6,2wex,2i04,
2ypi,2ych,2gm9,2jev,2yqj,2wz0,2rk7,2gn2,2pl3,2yfz,2cht,2xj1,2j34,2qwo,2ym3
,2h12,2vl4,2pht,2a69,2xe8,2y80,2tys,2p15,2vjy,2v7a,2hoz,2a9z,2vjm,2bf7,2dg
9,2yi1,2e8w,2qhu,2rj5,2gqw,2r0u,2wcu,2xvj,2nm2,2p09,2fze,2cju,2pjb,2g9z,2j
8r,2bgm,2o2d,2qa1,2yng,2v5x,2wsa,2ga4,2ydd,2bv3,2ext,2p1n,2yym,2z8p,2bgu,2
z94,2bkk,2a1e,2ezu,2lig,2j9h,2weq,2j07,2ahg,2yxn,2zat,2h8h,2ces,2pv7,2x97,
2byr,2yx7,2v7u,2ivu,2c29,2itn,2vlf,2bhi,2am5,2v57,2rgd,2pfy,2aqh,2a3i,2vkm
,2y5n,2j7e,2ykb,2gu4,2hek,2ym8,2pio,2j7p,2aoe,2j78,2gv8,2qwk,2aad,2bge,2h5
z,2cc6,2jhd,2h4n,2w7m,2wkv,2pz5,2azc,2qlb,2v2h,2gf3,2qhm,2bmk,2wda,2yne,2y
oh,2f2k,2yft,2ql5,2hdr,2e6d,2gqs,2q89,2r0g,2zl7,2iya,2q1s,2ncd,2c99,2iog,2
reg,2ga2,2zdz,2wr3,2qb2,2bhy,2vqy,2q9c,2wmj,2jap,2obf,2geh,2wcl,2fhb,2qjy,
2fv9,2gu6,2qi5,2zqn,2a1l,2bqv,2gte,2y4n,2pov,2q8e,2hcj,2brg,2fr5,2vl8,2a4w
,2rk8,2r5c,2wnv,2w7g,2zws,2yk7,2b1i,2hrp,2fpm,2c42,2c2a,2gg6,2i4w,2hmt,2rc
h,2dw7,2fpk,2cbo,2udp,2uy0,2foy,2tmn,2cmk,2b08,2za0,2ev1,2qfv,2oht,2q3d,2i
bu,2ovr,2fja,2d5c,2vk8,2xxt,2b4g,2y76,2hcr,2nt9,2yi0,2f5t,2chb,2bgc,2gcn,2
gab,2c9e,2q96,2y4k,2v52,2d7f,2zlc,2h3h,2vnj,2aec,2j0b,2uw6,2q4x,2b7d,2xa4,
2jb2,2hvc,2xk9,2zqo,2p1q,2dpy,2wm4,2o4n,2ycj,2afw,2dkc,2rhu,2ath,2vcg,2o0j
,2c8a,2pk6,2e8x,2b3b,2vs5,2oh7,2h8g,2nv9,2x8e,2q6u,2ze3,2vft,2oga,2il2,2h3
w,2wds,2ou0,2wzw,2xfo,2yoj,2qtr,2q6b,2c81,2qcf,2q7h,2xxx,2wti,2v0k,2oid,2p
98,2r4e,2hdk,2ghl,2b8v,2rby,2f6t,2bla,2p8c,2yd6,2zda,2ouz,2q54,2ybk,2uv7,2
j6u,2fmz,2ans,2hgz,2z26,2x1l,2rc5,2a6h,2nxg,2bos,2w2v,2x4r,2j31,2w5a,2axr,
2by2,2yi5,2woe,2hs2,2ynf,2bet,2qrz,2v26,2cdu,2c6k,2bpy,2r9x,2w2t,2gx5,2fnt
,2gqt,2f9w,2zi6,2q8g,2ael,2iw3,2xoe,2ax8,2abj,2ble,2ahr,2zka,2c8h,2g21,2d0
h,2zsc,2vag,2xzk,2d52,2g9u,2gh5,2blv,2ob2,2c80,2ww5,2x67,2fns,2wmk,2p16,2o
i6,2a19,2cje,2dtx,2xp4,2zex,2w02,2ajz,2wqo,2vaa,2brm,2zvj,2dbq,2vyp,2qpm,2
pkr,2vnt,2j60,2g5n,2anq,2qrk,2ybt,2cet,2i6b,2vel,2j9d,2vwa,2eak,2img,2a9g,
2oyk,2ca8,2w5i,2r3j,2b9h,2wgu,2xpc,2arc,2rcn,2gpu,2pvk,2ioc,2hve,2w4i,2ptz
,2pou,2xpd,2o5o,2wi1,2oi5,2r4v,2gqr,2wkq,2qd3,2hv8,2zia,2a3c,2y7g,2j3l,2a0
y,2yke,2veg,2hhn,2awh,2ggb,2zf0,2qoq,2v6k,2zx7,2pll,2ggc,2gmu,2r4j,2v1x,2f
xl,2nru,2r4t,2vjc,2h04,2r0p,2cun,2wjj,2igx,2zhd,2aix,2wzh,2pqb,2xwe,2g9q,2
g9r,2qwn,2zw5,2xvm,2q95,2oic,2h6n,2cn3,2v5u,2rj0,2gnh,2bfn,2y68,2c5n,2f92,
2fr0,2wab,2zif,2dhf,2hzq,2zj4,2voh,2yiw,2fc2,2jdh,2q3m,2vyq,2xcm,2r3w,2no6
,2rbp,2qi0,2f47,2dpx,2oem,2we2,2jfv,2ri7,2e3r,2omg,2x8h,2gg8,2ztm,2web,2il
t,2g3f,2hi4,2quz,2weo,2gue,2we1,2x6o,2ev9,2wu6,2iys,2bat,2gj5,2bnf,2wou,2p
3u,2ak5,2jhf,2zkn,2xk2,2uvx,2an9,2uzn,2dvd,2gvc,2r45,2y1g,2c1q,2wao,2w62,2
wzi,2xjj,2a3b,2i2b,2w5p,2xya,2ve3,2wzv,2vdj,2om1,2f3e,2jkm,2d1j,2w9g,2yr6,
2qtv,2jkn,2vd0,2av1,2dvn,2w2x,2iyl,2whp,2ev2,2v8k,2fzz,2j6h,2hxw,2yem,2uwo
,2vkf,2v0z,2we3,2a5j,2fzw,2aa3,2fle,2ixb,2fgl,2jxr,2zm8,2prg,2ag9,2viz,2or

v,2xmi,2o4s,2q3e,2uw3,2x1z,2v9x,2pty,2qch,2p3l,2yec,2r4f,2ht8,2vp2,2v92,2w
0j,2q4w,2vce,2prl,2qv6,2hxu,2qwf,2bbf,2c58,2yki,2rgx,2oyh,2pj6,2w58,2r5a,2
qbm,2wkz,2v2x,2gh4,2h7r,2j4g,2fkw,2cst,2p39,2by3,2gmh,2oxn,227l,2vs3,2fhj,
2z7x,2apg,2gr0,2pak,2yk2,2qzs,2ioy,2j47,2c5g,2wut,2pcp,2w6q,2zd1,2h21,2vhc
,2xzg,2q21,2jaq,2ayr,2ygp,2axf,2zfm,2nwg,2bi8,2c9c,2zk6,2ziz,2c70,2yip,2az
5,2xz7,2cgj,2q0l,2bue,2y71,2iz1,2ykj,2zid,2yer,2imb,2xfw,2fm5,2ojf,2bal,2g
37,2y67,2ygm,2a85,2o5m,2dvo,2aa6,2b50,2rbk,2v4l,2xtm,2bqp,2ai7,2ylg,2ew1,2
ztl,2xt3,2bxy,2ykx,2v86,2f9m,2vuc,2qhy,2qk8,2f98,2yyj,2x68,2ag8,2bry,2i4g,
2wi5,2uwe,2qwp,2cli,2oph,2x0w,2hxm,2j9f,2pvm,2as4,2xby,2ixl,2ihz,2y1t,2yn8
,2oh0,2qbr,2v83,2e1w,2cjp,2e77,2z78,2cer,2h29,2c8z,2izg,2xnn,2gz7,2qqe,2vz
0,2i8u,2w39,2zx6,2qvu,2q0r,2b58,2wjg,2r8o,2bln,2xjd,2pvj,2wkp,2b3v,2axi,2q
zy,2w7j,2isv,2hjr,2hrl,2xvf,2x75,2tmk,2ihu,2ylr,2x9n,2p7z,2beu,2bra,2h59,2
z7i,2am1,2zi7,2axg,2obs,2mbr,2yg3,2y77,2giq,2gvg,2gwc,2psu,2wwc,2q2v,2fy4,
2ye8,2p85,2nu8,2zuv,2hs6,2q8y,2cjg,2peh,2can,2v89,2d0f,2ieb,2hbl,2vfg,2v9m
,2r8q,2v2c,2e9l,2y41,2gsd,2vte,2zhn,2adu,2y24,2y88,2rc1,2roy,2caq,2q0u,2ox
d,2gmp,2wc1,2ntj,2ztv,2pzi,2vg3,2qzt,2j7b,2xh2,2mpr,2uyt,2bre,2v2q,2vb8,2d
3n,2az8,2jfq,2b4r,2iwx,2j5v,2xmp,2f1j,2anj,2bka,2ves,2h4z,2fu8,2fe4,2ji9,2
w7l,2rtg,2efc,2c5y,2bdg,2c90,2g8t,2e1a,2gdr,2jc1,2ilp,2c05,2yvg,2ack,2v7p,
2pk5,2ci5,2y0p,2xpk,2vti,2noa,2zhm,2oi2,2a1u,2wtx,2w41,2jac,2f5v,2vmw,2htq
,2qbs,2bxi,2vkv,2b1q,2wl0,2e40,2xan,2izk,2rfy,2b5s,2jjk,2ea4,2wnj,2hg8,2fw
i,2ffr,2chm,2ouy,2p2q,2of0,2bwa,2x4t,2xch,2y99,2f6v,2xyq,2g22,2am4,2clf,2c
lq,2c5s,2ejt,2xrs,2a4f,2wo9,2zyj,2i7n,2qpl,2vpn,2wqf,2yis,2zgx,2gcp,2ffq,2
q8h,2fl6,2rkn,2xua,2pwu,2baj,2vhq,2bl9,2rj9,2w7f,2z2a,2aer,2v72,2vvv,2rhr,
2r97,2brs,2r8p,2j1s,2y8e,2yvf,2b4t,2bsl,2hu4,2fhf,2iho,2pj9,2qiq,2yee,2qwm
,2ccr,2xcg,2rbx,2fxf,2d4n,2bzs,2d3m,2a5s,2dtw,2agd,2qun,2ipf,2xdl,2iyr,2d0
6,2z7s,2qhz,2jh6,2vwg,2gsp,2p0c,2pt9,2r87,2vrb,2no0,2z1w,2e98,2ntp,2zce,2p
3k,2fax,2wqn,2wyf,2fz9,2zq2,2v0j,2xk6,2bei,2z98,2qe4,2clv,2prz,2znu,2rg0,2
aky,2wmw,2deb,2wuz,2evd,2qia,2e9n,2j3m,2x4q,2c5u,2hz0,2zbl,2c96,2raz,2vmt,
2qs2,2ox0,2xx8,2xzj,2p4i,2vot,2y6c,2c7x,2wqq,2pu2,2e3o,2xhm,2i5c,2vbf,2hjg
,2c9o,2xbl,2ydp,2gzl,2efp,2w8w,2ea2,2iko,2wk1,2i74,2wi9,2vzr,2qxf,2yn4,2aj
y,2i29,2f2g,2hl1,2y2f,2i0k,2qfq,2de3,2no1,2xmy,2xg9,2br9,2piz,2xiy,2ay7,2i
7c,2uuk,2q7g,2hy0,2ay6,2xx5,2xyu,2q4b,2wdo,2nxh,2hdu,2xlt,2wnf,2wt9,2zx2,2
yog,2oys,2vf3,2avv,2bts,2vny,2bnr,2vk4,2pkn,2z5y,2vp3,2zdt,2wlg,2bv4,2vpe,
2aqj,2pkk,2zsh,2hxt,2vvq,2hnv,2ff7,2c1b,2j5s,2ygo,2ki5,2yiz,2nx3,2y66,2p72
,2q3o,2qcd,2bve,2pvq,2aba,2zzx,2vnv,2i4i,2z3i,2dte,2vwl,2bbb,2o64,2v32,2qf
s,2i3i,2gnk,2fdw,2qk5,2gvv,2ce2,2wg9,2yim,2rbz,2wei,2doo,2vwu,2v8l,2vnp,2j
8t,2pwr,2x8i,2hx2,2put,2y0c,2wf4,2fvm,2rhq,225l,2g5v,2pfz,2djh,2hb7,2okj,2
xb8,2uv4,2vmd,2ymd,2d1r,2anz,2h9j,2af9,2xgz,2i4x,2yp5,2bwn,2xjc,2qlu,2bgr,
2q9s,2wpx,2rfz,2a2c,2ead,2byc,2jg1,2q27,2jlr,2an3,2p4q,2vj9,2ifr,2f9l,2wxd
,2x1r,2g5l,2yp0,2yq9,2j30,2dg3,2exk,2vh9,2vo3,2rtn,2rkg,2fli,2z50,2jav,2w0
d,2wbb,2q36,2ate,2ha6,2xfj,2q9f,2x0h,2c3q,2wxl,2e41,2v3e,2jjb,2fah,2fap,2f
nu,2r3f,2x3j,2phu,2pd9,2nvc,2q7d,2ya5,2cfi,2ab6,2rf2,2cbt,2e5y,2b8l,2omh,2
zux,2ixf,2p8h,2hpl,2q9p,2cdn,2skc,2eun,2xg5,2qd8,2vew,2ez8,2evo,2y1w,2xin,
2z9i,2xul,2w5g,2r2n,2d60,2h77,2z8e,2ji7,2qqf,2i2a,2vlj,2ikj,2wk6,2io9,2pdi
,2p41,2wox,2zaz,2wyd,2cc7,2pyn,2byh,2hr6,2pj2,2zno,2wi7,2nry,2rdg,2w83,2qa
b,2hfp,2w17,2q1a,2oax,2vkl,2d0v,2i5x,2h6k,2ylx,2jbz,2ydt,2oww,2rfi,2man,2p
9a,2zq1,2xem,2aj4,2w92,2ebs,2vn9,2vco,2p6w,2j7m,2fwj,2q5s,2q1u,2q5p,2v5k,2
aob,2rj6,2wmn,2pia,2aoa,2fvy,2xab,2j9y,2cyb,2qhn,2gal,2vww,2e9m,2qfo,2f57,
2c4j,2a1h,2zoz,2ntk,2ht6,2efj,2o07,2biy,2wnz,2pwy,2h39,2cib,2qwx,2olo,2wmr
,2obt,2jaw,2bvn,2jjc,2dsa,2jdd,2x9l,2qg6,2aiq,2pj4,2q7y,2e8v,2j4j,2w5r,2rc
b,2irz,2bjf,2gtk,2v9c,2nsd,2ole,2gwk,2aio,2gfs,2jkp,2ray,2gxq,2viv,2piu,2x
dq,2pa4,2jcj,2b01,2wcj,2vev,2iok,2hv7,2ham,2boh,2wok,2yiq,2yvo,2ycm,2bdb,2
cm9,2cl7,2vz6,2jgb,2az3,2qwr,2amt,2xg4,2avo,2x9g,2x22,2vp4,2vdv,2xry,2rka,
2yp9,2vgo,2zcq,2fmx,2ptm,2fvj,2h2j,2e3t,2i0y,2cdz,2xfy,2v7m,2ukd,2wgj,2wvt

,2vjo,2q38,2z9v,2nxw,2fdh,2ds0,2pja,2hlv,2xjx,2c3u,2ohp,2aa7,2wdq,2xxw,2ag
6,2fky,2f69,2qj7,2p3i,2rl7,2vnn,2bzn,2rab,2b7r,2a7n,2fsg,2v2a,2ckm,2wsv,2w
es,2foj,2zyn,2zya,2q3y,2fy3,2h90,2x2r,2x08,2o9v,2on6,2pir,2rtf,2i16,2fgv,2
gt4,2y5d,2zm3,2nnd,2wel,2q4v,2f8g,2y6q,2ooh,2ccg,2z6c,2qcx,2fs4,2efh,2j79,
2zxd,2v8q,2pwe,2cl5,2h2a,2zyo,2gsr,2c0t,2gst,2h6b,2ync,2p37,2p1o,2cxo,2puy
,2zp7,2yih,2h4f,2y9g,2qur,2yoz,2a86,2b1p,2z5u,2vin,2arb,2qvr,2plk,2ht5,2d5
z,2i1r,2xom,2blc,2okk,2qo9,2j0y,2vhz,2bju,2bof,2w8y,2q7v,2e4u,2onm,2fw6,2s
em,2xsb,2acr,2bel,2qir,2zl2,2gco,2bbt,2yni,2x2l,2rie,2z77,2zsi,2wzs,2yyi,2
ot7,2qv7,2vgp,2pb1,2rgo,2vha,2ai2,2brb,2c8y,2xnv,2vwv,2yed,2y81,2wz6,2xkd,
2fh5,2yir,2htu,2qrh,2bsy,2cy6,2bs6,2b54,2cvz,2r42,2zdn,2ez9,2qcg,2wul,2q6c
,2dji,2ors,2v4v,2wta,2fxu,2wn3,2agw,2r7n,2rth,2vsu,2hah,2rgj,2im2,2z48,2fh
e,2nmn,2h52,2wzf,2idz,2x32,2fv5,2g70,2rku,2c3j,2x61,2wk2,2yhy,2fu9,2cf9,2b
51,2pa7,2ewg,2x9v,2w8z,2w5o,2q1v,2x41,2f6u,2irx,2cda,2p4j,2e2b,2ozr,2y5k,2
pid,2br6,2zgv,2zse,2rtq,2gah,2d1t,2f5z,2flv,2f80,2p8n,2vcw,2o67,2pvn,2jkh,
2gpb,2pd7,2fwy,2o7d,2x34,2tlx,2jbh,2qxw,2z7q,2h15,2x05,2ign,2hzi,2fzk,2ji6
,2cen,2iy7,2uxz,2x4u,2ev4,2yeg,2vbg,2fxs,2nph,2xvn,220l,2gwj,2hnk,2v3d,2py
m,2w67,2q29,2ddf,2jc9,2zdm,2zb7,2ff4,2ze9,2ofd,2r6w,2uvz,2wpf,2qxm,2qgd,2p
j3,2d6m,2osx,2y98,2zm7,2ga9,2v2z,2p34,2cll,2xxv,2iqg,2xbx,2qi1,2d3z,2fhl,2
v0l,2p8s,2hct,2h3u,2bd0,2og8,2of2,2q6o,2fjk,2jah,2qwj,2aa2,2qx0,2e9s,2xbj,
2cgw,2wbv,2j0f,2pdr,2a1m,2x3u,2qbq,2nq7,2f8c,2vo0,2v73,2gqx,2oh5,2vlh,2b16
,2wdz,2xnp,2j0v,2jeq,2qkn,2b35,2h9k,2g6p,2xbv,2a84,2pdx,2q7e,2uuj,2b4b,2yc
d,2vs1,2eae,2zru,2xk7,2xm2,2qwe,2qo4,2d2g,2vur,2fjn,2jcc,2one,2be5,2g8d,2c
fm,2ol4,2vd4,2gnj,2iw9,2zd8,2aay,2hk5,2f59,2ior,2y5l,2wch,2hvk,2vcx,2yid,2
pp3,2j9k,2ops,2o2u,2gu5,2iw6,2glp,2o0h,2zyk,2y64,2vto,2abb,2o63,2ria,2awn,
2gvj,2vnh,2p33,2tio,2vdf,2rhs,2aru,2f7p,2ww1,2z8s,2xxq,2o9r,2x65,2atj,2cd8
,2v7g,2ya8,2dg4,2jec,2ha5,2e2q,2ff1,2cul,2wic,2er7,2fkf,2ab2,2qwh,2vzz,2w4
x,2vo6,2vze,2p54,2xds,2qrg,2prh,2olz,2cvx,2rid,2fbz,2ccb,2v58,2uuh,2yz3,2z
sd,2vet,2y7z,2v3k,2wlf,2vuk,2zaw,2ogy,2chn,2vja,2yc3,2vjb,2exx,2vgq,2vng,2
h03,2blw,2q2y,2bbs,2hf4,2pie,2zdk,2b8q,2xjf,2bfy,2zcv,2j94,2yjq,2ym5,2rtm,
2fmb,2dfv,2p2k,2xhr,2fxe,2yj0,2huu,2x24,2ce9,2boj,2pgz,2o4l,2hnx,2far,2y69
,2q7i,2goo,2gq9,2gz1,2qm9,2q5o,2b7o,2src,2pp1,2vbq,2gdj,2nm3,2vu9,2bjm,2gi
l,2wbd,2fyd,2cm8,2j1u,2o1h,2usn,2weh,2pip,2bu4,2wec,2j1g,2hv5,2r7g,2ber,2w
7x,2id2,2cwf,2rct,2r3n,2oxp,2po2,2fbr,2wx2,2oo8,2dva,2zvq,2q3k,2r9w,2j4k,2
cnd,2rgc,2om2,2q6k,2iy8,2f94,2w0z,2ow6,2ipi,2bdm,2oyl,2qzk,2xh4,2f8i,2jd0,
2bfv,2vas,2vtl,2awx,2vvg,2p7a,2xta,2hkj,2np9,2f62,2aj8,2ow9,2v8p,2j7d,2cgu
,2zec,2gz2,2o3t,2bsa,2hl0,2vno,2zyv,2ick,2f2l,2eju,2b9i,2e9c,2yca,2eax,2p7
g,2vni,2wtj,2p94,2be2,2yeh,2ojj,2o4p,221p,2d7c,2j7h,2xo8,2zi5,2rbn,2ze1,2p
9t,2j77,2xxu,2bgd,2e4v,2by1,2cnx,2yic,2v0c,2veu,2jgs,2pkm,2wot,2bxv,2b7a,2
a0c,2eva,2eur,2x77,2bzd,2xba,2gsq,2uzh,2pui,2uag,2e8a,2zmm,2pg2,2ac7,2jji,
2pdq,2z55,2uyv,2vmp,2o4r,2std,2nv4,2cbs,2ax6,2hej,2vp0,2ilv,2isj,2g1r,2nx1
,2bi7,2pd8,2e6u,2pow,2zev,2np8,2wyj,2r6j,2e22,2oxx,2xko,2iq0,2ybq,2q5k,2uz
3,2b04,2amb,2wvg,2xw0,2zym,2xda,2f8f,2fgi,2qcl,2p9i,2gnb,2yef,2dsu,2eug,2x
xh,2rtk,2b00,2z27,2w8j,2zog,2bkt,2yfc,2vyn,2vvm,2wkt,2ntb,2hu7,2i2d,2amv,2
ica,2ske,2rte,2gq8,2ohn,2p1u,2oxr,2qcm,2r5t,2zdu,2vr4,2b9a,2ojw,2ijg,2qfu,
2qyl,2ygf,2iut,2haw,2fl2,2zo3,2b9w,2evw,2isw,2jj3,2q93,2okl,2npa,2z9b,2r23
,2qd1,2wg1,2bek,2rix,2qxx,2hdj,2rh1,2v7t,2dgn,2hmb,2e99,2zz7,2cxs,2jh0,2pe
y,2o4j,2pzp,2vpr,2hs1,2xej,2ycp,2prb,2pxh,2jiz,2hdq,2vip,2p71,2pcu,2q97,2i
vd,2uvf,2bmv,2isk,2whi,2e5a,2wmx,2z9d,2aqi,2eg5,2jj9,2cip,2huw,2r0d,2fmo,2
jg8,2b99,2wbw,2gpa,2w09,2gvy,2r2m,2cwh,2zvt,2bgs,2hxc,2cir,2zeu,2o3z,2nst,
2iyu,2ooz,2zfn,2wt1,2wpb,2qf7,2ikh,2uz1,2uuf,2rbw,2wkj,2qbw,2cc2,2ewp,2f6z
,2x52,2zft,2pri,2rti,2qdt,2b8t,2gdz,2f5b,2d2i,2add,2w12,2al5,2y42,2ggx,2ye
b,2gn4,2ye9,2obj,2vn1,2jjp,2v3h,2y3s,2pv1,2pin,2jfy,2gg9,2a4m,2ux9,2o7i,2r
4u,2euk,2izz,2vx5,2yxj,2i4h,2x5f,2vfl,2dm5,2i2s,2wv8,2qim,2h3d,2jfg,2hs3,2
zaf,2jg0,2w06,2eut,2vle,2c6i,2pdw,2hds,2e2o,2eud,2wkm,2qrl,2pnu,2x96,2qwd,

2wh8,2zyd,2rge,2xcu,2dkh,2v21,2pdl,2ylp,2d20,2xje,2anm,2gt9,2qzx,2xf0,2flh
,2clh,2g8r,2p1c,2qtc,2iyo,2ydk,2yd8,2pix,2cj9,2rfn,2zcz,2bac,2qe0,2p0h,2qe
t,2ada,2zgb,2hog,2jdy,2bik,2yqs,2oua,2jar,2j72,2zgm,2ys6,2g8n,2vmq,2igq,2b
v7,2h42,2a8u,2erz,2jgv,2fus,2d1q,2h4h,2jdn,2hfn,2pq9,2hha,2awu,2zma,2cog,2
eo7,2ztu,2v6g,2uxr,2zrw,2jh5,2q9b,2uw4,2dm6,2c8v,2b7s,2wov,2ifb,2euq,2qg2,
2q0e,2zl6,2gmk,2gz8,2g7b,2nva,2wr7,2cn5,2oty,2q7l,2xp6,2oci,2ork,2bsx,2hf8
,2e0p,2f17,2cxp,2qx6,2hkz,2nzu,2a5c,2zhk,2yvp,2xx3,2xef,2fx2,2xxp,2qb3,2xp
8,2ec9,2ck3,2phr,2nnp,2h4k,2zi2,2v7d,2omn,2qeh,2hj9,2idw,2wm0,2aqk,2r3t,2w
ug,2yga,2pzl,2xtz,2pj5,2exl,2r7k,2wt2,2hb9,2qmd,2vwz,2q6m,2d3u,2zn7,2v3s,2
v7q,2ccs,2bkx,2iw8,2w6p,2wzm,2rdt,2o1v,2ohx,2byi,2pav,2ofw,2pys,2o7n,2d23,
2xrh,2zz2,2r0n,2pdf,2omi,2w5b,2bw7,2pvf,2piv,2vu0,2xyv,2yea,2ete,2clo,2agc
,2olq,2qf1,2fke,2iid,2oxi,2vvs,2hqm,2vx6,2hj3,2va7,2pqc,2oze,2cjm,2hnl,2pd
y,2pdj,2wtn,2pw3,2b9y,2fzm,2c91,2qvz,2f1g,2p83,2av6,2vmo,2q70,2csn,2x5o,2f
99,2owb,2h4x,2r7m,2vk2,2ovu,2axa,2aco,2wzg,2w3k,2zpr,2xiz,2hgs,2dw4,2y8l,2
jjj,2fgq,2uz2,2vpq,2r64,2g9v,2g86,2w52,2gc8,2hox,2z92,2zc0,2wdy,2bew,2wky,
2bkj,2v05,2bhw,2gkl,2ql9,2qeb,2jih,2qm7,2b96,2a83,2yk9,2j4i,2vf7,2w97,2xeg
,2i4o,2if8,2zgh,2qz4,2qen,2phw,2wo8,2enb,2inv,2kce,2vhx,2jf0,2ewb,2ntv,2y7
p,2vvn,2p2i,2arx,2q16,2uxh,2bm0,2arm,2wi6,2ven,2ay1,2z4o,2vfe,2ako,2qgi,2r
ar,2a2r,2hp1,2aqx,2z5f,2pit,2f34,2reh,2rc2,2o3q,2isf,2ntr,2vo5,2wmv,2hw1,2
afz,2gtn,2ohk,2jlb,2rkv,2x2w,2ezt,2xk8,2eu3,2fsh,2b55,2fh6,2or4,2jbm,2zcr,
2oke,2y30,2xog,2ycq,2f9g,2xm1,2vss,2v7l,2i2f,2x4p,2yfb,2xei,2v3a,2wkr,2fvk
,2y1f,2xi4,2uyn,2j27,2vqz,2b9j,2g7c,2dv0,2hfk,2a4n,2f7c,2gyv,2a8y,2a40,2x2
0,2c78,2wf1,2vgd,2vt5,2g72,2qg0,2zeb,2pw0,2yno,2y1p,2fp4,2q1h,2an5,2d36,2a
id,2eum,2sar,2qcu,2x23,2rdk,2qrq,2h26,2ez4,2qio,2qwl,2b77,2f7i,2nto,2jhi,2
wb5,2fhc,2rtr,2xgw,2pog,2zb4,2uvy,2gni,2w6m,2ylq,2zdv,2z1x,2wk9,2v7o,2qes,
2qx7,2a78,2wly,2qnq,2y4g,2wdb,2fwp,2zkb,2ayl,2rnt,2qmf,2w0b,2bdf,2z28,2vi0
,2r75,2q3r,2poc,2gqa,2a65,2fjp,2a1n,2o06,2z4y,2pkl,2fme,2nsx,2f3u,2g5b,2y7
x,2q94,2wp6,2fb3,2xb5,2efe,2qvv,2vbm,2z7h,2r84,2cne,2a06,2y4j,2znt,2xgt,2j
cs,2yd1,2qt9,2ye7,2azb,2wjo,2dbz,2w14,2dsc,2hyq,2bd4,2xis,2fc0,2fn8,2nwr,2
cyh,2w7y,2flm,2r2h,2w95,2dt3,2h1f,2x8d,2cw6,2b9f,2q4y,2w8q,2x8s,2er6,2rj7,
2pr5,2y4s,2qhd,2eix,2whl,2ce8,2gga,2zc9,2wpw,2fr1,2z5x,2pze,2rj8,2pvw,2bne
,2v5a,2vro,2qjm,2vi5,2iyy,2rg5,2eb5,2r3q,2icq,2g24,2g30,2yit,2xtn,2gj4,2x4
z,2c0i,2uuu,2a3w,2pe0,2orp,2oz5,2yaj,2o58,2x92,2q55,2dqm,2exs,2dkd,2y7f,2g
l0,2nv2,2fxv,2bss,2uvj,2d2v,2v7w,2x63,2uzw,2x91,2pdb,2oh4,2w9s,2r43,2x3f,2
pi8,2zyw,2j41,2cmc,2rhx,2quj,2zbz,2evl,2akr,2zwu,2xhk,2vcn,2v17,2b53,2fzh,
2c3p,2xi7,2gpt,2yej,2fpv,2a3a,2vfh,2qd9,2zpa,2gtw,2fw3,2oc2,2cxg,2whf,2imw
,2rh6,2ww4,2yaz,2p5e,2qwb,2nv6,2j80,2pax,2vie,2azz,2acs,2ovw,2ovv,2cld,2zs
a,2ha3,2pvy,2rfs,2uzs,2whw,2pwc,2iv7,2e4g,2h3p,2fkm,2ol0,2jkk,2q9u,2j75,2c
1x,2ze7,2wc6,2xpb,2ovz,2rkd,2hnd,2j1v,2ii3,2f6y,2wyz,2fdp,2nmz,2fyu,2z9z,2
g78,2c03,2c6o,2bt9,2yjd,2gag,2yph,2zps,2wgz,2xp9,2cfu,2o4g,2a94,2qxl,2w70,
2uw8,2phn,2z81,2cd2,2qr9,2xyt,2h9t,2v60,2zfu,2r6y,2qci,2xvl,2ofu,2vqt,2iby
,2p2m,2qhs,2yx4,2x1t,2x2m,2cct,2al4,2v88,2b5v,2pj8,2bsr,2o7o,2v3o,2zj3,2rg
a,2i6a,2z4x,2gsu,2jdp,2w5s,2wuf,2r0c,2zmf,2g5f,2wmc,2qx9,2dfn,223l,2pam,2b
cd,2qo7,2e8t,2req,2c9z,2wju,2vgk,2agv,2xfq,2ylz,2d7i,2gv6,2nta,2oq5,2nnk,2
v0u,2ifw,2zb0,2f1a,2pqz,2wow,2fc1,2gnd,2epn,2yg2,2pdn,2v59,2gq2,2f1b,2z8f,
2g00,2zey,2xde,2w1h,2xz3,2ido,2iyw,2jjq,2p4s,2vj0,2gnf,2bnq,2qii,2d37,2i0d
,2rb2,2idv,2q1j,2vwm,2cng,2ay2,2hwq,2we0,2gd0,2b1g,2ixi,2scu,2hb3,2i1v,2uz
t,2vta,2a15,2w05,2zjw,2g0h,2ox3,2dw6,2gss,2wmq,2r86,2qrd,2wyw,2gek,2axn,2d
81,2r0h,2puh,2nun,2xp2,2rtj,2q4h,2pt6,2qbo,2px8,2w26,2qse,2oq6,2py3,2e5v,2
hoh,2c6d,2cm4,2xfp,2i80,2zpk,2ce7,2vg0,2c5l,2gqu,2ng1,2bu2,2vku,2h3e,2d3s,
2nlq,2zox,2r00,2x7t,2r6r,2q37,2imi,2fk8,2o4c,2i0g,2rgb,2bog,2dbv,2ihq,2oz7
,2rki,2qs3,2ew6,2hxl,2ds1,2y5s,2ydw,2e3n,2phb,2uv2,2a3y,2bs3,2oym,2z8d,2vw
x,2z3y,2gzw,2wbq,2p69,2o5z,2fju,2gd2,2gd6,2fop,2cjw,2fvd,2d0e,2j38,2jae,2y
m7,2zu2,2bcg,2ovx,2wc3,2i66,2hh9,2r38,2x3n,2gu8,2f3r,2vtq,2xwl,2is0,2chg,2

azd,2qi7,2dor,2r2l,2lbd,2v6o,2mjp,2vbv,2j62,2r2b,2e92,2ef6,2oun,2uuo,2pot,
2bm2,2i8c,2c6h,2hmu,2exm,2cgf,2zfp,2ak3,2eex,2yge,2ai8,2nw9,2jb1,2zgw,2v1a
,2cb3,2xp5,2wbg,2pdu,2xgs,2obm,2v51,2wfg,2yi6,2e5c,2p9h,2ff6,2bto,2nt7,2qw
1,2bb9,2aot,2r3k,2zi4,2iht,2oro,2p1t,2qt0,2jkc,2ae7,2ynd,2j1a,2fb2,2fue,2b
jk,2hmv,2fzb,2gjp,2i17,2fn1,2pdp,2x7i,2f7e,2yex,2xig,2bzg,2ao6,2xx4,2v96,2
pwf,2evs,2bmb,2rbv,2wyg,2c93,2r0z,2e9o,2pgr,2e7f,2cn0,2nn1,2wl5,2e93,2xw1,
2zb3,2z4b,2j65,2xpa,2x2o,2xi3,2o2c,2bbq,2ed4,2d24,2y1d,2bxz,2iyq,2d41,2wa8
,2ewm,2j6x,2cxt,2vs4,2x7j,2uwn,2wj4,2pye,2yl8,2rgg,2vmx,2bys,2xiq,2oi9,2z4
v,2b1n,2gou,2pjo,2yy8,2as2,2dv9,2ab8,2nsl,2vbu,2yyl,2cji,2f4j,2xdx,2bhz,2i
67,2zof,2jcq,2ou7,2kin,2gg7,2j95,2bdj,2uwl,2ykn,2ok8,2j4a,2vmf,2fhk,2iei,2
wa2,2nu7,2nz2,2c1z,2c02,2zin,2ax9,2ei4,2w3l,2ews,2c3y,2uy5,2wea,2whx,2c12,
2uw7,2gvq,2q3c,2h5a,2duv,2g9k,2qnn,2vct,2h92,2j6s,2y82,2ay9,2rcl,2wvj,2i4d
,2fdd,2pez,2w6n,2y1x,2z8g,2qtb,2cl6,2x93,2gc3,2f9k,2i0a,2hzy,2xq0,2yc5,2r5
e,2f64,2cix,2gwl,2qoc,2j1t,2x8y,2r3l,2xtk,2z1u,2ojg,2q5q,2otz,2jfl,2ztg,2i
hv,2wxb,2q61,2ddh,2vg1,2y3r,2cma,2wf2,2ojt,2zz5,2pyd,2vut,2gyw,2ci0,2viw,2
fmn,2vwc,2v2e,2xa2,2nos,2dxs,2c4h,2xcf,2wze,2r40,2c6l,2fzj,2fyc,2b03,2wf0,
2z7l,2rin,2x7c,2pj0,2yls,2z7k,2p2l,2xh7,2qmc,2y8c,2a4x,2qx8,2o2y,2ok1,2bs5
,2vmg,2iqd,2c94,2dqt,2jfp,2h4g,2ofx,2i7p,2qds,2rj1,2xht,2eus,2iaj,2z52,2hs
8,2c68,2oal,2w1u,2oxy,2v41,2d2o,2fje,2d3y,2xva,2v8x,2zir,2cuz,2q46,2r5p,2c
c8,2xbn,2xn3,2y27,2hjp,2fj1,2fut,2j2i,2hk9,2bgj,2dqu,2oi0,2pjc,2qtn,2ql7,2
eft,2h1h,2zxg,2qum,2eu2,2o5d,2pmk,2riy,2vzm,2bfr,2c3w,2pk3,2vx9,2uv6,2y4o,
2yb7,2e6v,2bmw,2a3e,2yix,2c1p,2ort,2jls,2f78,2pza,2xx2,2h02,2zw9,2uy4,2y85
,2gfj,2x0k,2y5e,2ybo,2qw8,2pob,2b3d,2ouu,2f1k,2imf,2c8w,2xc0,2bk5,2vez,2bf
c,2x06,2v2g,2q92,2zb2,2x0v,2ew5,2zj5,2oq7,2py4,2qr2,2fjb,2o1c,2z9c,2xfi,2o
ff,2zpt,2vqu,2aie,2e8u,2wxp,2cye,2o0l,2hty,2p53,2nsh,2r1x,2aqb,2qge,2yp6,2
rc8,2xn7,2ft0,2wer,2aib,2zb1,2h55,2vx1,2x00,2j9e,2q1l,2z4z,2nt8,2gm1,2vtd,
2rbr,2ffb,2egh,2ihk,2c4i,2z9y,2io6,2ozl,2d7d,2cig,2vrx,2vnf,2brc,2q1t,2rkf
,2x0u,2j1e,2gyi,2i65,2qoe,2a9y,2rlb,2pwd,2vj6,2pwg,2vtt,2bmd,2ow0,2bff,2jc
k,2qfy,2vp5,2wnb,2auy,2f7a,2cts,2uy3,2z3h,2yct,2wc5,2pnz,2wm1,2je9,2wka,2w
k8,2q6s,2c2w,2v3u,2iv3,2cej,2ow7,2x5d,2qd7,2q6h,2uyi,2d3l,2chx,2fqy,2fik,2
qmj,2owz,2nuo,2mas,2zd0,2abs,2rbt,2jh7,2ydu,2vfk,2r3o,2x09,2vsl,2c25,2o4k,
2i47,2vqd,2j3u,2yye,2vc9,2wd3,2v7k,2o9t,2q0a,2gfd,2o40,2f2h,2a5b,2xxr,2gjl
,2wgt,2p8o,2bif,2x7d,2gc2,2bab,2zax,2q1b,2jcv,2e7w,2hyy,2f4b,2ei7,2par,2qs
4,2avs,2bqz,2pvr,2g8a,2xc5,2o78,2j4d,2vtj,2znk,2jcx,2pau,2bsc,2oa1,2dri,2p
wl,2z25,2opm,2cbu,2jhg,2a2d,2vwh,2qbl,2xjv,2jke,2p2j,2npx,2fqt,2dvu,2qc6,2
uwd,2fib,2vwn,2gq3,2ag2,2pvh,2ycf,2uv5,2vfu,2woi,2y5a,2rfh,2yqh,2qdx,2c53,
2ay4,2vlr,2ye3,2vtn,2d0k,2c69,2qcs,2cmd,2ffu,2xff,2uup,2pr2,2p3j,2a0x,2imk
,2x8z,2d6o,2w68,2asf,2gfk,2vch,2wmo,2xdu,2e1t,2of8,2as1,2est,2xck,2b7n,2eu
u,2rgp,2wcv,2git,2vfz,2gbb,2q28,2y61,2bir,2cnh,2dur,2a8w,2izi,2xys,2vij,2b
aw,2ilz,2nt6,2cgx,2ft9,2ibw,2qo5,2cbv,2uwp,2yrx,2g1y,2bu5,2g2y,2wos,2who,2
qn1,2jdv,2ef9,2fnn,2nxe,2qvd,2a3h,2hka,2bpv,2xjg,2vcq,2qpy,2ayw,2bpm,2on5,
2uze,2buc,2c8x,2zut,2ej1,2zr9,2jd5,2xbp,2zyu,2xe6,2fpl,2bme,2bkv,2viy,2gyo
,2bx7,2y65,2r09,2xke,2h9i,2jdk,2oei,2vo4,2gcq,2jkb,2o3s,2olr,2r3g,2wcm,2b3
f,2fqw,2wn9,2bt4,2c4w,2aes,2pfc,2cvw,2xkc,2snm,2ztk,2gft,2nu5,2i8t,2bvc,2h
6t,2hk1,2as3,2q64,2qz3,2vvl,2i56,2rb0,2v5e,2d6n,2et1,2nli,2dio,2cns,2hre,2
rdw,2okg,2w1a,2a5x,2zgn,2bsm,2b5w,2xd6,2fyv,2wez,2pwp,2q2r,2uym,2gew,2d38,
2ds8,2fdf,2qrm,2rhy,2wi3,2xoi,2wej,2r1w,2aq7,2dvx,2q8m,2wxg,2bnj,2c27,2vex
,2e9d,2wyn,2pks,2zrv,2v95,2onp,2h4j,2coi,2ffc,2c7v,2yja,2o4z,2nnq,25c8,2fq
i,2hhp,2ohs,2wqp,2f3s,2zzv,2c1a,2wog,2hb8,2zjf,2ygn,2ihj,2cyc,2c3h,2ak1,2c
b8,2euo,2qx4,2zvp,2wn7,2pe1,2ccj,2rtp,2f67,2jig,2a2i,2cik,2vhj,2ohq,2tct,2
ylo,2i4p,2wlq,2fpz,2xii,2qnp,2xoc,2y6l,2c5a,2i6o,2qjt,2hwr,2pdm,2rhc,2wco,
2b1r,2r3m,2y5z,2yjw,2p5n,2dpm,2vpp,2z4w,2f3p,2qi3,2uzi,2b4q,2ovh,2r2w,2xau
,2bst,2yei,2zm9,2wqe,2ajx,2x9f,2io8,2ffa,2c5b,2v0d,2vg2,2wts,2xe7,2yr5,2jk
y,2yyy,2feu,2ff2,2zkj,2p1p,2gfa,2nyr,2xno,2by0,2w3i,2oaz,2w15,2bmz,2j51,2b

sb,2rjh,2i3h,2rdu,2pbw,2qs8,2cbx,2gcg,2yb9,2azr,2r14,2dkn,2fjm,2p8b,2tsc,2
q4g,2b0m,2gir,2zzw,2pxa,2jd1,2qd4,2vnw,2rde,2fyb,2h7c,2orr,2vxk,2fto,2ghm,
2vtp,2vzs,2xd3,2wpe,2vj7,2d0o,2wd1,2asp,2xj7,2iba,2p99,2w63,2ivv,2ix0,2wmi
,2oly,2v34,2pun,2v8h,2hvn,2p1v,2wxo,2pyw,2jdo,2clm,2vkg,2pjl,2pz8,2zgo,2h2
3,2pvl,2jdi,2xzi,2vo7,2j7g,2a7x,2b14,2qoh,2j1p,2pdg,2vjx,2i4j,2drz,2dqs,2a
ac,2bf6,2ien,2j33,2whm,2ikf,2xc3,2dhn,2xam,2d1k,2v7b,2wcx,2nnj,2bs8,2y5f,2
ypc,2xyj,2icy,2jcb,2cv3,2bxp,2bha,2yfd,2zdy,2wor,2xn6,2hsa,2q2q,2x1s,2it4,
2a8h,2zb8,2gks,2vvo,2fub,2xn5,2a0s,2bz6,2iyv,2xce,2q5f,2ahc,2je7,2ofp,2qtg
,2qtt,2are,2y6p,2gg0,2nq6,2icx,2xb7,2j90,2tmd,2gmm,2fpt,2qjn,2qv4,2znn,2ay
5,2ie0,2yof,2f38,2qgc,2jdt,2pvv,2btr,2e0a,2vab,2ei6,2d1n,2cf8,2vyv,2zts,2c
04,2qgw,2wep,2iuq,2qd6,2jib,2xyr,2uue,2wc4,2gh6,2i19,2c5e,2zzu,2qi4,2ama,2
hdh,2i5f,2c3c,2jf4,2vey,2zmi,2ag5,2vth,2hnu,2azy,2ati,2v8e,2dx7,2qqd,2fzc,
2vdg,2rfx,2jaj,2v9n,2ofb,2oj9,2x62,2c8f,2z49,2o1o,2y5h,2pdc,2c92,2oxz,2ddq
,2xnm,2wcg,2yac,2hui,2i4u,2vx7,2vos,2iu8,2z1z,2wl4,2vke,2ipw,2izj,2hd1,2ye
2,2pqw,2oat,2foo,2o7b,2ya3,2jdm,2pyi,2ba9,2hvw,2cjz,2bpz,2q72,2xc4,2ra0,2c
0u,2cnc,2fav,2bsw,2zhr,2gj3,2v8w,2yx1,2osf,2d5x,2z20,2j5z,2pql,2wue,2uw0,2
q58,2y3f,2b4d,2hx3,2z3n,2nn7,2c3i,2rtd,2hm1,2ye4,2wu4,2cem,2ai3,2rh4,2hh5,
2y91,2y31,2rtl,2q7t,2qbu,2wih,2r2g,2j7t,2b4f,2hiz,2zcs,2zgz,2cnt,2dux,2dwo
,2y7k,2w0a,2woa,2yab,2qrp,2bu7,2wf3,2x70,2fgu,2xoz,2j09,2zmj,2dt5,2vtz,2ie
g,2wvs,2zwt,2wci,2hvo,2ima,2pdh,2qwi,2bzz,2rio,2ff3,2i6u,2ww2,2jff,2j4z,2v
8y,2q88,2zla,2vwq,2a92,2wgg,2fox,2g25,2uv0,2hmp,2hl3,2e3p,2z93,2q8s,2gjn,2
qzr,2whq,2wgv,2bok,2e3q,2fzi,2yw2,2vzt,2b82,2p70,2qgt,2zfi,2foi,2izl,2ctc,
2v0i,2iyz,2evc,2no7,2xk3,2ohu,2wr2,2vqo,2ieo,2y33,2p95,2b6n,2jdu,2a1o,2wji
,2acq,2wgc,2jl1,2whg,2wnu,2jew,2g20,2vts,2j0l,2j2u,2gms,2ah9,2xfk,2c67,2yo
r,2b15,2bhe,2vvt,2dln,2pqd,2jl4,2zwz,2g26,2xve,2q8d,2r8v,2bp6,2r3p,2bwj,2r
1y,2rox,2gnm,2g89,2iki,2yve,2brp,2gc1,2zok,2tps,2gzm,2gdu,2jlc,2f6w,2jen,2
bod,2npq,2v00,2xbk,2q7j,2pnc,2nsj,2yaa,2qbp,2e56,2ibz,2q0d,2fgr,2r3d,2wya,
2yxu,2wgh,2af6,2q1w,2wmh,2a8x,2v77,2qry,2qzz,2vrq,2faq,2vcs,2os2,2uyw,2dub
,2j44,2aa0,2yfe,2bo6,2j9g,2hkf,2av7,2c7u,2qq0,2qf2,2yhd,2zl4,2vcv,2pzk,2xd
9,2c1n,2wle,2hkk,2r3h,2x21,2aax,2gv2,2gyu,2fna,2oos,2dt1,2ccv,2p0d,2h79,2n
mx,2i57,2clc,2eg8,2cja,2bo9,2px2,2ez7,2xim,2vrj,2ye5,2h44,2bk3,2v2v,2ch5,2
vh0,2e7x,2ar6,2jhk,2iyf,2yio,2jko,2gn8,2rke,2cfc,2wyv,2cbj,2sli,2b07,2eg7,
2ivn,2oah,2zmk,2riu,2vcf,2ii5,2p93,2z6j,2xon,2wz5,2rho,2q8z,2fm0,2wu7,2d2f
,2j9c,2aeb,2pbd,2iit,2cp4,2c6e,2jfh,2xn2,2ae2,2vwo,2d44,2x0y,2idx,2qqc,2yk
l,2vwp,2rbu,2wr1,2w66,2za5,2qzl,2gtm,2f6d,2qa3,2p20,2fvv,2a42,2v8g,2qn6,2j
8z,2rbs,2g94,2cvd,2iry,2bu8,2vcz,2g5u,2dt9,2x7s,2air,2yhw,2y7i,2iej,2nxi,2
p73,2oxw,2f35,2pu0,2c6c,2a7p,2xp3,2hzl,2bxh,2aw3,2e2x,2xvv,2zga,2jdc,2bnn,
2i6p,2x90,2gzd,2wi2,2gn3,2e7l,2exj,2has,2c1s,2q7q,2hf9,2g99,2as6,2hf3,2zio
,2cek,2q7k,2f3k,2gde,2fwz,2bh9,2clz,2pgf,2jbj,2j7c,2d2h,2fcr,2x8g,2in6,2y6
k,2zuu,2xdk,2rhl,2puv,2pfg,2cni,2od7,2j59,2vhe,2d4v,2gdv,2zhz,2wq5,2fgb,2y
00,2jao,2g7q,2cnf,2oh6,2p5w,2yjx,2qhv,2v54,2c97,2pul,2gsa,2ax7,2r5q,2yg0,2
pzj,2rb1,2hny,2w8r,2f3q,2aa5,2igo,2f71,2quh,2e94,2y6g,2bvp,2il4,2yp8,2jb3,
2c3m,2nom,2o8h,2dwu,2q1q,2z29,2wpc,2oag,2apc,2fhn,2o8j,2c3d,2duq,2gz3,2ij7
,2wjh,2eup,2wnc,2xwm,2gtx,2a9k,2tep,2vq5,2wsb,2fr3,2wyc,2o2q,2cbz,2ya7,2e5
9,2clk,2nuu,2zfa,2a8g,2z6i,2z4t,2ycr,2rc7,2zim,2az9,2ceq,2ri1,2ha2,2ybu,2f
zd,2fp2,2q6v,2e1z,2guc,2qca,2p2b,2pgl,2ot1,2z7r,2yb0,2jc0,2ofv,2rbq,2fz8,2
x99,2rnf,2bwc,2qno,2waj,2fa1,2zl9,2duz,2fsv,2j32,2aia,2qwg,2o3r,2xib,2ofe,
2xnb,2pel,2ohv,2h9e,2z6d,2piq,2cqs,2rht,2cy0,2yay,2hj4,2rhw,2vzu,2x94,2jcr
,2hwh,2x38,2cgl,2e9a,2hxq,2v7x,2vyt,2wvz,2e2r,2jkg,2z97,2x6t,2q0k,2ph8,2wq
4,2uuv,2o3u,2wd2,2yg4,2p4y,2var,2x39,2wed,2wwf,2dxu,2oeg,2o3p,2a9j,2gk6,2i
4q,2uzl,2hu6,2boi,2cjh,2xbw,2xez,2hwi,2nv7,2vfs,2ay8,2qp8,2nvd,2i4z,2d8l,2
gg5,2oy2,2i2c,2acu,2xh1,2r5v,2puj,2fsz,2vh6,2vor,2tpr,2cxq,2vwy,2bro,2i4v,
2nz5,2rl1,2xum,2csc,2ph6,2alg,2wva,2g79,2fpu,2xuc,2qu3,2aqd,2w7k,2fr6,2zyi
,2iyx,2fh8,2x2p,2gru,2g1a,2vhw,2hpt,2yme,2cdo,2zas,2qpu,2x2t,2orj,2e27,2ym

6,2hvd,2fcu,2fx6,2zwa,2gtz,2v1o,2bwm,2cle,2zuw,2fqo,2izf,2f7x,2hfo,2uvm,2q
b5,2xfn,2a0w,2coj,2o66,2iuz,2uzv,2psj,2xm9,2a5d,2e5d,2pj1,2ydj,2cl0,2trt,2
wuu,2zxi,2pzf,2v7v,2r3x,2xh0,2o7f,2vqm,2a14,2pae,2cqt,2zba,2xod,2oq2,2wn6,
2jfx,2h8z,2aov,2xix,2cxr,2vio,2q7w,2pho,2nws,2yy7,2zpq,2h13,2hps,2bes,2vu1
,2b9x,2e4w,2agt,2fpy,2er9,2ggh,2ixh,2zgy,2ccc,2jau,2bu6,2bs7,2qui,2w5t,2vv
u,2jfz,2hjb,2bvw,2e20,2vap,2d5i,2wt0,2c49,2eac,2qei,2du2,2rk2,2jf9,2qu6,2i
ne,2g1s,2vvc,2vdy,2d0n,2uxi,2xj2,2w13,2hrm,2pe2,2vat,2ol1,2ast,2no9,2jhp,2
og2,2ntq,2z8l,2yp4,2om0,2ymz,2vw5,2ji8,2giu,2ikg,2yel,2qyn,2rgh,2nuc,2guo,
2hyr,2zwi,2oo0,2p2a,2gtf,2p8u,2flr,2p1e,2ps1,2o0u,2wr4,2j5b,2brl,2vkh,2pu1
,2aso,2uxu,2ovd,2r58,2iyg,2zj0,2vwt,2f18,2btm,2x9d,2z3u,2xfh,2bj4,2de4,2ft
b,2fvc,2zx8,2q6r,2dtj,2aq6,2a9w,2is6,2xqq,2i0l,2ydm,2qyk,2ejf,2vx0,2x4o,2z
i3,2yk5,2gv7,2yi7,2r3r,2ybs,2ogz,2cyf,2viq,2har,2bp1,2q63,2pka,2oln,2qeo,2
i1q,2fzg,2g1k,2dcf,2gh9,2ow4,2w3o,2zfs,2gfe,2px5,2a2q,2gci,2sfp,2v8m,2pmh,
2vjd,2xvd,2z60,2wat,2x42,2tbs,2y0e,2qie,2rj4,2itv,2x5w,2w90,2yr4,2jc4,2bup
,2yk1,2gqg,2gbp,2uvi,2h6q,2y46,2bza,2xk4,2xjb,2bak,2ym1,2pzg,2zej,2bvd,2ym
4,2h6p,2ai1,2qi6,2xkf,2rl8,2w5f,2ze5,2f70,2wmg,2pc8,2uw5,2dyp,2i1m,2y5g,2x
yd,2iw1,2p7q,2od2,2v7y,2c84,2a8s,2vqq,2xel,2zxc,2r5n,2iiv,2dap,2pn6,2plm,2
vca,2ixa,2a2s,2pmp,2ajv,2xy9,2r7l,2inz,2itm,2nq8,2c3l,2zmh,2ovy,2cmf,2bb7,
2r3i,2h9d,2a21,2d1s,2phf,2bxm,2wvu,2ex1,2jjo,2gg2,2uzo,2vu3,2tsy,2rc0,2fxd
,2bwg,2qb4,2f6x,2xrq,2b52,2vba,2f7o,2vv9,2avm,2oiq,2xz9,2im0,2acv,2x7b,2f3
2,2o1u,2xg3,2gxs,2jkj,2ns1,2glx,2uyp,2psv,2wlz,2fdu,2d09,2rg6,2yig,2asm,2w
9h,2wd8,2we5,2qo6,2our,2bej,2pr3,2nsp,2afx,2c98,2w5y,2p3t,2qey,2wj6,2pd3,2
c6q,2zj1,2a5f,2qbn,2jiw,2r9z,2vfi,2wne,2qiv,2qmg,2rc9,2rbo,2qk4,2wkw,2jds,
2uyq,6poq,6on3,6e2u,6s45,6j1i,6tlm,6mbp,6jtd,6t2k,6sj8,6cb5,6lq2,6lx9,6f22
,6a8a,6x1y,6ura,6vcj,6cgq,6rnw,6c7b,6rlv,6l6c,6ox2,6qlp,6jad,6juj,6cbk,6n1
e,6jjm,6eb2,6x2e,6f7l,6hxp,6g33,6opx,6eqx,6bdq,6pr1,6ix9,6evo,6gzm,6cj1,6r
kj,6v6y,6f28,6d6m,6nx8,6e9a,6t8p,6usy,6o7r,6rq5,6cyb,6rl3,6hgz,6c1r,6im3,6
sjs,6idb,6j18,6dz3,6o4d,6ylc,6qox,6p2u,6m8e,6yqt,6fpm,6cy3,6jsz,6t0p,6huy,
6eee,6c9b,6bc7,6toy,6aot,6aao,6i74,6q9g,6cgd,6d1g,6xae,6rgx,6ixd,6k5j,6hp5
,6hjd,6l5s,6qdg,6v3j,6mj4,6hmm,6nhc,6gaq,6pvx,6ayq,6ujj,6l11,6kvc,6l0x,6xu
e,6h09,6ocl,6hwu,6qal,6hrw,6t9c,6dup,6cy7,6r4u,6b1n,6od6,6l5o,6ssy,6rd2,6j
wx,6i7i,6h4x,6abp,6qp1,6q13,6s50,6qou,6pj3,6pgn,6gi8,6el9,6cdy,6y4e,6v9l,6
c1j,6no1,6tpd,6p3v,6f7u,6nsd,6jn6,6z08,6g38,6fz4,6aap,6f60,6kjj,6lxc,6suh,
6yug,6tm9,6ooe,6no9,6lkl,6roe,6pix,6c2r,6s74,6h37,6oxu,6gl4,6rby,6mvt,6hl9
,6lpf,6kxn,6f3r,6t49,6o5m,6mcj,6g91,6f6i,6d78,6e7i,6s71,6pf6,6l7x,6npt,6bc
3,6htn,6si4,6n78,6usv,6eck,6w0n,6lx6,6hqr,6b3c,6p9q,6zi3,6wmv,6sa3,6g92,6h
ou,6s0f,6sah,6y00,6ku3,6qo2,6wrk,6yii,6et8,6pjo,6z4d,6otg,6i5i,6szh,6d15,6
g7q,6l4z,6d5j,6faf,6sal,6c7j,6zl3,6bmw,6j3c,6hgx,6os4,6lon,6mr9,6gw2,6mnh,
6g93,6q4b,6fu1,6npa,6h74,6eiz,6lw2,6hl2,6pg5,6lce,6bvk,6hp0,6n9l,6pvz,6r66
,6w4y,6ku8,6odz,6qxj,6x2r,6jaz,6b4h,6pr6,6u26,6fx3,6ep0,6qag,6si0,6pyb,6ui
f,6hs4,6qaw,6u98,6gbv,6axm,6vs9,6jlv,6gas,6tal,6ez6,6d1a,6fgv,6r4g,6hso,6i
3v,6sxr,6ksi,6bfn,6fqn,6pgx,6r1b,6wf3,6fdc,6f4s,6d6o,6p5s,6oa0,6e3d,6oal,6
f94,6hmp,6sir,6yei,6djc,6gw4,6ooj,6eqq,6joz,6qll,6haj,6spu,6yta,6or1,6ekd,
6i2p,6he2,6fyf,6b0y,6poh,6pn8,6tew,6n7a,6spy,6o0g,6mtn,6eux,6msa,6llw,6t2t
,6cpt,6rnp,6oio,6mh7,6dh8,6qlo,6w0z,6eku,6qeg,6qcd,6h7z,6jmh,6r4t,6k58,6x0
k,6u03,6oi8,6a8n,6tzx,6hl3,6q27,6sh6,6d9r,6br6,6xzx,6rtt,6q7a,6d4a,6ouv,6q
qi,6mby,6rqd,6h8r,6t11,6lju,6tr3,6egd,6w07,6dil,6ftw,6d49,6op1,6aos,6u2x,6
pdb,6lrk,6u5m,6rgz,6fgd,6sp0,6ijl,6ebw,6mgk,6ujf,6nhe,6bo1,6e4l,6o9x,6qef,
6gfp,6i9m,6goh,6s7u,6oxh,6dv0,6cup,6qoa,6sk2,6frm,6lpy,6bu1,6qfe,6uvv,6t2w
,6q9q,6x7d,6etf,6j76,6mps,6fx0,6cqa,6w35,6b5h,6gck,6ncn,6e05,6eo9,6h40,6qu
x,6pnx,6q0u,6u05,6p8b,6f92,6mbv,6qgf,6q4y,6dqt,6fjm,6zl6,6czy,6hzc,6jv0,6y
k4,6nwm,6k0t,6ft7,6ukx,6ebe,6nd2,6sum,6ggj,6guk,6ea1,6q9k,6yzr,6rgb,6sat,6
oin,6anv,6fc8,6bmr,6y6d,6pzs,6qgk,6hb5,6o4w,6h3f,6cjj,6r5d,6wlx,6v1l,6rl4,
6aix,6k4z,6e4f,6wrn,6mjg,6mt3,6u5y,6cha,6jz3,6dje,6op2,6tfq,6yao,6q96,6onj

,6gph,6fs8,6vt9,6gjw,6znd,6ry6,6nsp,6vo6,6d56,6eoz,6u6c,6rg2,6ufd,6kp7,6ne
v,6qrn,6d3q,6epp,6f84,6yo3,6a8c,6lq8,6e6g,6ps2,6uq9,6f1w,6r7a,6r19,6auu,6e
k3,6dnt,6dj2,6mxb,6u4p,6a8b,6aby,6ivx,6pvd,6cjl,6oxx,6gtz,6rgj,6k0g,6llz,6
o9c,6br9,6f8b,6qr8,6c8p,6tot,6do5,6t6r,6gqu,6htm,6p99,6ho9,6qrb,6ldt,6o9p,
6myd,6p5x,6hof,6jt4,6n2n,6n95,6qf4,6gtq,6rqo,6s90,6w6z,6c0m,6kse,6y7x,6gl9
,6lp6,6tx9,6yp4,6wj9,6r4d,6ll6,6bjo,6bvj,6sas,6fxm,6yqu,6q9h,6b2w,6btq,6qr
l,6svz,6np3,6a8h,6r9v,6es0,6sph,6q2w,6uo0,6f3m,6b5a,6toz,6mvs,6fiv,6u4t,6u
49,6ytg,6h4t,6fjf,6ol8,6p2l,6h46,6ul5,6o3z,6x8f,6lqc,6ev0,6qe0,6ufs,6v6l,6
qz6,6kof,6jg9,6svg,6tdf,6rjz,6qi4,6hfk,6pkc,6h1l,6d9x,6q4d,6qlu,6pnh,6pn3,
6j1v,6ect,6wii,6wk2,6qgd,6pne,6ssg,6o5u,6r4k,6m8c,6vns,6mjf,6flz,6fi0,6zjt
,6nx3,6bca,6f06,6nbl,6ngx,6gst,6xpt,6eeo,6ghh,6cn6,6yti,6p78,6j17,6e6p,6o5
v,6cq1,6d6p,6q28,6m1s,6uoj,6leh,6d1l,6l1h,6hkn,6h59,6qmt,6spw,6bxo,6pjl,6h
xn,6owh,6cbf,6kwf,6pjn,6n9h,6cd4,6xtz,6g7a,6svr,6a1t,6g9j,6mub,6y64,6htg,6
qlv,6gxt,6bmx,6fba,6ugl,6wp4,6dam,6pov,6naf,6dvx,6rq4,6fyq,6lx5,6sw1,6pjm,
6g2a,6sy1,6oxd,6hux,6dzh,6lk4,6as6,6sdd,6d7m,6cg2,6wuk,6qip,6f6h,6coj,6uf0
,6hld,6eiq,6mdb,6u9n,6dsp,6d5h,6nh7,6a7s,6orf,6grg,6fzb,6j0p,6siq,6y88,6to
j,6hxl,6ami,6png,6rl2,6eo8,6usa,6noj,6ub4,6wiw,6np4,6n0x,6pyr,6ngs,6bhs,6q
ra,6ege,6mo4,6gr5,6b38,6frn,6i4u,6h33,6ue3,6s4p,6dum,6i5r,6box,6ary,6nh4,6
av7,6j31,6y5o,6zuu,6hxm,6ffl,6u3j,6r1p,6q63,6hoe,6rh7,6m5m,6pmv,6hcl,6vcl,
6p9t,6tyq,6nza,6a40,6p8z,6be0,6bsn,6cyv,6a15,6r77,6bh9,6vhx,6ex8,6ekz,6qi2
,6ohk,6rnf,6p3h,6bgw,6zlj,6twk,6a0o,6xp7,6azq,6ngi,6pvb,6ry2,6p3m,6ug8,6pw
m,6u3y,6eql,6gpr,6cz7,6bk0,6f3n,6rbx,6pi0,6gnr,6fo5,6qhy,6kb0,6kb1,6hni,6d
fr,6i5k,6ix7,6xp2,6i4z,6uln,6nwt,6rq9,6iyf,6r58,6qz5,6rv5,6bgu,6wjb,6fnj,6
ooi,6ng1,6prt,6fn9,6vjc,6f3f,6ay3,6g5q,6wp9,6or8,6vr1,6dnk,6hfn,6mlh,6df4,
6i9l,6gfa,6mvp,6dnz,6vxt,6jcl,6nh3,6poz,6o9u,6ydj,6bii,6kri,6e7u,6bsw,6t9b
,6c0o,6p4v,6sge,6oph,6rot,6ee1,6rmp,6poi,6p8x,6yb8,6v9m,6mo8,6wt4,6pn0,6c4
c,6qoq,6cmr,6wyq,6rv9,6upd,6g28,6cwj,6t0d,6k05,6ehk,6f9u,6vlv,6bxq,6nv9,6n
w7,6hrx,6zjw,6gax,6tfx,6bmv,6ezy,6sj2,6do4,6fsi,6ung,6y09,6jai,6f4x,6jzh,6
y8s,6r2j,6pox,6dif,6hdu,6t4s,6tom,6v7h,6ox5,6i13,6vo8,6jeb,6e42,6hoy,6i95,
6md1,6kvq,6rcr,6i41,6ma6,6m1j,6j3p,6zuv,6hzy,6axq,6nli,6d5g,6r5u,6bwq,6fld
,6ffk,6dzf,6jz5,6gjh,6lpj,6e3g,6sqz,6si9,6ch5,6xua,6bfk,6czd,6mpt,6b8z,6s4
g,6ft1,6zln,6den,6tbb,6mfl,6gr0,6zsi,6txf,6ph9,6nlg,6ezq,6pmy,6bxn,6c8s,6o
7p,6qa8,6bmm,6eof,6ee2,6ln3,6rhj,6hph,6g8f,6dj5,6lj6,6qu0,6qdf,6e06,6pyj,6
c9r,6nz5,6ubi,6jbd,6chp,6ccq,6gar,6s20,6ca4,6vd2,6a7p,6g1p,6saj,6dyy,6k4c,
6qmj,6lgg,6j3h,6fhu,6c7y,6eat,6p2t,6szt,6h1s,6ypg,6l8p,6b4n,6h4p,6av1,6ufl
,6l12,6qxu,6f6n,6qri,6upr,6hf0,6uoc,6x9a,6b6g,6qkx,6s4r,6dj7,6pda,6npv,6fn
h,6vr5,6txv,6iwn,6mb5,6xim,6sye,6o88,6zyj,6a5y,6kpo,6m96,6u8l,6h12,6nzp,6k
gp,6vrn,6lrz,6hb6,6t0x,6pqz,6hnc,6vl1,6a11,6gn6,6ww8,6miv,6qyq,6h39,6f6k,6
c76,6ecw,6ub1,6btp,6to7,6v7d,6j19,6vsd,6t70,6q2p,6ss8,6dth,6mb7,6pvh,6v1e,
6nxd,6mja,6bc5,6k2o,6lj5,6qvl,6noo,6b79,6vv6,6d9t,6lla,6m82,6qku,6eqs,6hfp
,6grh,6zxx,6bkz,6udx,6qdj,6apv,6sfc,6qyf,6j10,6s4b,6t1t,6s4t,6c8t,6ty6,6nw
h,6dz5,6av6,6eth,6kk5,6ngz,6tbp,6x2y,6ggi,6q2e,6ceh,6rbq,6z02,6vkd,6c7f,6m
ay,6tw2,6cwa,6ekw,6rk2,6lra,6c3d,6ei5,6uxn,6j0k,6wq9,6zu4,6gh1,6cge,6dh7,6
b97,6gl8,6v16,6a04,6mnc,6c98,6ghp,6pwl,6uxr,6kb2,6o9b,6y4y,6w58,6jke,6sea,
6n85,6phw,6ncz,6fn3,6qyl,6ecz,6i7a,6u9k,6exm,6b3v,6bmu,6ypj,6hvh,6u7a,6i0r
,6a2l,6szl,6fnf,6rd0,6za5,6q4k,6ql2,6hmc,6x0i,6pde,6z86,6eq1,6ypv,6dzr,6f5
w,6a6a,6b6f,6ldr,6yg2,6aoc,6jz8,6w2l,6rhf,6e7j,6uqb,6nh6,6mvq,6dyw,6tq5,6x
4o,6evd,6rgy,6qzh,6prd,6g46,6fea,6nji,6gmz,6ngc,6o95,6bc0,6hqq,6pq1,6w9h,6
jd5,6wny,6yk2,6el0,6c7q,6rtj,6t82,6qrd,6a1g,6i01,6yz1,6f7x,6b7c,6u30,6s33,
6o7l,6r8g,6br1,6kx6,6a71,6daj,6rsk,6skc,6g7d,6epn,6wyz,6lq3,6j0g,6mqd,6nxi
,6dwa,6fuj,6vgf,6mdd,6ffz,6gbw,6apf,6ull,6lb2,6wj7,6h29,6qge,6kzd,6nig,6ur
1,6c99,6q7k,6t6c,6lur,6mt4,6lu3,6e13,6ial,6hwt,6ju7,6vny,6i68,6l6w,6t1u,6b
u3,6ubw,6h5e,6b4k,6ony,6r1k,6jmv,6vce,6i5p,6vjk,6sqw,6mtu,6cz9,6t1b,6p86,6
rjr,6ixm,6pn7,6kqq,6x99,6sm8,6fsj,6vl0,6r6w,6ew2,6cvx,6qqn,6vtd,6eo0,6hoc,

6gnc,6v0u,6dem,6rv7,6m2n,6rgk,6xxt,6qxh,6t12,6h9v,6fuv,6c3a,6g98,6th0,6ic2
,6jav,6xx7,6l16,6vpl,6ep5,6jt8,6lj3,6tp0,6cgn,6mqc,6sz7,6at0,6lx7,6bk2,6gb
5,6o4b,6d1y,6ljt,6jr8,6h36,6hpw,6gvd,6bgx,6h1o,6nul,6c9v,6f9t,6h14,6vnq,6n
wj,6r29,6ht0,6kb4,6piw,6xfv,6s38,6g3a,6rrg,6o8u,6o13,6f8r,6ucs,6hmh,6yw1,6
tde,6qcn,6nel,6hje,6r3v,6woj,6urx,6r0u,6qoj,6wvh,6n17,6gcl,6ul2,6h8h,6ekn,
6ttv,6rfn,6rg4,6eky,6bun,6ud2,6i11,6h75,6lgi,6md6,6bgz,6mo9,6k91,6ru1,6dmj
,6hdy,6imb,6chq,6t07,6czh,6aip,6z9d,6cwh,6av4,6qnj,6p0m,6fzu,6dab,6sqd,6n9
7,6kx5,6s0w,6stz,6dha,6gkv,6ecx,6ljx,6dvl,6scs,6jut,6pu3,6zli,6w9l,6dyv,6b
my,6mxg,6s32,6jyn,6vwo,6iil,6kay,6f9v,6rb1,6jk9,6f23,6i8v,6hgq,6gbc,6s88,6
sam,6xje,6bfl,6q49,6ccs,6iwg,6jkm,6olx,6pbi,6wgh,6h9x,6o0q,6rcj,6aah,6oxw,
6ngl,6w3r,6u9m,6rvp,6toi,6pjc,6lrg,6a84,6q4h,6vlg,6mnx,6ufp,6w3t,6qlt,6xxy
,6bc2,6pz5,6a1c,6w06,6asz,6b7d,6qrm,6l89,6ohd,6rjq,6nze,6hmw,6pit,6gnu,6jh
a,6fkl,6kqp,6c6w,6v1u,6bbl,6opz,6a6q,6c8y,6ap7,6k96,6w3k,6nzf,6djj,6uaz,6w
3p,6r51,6icn,6jzs,6h4y,6zb6,6toh,6vaw,6qda,6n3r,6r0h,6opy,6jwa,6fs9,6faa,6
vo2,6qme,6nqg,6dz0,6d1c,6ofy,6sqy,6end,6y9d,6df7,6dbn,6gb9,6ep4,6b5k,6rvz,
6cdp,6zqt,6syc,6b3d,6fqd,6hth,6b6e,6vnv,6iey,6bfd,6h38,6bbu,6rkn,6kpt,6zux
,6oja,6f2v,6nzt,6d64,6nxz,6rw1,6uee,6pr3,6cco,6pqd,6iwh,6wn0,6yvz,6k8k,6ja
q,6ux1,6i14,6tp1,6uyb,6pob,6tyn,6bdv,6pp1,6uvm,6i6v,6k5o,6to2,6hpe,6qfx,6w
w1,6ddz,6ift,6v7g,6y4f,6ykt,6pys,6wjo,6ecr,6c0t,6es9,6t5p,6uuy,6jo3,6smb,6
bn6,6j2f,6bq4,6tht,6too,6fau,6way,6q9s,6o87,6ark,6s56,6p4t,6hgl,6emh,6lzi,
6br8,6tjr,6hpx,6r4v,6l15,6l67,6fqk,6hkm,6i63,6cam,6dai,6fiq,6vc2,6fnc,6h1t
,6ev4,6i4q,6yo1,6hcb,6c8r,6ju9,6rkt,6c7g,6jq8,6oey,6eoe,6m8n,6ggc,6wq7,6ev
9,6p0j,6b6c,6gg9,6tto,6p5o,6aci,6k0n,6aus,6b3h,6p4m,6pf5,6plf,6a2k,6nll,6f
2u,6kju,6w7i,6eyb,6rj6,6hyj,6mv2,6i3d,6o4i,6kxm,6l2q,6faw,6nyv,6nm7,6geq,6
tfs,6xpc,6s4n,6vuf,6uoz,6c4n,6qq4,6fmz,6o7s,6g3j,6qce,6vrv,6fi5,6p8w,6owe,
6eq8,6f8u,6w9r,6dji,6vgc,6mx8,6bup,6w65,6ka5,6gg7,6ajx,6ge7,6xpw,6av3,6pwg
,6s1v,6suy,6gdq,6vk8,6qoo,6be3,6dub,6o0k,6dw7,6ccy,6avi,6ru8,6o48,6cb2,6t5
q,6r41,6eu5,6q17,6nw3,6stg,6s03,6o7q,6ayv,6eza,6n3x,6pg9,6ur0,6r8l,6yte,6l
8h,6c02,6q9w,6cpw,6m9b,6zyh,6fyz,6qhw,6ew7,6y8n,6a4d,6dn7,6phv,6ilt,6edd,6
r3u,6ya7,6rcy,6hwr,6fam,6dtf,6mqe,6cec,6r71,6go4,6cgg,6f8o,6bg5,6d28,6hfl,
6mnv,6rzx,6moa,6zun,6qzb,6sv8,6o4g,6fgg,6duh,6v63,6c0j,6o4f,6qdx,6ew8,6v1f
,6i7f,6kb5,6t01,6xvw,6t7p,6jc8,6mqg,6vus,6dn6,6s5j,6enm,6jt9,6eq0,6i0p,6uz
r,6p10,6p9p,6loq,6bz2,6gh4,6tr4,6uq8,6vs5,6gss,6quu,6kba,6f30,6mvm,6fnu,6c
ef,6gqr,6pyz,6nnh,6ub5,6ha0,6x4q,6riv,6tuk,6lx4,6qi1,6miq,6s23,6v9n,6ypk,6
vei,6i4p,6jdl,6im4,6jop,6efg,6hf3,6e43,6t0c,6mre,6pxh,6mb6,6r68,6nse,6cqw,
6ny7,6aaq,6qsi,6q2q,6ko6,6cmj,6f76,6sbd,6cxf,6q4z,6sdk,6rg9,6ahs,6ftn,6dh5
,6oof,6p0o,6qhu,6uqm,6fx1,6u3k,6bk1,6vsp,6min,6opu,6msn,6szy,6eln,6h1d,6ly
b,6osp,6thx,6pib,6p4q,6djh,6en5,6p9b,6s54,6i3u,6nmq,6nol,6h0b,6kz8,6yn0,6x
jm,6w8l,6c4a,6b96,6s0s,6rjn,6l1n,6eed,6qyp,6p9m,6vst,6uez,6ptb,6vty,6dl7,6
wfo,6ci5,6b4l,6mvu,6arv,6few,6n79,6t08,6tow,6dy7,6y4z,6j5x,6fo8,6kjp,6qos,
6e5a,6p3p,6eno,6kb6,6dim,6t5w,6hbn,6d31,6ezc,6yko,6mb4,6wvi,6u7c,6cd5,6pgj
,6j1j,6wp3,6hu2,6t0o,6u7o,6o80,6rpb,6iy9,6szr,6gob,6l21,6mdf,6ae3,6g1o,6ji
d,6nsv,6a4f,6vza,6eaa,6bn5,6rx8,6cdn,6xvr,6asu,6nhx,6j27,6fod,6tdh,6gj7,6l
jq,6fqu,6zdv,6w39,6rq6,6o4c,6wp6,6oa1,6pwh,6ch6,6qr2,6n7d,6m6p,6fw2,6gyt,6
oex,6gi9,6v0s,6k35,6n3v,6k0u,6bqx,6nzb,6w02,6fgt,6qad,6qba,6xug,6u18,6gq6,
6j7e,6u1v,6hfv,6fof,6pj0,6j7u,6j38,6gex,6byt,6gx8,6gsp,6c0l,6bky,6hrt,6rh1
,6aaz,6e1q,6v0x,6bdr,6ljv,6o11,6ryk,6zy1,6rki,6nwk,6dwd,6asi,6bkk,6gdy,6lj
2,6wdw,6yt1,6hrp,6nba,6qhg,6lsd,6gd6,6e4v,6xnp,6i4d,6tdt,6rjy,6i4r,6ulx,6r
m1,6qjv,6dch,6v9j,6xvc,6pg1,6das,6c90,6tos,6n69,6ho5,6gf9,6ckr,6jz4,6ajp,6
p3n,6f6g,6lio,6xhc,6eve,6viw,6n94,6yhr,6x2x,6rsz,6t4o,6hzv,6d1f,6ymf,6g3i,
6db3,6rr3,6pnd,6y3b,6kcx,6pn9,6qeh,6vzd,6r11,6fnm,6rup,6gu5,6wf5,6ydg,6rxr
,6mxc,6k0q,6fp3,6rxq,6s7s,6g3q,6j8r,6fnr,6ssq,6pj1,6u2s,6hq8,6ap8,6auw,6oe
z,6snn,6o0j,6o70,6nsf,6quv,6pvy,6z9b,6g1u,6gj8,6r0q,6xze,6ndl,6ywn,6tsc,6a
6m,6dmf,6mbu,6h4f,6guj,6rhc,6bnd,6qa3,6gga,6fty,6jmx,6hto,6mt9,6hs1,6lop,6

urw,6i65,6d4u,6ox3,6fyl,6cvy,6n92,6sr6,6c6n,6hnw,6vmz,6j9y,6ab1,6omr,6o6f,
6gih,6bmh,6mzq,6fyc,6dug,6ggf,6m9m,6czf,6ced,6g1g,6kx3,6k2n,6c5j,6bgk,6ojx
,6el6,6qr5,6hqp,6ci4,6id4,6wgn,6n4m,6ghl,6wuw,6b9e,6vrz,6fgy,6a1f,6bj8,6i2
q,6wmo,6h17,6pp3,6gr1,6rga,6p0l,6amb,6ix5,6hq7,6rzq,6dn8,6gb3,6k8z,6nni,6s
vb,6f1n,6fmc,6oq7,6xui,6fjk,6oiq,6gd0,6bwl,6vdf,6zwb,6qj5,6fvl,6xzs,6d61,6
yv2,6pp2,6qvt,6mdc,6kla,6rcw,6fxt,6pou,6b9t,6g3r,6uex,6ykv,6t5u,6ftc,6ecg,
6sun,6kmp,6c5q,6x3p,6whd,6o65,6ab0,6sv0,6kjr,6t5j,6f8j,6f71,6a17,6oc0,6jom
,6el7,6j5l,6lvk,6hpf,6f5u,6jbr,6npp,6tog,6bh3,6uky,6lor,6ljn,6yus,6s4o,6qk
r,6hro,6uzw,6ck2,6l6a,6hme,6aqj,6ha4,6mb0,6ix3,6i6t,6tep,6nn4,6uzu,6fmu,6l
2c,6qrg,6sjo,6zwp,6pet,6q8c,6e0b,6us2,6jsg,6h6x,6nor,6i16,6v7e,6tam,6g96,6
oir,6hh6,6bym,6s0e,6rjj,6ka6,6mhe,6rg1,6z1n,6ydm,6m4u,6g3c,6vzb,6ifd,6gwb,
6lf6,6ly4,6fzg,6dbz,6ljm,6bg0,6akw,6oou,6vtb,6s83,6w01,6s79,6vcn,6upq,6hxu
,6gnt,6xxz,6on4,6j2h,6wbz,6rq8,6f4f,6vpm,6pcc,6k9z,6svi,6xva,6fl4,6pdf,6py
w,6s7c,6yvw,6i75,6kvp,6l17,6jnk,6pot,6uob,6jb0,6mau,6sjj,6q7h,6dio,6s4q,6c
hm,6kcn,6hkz,6d55,6g6y,6btn,6ej3,6gpu,6ld2,6lya,6p7r,6o5t,6nzm,6yqn,6b9b,6
wq4,6g74,6h34,6sbo,6ul0,6ypn,6pf1,6bdp,6oy0,6lj4,6lq7,6zj9,6tg2,6b3t,6r1n,
6pk2,6ngj,6bzt,6vjf,6exq,6e5t,6tp5,6o0f,6ooh,6e6m,6tx6,6l22,6ge9,6zll,6t74
,6jvc,6jap,6mvo,6fz3,6uw1,6ba7,6qrc,6god,6nwl,6bgy,6gw0,6jls,6fc1,6l6b,6nn
o,6ny4,6xuq,6q7s,6a2w,6pl1,6fk3,6a9o,6s43,6n9a,6rkg,6gfk,6eir,6c7w,6ald,6e
v6,6kb8,6g12,6zwr,6m88,6vl4,6cep,6ppw,6r5n,6uoi,6sd7,6md7,6rwh,6a5m,6l1o,6
dq8,6ep7,6dur,6gba,6bur,6gta,6kzz,6c0n,6ad9,6rcx,6yk3,6f8v,6eqz,6rbw,6uql,
6rrp,6qya,6kqs,6b7b,6sv5,6xoz,6rft,6el5,6dj1,6wfg,6rv8,6k4d,6knz,6dv4,6hkj
,6jah,6fpl,6rff,6uaw,6jpw,6jst,6oos,6qmd,6slo,6tfi,6p7d,6qz1,6gql,6zdr,6b9
r,6jy4,6dyz,6p5r,6ee6,6adi,6fyp,6e5l,6mo5,6eis,6h51,6t5r,6ng7,6exs,6s70,6g
01,6jvw,6ff8,6h4z,6csc,6hmy,6lit,6a3j,6eg9,6c1x,6yqi,6mcz,6i78,6rkx,6k0h,6
rj3,6stf,6ede,6bms,6smr,6o9a,6f20,6k04,6mgc,6sbt,6ilh,6anl,6r8q,6ili,6st0,
6dh4,6e47,6jil,6hgs,6ihk,6x7b,6q6y,6rj2,6s6k,6hbd,6wug,6j29,6pyy,6g6t,6m8d
,6pu7,6tgd,6vz9,6ngy,6jfk,6b3p,6d6w,6e4d,6mak,6cjr,6w7f,6bgc,6gos,6hry,6sv
9,6zly,6onf,6ico,6hf4,6oxp,6t2h,6w3o,6rs9,6x3r,6n0b,6mnp,6vs6,6cx9,6g1w,6x
p9,6abw,6np8,6j1e,6dwj,6b36,6qu7,6zug,6i8m,6oxy,6hs2,6hmg,6pvj,6j7m,6q54,6
h3q,6cel,6kzv,6u2p,6giv,6oqc,6i79,6l18,6enl,6vif,6g9m,6mob,6axb,6aim,6krs,
6sq9,6if2,6v0q,6piv,6w9i,6gcw,6bja,6qvo,6tld,6vv9,6qxq,6uiq,6mod,6w1i,6g0z
,6dh2,6uce,6o08,6zv7,6cvd,6gip,6flu,6fuh,6tor,6d17,6m83,6t8m,6tp6,6k0r,6b6
t,6uvk,6hqo,6f8n,6pzr,6rzr,6l8j,6qsa,6pjg,6ojk,6pgp,6nln,6die,6lik,6tt4,6m
dv,6a87,6ct5,6u3f,6lgf,6uqd,6pah,6loy,6k8t,6moy,6gjb,6esu,6hwl,6f3g,6dgx,6
eva,6pr8,6r5e,6jy3,6ey9,6zv8,6v2e,6l7h,6zay,6p9k,6aa5,6y9s,6uqw,6qlr,6fii,
6i4f,6no0,6fek,6lql,6ny0,6hgv,6ma4,6nop,6mia,6a23,6eqv,6h6r,6p85,6x8e,6ct2
,6m6y,6pjb,6ch4,6knh,6i4i,6rj5,6e8p,6ngp,6iar,6ggn,6i4l,6vlm,6gu0,6x5r,6a6
3,6pye,6dvh,6g5u,6hqy,6trn,6yv0,6jxn,6j34,6o2z,6flg,6seb,6m7i,6yqq,6bgv,6v
s8,6uo3,6pva,6fl8,6oi2,6hai,6hsg,6chh,6ewr,6d1b,6udu,6wfn,6zld,6lq0,6d1u,6
uvj,6lki,6i96,6hgk,6t6x,6eol,6wxz,6hax,6nfi,6lx8,6a06,6yo7,6dyt,6mu7,6n12,
6nco,6wbj,6fr2,6au6,6ecp,6qov,6hqs,6uxp,6wmm,6rl9,6bvm,6gtv,6jk6,6hza,6qw5
,6poy,6yph,6n6n,6rqk,6szg,6bdy,6q9y,6f86,6ev5,6zio,6nvu,6fna,6a99,6qii,6av
2,6zrn,6rbv,6g09,6xce,6n5c,6cx5,6l1q,6qlq,6gfz,6q60,6qbh,6nee,6pdh,6ljr,6h
rz,6des,6mji,6hu1,6oi1,6nx7,6ll8,6zj0,6dh0,6buo,6d4s,6jb4,6fhq,6fs0,6oxs,6
kx7,6j24,6fqh,6g9x,6rkh,6oo6,6ngt,6auy,6mt6,6bq6,6vyn,6ocn,6sqb,6cqf,6bt4,
6hez,6pdj,6s9e,6jao,6ngd,6uh0,6pfo,6nhf,6i8z,6o0o,6u7p,6eji,6qr0,6gx3,6ngk
,6i3s,6ijb,6qfq,6g4e,6r4c,6rm5,6hh2,6k0i,6jjr,6gi6,6sll,6if3,6g8x,6izq,6v9
5,6i61,6nh1,6wpg,6dih,6cqy,6z6i,6wok,6hhr,6nzc,6pj2,6eqp,6pg3,6rfe,6zjb,6o
jb,6s3c,6r7w,6l1z,6jjb,6us3,6yqp,6s6d,6yd9,6vlc,6vxq,6ex1,6qjx,6fin,6wrq,6
ael,6df1,6onz,6c7n,6ky9,6vuj,6lg2,6h2n,6dnd,6xzo,6u8o,6n8x,6yqj,6eop,6gpy,
6npi,6f56,6sls,6jmy,6ljj,6o5j,6lri,6wja,6sps,6r5v,6b4u,6e4a,6b5q,6txx,6spt
,6fkd,6uek,6fux,6e2m,6piq,6ttx,6zjd,6ggg,6pzo,6cfv,6bka,6fbo,6py0,6lub,6ta
n,6b1k,6fd6,6ugp,6udi,6xnv,6no3,6rz2,6wpl,6dyn,6k0v,6dxg,6b0v,6nkm,6b83,6d

7f,6a21,6t10,6poa,6nz9,6qo4,6upm,6hli,6tm3,6i20,6jn7,6tq4,6os5,6csp,6gu6,6
w50,6s1x,6ytd,6qb9,6q4i,6dys,6o1j,6eab,6got,6xk0,6fdf,6ffi,6p4r,6l1g,6sdj,
6max,6gd4,6cbz,6sbg,6apz,6iw6,6f4k,6lj0,6gxw,6tzm,6jam,6qe6,6sb7,6oqy,6sdu
,6fne,6iwo,6opv,6q8b,6wzy,6f8w,6spn,6gcy,6cyg,6u8b,6p8y,6h50,6hd4,6joq,6ek
q,6aac,6qaa,6bds,6rm7,6qxg,6mqn,6pi1,6jwy,6udv,6nmx,6b69,6oqx,6fye,6d4v,6s
yz,6ngn,6nyw,6sz2,6es8,6j4t,6evc,6fym,6mio,6w1b,6jw7,6fnx,6e2y,6k3n,6gon,6
g05,6spj,6a1p,6tck,6pgr,6cbo,6hjg,6dtn,6kxb,6v7c,6e20,6s7k,6phz,6pgd,6cib,
6ig8,6epo,6e2a,6h4o,6nv2,6f96,6ly6,6m92,6jo1,6loo,6bra,6frf,6b1c,6bs6,6onh
,6ehh,6j2i,6d59,6q0d,6bfx,6p9e,6ax9,6tcu,6u4q,6lk2,6ps3,6a13,6fs7,6xpu,6bh
1,6of5,6l57,6o24,6saf,6pk0,6ils,6za0,6oxo,6gxd,6jc7,6q0z,6c2i,6vsg,6qod,6h
qd,6tgu,6el4,6bs3,6xtk,6daa,6tvs,6kxh,6vij,6fh6,6hh5,6hsh,6gb4,6ts4,6low,6
vcm,6f8x,6s5q,6o15,6aec,6s0u,6ce8,6vru,6uhz,6bkq,6abx,6qdc,6p0k,6hil,6gna,
6ks1,6ti8,6bfh,6lam,6o4z,6we6,6s4k,6qxl,6tgl,6g9a,6nne,6zhz,6deo,6p5u,6mpa
,6aid,6p84,6req,6ev8,6gns,6cjf,6mvn,6wyp,6hoa,6hr2,6trh,6q3y,6gay,6oxq,6cf
c,6g36,6mwh,6um8,6ho8,6pya,6gy1,6pjf,6nxj,6bpv,6a20,6qr7,6eut,6kdx,6qr4,6x
j8,6pbp,6fxr,6nuj,6ktk,6yw4,6vcy,6oxc,6nic,6uqy,6a0v,6wq5,6rb5,6ngr,6vkv,6
d1h,6qli,6ftz,6rri,6w9k,6cts,6bbs,6md9,6ut0,6ybk,6e6k,6jz7,6i23,6sv6,6gfq,
6yxi,6kv1,6hny,6ufc,6q4a,6fni,6g2r,6dtg,6gty,6o6o,6gh2,6gqj,6hly,6lkj,6vuc
,6g07,6no4,6b42,6a3g,6ltv,6s1e,6xvt,6ryz,6f6f,6uj4,6hob,6r49,6nhz,6exl,6p3
o,6eya,6fgq,6ujo,6k0s,6mqf,6pga,6pje,6gb7,6j28,6ne5,6ykx,6ter,6uo5,6wyo,6u
ly,6tmo,6bhv,6xcf,6p2i,6pgc,6p6g,6aud,6bsm,6ezf,6vhw,6xu3,6ms1,6s3b,6htz,6
mom,6nna,6d5v,6xp0,6e0q,6b5e,6gnm,6b89,6hzu,6ukv,6gtx,6juk,6d4t,6p2b,6tfb,
6nws,6m9l,6ikg,6phx,6qhz,6sde,6pn4,6c4m,6pd2,6u2n,6yo9,6pvw,6t06,6hmq,6pdc
,6r4f,6tp2,6bhq,6d6c,6fi4,6i77,6bpr,6vdb,6c5k,6udy,6j9j,6qqq,6t5b,6o6g,6b6
p,6pc9,6sb8,6nso,6fog,6fnk,6bpx,6peb,6ew6,6ru7,6z89,6b92,6v90,6vs0,6ncx,6f
kc,6a0y,6dw3,6f4l,6a08,6cdr,6n16,6jpl,6nw2,6k0j,6u0p,6qei,6wjy,6eda,6to0,6
jib,6hl1,6yb9,6eur,6d3e,6tx5,6mj5,6ht1,6hk4,6o5a,6en6,6yz0,6cwn,6ba4,6r0p,
6ho1,6fvz,6hm2,6qqr,6s02,6dxl,6rg7,6d0d,6cff,6pq8,6e91,6h7d,6pq3,6xd7,6np5
,6d1z,6cay,6pte,6ksb,6ris,6o4x,6rmf,6onx,6rl1,6zuh,6qht,6ofq,6b4d,6p7u,6nb
w,6nnu,6vql,6efj,6sq5,6cig,6a1m,6ayi,6sq0,6h0h,6w4g,6how,6hsk,6ild,6ygj,6j
26,6viy,6qqw,6k9h,6hql,6rt2,6o04,6xec,6hfh,6xdm,6asq,6fnt,6q6m,6slf,6fi2,6
aa1,6g4b,6ti9,6rnk,6t6z,6a72,6jc9,6glb,6qga,6i55,6ngv,6ngf,6q9u,6izf,6tt3,
6dyu,6lp7,6gnw,6far,6q3f,6j1w,6mb9,6fil,6o4k,6i82,6ec8,6fu4,6o98,6s8y,6rkq
,6akd,6q4f,6pmw,6i4b,6c28,6fbz,6cp4,6ihd,6haz,6b80,6r18,6ehu,6qqj,6rvf,6qq
u,6hpy,6ma8,6y12,6rbc,6l6e,6wui,6rsi,6vav,6gfo,6h4s,6vc3,6qo6,6dvn,6uli,6c
8x,6j8w,6b3a,6byf,6qfu,6f3u,6fjj,6ycw,6qnc,6cs9,6wlf,6siw,6jmw,6bs4,621p,6
cct,6tzo,6yqo,6dqr,6pay,6dgl,6z27,6phr,6d1j,6tnr,6xu5,6t04,6pr7,6npm,6o6y,
6yv4,6v7j,6aps,6pau,6dq6,6mvh,6lkk,6eil,6r0i,6rbs,6n0y,6nnt,6svj,6ykq,6sin
,6g9u,6cw9,6zn0,6v6z,6s40,6tx7,6u34,6f68,6omq,6hnx,6t3q,6a1r,6n9r,6nc1,6us
u,6nfh,6f8a,6kx2,6slg,6e48,6v7f,6ay6,6r5r,6gsy,6tug,6lgc,6k24,6hfq,6rgg,6n
mb,6y8e,6i5g,6qyo,6d3u,6v2f,6qlk,6kzc,6sz1,6e2o,6abl,6vim,6laa,6cza,6hoi,6
gfr,6lj1,6gtw,6u80,6ayh,6y5m,6l8a,6s9g,6a0t,6y6v,6ijp,6eox,6qab,6fcf,6up4,
6d6l,6sq7,6r9x,6sw2,6cw7,6m9r,6gge,6czv,6c74,6hpz,6ppz,6u65,6h4v,6oah,6r0s
,6faz,6j2j,6v40,6c67,6ax4,6i5y,6qas,6qol,6ten,6yem,6sux,6s31,6ysk,6fby,6s9
q,6v2q,6svx,6dyr,6bl8,6d5w,6c9p,6vd0,6bu0,6g95,6qok,6n42,6sds,6qom,6n93,6n
43,6rqe,6gnp,6ktq,6cz4,6bfw,6m77,6pvv,6gu2,6gdo,6def,6j61,6dgy,6rpu,6add,6
ooy,6gvf,6uyc,6izw,6nhd,6asy,6x3o,6hg6,6evm,6q4j,6rt8,6bjb,6ilc,6ong,6d20,
6n4e,6bh5,6tuz,6te2,6mna,6fwh,6g9i,6gnh,6djd,6b7a,6yku,6o50,6xps,6oz1,6v1h
,6s5k,6nlj,6ql1,6vly,6up5,6lge,6alj,6b98,6dxs,6qkj,6mlf,6ook,6z5t,6b6s,6do
3,6ps9,6oog,6qe9,6gvz,6m7x,6b39,6x1z,6qej,6g3h,6pn1,6b9p,6v5p,6uvp,6ba2,6a
i9,6fzf,6r8p,6r3m,6i66,6ceo,6orh,6nzv,6pgb,6pu8,6ci7,6axn,6duk,6fh4,6xvy,6
a98,6zgp,6ubb,6br4,6cu6,6hqt,6pi5,6oe1,6gbf,6h6k,6dh1,6gd7,6x2m,6c17,6a9c,
6zjz,6gxa,6rcb,6nsu,6lud,6phy,6ob7,6xlp,6bg3,6ew3,6ses,6qb4,6fqj,6mjj,6dd6
,6v62,6fd4,6mfx,6q6o,6o7y,6oi9,6kjz,6cid,6eqm,6fr0,6lpl,6evp,6egz,6suo,6rk

0,6eng,6cho,6ury,6gy9,6ce6,6mt5,6g9b,6r0f,6csr,6e6h,6dzq,6n9q,6emu,6agz,6p
u0,6aux,6vj3,6e0l,6ne7,6ko7,6p7k,6cbl,6qhp,6a0d,6fbb,6l6d,6v7a,6qog,6c5u,6
dfn,6viz,6lah,6lue,6zsj,6h8b,6i90,6jws,6gk6,6afr,6hm1,6tz7,6uak,6sti,6uau,
6p3t,6p0n,6ej2,6pmz,6vub,6etd,6fmd,6jyx,6xp1,6esm,6o1t,6g5l,6piy,6fn2,6jdi
,6a2o,6rka,6cyi,6ewz,6li7,6qff,6vch,6fch,6vcq,6p9v,6v5n,6qr1,6x4m,6a0m,6dq
a,6eze,6cgp,6bve,6sud,6xv3,6o3p,6i0b,6r8w,6s4h,6hku,6qlg,6p2o,6bc4,6nns,6n
7c,6piz,6a1h,6w6y,6hlj,6nnd,6lkg,6c7x,6f69,6sy3,6o4h,6vld,6q9n,6r0k,6i64,6
hqm,6vwc,6sug,6s7a,6hcw,6mae,6pow,6qk1,6fyk,6b9d,6cbm,6ijn,6hu3,6guc,6rgr,
6fsg,6ap6,6x5l,6zla,6kb7,6rk9,6n3z,6tns,6gdp,6oip,6njh,6npe,6s52,6pjd,6zad
,6ks9,6b9i,6cr2,6v1p,6tge,6qhv,6hur,6rfi,6xeb,6aan,6gcb,6pvg,6prb,6t0z,6qt
j,6tr7,6qgb,6i6r,6vk5,6vnd,6w8h,6kkb,6ng4,6rkl,6p14,6hh4,6g9h,6ci9,6icp,6p
05,6kpm,6aln,6mp9,6rnt,6r1d,6ebq,6rje,6lmi,6v52,6dh3,6npu,6cjv,6e99,6y4d,6
i3c,6g0v,6opw,6bfo,6x8g,6hjo,6ii7,6o7n,6dvi,6wv7,6qdb,6rht,6vcp,6exk,6uas,
6hmk,6jyg,6gf3,6hml,6kax,6bum,6o9y,6w9m,6oo1,6ouw,6qkg,6w3z,6hmr,6e8z,6t4a
,6rfw,6o66,6rl6,6pcd,6vjl,6ay5,6goe,6myw,6gpt,6mi1,6u1h,6q73,6t02,6tek,6sa
q,6qqs,6fpu,6r6j,6del,6ueg,6cn5,6mh1,6hzb,6c85,6p8k,6jd4,6s55,6d1i,6cwf,6x
u1,6r7d,6p83,6or4,6ax3,6m8a,6auz,6wnb,6pk3,6bk3,6tr6,6oyr,6c5f,6edh,6bh2,6
jqb,6rp1,6pgu,6gcq,6e3a,6te6,6acs,6qoh,6rhk,6svd,6maz,6pck,6s0g,6bqd,6hm6,
6fyj,6exj,6x2s,6dhu,6ju1,6ihe,6rke,6cgz,6onk,6s37,6swx,6nnc,6e2b,6pmx,6i0z
,6qon,6tof,6hnq,6f82,6qqh,6qgh,6xxi,6dmi,6ya6,6ghv,6usx,6bqj,6fd5,6rgd,6ht
8,6tv4,6wk1,6vlo,6pr4,6ugr,6n19,6mlw,6l1l,6daz,6idg,6po8,6qjw,6f4w,6gqt,6u
b6,6obx,6r6y,6uwv,6abm,6f6u,6rp3,6nc4,6pjh,6kko,6hod,6tbo,6r1c,6i6f,6p8l,6
aeh,6asn,6cck,6bks,6deq,6er9,6f66,6bto,6rm9,6rh2,6knk,6zju,6gcp,6hch,6p11,
6rqb,6mfe,6dxq,6mju,6shu,6mj6,6ab2,6rbu,6sw0,6yo4,6pzu,6iyk,6qk2,6sz3,6n0q
,6fs1,6n82,6f2y,6gdm,6ulk,6ie8,6uo2,6cfg,6wgx,6x2u,6giu,6ggd,6bl0,6czs,6bo
f,6rbm,6xb3,6m24,6qz3,6jsf,6mcr,6p6k,6qs5,6q4c,6wpu,6o4e,6n8y,6klk,6j7k,6u
gn,6yk6,6df6,6m9w,6fow,6d1k,6qrf,6kfz,6tpf,6f90,6f8t,6hnr,6cul,6kbj,6yuq,6
gmr,6x9b,6f3j,6bh0,6lrc,6e7r,6rmb,6iam,6qmc,6fkw,6vv3,6pcl,6qzy,6djf,6w1k,
6m90,6bld,6s77,6ow4,6fbp,6rts,6bj3,6y8y,6u6w,6uw5,6qz8,6q9c,6fho,6fui,6apl
,6fav,6kxx,6nlk,6gze,6dfe,6srd,6ngu,6i6p,6bp1,6zjr,6jme,6tg3,6ea2,6vxe,6k9
s,6h4q,6sz8,6cxx,6eyt,6a0s,6rb6,6ag4,6zpd,6u9a,6qaq,6exi,6qio,6rnb,6u67,6e
l3,6j39,6quw,6bkt,6dqu,6g14,6v8k,6siz,6i4g,6fhx,6md4,6xpv,6s57,6f48,6p0z,6
ttm,6d5m,6b6r,6t0m,6mp7,6jx9,6hyh,6pic,6r7k,6kj0,6aic,6ynr,6ql3,6hdq,6vix,
6qni,6cpa,6nrj,6bxl,6yg4,6au9,6ej7,6auv,6jmf,6l24,6frj,6r2d,6bq7,6u37,6sjz
,6oy2,6gnf,6rjl,6orr,6ibb,6ova,6kpn,6hsf,6lmq,6g1q,6s1z,6r5t,6jdk,6bo2,6x3
n,6yap,6ee4,6f1j,6jrx,6slw,6evb,6gqx,6rob,6rb3,6dq5,6m93,6j4h,6b1i,6rlu,6y
20,6hxo,6nbe,6ewe,6brf,6yks,6uqz,6rtu,6aaj,6jwb,6bxk,6sul,6k92,6equ,6mor,6
i02,6y73,6w1h,6fa3,6ukz,6uxl,6npb,6nyo,6wfk,6qhx,6cug,6pj4,6fw0,6gx4,6ezg,
6jp3,6rhm,6jt7,6p9s,6s04,6ry1,6eev,6i4j,6yk5,6r6v,6cc0,6lq6,6jbe,6zqs,6wmn
,6hah,6tnc,6f88,6sdt,6hxi,6ooo,6ss7,6t5y,6po7,6n33,6mw7,6iik,6f05,6mwz,6cj
9,6i8x,6epz,6rz7,6ofr,6mds,6p5v,6hb7,6vse,6os6,6p9l,6z9i,6hd6,6cke,6gi7,6r
12,6pbn,6uzg,6wyc,6g2s,6gz2,6rzs,6z10,6wnd,6xzy,6hwo,6ey8,6efv,6v2o,6bsl,6
mwe,6sj4,6q8j,6sjc,6jqf,6bxm,6qi3,6rpn,6jxl,6cdj,6di7,6qnb,6cd7,6nzy,6g6r,
6htt,6i0s,6lj8,6hla,6nrg,6hot,6qed,6pf3,6gj4,6f77,6pvc,6rc2,6ha3,6svn,6hu0
,6gsv,6p7z,6qk0,6qkw,6t4p,6x4p,6nsl,6av0,6ksa,6ock,6h4r,6tgx,6lis,6hg3,6l6
4,6rnc,6h4u,6voe,6b59,6t05,6n5f,6h5w,6ovx,6ygb,6o36,6rjo,6jtb,6f01,6a19,6x
nn,6opd,6g34,6czi,6sbc,6qdd,6a64,6hay,6dki,6g5j,6f4o,6zir,6v14,6tim,6snx,6
jay,6tgi,6c9s,6npr,6nea,6gr3,6hov,6brt,6kjq,6f0w,6kz6,6k2z,6t5d,6po5,6cuo,
6r6f,6o6z,6i4h,6qfh,6hlp,6b6u,6hgr,6cdo,6qkt,6i5h,6sao,6jkf,6vtn,6v1m,6ckw
,6fzh,6rtg,6fcd,6exo,6fex,6d1m,6q74,6gr7,6elp,6ppx,6y78,6am8,6iwp,6jar,6ob
5,6jux,6ho2,6ep8,6n3o,6nnp,6qhq,6evf,6rr2,6kpr,6muj,6vhg,6uwp,6t2i,6b74,6p
eu,6ewo,6f6c,6l6x,6kro,6t81,6zje,6ajy,6oa3,6j1g,6vkk,6vnx,6uqx,6rgc,6ac9,6
rkk,6ry7,6di0,6kps,6uxq,6c4g,6epa,6ogt,6i5v,6cdm,6v9f,6chl,6ho0,6q4g,6c2z,
6hqn,6aqs,6qi0,6liz,6fid,6hus,6g83,6f6e,6rms,6d1e,6ync,6ypf,6j2d,6uzx,6nnr

,6ee3,6typ,6o47,6veo,6g15,6qqt,6ng8,6sjy,6q0n,6akr,6d4r,6h3d,6a2n,6z72,6x2
p,6ma7,6a3i,6fio,6gsu,6y55,6cm2,6dmk,6o6n,6e23,6p29,6rkm,6tel,6gzd,6a6u,6p
8a,6mkl,6de2,6n84,6cs8,6q93,6dik,6ra3,6edg,6xya,6r2c,6fz5,6uwu,6ocj,6sk4,6
h96,6p4n,6h8s,6rs5,6gkz,6fkp,6mgb,6xex,6dlx,6q4r,6g9k,6r2u,6uxm,6vpi,6e8i,
6ak6,6nga,6koj,6oqq,6c1s,6np2,6gjg,6sbe,6vc4,6vg5,6i2s,6aa4,6si3,6eom,6gqs
,6xv7,6yvx,6vov,6gmg,6aa8,6w7g,6alp,6xe4,6ezb,6acn,6h9w,6gjk,6da9,6zac,6sp
x,6i8t,6d5l,6gwr,6umk,6a0j,6ibz,6goo,6vii,6o5x,6i83,6rbp,6z4w,6a05,6gw1,6c
bg,6uhy,6mry,6o0m,6gpp,6p87,6jaw,6ya5,6vhn,6l59,6wk9,6te4,6ug0,6o7m,6y80,6
ojl,6oid,6spr,6bbv,6vrf,6i6g,6s5m,6gla,6dko,6t6w,6cyd,6r3s,6rqi,6g3o,6rbo,
6t8t,6m95,6ngq,6lf2,6da3,6c9n,6css,6r00,6l6o,6u1y,6svv,6cey,6g97,6tkv,6x0j
,6qqz,6gfs,6oo8,6et4,6hde,6ts6,6ev7,6i10,6ccm,6bxp,6zyi,6omu,6b6d,6imd,6gn
v,6v4c,6f9q,6br5,6f5h,6ogq,6b6n,6pnf,6dcy,6ho3,6rwi,6bm5,6p89,6dgq,6nzq,6r
p6,6cie,6ow2,6tei,6llc,6lpk,6u4j,6m81,6rcm,6hxe,6gpf,6t4e,6pr0,6jax,6vw7,6
o7o,6tvl,6mp8,6cyc,6k8u,6geh,6a0u,6bfg,6q37,6xj4,6oxr,6kk6,6hrq,6pi6,6nfy,
6rg8,6l68,6y8b,6wkj,6g3v,6tvb,6x4n,6sdc,6p02,6qnd,6usz,6ydc,6w90,6rq0,6n10
,6e08,6rvk,6a7e,6tvg,6rbr,6lp8,6zfm,6o86,6yna,6tu9,6fhk,6gxq,6jz2,6b2m,6hb
z,6qmr,6wo2,6vkz,6fzr,6qhc,6co9,6myj,6qoi,6fe1,6dax,6fv4,6r0g,6jyu,6tj0,6m
4p,6rbl,6vh6,6ykw,6jwq,6qgg,6slv,6txo,6kk0,6i5w,6rbz,6svw,6ly3,6f1x,6d6n,6
r73,6juu,6pia,6hti,6scz,6pk1,6q67,6j23,6xxj,6nj0,6op4,6fyb,6d5x,6xuz,6vnr,
6lgb,6jan,6mta,6rlt,6tr5,6gsw,6hm0,6tzk,6nes,6prf,6jmd,6ujl,6sgf,6r2b,6maj
,6prg,6zuw,6k90,6ojp,6cxu,6ft9,6oqn,6qor,6b07,6j1o,6qo3,6oe0,6uvy,6eac,6i6
x,6o57,6hlm,6ge0,6gjd,6pe3,6b6a,6ft8,6g9d,6t71,6k7t,6pvf,6c0k,6r9u,6ewf,6o
46,6nge,6qow,6one,6nkk,6f5v,6ict,6e7t,6u5l,6rjm,6ugo,6rk7,6ess,6n3w,6fyd,6
qea,6r1a,6xta,6fa1,6fcl,6piu,6lrd,6geo,6b68,6fzj,6ucf,6qre,6cjs,6fuk,6tdg,
6n4n,6fz2,6mde,6vik,6f81,6jz6,6msc,6mey,6q3z,6dcg,6ybj,6c9q,6fg6,6q9o,6dbm
,6qa7,6hwz,6nt2,6p3d,6zi7,6gmd,6ob3,6nxb,6t7z,6lzj,6qav,6fkj,6qmm,6fnb,6sy
i,6yw0,6pdg,6e0r,6tj9,6oic,6g1v,6m8b,6hsj,6mtl,6a9f,6f3s,6p13,6szn,6pli,6n
ci,6t4c,6gxu,6r0t,6da5,6at2,6vvb,6gjs,6twf,6a00,6pml,6e2n,6h5x,6nxg,6npx,6
ttw,6d18,6gsz,6r7s,6fng,6pht,6a46,6gof,6tol,6d6y,6oy1,6phu,6q9j,6hvj,6izm,
6f6a,6oxt,6dn5,6byx,6ghj,6vco,6e2t,6v1k,6hnz,6v1b,6wbh,6mcs,6gbx,6gsx,6vfz
,6qcb,6f78,6jw8,6rc6,6hdw,6nux,6mgl,6rc4,6u1f,6v33,6r5o,6d6d,6iyb,6uw7,6ec
h,6d4q,6ma3,6xxm,6ryo,6ea9,6a0k,6d19,6fzp,6f4b,6i6h,6rgw,6rir,6ox0,6i4k,6f
zq,6j7l,6ges,6uw3,6n96,6c92,6rd1,6oxv,6a0g,6yrp,6rz6,6jj3,6qgv,6s7z,6cgc,6
av5,6kgr,6rih,6wy7,6fac,6gxk,6x5t,6m91,6lq5,6ag5,6k3l,6dgr,6cut,6wq8,6vw8,
6a5k,6pgo,6f3p,6rtw,6r0v,6hpq,6tok,6hdn,6z6a,6p9c,6dgz,6ms9,6lov,6ytm,6gtp
,6ora,6w72,6o3r,6tc1,6kaz,6d97,6f67,6r8o,6joe,6a9t,6pmd,6dw2,6hk3,6sd9,6se
d,6sio,6l86,6fyv,6rx2,6su0,6p5w,6upf,6oyd,6ak5,6djg,6hkv,6fdm,6thw,6i4s,6f
ut,6gey,6x42,6cwr,6rq3,6wy9,6urc,6kwd,6zjq,6om8,6qhb,6am9,6ko8,6lcf,6kb3,6
gqq,6mbo,6ls5,6n7y,6i62,6rnd,6mda,6stl,6no2,6g39,6hjx,6ime,6s00,6qqy,6oot,
6ub0,6bn0,6u9r,6apr,6pr2,6nhb,6cpf,6a55,6k37,6qfg,6vxv,6f3i,6sql,6n83,6vv1
,6f85,6nya,6f26,6xxk,6ma2,6sip,6wv3,6jof,6eif,6t0j,6dz2,6w4z,6r13,6npd,6f2
n,6o5g,6ccn,6boj,6spm,6cg1,6sox,6bl1,6vur,6a8d,6e8x,6e1j,6jig,6vve,6pn2,6x
uw,6hl4,6a1a,6hor,6yqk,6p9a,6icq,6ayu,6bmn,6mu3,6tmc,6dar,6pgf,6cit,6hzd,6
qbg,6ho6,6auq,6c0b,6pki,6f5z,6rj4,6ain,6n3k,6bqk,6s1y,6hle,6fgc,6hvi,6pqs,
6s9z,6mbt,6c6p,6xrc,6fe0,6d50,6qr6,6nnn,6uxj,6qyk,6ew5,6wdv,6ynb,6at3,6h3o
,6lgt,6jl2,6eea,6f6j,6cyq,6s2p,6ykk,6qxr,6g8b,6c7s,6h6o,6sbh,6rc5,6e40,6ub
a,6ho4,6pna,6ld5,6ytw,6j3o,6rh4,6ftp,6oob,6g80,6mg5,6y5c,6cbh,6gpa,6duf,6q
qv,6qva,6cur,6kgo,6c0p,6yuy,6yt2,6o0e,6iej,6qwu,6siu,6ssa,6fnq,6yjw,6bsx,6
pp0,6tvt,6kcm,6dwk,6tpm,6apu,6vd1,6i4e,6fqz,6hzg,6qch,6o4l,6moo,6kqt,6mbq,
6c1i,6s5u,6c7r,6ar4,6d6a,6f7w,6aov,6hx1,6dzn,6b2c,6mll,6usw,6dqq,6qmk,6asc
,6db2,6rk8,6qfv,6ybg,6sjr,6zgm,6h73,6lgd,6eb1,6nh8,6yaw,6z74,6fyo,6vr6,6li
0,6m0e,6n1x,6ykl,6epm,6uo4,6nwo,6sbr,6tcw,6oag,6iim,6fa4,6hsn,6ecv,6cdz,6n
z4,6nyh,6l7v,6o1e,6qz7,6j1t,6pyp,6y8a,6bzz,6pnc,6g35,6h6w,6f70,6gi2,6hh3,6
jt3,6c2t,6ilq,6pku,6sp7,6dd1,6fku,6e2w,6t4n,6llx,6ibx,6fzx,6p12,6i4m,6j3b,

6j95,6d41,6ffb,6m72,6dwi,6iiu,6pid,6i12,6epy,6eog,6a2m,6q55,6ffx,6r7j,6nrh
,6fe5,6b8b,6hf5,6e5m,6b6q,6vod,6e5w,6c7e,6x3y,6o94,6hx8,6oha,6ase,6p8q,6e1
z,6smt,6cyu,6ly7,6nwc,6fhy,6kot,6kk4,6gue,6h9y,6wbw,6xuh,6h8j,6c8c,6e5f,6o
hl,6npc,6nng,6hsp,6ng2,6af6,6jba,6xvk,6nz6,6c5h,6guh,6zl5,6bkr,6fa5,6pjk,6
bgs,6y5p,6kg2,6dw5,6upe,6ryp,6ukm,6qa4,6h52,6nh5,6gh3,6bt6,6bq3,6hl7,6l50,
6dzx,6cgf,6svu,6r8r,6u1i,6dml,6bkx,6htk,6vv7,6q0q,6aad,6vpj,6q8k,6zjx,6mrv
,6rhl,6cdl,6sj3,6chz,6ptz,6qln,6jhb,6n3y,6cic,6yty,6hfj,6obd,6p2m,6pq6,6jc
j,6ce2,6lm1,6f4p,6mwl,6nlm,6ho7,6kc4,6jmn,6oak,6vqf,6enx,6net,6nh0,6i76,6s
1u,6d9m,6bcb,6gg8,6oib,6cqd,6elo,6q0m,6arj,6ncj,6l14,6r6i,6h7y,6dvm,6bm6,6
v94,6b3f,6euw,6qx6,6l1y,6txw,6q7t,6xvq,6m89,6d9s,6w0x,6ngh,6ayr,6cvw,6o0p,
6e41,6c4p,6c7d,6qah,6qop,6hlz,6qp0,6e1y,6j67,6ttn,6gva,6t6y,6er4,6zk8,6cwi
,6g3s,6apg,6nv7,6gxv,6sd8,6fh7,6gb0,6d16,6rsl,6ok8,6g02,6pn5,6d6b,6gi1,6bq
5,6cif,6cz8,6pn6,6yz4,6vue,6zyl,6ttp,6uwx,6lip,6gj6,6enc,6v2t,6nh2,6ewa,6w
70,6f3b,6za4,6pg7,6jqr,6hgi,6fmn,6eq9,6b8o,6ftf,6gom,6r9s,6cxk,6adx,6lil,6
wbq,6zvl,6tx4,6gpn,6ksm,6hjk,6hmd,6c4t,6a1l,6y4c,6r1w,6rvn,6dak,6e6c,6sse,
6euz,6foq,6n7b,6f9r,6six,6rm8,6v2p,6arx,6ld4,6rk4,6ftb,6aul,6say,6pyl,6abk
,6im6,6apt,6pbm,6qtn,6fmj,6uux,6gl0,6mt0,6g0n,6i18,6ng6,6n5h,6j1f,6i4t,6v1
j,6yg5,6n0p,6sk1,6pg2,6c7l,6ton,6ibo,6ng0,6m2j,6xi9,6h2v,6euq,6h9r,6n6t,6y
jc,6hjj,6qqk,6mv1,6eij,6hwv,6fxy,6dd0,6lj7,6hlo,6ix8,6qr3,6zjs,6ev2,6ful,6
b16,6xp3,6fvm,6lx2,6pr9,6fx2,6c2q,6kwg,6vhz,6gjq,6jez,6bxi,6tp8,6rra,6sa8,
6pve,6fcx,6gev,6pdq,6kxl,6bbx,6wkf,6md8,6se9,6sjn,6d1d,6i2r,6e0d,6ugz,6wrt
,6wkd,6bqc,6sac,6evn,6y8o,6fh3,6hoq,6gcc,6siy,6h8p,6ops,6uel,6rq1,6tx0,6p9
z,6hyt,6h80,6qlj,6vlu,6nss,6n67,6v17,6cuz,6nch,6ngb,6pji,6gj5,6ggb,6uar,6s
39,6qae,6z69,6t3g,6clv,6pvi,6nfv,6paf,6f6d,6c75,6q8a,6ciy,6std,6gwe,6h2z,6
a16,6j5y,6gd8,6eii,6fwc,6kzx,6pg4,6b8y,6i9n,6qa1,6ow7,6syd,6kii,6jon,6dqo,
6o53,6a6s,6up9,6f2l,6kji,6fgh,6ugq,6ox4,6kxa,6vsf,6x4b,6bg4,6e46,6mdu,6a62
,6v8n,6no5,6mfw,6xvj,6bps,6gin,6r1m,6pg8,6qyi,6cyw,6ecu,6nw6,6qyn,6j1l,6ee
b,6zlf,6j2m,6fck,6o1g,6tlk,6huz,6pyv,6m69,6rfz,6z1j,6y9r,6q52,6uff,6ljw,6i
cv,6p9r,6aut,6prs,6chn,6top,6t8k,6qgj,6pge,6fs6,6oqa,6tc2,6crk,6f51,6cre,6
r1x,6p1d,6wm1,6l1f,6sbq,6jea,6tva,6ts5,6zjc,6q21,6pre,6fgl,6ccl,6e0n,6rly,
6tym,6q9l,6pii,6ppy,6gh9,6ngw,6d2o,6cbn,6opt,6rhg,6r5b,6d2n,6bvi,6usc,6wuf
,6x94,6ar9,6rit,6t8l,6cxe,6d5e,6wgw,6jzu,6jag,6kck,6pp4,6ato,6ici,6f97,6nj
j,6gmn,6n0z,6jdw,6cis,6rg3,6qot,6oxz,6qyg,6enh,6rma,6b7e,6m7h,6hgf,6pka,6h
lx,6vcr,6vph,6ia7,6o0l,6fl3,6kk3,6xb4,6cz3,6be4,6ntu,6i6c,6ghr,6t03,6fqo,6
bh4,6bmq,6gvx,6aam,6jn1,6ekx,6oms,6r2a,6kxy,6ps7,6bl2,6t2x,6ma5,6ysa,6j3f,
6y4g,6hp9,6y7y,6lp0,6dbk,6hfw,6mso,6pnb,6o1s,6mjw,6nsg,6d0e,6pqw,6ap9,6hty
,6yw3,6yuw,6ofv,6rum,6k9u,6qxd,6yaq,6js8,6g3f,6qha,6vux,6wrm,6acr,6x9d,6ub
3,6er3,6gqo,6nec,6f7r,6p88,6edv,6sk9,6r67,6cea,6ru6,6dl9,6tcf,6zqz,6m65,6k
gq,6dw4,6h3k,6j72,6oo9,6rto,6c0s,6a5q,6unx,6y7w,6r4a,6oim,6h35,6gog,6yde,6
n77,6ttz,6prr,6ew4,6pjx,6a8i,6ktl,6i22,6cyh,6jtc,6egn,6a7c,6lq4,6z4b,6noq,
6ssf,6kv9,6ujh,6b5g,6p63,6oqd,6ogp,6ha9,6mv4,6f3l,6s7w,6g0b,6pb9,6eks,6taw
,6z44,6nrf,6h76,6kmr,6tyo,6y0o,6n35,6esn,6y0n,6i17,6l1p,6nv1,6p5q,6izn,6y8
l,6rqp,6ab8,6t09,6gpo,6lxy,6k9r,6q4e,6qgr,6ufb,6ajv,6ady,6thz,6b1m,6fyr,6n
g5,6jxs,6pc3,6pfj,6kbf,6kia,6qgt,6pws,6cxt,6g9n,6g0w,6enq,6cen,6kun,6yps,6
tu1,6mtj,6q39,6c7i,6ze8,6uat,6l13,6o51,6h2u,6onr,6qzz,6op3,6uik,6fk6,6rgu,
6vsu,6ox1,6dq4,6mne,6asm,6iae,6xw7,6rc3,6i0k,6i15,6rh8,6g47,6y81,6gnb,6cks
,6jjn,6pd7,6hsz,6qcl,6j2e,6t8r,6pg6,6ug7,6kk2,6qlh,6f4q,6jzy,6bsv,6jse,6g1
i,6c0r,6s26,6dew,6st3,6szx,6md2,6pmf,6rsw,6r1s,6e90,6uxi,6s51,6ziz,6aiy,6g
wf,6r9w,6ybl,6i93,6owi,6rvq,6wp5,6vw4,6hm7,6kx8,6era,6r4j,6m8q,6bfe,6fgu,6
uku,6fns,6tbx,6lxa,6i67,6uo7,6gcx,6pdd,6ra1,6h1e,6hgg,6ljs,6nwd,6efk,6ryn,
6ie9,6egu,6gqp,6nem,6ngm,6ayb,6ayo,6po9,6rwj,6hg2,6pyf,6tod,6j2a,6nx1,6b3b
,6mhf,6mo7,6psb,6dnp,6tt1,6pra,6qde,6neu,6gqm,6zho,6suk,6a0l,6lxb,6co5,6fp
4,6py9,6cwl,6mb1,6g4f,6b0z,6vke,6hgj,6lhj,6evr,6wfl,6yl4,6c3c,6qr9,6oqb,6h
x5,6dh6,6aik,6bmk,6hno,6kc2,6ycu,6q89,6qns,6x0h,6y5y,6gia,6f8c,6mqr,6i94,6

g37,6qd9,6c3l,6c5b,6b04,6mvk,6poc,6pz4,6qcj,6sau,6myv,6e22,6ovz,6m4m,6c6o,
6qe5,6atr,6b1b,6q48,6rvl,6j9w,6yw2,6md0,6edk,6b6o,6nfu,6hxk,6jr7,6hca,6fpg
,6rtm,6qls,6x5h,6pxu,6axu,6f9g,6h21,6qoc,6hdt,6ynt,6grn,6d4w,6dl2,6cqu,6eq
w,6f6s,6e92,6kb9,6csq,6rul,6gqk,6ela,6bvl,6k0p,6bnj,6hqk,6lds,6f2b,6udt,6t
tt,6nx6,6b21,6x5p,6lt6,6twi,6upj,6t0a,6ko9,6aur,6uka,6qhs,6ep2,6moz,6t5v,6
kp2,6pw3,6mbn,6n4l,6i7l,6pbt,6eum,6gsg,6tmn,6eoa,6o0w,6jbb,6buq,6fmq,6rsj,
6ld3,6eli,6ei4,6hmb,6gp8,6vre,6f3d,6p0i,6hs0,6qa6,6cee,6us4,6nri,6qau,6eeh
,6tj8,6qw7,6b81,6jkz,6rcf,6bgq,5fkk,5axu,5t3h,5kcf,5mpt,5ox2,5r25,5bx4,5ur
9,5v4x,5acf,5i80,5ymr,5cqt,5xs3,5al2,5ja7,5org,5vun,5nvh,5o3q,5hu0,5mlz,5f
8y,5ad5,5zfi,5ty1,5a66,5fho,5zgq,5wpj,5o4l,5wb3,5mff,5liw,5br7,5xb2,5z2u,5
xcs,5n34,5x20,5o98,5ahn,5iqf,5n5u,5a88,5ca3,5ftw,5wmo,5aku,5m1a,5ayf,5i2z,
5fnr,5eoe,5ljt,5icd,5etl,5fnt,5ff4,5xu9,5c0d,5zms,5fdz,5bpf,5wr6,5kds,5fem
,5m6e,5z4h,5ioy,5cxh,5mty,5xoy,5d3p,5ukv,5o7w,5fh0,5adc,5msr,5u97,5fr0,5a6
k,5thu,5dx7,5h8g,5krc,5cs2,5lbx,5unz,5vtv,5gsr,5er2,5mpk,5wlo,5omj,5knk,5t
ns,5yqt,5v13,5g65,5dnc,5efa,5du8,5etj,5bot,5hv0,5lvp,5jq5,5hhq,5a99,5etd,5
aeh,5tn4,5o08,5c09,5lvn,5sxr,5gws,5jlb,5ote,5vlq,5kce,5kgt,5efc,5g6a,5z46,
5wrj,5tdm,5aac,5fns,5o1f,5bul,5wzt,5ctu,5m9x,5bns,5epr,5qcr,5cas,5pgx,5m4e
,5uih,5i3a,5x23,5g5w,5dqe,5glz,5lb6,5jfv,5b6e,5yu1,5d48,5tet,5l7u,5qah,5di
d,5bxv,5f2s,5v7a,5eja,5v02,5kbf,5y5q,5efq,5ouh,5b41,5fk8,5fm0,5el9,5std,5d
ep,5aki,5swn,5mqe,5foq,5a5o,5iw0,5t03,5c8r,5dxv,5ie0,5dcy,5wex,5ews,5lby,5
vnl,5y6q,5oim,5mzp,5vif,5zgr,5iu8,5r9n,5fg3,5oi2,5mm0,5yf1,5dda,5i8b,5krk,
5lzj,5ra5,5enk,5c3b,5hi7,5vcz,5cgt,5lau,5iiy,5qad,5a5w,5vd1,5g6o,5jy4,5owa
,5wp5,5mbd,5k4y,5c9p,5m5g,5glm,5ad3,5wuu,5x0j,5eek,5t37,5jda,5g6q,5qab,5u6
i,5vum,5h24,5drq,5dt2,5m58,5tuw,5g3n,5kct,5wef,5wa7,5y5t,5ddk,5qu0,5aba,5e
4n,5uo9,5jep,5adt,5fvy,5cc9,5n75,5xj7,5me4,5nou,5xpl,5n5r,5aan,5b4a,5uqn,5
np9,5lb3,5cu0,5vuj,5fot,5dwg,5mpz,5op8,5qa4,5n9c,5dnl,5j7w,5uf0,5e9b,5llt,
5m2g,5r95,5v83,5h81,5gm5,5ehg,5vmf,5iv0,5hyv,5o1c,5xss,5tys,5dky,5lzg,5e76
,5j6d,5r9l,5djt,5ku9,5r8f,5kbz,5zqq,5qat,5kbr,5jh2,5uql,5i9w,5fuv,5xsr,5lo
0,5zed,5je7,5sym,5kk4,5v5l,5vce,5nn4,5drp,5jxq,5lrg,5d62,5nhz,5tb5,5x2z,5z
hk,5lxs,5ygx,5i7x,5zhe,5ult,5ta6,5k0i,5ick,5obk,5akz,5v18,5nr8,5cmk,5dhj,5
amc,5ubr,5u5k,5f6y,5joc,5lny,5u00,5z54,5m7p,5bwd,5hcv,5tz6,5u3s,5fpp,5dhr,
5i4o,5ett,5swb,5icm,5fff,5svw,5il2,5yft,5mip,5cg1,5c0c,5wk7,5d85,5d2r,5w71
,5mmn,5aqu,5fpr,5ou1,5ehm,5bke,5cic,5tdb,5wyf,5u3y,5a6i,5c1h,5dfd,5u3f,5ut
1,5dnj,5oec,5fqs,5orl,5ueu,5c9w,5u41,5clo,5lfo,5del,5uqw,5ief,5cjh,5amh,5h
cn,5j8z,5cfv,5ifk,5xl4,5a4k,5cfl,5zk7,5mu9,5msv,5cxa,5k6o,5nhg,5olk,5r2c,5
nm1,5k06,5qaq,5o6x,5xd3,5a67,5kri,5wo4,5yne,5vv2,5olv,5mot,5r8l,5j75,5fqg,
5y0x,5mam,5c0k,5vvg,5b0r,5mlr,5aen,5t19,5cb3,5v4r,5ra1,5nwe,5iqe,5xih,5iev
,5y8j,5mbk,5i8y,5x9p,5e6z,5fnq,5yqm,5u0y,5b1s,5adm,5y2t,5ers,5ts5,5mi5,5td
z,5uau,5w7x,5kf2,5mgk,5c84,5ma1,5kp4,5vib,5dug,5w8c,5xfh,5mxo,5dr2,5zm4,5a
81,5fu3,5z5f,5k6n,5klp,5ark,5zro,5x2m,5okl,5ut4,5hdu,5of0,5sz4,5ffr,5b0x,5
vv3,5ncb,5cfq,5fe2,5kp1,5bw4,5dg3,5vc4,5nim,5flo,5h5o,5eu4,5bqi,5aqj,5r0i,
5l1w,5u4t,5b3a,5n12,5xnc,5jcx,5lyn,5o12,5vty,5kdo,5lom,5wjd,5j63,5wht,5chr
,5gtp,5b25,5cs8,5dmm,5a65,5tq4,5bwo,5liy,5vsd,5d42,5l95,5mtu,5n87,5oa8,5d6
j,5u5x,5r94,5etx,5tuk,5lef,5y66,5j5o,5m42,5hka,5c7c,5d0h,5u0n,5g3z,5htz,5n
pr,5qtt,5i9y,5ivt,5wgs,5ada,5ull,5p9i,5etv,5vmc,5nns,5fvw,5nhh,5mj5,5g0n,5
u7d,5eh0,5adg,5kcy,5myl,5j8m,5mx6,5h2d,5tp9,5gvh,5his,5ew0,5sz0,5qil,5hy1,
5cpe,5ftg,5aqv,5n6y,5c7n,5e5g,5hc7,5ekx,5jap,5i5x,5gtn,5l7v,5zlc,5ykt,5nuf
,5z1n,5f9t,5n65,5a8p,5fvj,5jy6,5n5j,5u61,5icv,5o7u,5qas,5b0q,5vxc,5bq0,5ii
5,5o1h,5ugx,5il1,5nag,5z9s,5vfi,5tiu,5iey,5veq,5svi,5aaa,5d7d,5cft,5c4f,5m
uy,5jox,5av2,5u6v,5ke0,5ta2,5qay,5bwr,5b2z,5u1m,5hjk,5gm9,5bnr,5x30,5r1v,5
nn5,5r8m,5m5s,5kf8,5v79,5yia,5u6n,5vut,5kr2,5nwb,5mso,5fp0,5cct,5m1r,5sxm,
5lat,5ac0,5ald,5n6s,5z4o,5kd6,5ug2,5ef7,5l8c,5bvb,5vyu,5jwp,5cj6,5nxo,5ctx
,5xs2,5ihe,5lu7,5ias,5jzd,5arg,5zzo,5ec4,5jv1,5hxm,5y12,5mug,5g2q,5apj,5tw
c,5v7y,5wje,5nyz,5thj,5w22,5voh,5ra6,5unu,5tme,5ctb,5i3y,5alj,5dxm,5wjj,5a

lf,5tn9,5o1g,5h5e,5fp9,5i7s,5lpz,5id7,5d2k,5nh6,5ywx,5m16,5z2m,5nmk,5cks,5
qdc,5nk3,5trf,5eu1,5am4,5ty9,5osw,5mzk,5m5f,5tb6,5qqp,5o3f,5dm1,5zw7,5oo4,
5o4k,5nh9,5f31,5g0p,5iwq,5a8z,5op9,5wal,5ie1,5g6f,5gmd,5u14,5zzs,5owl,5djh
,5dif,5tpv,5x8i,5eh5,5edg,5ya5,5vm0,5zcu,5qin,5gls,5kms,5uv1,5hq0,5fkc,5kd
q,5o4t,5wgu,5lxp,5tyh,5l01,5fvv,5amd,5a03,5kzi,5glo,5m0s,5aqn,5yvk,5aqr,5c
i9,5ky8,5lgz,5cu2,5fc6,5twb,5iui,5vhb,5swi,5f2e,5vjg,5mqm,5crz,5r9v,5ivy,5
ja0,5jq9,5otc,5kxb,5t9u,5oep,5jaq,5mkj,5fux,5vw3,5d41,5bxq,5hbh,5tvj,5aqo,
5dsx,5c4s,5uod,5i46,5fi4,5u06,5j47,5not,5nu7,5thq,5l30,5lqs,5u5n,5ork,5dfp
,5nxv,5ac4,5ojn,5m6o,5adj,5x86,5fc4,5b2e,5a1j,5fb7,5nr7,5e8a,5uox,5ej2,5om
g,5arf,5t6b,5akt,5mxf,5gwr,5kod,5fpe,5f2n,5syz,5a8y,5n64,5txr,5lch,5log,5l
xt,5aa4,5enq,5wnl,5ynw,5dxq,5jdt,5ake,5nha,5tvf,5f5n,5jci,5ew8,5ix0,5h04,5
t9e,5n63,5yb7,5hne,5n9n,5myn,5tix,5xl6,5c1l,5mwg,5e40,5oku,5tp0,5g3r,5mov,
5x1j,5t9c,5dey,5ixs,5emj,5csp,5r9a,5ao8,5tya,5ocq,5d1j,5w5t,5u0l,5a2z,5n9r
,5mm9,5dkr,5gp0,5l9k,5wgl,5ohi,5mw4,5ols,5umw,5vui,5ime,5eq1,5deg,5wfr,5dc
o,5fnj,5c8l,5thy,5nio,5ub5,5nvf,5cp5,5a2w,5thn,5lgb,5wmq,5nxx,5r09,5nj4,5k
jk,5x73,5dap,5a3w,5ocs,5ti7,5xld,5j5z,5gjo,5veo,5dw1,5qcq,5u19,5fxf,5d9n,5
n9z,5d5d,5dyd,5odi,5zhl,5kxd,5em1,5noy,5c0b,5otr,5ihh,5jix,5wi0,5t4u,5w7p,
5dus,5ojy,5jab,5iqb,5ixe,5jrs,5uio,5y8i,5ans,5g4r,5lji,5gid,5y8p,5a28,5bvo
,5ko1,5gly,5h9r,5z48,5dyo,5j4t,5ow8,5aqy,5bju,5irc,5fli,5l1p,5cxx,5i5t,5hw
r,5m18,5tiv,5r8h,5kmk,5hbe,5q0e,5wm7,5a02,5fqf,5wdp,5m8t,5ysz,5jhb,5zbs,5h
ws,5tze,5cy9,5eh8,5afl,5i7a,5dbv,5nar,5c7o,5cx6,5w97,5npc,5ox4,5om6,5juv,5
ti4,5hjo,5g5f,5dji,5zk9,5a8o,5ml5,5hyl,5xrv,5g0v,5ra9,5g6g,5a2l,5a6l,5epo,
5cvf,5xin,5d63,5k00,5eva,5u5w,5ack,5e9c,5ey4,5mnb,5ceq,5nv8,5kdu,5xcz,5d7c
,5o0z,5x5u,5yw0,5fp4,5wfw,5gvm,5hwk,5hn7,5ag6,5yk1,5j5g,5joy,5i6d,5mlj,5dh
p,5byb,5fbe,5j8o,5dv2,5sy3,5wdq,5vuy,5c0e,5ega,5f27,5vee,5grg,5ktq,5d6p,5v
2j,5x2a,5h4s,5i83,5auo,5tes,5vrm,5zce,5g68,5ypm,5qcm,5hci,5j6y,5fvr,5i9x,5
bir,5f79,5owc,5if4,5mro,5u88,5ece,5iab,5cak,5ysy,5wzv,5ujx,5jil,5ey0,5neb,
5icw,5l7e,5b72,5wm3,5fvs,5u5u,5ji8,5nnb,5kcz,5c4b,5m28,5w7u,5cdh,5eel,5o0b
,5wuw,5ac2,5h5i,5ma0,5or9,5bpa,5njf,5z6t,5nw1,5e4r,5uin,5u43,5yx4,5u2m,5ms
s,5mrk,5jnw,5z9l,5oo8,5tmr,5jhn,5wgx,5wfo,5h5a,5bjv,5b6d,5vb7,5obu,5ew9,5x
w8,5ggj,5gzk,5d6t,5g5v,5nk2,5ln8,5qcu,5jf4,5l87,5am1,5lbe,5zg1,5jvd,5ky2,5
l7h,5eyr,5x8q,5exm,5r8a,5oni,5f05,5cjm,5qaz,5vyt,5ui2,5e8p,5jlg,5bo6,5f7x,
5ao7,5tk9,5lwv,5kab,5oa6,5ni0,5v9h,5mrm,5mh4,5tg5,5bvw,5y9l,5vcw,5tsu,5ovi
,5vja,5u23,5o3e,5ftc,5wks,5yly,5a8k,5ih0,5ih9,5x67,5jdv,5ngt,5f7r,5nbp,5er
r,5z49,5x17,5svo,5lvq,5nox,5bv6,5kox,5ag3,5vsc,5ij3,5m0d,5jzj,5tva,5ni6,5a
l3,5w2h,5m4m,5fbb,5abh,5ak6,5dhq,5mgj,5udp,5ccy,5mpq,5ewh,5fvx,5mks,5y5p,5
fnc,5mag,5coo,5ful,5qby,5mai,5wkr,5fhi,5ynt,5afj,5cq1,5kok,5h7q,5z53,5ff9,
5i40,5ei4,5c4i,5kga,5l41,5uo5,5o7e,5yql,5os1,5b5g,5wfp,5mxr,5vef,5ovc,5txs
,5drs,5iqd,5f6w,5ko7,5tzz,5kr1,5gop,5abf,5c3s,5uex,5doz,5k4v,5cei,5tv0,5fj
3,5nob,5y8t,5eou,5f0f,5czk,5okt,5jmp,5fue,5boj,5tpg,5e28,5i8z,5mob,5ciq,5r
9f,5ksp,5ew3,5o9v,5j7x,5nnf,5krs,5ipe,5j1w,5vz0,5oqw,5vuu,5hyr,5ia1,5ggp,5
x6r,5dux,5wgk,5ae3,5ti2,5khk,5d25,5jwv,5dav,5njh,5nft,5npe,5njz,5g1z,5k9b,
5zdn,5g6e,5nki,5na9,5cad,5k52,5bsz,5okr,5l0u,5lwr,5jqt,5v40,5jss,5fe6,5cbj
,5i0l,5c7r,5tar,5mek,5lcx,5r1u,5lcf,5qtx,5jiq,5y6e,5ezu,5vlc,5yk0,5vv1,5d7
p,5n1q,5edt,5l0t,5dgr,5x8s,5ajw,5iqt,5wxu,5a4v,5eqr,5acc,5yzh,5bph,5ypn,5x
74,5k2m,5d75,5ibi,5j9x,5h40,5kqr,5a98,5v9f,5zq4,5coq,5i39,5vsm,5g3w,5tuf,5
u0d,5r87,5mow,5f00,5etb,5fnh,5niw,5wxg,5js6,5hqx,5oir,5dne,5t54,5k5n,5tw5,
5m7u,5c7v,5nwc,5nzn,5g0g,5y84,5eis,5fsy,5hca,5hr5,5laq,5ab7,5erv,5eoy,5uln
,5opr,5n81,5ind,5wgw,5edc,5qdb,5e50,5dex,5we9,5o4v,5nf9,5r9i,5n6c,5mfq,5hs
a,5gg7,5nhf,5yg2,5ei6,5ltz,5ldq,5t2i,5grm,5ok4,5chc,5ida,5o1d,5z4t,5xyy,5v
w7,5wfq,5j5i,5ayj,5hk9,5o9q,5uc1,5gpb,5ufm,5lxb,5olr,5n70,5b5j,5h3q,5cfw,5
oc3,5dy5,5el2,5nfa,5akk,5kcb,5nu2,5k3b,5xk8,5v49,5opj,5fw0,5fch,5nbw,5ahf,
5ox1,5xsa,5v8e,5c8q,5qar,5aol,5qbv,5lmc,5fbo,5ipj,5cq0,5fjz,5u21,5duv,5xxn
,5jgt,5ein,5yji,5y0f,5w3i,5uoa,5ijq,5hje,5kiq,5k8p,5kew,5vur,5glp,5hrp,5hw

4,5nee,5ifu,5o3b,5ag5,5zps,5gsa,5o7d,5adr,5uwa,5dcs,5xww,5vd2,5ula,5flq,5m
x8,5h7h,5tyi,5ahw,5kkc,5l4l,5mhp,5iax,5g17,5nna,5nlm,5o6o,5ikh,5uwr,5oew,5
r2b,5m6f,5uxf,5bjw,5an1,5mji,5g6l,5oot,5ybo,5yjs,5oke,5hgb,5xst,5var,5val,
5t4b,5y5u,5vv9,5ot6,5f1l,5tz1,5amv,5j4k,5fvu,5hwq,5uf1,5g6p,5g6d,5e2k,5fwd
,5n0d,5g3l,5o3r,5fe5,5wdn,5lgx,5dog,5qqo,5bui,5o0a,5c9j,5m7i,5tzl,5l3a,5y0
q,5v2t,5vkt,5r9p,5akh,5mvd,5yms,5c85,5alh,5vrj,5glr,5aa9,5ivs,5daq,5mz2,5q
tw,5ouk,5zho,5ohj,5n9k,5ivr,5ccl,5yf0,5idn,5hjm,5a8x,5o0f,5ov7,5y9m,5ei1,5
lvr,5wgd,5epa,5l3r,5y02,5l6e,5auw,5hzb,5usz,5wpk,5yz2,5ox3,5xmp,5toe,5ksy,
5ayt,5f1j,5dh4,5tpc,5vsl,5z5t,5a7y,5ka1,5nxe,5tq6,5x5d,5etr,5r0l,5uav,5vqn
,5f5c,5k53,5ad2,5ne2,5ky0,5hgu,5h19,5xwl,5lol,5nzf,5v82,5wke,5jy3,5zae,5r9
m,5eei,5zf7,5j6m,5o9y,5tcj,5ucd,5fnl,5x9d,5axi,5nub,5iqz,5b4v,5ybs,5hio,5n
rb,5nky,5iv3,5aix,5l4i,5g6u,5tfz,5m8d,5o55,5g3u,5a5d,5erm,5bxa,5dbf,5a4t,5
mu6,5xpo,5oaw,5jg1,5vus,5b0w,5jbe,5y7a,5l0b,5t46,5k8v,5r88,5i2c,5v0n,5y94,
5f76,5ito,5r24,5xwj,5h9o,5tln,5a2i,5op7,5uij,5maa,5e33,5i85,5e4f,5ais,5csd
,5oue,5ky9,5r28,5daw,5ect,5mri,5zhj,5vwk,5es3,5mb3,5gst,5auy,5amg,5pgv,5b4
t,5njv,5z75,5mfr,5i9v,5dry,5n58,5qim,5jm8,5dly,5mmf,5hgz,5whd,5grt,5ied,5u
oo,5iv4,5x40,5ovk,5equ,5lbz,5o37,5qaf,5xg5,5r9q,5zlt,5kcd,5in3,5i2n,5os5,5
buu,5dfm,5evk,5efh,5a7w,5eu5,5vq8,5dqc,5am9,5f3g,5ljd,5feu,5yh8,5kcp,5gvl,
5r8w,5itp,5wf7,5e74,5wou,5zbd,5aqf,5d3l,5r9o,5z9p,5al0,5zu3,5kdt,5cps,5yoc
,5m6m,5cmm,5tex,5cts,5nqr,5f8w,5ep4,5v8u,5tq5,5g4o,5jal,5i0m,5lik,5dlw,5j5
r,5lun,5fpm,5cno,5a14,5pgy,5t6z,5xk9,5lia,5ha7,5r0d,5hrr,5v80,5wx3,5npd,5k
8c,5fa8,5yrm,5uik,5ygy,5jf1,5y8l,5cp8,5o42,5kqf,5xj6,5dpx,5qty,5dzi,5wpl,5
osq,5tri,5vpi,5ra3,5mxw,5dn3,5fpb,5ghq,5fp6,5f08,5a5r,5cco,5ice,5k4x,5o2e,
5enl,5gsx,5byi,5iqc,5g4l,5dw5,5q0d,5fp5,5vi9,5g02,5uwk,5y9d,5lga,5fkb,5jam
,5g3s,5ld5,5er0,5aoa,5o2d,5bo7,5ni5,5mte,5miq,5oyh,5g57,5edi,5tjh,5mg2,5m8
r,5ogh,5mxq,5z6f,5e73,5l8j,5a7c,5xb3,5kj0,5eko,5dia,5mfe,5vv8,5n0r,5bnl,5n
kf,5xs6,5mno,5lae,5mgc,5u3r,5x4o,5uoc,5a2j,5nxi,5o8e,5neg,5uo2,5zye,5n0t,5
czy,5k7g,5g0o,5i0j,5ivv,5mnr,5u11,5aks,5hgr,5o6j,5yid,5fk7,5cy3,5h59,5l9z,
5ud4,5ade,5l1v,5okk,5tzr,5ntq,5ulp,5d9w,5wx6,5z44,5m7y,5nfb,5fh3,5v8q,5hd0
,5yna,5w0l,5ct0,5vah,5dhf,5h8n,5dfc,5uab,5awq,5t67,5sz5,5zob,5l44,5a5q,5js
j,5mzi,5x7q,5cx9,5uqk,5n0u,5ibf,5tr6,5mwe,5ur1,5irn,5ot8,5oq5,5dsc,5ck4,5h
hm,5qb2,5gmh,5ibp,5mod,5zeh,5l8n,5wgq,5qcx,5uo6,5i95,5g45,5imi,5ur5,5ysn,5
ou0,5qb3,5yrv,5ie3,5tq8,5etk,5uwq,5vn1,5xvs,5lvd,5cel,5f60,5xwv,5eyp,5zkc,
5om4,5tnb,5u60,5mfi,5kjh,5exk,5x2n,5dbm,5hu1,5gs9,5wpz,5xvh,5dh3,5x66,5ju6
,5a7r,5bxj,5meh,5w4w,5unx,5xqk,5o39,5krh,5gmm,5g6n,5k2b,5c27,5a2r,5t7g,5o1
b,5cbr,5ets,5xz9,5jxh,5unv,5w8h,5i51,5g1c,5dp2,5jo1,5o4m,5akv,5ur0,5imx,5h
13,5acl,5qak,5w7m,5jic,5i3i,5h7k,5nsd,5tm7,5az1,5dw6,5ngs,5emy,5goe,5zm3,5
o3d,5f0h,5vcv,5hor,5im3,5ad7,5n6t,5vup,5l4f,5ira,5dvs,5ul4,5e9i,5doh,5bxp,
5tmn,5f91,5ldm,5con,5htd,5c83,5bsv,5mwj,5hmw,5dbi,5dy9,5yv5,5qap,5aqg,5h3a
,5orw,5erg,5gsd,5eef,5nov,5fs6,5zz7,5jdz,5w0q,5ah7,5cxz,5lca,5nwg,5nos,5la
0,5xlz,5o3g,5tl9,5eib,5r0k,5yjk,5ho6,5ye9,5gm4,5fl6,5yu0,5cj2,5fjj,5c03,5h
n6,5a9u,5d4y,5wgr,5ooa,5jg3,5twl,5k4q,5wcz,5j3l,5f3t,5eqf,5ij1,5ly2,5nfp,5
iki,5vnb,5utq,5h5k,5yfu,5wdc,5vij,5fay,5ocm,5dlv,5b5b,5ehp,5e7j,5mdh,5j48,
5jjm,5vv6,5kx5,5icc,5xo2,5fbn,5opm,5k72,5jnl,5nap,5noq,5uga,5mbc,5xxs,5exn
,5n6b,5uo8,5fj2,5bkd,5zfb,5w76,5muj,5nk6,5ami,5gvp,5ia4,5gsp,5cpz,5d3r,5gt
9,5tdu,5xsm,5t8r,5g27,5t2p,5etp,5ltj,5yak,5aju,5k9c,5wlv,5iu7,5ucj,5vu4,5a
7s,5kju,5g61,5xtx,5f7v,5jsf,5bz4,5ale,5adf,5i2r,5vv4,5fe9,5aov,5y5e,5jax,5
ibd,5i43,5yhe,5tn6,5vro,5l2s,5wdw,5die,5pgu,5i5w,5m51,5y4k,5idm,5vw4,5hh1,
5mim,5u6d,5mb0,5cfs,5e8n,5aqx,5ceo,5vpx,5kcq,5t0e,5y24,5gz3,5vs6,5eob,5brh
,5ynv,5r8o,5c8n,5obw,5v3d,5jws,5gto,5nvw,5lje,5vb5,5z2x,5t96,5ztn,5lkg,5w9
9,5kka,5k0f,5wm4,5bu2,5yoe,5hrw,5xxm,5g4n,5trk,5n2d,5zwb,5t7s,5mpn,5usj,5k
qs,5jv2,5whb,5l7w,5ws3,5unr,5fh5,5n2i,5u44,5zg2,5utp,5w73,5r8e,5kcu,5ehw,5
yzc,5n1y,5gm1,5ypl,5w7e,5wja,5u5i,5vv5,5byl,5nlo,5mpj,5kgs,5t18,5uip,5je2,
5jse,5drm,5kqg,5klu,5aap,5z9b,5yee,5vwi,5f7n,5kej,5vv7,5ihr,5e9e,5n69,5d1r

,5w92,5k5z,5fcb,5bpy,5u24,5e0i,5fpn,5xjd,5f2t,5tcg,5hd4,5mmr,5agy,5exy,5g4
6,5r9x,5i79,5hzc,5tlv,5l53,5afz,5eyd,5wj6,5jsp,5ck5,5h15,5cbl,5iwg,5agd,5c
26,5iwu,5v2k,5hi4,5u0z,5l9i,5i04,5y13,5c2n,5uas,5fiu,5r85,5e19,5f3z,5csv,5
u3v,5nlp,5xqx,5hae,5tnt,5e70,5j6a,5k9d,5llb,5w3q,5lp1,5r92,5e72,5ml2,5irq,
5mtp,5gxu,5iw8,5xii,5i5s,5ftq,5fjm,5ido,5mvr,5ur6,5v9l,5edd,5igs,5m5a,5ti3
,5bva,5cjf,5yss,5e23,5uwf,5r9e,5tjx,5d7x,5nhv,5v2l,5d26,5eq3,5w2s,5j94,5g6
k,5ml0,5dl0,5jzb,5oty,5wev,5fkn,5wxh,5t7h,5dzh,5o3i,5c4c,5mn4,5z6j,5zf4,5j
9f,5khm,5vd3,5uil,5thi,5di9,5e1b,5adk,5jnn,5uob,5u0t,5h0e,5bue,5ak5,5r8q,5
akl,5r9z,5bvf,5lfa,5hmx,5f5r,5ahg,5dj5,5r8x,5m5k,5cbb,5o0j,5th1,5o5a,5unj,
5bmm,5j2u,5yxc,5f6m,5y0v,5fu2,5dx8,5ely,5x4m,5f06,5za0,5lls,5mjn,5ff2,5zic
,5zrg,5zxd,5uwm,5uwh,5xlc,5ucp,5g0t,5ahm,5xz7,5nlh,5sz2,5ddw,5bmp,5fa5,5e8
f,5w7c,5thv,5j41,5d4u,5usy,5a8r,5u0f,5odc,5kq8,5r27,5nba,5djg,5wm6,5ezg,5t
zh,5x13,5fvq,5ain,5oqu,5b46,5n21,5ovl,5jf5,5ox6,5y2b,5dcr,5mrp,5f2r,5jai,5
j20,5ebv,5mp8,5a7b,5xms,5yrt,5omh,5m62,5kva,5d3c,5ea4,5khj,5hrx,5cve,5iuw,
5r8p,5uao,5ir5,5du4,5yic,5w0n,5u6s,5nre,5r0f,5alw,5vo1,5k0s,5eac,5u8z,5msp
,5o6m,5zni,5bw6,5brv,5xik,5fnm,5jrq,5sxt,5o0c,5sz3,5dtv,5oc1,5jcn,5j2x,5wq
6,5e3r,5a05,5nze,5dkz,5g0w,5ni2,5jck,5nih,5nun,5xfv,5lb9,5vka,5zdk,5nah,5a
wp,5x1n,5un9,5r91,5dmf,5ijs,5a57,5vsf,5oci,5kpc,5ntw,5ol4,5meq,5toa,5aob,5
nkc,5u99,5l8o,5uiv,5ufg,5auv,5ovp,5hld,5kjw,5a56,5ggk,5oum,5m6n,5h4r,5w8b,
5ovx,5ezh,5jwm,5owy,5e2p,5ul5,5t3n,5br1,5orb,5tgk,5x7e,5xqa,5lrc,5om3,5zqr
,5jo0,5ez1,5t7i,5e0w,5ftf,5u46,5dgz,5uf4,5nyh,5j89,5ka9,5cuo,5hzz,5oeq,5eh
y,5oip,5av0,5oe4,5tpu,5g2b,5mt0,5vw9,5q0h,5tds,5r8n,5i8c,5tux,5b3x,5mmo,5w
pm,5ze9,5wei,5jfp,5ypk,5uuy,5f2f,5gmv,5eyg,5vcy,5ahs,5z21,5gp1,5o8q,5hvs,5
vwb,5dkg,5dt0,5f24,5ldk,5eno,5z2f,5ovf,5w3y,5eeg,5x6p,5tn1,5tci,5tco,5t2y,
5msw,5a1g,5bve,5hes,5aly,5agi,5n4t,5aom,5ae2,5hs2,5nu6,5h2u,5gwe,5jwi,5oe6
,5uap,5alu,5bno,5w9c,5v71,5ddc,5fbp,5ycs,5a4q,5c87,5et3,5aav,5m8u,5nuk,5xz
a,5ml3,5uk9,5myg,5za7,5bx5,5tvr,5m70,5mka,5b2d,5ddb,5w9g,5a83,5l91,5uun,5o
4j,5amj,5i3k,5h67,5div,5dy4,5ybp,5g3y,5l9g,5xqw,5bvd,5def,5kcc,5ajb,5tmp,5
j51,5zgp,5acy,5zaf,5yas,5w44,5ut6,5j7g,5btx,5g6i,5nmj,5y8z,5tz3,5ut3,5zca,
5kmm,5kww,5uo7,5ig6,5upz,5l1u,5bqh,5tt7,5hwv,5tht,5vqw,5lpk,5c3r,5ukd,5ent
,5edb,5x03,5z5h,5fa6,5jog,5m90,5fqc,5h8s,5adv,5dub,5fol,5bwv,5g1a,5ra7,5bx
r,5m8g,5ii2,5myr,5k04,5os0,5cvh,5jcm,5n9s,5ff1,5yjm,5wre,5o5y,5hza,5hmk,5c
8p,5gr7,5vw5,5b5f,5lyw,5lvo,5hc8,5ehj,5syi,5caq,5hz6,5fuw,5whe,5tue,5ciw,5
jf7,5dm2,5mnn,5y0n,5dk4,5fsn,5krl,5b5p,5c3k,5ep2,5wbm,5u3b,5vtw,5xwa,5yyf,
5a8w,5fwg,5m5d,5jfs,5mt9,5wg9,5mxx,5oxk,5di7,5lm6,5ep3,5zmy,5cx1,5dv4,5l77
,5ktn,5fhq,5zbc,5ysd,5wgy,5gqm,5t64,5h7n,5afy,5afv,5awd,5fcz,5t4v,5tpx,5vv
d,5nj0,5kgj,5m6h,5ff3,5kql,5anu,5x0g,5yll,5bry,5hpo,5oco,5gie,5qcp,5g60,5i
a0,5ly3,5c2h,5itz,5uj4,5lpa,5lbo,5xio,5eud,5ybf,5in4,5jao,5kcj,5mky,5tch,5
lj2,5n0q,5e2o,5mcb,5w7q,5jmy,5ny6,5ut0,5c0a,5r2a,5msd,5bvs,5wr5,5aqs,5nrf,
5lpl,5i7y,5eml,5lzi,5ofi,5r8v,5yrj,5acx,5xxi,5d3j,5klf,5dl4,5li1,5n18,5vwa
,5m06,5jwc,5t9f,5gub,5c47,5wc4,5h8t,5ota,5e9m,5h5b,5t8p,5vwq,5jwt,5ak3,5am
0,5bsm,5cb4,5ygv,5fs5,5sxs,5k0h,5j5f,5r8u,5cs6,5y0z,5khh,5d3v,5v1b,5qd4,5c
38,5nw7,5bvt,5zxu,5ibg,5c3q,5l1q,5d45,5du5,5jfc,5awm,5lub,5hpy,5d7r,5hji,5
ai9,5edh,5i3p,5fmd,5c0i,5c3d,5uwt,5nwj,5iha,5ktd,5zef,5ted,5dh5,5gz9,5l2j,
5eyh,5i13,5i9m,5az7,5few,5mmp,5yc8,5fqp,5ehr,5x68,5ewc,5xxh,5nra,5r9s,5mf5
,5u8f,5nu3,5l8p,5vwr,5dw0,5kjx,5u3w,5jah,5v60,5v86,5z3j,5ir4,5oce,5lbf,5n4
9,5mvs,5x2x,5o9o,5dz1,5of9,5v5y,5v42,5zcc,5mit,5jf3,5mja,5ors,5w3t,5mk9,5w
ds,5exs,5fvz,5m4p,5jzi,5fh8,5fau,5vul,5glu,5osd,5elw,5b49,5vb6,5bvh,5w0c,5
jat,5q0f,5gmn,5y2w,5a58,5f9b,5nxy,5dtf,5fx2,5l9h,5bx3,5f2k,5iqh,5c4d,5aoi,
5av7,5k2a,5qam,5d69,5bxs,5m8o,5a1t,5tzc,5to8,5ofb,5ems,5kaw,5hdv,5teq,5nvv
,5d9j,5ifs,5nln,5enr,5otf,5e3a,5tly,5lli,5uiu,5owz,5r98,5nka,5kle,5wn8,5c4
8,5w78,5dxj,5w8v,5tc0,5ye7,5yqn,5tm3,5e2q,5htk,5vbz,5ev8,5irw,5fbc,5glt,5h
02,5ess,5wa8,5auu,5oai,5osk,5kda,5xna,5vqs,5ckw,5t7t,5t6x,5xmq,5jj0,5hbn,5
zjd,5bxt,5sz7,5f9a,5t4e,5kpz,5zc5,5l3w,5ewa,5dxa,5bnn,5kl0,5r9y,5u1i,5bvg,

5gln,5cxi,5z2p,5dpv,5o9u,5uk8,5j0d,5g0r,5t2b,5uuo,5f41,5diy,5nrw,5zi7,5tzo
,5ot5,5orz,5z7p,5nv9,5ac5,5r8y,5m0t,5v8r,5ktp,5nk4,5fnk,5eu6,5x9o,5mkz,5fu
p,5hiq,5nmw,5je5,5vgo,5j3r,5joh,5yz7,5svn,5opv,5ivx,5os2,5aiv,5eru,5bnm,5u
wo,5g44,5op2,5gx7,5h1m,5num,5zf9,5g67,5gk9,5r0h,5mys,5mzx,5okq,5dg2,5igj,5
acw,5r1t,5ex5,5zun,5jgs,5v9g,5b47,5kv8,5g21,5xiq,5gri,5db2,5mme,5n17,5tsh,
5oya,5zjz,5n84,5a89,5j42,5ave,5woo,5vn0,5k5m,5aci,5l94,5ios,5vp1,5a0a,5iq1
,5ow9,5dvj,5egs,5al4,5qd7,5tlo,5ufc,5oop,5ncg,5dy8,5dk9,5d9o,5ovg,5oul,5g6
m,5te2,5ehi,5zgi,5mwh,5yec,5m5q,5xvq,5dx0,5ytu,5ljq,5hgv,5zw2,5yog,5yjp,5j
di,5hgp,5l9n,5a3n,5lqf,5abm,5tjn,5mxh,5g6c,5oro,5ivq,5swq,5jdc,5buj,5ai1,5
czb,5tku,5v9o,5xc3,5y7c,5ih1,5lrf,5kf6,5jj2,5dyb,5ecs,5j60,5ghi,5u5t,5whu,
5i7r,5tks,5eau,5tkr,5ggn,5zwp,5ya8,5f6d,5fkp,5dr9,5umx,5h5j,5eiw,5z99,5tzg
,5ow7,5w7k,5dju,5oes,5liu,5om2,5tdc,5dnu,5ab9,5uds,5nkg,5e4t,5i9t,5ee5,5b3
8,5l7p,5b5o,5mz8,5u2d,5lnq,5hla,5fui,5tzj,5zbk,5c2e,5n2x,5hp8,5t95,5tqz,5m
1t,5osl,5d1t,5u39,5ljg,5v4w,5d61,5ewy,5mzg,5a2c,5v5n,5eio,5yu4,5koc,5nn6,5
orh,5mbh,5ca0,5ljb,5kqz,5iub,5nz4,5ky5,5yj6,5w8x,5azb,5ead,5l0r,5uwp,5c8c,
5clp,5ap0,5up0,5nut,5r9u,5x62,5c3p,5tyq,5ap7,5ff0,5m66,5j7q,5vys,5vo3,5esb
,5qaw,5bu4,5dmz,5ayc,5hgm,5iqs,5h0h,5jst,5jph,5a82,5add,5j4x,5a5y,5r8g,5r2
d,5otd,5zzz,5mwp,5eyy,5j71,5fh1,5cqg,5fe8,5fpa,5tkt,5lj1,5tuz,5znc,5vyz,5w
0i,5vw8,5o48,5bwc,5m3w,5xl9,5eow,5n55,5u82,5x4p,5m45,5g2g,5odq,5fhm,5dch,5
zax,5fvp,5hm3,5ra8,5h4i,5ml6,5a8e,5kwy,5ura,5j6l,5b7v,5ka3,5htb,5jcu,5ek3,
5aqt,5hhz,5y0t,5tlf,5wau,5qa8,5a5p,5csy,5elf,5h17,5h42,5xeg,5ove,5br4,5nkb
,5e65,5qcy,5eo0,5wdj,5hx1,5c9x,5nk1,5f0c,5jbx,5ncj,5t1t,5ads,5wlt,5idb,5e2
m,5kau,5v59,5ist,5mcp,5upj,5uqd,5nqa,5d9m,5bv3,5zk5,5ysp,5tk7,5fs0,5tzw,5g
oo,5a7n,5i7o,5uny,5wsb,5nak,5wqj,5exo,5zzq,5n4m,5o3n,5eo1,5t0m,5mbe,5fnf,5
m03,5adz,5e9l,5u7j,5yvr,5r0a,5ls7,5d29,5ni8,5k7h,5c1o,5dtw,5jfw,5n2t,5fxe,
5vkr,5xql,5g11,5t9b,5lsg,5cgp,5ml4,5i5v,5x1m,5o0h,5hwc,5ovv,5fra,5bqg,5vkm
,5l3z,5fi8,5xp3,5dqy,5t92,5zaj,5u12,5i3m,5t0b,5igk,5h84,5ext,5mgg,5kf9,5f2
o,5ec8,5wgc,5urv,5ny3,5tqe,5yng,5jas,5ylk,5e5u,5yf9,5uhv,5mwo,5zw3,5aut,5j
f8,5yrl,5ud2,5xn3,5hxi,5tmw,5umy,5x5m,5vtr,5z66,5mfh,5meo,5kcw,5wc2,5jg5,5
hmi,5x3d,5avf,5l7g,5lz8,5abe,5gjc,5l9q,5hvj,5bs0,5i8w,5kml,5ikb,5dhu,5fvt,
5yyb,5ij2,5ev9,5yaq,5ma7,5oo5,5fdc,5gvk,5k6m,5jww,5wdr,5y4r,5xw9,5e7m,5uov
,5j2t,5ybq,5cep,5w70,5c4o,5trl,5fus,5fqr,5ict,5d0e,5now,5xvr,5r8d,5op6,5u0
w,5f8f,5a1f,5man,5k1p,5iug,5u8u,5dn9,5odr,5wg7,5eif,5i0d,5y6n,5ow1,5ipc,5u
10,5cuq,5zb4,5nvx,5glw,5i3w,5in5,5zzb,5zeq,5c29,5kjz,5j8q,5xet,5zxk,5d3n,5
mxv,5lkz,5orr,5czm,5niy,5ak4,5bjz,5xig,5tov,5twe,5dbg,5ngl,5gga,5a5s,5l0s,
5ody,5dlz,5ra0,5d5a,5lyr,5fng,5xlx,5wgg,5m6z,5i3q,5k8s,5v6k,5nu9,5njq,5o6z
,5wed,5gme,5i8t,5f1u,5nc1,5o2f,5k4i,5ymt,5h1w,5wuk,5ebt,5eh7,5fdp,5r0b,5cy
f,5vjf,5lqq,5ky4,5deq,5boe,5cu3,5x4n,5egp,5ljw,5ah9,5lkt,5m8w,5od2,5gg6,5n
c9,5e1c,5tpa,5a6h,5uwi,5hmz,5eol,5ad4,5anv,5ygf,5elv,5dq8,5r0m,5myd,5df1,5
hkb,5o4a,5dz3,5bsx,5wzw,5tlt,5lc8,5f8r,5d2h,5tcy,5c07,5fwj,5o5z,5o5f,5aqh,
5m4h,5r96,5mzz,5xai,5fdg,5na5,5kow,5l8k,5hlw,5lyg,5aa8,5him,5bk9,5cbi,5o2t
,5ybl,5pzk,5jaz,5a69,5fpv,5al1,5v32,5alx,5ka7,5mhq,5dz0,5x24,5byz,5cb8,5oc
t,5e0x,5a94,5hd7,5uli,5ajy,5k9w,5krm,5ak8,5lyk,5nw0,5alz,5abu,5bu3,5r1w,5j
in,5w85,5wap,5xsj,5adi,5kas,5nb6,5zcm,5n5s,5xvv,5e9q,5kqa,5tmt,5xva,5bxc,5
c08,5glq,5hni,5b0s,5qaj,5iwe,5jvb,5zz6,5jez,5r89,5y1b,5y8y,5dw2,5ia3,5yg4,
5w8d,5nzp,5ve5,5qai,5lm3,5vub,5fiv,5uww,5kad,5xd1,5iu4,5kqy,5d2b,5ec9,5ntp
,5fh4,5ty8,5ijz,5f3e,5yg3,5fog,5l2z,5ekb,5h8h,5gjh,5fpo,5otz,5gvr,5zrs,5zg
0,5tc3,5ive,5d47,5j1j,5kxh,5j4h,5hs1,5ot9,5xla,5ow5,5nvc,5xxo,5jes,5flg,5i
oq,5iak,5gzz,5lkc,5ypi,5hil,5j55,5r8k,5xfi,5fow,5r9b,5jeh,5odx,5ulj,5ej5,5
ai0,5l3v,5aqw,5i8g,5wct,5yvm,5alo,5v8p,5xsu,5nti,5jcl,5tvm,5x72,5fag,5g2o,
5v6s,5a61,5dby,5o43,5dwe,5ou3,5uo3,5ikr,5pzl,5x2c,5cty,5h06,5fqk,5mel,5brd
,5mut,5yjw,5qal,5aun,5adw,5l11,5f3d,5kzv,5hwp,5tmv,5fi7,5bww,5xnh,5n0s,5va
c,5l6d,5uc4,5lrk,5mil,5fpz,5ync,5g4j,5fh2,5owh,5hn9,5jjr,5awb,5fe3,5ju9,5h
ra,5n6n,5w7y,5c7d,5wfz,5mk4,5wgz,5vq4,5c2a,5g5g,5k14,5mgf,5ajp,5m8x,5u45,5

zu2,5y77,5yfs,5isq,5nmx,5coj,5brp,5ws7,5c1g,5t63,5i35,5a6n,5nh8,5tt3,5dc5,
5drc,5m3e,5tzd,5mat,5orj,5u8w,5o70,5bvk,5vbe,5omr,5bqx,5ajm,5lxc,5aic,5xvk
,5b30,5of1,5b4u,5orv,5n9l,5voj,5dxt,5th4,5etn,5lwo,5dva,5z4m,5igt,5a0y,5zw
8,5kos,5dkq,5vfj,5tlp,5i3b,5g41,5h09,5rge,5l0v,5hc0,5wbx,5hid,5xmx,5l1t,5e
jl,5lu8,5ufe,5mb5,5tnp,5ndf,5e58,5tlg,5mo7,5wbf,5i88,5w5r,5i60,5dt6,5odh,5
o2n,5cap,5syj,5e4a,5krt,5nhj,5ttd,5ma2,5jvi,5a0u,5oms,5m7g,5bmv,5ti0,5wdo,
5o96,5zbz,5evd,5iot,5ut5,5n31,5aig,5tkk,5f90,5vnn,5li3,5ixb,5yse,5ncp,5iua
,5xps,5wl1,5flt,5vh4,5fl5,5sxn,5g20,5yto,5cf5,5vq1,5kat,5vks,5nho,5zys,5db
3,5n26,5h47,5eeh,5ut2,5dpe,5z74,5kp3,5uim,5tbm,5dgh,5d3h,5fsi,5ynh,5ybr,5z
rl,5a8q,5e9g,5zzr,5tzx,5h14,5r1z,5mxb,5n5k,5mqv,5id9,5ury,5v3x,5a7o,5oth,5
k50,5o3a,5vbm,5ox5,5n93,5o9r,5cpb,5cah,5bqu,5oq6,5uns,5zi9,5g09,5dyc,5fan,
5fit,5ca1,5c4u,5all,5c7t,5dm4,5fhf,5tbe,5kxq,5t1u,5dqf,5h1t,5f33,5e88,5w7b
,5nib,5fpd,5k3e,5vq3,5bwh,5grd,5vjd,5a0f,5ds8,5vra,5aog,5elm,5qb0,5kro,5ug
h,5c1w,5mx4,5gny,5hkn,5t36,5ufr,5xj8,5gon,5ox0,5l9v,5cu6,5jid,5iar,5w8q,5x
ij,5f35,5lma,5mon,5fs9,5yt0,5xk7,5p21,5c49,5wmr,5nh7,5ar0,5cr5,5d3s,5uoy,5
h9s,5tde,5mfs,5alt,5zqk,5g4s,5kkb,5i3x,5g3m,5cr1,5yif,5e3d,5ncd,5hmr,5j6h,
5anq,5b0i,5h9n,5oog,5y8c,5a1r,5oks,5yoj,5hrv,5egu,5lix,5u62,5lz7,5clu,5igv
,5di3,5ufx,5bsr,5xoi,5aau,5a0b,5fb3,5auz,5etm,5xvx,5m7s,5alb,5uew,5ng1,5tb
o,5niz,5al5,5mn1,5nji,5kmi,5veg,5kr8,5nth,5ecv,5t78,5mwz,5hs6,5vpz,5zfj,5a
6m,5t1s,5eg4,5zbr,5dd9,5fry,5a3t,5aky,5kts,5ey9,5kup,5e8b,5fom,5iav,5u75,5
nk8,5ci7,5m3u,5t70,5dw3,5lxh,5xwc,5x0b,5wxb,5u3x,5dnd,5omy,5fum,5ndu,5n5t,
5hm0,5gho,5m57,5u5z,5tm9,5lbb,5jad,5fwr,5jtt,5ouu,5nn8,5b09,5adn,5uo0,5g4w
,5o11,5o0s,5y8o,5zj5,5yea,5aml,5e62,5xhk,5dwj,5v97,5ei8,5jwa,5vgy,5z5u,5os
s,5r08,5lwy,5i5u,5ofk,5axh,5oxm,5gic,5duw,5t7n,5vgr,5am6,5oq8,5mfg,5r23,5l
9o,5opa,5c4e,5gji,5jcw,5w58,5i4n,5zbl,5aoc,5eb4,5lyy,5jib,5kdh,5o0x,5xco,5
oeh,5fes,5ibe,5abw,5aee,5en3,5exa,5aal,5hz9,5vcg,5y1y,5t4f,5dvi,5ysr,5kut,
5hua,5jzs,5lwm,5r20,5x7z,5ex3,5iql,5a04,5ttj,5oe5,5b36,5aho,5mr6,5kmn,5osf
,5zwi,5z5i,5unw,5cf8,5eqs,5mua,5c1d,5yfj,5a5n,5hik,5ae9,5iuy,5uy9,5dnx,5m4
u,5t9z,5xdr,5hrs,5xzf,5e97,5qax,5g48,5tt5,5tld,5nhc,5iyz,5ynd,5ylf,5uey,5v
x5,5agk,5ezz,5l9r,5osx,5tf9,5mw8,5k8b,5dwq,5r9k,5bkb,5hbs,5fso,5jf2,5z9q,5
lbc,5w19,5y8w,5igw,5dtm,5a95,5tob,5om1,5voo,5nxw,5eae,5cou,5htv,5fcf,5cj3,
5c2f,5bwm,5e9h,5xdt,5ees,5l8y,5om7,5z2r,5mah,5jhd,5d9g,5nsw,5ko8,5mof,5kr9
,5ak0,5xkr,5g01,5yvs,5i91,5o0d,5ha0,5mfz,5alc,5ivg,5aab,5bp5,5dbx,5ap1,5u6
j,5cok,5qd5,5kpk,5gg8,5lz9,5dp0,5zrh,5iqn,5jfl,5a19,5ih5,5td4,5qae,5opu,5c
c2,5bpp,5kor,5w8k,5fsl,5j61,5ov9,5b5s,5b4l,5bsu,5mes,5v41,5otq,5d7o,5g66,5
d6q,5ac1,5lxm,5nmh,5l6g,5vua,5f01,5lh8,5hzn,5dxi,5g40,5fub,5l83,5ik0,5eqp,
5r1y,5ag4,5wzn,5vom,5izf,5mi6,5r9d,5e89,5x6q,5e2n,5kvi,5ad1,5xof,5t9w,5odu
,5f5z,5lgw,5hgd,5fc5,5zct,5lof,5o9p,5kpg,5tqh,5n99,5igz,5t2z,5a1s,5mw6,5j2
7,5mrb,5ama,5eci,5uz8,5h8f,5ln2,5m77,5d59,5t55,5vp0,5o2j,5opk,5drd,5imp,5k
d1,5hlb,5m5b,5lu9,5krd,5u6c,5ysf,5k75,5m2a,5k6a,5fp7,5nkd,5inj,5ld8,5fks,5
qd8,5evy,5ivj,5l2t,5h62,5afk,5e5h,5w2i,5ng7,5u9j,5mf6,5n7m,5jv0,5n82,5je1,
5je0,5jf0,5nva,5awj,5nau,5cl0,5a4u,5mbj,5g0h,5kf7,5moh,5uc9,5lne,5qa6,5fv9
,5nhe,5w8g,5a3x,5xvt,5z8k,5k4j,5w75,5w0e,5g5z,5vgd,5mgz,5mxm,5mt4,5aza,5iv
b,5aqz,5vrh,5ths,5twj,5tjk,5y1f,5m4i,5nqb,5eie,5f25,5adb,5i2f,5lv1,5xkt,5w
gt,5wqo,5cwa,5jar,5fad,5lrj,5tda,5e7n,5zza,5u9d,5jft,5o74,5css,5akj,5f5a,5
dgw,5mgn,5ggo,5l7t,5l2n,5f6x,5ooi,5i89,5i5e,5nsf,5yvz,5ooh,5n6q,5c8w,5c3a,
5dax,5gz6,5kpr,5o90,5iq4,5w6o,5lrd,5ofv,5a7p,5vpy,5kvs,5b39,5mze,5uxh,5mhb
,5zpr,5mdr,5sga,5k21,5txy,5n5d,5cit,5j1u,5h8e,5xh2,5gmz,5ih6,5dls,5mkr,5iu
v,5lq8,5gnx,5agl,5fhn,5my8,5vd0,5a09,5kso,5w6e,5eyk,5a5v,5een,5ik1,5egh,5n
9p,5p9f,5u40,5tmo,5oji,5eqe,5pgw,5qcz,5duf,5ost,5efw,5nuc,5utr,5j54,5cae,5
uo1,5ali,5h9l,5e14,5oa2,5u7k,5coi,5cvd,5wri,5e6e,5c0f,5htl,5x54,5brf,5htc,
5m0m,5af9,5lsc,5f62,5tn5,5cew,5w3x,5f3c,5ibh,5iag,5kvm,5y2c,5d2j,5fxc,5ggz
,5r0c,5nci,5le1,5z0a,5n53,5tui,5tbu,5qtv,5ggl,5nse,5cf6,5n0e,5hjs,5chq,5z7
o,5tmz,5a9a,5fs7,5mlh,5mfp,5o7v,5n7o,5lz4,5tuo,5dwr,5dgi,5fqh,5cpp,5aep,5c

28,5qau,5w3k,5o13,5v03,5qc4,5ej8,5ej4,5nle,5lot,5eq9,5u38,5ujj,5iae,5f1r,5
oc7,5nhl,5acn,5e6h,5jop,5ior,5ddf,5igp,5kyt,5tmm,5ojl,5mym,5ej7,5zg3,5c9r,
5dxe,5j8u,5bsb,5vpw,5mex,5yj1,5jc1,5jq0,5hhf,5emn,5khi,5c79,5lmd,5mjk,5o4n
,5fbd,5dx3,5l4m,5mkg,5h0b,5hcy,5qda,5zqo,5uqx,5kz0,5eqy,5zbf,5ee7,5h5g,5j8
2,5agm,5jmn,5c4t,5o86,5dde,5tm5,5mo5,5wbz,5luu,5j5y,5vyy,5ufq,5l1s,5isl,5j
sr,5m12,5f63,5dce,5hf9,5ede,5fe7,5u5d,5i8s,5uxo,5nd0,5uzk,5qd2,5v21,5n7q,5
v5d,5k4z,5xtv,5w11,5ugf,5exe,5w67,5w2o,5eqb,5o3h,5jbf,5dzt,5e13,5hz7,5kgh,
5hga,5ddu,5ae8,5cpl,5ei3,5r9r,5d4a,5eo8,5e9k,5bwt,5ncf,5gqi,5icf,5tt6,5ehz
,5h56,5yvt,5d3x,5r8j,5axq,5nor,5zzx,5mo6,5tlm,5m6x,5u3z,5o7a,5dmc,5ny1,5mz
y,5gw7,5fpu,5ws9,5a85,5wk9,5z84,5evb,5xd5,5f6u,5fwe,5nlr,5t0a,5nrd,5l9b,5f
ov,5diu,5ago,5drn,5y0p,5ha1,5h68,5hi3,5wxi,5gp7,5g22,5tyr,5hpz,5g1n,5v9u,5
vdb,5g62,5ift,5dtt,5tto,5dlx,5yfw,5htp,5e29,5a7q,5k2d,5nit,5dyj,5y7t,5hjq,
5fp8,5dlr,5xtl,5d3t,5web,5tgu,5xzh,5hkg,5abg,5yd4,5dig,5o8h,5d0c,5o8d,5f9e
,5cbs,5fq0,5y80,5mgd,5hww,5y1g,5x80,5vuq,5xt0,5xdv,5nk9,5lrt,5e12,5x1v,5mb
x,5moo,5t8g,5ep9,5ra2,5a6b,5ti5,5epy,5y2o,5mc8,5d58,5jd7,5yfz,5zj6,5fwa,5c
tp,5wbo,5v34,5ipy,5m9w,5hv4,5cpy,5nul,5lbw,5j1s,5xd2,5guf,5y0g,5jau,5vih,5
oa7,5kvh,5u90,5ng9,5cga,5apg,5z5s,5lfv,5fez,5ufp,5ky7,5wzs,5oif,5y3n,5axv,
5t85,5flr,5zrr,5lh4,5m1z,5mis,5l4n,5bwx,5kao,5w9d,5kgw,5hoa,5mqw,5yl2,5hdz
,5hcf,5mih,5uda,5o9d,5nve,5wsy,5cpo,5owk,5vfc,5tiy,5dx1,5vw6,5vp7,5aia,5ng
r,5aph,5hva,5hu9,5aip,5ta4,5ntd,5za8,5je3,5g6j,5u1q,5lht,5j62,5ud0,5sz6,5s
zh,5gvn,5ixh,5jei,5fhr,5f7y,5ylt,5jxn,5lwd,5mfv,5njb,5tiw,5ert,5kr7,5x3r,5
mo4,5fii,5dkk,5krq,5bvn,5u8a,5zco,5flp,5g55,5y86,5kd7,5rgd,5r21,5r99,5gx6,
5ulg,5gm0,5uy8,5am3,5g3j,5n8c,5kbe,5v5e,5m8z,5h4j,5tbt,5isy,5kzd,5vvb,5aoj
,5zwa,5hdx,5ucz,5mrd,5u2n,5j32,5kd9,5hgh,5wxk,5lpm,5fyr,5c0j,5lx6,5ofw,5h3
9,5n2f,5exd,5vyh,5b19,5sz1,5eps,5w8i,5yj8,5u3e,5t0k,5jf6,5c5h,5ni4,5ddd,5a
qk,5c0g,5mqg,5fh6,5otl,5je4,5l8l,5yie,5fgk,5ory,5n0p,5fqd,5ktc,5dro,5us4,5
g10,5d0r,5u86,5orn,5e98,5abx,5dxg,5x8f,5wm2,5m0e,5xsn,5w2q,5ysh,5tpr,5foi,
5oc8,5a86,5h1v,5ftb,5uca,5niu,5mqk,5h9q,5xnz,5z6k,5iid,5lvl,5cpn,5hlp,5svv
,5l6f,5e4o,5hrq,5c9u,5eo7,5t6m,5koh,5jgk,5vuc,5mb4,5igi,5ubg,5r8b,5kra,5vv
0,5vuo,5vx9,5zb0,5jhu,5d8u,5e2l,5o4h,5myw,5e9w,5cku,5otn,5u5v,5h7j,5hz8,5c
fm,5lrr,5aln,5cnn,5jfu,5eyw,5fmb,5dkn,5n20,5acm,5xl7,5xd4,5vbc,5os3,5xmv,5
ynu,5y93,5t3o,5wkc,5ji6,5o1i,5h3e,5iqi,5eyc,5gpg,5dy7,5wc5,5br6,5alm,5jri,
5oca,5iis,5o7y,5dz2,5edl,5e1s,5zge,5js3,5t05,5jim,5adq,5i0g,5ose,5hhp,5ldg
,5zri,5z6m,5rga,5jsg,5ntn,5n0v,5r9g,5ovh,5dyw,5wqu,5f1h,5u2b,5zxb,5ocg,5t4
j,5btv,5vkc,5im7,5fv3,5azf,5zm0,5pah,5hda,5zzy,5e8o,5zi2,5v31,5n05,5de1,5a
i8,5eq8,5e2b,5ihc,5dgn,5ubi,5vuz,5w6a,5njr,5yob,5nke,5flj,5m60,5b48,5brn,5
os6,5wgp,5ycr,5mvc,5zyn,5eu3,5db1,5qav,5m37,5o6h,5tvi,5tv1,5zi4,5lpb,5o7f,
5exl,5fmm,5lov,5wgi,5wgm,5tg3,5fun,5ugl,5ud3,5z9f,5i0e,5lwp,5ly1,5gql,5ljc
,5n4i,5o3c,5e6o,5wr3,5vxo,5b0j,5u7l,5i96,5ism,5mn0,5w0f,5jca,5d9x,5w2p,5ch
7,5m4f,5dyg,5omu,5av3,5oi5,5n7j,5c6c,5nby,5etz,5tv6,5x8b,5mtt,5zrk,5e3g,5g
0u,5oia,5z6s,5or8,5u98,5cpm,5olz,5al8,5zqs,5gv7,5eto,5ev0,5jp9,5h5l,5ewk,5
e1e,5r22,5ljp,5hq8,5z69,5ivf,5wkg,5c88,5a5f,5m91,5hct,5isp,5j3z,5jcp,5d24,
5y79,5xkm,5dit,5xh3,5tdt,5am5,5vpt,5lq1,5fqv,5kqx,5h9p,5qct,5i3v,5orp,5n5w
,5tw2,5c5t,5epn,5kvq,5mq6,5fd0,5ax9,5vuw,5ldp,5upk,5mir,5zjy,5dlt,5j7z,5ip
a,5e95,5m92,5jq2,5tk8,5wa9,5g6h,5wiv,5nf7,5k7k,5lua,5duh,5tjl,5fu4,5njs,5t
7p,5n8v,5e2a,5akw,5whr,5wbq,5x0k,5enm,5mnq,5u0v,5owd,5svf,5c36,5mzj,5jt9,5
r0e,5tml,5fko,5vyq,5mtr,5cop,5nu5,5yri,5m7e,5n4q,5zfa,5p9g,5la2,5x0e,5oor,
5kxe,5a9p,5afh,5jga,5ccz,5vo4,5l2y,5x7r,5b2f,5fe4,5la1,5xjn,5njp,5qan,5ekm
,5hjr,5msb,5ita,5ccm,5yap,5alg,5k0a,5cpw,5iuc,5hym,5xt4,5hx8,5bs4,5d9l,5vq
2,5eso,5gwt,5j7f,5bo9,5bst,5f1v,5omk,5ksz,5mw2,5mue,5j64,5o9f,5lwt,5fjn,5a
96,5l3u,5tkj,5zz1,5sy2,5l4j,5las,5n7t,5xpm,5fix,5fp3,5k3w,5vfm,5mst,5l4r,5
qa7,5ja3,5tmq,5ip6,5i0f,5urz,5fre,5abz,5jfr,5mui,5a80,5qdd,5f2b,5a2b,5gqf,
5n0f,5a64,5fh7,5lx9,5esu,5xsw,5v6f,5nu1,5ni7,5mem,5qb1,5nxg,5x1k,5hmy,5khg
,5dj3,5yqo,5cjn,5ouf,5faw,5nnw,5byj,5l7y,5tow,5cyv,5z20,5ku1,5r0j,5r29,5qa

5,5az8,5o1u,5ktt,5oic,5ie2,5wf3,5hrn,5jo2,5cna,5uff,5mxg,5agp,5a7v,5dx4,5v
m6,5uws,5k13,5akx,5xm3,5due,5wfm,5hmh,5cf4,5lyq,5zag,5bn3,5cg2,5jlj,5y72,5
wzu,5jiy,5ab3,5lws,5icp,5vyr,5w8j,5hz5,5xv7,5ln4,5alv,5h20,5da3,5khd,5fmc,
5m55,5nwd,5oq7,5yt2,5my4,5iex,5gwx,5azc,5nue,5hdj,5f09,5n1x,5htx,5hwu,5a59
,5igl,5f3i,5kcl,5acj,5m19,5hc2,5m17,5mku,5lpg,5oe3,5tq7,5h5f,5eag,5msc,5fo
x,5ysq,5awv,5grf,5k1d,5iqv,5k7w,5apk,5l2r,5ai6,5dnk,5uck,5wha,5dyz,5z9e,5t
t8,5mxc,5hyk,5op4,5oxn,5lu6,5ufs,5x4q,5u6e,5olb,5hoh,5dpf,5do8,5nfz,5h41,5
hv7,5dt3,5p9h,5whx,5iaj,5qcl,5kf1,5x2f,5fni,5b4o,5wi1,5z5v,5zcd,5jm1,5maq,
5ih8,5r9h,5nju,5vvc,5hls,5agj,5x5o,5ikt,5t8u,5n68,5ofl,5yrs,5u83,5ybt,5i86
,5u5y,5iqg,5f2p,5itv,5dya,5g3t,5z0s,5e15,5i1q,5x93,5hhn,5tlu,5tef,5hx6,5og
x,5okg,5b3y,5fqt,5lnz,5igy,5cx8,5i8f,5ucn,5g6b,5ad6,5ech,5nua,5dxr,5fbg,5f
4b,5f12,5mkx,5exz,5vuv,5hzx,5h0g,5nhd,5a4w,5fi2,5eoo,5hvt,5m5t,5r0g,5r8i,5
sun,5qa9,5jfx,5v24,5nld,5e9x,5u0e,5na1,5wyz,5mmg,5mh1,5m04,5tel,5m69,5vop,
5nty,5eb3,5lzh,5nu8,5wfc,5d5q,5wqa,5mi7,5g1x,5vq6,5tqf,5j59,5nll,5v7t,5ufw
,5tlb,5igr,5iok,5i8p,5kb3,5qag,5a5a,5ovr,5o07,5vam,5f0x,5t79,5g4c,5a60,5l2
m,5f61,5tza,5n0j,5v22,5nge,5tm8,5mm3,5wqk,5ia2,5u5h,5vii,5hvy,5qcw,5zs3,5o
cj,5v2y,5x1l,5fah,5uat,5jtu,5ctw,5o72,5ad9,5myo,5te1,5fe0,5rhn,5h6s,5nkh,5
en4,5lu5,5yrg,5mrh,5cau,5k7u,5oax,5f03,5ayv,5hh0,5g0i,5r8c,5egm,5mul,5bsy,
5wl0,5bnp,5fzj,5fck,5f6v,5kjy,5dyu,5n0x,5boy,5wmu,5bsw,5ehv,5la6,5icj,5wyx
,5nw2,5ntk,5bsg,5ukl,5bnw,5r93,5nae,5mzc,5tzf,5yp6,5drj,5mzm,5otm,5i34,5t6
s,5wmp,5ai4,5u5g,5zqp,5juz,5f32,5drb,5foe,5tll,5g69,5vse,5ihj,5ab6,5fsm,5h
n0,5ugi,5mge,5z7b,5f1x,5kxc,5ysi,5qik,5w8a,5aok,5t48,5wvr,5ant,5lz5,5bya,5
r9j,5h63,5oto,5it1,5av4,5nhp,5gg9,5h9i,5er1,5uqm,5o1a,5h8q,5mvv,5afn,5lu1,
5et6,5lyh,5ik6,5i5y,5i2e,5kax,5j6n,5a1p,5unt,5gqx,5r26,5k1x,5moe,5ui0,5vux
,5zdq,5fqj,5eyx,5alp,5n6g,5ue4,5ybn,5krf,5vcm,5ap6,5o1e,5zxi,5tdd,5noz,5lb
5,5luh,5iqa,5zf8,5gue,5n9t,5u13,5n3y,5d6c,5y8h,5b3w,5nxp,5drr,5xuf,5fto,5q
cs,5olg,5kag,5mzn,5ieg,5yqw,5ibc,5fnd,5wa0,5ra4,5vp9,5lpj,5anw,5x14,5wb6,5
ypu,5tk6,5ekh,5obv,5ehc,5kam,5m2f,5a07,5ktr,5orx,5upf,5u3q,5vsb,5ad8,5iee,
5zzp,5cpx,5k2c,5xz8,5mft,5ky3,5n67,5fxd,5v3y,5he7,5aew,5xdw,5wg4,5t2u,5n2z
,5hrc,5kto,5gsn,5nk0,5bsf,5oi8,5r8z,5x8c,5o4f,5fkl,5tjo,5jxz,5osy,5li9,5h9
y,5y8m,5jyy,5e3q,5dgu,5jdy,5u0g,5fkm,5oby,5tqg,5v6v,5vmg,5fbf,5e7o,5nii,5v
c3,5exw,5dtk,5zqy,5d4v,5e2s,5wdy,5ez7,5g0s,5mwy,5os8,5tmu,5bjy,5gqj,5hf3,5
vw2,5jan,5t8o,5n4s,5mwd,5ved,5nlk,5i9b,5n1p,5ouj,5jsq,5fpk,5xvg,5ioz,5csh,
5ugm,5h5h,5thz,5xly,5af7,5l4s,5ai5,5fut,5qaa,5b4b,5lgo,5dnr,5a4c,5naw,5ugw
,5jp0,5nsx,5fvo,5g42,5ca6,5zpq,5axp,5oo9,5byk,5tc4,5kby,5tha,5a27,5u3t,5bm
y,5oei,5vnf,5lj0,5jh7,5j86,5fs8,5nd5,5fep,5m4c,5uec,5agn,5i9l,5jbq,5jmw,5j
g0,5p2p,5m27,5cmb,5r9t,5aib,5gud,5uv2,5two,5op5,5lsv,5iuh,5ezx,5iwf,5k1f,5
alk,5ezy,5vq0,5wr2,5n3w,5uit,5d3q,5fzn,5enp,5ti6,5vje,5w10,5qac,5nlq,5gss,
5l7k,5zqx,5bru,5ele,5r97,5fcw,5hmc,5xha,5eyt,5rgf,5gsv,5fsk,5cph,5aux,5za9
,5ou2,5kit,5vl0,5lif,5t6w,5ze6,5kgp,5qao,5jwu,5t4k,5he4,5ivc,5hhw,5wsc,5x8
g,5d3y,5he5,5oa4,5qd1,5y8k,5n16,5ayi,5xpi,5m24,5z6l,5uis,5d21,5zab,5jle,5g
of,5i3h,5ode,5l13,5eqq,5ajz,5qcn,5a06,5svg,5ixk,5igm,5myt,5deb,5z2l,5twk,5
cte,5d7e,5hkr,5xl3,5mw3,5jbi,5epq,5hfa,5tfx,5u8c,5xdu,5rgb,5vuk,5os7,5mar,
5hpu,5msu,5dqw,5r9c,5i49,5w7d,5xuj,5y7r,5hkm,5v87,5xmu,5u18,5u1e,5n3v,5fls
,5fe1,5wqp,5ii1,5odt,5fl4,5nt0,5tms,5ne5,5mtx,5f7u,5ej9,5sxq,5o4s,5uj3,5js
d,5y6d,5xc5,5fbi,5lyx,5nt4,5je8,5kpt,5wqc,5j6c,5i93,5ezp,5vkb,5l92,5jkg,5o
59,5cvs,5d0g,5wgv,5cu4,5cpr,5r1x,5l1o,5ccc,5vc5,5vl2,5buk,5hcl,5dwi,5va6,5
jgl,5zec,5i7u,5j14,5u8v,5m96,5ngz,5fi3,5xsb,5nk5,5jcj,5kjm,5cp4,5wlb,5i29,
5yjf,5m36,5lxi,5mxk,5ov8,5upe,5w49,5ns9,5m53,5hi5,5ndb,5fnu,5nqq,5d1s,5l9l
,5ye8,5a8a,5r9w,5fal,5brt,5hss,5k7i,5u7v,5frd,5nsp,5crm,5uz0,5jjy,5e8j,5dv
p,5kmj,5faj,5ikq,5cge,5opc,5jye,5lmk,5qd3,5wx7,5ys9,5ncz,5ibr,5fdi,5iru,5d
tr,5hip,5nea,5am2,5dxp,5enz,5a0c,5fou,5jjs,5bre,5kr0,5ehq,5ak2,5uwu,5hbr,5
dxk,5a2k,5f4n,5ows,5o1j,5ufn,5kcm,5wmn,5owt,5we8,5o7c,5ojg,5e7g,5t52,5ekj,
5ugu,5h86,5nkn,5j1r,5wp4,5mfw,5tm4,5zpt,5ggb,5i12,5cfp,5n0n,5lgv,5cmc,5vc6

,5goz,5nk7,5oxo,5jsm,5afx,5vit,5ii4,5fqb,5uez,5f74,5ghj,5u55,5lz2,5z8a,5ae
i,5oje,5nn0,5u3u,5z2e,5bvc,5g03,5qtz,5ixg,5yrf,5ghn,5yok,5jq8,5eg2,5mli,5o
ye,5fsx,5oti,5van,5hux,5r90,5o9w,5bk7,5d7q,5wp2,5x0f,5lmb,5c4l,5xw0,5zai,5
bqs,5oel,5etq,5u42,5o2z,5isz,5aye,5o22,5nb7,5nhy,5n66,5k4u,5o9x,5u7i,5eld,
5yy6,5cqj,5gnz,5no9,5nab,5rgc,5m8y,5uwj,5jq1,5kv9,5ea2,5mo8,5m56,5nwi,5w7i
,5gal,5dm0,5nnt,5lqm,5oyf,5syn,5i9z,5bjx,5lud,5x7p,5v7p,5mb7,5aqp,5h1u,5yi
b,5h94,5adl,5ghm,5xs8,5pzn,5ois,5lgd,5ia5,5wiu,5n1z,5u69,5j1t,5b3z,5u32,5z
r3,5kzy,5n0w,5c7a,5oj7,5yxh,5gv8,5uad,5l2o,5lre,5osz,5w7j,5nls,5r86,5bkc,5
vd6,5w1e,5ae1,5vvn,5ubh,5am7,5age,5uo4,5fbk,5hwo,5irr,5yli,5n0o,5wm5,5bwu,
5fxl,5opb,5ej6,5f37,5dcu,5qcv,5mer,5tzk,5ops,5mz3,5xia,5e2r,5zee,4ywd,4m00
,4kpm,4je8,4oz3,4hn2,4l7p,4op7,4xmb,4xke,4duf,4cif,4pm0,4l4g,4plt,4z3k,4pb
g,4l5b,4epp,4ql3,4jmu,4oi4,4ws3,4x6o,4dnj,4ocu,4okp,4dgb,4i0b,4brl,4xjp,4b
0t,4zij,4k9w,4w8c,4epy,4pg0,4cd3,4j82,4dli,4e2w,4tim,4xue,4jsl,4bdb,4ucn,4
btv,4rdn,4zno,4z6q,4ish,4pyq,4dec,4hxw,4ebz,4cgo,4ban,4cw5,4p0v,4wei,4leb,
4nji,4iuo,4o8a,4ivw,4nz8,4xoi,4rei,4l6h,4ih3,4de5,4qm9,4a41,4eh4,4ey5,4b2h
,4zhg,4mcb,4mj0,4k9y,4au1,4nm7,4k77,4npt,4pg9,4h39,4qmy,4d0g,4amf,4edt,4e5
m,4b2g,4em7,4hzm,4pv0,4bbf,4gzw,4d89,4lwa,4zzu,4fg9,4xdm,4gb9,4y3o,4qt3,4w
vl,4ase,4udc,4cj3,4au7,4xzm,4qjw,4yh3,4k38,4yl0,4bca,4nda,4hw3,4yxu,4mof,4
z87,4cx6,4c9w,4dru,4r59,4unp,4ikz,4ubt,4gbp,4x17,4idz,4lbu,4q4y,4apu,4ep3,
4rkz,4gdp,4ia6,4jib,4jpe,4wvc,4u1j,4bam,4kos,4cb4,4u0o,4mrf,4evr,4wkb,4cw0
,4c4n,4zec,4q6d,4jpa,4yty,4i2p,4lgy,4b7g,4c4j,4apb,4zh1,4ma7,4qwt,4rm2,4tk
j,4kgz,4ckr,4jz8,4isf,4xbt,4d7h,4pio,4gvs,4iih,4wzt,4kmv,4oxi,4c27,4cjt,4k
d1,4idc,4ryl,4n5m,4or7,4elf,4fe6,4dpf,4igt,4tw9,4rfd,4cqk,4nc7,4gmz,4p6t,4
zvk,4y0q,4mmb,4b7f,4lbk,4zpg,4bud,4djr,4okt,4bw1,4lo1,4jh5,4l94,4m7b,4wyg,
4to3,4d5g,4ey6,4mmp,4ij6,4r5b,4kub,4hdb,4loc,4pum,4dkq,4dg8,4pi9,4v2z,4rrn
,4x24,4my2,4p8x,4x5c,4blv,4bua,4co0,4j49,4yed,4e1v,4gr0,4xx3,4k47,4o2b,4lo
q,4i72,4u82,4imo,4uch,4uak,4umb,4c7y,4ogq,4n49,4kqk,4np7,4tmz,4zoa,4nr9,4z
4w,4d9e,4owv,4ejj,4xh6,4lut,4b4g,4xv7,4ef6,4ywy,4pno,4geb,4a2z,4v04,4q01,4
h04,4tww,4kbk,4l8f,4cgh,4o9e,4wdd,4xzn,4fvw,4z24,4rjk,4ixt,4y6q,4l50,4u1i,
4cpx,4ix6,4tz3,4mo2,4c9g,4zlz,4kor,4ctv,4cjr,4cx3,4zb5,4zb6,4uix,4d02,4f9c
,4jfo,4mux,4nbk,4m81,4ptz,4wvg,4ajh,4z2i,4dql,4zn7,4ym0,4hgg,4l7n,4i4b,4n8
e,4ax9,4x6x,4i50,4d4i,4lam,4lye,4foy,4gi7,4xjo,4uxq,4nte,4uz3,4qek,4wb8,4j
a1,4tmw,4fl8,4b9k,4oy3,4yxm,4knb,4qui,4ocp,4jm9,4dmr,4a9k,4mi9,4ih5,4bt4,4
l7b,4k5h,4r37,4c1q,4wmz,4ows,4prq,4uma,4rgc,4ekp,4a03,4ghi,4yt7,4yes,4a00,
4l4j,4gqn,4hgo,4hoh,4k3h,4lo3,4h97,4w1v,4u2s,4fee,4d8c,4xdr,4mkg,4w1o,4u95
,4de1,4e1m,4ivb,4wea,4lbm,4d4c,4xbn,4py2,4jd0,4uvw,4gz3,4yhm,4qmo,4yo6,4ot
i,4r3l,4imf,4tsx,4loh,4jyd,4s15,4nbt,4n4v,4d1x,4cv1,4fii,4ne0,4cea,4tro,4p
hu,4dem,4kn0,4gk8,4l7r,4hse,4jlt,4w4y,4gyw,4abw,4og8,4ock,4fx6,4gmm,4nv1,4
luo,4naq,4zyz,421p,4mv5,4x5q,4z84,4d7m,4mjq,4ofu,4tl6,4q1e,4yuw,4wes,4ucm,
4p2n,4is9,4g8r,4x9x,4jui,4qle,4rr7,4dew,4q4s,4b7b,4fms,4ajp,4uz9,4xdo,4g27
,4da7,4qm0,4a69,4rx0,4u8j,4otj,4cig,4npm,4v3v,4lbt,4qpm,4emw,4p4m,4edg,4gu
9,4zx2,4nka,4dqh,4c4p,4n9e,4u6s,4yv0,4ixh,4a9c,4uvt,4d0x,4l5l,4m49,4piy,4y
jr,4moj,4gqb,4y9g,4lbr,4tw7,4p5f,4cfp,4r7b,4eb3,4xj2,4lbo,4rrk,4tmv,4u2z,4
bv9,4x9r,4rsl,4ihi,4h6q,4w4w,4lbn,4yhb,4mi6,4app,4z9b,4owo,4c68,4jop,4yqh,
4buf,4yv1,4agl,4lh0,4qat,4qrq,4fdp,4wca,4kch,4y1b,4p3r,4hy2,4j7d,4alg,4ynb
,4plp,4he1,4w54,4pss,4m21,4jbi,4z1k,4mvk,4d1j,4ha6,4z46,4hx1,4a45,4hk6,4gn
p,4aro,4ibg,4czj,4bkt,4i8z,4emt,4paa,4pne,4xpl,4v31,4w56,4f7m,4i0e,4usa,4n
h7,4l1x,4nmc,4utu,4pmm,4nbu,4f9y,4wr4,4dvr,4quw,4q5m,4xcp,4j0p,4zx3,4lm2,4
erk,4h1y,4yux,4cd5,4cne,4u62,4m5i,4wkt,4ijh,4eqs,4lqy,4k28,4hau,4wza,4ws7,
4l2g,4llx,4gfn,4ghp,4w97,4xuh,4qg8,4ck1,4lcq,4iev,4j02,4uug,4k5o,4rak,4ops
,4id5,4x14,4fft,4opc,4d33,4mr6,4pgt,4iaz,4phv,4ejd,4yyt,4y1p,4jye,4ojo,4ki
o,4ybk,4jwy,4k6i,4q42,4cpf,4khr,4udd,4xrg,4rv9,4p53,4q7v,4d5q,4jso,4i6h,4i
jr,4avl,4qnw,4y85,4koa,4xym,4r4y,4ys0,4alw,4i89,4nkw,4x7g,4qdi,4u6z,4orr,4
jva,4p87,4iwc,4zy0,4ugq,4mx0,4b7e,4qyy,4w88,4hjz,4ktr,4g0q,4ct1,4gae,4eer,

4amx,4k1b,4jiz,4rr8,4azq,4fr3,4wx0,4bqp,4mpo,4lww,4oas,4chq,4pa0,4g2i,4pul
,4zcw,4pmz,4k81,4wt6,4j0s,4ndr,4lxj,4x6p,4w93,4q1d,4nrb,4opg,4mk8,4x1z,4at
4,4ivg,4h0y,4moc,4lvt,4qg2,4ig3,4cwr,4u54,4aiu,4cft,4du7,4x7q,4e2v,4dqu,4n
x6,4ybs,4r6n,4edf,4gj8,4tqa,4fmh,4a61,4jem,4qx4,4wyc,4rfr,4ipm,4n4l,4d36,4
obg,4i5i,4bw2,4rxs,4bgm,4h2b,4bsb,4due,4a6w,4j74,4ms4,4zk6,4egn,4ihl,4bc2,
4bds,4qlf,4ms0,4rlk,4dwv,4gtr,4f1t,4jto,4ymz,4dff,4qmx,4d79,4gzt,4zyu,4ixe
,4cc6,4k2g,4ob6,4q39,4p7n,4cb6,4cw2,4g1z,4hev,4f09,4q3u,4gp9,4g7a,4kcu,4ar
b,4k1j,4xsr,4djo,4nzo,4wyz,4m2k,4e5l,4y2h,4rrl,4jsm,4xqb,4gsy,4yw3,4h3b,4f
k7,4fhc,4gj3,4ea0,4jsi,4hws,4yth,4o0r,4e1g,4q4d,4bz6,4mrq,4qij,4bmp,4u6d,4
baq,4cbx,4f8p,4b7p,4znh,4jyf,4hrq,4xt8,4epm,4moe,4f7e,4wn5,4ie6,4lpg,4hsj,
4o97,4gr8,4lnw,4hwz,4wcu,4kqi,4xfm,4ztd,4bti,4ijw,4k5m,4wub,4gab,4his,4aj3
,4e03,4kdo,4txc,4obk,4k4e,4o7b,4kwf,4y0k,4dhu,4ide,4xgu,4lz5,4qos,4eu7,4ep
r,4xz9,4nvq,4yo8,4owq,4r75,4ggl,4xu6,4aua,4ai9,4o59,4f65,4oa7,4wor,4qga,4o
be,4uvx,4wnv,4pce,4ibc,4bre,4olh,4ih6,4od9,4j14,4coe,4i94,4bs4,4n9a,4d19,4
y9l,4cgg,4afj,4ier,4bzo,4psy,4i85,4aq4,4tyl,4zwi,4dij,4kne,4zlu,4nao,4cfa,
4qxm,4xx4,4wn0,4man,4yz5,4hwp,4r9a,4yef,4yg6,4l32,4d9p,4zob,4af0,4bra,4laz
,4cu0,4bu9,4z6u,4l0s,4wkp,4j56,4v0r,4jfv,4i1c,4w55,4hn4,4jfj,4ojl,4tqk,4cm
9,4x3t,4wuq,4otq,4bjx,4yj1,4b4q,4cwv,4mpe,4o6m,4m3f,4o3c,4zpf,4v03,4idd,4u
gl,4ctx,4ok5,4kcv,4wnb,4r2m,4itm,4zw6,4a2b,4wzv,4d57,4gtc,4j7h,4pkv,4pte,4
ma4,4gd9,4qo8,4la6,4poj,4eqg,4jmz,4ibe,4umy,4agc,4hb0,4m8d,4xb2,4kfp,4o55,
4d00,4o24,4ohv,4yzn,4o3f,4ge2,4j9z,4hwr,4tmt,4dvn,4zax,4cfd,4pyw,4ivn,4k78
,4fri,4ymu,4x4a,4cjl,4kz6,4e4m,4a80,4zwy,4do3,4hmn,4c5k,4lwm,4yt6,4znq,4yp
e,4fr8,4wns,4cpt,4dbs,4puk,4bfu,4kmw,4tv1,4nm3,4jma,4v2o,4xda,4jdr,4dsh,4d
pt,4q3e,4mmm,4gt9,4zun,4iu5,4kwk,4cvn,4jss,4uso,4ccb,4na4,4ehy,4leq,4owk,4
hju,4qsi,4ifw,4nju,4rll,4g45,4uh8,4bb4,4yl1,4pl8,4ca6,4lz8,4mut,4qt2,4hvh,
4dm6,4mlm,4quo,4gcp,4g33,4cpy,4fia,4upq,4hx5,4bao,4ctu,4i0s,4gql,4qjr,4i4f
,4nfd,4aoo,4em4,4ojm,4gmw,4clj,4w9h,4iss,4v2y,4lau,4kxl,4v28,4km0,4moi,4k5
z,4wts,4o7f,4mqp,4eet,4u1l,4hg7,4w5j,4zfl,4mg5,4kcq,4pfj,4hzp,4afk,4nbb,4f
9m,4ehh,4x6f,4iks,4iie,4uh9,4de2,4y9f,4b2x,4at3,4v0q,4p8o,4kvw,4h0t,4dvi,4
cyn,4m2u,4kri,4r91,4h2u,4xw4,4lxi,4y8v,4xz6,4x7j,4yuv,4ze3,4pxl,4kcr,4nxr,
4oq3,4b6e,4gmy,4yuy,4m3g,4itj,4xti,4o2f,4y14,4lwc,4mwo,4az0,4nyf,4o0l,4cb5
,4mgc,4z6t,4a7x,4jao,4g2h,4dgn,4j0r,4uuw,4rw5,4u61,4x10,4nes,4cwt,4eyr,4d8
6,4cs4,4v3i,4riu,4iw2,4kyh,4d2y,4fi8,4ktk,4mfk,4r73,4qd6,4i9n,4c6n,4bri,4x
by,4gda,4lfl,4non,4lgu,4q2v,4o0y,4kfo,4ply,4rmd,4nue,4tx0,4fc7,4edy,4cu1,4
hap,4gw8,4zjz,4ixv,4m69,4l7f,4ahu,4f9v,4za5,4bfm,4hym,4ek7,4bcq,4r3w,4a2a,
4gjy,4egh,4k1k,4dmw,4qo9,4rd4,4x3k,4p7q,4ugy,4btu,4kcw,4x1f,4lu3,4jwr,4lbv
,4ryd,4k85,4cga,4q18,4u8m,4x3u,4zp0,4nx4,4hee,4qmq,4jk3,4qms,4x0c,4d5j,4z6
z,4asd,4cae,4km2,4b1d,4ohb,4ctb,4rp9,4el0,4igq,4cf2,4qfc,4djy,4k1i,4imx,4d
ai,4q40,4x2j,4nx7,4qkn,4x7h,4cfn,4ais,4b76,4p5e,4jjg,4h03,4czl,4eeh,4usi,4
eay,4kz9,4xck,4d31,4p6g,4ib1,4cr7,4tq3,4ceo,4m1w,4zmf,4ugr,4mob,4jia,4ejh,
4e3q,4om8,4rdk,4z82,4kqj,4cyd,4uxu,4nbh,4md6,4zb8,4a7i,4btb,4i7n,4do4,4ne2
,4tl9,4bq3,4zjs,4eym,4z2w,4m6v,4a1o,4au0,4yz9,4znt,4ehl,4wpn,4lkm,4x6r,4al
h,4wf4,4bw4,4gjq,4f99,4r9w,4jm8,4d3b,4q6n,4rh9,4p6w,4ajl,4wup,4p31,456c,4w
9l,4jpt,4hxm,4njh,4fe3,4o15,4rr6,4zur,4nzf,4f96,4nxc,4rh0,4mdh,4zfi,4xia,4
c81,4wze,4mqr,4u7f,4ot6,4cjs,4rt0,4f1m,4lxx,4okk,4cmb,4yju,4u9v,4j93,4dkx,
4lh5,4fw0,4wh9,4rel,4m44,4x15,4lp9,4dfg,4frm,4u2t,4rd2,4r1v,4yta,4lph,4y73
,4hxp,4b4b,4n84,4e93,4kip,4b68,4gxr,4kew,4cx5,4psq,4c6m,4a23,4b78,4ctr,4qy
f,4fhd,4l0b,4m37,4ui4,4nae,4uox,4cpw,4q3d,4m72,4bcn,4i2d,4z1v,4hwt,4zx4,4w
s8,4gv1,4p68,4tv8,4m12,4lvb,4ocv,4k5r,4jqm,4r0i,4rse,4bkz,4mic,4ydq,4upo,4
fyo,4e5k,4k21,4msa,4uyn,4u0v,4np2,4qjx,4yjt,4rek,4ugu,4kjv,4a85,4nu1,4x60,
4wxg,4cwn,4mg7,4p8y,4j6d,4gnc,4g44,4maf,4jn2,4lhw,4oiv,4lzj,4c71,4rpm,4mlt
,4d88,4eoy,4j1f,4j98,4mos,4ekl,4m5m,4urn,4rz8,4b1f,4wna,4d1z,4k49,4z6r,4wy
f,4p26,4eks,4gkv,4bky,4er1,4zi3,4xpi,4qxq,4d5p,4hla,4b9b,4h6u,4tq9,4y86,4r
6r,4m13,4n32,4k5i,4b7s,4fe9,4ep9,4q3q,4v3y,4nfh,4eue,4i4z,4pku,4dn0,4dph,4

o13,4cd4,4hmu,4y2p,4hz0,4xhd,4pme,4i3v,4eji,4ac2,4c13,4nt6,4fmx,4j09,4j47,
4j08,4ygf,4y59,4mlx,4nba,4ydu,4pf3,4m82,4i8w,4wxb,4dk4,4gky,4d4u,4enh,4mol
,4gtl,4ilx,4jsa,4gox,4ctt,4ktj,4wvs,4cl8,4jsc,4u43,4n9q,4mfz,4iw6,4xu2,4ib
3,4ncn,4wdb,4dbm,4ugs,4o0x,4m6y,4bq5,4mrs,4r57,4iq9,4yz4,4xvx,4w9f,4mhs,4g
m4,4zy2,4tz8,4ejn,4cr6,4eu3,4j24,4wlk,4b9e,4lj6,4wck,4azp,4rx8,4cjk,4abv,4
gvq,4oya,4xj3,4huv,4j0t,4uyw,4e2j,4lc5,4cjn,4jjs,4ptn,4k30,4hrw,4ojr,4xxr,
4rz3,4uec,4ibk,4xli,4lek,4gzx,4hk8,4cgl,4dfx,4n39,4n8w,4a86,4al0,4wh2,4gyo
,4zg7,4rnw,4hy1,4jym,4v0k,4ob0,4j3l,4xoq,4r29,4l49,4hj6,4wvf,4n2z,4lag,4ru
s,4fh6,4kju,4bj0,4pbs,4jkw,4zw7,4fmj,4hux,4qwp,4ynk,4kyq,4dxa,4gyi,4lyh,4p
n2,4re8,4yoi,4d5e,4uhn,4q90,4rje,4yaf,4k90,4m52,4cj4,4qsj,4ec0,4lus,4ara,4
crd,4qf9,4lj9,4j25,4ehd,4zuq,4ori,4iid,4owu,4a81,4d72,4ech,4d32,4s23,4o05,
4fer,4zaf,4hpj,4ufj,4fph,4oxy,4dhn,4zbf,4pc6,4eqe,4u7v,4zy5,4ptj,4i9b,4wxh
,4i6s,4p0w,4n7j,4dhs,4cww,4att,4r6o,4eh8,4ipf,4rnv,4qed,4gix,4dae,4i01,4ha
w,4urz,4gni,4b5b,4ffi,4bcd,4x81,4z3m,4nmq,4ww9,4yv5,4j4v,4tte,4a79,4iv4,4d
dr,4yav,4d39,4jv9,4az5,4a91,4kvx,4b5s,4zo3,4mt9,4i4t,4lbz,4j6o,4bz8,4s00,4
lxy,4lps,4fbx,4inw,4zgk,4u0f,4kv8,4v32,4bnt,4lwb,4bdn,4uga,4lw1,4rcf,4k9h,
4iok,4jsk,4jir,4jh6,4q4r,4xiq,4b64,4a3r,4nbl,4p9v,4dja,4ck2,4y5d,4lge,4gfo
,4ou4,4b82,4mg6,4jvf,4flp,4bo1,4pnf,4c72,4ego,4hvc,4l9s,4bi7,4r54,4j2v,4rw
g,4e4q,4kp6,4bcw,4wzl,4di2,4hzx,4ib0,4x11,4kjj,4fxr,4ko8,4z2k,4h5d,4na0,4h
zf,4jzf,4l7i,4k5l,4cx4,4ojp,4ia1,4aq0,4q71,4wp7,4tnz,4oca,4umf,4daf,4kb9,4
yak,4i5p,4qxi,4xm6,4y3b,4wz8,4jii,4a6f,4ufz,4riv,4brd,4ymb,4z2t,4fpk,4mep,
4q0u,4n4j,4eu8,4ig1,4ks1,4p1v,4rte,4d3h,4d5i,4qor,4yh2,4jsf,4dkp,4bb5,4wri
,4o9v,4old,4ktp,4gk9,4nj3,4gvj,4q19,4dv9,4lfm,4d08,4qyh,4z1e,4caf,4l4l,4ou
j,4qj2,4ii5,4z83,4eyl,4oz1,4hdk,4g46,4cj6,4wzy,4bjz,4gxs,4rcz,4k5s,4tyv,4e
qc,4iey,4wq6,4cyi,4z1j,4d0t,4cqd,4pge,4cjq,4k4r,4yv2,4wrb,4hbm,4zae,4lw3,4
abu,4anw,4yw8,4ivc,4ax7,4b2i,4zly,4j6b,4e2x,4gv2,4s27,4f2o,4k7o,4w89,4kzq,
4p8m,4l0v,4woh,4p7r,4kl9,4wms,4onc,4m5g,4fhp,4mrt,4dee,4mln,4ymq,4itl,4m3m
,4mmd,4eeu,4rx9,4efu,4jic,4zsj,4xsu,4xkg,4gvy,4q1c,4d7n,4dr9,4y4l,4oim,4cr
b,4em3,4rxr,4r51,4zls,4ug9,4ydd,4cdt,4zup,4bu4,4ykf,4bas,4l6t,4d30,4ips,4o
g7,4frp,4d6z,4dxg,4cx0,4apz,4qtb,4acm,4qte,4msc,4yjp,4ctq,4co2,4k4p,4n5g,4
cu8,4dw6,4anp,4eop,4glj,4m14,4xz3,4u8l,4klv,4tzk,4tvg,4g5h,4a38,4a6l,4mx5,
4xy8,4k5n,4gc1,4zhz,4tqn,4ow0,4psr,4qag,4aos,4brq,4dq2,4mss,4nec,4ozj,4n97
,4eoq,4sga,4qp8,4ez7,4p33,4iwf,4u8v,4oar,4msn,4dub,4luc,4wda,4m8e,4p3y,4xr
q,4tnd,4d1s,4xtd,4mns,4ui3,4ki0,4ef8,4hnb,4lo2,4x4l,4jxs,4xt5,4mwp,4rkk,4r
l4,4yxr,4b4m,4yc7,4drh,4dpi,4fg2,4xwt,4x7z,4ha9,4bke,4o95,4nvp,4ag9,4ql8,4
e91,4yaw,4hez,4bqz,4k3k,4qlg,4o7c,4ugf,4j0k,4mwy,4yvg,4hjs,4rg3,4fht,4mjr,
4u5i,4dd8,4jh7,4c3a,4qja,4j1k,4kwd,4i54,4umj,4jse,4oc7,4to4,4cpn,4udg,4lm3
,4d3t,4e96,4p8c,4txi,4lz3,4yz2,4dlk,4tvd,4wua,4y9t,4dhp,4d63,4jqe,4br7,4r4
3,4ly9,4k10,4efh,4qml,4zip,4j0q,4kwh,4anq,4u5u,4ykg,4uvr,4xmf,4oso,4uvz,4d
9c,4ipe,4ptg,4q1x,4elg,4ud9,4hxq,4jxw,4co3,4lcn,4yfb,4xr8,4ckj,4lb4,4o4d,4
ls7,4m7f,4cqm,4xxg,4i10,4rfy,4r9f,4fvr,4yoz,4q38,4oqd,4ke1,4egc,4rn0,4m7t,
4a05,4pg1,4myq,4hxl,4gde,4ayq,4qmu,4otr,4d7b,4d38,4y94,4jvq,4l31,4iaw,4foc
,4j06,4njd,4o1f,4cre,4nqx,4g10,4pik,4okm,4ckl,4mkf,4r98,4rg5,4gal,4j96,4e4
a,4qgi,4ctm,4ovi,4e67,4nsq,4oef,4b6s,4bdi,4rl7,4h2t,4j51,4c67,4q36,4j8o,4i
12,4krg,4ddn,4h8i,4clr,4pkr,4lm1,4af5,4i7e,4onf,4pps,4ey2,4j1i,4kax,4wk9,4
xwy,4eph,4yx9,4c7t,4frh,4irx,4csd,4dve,4wo4,4z3x,4bcm,4dei,4twc,4eu5,4zow,
4zsi,4uwh,4c6q,4fcr,4mpc,4c7i,4jlk,4bft,4mr5,4l5z,4jal,4mwv,4l7o,4gll,4rn2
,4dpe,4dti,4upd,4uvu,4zyf,4rme,4l40,4r78,4pjk,4xua,4bz5,4poh,4iqu,4m3p,4hd
q,4je7,4ak7,4u73,4q7s,4pd6,4loy,4hj2,4lsy,4yfy,4m6q,4pfw,4w6z,4ajj,4q6g,4a
nm,4g95,4p1r,4gkm,4zab,4nf6,4qe1,4e3f,4j8m,4br5,4cmj,4ktf,4wa9,4xv4,4ume,4
bks,4gnl,4ylf,4xd0,4cqu,4eht,4gqp,4y6n,4ctn,4u1n,4c36,4ob1,4cqx,4q87,4zme,
4icy,4jbv,4hoz,4ep2,4lm0,4ma8,4p05,4jsz,4u2l,4rzm,4g55,4qr3,4rrc,4ix5,4l80
,4w9d,4fdc,4ty7,4ek9,4h1e,4mkb,4s3b,4g32,4gxd,4g86,4o4k,4hpd,4ywu,4pwh,4nm
b,4x6n,4ly0,4nbe,4ovl,4cr9,4g6g,4z3d,4ajx,4kpx,4isk,4zb2,4omj,4i22,4qhp,4t

sk,4jne,4mzu,4h7c,4x9m,4jlm,4uzi,4tmx,4dw3,4cm8,4dyg,4ruy,4pfu,4dpd,4ug6,4
uin,4fgh,4nma,4y4g,4u1m,4ghh,4but,4z3w,4o1p,4feh,4auj,4zs9,4n4e,4x5e,4xfr,
4men,4k4b,4x2v,4l4a,4j1p,4ele,4q0v,4bj8,4bmz,4jsg,4ht2,4hld,4er4,4idt,4tu5
,4rcv,4z7x,4qxs,4m0e,4r06,4ybm,4e4n,4opd,4kp8,4c70,4cd2,4drq,4bcr,4ee0,4r9
j,4xew,4a4l,4xfg,4gy9,4pjj,4fvq,4qeh,4njk,4y0p,4n66,4rl2,4epx,4s39,4y6o,4l
kl,4xf3,4bcs,4ea1,4dre,4mdn,4mrg,4y8d,4u0n,4amj,4pks,4zwb,4xg6,4z4r,4x3j,4
igk,4rvl,4wcn,4z2r,4azi,4i4v,4o2c,4qmp,4pf0,4j28,4cku,4bek,4q86,4pq7,4aa0,
4mvy,4iew,4uuu,4uoz,4xkx,4hph,4eqw,4yw9,4hr0,4z94,4ogw,4pin,4qjo,4bqg,4faj
,4ruk,4k4j,4o0b,4bsh,4bqw,4col,4j5h,4lcg,4zwg,4mw2,4dhm,4x8n,4hmx,4yqe,4ko
x,4aje,4uy7,4feq,4jie,4mdb,4r8s,4cl6,4qnr,4wg0,4wnp,4gk3,4mcp,4xh1,4cta,4d
0z,4yvh,4jxv,4do6,4hww,4rd3,4xy2,4mk9,4haq,4mi3,4bo3,4zgr,4lbj,4g43,4lhm,4
ejl,4xp2,4r3c,4n8r,4pyx,4cam,4jna,4z2l,4iaf,4fvy,4s1d,4buu,4q0a,4fdn,4l4c,
4tq8,4nan,4d17,4zz3,4frb,4s26,4gte,4kfu,4d5b,4ux9,4p6c,4knn,4m26,4ye3,4nz2
,4wpa,4bfz,4uns,4rdl,4yc0,4o2l,4uqh,4klx,4iig,4v30,4x7y,4bf2,4m20,4rks,4dl
c,4hey,4kup,4z4u,4ztc,4ttq,4prv,4euo,4e1o,4ixw,4wiv,4ctj,4ze6,4zwp,4uiv,4c
qy,4fm6,4iet,4mw0,4ox5,4emi,4qta,4tu4,4qx8,4czg,4zyx,4nev,4d62,4ynl,4wmx,4
m56,4kn3,4gaa,4x5d,4n07,4eqk,4eax,4j3i,4mw4,4clx,4ys9,4i7j,4qmt,4y0x,4eua,
4yne,4rxd,4ft8,4cbu,4x1g,4mq1,4drv,4za4,4nz4,4knx,4zfz,4j26,4ure,4tvh,4mjo
,4nbj,4cdn,4e0r,4h2w,4d3m,4c4d,4crz,4dbv,4xb9,4l9i,4jfu,4f8l,4l8q,4lvf,4ix
u,4fwp,4xr4,4nwz,4z2u,4xyb,4q73,4a87,4ifz,4nah,4l5m,4r9g,4ge6,4us9,4xpp,4j
yv,4ufm,4zzy,4clk,4ucp,4f31,4zdc,4s3f,4i60,4y9d,4cfk,4rpo,4cce,4car,4mgd,4
mwq,4q8x,4fch,4w6e,4r93,4f0b,4lhh,4cjv,4e6q,4loi,4q3t,4b1u,4bhf,4wuy,4bku,
4qru,4axe,4nmp,4lh6,4za0,4mjp,4anv,4fa2,4yxs,4f1y,4f1l,4joe,4zol,4ag3,4xsp
,4zjw,4y2s,4hiq,4brx,4uac,4l5o,4mqf,4zi2,4dch,4i5m,4gqg,4tvj,4rra,4itu,4pp
5,4wrz,4li6,4xcj,4jp4,4rii,4y72,4ltw,4u60,4ucg,4p44,4avk,4j6c,4q3f,4aji,4c
v0,4isa,4bo2,4x6e,4xq9,4xtw,4izd,4uge,4k33,4i39,4jst,4asa,4n8q,4gj5,4g8n,4
nce,4uoy,4qub,4wyu,4n4t,4wvo,4clp,4u7q,4hon,4l3j,4owa,4yzu,4a9u,4yll,4ucc,
4u6q,4tyt,4oc2,4qtu,4lxa,4o3b,4dvt,4i02,4ckq,4bva,4o1m,4nw5,4kwl,4x2i,4la7
,4d2z,4b6c,4mkt,4ot5,4alx,4lm7,4ojf,4qu9,4gcq,4ug7,4hnc,4iic,4lwv,4kxn,4lr
b,4wb6,4oz2,4y3j,4m38,4cvb,4hmw,4bu8,4por,4yjq,4kou,4h2z,4bn1,4s1k,4h2g,4m
su,4zqm,4ot2,4m7u,4do1,4k80,4ez3,4j0a,4mo5,4ig0,4w1x,4z8d,4tr1,4ddy,4n98,4
eub,4g87,4zz0,4joo,4us3,4ezd,4eux,4jvn,4uhg,4epl,4tsm,4y32,4b11,4jgt,4gpb,
4k1y,4u8n,4mbg,4bfn,4pl7,4q0p,4ezw,4ke5,4g9p,4ite,4kck,4qn7,4a22,4ut4,4hsu
,4b7k,4cyo,4zyv,4zfy,4bnv,4luz,4bby,4rrd,4cma,4mqu,4ibd,4al4,4usr,4mg8,4l0
i,4l2h,4nzm,4u1z,4py1,4c5n,4ibb,4f6z,4nbd,4oc0,4cfv,4zsr,4fpy,4msh,4c6u,4d
56,4z93,4dux,4wvu,4gjv,4wov,4lok,4iir,4plw,4agm,4ep7,4bcp,4eh5,4xnc,4lxm,4
gii,4tr0,4c7h,4kb8,4mnp,4cxq,4o4r,4ehn,4l8m,4k3o,4l0t,4gzq,4det,4u31,4j3e,
4b1x,4j1c,4wq5,4tmb,4lb3,4wdg,4yx4,4q06,4qxr,4dea,4pyo,4hyw,4mae,4mv3,4ouc
,4clt,4lpk,4ejg,4dt8,4g0s,4qfz,4ac7,4yua,4kzl,4m5p,4abq,4d7e,4jb1,4gw0,4nw
j,4eo8,4fhg,4uaa,4crg,4l8d,4pu0,4v1t,4ctk,4jny,4g42,4e0x,4oev,4kux,4den,4p
ct,4irp,4bzr,4ugg,4er2,4bv3,4gqi,4gna,4nmr,4m0r,4j0y,4p8l,4rsk,4uhi,4n9v,4
s13,4kyc,4qrh,4rsz,4ear,4b3u,4rjd,4rcy,4ini,4xbw,4bgk,4tlr,4m54,4nto,4c12,
4d7i,4g74,4cie,4ws1,4c43,4i7l,4fwe,4jn4,4ugw,4lz0,4cgi,4av0,4iad,4w7t,4rhx
,4p8k,4r92,4ue7,4fgg,4l2k,4y2b,4kh6,4ldj,4dsn,4ij4,4u0w,4mp7,4umx,4mus,4pz
v,4bme,4mys,4isi,4oco,4k7f,4bc9,4cjp,4qjp,4gtf,4rut,4oee,4g2r,4a6h,4zbq,4h
2i,4lux,4rp8,4zt6,4r5a,4iu1,4a30,4cef,4rhs,4mok,4egi,4up3,4x50,4ofg,4j36,4
zex,4bny,4avo,4cg9,4ao5,4a9i,4iee,4cmc,4fpf,4xhv,4q6o,4b6r,4bt9,4xqc,4at2,
4blc,4bpm,4pu5,4qul,4lh2,4cfc,4zu7,4r8h,4i6g,4plf,4dvw,4d2c,4xxh,4ifc,4kn1
,4pj3,4l8u,4ehv,4jsj,4qdf,4afg,4gtb,4mp6,4hzw,4a3w,4qj7,4i74,4omc,4x1t,4co
d,4ju6,4fl0,4yog,4ghd,4em9,4m2f,4jd5,4mga,4jvm,4rri,4frj,4kby,4pmt,4x2u,4q
sa,4uh7,4oxn,4y30,4bu5,4lnp,4gue,4q9y,4gyy,4wsy,4p4t,4f5b,4bxf,4dgr,4cfu,4
jyc,4kz4,4pow,4ugm,4c8i,4ith,4lcr,4ytc,4ixg,4zoc,4fs3,4e1n,4pc8,4s0g,4i6w,
4i55,4h3q,4ey7,4j86,4ruz,4uwd,4a9e,4q99,4kqr,4z1q,4qoc,4j0m,4cv2,4q4e,4qir
,4zvv,4oq6,4en4,4s38,4bnu,4m22,4dab,4m2w,4bck,4l6z,4wvw,4zt5,4g90,4yau,4dm

n,4h3p,4bqr,4f7t,4o28,4hzo,4ub7,4x05,4eng,4ful,4f7p,4icc,4z55,4qxv,4ui6,4g
47,4ds0,4iu7,4i7m,4fhi,4jt9,4rvg,4bqx,4jpc,4ws2,4gc4,4c2p,4jwl,4rj3,4n93,4
wop,4b73,4urf,4euu,4xen,4f2w,4y3y,4uxl,4lmb,4wx2,4u0e,4ezr,4hnf,4kvd,4g05,
4nwm,4qnb,4cfw,4kow,4tts,4uw4,4ura,4kqq,4own,4ea2,4qc2,4lrg,4dz1,4yhk,4agi
,4cwq,4r3n,4xo8,4z4t,4lv9,4zxt,4de0,4j54,4nos,4oyx,4l06,4yds,4qe3,4djq,4jf
l,4o33,4p9z,4m3e,4yab,4ibm,4drn,4qna,4j3m,4rdh,4wmv,4lr8,4mig,4xt4,4yt1,4h
bv,4pit,4ijl,4m5u,4lht,4ikt,4tyj,4yw5,4qx5,4ugx,4zlf,4cwx,4r41,4nz6,4ng5,4
asj,4gqr,4ey3,4pam,4mib,4xhg,4clz,4s25,4lx9,4qj0,4c1u,4bah,4uuj,4m84,4dcm,
4jff,4yki,4c91,4ahr,4fvx,4lah,4f3k,4bjy,4evs,4f0e,4c7f,4tnb,4up4,4y4j,4aoi
,4au8,4zx1,4wdu,4hv8,4m5s,4tuy,4ks7,4ciw,4qpd,4r84,4zed,4lop,4ze0,4lpb,4r0
a,4u93,4idn,4kxb,4zy1,4epq,4ku7,4q56,4kdn,4mv8,4g8v,4xhe,4y9c,4awh,4me6,4z
ek,4dv8,4wr5,4lzr,4xne,4lxb,4luv,4d35,4b7x,4zo7,4bii,4fbv,4fyw,4ua3,4fal,4
tmk,4imt,4zi4,4j7u,4j53,4mcd,4zsk,4i7r,4dht,4b72,4u8k,4jx9,4lwt,4zyi,4qy3,
4hyi,4xyf,4ugz,4c2z,4i53,4k3n,4x49,4jnk,4jfd,4uqo,4lo6,4fyv,4v36,4gv4,4gg5
,4e5p,4n5n,4jlj,4xp7,4jt8,4go7,4tqg,4osp,4oh4,4wqq,4pk3,4zm3,4lng,4rhp,4q8
y,4kvy,4x2f,4kh0,4i91,4cki,4l6s,4x8e,4e33,4j0z,4qye,4kif,4bc4,4uz6,4m6p,4a
9m,4ld2,4qsb,4fgz,4fls,4jfk,4w9p,4c74,4zbd,4bco,4cje,4i4e,4uiw,4oh5,4rux,4
lit,4hmt,4zs4,4h0i,4djv,4xj6,4otk,4bol,4ajk,4pwj,4b6p,4eyj,4k5g,4iu4,4x2k,
4f8o,4ptv,4ms1,4wy3,4fp2,4tqh,4arw,4ymj,4b70,4mlp,4w53,4m4k,4bdr,4d75,4rm3
,4d3s,4qrs,4dta,4bjb,4mpd,4u9w,4jxc,4o9g,4xrz,4el5,4c9l,4jv7,4mro,4rcp,4hp
i,4plz,4occ,4rvk,4o8p,4bw3,4nwk,4zvz,4cd7,4cuc,4ggz,4tz2,4fdv,4fra,4d0w,4b
fp,4jwj,4xrb,4mmc,4bi6,4avh,4quj,4auy,4fdo,4ydx,4ms3,4uyf,4l7s,4uvl,4yl2,4
l9y,4r8y,4iac,4mgj,4bwa,4bnz,4f10,4zib,4gsv,4qo4,4co1,4a39,4g2w,4rc7,4l3l,
4h3j,4bvh,4e5f,4jt2,4pkw,4u63,4cmo,4rd0,4cxr,4q4p,4wwo,4urx,4gta,4qec,4awq
,4wko,4d96,4d3v,4u2a,4cd6,4li4,4n16,4uyh,4qoq,4la0,4z06,4okd,4y9r,4znu,4m7
1,4kv1,4kvr,4pxe,4l1w,4oc4,4i4w,4rvd,4gbd,4zbb,4gm0,4yvz,4dxj,4akn,4hxr,4q
om,4h2s,4wq2,4mc3,4c34,4b83,4qmw,4joc,4flk,4erf,4o2e,4ruj,4f9b,4xk9,4oqe,4
x7l,4r5v,4ocx,4fro,4jm5,4c5m,4h2x,4cer,4h8n,4e49,4zum,4hy5,4kcl,4l9z,4cpu,
4p4e,4pv8,4lrj,4pfy,4xw6,4ea7,4csv,4el9,4ihq,4xaq,4fyx,4ew2,4ceg,4d3i,4ox2
,4obf,4hbx,4f7w,4guu,4ctp,4ri6,4b3b,4h9x,4at5,4ia0,4doo,4cto,4tz5,4xo6,4zu
4,4bow,4ccn,4y67,4iak,4fam,4hv3,4pfk,4c0w,4p66,4pjl,4jej,4r6w,4hzd,4acu,4r
pn,4ju3,4zul,4ktc,4q6e,4deh,4zea,4z0h,4fgx,4kgv,4btk,4kvs,4g48,4hs3,4lfv,4
cp7,4gyk,4wdi,4n7u,4xps,4hej,4fyn,4ujb,4tnx,4djx,4bqo,4ce2,4e8c,4zrn,4mwh,
4rm0,4b42,4k5j,4rle,4imu,4do5,4hvs,4qop,4fbl,4isz,4ufb,4u3d,4w59,4jvl,4ng2
,4dls,4tv9,4g8z,4pfp,4eqr,4qjk,4du2,4ob2,4uhf,4rf2,4b2k,4pya,4rt1,4c7b,4zq
p,4q3a,4h6r,4go2,4etz,4wvj,4rpl,4fvt,4coq,4dn8,4l2f,4ufu,4l9p,4oct,4jvb,4o
ym,4cv3,4uvy,4w5i,4ok3,4cmg,4kcx,4h9g,4x5b,4lj8,4c2f,4ogi,4og4,4mkc,4nks,4
cr8,4xgx,4n5v,4n3b,4frq,4uap,4evi,4o7a,4cq0,4xqa,4awk,4rmn,4der,4xmo,4g6h,
4h4e,4pw9,4rx5,4mv1,4dvs,4uha,4bbe,4hb4,4xyl,4u1o,4hdc,4x28,4qua,4m5l,4uiu
,4uhm,4dx5,4j8t,4cle,4rab,4rc5,4q3r,4pwk,43ca,4bae,4yjo,4znw,4icl,4poo,4nr
c,4qiy,4htx,4iay,4rrt,4cf0,4x7r,4hfp,4yw6,4rfz,4lh1,4p6p,4j7n,4mzh,4kft,4h
2f,4f9a,4jin,4x3i,4jqg,4x36,4c9p,4lcf,4ef4,4mju,4rkx,4o5g,4cu7,4zag,4fi7,4
x07,4o9s,4kar,4xu0,4o16,4jbz,4aa4,4phs,4qyg,4hmd,4eor,4r9b,4j5y,4kjg,4ie9,
4g9e,4uja,4qzu,4jp9,4px6,4e32,4qa0,4n02,4j17,4ty6,4ysx,4nus,4d8a,4jh3,4j4h
,4gps,4xlv,4ojb,4q30,4x48,4n1f,4a06,4ajc,4kvq,4gh8,4xoz,4lxt,4ylz,4xp0,4cw
p,4cp8,4jiq,4l78,4eoh,4wmr,4xuq,4h5t,4rdi,4u5x,4qa8,4uc9,4kas,4eip,4qpw,4d
fu,4yax,4z40,4m8h,4p4f,4dhl,4j77,4i42,4jz9,4obw,4iv9,4mrz,4pzw,4jql,4hew,4
orm,4xip,4dly,4ewn,4b5d,4hy4,4ztl,4ugb,4qfx,4ibf,4dg0,4qtl,4hyv,4o72,4udj,
4zdj,4kjl,4y6p,4q4q,4wkn,4jqn,4kvl,4puz,4lre,4yal,4moh,4jnz,4c6l,4uwj,4k5u
,4zod,4q6r,4yp5,4ur9,4a01,4ufg,4dtw,4zew,4xw5,4g8b,4fyy,4m5k,4zu5,4xt0,4ah
9,4d4g,4i09,4li7,4g9l,4lh7,4buq,4zvl,4a7b,4csc,4wbk,4pvp,4jzx,4wkh,4g1c,4c
2g,4na9,4wla,4ou3,4tmc,4d97,4zo6,4oag,4mr9,4q8z,4z4y,4xy9,4ahs,4c9x,4wf8,4
qa2,4jh4,4hxs,4k5k,4meo,4imw,4zwz,4h53,4a31,4rn1,4cm1,4zsa,4pcs,4kqx,4mog,
4f3i,4g8i,4rlw,4bfo,4agn,4jve,4jnm,4ndo,4di9,4ww6,4nf4,4wi1,4bpr,4mv6,4tkb

,4ibj,4zpe,4ooe,4iu0,4cqw,4aac,4hjv,4q0q,4uho,4f39,4dbw,4jwh,4fok,4zo5,4xw
r,4edv,4zke,4dds,4m2c,4ncl,4rnq,4x35,4o6n,4itb,4r2n,4e92,4r9d,4z1n,4e7v,4z
nv,4xtz,4x6y,4ej8,4ddh,4eil,4wet,4qrc,4s3q,4uw3,4o4h,4n69,4tqm,4b7h,4ua8,4
l4e,4emj,4rch,4ap3,4q1n,4xvy,4kct,4bwl,4g37,4w57,4gsu,4utw,4pr8,4pyh,4oal,
4zay,4jzd,4dqf,4u5j,4bhi,4dfr,4wvv,4q9n,4a6b,4knr,4kcg,4jrz,4frk,4c6w,4cm3
,4n87,4po2,4dfl,4rif,4kbn,4y2t,4oty,4f7v,4u21,4u2y,4nmf,4lrz,4ocj,4mo8,4d6
p,4yg0,4c01,4c37,4zdz,4r1s,4pyl,4h07,4wa2,4x0z,4r4j,4fx8,4h12,4a0s,4q1a,4w
4v,4p6x,4aj4,4key,4at0,4cty,4inb,4ym3,4d4v,4jay,4fz6,4ynt,4kbf,4lcw,4qgh,4
kmz,4b6o,4kai,4z2b,4rrb,4ghf,4l7c,4bh1,4jwf,4lrf,4z4s,4rvm,4ncy,4fre,4cgp,
4w9s,4i11,4ptx,4x47,4e6d,4mwb,4ijv,4xzj,4eh3,4xw2,4itz,4oke,4b9z,4cwf,4za7
,4cyp,4k5d,4gah,4glw,4ce5,4lyn,4p8n,4n34,4cli,4zkl,4ddk,4z4p,4tuz,4ohw,4ce
e,4hjy,4qc6,4lja,4xxc,4zc9,4gst,4cpz,4xvq,4y0s,4rhe,4za2,4kxy,4d13,4m2g,4k
il,4zbv,4c5l,4zx6,4z41,4lch,4zwu,4w9i,4f3g,4gk2,4mme,4ubv,4xcl,4p4d,4uog,4
foq,4yu7,4x12,4xoy,4d8n,4dko,4m6k,4cmk,4l2z,4lcs,4hr5,4dx7,4mv4,4r6p,4emv,
4ebv,4upn,4qj6,4y6g,4za8,4dyo,4dlz,4l8w,4k84,4mot,4ks4,4k2i,4pah,4g9f,4ksy
,4x9w,4ewo,4piz,4twp,4pzy,4rrh,4wk7,4jlh,4u1h,4pfc,4hyu,4twn,4j1e,4lvd,4dr
k,4l1t,4pc2,4mho,4q09,4rqu,4z78,4crl,4tjx,4ko0,4aox,4s01,4gu6,4a8t,4xdu,4w
ud,4x9v,4ejk,4r81,4wf5,4dwg,4d83,4ee7,4x7v,4br0,4gvu,4psb,4m25,4ajn,4q9o,4
ipw,4yeo,4x9n,4zaa,4c3x,4h1j,4ogv,4bt3,4pjm,4x0m,4p42,4bgb,4huu,4ly3,4bqy,
4qb8,4iv2,4yvx,4bc5,4b1w,4fju,4bie,4cz1,4lko,4pvv,4z2h,4e73,4wo0,4hlw,4bkj
,4ylg,4n5d,4c1k,4owl,4y38,4qtd,4yat,4quh,4jjz,4aah,4awo,4acs,4b98,4kbb,4xb
a,4dtk,4x7k,4d8z,4akj,4kbs,4gc2,4b65,4xt9,4tlc,4lyq,4jqj,4ijq,4okx,4gw2,4p
th,4e5w,4ozl,4qxc,4z6o,4xu5,4yjs,4mbc,4b3c,4bvm,4i6a,4bvb,4rss,4bzn,4re0,4
ni0,4o71,4jlv,4f6x,4c0c,4g17,4k5f,4c7w,4bx7,4aic,4iwd,4u0m,4dju,4ytz,4rdz,
4brn,4r2i,4l4z,4g2y,4bup,4tl8,4mub,4f0f,4eev,4xya,4upj,4pgc,4aa5,4z6s,4lyj
,4adl,4pb2,4evq,4op0,4asy,4mz7,4zsd,4edz,4uci,4q1y,4adc,4ag5,4da5,4ims,4qs
9,4nmd,4pfo,4aia,4d5o,4dk7,4azc,4hay,4j6x,4dol,4lbp,4g01,4knl,4rlq,4nbi,4a
7a,4lm4,4ajz,4xit,4m3b,4cp3,4jzr,4ihj,4ky2,4x5r,4i0t,4fl1,4usw,4hpx,4tjz,4
j22,4rxe,4i4x,4zvf,4zla,4rc8,4fyz,4yhq,4hmv,4nl1,4pan,4r5h,4wuc,4yw7,4og6,
4yyy,4caq,4wry,4xpq,4m4q,4b85,4m2r,4wlb,4axa,4rwj,4irl,4g0p,4nx2,4wnc,4bw9
,4rls,4u0i,4xta,4p45,4nhv,4yp6,4uag,4cqf,4d3o,4frd,4d9b,4bqj,4jf5,4usu,4zw
8,4fmu,4w9n,4nru,4j83,4url,4ubp,4ffh,4frz,4qge,4d4d,4qwn,4m5j,4uwi,4lxu,4i
xf,4xg3,4kvz,4w8b,4i4i,4kb7,4i3w,4dri,4ajs,4e13,4qn5,4j79,4l51,4eox,4b12,4
cqr,4u98,4zyw,4xg7,4wye,4ws5,4c73,4qoo,4kcj,4l8b,4dtb,4uit,4yai,4jbt,4mvh,
4c0x,4ms6,4r76,4qzs,4z0g,4wz6,4q1f,4fiv,4hxy,4ad4,4pmf,4u8o,4ntk,4b5u,4iil
,4x3h,4hge,4zb9,4uh6,4pxm,4fi6,4eon,4dso,4jl0,4rwl,4yml,4zee,4fhe,4gnq,4bt
j,4xdt,4h4d,4yz7,4m3d,4fow,4w9q,4zgm,4o07,4a6z,4f2p,4mq5,4tvc,4nv7,4ohp,4w
s4,4bu3,4p83,4m1s,4uj9,4ean,4dvx,4jrg,4qon,4xry,4fh2,4lzs,4pkg,4qgf,4p8e,4
gks,4kco,4y7v,4z9l,4i6y,4zzx,4qo7,4egk,4pp0,4aff,4k36,4qa4,4o09,4xb4,4rrr,
4wdx,4fpe,4e5n,4jb0,4dxd,4cet,4nwl,4l6g,4qys,4u71,4ks8,4mih,4n1g,4oc3,4jfw
,4i7t,4hav,4lva,4a6c,4meq,4hxn,4aax,4x3s,4ufe,4mix,4v0l,4a34,4d03,4b63,4gn
z,4yoj,4q9s,4r1y,4gwt,4p7e,4dsy,4fes,4rrj,4k4c,4xty,4cm7,4p5d,4e4k,4xj5,4y
2r,4lal,4oth,4b2l,4jsh,4h0v,4o9c,4xh5,4uh1,4avv,4rsc,4to6,4exo,4q4c,4qdc,4
pxz,4dro,4cm6,4ww7,4jm6,4rx7,4lvg,4f3c,4jfq,4bg1,4f4b,4b3j,4paq,4a4z,4mkh,
4wnk,4umc,4ekr,4kib,4rbp,4b14,4tqq,4uw6,4bz9,4bdq,4fn4,4ktl,4p5y,4fpj,4l77
,4dvf,4n00,4o61,4b1l,4cyz,4b2z,4job,4b0h,4dgm,4dlt,4kbi,4u5v,4og5,4rih,4md
a,4dpu,4oht,4chn,4zaw,4azw,4eo3,4ybn,4zuk,4p3q,4o4e,4oe4,4xur,4m6t,4tln,4x
1p,4ei7,4k9r,4yvn,4dzz,4azb,4pwi,4bsd,4oop,4xtv,4cw3,4o6w,4pe2,4plc,4tr9,4
u5s,4n95,4wno,4hme,4a83,4rrg,4qx6,4x8s,4u6e,4j0h,4qtc,4rkc,4g0y,4a7c,4cvz,
4hp0,4ajo,4l7d,4efk,4kgm,4mrv,4nat,4y29,4cvc,4ds8,4dkb,4b4w,4wqp,4kot,4aif
,4a9h,4mg9,4mzf,4c1t,4s1l,4ag8,4a3h,4zvn,4jfp,4h1u,4y6l,4pl9,4pvd,4rvt,4jf
s,4f22,4cm4,4hzz,4moq,4u0s,4q0s,4tkh,4qpe,4c7k,4cc5,4o78,4lxk,4xly,4uov,4e
hk,4hkf,4i5x,4cte,4cpp,4coh,4yus,4rzq,4ode,4o10,4ghg,4c80,4jyu,4rhg,4gh0,4
rxc,4kv0,4y6s,4ehf,4kfb,4av5,4kak,4zb0,4l4b,4ai5,4oma,4usq,4dld,4uc5,4tn7,

4tpw,4tw6,4pqn,4pex,4z6h,4ywa,4zsy,4xkf,4bzs,4dpy,4e4l,4ls3,4k37,4wb9,4qxh
,4tle,4myd,4z0q,4d3n,4zqo,4bmo,4zgp,4akc,4oc5,4fm8,4xb1,4lik,4j46,4eyb,4tw
3,4d12,4nb4,4s28,4huo,4awi,4y6m,4goj,4lp0,4ltz,4c0r,4b1v,4nbn,4nmt,4j31,4z
1s,4bfv,4ri1,4qpl,4we5,4bui,4cqe,4uvo,4w9k,4eud,4x2g,4jvr,4n36,4gxq,4zdb,4
m1u,4yiw,4bnp,4jju,4umd,4w8e,4lv4,4cmt,4eh7,4j7g,4fhv,4unn,4ndn,4cn6,4xe0,
4ct2,4heq,4j4b,4wh7,4xsh,4xum,4j7e,4lwu,4wrs,4nfl,4ctc,4g3r,4cpk,4haz,4ewh
,4oez,4ifx,4zo8,4tvy,4al9,4x7u,4b4d,4chy,4pzx,4awp,4e31,4ix4,4djp,4ezx,4ax
6,4uoc,4ccw,4bt5,4eaa,4o25,4ise,4ysl,4u8w,4f9u,4c52,4kin,4xoo,4wki,4nw6,4w
hg,4gi6,4ask,4zvc,4qjl,4krs,4ww8,4x8d,4fqf,4nf5,4ej1,4bbx,4bjc,4wmw,4xd8,4
rpg,4zsc,4k55,4uw5,4o4z,4cpr,4jaq,4s18,4wzh,4pf5,4ltm,4dmg,4chp,4uf0,4e70,
4x7n,4u8y,4mzq,4q1w,4xld,4o5b,4n0e,4cfs,4ws0,4k9g,4oyr,4pp6,4req,4uor,4pgk
,4h3f,4ht3,4zbi,4kci,4tld,4w52,4r8q,4w4s,4deg,4buy,4gr3,4cx2,4rlu,4grb,4yp
s,4pmy,4zm4,4hvi,4jsd,4q0l,4fbe,4jzb,4pki,4cmi,4pzh,4ubs,4bb3,4qyn,4xtj,4o
fr,4mxo,4xye,4j0l,4q3c,4tyo,4y5i,4ddm,4pxc,4y7r,4qj9,4xv1,4phm,4o2r,4ty0,4
xiz,4ym1,4ohu,4ify,4bqs,4xnv,4nnc,4j45,4bmk,4png,4iei,4zyb,4fcw,4b4u,4zes,
4zl4,4iph,4zgs,4fcq,4jta,4jjj,4z0k,4x5s,4r4u,4hbw,4qty,4z8y,4jn9,4i7o,4zv1
,4mom,4iwn,4nll,4njs,4muq,4mw1,4cju,4nb5,4ple,4luw,4tl7,4y95,4gvm,4yt4,4qp
7,4zz2,4u0p,4xbq,4h5e,4l8a,4ch4,4qg4,4qxo,4o21,4hdf,4y7u,4a7w,4wt2,4v15,4q
r4,4xwm,4ufa,4deu,4zzz,4pyy,4jgx,4qly,4nuc,4r8m,4x6h,4uj2,4mbi,4y2x,4rfc,4
cik,4bfq,4dqk,4hgf,4ytf,4uvp,4nal,4yrw,4z1d,4pz2,4fqt,4c3y,4aja,4rf9,4pz6,
4a78,4h3g,4aut,4gmu,4c2y,4xqg,4dpl,4x5y,4g8m,4d3u,4mn3,4kba,4aj1,4dqc,4bfd
,4bzb,4rrf,4zjj,4pft,4blw,4kfn,4k91,4k2m,4pqg,4lbw,4xca,4gtm,4fdu,4cfb,4c6
z,4u3b,4dbc,4qbj,4m5o,4azt,4woe,4uff,4jbo,4uzh,4bf6,4g1q,4ym2,4wb5,4udw,4f
5z,4hdg,4tmn,4rvh,4yuz,4zhm,4cwd,4e1z,4gn9,4kcs,4hb2,4ua7,4qg0,4hdh,4eom,4
iqy,4igo,4y9u,4gtv,4ce1,4a84,4db7,4pc3,4i0r,4we1,4ttb,4pm1,4gwd,4pxq,4whh,
4m15,4dhq,4jaw,4iax,4ie5,4bh0,4rfw,4k60,4q5h,4njv,4zhd,4l2l,4g1n,4j1z,4kic
,4qp4,4piv,4qu0,4l9k,4ee1,4cr5,4o6e,4hpu,4jgp,4hgd,4ztn,4nw7,4zzn,4zpi,4nn
b,4hut,4akb,4f29,4cco,4qg1,4g1x,4jx1,4xcb,4p4i,4p0x,4klw,4czh,4be9,4azh,4p
ci,4xt7,4jpg,4l19,4dtz,4m73,4ual,4jgo,4xwz,4pao,4fag,4yh4,4dp3,4l2i,4he2,4
jap,4gjz,4c2v,4h9t,4qfy,4b16,4mwr,4hvb,4rda,4ccu,4eft,4o0j,4lv6,4gwk,4g28,
4avj,4iae,4lbs,4yi3,4eqh,4cst,4xac,4de9,4xt2,4l4m,4e3h,4wxf,4exg,4r2l,4jc1
,4h0x,4ceb,4hxk,4yl3,4wth,4kq6,4ac3,4edk,4lc0,4a9n,4h4c,4jih,4hy8,4u44,4o6
7,4jhg,4bv6,4w9j,4xf6,4ugc,4s1b,4qmn,4zte,4btl,4y2v,4p54,4g6i,4eql,4gjc,4c
ub,4ctw,4d09,4gjs,4aw5,4zdm,4b1c,4pzs,4hkw,4omk,4xx0,4xv5,4x7w,4y2q,4xzh,4
yvv,4fvz,4jnj,4rrs,4o04,4j5j,4kv9,4r6q,4yc9,4utg,4ugn,4phr,4jps,4pi7,4obh,
4f2b,4j44,4oyo,4hn1,4qe4,4n6h,4fq4,4l7l,4i7p,4msk,4two,4xg4,4mb2,4a5s,4iq7
,4ikr,4n8d,4pkh,4p4b,4qvh,4rxp,4e9z,4mb9,4blr,4jbp,4gzp,4xar,4qiz,4z76,4qu
g,4fic,4cwz,4qg7,4dxh,4lnx,4isu,4av4,4cme,4z3y,4xv2,4igh,4ad3,4jpu,4to0,4y
0r,4rlf,4cgn,4xzs,4ln8,4r5z,4ln7,4aj2,4zba,4jt4,4kvi,4c4a,4mtw,4k69,4oea,4
wa5,4bus,4loo,4dqb,4i0z,4jyb,4v27,4ca5,4q9m,4m9q,4zom,4bix,4dt9,4ynu,4avg,
4nst,4k4d,4fil,4ugh,4ns0,4zn9,4epw,4kwo,4zv8,4l33,4ugd,4ufi,4i2i,4hsn,4azg
,4ivt,4u9u,4jca,4axd,4izc,4y12,4yzt,4qbz,4lgg,4agp,4ws6,4b05,4oi5,4g8g,4ey
k,4zbc,4trn,4r3k,4wcm,4ph9,4wyw,4zqn,4wtr,4v3x,4ef9,4qa1,4m5t,4kpa,4ilz,4n
62,4gnm,4rrm,4twl,4ffg,4x1q,4dho,4d4s,4x0t,4dh6,4avn,4qzt,4zt4,4xfi,4b5v,4
bue,4mo4,4ui5,4s3a,4ur1,4heg,4n9y,4n7m,4d1p,4ywb,4tlz,4i00,4zsg,4nak,4eh2,
4j78,4hyh,4n94,4lyw,4pey,4yma,4cd0,4k7n,4d0n,4pjn,4c8e,4x21,4tm3,4mfl,4wzk
,4nm0,4v3w,4lr9,4jwx,4fll,4jat,4muy,4lj5,4iku,4xu3,4zsl,4oxk,4r33,4lnb,4np
3,4m2e,4ybz,4zli,4lce,4qjc,4eep,4d3f,4yh5,4duh,4u7h,4o1l,4wp9,4c4r,4xf0,4z
h7,4hat,4wqm,4zx0,4wp8,4fod,4ieh,4f1o,4qck,4hms,4yca,4hsx,4zw3,4tvk,4p3p,4
q3b,4g2g,4xoe,4lae,4m11,4j81,4e99,4hxo,4j55,4d34,4y9b,4c4f,4aaw,4dwb,4rao,
4cgm,4clo,4zlo,4zav,4xx9,4rmg,4nyt,4y5h,4wa4,4coj,4gr5,4zan,4wm8,4equ,4b1e
,4j1h,4pgi,4cec,4j1q,4p6s,4ufy,4qj8,4oc1,4gim,4fhh,4rce,4pb5,4zlg,4y9j,4za
d,4asg,4b34,4nmm,4crc,4n70,4f64,4eo6,4ncr,4kgx,4yac,4h75,4ont,4i71,4s3c,4d
ct,4rhy,4gqq,4d4b,4uiz,4qlk,4d9m,4bdl,4nrr,4n99,4b1y,4cex,4i8p,4aw0,4xis,4

pez,4fpl,4oic,4n7c,4w58,4kfs,4mcr,4xrj,4d8s,4upr,4j04,4rfm,4n86,4kf1,4ctz,
4iif,4jx7,4eh9,4d37,4a0m,4n82,4rga,4acc,4aw3,4y7c,4tmf,4fl2,4erw,4khm,4ien
,4e6a,4n9d,4i7q,4i73,4bgh,4bfy,4xo7,4kap,4idf,4rpj,4u6c,4c7j,4mwx,4nf8,4b2
j,4q6q,4q41,4jkv,4o30,4yji,4kz8,4ynm,4jip,4nzn,4uy1,4c9o,4des,4dkr,4pee,4g
xg,4ojq,4nfe,4i3x,4eq0,4mti,4drj,4frl,4m5h,4gm1,4mfq,4aql,4fli,4buw,4gsn,4
qb3,4whz,4mdq,4kzo,4oey,4moo,4oha,4rd6,4f6v,4hxx,4nir,4ai8,4zs3,4x9d,4p3h,
4f04,4rw3,4ea9,4da6,4w87,4hu1,4gtd,4ae1,4cwy,4to2,4p4j,4j8b,4q21,4hva,4zsw
,4plg,4dqe,4y93,4c61,4epj,4txe,4cnh,4fp1,4u1k,4mr4,4h8a,4bc7,4mr8,4v24,4eu
v,4ehq,4mv7,4r08,4og3,4jdi,4i0g,4del,4bj9,4g1v,4hia,4ih7,4zzt,4yha,4mu7,4z
9d,4gr9,4uj1,4upp,4gn8,4hk9,4lsj,4ys7,4zdy,4i52,4i0f,4wey,4dma,4cd1,4uam,4
bk2,4bu7,4f8n,4ugo,4ogt,4kcn,4otf,4cw7,4yw1,4x3r,4xtx,4tqi,4q08,4he9,4wdf,
4b5o,4g5y,4rac,4mzn,4gg7,4kkx,4k79,4hgh,4w9o,4c5w,4x7m,4i3a,4cgj,4rf8,4xe1
,4ieu,4tuh,4id1,4mr7,4lxz,4x1b,4d3k,4c9k,4mwe,4c6h,4e1b,4msg,4mp8,4eiy,4xv
e,4x59,4gss,4q1b,4rhn,4a3u,4bn4,4ct7,4lya,4zfc,4awf,4ur0,4jr7,4wq4,4zza,4t
f4,4m7v,4w9c,4p7x,4hlc,4luu,4xzl,4zhc,4zns,4h3i,4tvb,4mp2,4p77,4dyd,4gsp,4
lay,4kv4,4cwb,4gv0,4jsr,4e2y,4uye,4u00,4cgu,4r5f,4duo,4bs0,4qbk,4d9n,4qtx,
4cno,4gys,4mxp,4ni1,4oex,4ntm,4k0b,4cws,4ca7,4nkt,4pyn,4zcd,4kp5,4uc8,4xbv
,4ap7,4inx,4y0v,4y0d,4yi5,4ijp,4i0a,4rdy,4wkm,4qlj,4n0s,4zji,4cip,4lwp,4f0
7,4a8p,4cra,4o2p,4k5e,4pf9,4g09,4lr6,4kpu,4ifp,4mk2,4b77,4zal,4clh,4yhy,4c
k3,4c28,4avb,4l1k,4p3i,4r5n,4kig,4c58,4c4m,4tor,4m10,4zz8,4d3a,4xb5,4yxo,4
k2y,4yxi,4hwo,4wyp,4oue,4hxj,4jik,4rfl,4nau,4uun,4wvh,4zvm,4ywv,4fys,4zsp,
4k8s,4qn6,4mre,4adm,4lax,4kp7,4i9t,4wwz,4ymh,4g0z,4drm,4jqv,4bq4,4pbr,4b7z
,4wq3,4iqk,4v3u,4d7o,4uh2,4uhl,4qd3,4jaf,4keb,4jp3,4did,4b7j,4ht0,4ags,4ud
k,4h2v,4elh,4ylk,4mr3,4kz3,4qp2,4fqw,4ntd,4o91,4kys,4de3,4kvg,4pxb,4fmi,4z
yy,4lix,4ikm,4rvu,4mcq,4h2d,4y4d,4xzi,4ok1,4rxa,4llj,4o4f,4a07,4rxq,4dhy,4
ma6,4pjv,4aw1,4gvi,4ch5,4r5i,4nfs,4ibi,4nxe,4lsa,4knm,4rmj,4c5d,4kwi,4ehu,
4brc,4lrr,4x8v,4px9,4ryv,4zac,4e3d,4xp3,4zsh,4led,4pw3,4ifi,4xg8,4dfb,4dsg
,4img,4hmz,4wgf,4iva,4cph,4wb7,4as7,4wuj,4qmv,4dfw,4m74,4uqs,4p25,4pjt,4ug
8,4m6l,4p0n,4f06,4nv0,4ref,4idk,4c35,4jv8,4aqc,4qx7,4kfj,4j0i,4c7g,4o2a,4k
oy,4qxb,4brk,4nrt,4as0,4obq,4lw6,4cpq,4nt2,4qmm,4ifv,4dfn,4b10,4gno,4wm7,4
dlu,4ezq,4nxg,4kt8,4i9j,4eha,4ups,4lxw,4dlv,4qud,4eky,4zkd,4ltn,4r5m,4j75,
4c3f,4ly1,4ufh,4kza,4cwo,4rzb,4cib,4tq4,4mop,4ge5,4azj,4hso,4k1l,4nwc,4a9j
,4l70,4fbp,4jjf,4afh,4nd8,4tuo,4b0g,4yry,4hv7,4iu6,4add,4tla,4h69,4djw,4k1
8,4hax,4jor,4pv5,4jfm,4pzr,4gid,4lov,4i4s,4lol,4n14,4ava,4mv0,4cnl,4l64,4z
y3,4zrb,4xcz,4xj4,4b80,4mx1,4ept,4o08,4ddl,4drx,4u23,4hcx,4y24,4z3e,4uvs,4
j0v,4kat,4p58,4onq,4b8v,4c38,4y4v,4d3j,4wni,4cnz,4flj,4aru,4ghe,4knj,4nil,
4yho,4amw,4up5,4exs,4zu3,4hcz,4o1g,4n4s,4cm5,4gev,4qhv,4fx2,4ji9,4lnu,4igf
,4wcl,4eyw,4ayu,4hvk,4tnp,4o1d,4gh5,4lhv,4j43,4xh0,4rad,4azs,4zyt,4ca8,4yb
t,4yhi,4bux,4l10,4ees,4de8,4gpt,4n8z,4k3l,4k41,4if5,4bib,4dlw,4txj,4idb,4u
gt,4p6a,4jln,4cs9,4bak,4xqe,4iy7,4p86,4pb4,4o1e,4d7a,4pwf,4u5o,4eqx,4qrf,4
eyf,4heu,4eyu,4dlx,4ea8,4uss,4cpi,4pab,4yk5,4d06,4are,4owm,4v20,4kao,4b1m,
4ovz,4xjc,4zbr,4qjm,4y79,4bxk,4zxy,4ch3,4m51,4kwc,4ogn,4mpr,4jlz,4wmu,4qrt
,4l1f,4mty,4n65,4p7u,4o70,4hjt,4n9i,4ivd,4drp,4o79,4cld,4kql,4jy9,4was,4kc
f,4tos,4s1g,4pv9,4gto,4jqk,4cf9,4i3g,4lkk,4xhk,4jdc,4zvy,4olt,4yfa,4q4o,4i
e1,4a32,4a71,4jnc,4l6c,4uyd,4c6x,4ekv,4y6u,4wyd,4p10,4b3d,4ce3,4u03,4uzl,4
c4t,4hdo,4iai,4qr5,4m5n,4pg7,4zsq,4zg6,4phw,4yad,4i7f,4q03,4inq,4bps,4muv,
4m0y,4lxh,4ndp,4hgp,4b69,4npl,4efs,4lfn,4e5j,4uiy,4nud,4ntx,4lcp,4a14,4kov
,4ces,4h1m,4fai,4oj8,4jd3,4iwz,4j34,4nbw,4gih,4eu9,4x06,4igs,4ju7,4zvi,4hf
4,4rl0,4cy8,4p5z,4urg,4ja8,4avi,4njt,4rro,4j21,4b5t,4bbh,4ns3,4mlo,4nwi,4g
fm,4eyg,4is0,4ysw,4y76,4uoh,4trj,4tri,4q72,4cgd,4iea,4bk3,4otv,4q0k,4fl3,4
oqr,4k9t,4lfi,4zfk,4dvq,4hby,4rg0,4aa7,4fgc,4x8t,4cmf,4a6k,4m0f,4mw5,4n8v,
4mwd,4m7j,4c6i,4tz1,4z6v,4hys,4na8,4ie2,4clw,4sli,4qgg,4pni,4l8v,4mcc,4kwg
,4qll,4ciy,4yp7,4n8i,4xf1,4d5h,4ugk,4e1k,4rdd,4qee,4puj,4tqp,4kva,4r6e,4pk
t,4d5d,4ga6,4qu5,4cg8,4uib,4pf7,4obj,4utv,4cq8,4ib6,4o75,4dm8,4uai,4nqd,4f

yr,4o76,4gts,4und,4mta,4du8,4pfx,4x16,4eoj,4phk,4cj5,4upt,4fa3,4zfv,4ucf,4
i6q,4jrb,4qc0,4gg1,4oes,4zzw,4cng,4wh6,4x5p,4p06,4uqr,4xl4,4ppf,4w85,4rxh,
4mds,4fpc,4czm,4bk1,4to8,4fem,4uia,4je5,4czf,4ui8,4zhl,4brh,4r5t,4gsz,4mby
,4tzt,4peg,4yzc,4eab,4j4o,4nfn,4ivy,4x6m,4o7e,4mgb,4nh8,4lkq,4ccc,4d2s,4ei
4,4xvu,4lrl,4ip7,4whm,4aoa,4ps5,4o8o,4cth,4u7g,4zrm,4a33,4x1x,4oks,4cpe,4i
9i,4cck,4m4b,4z69,4rql,4wjt,4n96,4z4v,4s29,4xt6,4mk0,4gv8,4x8o,4uda,4ui7,4
itp,4ze1,4c1w,4k4q,4ehr,4xv9,4gr1,4o1b,4g5g,4wdt,4dce,4l09,4r07,4fua,4ux4,
4u8z,4cf1,4obo,4ajb,4j52,4can,4awj,4i5h,4l8z,4cq5,4q7w,4b4r,4ce9,4dst,4a9y
,4b9q,4dsu,4w1w,4f97,4jpl,4r6s,4ncw,4n0d,4wpf,4c4s,4tsz,4fzj,4c39,4ew3,4hv
d,4k26,4psw,4z6i,4hze,4hog,4uux,4yao,4usf,4aq6,4jmo,4r74,4r9c,4rcd,4tun,4i
7k,4y2j,4r38,4ies,4b52,4v1f,4jbq,4xf7,4plv,4hdr,4b6q,4zi0,4o4l,4dcs,4gcz,4
m5r,4j48,4kpz,4xoc,4opi,4fob,4jr5,4ha5,4znr,4jfi,4k1z,4dy6,4wvt,4ugp,4zuo,
4jn6,4g19,4ypa,4ap9,4ipj,4lyf,4o12,4zyc,4cez,4llp,4ba5,4mrw,4pwg,4n5p,4zqx
,4mpn,4l03,4wtv,4bh4,4k39,4giu,4di8,4wwp,4ezt,4xub,4xc0,4iz0,4hds,4cap,4ok
z,4j33,4mrp,4c66,4nms,4uwm,4s3e,4tlx,4qme,4ryc,4qac,4lts,4oeg,4c2w,4y2y,4z
5w,4e2o,4g8y,4o0z,4rwf,4l34,4kbc,4jh0,4e6c,4n1u,4uhe,4kni,4ad2,4bu6,4tlb,4
l61,4mf1,4wfr,4udb,4uvn,4mfp,4yrd,4j57,4jqh,4r95,4css,4xu1,4e5h,4n6g,4c4c,
4odv,4p7k,4oyp,4dbh,4cjx,4fus,4bge,4xsg,4fk6,4cps,4kyk,4gfd,4cho,4wnu,4fik
,4p2t,4uqc,4o77,4p32,4l4d,4kqg,4zei,4o4p,4wmy,4b2m,4b33,4gpe,4a59,4qvx,4hp
t,4d46,4o43,4ekq,4whk,4nxv,4ek1,4u5n,4fcd,4wa7,4ndq,4bb9,4jls,4csj,4i87,4i
90,4xtr,4xv8,4kqw,4w8d,4xud,4q81,4d7j,4s3g,4fg8,4l4f,4iju,4n3r,4ork,4ohx,4
uh0,4ke0,4zx5,4az6,4k4a,4f9w,4e90,4rgq,4hi0,4ie4,4b47,4yt5,4u7w,4kxq,4unq,
4mww,4gqe,4a9l,4k3q,4cyq,4fh5,4co4,4ydm,4mbp,4xir,4r0m,4x8l,4itr,4x8u,4e2z
,4ehz,4k5p,4mor,4uxm,4n4m,4j0j,4ced,4tqc,4g2j,4cs0,4prx,4b00,4u1b,4zxa,4bf
w,4unr,4ay1,4c6j,4ffs,4oyb,4cb7,4g68,4aig,4xvr,4cjf,4dcg,4txn,4u0k,4kjk,4k
yd,4uxh,4cd8,4feg,4iw8,4n6w,4cjw,4lq9,4ufl,4c2c,4es5,4yyl,4wv8,4llk,4kec,4
cfl,4b7r,4lc1,4pnz,4pp3,4i9z,4a95,4pot,4n6z,4nul,4epv,4oit,4hf8,4g16,4m0z,
4qii,4ebf,4ufd,4e43,4jv6,4yb8,4gus,4d04,4zoh,4qlx,4azy,4c1m,4tm1,4i03,4ufk
,4u69,4k2f,4q07,4n9z,4l4v,4fh7,4qp9,4jmw,4qya,4oqv,4o1z,4o4i,4zcc,4rmi,4i4
u,4cbw,4ii2,4gu5,4fny,4f2q,4mqy,4eh6,4xct,4fz3,4ok6,4cf8,4q4k,4agq,4bpz,4j
xh,4wtu,4u8p,4g8c,4k3r,4bo0,4gtt,4c8g,4cnk,4ipn,4pi8,4o7g,4esi,4eu0,4wy6,4
ige,4wx1,4rh5,4bhn,4c7z,4aml,4mrh,4if4,4crj,4bqh,4buv,4ceq,4q3s,4wh3,4ict,
4y6r,4nqv,4gbm,4ago,4ioq,4blt,4b74,4xh4,4x5z,4q83,4aoz,4kh1,4g31,4xuc,4wev
,4i9h,4mka,4g9n,4m8y,4ayr,4crf,4edr,4u1s,4d9h,4m1o,4jbl,4m2v,4e9u,4oew,4s0
v,4xxs,4wnj,4gut,4aht,4x2a,4wkc,4x58,4kiq,4jdk,4ij1,4h1v,4lbd,4mk5,4yag,4b
gx,4z3v,4mua,4jck,4n9c,4lxd,4n3a,4xdv,4dvv,4z6p,4mxj,4a62,4plh,4i8x,4kz5,4
bq0,4dsc,4cp5,4ezz,4ots,4u8i,4f13,4xd1,4xf4,4fcp,4o4j,4ybi,4ojz,4m1t,4jwk,
4ypq,4l1a,4ohm,4pxd,4fxq,4mra,4bf1,4k1x,4c69,4gqy,4wke,4urs,4br3,4ael,4i3z
,4ju4,4u22,4ad5,4ad6,4fc6,4x7x,4wnm,4z4z,4tn4,4b5w,4yhg,4gcs,4zt7,4u0j,4a9
f,4a6v,4rn4,4tqb,4con,4k3p,4bjh,4cml,4qb2,4nu0,4uh3,4e3g,4zze,4d6b,4bvn,4o
gj,4qvb,4foj,4ocq,4qs7,4hgw,4uh5,4std,4doe,4g9d,4nxf,4eoo,4gk4,4cao,4fut,4
h6b,4lbl,4r2u,4d5v,4zw5,4zhf,4bh3,4pos,4aq3,4qu8,4rnx,4lj3,4y46,4b5p,4hhy,
4b13,4az7,4uh4,4u36,4kwp,4apo,4p4c,4fm7,4gj2,4i5c,4b35,4chs,4a8v,4zev,4mwu
,4lz7,4ttu,4oka,4odf,4v3z,4xkr,4m6j,4al1,4zah,4x6k,4zxf,4cp4,4xas,4x13,4jz
e,4e0w,4ie3,4d81,4dlr,4zz1,4jag,4nu5,4h5c,4krv,4fcb,4ylu,4xdy,4i6b,4kz7,4k
yr,4wya,4zsm,4jft,4ydn,4k0w,4x6i,4xfh,4c2x,4atj,4e30,4r68,4ere,4ymg,4g4s,4
wbd,4jcf,4mn7,4mqq,4ze2,4wj5,4h2y,4rep,4eq4,4i56,4y9e,4lxq,4nwx,4rj8,4que,
4uvv,4k7i,4a8h,4u70,4hgi,4f4s,4brf,4ugi,4tqd,4k0y,4eoi,4b4v,4zwo,4q4x,4jsy
,4a4x,4fzv,4az3,4mk1,4a2j,4cqb,4dw4,4i9a,4tx6,4cx1,4rr9,4wdw,4v26,4zs2,4qm
z,4br2,4s3r,4z28,4fhw,4pbt,4dhr,4eol,4kxm,4b32,4jh9,4lrw,4qt1,4lp6,4opo,4q
o5,4i7s,4wvi,4xg0,4pst,4gyj,4yfz,4odt,4ugj,4i06,4zy6,4zn5,4o74,4o63,4upm,4
ug5,4ipi,4rlt,4oxx,4kzu,4i9u,4amv,4hdp,4xdz,4jhh,4cmu,4e5d,4eb8,4obp,4hup,
4gee,4r5x,4p8v,4kcm,4n37,4c14,4xqm,4dcu,4xf9,4d58,4xbl,4oba,4ntc,4ezy,4p8p
,4ona,4jzy,4nxu,4pmp,4d71,4hwk,4ch6,4rjj,4bnx,4jij,4m6x,4uyg,4b71,4rrq,4ue

h,4txm,4e5x,4l9w,4zaz,4l91,4ww5,4ff8,4d6a,4b2w,4j3d,4wr7,4od7,4m1y,4b9h,4u
ry,4qe5,4fou,4zwx,4wgi,4a6d,4z5x,4p5q,4zqc,4oyi,4q7p,4izy,4zzd,4f4p,4ima,4
rv3,4hvp,4l8g,4wmt,4bz7,4nm9,4iue,4nra,4wy1,4za9,4j7q,4a9o,4wk1,4fhf,4q15,
4hzt,4cgf,4okw,4cla,4oof,4ahv,4hdm,4np9,4lpc,4c6k,4wvb,4i0d,4d2r,4ioo,4yw2
,4cnf,4bgi,4j5r,4kcp,4n1t,1nww,1tng,1zuc,1a1o,1xd1,1fw1,1sqa,1ee1,1o01,1gw
x,1zpt,1w4z,1rxc,1qsr,1gex,1pbb,1g9q,1d7l,1b62,1vr1,1eje,1goy,1gn8,1a9z,1t
q4,1zj6,1z45,1pno,1tcw,1kgz,1bwk,1hgy,1b9s,1vc8,1bso,1zp3,1d1q,1dqx,1kbo,1
hpb,1x8v,1cd2,1roz,1b8s,1zj2,1a4q,185l,1lrq,1kwx,1ksg,1kji,1qjg,1gse,1o3m,
1d5x,1uto,1s2g,1xel,1y9q,1n4q,1n22,1n0t,1v2o,1zn9,1flv,1r5c,1v2v,1ez1,1tu4
,1hxw,1ki8,1txf,1z8d,1a78,1fm9,1ami,1uwf,1log,1gg8,1g4t,1gq2,1va6,1a5w,1e2
k,1uqt,1hxd,1xjf,1m0n,1gj9,1bh5,1asa,1bo8,1dgl,1tlp,1urx,1oxv,1fxv,1in8,1e
bz,1ii5,1hzy,1m6p,1s5o,1jx1,1y2e,1fnd,1bcd,1cl2,1uks,1i0v,1zgv,1r33,1um4,1
p1k,1yxw,1v6a,1ma0,1fyn,1me9,1h1h,1oxr,1gj7,1zip,1jw0,1v2j,1s3y,1pmv,1icq,
1c1p,1gx6,1hqw,1com,1diw,1ghb,1wc3,1iuu,1lr4,1tf8,1jc9,1bwb,1w0j,1me8,1ocj
,1fwy,1z0n,1aq7,1d0m,1js4,1it6,1bjk,1c2f,1g46,1upm,1nce,1gqy,1urg,1pd2,1iv
d,1uyq,1x2e,1xjd,1g81,1ftl,1rua,1nuw,1pnf,1uu4,1z4q,1t86,1yok,1cjw,1q84,1n
ht,1i0l,1x6v,1e8z,1csc,1yrs,1lsz,1h72,1isz,1o4h,1d3v,1m0u,1zq9,1ldy,1od3,1
nkh,1iup,1rvv,1p7v,1isv,1a55,1rw8,1g3q,1a3v,1nf3,1xp8,1sha,1nvr,1kek,1uxy,
1nzc,1lf5,1qhg,1hwr,1css,1epl,1e9b,1gjc,1o2j,1zd3,1ifs,1zzs,1mzm,1lmp,1qk5
,1h74,1u6r,1nsf,1fce,1veo,1qan,1bfc,1iwe,1fo2,1j54,1eyj,1rf7,1gg1,1gxs,1tr
m,1dvr,1ev9,1o6k,1p77,1o2s,1zsf,1za2,1fup,1wlk,1i3m,1ne6,1lfw,1jv3,1icu,1h
p1,1fiw,1sdv,1bzc,1kdr,1y3w,1sjb,1r4p,1mbb,1vyq,1mr3,1fuy,1m4h,1t3z,1o4b,1
lif,1hdy,1yv3,1kr2,1eyk,1nny,1htb,1rdi,1rsi,1vac,1a96,1t73,1sgj,1de6,1axr,
1wg8,1dvs,1u8a,1v2l,1xs6,1c1v,1nyw,1coz,1ztz,1m99,1yn6,1vq2,1zk7,1guy,1xiv
,1czk,1rrw,1lmo,1vg8,1yns,1nnu,1lah,1yft,1o4p,1zg9,1bnn,1pz0,1pk7,1v9h,1j0
x,1wm1,1eyr,1tuu,1j7u,1wld,1z5u,1gg6,1q1r,1xor,1d6z,1xmm,1yxm,1cw1,1cr4,1t
wi,1pvc,1hfb,1w61,1kjf,1jvi,1i7e,1q6n,1uwz,1o5c,1zsx,1xdg,1k7v,1dx6,1o61,1
qa0,1p6w,1nlv,1nl9,1zly,1cr1,1xud,1m8d,1yag,1jin,1zyk,1n24,1pvg,1tjp,1vfv,
1uke,1mid,1qxr,1tph,1tc6,1i2f,1n4w,13gs,1ygb,1vsv,1f3d,1p7l,1hdg,1q63,1xkk
,1wb6,1kfj,1gxa,1eix,1kei,1nu4,1bfb,1wkl,1h5u,1dyr,1ojr,1m8f,1js3,1u1e,1l4
e,1qoq,1d0v,1lzg,1cde,1zos,1pb8,1lny,1tde,184l,1m5b,1e2d,1d1t,1ing,1lp6,1u
io,1m7h,1o2l,1n5d,1nme,1qit,1szm,1jxm,1uak,1zmd,1jgl,1hq2,1z1h,1jnk,1dhi,1
zkn,1rzm,1slt,1jpa,1byg,1o4l,1m7i,1opk,1jd3,1uyf,1hdr,1p2t,1v3e,1ndy,1sd2,
1y5b,1c1b,1s3f,1v7u,1g7s,1h61,1qb1,1lq8,1g6s,1kc2,1tku,1mjl,1v6d,1ejj,1rvz
,1odi,1frq,1zhr,1vbr,1w58,1akr,1ktg,1g52,1sb9,1ted,1tn6,1c5c,1xe7,1hwi,1lw
d,1h70,1nqu,1n07,1rs7,1zk4,1fqd,1srg,1ele,1ylu,1vhw,1jzs,1lo6,1nni,1dg1,1r
ul,1j39,1hkn,1l5l,1at5,1n2b,1b57,1r9n,1u0j,1t76,1h65,1s17,1s3g,1z08,1i24,1
e5j,1gx1,1e7b,1gwc,1fp2,1bcx,1n6m,1jj9,1k7e,1dpf,1ofv,1dtn,1vwn,1y3a,1qpq,
1dj9,1zp4,1uuo,1g79,1nv9,1pua,1uyc,1nvk,1hj6,1zne,1qlh,1oh2,1mbz,1phw,1u31
,1icr,1o9o,1xqp,1drf,1e19,1utz,1n3o,1x96,1bil,1hd2,1vru,1add,1maq,1ns0,1nb
u,1j9g,1oyo,1hqt,1jpg,1p7p,1c5s,1qzz,1xbo,1l83,1skj,1yum,1hfr,1iep,1q5o,1w
li,1pg0,1o3k,1t40,1yqt,1l7e,1kbi,1qyg,1ghz,1sm2,1j71,1i1m,1kbj,1c7r,1kj9,1
jqi,1g9v,1bs6,1eqy,1pbq,1p6o,1z0k,1mky,1qb8,1i6k,1amr,1kt7,1axd,1tnd,1efh,
1g2o,1k08,1a9s,1mt6,19gs,1sbr,1xkv,1qv1,1cm0,1b4b,1g7v,1z0j,1ov7,1yol,1xqk
,1r5h,1fxs,1njd,1vag,1eax,1pox,1jio,1o1s,1own,1b0m,1p1n,1o72,1i80,1knm,1ie
9,1gs3,1fg7,1l7f,1n3i,1kdm,1cy6,1q54,1v2u,1w1y,1rjw,1krr,1t90,1bp6,1y8y,1i
x7,1o8a,1y1p,1g7c,1gs5,1f17,1zo8,1gr1,1fd0,1v2r,1i7z,1zsq,1ms7,1fxq,1bky,1
szd,1sdz,1a0q,1w55,1yno,1syl,1n5v,1ius,1gt4,1hkk,1e1m,1i7u,1byb,1tnk,1ot1,
1w0h,1pj4,1o2y,1hto,1nnk,1qpc,1ryc,1wbk,1v3l,1ec1,1ung,1g4o,1w34,1mwe,1nc3
,1uh4,1q95,1w19,1swk,1wbl,1dli,1l2s,1ti7,1jlv,1h4i,1v3c,1rtf,1puu,1nae,1i5
r,1ivr,1ish,1g1d,1txi,1nvs,1bkf,1sdw,1j17,1yat,1t15,1cxh,1ydp,1u59,1jkj,1l
ss,1e7h,1c6x,1bdq,1q36,1k0j,1a8r,1sc3,1vhl,1urs,1ns7,1o51,1e1o,1f9g,1ml6,1
qnr,1ix1,1g8o,1l4g,1z22,1q66,1g0i,1v5y,1trk,1cgk,1qfv,1oyy,1ndp,1jwy,1if5,
1xk5,1n2v,1nzd,1czl,1l4f,1bwn,1uxh,1jha,1zoe,1j1g,1p29,1g4k,1kll,1u4j,1pr1

,1m0s,1xkx,1ir3,1t2c,1s4o,1z3t,1vbj,1z35,1fkg,1lf0,1qca,1kyz,1tnj,1jk7,1kw
1,1g7t,1ql7,1w62,1bvy,1urd,1h7f,1lvm,1n5i,1d5m,1ojz,1r4f,1bup,1p9p,1bnu,1t
lx,1b55,1hsg,1sqz,1w77,1dd6,1p9l,1y2c,1z38,1us2,1el9,1w49,1uy4,1j8q,1bx1,1
kxm,1i4f,1h69,1nsu,1pjs,1n83,1ftn,1oyq,1q8t,1hvs,1l2a,1pck,1bfd,1cs4,1r89,
1udc,1fpg,1mm6,1zei,1gor,1kce,1xh7,1dqp,1px6,1bgn,1z4m,1nsx,1yti,1i6m,1w6m
,1n9g,1at6,1w6y,1aco,1dnc,1xhl,1pkx,1jv4,1u8k,1h5q,1dob,1xp9,1hmw,1az1,1kr
e,1zoa,1w2u,1syv,1yzq,1im8,1o7o,1uxk,1vwr,1zeg,1tpc,1gmp,1ai9,1qga,1x15,1a
j8,1etb,1ll2,1ae1,1jfh,1o25,1qew,1t64,1vlh,1nmy,1w1p,1r8s,1lbb,1dm3,1br6,1
fyr,1syd,1smc,1o47,1k7d,1km1,1w7k,1yp0,1d6e,1llf,1los,1n3q,1d6a,1tt6,1gqk,
1d4h,1trp,1my4,1mdl,1z9g,1pxd,1jdj,1k6m,1w1r,1wpq,1qnf,1xq0,1ctt,1k1u,1r5l
,1y1m,1dmy,1u8q,1khe,1fkx,1g7p,1wbs,1mtu,1h8g,1q91,1hz1,1axa,1r15,1t2a,1g2
l,1tx8,1zoh,1ucd,1hm9,1xhy,1zdf,1l4k,1rnj,1ycl,1ia1,1gia,1yw7,1jso,1icm,1l
ek,1bnw,1s2c,1q2c,1wut,1w3j,1g8z,1n31,1xje,1b7t,1ds6,1jub,1skq,1vji,1tc0,1
pj9,1h5t,1hih,1vja,1p1h,1fyf,1p6b,1wdy,1s0z,1pph,1pmh,1ffq,1gii,1i8q,1xbc,
1rp7,1gco,1a4w,1eby,1uxj,1xnk,1r80,1xlf,1ej2,1ec0,1fq5,1njs,1drj,1em6,1q8p
,1tu7,1u2z,1x31,1lyb,1v5z,1u0g,1juf,1wkw,1t32,1a2b,1nuc,1uu7,1cvu,1hso,1r6
d,1ie8,1wsw,1ltq,1i1q,1ftj,1q65,1e56,1q0s,1hmp,1g05,1jvs,1x76,1vp6,1zui,1y
li,1c96,1w4x,1ur9,1kdo,1q3h,1ugx,1t1w,1aef,1t1z,1fpc,1n2i,1zq5,1zxm,1wua,1
s8f,1one,1rkx,1g2n,1kq0,1xpz,1onq,1zjz,1y9g,1hi4,1ony,1ykd,1m64,1e4w,1yj6,
1pye,1k0c,1riv,1gz1,1wzu,1hsa,1hm2,1wu5,1rby,1ogg,1h2b,1syh,1qja,1rvt,1wbm
,1kpu,1y60,1f6t,1oio,1mk1,1a1e,1as3,1uh5,1x08,1jdz,1so3,1een,1nqw,1z5a,1cp
u,1agf,1bit,1xx6,1wq4,1kqo,1eez,1ojp,1ph0,1vik,1duz,1al8,1tvw,1gz9,1nxy,1e
1l,1db4,1lc3,1so4,1gsy,1v16,1o03,1keu,1iex,1b5q,1ukz,1kgj,1k9e,1bzs,1sj0,1
fs4,1p4c,1vwl,1jz7,1aex,1y7t,1ri4,1gqg,1pnq,1rs8,1ovj,1eye,1hwl,1gt6,1b38,
1fa9,1dyh,1vrw,1ppj,1upp,1aka,1mq0,1way,1lrk,1lra,1ia2,1zpl,1nhi,1w8u,1rk1
,1uad,1six,1d8t,1j96,1qr1,1cbs,1lqa,1gve,1n52,1znz,1mrq,1uh2,1ake,1wxj,1r5
5,1kel,1m21,1lij,1d3c,1s36,1zng,1c7t,1h7w,1ei6,1xu4,1iit,1os2,1bxs,1su2,1t
j1,1e55,1mvs,1lnm,1hyt,1brp,1efc,1mvy,1xh5,1xvu,1jvu,1w9d,1ry0,1a9q,1s20,1
jt2,1tb7,1kk3,1kya,1h1d,1ohj,1ykc,1fin,1ra7,1xp0,1rxk,1utc,1xw6,1nah,1zzu,
1zvi,1gar,1die,1uvt,1n2r,1map,1pd9,1ce5,1k6t,1i2c,1t8x,1obh,1qxk,1lxm,1nqo
,1p2a,1u2s,1kie,1z34,1uag,1gxw,1vjo,1qs4,1zgd,1ekx,1e5f,1yoo,1ivc,1dor,1z0
m,1dbt,1oum,1efz,1e2s,1mrg,1so5,1nqa,1wng,1vwi,1cf2,1lt8,1hm3,1d7s,1qsn,1g
2a,1rf4,1dy4,1k3l,1jn2,1l4n,1i0r,1cbo,1fk3,1ed4,1g36,1q5k,1enu,1d8c,1xu7,1
rs9,1mx3,1sqf,1g32,1dmk,1vco,1bfv,1b4x,1t9s,1wkm,1tt1,1dl2,1ngp,1gro,1f0l,
1yqy,1kqw,1j4r,1yl1,1z42,1lca,1ifx,1i14,1a9y,1dbv,1fkf,1li2,1ie3,1yak,1vzu
,1ypg,1puj,1ki7,1opb,1oya,1v7c,1xuo,1lua,1bs8,1x7b,1o9v,1vyg,1str,1hjo,1g1
7,1anc,1jgd,1uuy,1pj2,1lzj,1be9,1x7j,1t20,1qy1,1m04,1h0c,1c0l,1ivb,1czh,1m
0b,1qji,1yon,1en2,1hpo,1gnr,1e6z,1j49,1tip,1w22,1dvt,1t4g,1snc,1b4v,1pt9,1
e6x,1ta2,1o4m,1nqi,1t44,1hzp,1p7t,1rp0,1oa7,1sdt,1g0o,1lpk,1okw,1o2q,1u30,
1x8d,1v3x,1utp,1chw,1j0n,1nm6,1g85,1h2u,1mxd,1a5b,1oxl,1frp,1nrl,1ta8,1btc
,1k4q,1e3i,1ys4,1c83,1l6g,1xi2,1t91,1u95,1zed,1fv2,1ymt,1o0o,1e92,1a5v,1mx
u,1i1e,1qhi,1djr,1he2,1pyn,1c2t,1iwj,1fdq,1eo7,1vl1,1ni1,1i5a,1r58,1ld8,1j
mi,1fro,1d6w,1swr,1jk4,1wuu,1d7y,1w1g,1uop,1qv0,1p39,1yw2,1p2b,1iov,1rzu,1
vb9,1rpf,1ghw,1h84,1jhx,1y1a,1b56,1atp,1ym2,1qbt,1r38,1gw9,1lro,1vbo,1szs,
1v2n,1yfs,1o9s,1jg4,1tpy,1n9l,1ah0,1fbz,1f4l,1r6n,1id0,1exf,1y0g,1a82,1jwt
,1lbt,1z28,1lhn,1ndf,1cc4,1g2k,1r0e,1b16,1drb,1h3t,1zao,1gwm,1t26,1o9j,1nx
3,1wl4,1ot3,1p2e,1b6t,1g37,1e3g,1tqw,1zn7,1thz,1ntv,1bid,1iqr,1di8,1uf8,1n
gs,1nu3,1bqe,1wc5,1ur4,1k3y,1j7k,1am6,1hlf,1p61,1rcf,1uqu,1yzk,1trb,1znh,1
ch8,1mqb,1o2x,1mf0,1g53,1rqh,1vjc,1n6b,1xoe,1icp,1nvq,1v2k,1phk,1yt0,1o0m,
1br5,1fw0,1loh,1waw,1jf0,1j6z,1o3f,1jaq,1ofz,1u00,1quf,1nuz,1u3q,1j01,1zr8
,1pzo,1z17,1qmg,1jpz,1xan,1sb1,1pb1,1u8y,1daf,1x39,1jqw,1fdj,1riw,1vyf,1tk
f,1t5z,1vwg,1eef,1j5p,1ro9,1lkk,1hpv,1s89,1mu6,1q8a,1iwi,1f4g,1t1x,1m5f,1v
dn,1fjt,1yef,1d1g,1gx0,1fk6,1d4k,1y2d,1cqf,1epb,1i7b,1dqs,1xds,1y8q,1cle,1
b30,1wlw,1h2t,1o49,1om1,1osh,1nga,1nhj,1x3m,1c23,1q0n,1u3i,1lho,1t87,1iow,

1nn5,1h10,1oiw,1g6o,1aha,1rf2,1nwk,1gky,1m32,1vgq,1nus,1o2a,1rhc,1rlj,1zk5
,1q4s,1fu4,1m00,1uih,1g6k,1zuw,1m2j,1ur8,1q6p,1dtd,1p0h,1c5y,1sny,1rpj,1ns
y,1k74,1x82,1bb6,1mja,1kr5,1dg5,1ryh,1loa,1sm9,1ryd,1xai,1iny,1gu3,1e62,1j
kx,1rwc,1xih,1ja9,1ppm,1gah,1fk9,1s7x,1u32,1okl,1and,1c5r,1v08,1vr0,1btn,1
ooq,1fki,1jys,1v6k,1dim,1f3a,1yb5,1o2r,1xuj,1rsg,1tw6,1php,1h6c,1jg2,1flz,
1oxn,1hg2,1t0l,1w84,1mh5,1erb,1nzz,1e3z,1v0k,1lnx,1owd,1cy1,1pxc,1x7n,1q19
,1zz2,1ex1,1gg2,1nw5,1tcs,1nai,1h0v,1lbk,1u72,1ian,1lid,1pea,1tnh,1izc,1kg
i,1mj7,1i1o,1jqd,1vps,1b3x,1dna,1t65,1c50,1f0x,1vwe,1svs,1gpk,1tyo,1lst,1n
di,1m26,1gre,1qsc,1cg0,1vwb,1zy7,1m7q,1umg,1pcx,1rnn,1j18,1otw,1sl3,1l7k,1
i2l,1mor,1ida,1ofs,1bmd,1f0u,1bm7,1hxk,1k7w,1b8n,1w3y,1huv,1dag,1gj6,1vm6,
1psc,1mfc,1kbg,1m79,1sa4,1rh3,1k8z,1e27,1svh,1iuv,1pbd,1tb5,1ce1,1ot2,1jay
,1rg7,1hna,1aeo,1bnt,1t7k,1lke,1u4o,1moo,1qzr,1mzn,1i31,1gt1,1s9x,1ydt,1lm
q,1aej,1eo5,1csr,1zt9,1vys,1li3,1gic,1yki,1p5b,1cdo,1hsr,1tmc,1ccw,1vwp,1k
1p,1yp4,1wgc,1dnp,1jhq,1nw6,1lta,1hye,1afe,1iub,1w31,1ttm,1ekk,1h4h,1yy5,1
hfa,1jld,1iuc,1m66,1o2o,1t2e,1oif,1gpn,1jao,1qk0,1o68,1an9,1i9h,1n7g,1vwo,
1q99,1z29,1fla,1c9c,1a8b,1elr,1dht,1xjg,1q8v,1zot,1rga,1rcv,1bo6,1xng,1l1e
,1unq,1pkd,1qkm,1wu1,1e1x,1u2g,1v6v,1lxc,1k1j,1gw1,1ur5,1t8t,1ynq,1bl7,1f9
u,1xcw,1v8y,1y42,1ib6,1h9z,1mw2,1v79,1qj1,1xe5,1pcm,1l4h,1bir,1nhs,1c9h,1q
d0,1tbw,1v40,1swg,1jcs,1h8s,1gkc,1z4i,1ixe,1oth,1duy,1jrb,1zny,1lob,1ats,1
je1,1iid,1yil,1ns2,1o9w,1vf1,1wvb,1dek,1apu,1d6u,1y93,1u1x,1os5,1byu,1eta,
1oiq,1xl2,1m43,1xq6,1udb,1j3j,1hpu,1x7g,1iih,1o1r,1b6m,1jtk,1eos,1lvw,188l
,1xoq,1lzi,1am1,1v2s,1r4a,1kav,1uy8,1goj,1bzl,1gan,1upu,1r0x,1oab,1sw3,1oa
r,1yoe,1z6q,1dvz,1tyx,1azs,1kr1,1juv,1jai,1wdi,1n7f,1nw7,1qg4,1p5d,1h83,1m
qg,1y7i,1i7c,1kev,1b1y,1c2i,1ac8,1oim,1nfx,1upc,1oc4,1k1m,1vze,1fvt,1xag,1
v3t,1eyi,1uxa,1fty,1z4n,1rej,1inx,1ov6,1utl,1yc1,1tqf,1y2a,1c5t,1r18,1wax,
1k1n,1bp1,1rob,1k3a,1fui,1kzk,1doe,1zg7,1gx9,1uhj,1ofd,1r1j,1yiq,1zc3,1flr
,1l8p,1c8k,1qrp,1qh0,1sr7,1dqo,1yu9,1mxg,1jxn,154l,1ns8,1xt8,1rr6,1kqu,1ds
s,1goq,1h48,1lw5,1nge,1x38,1bzm,1ftw,1gi4,1k3t,1po7,1h5s,1nf5,1llq,1pwm,1g
6n,1g0n,1owe,1u7t,1hww,1jyq,1xbv,1vza,1mmn,1wop,1hg4,1eyw,1wma,1e81,1he4,1
ky7,1lrw,1fut,1d8d,1w9u,1xny,1ot9,1gi6,1ll9,1z59,1y2w,1ro6,1afl,1eq2,1dlv,
1kxp,1q6k,1pfu,1k0i,1fe8,1hop,1nv8,1i0i,1ubs,1oj9,1m51,1c27,1nfr,1zi4,1ofu
,1opm,1qlb,1tpz,1d4a,1g16,1e9f,1bo7,1lot,1luq,1yra,1oqm,1n6r,1ibr,1f86,1mr
f,1px8,1vwh,1bc5,1aax,1ona,1pr9,1c1u,1oij,1w1a,1c70,1y8j,1ddu,1nwg,1y52,1r
e3,1fff,1djl,1uda,1q5m,1e98,1u0a,1v3v,1m9n,1j0d,1sc6,1mp0,1kjh,1zp8,1jcq,1
srj,1ovk,1mrs,1c1y,1jtq,1utn,1nx9,1i9l,1rai,1pz4,1l6i,1el5,1xtq,1pq6,1zth,
1lb9,1kt4,1rdp,1vpd,1e4y,1w7f,1b5d,1ydr,1lpu,1j1f,1cw4,1ore,1zgs,1zhf,1bwc
,1dr1,1xff,1bty,1ehi,1djn,1d0s,1kta,1pg4,1ie4,1uay,1vso,1ven,1o5g,1cf3,1p8
r,1m5d,1nl7,1jsv,1tjj,1kij,1x7q,1gi1,1pw7,1lj8,1g3m,1zpr,1fwu,1pfk,1qyv,1g
lg,1j84,1npl,1dra,1fbp,1ngf,1tvh,1n8f,1rrg,1hiz,1qbb,1ez9,1fea,1o0f,1i6l,1
q79,1e96,1qjw,1a9m,1gqj,1r7v,1kwc,1bws,1gs4,1ouy,1u9q,1fbo,1x1v,1kar,1h1w,
1n8v,1gvs,1w9w,1a3t,1jdc,1y9r,1ahh,1xdd,1ev3,1vg9,1q3e,1f6d,1is0,1i2g,1dlj
,1c88,1gpu,1gqr,1n6h,1iye,1s68,1q8s,1xhc,1eow,1p60,1k5q,1v11,1k8c,1s1d,1jn
w,1dkq,1gsq,1g3d,1qb7,1kkr,1rkg,1nmk,1z4p,1q6l,1p19,1laf,1mxh,1tt8,1c14,1a
8s,1g4s,1tx0,1b0k,1nli,1u8f,1msv,1u91,1get,1uum,1g1b,1it7,1gis,1ir1,1j14,1
vcj,1ofm,1f3e,1uhk,1zb5,1siv,1cvn,1fp6,1u8u,1bpj,1jyr,1l2i,1yxx,1tqp,1g7f,
1ld9,1fkb,1q45,1ycp,1x06,1wqw,1oip,1ngd,1zjp,1pkf,1qo0,1ii7,1xap,1nja,1s3v
,1eb1,1a0t,1s3z,1xie,1akq,1icv,1i2e,1ml4,1m2t,1foh,1awf,1yec,1ws1,1m4i,1qo
8,1jbp,1uvc,1now,1yye,1lr8,1xha,1ela,1kj8,1lvl,1dvu,1t24,1k4m,1hos,1v03,1k
sl,1jhu,1q72,1jb9,1o4f,1aj2,1n6y,1vj7,1fzc,1ja1,1cr2,1g77,1jil,1l5s,1pzx,1
n9m,1gm7,1jrz,1g94,1pl6,1e8d,1bq4,1ows,1rdj,1rdz,1t74,1qf5,1onx,1nwl,1v6w,
1gpe,1yz3,1ads,1dds,1u8z,1eve,1so2,1qgq,1e3l,1ckn,1jte,1pxj,1jue,1gha,1h47
,1oe0,1d04,1w9v,1c2k,1ojj,1doc,1aed,1jpc,1sw0,1kj1,1tym,1vd3,1rm5,1iut,1is
y,1uhb,1p6x,1bwa,1foi,1gzc,1u26,1b6k,1djw,1gfz,1rd4,1hpx,1j2j,1if8,1xnn,1c
86,1p6n,1q2d,1xpm,1m4g,1mvt,1xid,1b0p,1dgf,1fyd,1xq3,1mo9,1t3r,1kvs,1ek0,1

l5o,1vif,1vij,1eg2,1y7p,1u3w,1bhx,1bjv,1v8k,1h78,1okk,1h16,1rd9,1cka,1q6z,
1of6,1o35,1obb,1kjm,1ghy,1p1j,1aux,1t0n,1fu8,1afb,1hg5,1t7i,1hmt,1stc,1hld
,1aqx,1tlc,1tcx,1o3h,1qq9,1dyi,1okc,1n8u,1jr8,1ryi,1qf4,1ahy,1up7,1zga,1ha
g,1j1b,1zhm,1ovh,1ane,1d1k,1mmx,1js8,1kdp,1zn8,1u3u,1fo0,1llr,1dfo,1jsn,1k
9t,1utt,1x1t,1p6l,1syo,1jh8,1yzt,1nis,1tze,1ra8,1rql,1hew,1uam,1lna,1f9d,1
rt9,1ewj,1g55,1rbp,1q7b,1pez,1nn3,1cj3,1wbo,1eqc,1gyk,1uyx,1c5l,1zh8,1iin,
1j07,1mi5,1a4k,1ywo,1xuh,1lo0,1u0f,1wc0,1i9g,1nsa,1vc9,1ty9,1ig3,1mq4,1qpd
,1fhd,1pl1,1z18,1vid,1th8,1m1b,1rm4,1p4m,1el8,1h81,1ky3,1yc4,1loy,1p4j,1xk
w,1eny,1s3u,1tgy,1igw,1h6h,1zht,1znd,1bgg,1na3,1zs2,1tzc,1f8f,1sz3,1t1y,1v
fr,1jdf,1avn,1qm5,1h2z,1c5w,1pkj,1hon,1feb,1nas,1yve,1gp4,1c1t,1j4h,1c3m,1
cer,1ion,1o6l,1odw,1skg,1jcx,1yhl,1ai4,1q1z,1lan,1az8,1g0h,1xin,1oc5,1zba,
1m05,1pyx,1jmf,1kk2,1c1x,1azl,1ruk,1xmn,1k7t,1hk4,1i06,1nhe,1iuw,1oix,1ecj
,1fv0,1fqa,1u21,1byd,1jvx,1w3t,1nsm,1uw6,1fm8,1r34,1uu6,1f1v,1z3v,1u1d,1v8
l,1ahi,1k63,1p72,1dxo,1cnq,1as0,1gxz,1nyt,1n0w,1gz8,1kor,1yek,1w0w,1poc,1m
5e,1sua,1nc1,1nb9,1h4w,1kjq,1vzt,1l1q,1pxb,1s0x,1tog,1k4y,1bn3,1woo,1y5m,1
zhx,1pt8,1i10,1jxz,1hzj,1gi7,1jsz,1nmm,1kwv,1dzt,1fdt,1rdq,1jtv,1ukw,1wvj,
1cg6,1q8u,1bqi,1vft,1u3f,1w6k,1mu8,1bju,1xh6,1tx2,1oo6,1kay,1rah,1duv,1kfb
,1mq6,1h17,1n6o,1uu3,1jnr,1vi2,1q7a,1bu4,1z2n,1zzr,1qv6,1g3e,1r6w,1qj6,1i9
0,1u8m,1glp,1qr6,1xjb,1fk8,1ec9,1pek,1lbc,1q0y,1ly4,1vje,1w6f,1lt5,1jr0,1m
6o,1kvu,1w8l,1c29,1kkp,1ba0,1mx1,1wt1,1igj,1p43,1c2d,1uzy,1f7q,1kep,1kv1,1
q4k,1inn,1a5u,1f9p,1o2u,1o38,1rwb,1slb,1go2,1cwn,1m5w,1pxp,1qco,1iyk,1ump,
1o33,1lo3,1kck,1ppi,1fgc,1uxv,1lp9,1msk,1mdc,1aj7,1tni,1re0,1k2y,1v2a,1oky
,1w11,1q1a,1flj,1ls6,1f7n,1sl9,1zgb,1rgc,1ky2,1do8,1fl2,1e5o,1qpj,1qh9,1yn
7,1enc,1pi3,1lnd,1aeh,1wno,1kqb,1dtu,1fwe,1nhw,1xjn,1v7z,1b3z,1oyj,1j4i,1y
xu,1m8g,1w7h,1ru2,1w0p,1oba,1qjb,1f3p,1g5t,1w2t,1c1s,1ker,1xrj,1aog,1h82,1
k4g,1lri,1tb3,1owh,1nup,1sm4,1omo,1iah,1wuy,1sql,1o3o,1dr2,1hnd,1nhr,1kt3,
1uux,1tom,1mog,1ukq,1x7h,1eqm,1r82,1giu,1isw,1wtg,1pfv,1k06,1cpi,1h2m,1byc
,1if6,1mww,1wyv,1vzc,1d0c,1mxf,1y4c,1bzy,1i59,1xur,1ii6,1i3h,1ohb,1qsg,1g4
9,1v8v,1dai,1tzj,1eyz,1x2r,1pwb,1uz0,1drw,1thn,1eed,1wc7,1v0o,1wk9,1i7p,1p
0f,1met,1oqc,1spa,1v6u,1y2x,1l1r,1p9b,1fw9,1vj5,1yro,1u9e,1ork,1we2,1o2b,1
vyr,1h5v,1o6z,1azt,1a5s,1m2x,1ng1,1yhm,1vfw,1fnb,1d0o,1low,1hck,1jl0,1ulm,
1say,1z6j,1kza,1v3h,1mwm,1xcl,1zvr,1hur,1dod,1xd0,1yzg,1niu,1z3n,1fkn,1nzy
,1m01,1uyg,1fpd,1q8j,1dc6,1v7r,1xli,1th6,1s5z,1jjt,1nq2,1ije,1nok,1joc,1t3
d,1rkh,1xva,1w8d,1l6o,1beu,1p0b,1n2j,1wvg,1eft,1hkd,1dp2,1lpg,1j8v,1e64,1o
42,1j3k,1knr,1bch,1nq5,1md4,1tr7,1fpq,1xl5,1x98,1re8,1led,1rje,1apw,1q6m,1
mw3,1epn,1e9h,1c5q,1lv0,1vko,1pkk,1c3i,1qdc,1t7j,1r81,1mv9,1q21,1wav,1jqy,
1ofo,1mts,1wb4,1awq,1dmt,1lor,1qvj,1kao,1bma,1o87,1u3d,1y57,1mq5,1z32,1ej4
,1tn8,1llu,1dfi,1wn6,1zpd,1s7y,1v1a,1so6,1o4q,1mqo,1u3s,1ez2,1ebl,1z95,1gy
8,1inv,1czi,1z11,1jw5,1ns4,1nq0,1hsz,1bn4,1fk1,1xg4,1ol5,1lhv,1gzf,1mud,1v
kg,1ufy,1v8r,1bkm,1t1s,1yvf,1cm8,1zl9,1jf7,1cib,1ule,1y65,1q0z,1tkj,1hvl,1
mm7,1dnl,1nlm,1fr8,1chm,1dkx,1p0m,1if7,1w6j,1v6x,1thl,1yrx,1s7w,1lb8,1f8a,
1my3,1us0,1kti,1qut,1qxo,1f74,1x97,1p1r,1jbw,1ej1,1n46,1o37,1e5q,1q92,1jpj
,1bg0,1nh6,1jnz,1uk8,1ebk,1wp4,1o98,1umi,1oc7,1jgi,1zs6,1fwv,1t57,1xuf,1e7
e,1aqy,1y6r,1z6z,1e7w,1s9w,1yc3,1ke6,1lol,1nki,1s19,1ogi,1m4d,1k8y,1p6e,1t
79,1n2s,1mp3,1y13,1ps9,1q97,1xh9,1za1,1x8b,1t0a,1gsu,1hex,1l5v,1fpe,1b1c,1
o3y,1if9,1agc,1ql6,1s3x,1e2m,1ytg,1zgc,1wbt,1w2c,1bxm,1pz5,1gpy,1bcu,1jg1,
1fbw,1wd4,1dgm,1z2i,1xex,1amu,1qk4,1ofe,1u1w,1vbi,1byq,1n20,1e3s,1j3i,1ky6
,1pl8,1lpz,1ocl,1a2c,1nes,1ecv,1sg9,1y6b,1yth,1utj,1ohr,1fmj,1a2u,1e5a,1e0
s,1nec,1u0w,1qlt,1wk8,1ga8,1tqv,1j1n,1ewy,1a8a,1ki4,1gr0,1mtr,1qbo,1e8w,1p
3d,1s07,1xon,1unl,1qkn,1mzv,1gi0,1zcw,1wpy,1oyc,1v9a,1ftk,1r8b,1nqx,1dz9,1
sr9,1ici,1c1d,1nt1,1gpm,1x8t,1xz3,1n6a,1hq5,1i8a,1atr,1d0l,1elw,1kgt,1nmu,
1on6,1t93,1zmt,1ljn,1z4r,1t2b,1t3t,1s5m,1nfu,1p31,1qop,1w4p,1fwt,1uyy,1lk2
,1gz6,1t8u,1i6x,1t2d,1s9t,1i3i,1g8s,1nxj,1p84,1ubx,1ppl,1o5b,1dmj,1brw,1q6
r,1trd,1ahf,1czm,1sw7,1z1r,1c9k,1fys,1msn,1yqd,1cqs,1v5e,1n5r,1tjk,1g1a,1e

ls,1guf,1l9d,1lo2,1sth,1okn,1nuy,1x29,1zvx,1rbq,1raa,1z41,1s0i,1xii,1usb,1
jg9,1ywr,1xah,1zha,1ldo,1yrc,1phh,1ro8,1vcz,1g48,1uyz,1aeg,1oj4,1sqs,1mre,
1q6j,1rpk,1fww,1hsl,1rx1,1bdm,1h63,1ltt,1e7g,1uoz,1kwk,1tzk,1e4h,1zpa,1nq7
,1gsn,1ahx,1l84,1geu,1mrd,1p1u,1sd3,1q3p,1bnq,1rp8,1bag,1g5n,1oaa,1j2g,1it
8,1ugy,1bqo,1q20,1obo,1a9r,1qgy,1mvq,1cp6,1m03,1mmy,1e3e,1wds,1v8b,1hy3,1q
9d,1q0r,1c3q,1jcy,1ua7,1b8a,1e2q,1k8d,1bmk,1w8m,1hxf,1nan,1m6w,1grp,1coy,1
mld,1fc4,1drv,1tbb,1lld,1q4j,1ht0,1zsr,1z8p,1mqw,1b5t,1npx,1m2w,1qmh,1ybu,
1khh,1aic,1v1j,1m0o,1tj2,1rcx,1akt,1upf,1rs2,1my8,1znj,1z4k,1fta,1xh0,1ld7
,1yvd,1ofh,1kqr,1z6k,1vg0,1v47,1tw3,1cxz,1y91,1csn,1p0p,1owy,1yik,1tyu,1wv
1,1oiu,1zlf,1ih7,1d00,1ki6,1c82,1cb7,1h86,1yz0,1loq,1evi,1tdi,1k2b,1wnz,1i
g1,1lo7,1bv9,1dmp,1tbf,1frn,1mjb,1gyx,1b0d,1c7e,1mw1,1afs,1lzx,1ini,1czy,1
nd2,1o31,1nf0,1yr8,1jgt,1fk2,1r1s,1hms,1idt,1r1p,1sla,1vzx,1to9,1hiv,1i3f,
1l8b,1m7g,1hox,1kbq,1mex,1e7f,1y2v,1mf4,1mw0,1p7w,1fxh,1ojo,1bu5,1if4,1eli
,1cen,1tlg,1snm,1bii,1ett,1e4i,1qi3,1e7v,1gsi,1sb8,1kyw,1d1w,1ai2,1p2h,1t5
b,1qr2,1fgy,1ylr,1cc6,1uzv,1tyl,1t9f,1lfd,1rnc,1q22,1bqp,1qz5,1mzs,1on3,1m
p4,1lsp,1od8,1nx0,1gca,1b6l,1pj7,1qhp,1g9c,1v93,1ps6,1m7p,1snf,1cbx,1jyw,1
gc5,1is2,1tvb,1km0,1wbz,1w6r,1pt2,1jir,1sdu,1h41,1zoy,1xxs,1ksk,1eom,1hw8,
1ft7,1cc2,1w1u,1ggn,1ipf,1mpw,1pxi,1qpk,1qgd,1ep3,1t0m,1sta,1fkh,1fhx,1mi3
,1jqv,1srf,1ye4,1i7i,1pq9,1wbe,1nnp,1xjq,1f0t,1z9a,1i4d,1a1m,1sww,1hyz,1d4
j,1rjy,1c9u,1um5,1kn4,1zk1,1obd,1fy5,1n6l,1ohs,1woq,1x09,1nzl,1hfq,1ri3,1c
bq,1rwq,1hsj,1czu,1h1t,1zdp,1vf3,1a3k,1axw,1vl0,1l7x,1tsl,1a26,1no6,1ep2,1
gbn,1xm1,1py5,1obg,1ut6,1vot,1l0l,1mfi,1b09,1rx0,1anf,1n2a,1mra,1onz,1gqh,
1vwj,1xh8,1dud,1v2x,1e63,1w1s,1ukv,1aer,1hvk,1lzr,1ocq,1qds,1oxp,1q8q,1qw4
,1g9j,1bhn,1nwu,1f8r,1fk4,1n55,1xym,1xwq,1u3l,1rri,1sd1,1oi9,1o8s,1zrh,1bx
q,1n4h,1bkw,1wbi,1e99,1uzu,1qg2,1tpv,1px4,1h7g,1dgg,1rmt,1ia9,1yuk,1o39,1o
mz,1cjc,1g35,1zs0,1c84,1lti,1z71,1dvx,1dbp,1vqw,1jy8,1e4n,1s1a,1tyw,1cx9,1
ox5,1sn5,1a6w,1gwq,1rf6,1eag,1p48,1ivp,1hfs,1grf,1kt5,1ojk,1wu6,1v4b,1z89,
1p52,1mgo,1yim,1ex8,1hhj,1s2i,1s38,1gcz,1gua,1ijr,1nss,1aqu,1age,1j9e,1yob
,1i75,1yqj,1jyv,1xoc,1mdr,1hw5,1yvn,1kli,1tiq,1zwp,1noi,1mvc,1q4w,1oit,181
l,1rc1,1w5s,1deh,1pr6,1uxw,1os1,1qi4,1qb6,1adl,1m9m,1g51,1rg9,1bxk,1sz6,1o
2h,1y5w,1zef,1sxk,1yiy,1qcf,1g42,1crb,1qo3,1l6f,1zab,1e2l,1i7l,1vf2,1xrk,1
ahg,1cbf,1g9b,1sqn,1p18,1rup,1ded,1dih,1xr8,1i8z,1qxz,1z3w,1kpf,1h8i,1tf9,
1s16,1hmr,1e8m,1jf1,1ktc,1uz9,1ww4,1kss,1dr3,1xpj,1g98,1af7,1qw6,1tg4,1gxu
,1ywq,1k5s,1o3i,1kmq,1ama,1lf9,1uby,1ayl,1h8d,1dhf,1mdb,1kjr,1nhq,1wcb,1gn
q,1jlx,1hdc,1lrl,1tt2,1i1l,1e40,1o5m,1va5,1w4o,1jw6,1sst,1k2t,1jp4,1a50,1e
7a,1ohe,1hii,1yp9,1ag9,1agm,1ckm,1jef,1qgz,1tsq,1tl2,1n6i,1t0i,1rae,1twx,1
xom,1hlk,1u1b,1h3n,1tz8,1h85,1ajx,1jiu,1cy2,1hef,1ifu,1h1q,1q6y,1k0l,1ow4,
1gzq,1eib,1t85,1tow,1yyn,1lin,1ipb,1o04,1iki,1wor,1y2f,1xzx,1uyr,1nox,1o3e
,1ry8,1wva,1esq,1d4x,1ejh,1v2f,1sbg,1xmj,1kfg,1fwk,1n2h,1n5t,1c7s,1c81,1nh
z,1qj7,1lqu,1stb,1awi,1hah,1rzx,1p1m,1tvu,1d7i,1kic,1xt6,1njr,1ho5,1drh,1h
ot,1bof,1qkq,1wxi,1qnu,1xic,1tp2,1mue,1ehh,1ui0,1kn2,1xms,1f7r,1o5e,1e1k,1
jla,1jah,1kfe,1v35,1jh7,1yid,1pzi,1w4a,1og1,1bgu,1uy7,1geg,1bif,1qbs,1bj5,
1h94,1zfp,1c3e,1o2p,1gv0,1eap,1tkh,1kdt,1uj6,1xs5,1hvj,1bxg,1k0a,1ydk,1j36
,1u7h,1gz5,1daj,1sow,1ua3,1lck,1lag,1fcz,1d4s,1geb,1c22,1tkg,1c4q,1fkd,1ng
h,1ykf,1vwd,1uka,1p6i,1t41,1qis,1pzk,1z10,1rdm,1h2j,1hnh,1epp,1msa,1c1c,1u
cn,1sp5,1zog,1xuz,1tlm,1ur1,1mmp,1t7r,1m7z,1bl6,1i5b,1dg8,1z6d,1leg,1wbw,1
jgu,1p2d,1daw,1qy2,1xt3,1ez0,1yqx,1ke9,1d4d,1meh,1aek,1q5d,1mrw,1epz,1pty,
1oj1,1t25,1ke5,1jvd,1kzd,1a7c,1rth,1dd7,1fem,1g0v,1z0a,1cmc,1xmi,1q6q,1s8d
,1csh,1zzq,1zaf,1wvc,1pz1,1mrn,1ex7,1mbq,1pj8,1a95,1hyb,1l9k,1oni,1ubw,1mf
g,1xby,1uxs,1e2g,1bv7,1ktw,1hxa,1q13,1qhr,1w48,1f73,1qbn,1fl3,1ozf,1kyy,1w
pg,1kmv,1etr,1p8f,1jkk,1j0i,187l,1vep,1sux,1s5e,1m78,1o6i,1uwl,1knu,1fxf,1
gt5,1tr5,1km3,1rz0,1mdq,1x01,1rds,1k3u,1e82,1ezq,1aq2,1tsd,1w0y,1qin,1xp1,
1k6p,1yeg,1zt1,1kwu,1oyf,1z6s,1yxv,1ciz,1xvv,1m0q,1w29,1qfy,1ur0,1got,1qgx
,1kf0,1o7q,1qh5,1b6p,1jq5,1mdo,1rdl,1r4s,1sc9,1osf,1v84,1hv9,1bnv,1v9t,1l4

l,1oke,1ukt,1ia7,1ngi,1h0i,1lmc,1bg2,1sep,1dq8,1p6c,1ptv,1u5a,1mg0,1q11,1u
u5,1m2z,1x8x,1aaq,1o46,1ceb,1e5d,1c4v,1xtt,1d3h,1ulf,1i00,1w13,1axg,1stg,1
tmn,1azm,1i0s,1fwn,1jom,1oo5,1n2e,1xug,1fec,1tw1,1upv,1i9q,1xh4,1g45,1tk4,
1qye,1rpn,1n1d,1wdq,1c80,1qg6,1apb,1vfs,1uy0,1c3n,1oga,1tx7,1ybm,1kp2,1fgi
,1x07,1gm9,1xyv,1q3q,1y3v,1zeo,1pso,1d7r,1bmb,1dfg,1eeh,1jn4,1tv8,1eey,1od
j,1syi,1l3i,1i52,1z8q,1jdx,1ptw,1mmv,1pwl,1nrs,1v4s,1v6i,1z2l,1yin,1xcm,1n
08,1pg3,1qwc,1jk3,1zw5,1m7d,1xpl,1oon,1mfe,1ey3,1fy7,1lnf,1qw8,1aeq,1o28,1
rlu,1j53,1t7o,1rj9,1dqe,1iq7,1gai,1q3d,1giq,1e1v,1zhs,16pk,1ur2,1toq,1n23,
1k4w,1e2p,1cgv,1kak,1eyn,1exm,1sqo,1eou,1kdk,1g54,1itc,1ou4,1zap,1ovd,1ocn
,1s1r,1fcb,1owb,1itu,1nux,11bg,1daa,1ru1,1fj6,1pw1,1b0g,1rsd,1x9h,1uld,1gi
j,1r7u,1qfm,1s1j,1vp5,1nrj,1s5s,1y0b,1ket,1lee,1upw,1bwv,1vwq,1e54,1ncq,1k
7f,1svz,1s39,1rjn,1o32,1h7q,1aku,1rac,1pn4,1l8n,1q2o,1hsh,1ta6,1vp9,1ejb,1
rm0,1zyx,1y2k,1sz2,1wzy,1afq,1k27,1p42,1z2o,1t5c,1cl0,1abe,1up3,1cxv,1yqs,
1rdt,1xef,1qhc,1cet,1u3g,1k2u,1vfz,1osv,1ojn,1ycj,1edo,1ww5,1qf0,1s14,1kz8
,1g93,1c3r,1gx8,1nsc,1ff0,1x2a,1iyi,1s7n,1ny0,1j1z,1r66,1o3g,1d4p,1iei,1z8
3,1r5y,1yjy,1que,1xyb,1khz,1bh2,1opj,1c2h,1c3x,1g76,1ev6,1w6u,1aky,1zem,1i
5c,1f3b,1jlr,1v1k,1b3v,1ldm,1a94,1pdh,1mjj,1be4,1ukg,1a0g,1cq1,1jgs,1krm,1
zvq,1mmz,1g86,1d5c,1mc1,1mn7,1yzx,1ee0,1om5,1mer,1wei,1q94,1nm1,1nzf,1f5v,
1xj0,1bf3,1odx,1cy0,1nl2,1s9p,1p6d,1fhv,1e20,1d03,1toi,1ykr,1tyr,1bq6,1nfw
,1t46,1oh0,1lj1,1t48,1e26,1v3i,1j1c,1h1r,1uo5,1pke,1uu9,1loo,1k0d,1yre,1a0
5,1m48,1ykj,1rjk,1b9v,1ebw,1t9c,1lkl,1af6,1ve3,1lqe,1fiv,1poy,1jmg,1jmh,1p
6j,1ndj,1v9n,1cbk,1xif,1kcd,1ah4,1ny2,1fp1,1xlj,1ozq,1eiz,1gtz,1lcp,1i9p,1
exb,1wb0,1kt8,1gyy,1f8d,1wv0,1pkw,1c9w,1ev4,1t96,1tu3,1swd,1kcc,1ve9,1if2,
1mly,1i0x,1k4v,1frw,1zvl,1lgw,1jhm,1nmx,1e6n,1o69,1kvl,1hdz,18gs,1b7a,1km4
,1dy3,1v7a,1pzh,1cwu,1rst,1t7f,1ij8,1rz1,1lwo,1fkl,1a80,1o76,1cul,1c0k,1pb
o,1vrt,1o2i,1jjb,1ru9,1mb4,1ixn,1hyu,1jsi,1inq,1sri,1ejn,1lqp,1ecc,1ynd,1b
14,1ohp,1ldg,1w3r,1y59,1fpi,1vdc,1jra,1yuc,1uqz,1jny,1wkr,1lf2,1jix,1uy6,1
d4o,1hvb,1nje,1hhi,1ffr,1k97,1n43,1l7z,1k21,1esw,1x14,1jry,1q9i,1x1r,1b2l,
1tfj,1g0c,1gte,1gd1,1uc2,1px0,1gir,1y6a,1z57,1qkj,11ba,1eam,1art,1qqo,1hwk
,1o6u,1pgn,1rgl,1uz4,1t22,1dad,1e8g,1iqu,1qte,1hg1,1bjg,1my2,1y3y,1lpd,1o4
j,1d7u,1mmu,1nc9,1xpk,1sjc,1kzl,1reo,1xw5,1h66,1u6q,1wub,1oty,1ax1,1slc,1k
a1,1lru,1ypj,1w5v,1vjy,1vp3,1u08,1rsz,1e8u,1pu9,1hi3,1dqa,1pbe,1xuk,1egy,1
ydb,1czo,1e1t,1n8i,1ppc,1iv2,1qzy,1nmb,1hvi,1o4o,1htg,1lh0,1w9b,1fc0,1iux,
1rjd,1fkj,1mj3,1req,1of2,1mp8,1onp,1owl,1fg6,1d3p,1fue,1siq,1e8n,1f8w,1e7s
,1o26,1p1i,1oz0,1rzy,1fh9,1hoc,1zdw,1o2w,1x7r,1zin,1kzn,1odz,1obv,1cg4,1md
t,1q4v,1xr1,1ual,1w0z,1wt3,1uip,1mvn,1ai7,1a6v,1ngc,1m5c,1isi,1f7a,1bp0,1a
i5,1q6c,1l8g,1tpb,1qr3,1g0r,1hfc,1cdg,1v0n,1z0u,1zyj,1cy7,1rye,1xz1,1ae8,1
qft,1jho,1c87,1yjz,1w3k,1cb0,1kv5,1lwj,1in4,1zx1,1tdv,1w5t,1kh2,1tmm,1dcp,
1zco,1ojm,1n2n,1mt7,1xnz,1rmz,1h9a,1htw,1sqt,1ktt,1in6,1zeh,1r78,1itz,1lic
,1xoz,1m3u,1mj9,1cyd,1uva,1rhl,1vht,1gsj,1el3,1a3u,1vem,1y30,1u9n,1h3f,1dg
b,1tj0,1ep1,1n6x,1fth,1o5q,1a5z,1ih8,1wbg,1of1,1y20,1ogd,1szj,1ank,1k1o,1m
tb,1dm8,1ng4,1nhu,1ec3,1lzy,1svm,1f20,1inh,1lzs,1fh7,1c3v,1rao,1s3w,1cdk,1
i1n,1a4r,1uk6,1xr9,1xc6,1rx7,1cj4,1j8a,1xmv,1lde,1uma,1y2g,1yzn,1lcv,1mtc,
1qll,1kfc,1g5f,1t4v,1khb,1xqx,1mes,1uk9,1dm6,1lc7,1rne,1rx9,1kt6,1zg3,1r0p
,1jot,1jyx,1r4u,1ah3,1aqb,1qhf,1ndh,1pot,1rx2,1qg0,1utm,1t7t,1tzd,1nmz,1ns
g,1h7l,1d8f,1ewf,1jq8,1zj0,1fpu,1s5k,1hzz,1bgv,1ya3,1nfs,1td7,1tdu,1o86,1k
m5,1p3j,1wn3,1fo3,1a9e,1o48,1w3o,1z5o,1vgr,1g69,1upj,1ps3,1tvy,1h7x,1ai3,1
fk7,1fsa,1c0f,1a07,1lov,1d1v,1u7z,1eb9,1b42,1j15,1f0s,1rv1,1vyz,1j7l,1xs1,
1lqt,1rsc,1g9f,1rp9,1duc,1nd0,1hsk,1fdk,1i58,1kbn,1e6q,1q8w,1ngb,1s4m,1hnj
,1nfy,1kew,1uz8,1zz1,1uf5,1lqf,1o94,1rgk,1iiu,1h22,1d2a,1sg0,1h1m,1z4l,1fz
q,1wbj,1m2q,1zc9,1eld,1ep9,1qbg,1bap,1wdt,1lii,1pzy,1cq7,1vwf,1g97,1cnx,1c
a7,1ei1,1tug,1ow3,1ho3,1n1p,1lte,1kuj,1b15,1dwk,1tti,1fcx,1oho,1ha3,1f6b,1
c0g,1kwr,1mkv,1trq,1ct1,1gg5,1kjl,1pie,1o30,1axe,1qfl,1z5c,1mjh,1e6r,1lp4,
1zgy,1gye,1mos,1ypl,1owm,1f3l,1toj,1sjw,1exa,1bd4,1a99,1i3k,1il0,1zj7,1o6y

,1cmp,1z2p,1hbk,1jki,1sff,1h6d,1jhp,1sg4,1h18,1lvu,1xi9,1p4n,1u0z,1tv5,1u6
s,1bo4,1wby,1is3,1vzb,1a27,1kts,1grt,1ry2,1n49,1h0g,1ukb,1ove,1xf1,1nl5,1a
gp,1yxq,1ri1,1mdu,1pig,1q43,1gnm,1r7t,1n5m,1esz,1suo,1xyc,1o8b,1ruv,1hgx,1
xws,1tsx,1w6p,1k6v,1rxj,1nst,1d7j,1eb2,1pmn,1bq1,1c2e,1ib0,1zch,1fbl,1wc1,
1o2k,1nuq,1cxi,1gx4,1nr5,1r5g,1y38,1tzm,1m6i,1sw1,1ua4,1uou,1rdk,1h9g,1h7t
,1m8j,1v9o,1mc9,1vb3,1twf,1eqa,1in7,1ay5,1s2d,1rr2,1f1w,1n1i,1pr2,1t10,1oi
y,1eyq,1ytm,1vpe,1obl,1pgp,1n5k,1x92,1ezv,1qxa,1ma9,1xdh,1w5x,1lo8,1yqz,1t
ag,1uae,1ixi,1fy6,1ad8,1g4j,1k2c,1zgi,1z5m,1yp6,1o34,1k5n,1xnj,1g9d,1ms9,1
uze,1a30,1uk7,1euu,1ww2,1b7y,1umz,1z07,1c9y,1t21,1s8a,1mr2,1s1f,1ben,1sve,
1m7o,1yb7,1se3,1yvx,1l9w,1q1y,1hpg,1jyl,1ngj,1ysl,1eko,1vkf,1e6e,1ynk,1xqd
,1rdw,1kr3,1wle,1gp2,1jq3,1lk7,1ocb,1w7g,1hbp,1y3p,1f9w,1ikg,1p5e,1unh,1mp
d,1gz3,1f7l,1uom,1h42,1fzj,1zua,1i37,1tk2,1rl9,1jhg,1lsh,1cly,1e06,1v2q,1u
18,1fj9,1dkp,1emd,1t5d,1ghq,1ky8,1tky,1k72,1o4g,1ztq,1w0v,1t7q,1dz6,1u3v,1
dak,1gjw,1sx6,1fbh,1k1l,1x70,1txz,1jgm,1qci,1yjq,1xep,1lbx,1kzb,1xfg,1al6,
1h23,1b39,1ldn,1nav,1pxn,1hrn,1lhu,1b4p,1you,1jz5,1tqt,1rco,1asc,1g9r,1ke7
,1bgq,1bd2,1npd,1mtv,1rn8,1lzz,1o9d,1mma,1r87,1re9,1mpj,1xw4,1evk,1abo,1m7
e,1m2r,1m75,1zfj,1hdo,1jbu,1ax0,1tox,1nm9,1mmw,1dlr,1g78,1yxi,1mk5,1pzf,1m
mr,1e3r,1av5,1d6v,1fnn,1rq2,1a0f,1d6s,1swp,1rx5,1gi9,1uj5,1kgq,1v5f,1t6x,1
eno,1ybq,1u90,1qha,1i3n,1c5n,1iic,1o3j,1lop,1yc5,1eqj,1dr5,1d1x,1t7d,1td2,
1hhk,1rum,1heg,1o3l,1ba1,1e1y,1nd4,1dam,1l2e,1v25,1icn,1mxt,1y6o,1wtc,1dkz
,1u27,1f0y,1ytv,1h1p,1r51,1x54,1f9a,1fg8,1vjd,1wbp,1opr,1i05,1bzh,1i0b,1dr
u,1kzc,1x87,1ckp,1gwl,1xow,1ee2,1m2h,1t4f,1xl1,1cea,1okm,1g99,1e2j,1rag,1e
cq,1agw,1vzd,1uy3,1wnt,1fby,1w8s,1vhd,1x3n,1h5r,1o02,1dhr,1h24,1g1l,1heu,1
m2p,1hyf,1cxk,1pzl,1dl5,1jtu,1tsy,1jie,1w9x,1uy9,1fln,1o5r,1fds,1kol,1n0s,
1mn0,1a69,1f4p,1u1j,1pi5,1rbm,1c9d,1clu,1rdy,1gim,1z4x,1n0u,1x28,1hvq,1k4h
,1h50,1x2b,1se4,1dia,1gym,1d4i,1n5s,1nhk,1cq8,1y8o,1d9i,1gck,1aem,1uwc,1zk
0,1rlz,1jht,1pzm,1jvg,1b8o,1gjd,1vay,1wb5,1h62,1w0x,1bob,1ygc,1tvp,1mrk,1p
57,1zdq,1owz,1tid,1d4y,1bb5,1xbb,1akv,1bvi,1pym,1xc7,1obc,1yee,1c3s,1fm4,1
usn,1v1m,1pg2,1i5z,1ttj,1k2r,1wbu,1jqx,1did,1iy8,1p0e,1ynh,1jt1,1c2j,1sgu,
1z5n,1c1w,1wht,1o6b,1kht,11gs,1xqh,1yej,1frb,1xjj,1xse,1ouw,1kpm,1ftq,1lie
,1s5p,1ajv,1uu1,1nu2,1xuc,1y2u,1zg8,1qpr,1v3m,1meu,1ws4,1yjs,1cps,1agd,1pn
l,1mfb,1pr0,1jak,1d7o,1u3t,1uj2,1amk,1ra2,1fj4,1e24,1l4y,1j10,1pj3,1seh,1a
pt,1rx3,1sty,1g27,1f9v,1shb,1a8g,1o3d,1n1m,1e3v,1rmy,1hm8,1fee,1yyz,1qi5,1
s5d,1i13,1zd9,1yyr,1hp5,1e00,1ahb,1mqq,1lxk,1ww3,1gny,1vhz,1z16,1ho4,1kqy,
1txr,1vpo,1akc,1noj,1ck6,1mar,1ozp,1h1s,1gad,1fk5,1x1z,1oe7,1lph,1f52,1sx3
,1mrj,1ot6,1a59,1ror,1oh4,1te2,1wxe,1sqk,1qb9,1e2i,1h51,1x8l,1pxo,1wrr,1z0
f,1qor,1fk0,1a0j,1mqd,1zh0,1u4d,1a4m,1u93,1b0j,1ptr,1f7k,1of8,1gz4,1hdi,1y
b6,1u8j,1pu7,1awu,1enm,1izo,1tvo,1a49,1mfd,1qip,1j6x,1of4,1kww,1z2a,1shd,1
h0r,1ek5,1svi,1e9c,1xgi,1cel,1n6c,1r16,1i72,1rp4,1klk,1glq,1xgj,1fn8,1zhj,
1kp3,1pvd,1g41,1epq,1k7u,1mnc,1aqw,1o9p,1d3g,1mwh,1s5n,1e9e,1umk,1aob,1ad4
,1i7m,1kux,1g8m,1sja,1u4m,1l5w,12gs,1fx9,1fao,1g3b,1kpv,1jg3,1np7,1xvt,1fu
q,1mrh,1rtw,1muq,1yei,1g74,1dv2,1lcf,1hf6,1ipc,1vj9,1uoo,1dej,1i6i,1v5g,18
6l,1v2t,1c24,1hpm,1j0y,1hsb,1x78,1cra,1snu,1ajq,1a1c,1k2v,1e2n,1z7j,1mmg,1
dz4,1y5a,1c3j,1raf,1elc,1hk8,1m2k,1me7,1dmi,1j78,1i3l,1jdd,1r1h,1bcr,1c6y,
1f4e,1hqp,1i7r,1wmz,1xij,1ado,1uqx,1bra,1oq5,1l7h,1evl,1p5r,1vai,1jpn,1nfq
,1qbr,1qpf,1b0u,1cnw,1h60,1b9t,1biw,1pl2,1i36,1n2x,1w5a,1f9t,1wy7,1q6d,1wu
n,1e9x,1ogj,1aj0,1hvr,1hn4,1ay8,1nco,1gno,1l1y,1e80,1w1v,1ty8,1hv6,1n3p,1d
if,1gil,1t66,1klz,1cw2,1daz,1jls,1a53,1zb6,1d2n,1fs5,1u29,1f9h,1pdz,1rge,1
fmc,1er8,1e71,1de5,1y3n,1qkt,1oqn,1i38,1xka,1fmo,1gwv,1gnn,1tqm,1ajs,1tzf,
1rl4,1o5o,1ryw,1gui,1nwt,1uxt,1e22,1our,1rqe,1r6b,1lgx,1tn7,1i9z,1wuq,1v2m
,1wpc,1hx0,1r0c,1mvl,1ss4,1m49,1njc,1od6,1o4r,1pn0,1i9n,1fur,1zcb,1ckb,1yp
m,1xf0,1k1y,1ta4,1tb4,1a8k,1arg,1dl7,1npt,1tj9,1qz6,1aq1,1vom,1w5b,1d2e,1o
gz,1q51,1j51,1od1,1oe8,1upr,1v0p,1dqr,1elu,1o6f,1w35,1a71,1tgm,1qgi,1hyv,1
fen,1jg6,1aoe,1b8u,1saz,1okv,1gvq,1uoq,1wxg,1nml,1djq,1m9t,1ndv,1lhw,1a08,

1y5v,1m3z,1zp9,1r2q,1ajn,1t49,1xcx,1ddg,1up2,1mrx,1kk1,1kzo,1sld,1kxh,1wbv
,1sv3,1e9d,1cza,1dfh,1oxo,1hdx,1xyy,1ddt,1dvj,1a8i,1qf1,1kvk,1bb7,1x89,1bm
2,1uqy,1ua5,10gs,1efv,1wms,1tke,1x8i,1q8y,1q4g,1gj8,1p4a,1huq,1f1b,1b8y,1e
fi,1det,1zgj,1u33,1n06,1arh,1qfz,1zjo,1fmw,1mi4,1tkb,1j91,1zp5,1m2o,1d4l,1
aiq,1qbu,1v39,1mg5,1li4,1r6t,1m9a,1fqb,1qsm,1wsb,1r9o,1jeh,1yoa,1q6t,1fzu,
1ofa,1g30,1ga2,1vyp,1upt,1p5g,1l5f,1n1e,1zc4,1k9y,1bkc,1yfz,1pxh,1uib,1lzc
,1uy2,1yet,1u2y,1nsv,1pow,1pn9,1z6p,1t9b,1p2g,1yvm,1lcw,1jjv,1xge,1fh8,1kp
g,1qr0,1xl8,1dpo,1kuv,1nue,1xb0,1g7g,1vmk,1qy8,1ics,1tvv,1ebf,1tll,1ksn,1v
bs,1r6u,1i30,13pk,1ges,1aeu,1l8t,1pob,1ax2,1rkp,1d3d,1olt,1xk9,1i9o,1o97,1
of0,1o2v,1s22,1mai,1iim,1qfo,1dww,1akw,1uca,1owp,1o0r,1npv,1c8v,1so0,1rm3,
1dg7,1jep,1nvt,1xl0,1j0z,1o6g,1v3s,1wbx,1wxy,1afk,1bx4,1vgv,1pgo,1jbv,1gwj
,1h9o,1x55,1gvx,1e73,1rs6,1ne7,1oyt,1vrp,1kph,1kms,1ydd,1ml3,1e48,1uw1,1q6
f,1f2o,1nn0,1oir,1u70,1lce,1bl4,1g6c,1b4d,1o4n,1jd0,1ctu,1e4e,1tha,1gdd,1i
jh,1y1z,1sjd,1w1q,1fnz,1z6e,1kc7,1v2p,1g9a,1is4,1kcl,1b3d,1cip,1bfn,1ghv,1
oj7,1ppw,1lrt,1z6t,1h7h,1zo9,1bkj,1rad,1r1q,1o23,1jhr,1j21,1udy,1z06,1v00,
1n5l,1mdx,1gal,1pa9,1c1e,1w1t,1c1q,1md3,1poe,1swn,1ehn,1tw4,1ly3,1ffu,1iyd
,1z0s,1x8j,1fqc,1uyi,1vrq,1j8r,1jdv,1xui,1np0,1up0,1d5z,1l0g,1m7b,1fws,1gu
1,1f3t,1pxa,1qir,1uz1,1n4u,1qk2,1d6h,1kvr,1ixo,1tc2,1ye6,1ouk,1vbh,1n5q,1z
82,1x1i,1mmb,1dmb,1aen,1fa2,1sn0,1dae,1kr0,1ej0,1z5l,1hfu,1e02,1iyb,1gv4,1
y6q,1qxw,1f06,1r5a,1xjm,1fej,1vzq,1rya,1qgs,1fcy,1iiq,1nsr,1d7a,1uxi,1w4r,
1olm,1szc,1oxq,1u1c,1cx4,1alw,1tpu,1amw,1m9h,1rl8,1dzm,1sbz,1d0a,1oi6,1tuf
,1o2n,1ee4,1inw,1rqj,1vpm,1qmz,1tww,1gql,1rq5,1dtp,1jol,1uaq,1cg3,1o4t,1r3
5,1dlg,1j0e,1xiu,1yqc,1lb6,1e7r,1mep,1w2g,1mt1,1q9s,1gja,1q6s,1f5n,1v0c,1w
96,1nhb,1dm7,1czn,1dtl,1u4s,1apv,1sle,1dw6,1hg0,1u4g,1ovp,1p62,1dub,1awv,1
m6y,1j09,1o29,1fqo,1jcm,1t4t,1m9q,1g6t,1gth,1x8u,1usq,1a7x,1drk,1pmo,1amz,
1ziz,1zx5,1xim,1pfg,1qhh,1i79,1r7y,1r4w,1ody,1hmu,1foa,1v6l,1cxe,1hxb,1xr2
,1df8,1oiv,1mdp,1hdu,1y79,1b8v,1svl,1lx6,1dls,1zdu,1zbc,1ju2,1jz6,1s0j,1kv
q,1ksu,1w5f,1gey,1xdn,1j79,1t7m,1pip,1lbz,1nvm,1mup,1f4x,1tiw,1lqy,1b0o,1n
71,1tpw,1khf,1ozg,1ha2,1i8t,1dzp,1s4p,1sc8,1zxc,1qho,1n95,1o0s,1s9y,1zio,1
qra,1rbl,1mlz,1npw,1kqd,1qpb,1kax,1awr,1qiz,1lbl,1csm,1nse,1l2t,1rb0,1pxl,
1mqh,1g5y,1a1b,1w44,1gsa,1cgz,1w7x,1u92,1j1a,1gee,1lax,1lyx,1g9h,1lby,1lrh
,1dgh,1l5m,1ovg,1o9b,1kze,1f12,1v0j,1dxy,1f8c,1lc8,1gae,1ece,1iri,1fzg,1lj
t,1uxr,1nzv,1wf3,1e4x,1kqp,1akb,1i5s,1g4p,1pum,1kim,1u8x,1bxo,1pfw,1hql,1p
jq,1l7g,1a28,1o9u,1g3u,1ets,1e7c,1tw2,1ndz,1xwk,1lof,1j1r,1siw,1u25,1fnc,1
d01,1hbv,1zzl,1xa5,1tc1,1c1r,1m67,1udh,1xpy,1n2g,1z62,1bn1,1ji0,1pxg,1dbo,
1mg1,1p4g,1a4h,1wci,1mqj,1mfa,1epm,1o2g,1cgy,1mfp,1pzj,1dyj,1yl7,1xdj,1nhg
,1lt6,1k9f,1m83,1uv6,1qz0,1ds7,1n75,1ioz,1bcs,1df1,1sh9,1fsg,1m9k,1lsj,1ws
5,1a4i,1ek6,1f8y,1zw6,1uyk,1nvv,1zj3,1b4s,1ql9,1l7q,1p6m,1m2g,1nuu,1gwn,1u
pb,1v7x,1ogo,1ulv,1a9u,1dah,1aln,1ki3,1i3u,1i9m,1tmt,1bdb,1mx0,1y4l,1e8c,1
cfv,1rms,1sys,1u1g,1yb1,1w6o,1s7u,1a8u,1o1t,1ctr,1k0b,1n4k,1r37,1gaw,1noo,
1xjk,1dm2,1nns,1ux1,1vg1,1grg,1fld,1ig0,1uml,1r8c,1mgp,1qi0,1zm7,1q4u,1q1g
,1x8r,1osz,1ype,1ytj,1xxr,1x1s,1o7a,1wrp,1gdn,1xua,1nhx,1w6s,1u71,1tuk,1s9
q,1jj7,1ndw,1z48,1e39,1djx,1p1q,1pbk,1qw7,1wog,1pr5,1ep4,1l5q,1nb0,1k12,1v
wc,1q4t,1cy8,1pax,1nff,1day,1ov5,1ehl,1dar,1f5a,1hwj,1nut,1tu8,1e6w,1hjw,1
z6y,1wda,1oec,1rsn,1csi,1e9a,1nsz,1uho,1yf4,1l0c,1i0d,1hku,1q6e,1pjc,1q8o,
1pwz,1ec8,1se0,1owo,1w2h,1gjb,1boz,1wnl,1epo,1xlr,1dzj,1xa4,1po0,1qxl,1f9o
,1kaz,1el4,1s9j,1n4g,1bcj,1nnc,1kwf,1a5x,1zvw,1r9j,1y63,1gsp,1egd,1cil,1xk
q,1f8i,1m0w,1o44,1zj1,1f8s,1xig,1c7o,1gmr,1dku,1vwk,1jm6,1zds,1nn1,1s83,1a
gb,1llo,1wam,1xcj,1j37,1w3l,1fkw,1gux,1s1s,1qkz,1r00,1yrb,1trg,1fjs,1c6z,1
c1l,1mhc,1syn,1czc,1dhj,1usc,1oai,1ke8,1itw,1kws,1d1i,1hf3,1m13,1hee,1szo,
1m4n,1r2j,1w8t,1hor,1b37,1rpg,1fzm,1r7x,1w5w,1on9,1w4l,1vah,1o5x,1zkl,1b66
,1oyb,1aa1,1h0s,1rbo,1gvv,1zx9,1evr,1pr4,1ub5,1p2v,1zjy,1gsl,1y5x,1efy,1gw
r,1ou6,1ncw,1gvf,1yq7,1ywn,1i7g,1oyn,1hi5,1y0l,1lwl,1mh1,1ki2,1u4l,1moq,1i
76,1rab,1w72,1h8p,1a4g,1xtb,1aix,1d6y,1dz8,1km6,1wqv,1yds,1akd,1d0h,1s1t,1

x9d,1jg7,1lv8,1t63,1fae,1wdk,1q9m,1swe,1jx6,1ryf,1vfn,1p7c,1t31,1n13,1wuw,
1tjg,1y2b,1t88,1k1i,1gzt,1bhf,1jiv,1kly,1hfp,1guz,1pe1,1qqp,1ohk,1xg3,1fch
,1r8q,1dkf,1gaf,1lt4,1zbq,1t5f,1eei,1zaj,1pzp,1xaj,1jq9,1jge,1n2t,1w73,1qp
g,1ko8,1asm,1jd5,1wlj,1zzb,1n4v,1os0,1vj3,1ux0,1zdg,1nvu,1krh,1ej3,1dpm,1r
iu,1aeb,1aua,1uyd,1v8s,1hrk,1d1s,1nf8,1tok,1wo2,1wdd,1yvz,1cgu,1e72,1mau,1
kjj,1s0r,1tjy,1bai,1hp0,1u2o,1pgt,1frz,1rnd,1fm6,1in5,1o3p,1ide,1n1g,1amq,
1caq,1yc2,1qti,1l5k,1ni4,1p2s,1aid,1ztf,1elb,1z4o,1ik4,1t5g,1b9z,1pd8,1zzt
,1pp9,1cml,1a9p,1rmh,1g0e,1w83,1oja,1i83,1zsb,1pfy,1qqs,1pvn,1lvb,1o2z,1ag
s,1uzf,1qxy,1vl8,1rvd,1qiy,1nqm,1odc,1w76,1f2p,1jrc,1n3z,1hy7,1oha,1p13,1z
0i,1u5r,1df7,1h46,1jg0,1pk8,1gvy,1ogt,1isj,1d7x,1rqi,1oc2,1swt,1oss,1scu,1
a8p,1wwz,1j16,1u8v,1xg5,1afa,1kae,1sby,1pvy,1z8o,1nqv,1cze,1ac4,1zrb,1c8l,
1zy5,1uky,1fih,1uwk,1ka0,1urw,1abf,1g72,1ezf,1gt3,183l,1mz9,1b11,1lt3,1vyw
,1zi5,1tou,1eup,1thc,1syf,1oh3,1fzk,1e79,1fhw,1uye,1xkd,1t67,1q3w,1uym,1ko
5,1tuv,1pj6,1gjr,1ro7,1d8m,1iy7,1kq4,1rls,1f28,1k9s,1k22,1o3b,1igb,1ftm,1d
el,1gvr,1tqs,1c5u,1phe,1f0r,1pb9,1udt,1q23,1ctq,1gne,1loj,1q9w,1li6,1uwu,1
t6z,1koj,1l4m,1i91,1ddv,1uxg,1ogx,1yaa,1v0m,1hnn,1gox,1zm1,1ive,1zji,1ju4,
1oxu,1fdr,1u1i,1ewk,1dig,1c2g,1uxb,1rqp,1ed5,1k1t,1z0d,1naa,1pcj,1m7y,1wcy
,1uwt,1atl,1dll,1h0a,1lbf,1fx8,1dr4,1aev,1f7p,1rkd,1wpv,1d1y,1q41,1uxq,1v2
w,1xld,1he3,1czr,1ros,1dth,1e1n,1gmn,1x1j,1kl1,1qjd,1ln1,1j1s,1jhv,1mqi,1w
4q,1htf,1hvc,1vbt,1rca,1rek,1g02,1ent,1b3y,1q6o,1t9m,1vad,1umw,1ii0,1s6h,1
dqn,1tnl,1gvu,1fxu,1s3k,1syb,1vwm,1npa,1jv1,1yzl,1kp8,1zrq,1jje,1lev,1pt5,
1l5j,1exv,182l,1ch0,1bx0,1dvy,1bwo,1vce,1foj,1iyh,1tq9,1uf7,1u8i,1i44,1pu8
,1n9n,1uxn,1b6j,1px2,1dhs,1w47,1y75,1pro,1xoi,1v2g,1s8g,1n6n,1wxh,1krf,1sq
5,1ujp,1f5f,1egh,1lze,1qiw,1ndc,1ww6,1fgx,1s1p,1fpf,1tps,1flm,1rdn,1lmt,1r
bz,1oc3,1mzc,1snn,1oz1,1m76,1i1d,1pq7,1y55,1men,1f76,1dzk,1lpc,1u1f,1o3n,1
uo4,1j2z,1km2,1fwm,1i82,1l0i,1q6i,1ct8,1s7g,1svg,1ucc,1dug,1tgv,1p6h,1kyv,
1nxg,1fmb,1i1y,1f8e,1rrv,1wt2,1asd,1v48,1w8o,121p,1qx4,1lod,1d09,1sem,1ed6
,1vpv,1md2,1v83,1qs8,1jqh,1wbn,1bsv,1lwn,1ssq,1qd2,1q3a,1j20,1ddr,1fd7,1b2
r,1kvt,1izh,1d2s,1vm1,1yw9,1abn,1jip,1vpt,1f5l,1bzj,1qrd,1mmq,1doh,1yv5,1h
1i,1ecm,1pvs,1k6c,1kzi,1rm8,1nrg,1pjk,1gvo,1h4o,1e6s,1lon,1o0n,1oex,1znl,1
lzb,1fxo,1buc,1m9j,1zdt,1rvx,1w2y,1w82,1uyh,1xp6,1wz1,1zar,1gni,1otj,1e4v,
1h6a,1z3c,1x9q,1l5r,1p1w,1tys,1bto,1zi3,1ney,1cjb,1g63,1ses,1het,1kny,1tdf
,1awb,1umc,1kjv,1smr,1rks,1jhy,1dwv,1o27,1fx1,1w4b,1q5h,1xbx,1n7h,1uu8,1ip
e,1ger,1wlv,1iv4,1znq,1lth,1jdt,1lnc,1bdu,1q0q,1lb5,1cny,1mxi,1b6s,1mei,1k
qc,1xt4,1qw9,1vhn,1m7a,1yxd,1o0h,1upa,1he5,1hdq,1v0l,1kvo,1q6g,1w2d,1w78,1
ne4,1izi,1p4h,1qlu,1hyo,1rc0,1isx,1qcj,1o5a,1a9t,1aj6,1mt9,17gs,1t1r,1rxo,
1j1m,1t27,1dwq,1ebg,1oge,1ugw,1tsu,1j6w,1iol,1inf,1xh2,1mez,1aia,1sg6,1cg1
,1xtp,1msm,1lne,1pk9,1g6i,1w5y,1d8a,1u3r,1qvo,1evh,1cla,1ppk,1xqc,1mtw,1xp
c,1px7,1qpe,1f2u,1rk3,1uhh,1mew,1uxx,1it0,1u8h,1nhp,1z4j,1aet,1yrd,1qqm,1y
5u,1y3x,1wcc,1jwk,1kqz,1g60,1cci,1wur,1i12,1fzo,1um0,1ypk,1c7f,1zz3,1b5e,1
jkl,1q2e,1znx,1dog,1njj,1zh6,1fm7,1sdm,1qrr,1ffi,1ny5,1nz7,1db1,1r31,1ulg,
1dcn,1ngg,1fv3,1ghe,1lwx,1lcb,1t4u,1fvx,1ez4,1m1o,1hyh,1gm8,1s7v,1xkb,1lrj
,1xbt,1cgl,1jkh,1p1o,1tjw,1bd0,1od9,1qqn,1kuy,1x9i,1uvb,1yni,1ae4,1c21,1u7
w,1yzu,1b63,1idb,1tmk,1njf,1tqu,1n2o,1i0z,1a1n,1xu9,1kec,1ec2,1qy4,1yda,1e
xx,1gfy,1b9i,1xlc,1nw4,1aqv,1gzw,1v7w,1c4u,1ajp,1k6x,1hw9,1pq5,1c5x,1qjc,1
suw,1pcw,1fu7,1fgh,1p6k,1ose,1qv7,1ize,1ym4,1rnm,1lvg,1p5z,1h73,1qf2,1esm,
1s63,1gr2,1p2u,1pi4,1g7q,1fel,1g67,1o36,1rnt,1qd1,1ftx,1jf2,1tw5,3vp6,3ri1
,3wk3,3rbu,3zst,3seu,3t34,3ryc,3vo2,3u8o,3uet,3vhh,3rs6,3zk6,3v5p,3zl2,3wk
g,3sea,3wst,3zc6,3t25,3ruv,3u15,3tvu,3sud,3unj,3wqw,3tkm,3x1y,3mbc,3t2w,3v
1m,3pcn,3wg8,3v1y,3vkx,3qxt,3qh2,3lk1,3t96,3wc5,3rkr,3zlv,3qj9,3r37,3t70,3
x44,3qnd,3vyw,3wpm,3uec,3rlb,3qx5,3wd6,3wb4,3vw9,3utc,3t2k,3un3,3wix,3wir,
3rwp,3q3o,3t9v,3ttn,3w6x,3wv7,3zra,3wxl,3t7k,3suf,3w7t,3krb,3qzy,3sqg,3vs1
,3qm1,3tzi,3tdw,3smi,3zxe,3t9a,3vz3,3x13,3n9q,3tdt,3wk9,3rid,3sp9,3u18,3qh
w,3u3e,3skk,3s7o,3x02,3t0w,3rdk,3o5a,3ub7,3vdb,3ujd,3x1w,3v35,3ufp,3wv8,3t

3y,3rv5,3uth,3x0l,3thr,3t6e,3uhm,3r3b,3roe,3x1m,3wn1,3r51,3rsc,3wn0,3ues,3
x0v,3qhd,3oro,3vnj,3wgm,3tlz,3u3h,3ryb,3zt6,3sem,3ryz,3uay,3wr4,3uov,3uj9,
3wec,3veh,3uoy,3s0d,3pcj,3tnz,3tfp,3t99,3s53,3rhq,3qxc,3ufs,3vex,3m1k,3s2v
,3wuw,3vuz,3x0d,3r1v,3vv1,3ryx,3sii,3unk,3v8z,3wg1,3w0s,3v3l,3zzv,3uyr,3zc
7,3vk5,3ufu,3t0x,3t10,3std,3rhj,3tax,3qpp,3vwx,3zns,3qi4,3t9u,3wud,3sfu,3w
eo,3wd9,3zya,3kv4,3v8m,3sch,3w21,3wzp,3rlr,3rdv,3t9h,3sx5,3w9k,3vg3,3qsp,3
tep,3uix,3vqp,3rs5,3rwm,3wqt,3zv6,3qvb,3r9v,3ta0,3v51,3zbz,3tsq,3usg,3pkg,
3res,3sbd,3zlr,3ty0,3sxe,3t2e,3znl,3r0t,3wwx,3qp8,3vw8,3rwe,3uja,3kjr,3rm4
,3r3v,3ssn,3wuc,3wr9,3zsn,3wca,3n9n,3x1x,3rt7,3x11,3viv,3uj7,3upk,3vs3,3ue
4,3vpd,3vt3,3t4t,3qlm,3r7x,3wz8,3qww,3zds,3zt3,3w6g,3vh9,3rre,3zp2,3r4s,3w
to,3qgy,3six,3qkl,3zxk,3ww2,3zcm,3s7l,3rik,3zep,3u8x,3wop,3v1s,3szo,3t0z,3
tne,3u4s,3zp7,3t3d,3u4l,3riy,3zlm,3n9o,3uxl,3zbt,3rz8,3r0w,3v9v,3uaw,3qvk,
3t01,3onw,3zlb,3umv,3rkz,3wdx,3spk,3x26,3u5l,3t5m,3t7n,3r5j,3r1p,3u11,3vzs
,3qpw,3zps,3tk1,3rde,3um5,3tll,3t7s,3v8n,3sfx,3wf9,3td4,3zt4,3tu1,3qss,3ws
j,3svp,3wl8,3p3n,3vg6,3vq7,3vze,3sf0,3tki,3wxb,3s6z,3r96,3s6t,3uhl,3wlv,3p
k4,3v5g,3wk7,3vqs,3tud,3ta2,3uxm,3t3g,3u2w,3ucl,3zgf,3srw,3tf4,3ttj,3zrm,3
zjx,3r32,3u2m,3vry,3uwb,3w0p,3sxf,3zzh,3rv6,3t2c,3t7m,3u3n,3zo4,3vmi,3v16,
3qhm,3zc3,3rcw,3ry9,3t5g,3v0m,3u8w,3rlj,3zzk,3tzf,3qj4,3uuo,3uo9,3voh,3qin
,3uxk,3zsy,3r6t,3utd,3uwo,3r36,3rf4,3tcz,3w0i,3u2l,3zs7,3v0l,3rlf,3wmp,3zu
2,3t9d,3rs3,3qo8,3r4z,3vt4,3t0k,3udl,3max,3ukd,3ukh,3wt6,3wkb,3sj1,3pm5,3z
wt,3sz9,3s70,3qyo,3ttc,3zbf,3rap,3u8t,3uzp,3shc,3u5k,3t6f,3v8p,3u2k,3t65,3
rzn,3os8,3tkh,3quo,3udm,3we4,3vfe,3zhu,3vqe,3ztx,3zgg,3zmk,3rgq,3wz6,3t84,
3rrz,3zml,3r3c,3tct,3qfu,3zsx,3szj,3u5y,3s56,3qft,3qt6,3wmx,3uli,3srq,3vha
,3qvp,3sfc,3sg9,3uft,3vi7,3vhd,3zy5,3zsr,3u2v,3tyz,3tut,3r9e,3uo4,3wci,3tq
0,3zm9,3qpv,3tti,3wk6,3tle,3wn4,3qgi,3wg6,3tlh,3ugc,3zgv,3wv1,3t4o,3s68,3z
kl,3uex,3t31,3wf5,3zux,3v49,3zv5,3usp,3w6f,3w54,3tyv,3vmm,3uo0,3vnt,3nwb,3
sap,3qur,3zoe,3ryd,3v91,3uxh,3ubp,3to3,3wgc,3vtb,3uv7,3rs7,3uzj,3sxn,3sa0,
3ru0,3v4u,3znm,3wdv,3utf,3rt9,3urk,3wue,3udh,3vpc,3zo7,3sie,3v2p,3vqc,3vxs
,3s1g,3vt7,3zcl,3lgr,3tl1,3woq,3nvv,3ufq,3vq4,3vc3,3t4s,3s7m,3szg,3tfn,3v8
w,3zvx,3wh2,3rhr,3uuc,3plm,3w37,3wqm,3sls,3zet,3zvy,3thb,3rgz,3vot,3zy3,3r
sg,3s44,3r8a,3zht,3syr,3sou,3t77,3zlk,3ubw,3k5k,3wy1,3u3z,3t8n,3r4b,3wdl,3
qic,3r1n,3wau,3t54,3s0e,3zwe,3rlq,3ver,3twd,3x1j,3sym,3sk2,3t14,3vby,3sni,
3w29,3ked,3tky,3up0,3was,3t4l,3sxb,3zmg,3ug5,3sow,3rfv,3t5z,3v36,3sjf,3vm7
,3rss,3v7t,3tif,3qjm,3tyd,3ry8,3wqe,3srv,3ti3,3qxy,3tp0,3zov,3pci,3tiy,3t3
u,3v3m,3tem,3tgp,3qps,3ui7,3vt9,3ti8,3utq,3ove,3rsj,3zyx,3ses,3x0p,3r5n,3p
cf,3vgw,3tdc,3vxr,3qry,3srr,3sl4,3zc5,3lfm,3rl3,3stm,3qf7,3te5,3tkg,3skh,3
kal,3vet,3qhr,3vrw,3t2z,3w0h,3x0n,3r34,3wab,3u4o,3ovr,3vn2,3sdu,3u3r,3rx7,
3w27,3wjo,3veo,3sxd,3qrc,3pch,3rsl,3w0g,3sxs,3qho,3qtv,3pkk,3szl,3rv7,3r19
,3q3n,3th5,3ucj,3vbd,3w5n,3s6a,3uu7,3urb,3tge,3tik,3k5i,3wha,3wd3,3t3c,3ti
6,3min,3zbs,3zm6,3wsf,3ude,3r3f,3two,3wvc,3vg2,3r18,3qg2,3zqa,3zx4,3qv8,3p
le,3rk1,3t4k,3t02,3vvc,3t5u,3wlf,3qfx,3rta,3vso,3s3v,3qxj,3ml1,3qp1,3s1u,3
re4,3mz6,3x0k,3tdb,3zoa,3rm9,3r4n,3x2v,3vey,3x2f,3rul,3qlz,3w6c,3t12,3wk1,
3qfj,3w0j,3t4y,3w5e,3tu7,3u96,3tbk,3uzy,3wqr,3tl9,3ua9,3vmf,3wk0,3w0r,3uni
,3v1k,3tyl,3u10,3u7s,3plg,3uh1,3t4q,3tia,3v2u,3tm0,3vru,3vhc,3tg4,3wug,3wa
w,3zeu,3pk5,3rdq,3wel,3v4a,3ujr,3udz,3wxm,3zwi,3zsz,3tmk,3vqd,3rz3,3w5t,3q
mk,3wdr,3qvu,3si3,3ust,3r6w,3zbx,3zr6,3r3z,3qvl,3qtp,3r00,3pk6,3v0h,3ogq,3
wlu,3wog,3zsh,3vbt,3w2f,3ziu,3w9z,3vbn,3zku,3sqp,3pcm,3wyw,3vbp,3w69,3si2,
3ubd,3ref,3v4s,3wio,3w3j,3zlq,3rnn,3vbv,3qow,3njz,3r3x,3td8,3t4n,3utg,3r95
,3qpn,3vri,3sae,3udr,3zso,3wav,3krp,3x14,3vvy,3udn,3t7o,3rab,3vp4,3zt7,3zq
t,3qti,3w0q,3zze,3whc,3wy2,3qvv,3u92,3sxi,3qkm,3vjk,3wh0,3ufr,3wk2,3wtl,3q
iy,3rc5,3vtr,3ttz,3qgf,3zdh,3vw1,3t1v,3rte,3zo1,3set,3wzd,3t7a,3tjc,3qxv,3
war,3r9f,3urq,3rry,3t95,3sz1,3zfd,3uaz,3r91,3wzn,3uax,3v5l,3zlw,3ku2,3l9x,
3wcm,3t9c,3rt8,3vbw,3wf8,3r0i,3vbl,3x0q,3ret,3tg5,3qxx,3qkk,3suc,3sxc,3su0
,3vg5,3tku,3u3u,3si4,3kpj,3sm0,3tkl,3zj0,3uph,3sao,3u4x,3zme,3vtf,3vsu,3sw

e,3tiz,3sha,3sl0,3se2,3we8,3x0m,3nkv,3wbd,3zcn,3w25,3vkj,3s7a,3rex,3zxr,3s
ex,3t9f,3s1s,3r6u,3s45,3qe8,3v1x,3pca,3tyx,3wjx,3v1h,3w2e,3rnm,3vt6,3nkt,3
u6j,3wqq,3qp6,3su6,3vi2,3w7s,3qt9,3uag,3r5y,3zq9,3syu,3n9p,3shz,3uol,3wi2,
3rdo,3w7w,3zw7,3tah,3qlw,3wcj,3md0,3t4h,3ung,3znp,3th8,3ug2,3qn1,3vew,3udg
,3r5w,3ves,3w2w,3uyl,3sru,3se5,3vzc,3rs0,3zw0,3zyf,3u5s,3qxh,3tfi,3zsw,3um
w,3ubq,3zt0,3tub,3ryj,3zpg,3zxv,3vbi,3ub9,3puq,3st6,3w5p,3qwf,3vqb,3uud,3s
j0,3vma,3u3o,3vhu,3sx3,3qh0,3t1f,3pcg,3ujs,3s9t,3sje,3rsq,3sdv,3svq,3rhl,3
t2y,3teg,3uew,3zvs,3qfa,3v7w,3vq6,3wve,3zt2,3svj,3zqu,3t7t,3wh1,3wur,3rog,
3t82,3uwk,3t3q,3t08,3v1n,3rrj,3s6x,3sfg,3wf6,3suu,3rz5,3tt4,3w3l,3uh0,3wle
,3zdg,3woo,3wcc,3q2a,3rga,3ttf,3qvi,3vhp,3zu4,3rtb,3zgj,3vss,3tkw,3qvh,3ru
g,3sg8,3src,3vgm,3t26,3vhv,3t8a,3w2h,3ug4,3qvr,3t1o,3wg3,3vbx,3unc,3uyk,3v
kb,3u40,3t0b,3vgl,3r42,3vhi,3rwi,3vd4,3tv1,3rz1,3tao,3zpt,3urn,3wxo,3t94,3
vca,3pck,3sv2,3up5,3qng,3wz7,3tfu,3smj,3v9t,3rv3,3vi0,3thj,3sm1,3zwn,3ua8,
3qi9,3vbj,3uj8,3zkk,3zu5,3zt1,3stk,3s9k,3uwv,3w5q,3u14,3ts4,3uox,3ww1,3szw
,3r1o,3qiz,3uvp,3s7d,3wzo,3tc5,3wd1,3qfr,3r3d,3vkc,3rvg,3r02,3qwb,3qtf,3vq
a,3vd9,3slz,3sjh,3ucc,3zhq,3t1l,3uw5,3sju,3qup,3quy,3uv3,3tlj,3wze,3kv5,3w
is,3wtj,3ufo,3vtc,3s0b,3u7y,3vbk,3sy4,3sur,3rtf,3s43,3uyw,3pr2,3s4q,3qx8,3
t27,3su1,3tw1,3zqe,3qqa,3zl1,3sw8,3zxz,3u8r,3v7x,3ssm,3znr,3ril,3r7q,3sm2,
3rm8,3qgh,3ujl,3uh4,3nnf,3t9e,3t2q,3pu8,3t29,3wnz,3sl6,3zjq,3zws,3uxd,3qk5
,3rl2,3upv,3rc1,3ri3,3wl9,3zs5,3swq,3zos,3ufn,3u3l,3rhx,3tkc,3rc9,3zll,3ru
u,3lkz,3pjk,3qu2,3tnw,3vi5,3sp5,3ska,3url,3w51,3vph,3wy4,3zu0,3syi,3zew,3w
fd,3wor,3rn6,3zr7,3uod,3zfc,3u2u,3mnu,3uj6,3r33,3ujb,3rtd,3ut6,3qpo,3sbi,3
rwo,3qxq,3v0w,3rof,3sso,3tyn,3uyt,3zbn,3qoa,3rj9,3sl1,3tmz,3wax,3rwg,3vjl,
3rsv,3ru2,3s8o,3sus,3s2z,3vxn,3wtm,3tlb,3vu2,3uq8,3sdg,3ryi,3sko,3rtg,3t88
,3zki,3rgg,3tzm,3sj7,3s2y,3wya,3wlb,3rso,3t0g,3nwe,3vg4,3tm4,3uqf,3r52,3t6
4,3ric,3rum,3qgt,3su9,3qh8,3ti1,3r92,3qfs,3w7u,3v8s,3txo,3w32,3rin,3wyc,3u
b5,3vnk,3r35,3sjk,3rme,3zu6,3v1p,3vvd,3pli,3rg9,3szb,3wch,3s9y,3veu,3upi,3
vq2,3r0y,3zkn,3s0k,3w8x,3qpg,3zk5,3zr0,3ryv,3s5x,3txj,3v1v,3qtc,3qto,3vi8,
3v7u,3r3w,3v8t,3ubm,3vmg,3zv7,3tty,3soi,3r16,3reg,3x27,3zg6,3vkd,3su5,3qt8
,3udv,3s5w,3u5j,3sjg,3rbv,3uc5,3mk1,3qox,3zvu,3vhz,3vc1,3wbz,3pcb,3wvm,3pl
j,3t42,3rs8,3zec,3wyq,3v7y,3rsr,3qfd,3w68,3u2d,3rk0,3w0o,3wv6,3vhe,3znn,3s
u4,3szf,3rmv,3ua1,3sax,3v0p,3r3o,3uoz,3rmk,3r9g,3s2p,3s2u,3pkh,3tpp,3zyr,3
ukr,3vhw,3v9l,3zly,3vo3,3zbu,3pcc,3vez,3tv3,3sr6,3t6d,3t0f,3w7f,3rs9,3r5t,
3zoh,3wol,3sw2,3vmw,3wmc,3suv,3tfc,3zdw,3wtn,3w1w,3vq9,3ttm,3t8w,3w1v,3v4f
,3ran,3v2n,3qp2,3zh4,3w6d,3wbb,3tka,3vka,3zwo,3vjn,3uxe,3wb2,3sxg,3zvv,3vx
p,3su3,3ucb,3qi3,3wjp,3w0n,3kj7,3tfj,3vg7,3ty3,3wkx,3t4v,3zbp,3sxr,3zkp,3q
e2,3vf4,3qxs,3str,3oyw,3tic,3tjh,3tm5,3ubj,3sff,3uw4,3vv5,3ru1,3udy,3tza,3
qwv,3vws,3wzk,3uev,3nrz,3zfz,3uww,3qri,3una,3tux,3rj5,3vjm,3tvq,3t2t,3kti,
3tsk,3rox,3wd4,3rz7,3v99,3zwd,3rfa,3u4r,3vv6,3sut,3zo2,3vt8,3sfy,3srb,3s1w
,3tvc,3x05,3t85,3u31,3wi1,3wyj,3zod,3vf3,3u81,3uvd,3vq8,3sr5,3s8l,3wyg,3si
g,3ucd,3qgw,3v4t,3u57,3rq7,3rtw,3siw,3qsb,3sn7,3s0j,3upy,3tfl,3u1t,3wvr,3s
fv,3pkf,3sz0,3zp4,3s3g,3vse,3vp0,3rfx,3vq5,3ro7,3tfq,3uzz,3pf7,3vv7,3u1y,3
wrb,3vsv,3tii,3w3n,3twc,3zyu,3tyk,3ve4,3tn7,3vrv,3tpt,3t3e,3wqs,3u06,3szu,
3tw3,3vrt,3ser,3tye,3zbq,3ryy,3whm,3qyy,3tur,3wd2,3zls,3zky,3vj6,3tlc,3v0q
,3vs6,3zt5,3zhv,3vg1,3w9f,3zks,3r4p,3rn8,3vc2,3zy6,3zln,3u3k,3wjw,3ube,3t3
v,3wqv,3sel,3sx8,3m2r,3wv2,3u6b,3s7b,3rux,3qj0,3u9z,3s04,3t09,3uz5,3s8x,3v
iu,3wn2,3ov1,3w8z,3tyo,3w5r,3te7,3uim,3sis,3r75,3voz,3v2q,3r17,3w2g,3uh2,3
zog,3x2w,3vqr,3zsv,3sxa,3t2d,3r5r,3w8s,3mmg,3wka,3wat,3vev,3qyz,3v1u,3t3z,
3u3m,3zy2,3vp1,3sx6,3zno,3u2c,3ql3,3t58,3o0f,3swc,3w2i,3rr4,3rup,3sp6,3k34
,3rsx,3upz,3u9q,3vx3,3uwz,3zcb,3t4j,3sue,3u9h,3tdv,3zbr,3tuc,3qrj,3rno,3vv
0,3we0,3wcz,3skm,3ukf,3rop,3rc7,3qkz,3rtp,3sew,3qlx,3wt5,3r6k,3kt7,3wb0,3z
f8,3tw6,3s54,3wy9,3zlx,3wwc,3rll,3qvf,3vnl,3shr,3svl,3u6h,3t03,3u6k,3v8q,3
wzq,3sjx,3zum,3qip,3sgz,3upr,3te4,3sfi,3sjt,3ute,3wl7,3qyl,3rcb,3t60,3rs2,
3s0n,3w3k,3zei,3upx,3r1k,3wdu,3tb0,3qv9,3ti5,3zuw,3tay,3vdq,3qkt,3upj,3v9y

,3rhk,3sdt,3rz0,3wiy,3v5t,3wcl,3vf6,3wbg,3zo3,3zm4,3ske,3pkl,3vo1,3t8v,3ti
4,3q14,3zwm,3rv9,3vnm,3w33,3vd8,3whb,3udd,3rmw,3zcw,3zqz,3wv3,3w5h,3udp,3s
m8,3wk4,3u4c,3rsf,3suw,3ud5,3qgz,3vc4,3uie,3r7o,3sy0,3qyw,3lhs,3t1m,3pk2,3
ueq,3qx9,3wcs,3vko,3qse,3s0i,3rcj,3scm,3r4m,3u7q,3zx5,3wyf,3ufl,3to9,3w4i,
3u1b,3rc2,3zl8,3zof,3wf7,3tsu,3td5,3rew,3qt7,3tea,3ztv,3vw2,3wjz,3vrz,3qu9
,3v8r,3x0y,3zi8,3uqg,3rl1,3xim,3qxm,3qun,3ovq,3uan,3rc3,3nw9,3rs4,3uo1,3zr
r,3st5,3t7v,3ulk,3sqy,3wkd,3x0o,3wjr,3vhn,3ur0,3udj,3uha,3tnf,3v80,3rrb,3u
ua,3quz,3qxg,3sey,3sv9,3ql0,3r5z,3su2,3u9d,3swg,3zw2,3rf5,3tjv,3qio,3ty5,3
qk1,3zhr,3roc,3zpu,3t50,3wjq,3wiz,3sr4,3zu3,3s5z,3rjy,3t1a,3vt5,3vzd,3r8i,
3ux8,3zsg,3ujc,3vrj,3plh,3sh3,3t1t,3rw9,3x12,3v5w,3x01,3zrl,3rgf,3w28,3rhn
,3tel,3r3a,3qkw,3snl,3ojo,3wr3,3ww4,3zrb,3t9b,3szc,3s5y,3tib,3zsq,3wdk,3sw
d,3wvs,3uzx,3qrk,3ufw,3qv1,3l4p,3ufv,3vzb,3qhn,3r77,3rv4,3r9c,3r1z,3vpq,3v
78,3wr7,3wc7,3vf9,3zup,3qwc,3wg4,3rt4,3pcl,3rtc,3zia,3unn,3sj4,3r7f,3utu,3
qfc,3ta1,3ko5,3rlp,3wrc,3voi,3oy8,3zoi,3rix,3kwy,3sdr,3t3r,3up3,3ufh,3sma,
3rx5,3zhz,3vbm,3vjh,3she,3u9y,3sli,3wxc,3zsj,3wdy,3t0d,3tgg,3sx7,3rho,3w5j
,3w26,3wt7,3v52,3vbq,3vhm,3vxm,3rv8,3rem,3x1z,3sww,3wsg,3zgc,3rc0,3rl4,3vx
e,3w9r,3uje,3sit,3ujf,3sbz,3rhc,3q3m,3tyc,3swa,3wdm,3to7,3zi0,3wbf,3zxh,3w
cf,3u2o,3nl1,3wyp,3zzf,3vh8,3r04,3k3g,3vcp,3ur5,3u6w,3qq4,3wk8,3vcy,3qkd,3
pce,3sbh,3uvm,3vqq,3lf2,3v66,3zmr,3vf8,3sj6,3zoc,3uzw,3thh,3srs,3san,3qp4,
3uwu,3wkc,3pah,3uv6,3zsi,3run,3wmq,3s8n,3rrf,3zho,3vv8,3wjy,3up2,3tcg,3x2u
,3u6i,3pur,3wkh,3zuy,3zhs,3vgf,3rwc,3zzm,3spf,3vy6,3qwi,3rhz,3qnl,3ueu,3wb
l,3tdh,3wgt,3tym,3w07,3wki,3uyy,3zyc,3zrk,3rus,3qut,3sug,7ao3,7jo2,7kj9,9n
se,7aax,7hvp,7a5q,7ao2,7k4z,7a2h,7ao0,8icd,7ak3,7jo1,7est,966c,7khv,7cqw,7
c7f,7jnt,7jh2,7gsp,7a4v,7k5g,7ju6,7kjf,7a77,7d5l,7adr,7ji2,7kjd,7gss,7bp6,
7c39,7a06,7k5f,7c66,7tim,8nse,7khs,7bor,7a79,7ju5,7k6z,7cw5,7jdw,7k6r,7job
,7cuf,7ct4,721p,8xia,7bq4,7c2i,821p,7bvy,7aay,7clz,7bur,7byw,7cgv,7upj,7by
s,7cqq,7jhi,9icd,7c5e,7bvz,7a1b,7ap4,7bqv,7jjq,7c34,7a0p,7joc,7cbg,8cpp,7j
id,8xim,7bwl,7bvj,7taa,7jvk,7chj,7b08,7c5f,7c6h,7ci7,7bwc,7c6i,7jhd,7cib,7
a22,7k5h,7jv8,7c8g,9ldb,7jvl,7a0c,7bqo,7ady,7c04,7jo0,7jnz,7kdr,7abp,7aki,
7c3h,8atc,7bvq,7jww,7bq1,7kjg,7jw4,9xim,7bq3,7amd,7k99,7a2a,7c60,7bpz,7c7g
,7jnv,7cbh,7aau,7bp4,7bql,7btc,7a5x,9jdw,7bp1,7c6b,7kdz,7c6m,7a49,9ldt,7ap
1,7a99,7cpa,7jvn,7ci5,7bzm,7kdw,7cpp,7k6k,7k4u,7btd,7kdo,9aat,7c67,9gss,7b
t5,7cm1,7a14,7kj8,7afw,7bqk,7cuc,7k2u,7c3l,7cqu,7cyw,7c3j,7c6t,7c7o,7c7n,7
kbg,7cel,8a3h,7a13,7c6n,7jo3,7cug,7a5m,7a1z,7k9a,7cu2,7k6t,7k4x,7c3c,7jwf,
7byv,7ab1,7cne,7c3n,7ap3,8gss,7cu1,8kme,7c69,7c6j,7ab2,7bp5,7cv2,8dfr,7k6x
,7ci4,7rnt,7byx,7enl,7bqu,7jws,7k6s,7c6l,7c7h,7buj,7jnw,7aaz,7jvm,7cwd,7cm
c,7kme,7brn,7c6k,7k6l,7ago,7cie,8cpa,7a4b,7d7u,7a04,7coj,7bpy,8gpb,7d2a,7c
3d,7jwv,7cic,7jvu,7c3i,7bx9,7ckj,8cat,7jwt,7c49,830c,7a78,7cbi,7a4c,7c68,7
cqn,7c2v,7jra,7avq,7c6r,7dk8,7c6w,7jvw,7c38,7cp7,7k5e,7a16,7cid,7a12,7k6i,
7k4q,7c4a,7ayn,7jnr,7ap2,7acu,9cgt,7req,7dfr,8cgt,8ruc,7jis,7jwu,7std,7ak0
,7btv,7bq2,7a15,7bzj,7bpc,7kje,7ci9,7jh4,7bq0,7cyx,7afv,7c6g,7cdx,7k6u,7bo
p,7cp6,7cat,7byt,7k6j,7c8f,8hvp,7c70,7jnx,7bvt,7cqx,7nse,9gac,7jtm,7ao1,7a
76
